# Enantioselective Syntheses of Wickerols A and B

**DOI:** 10.1021/jacs.3c00448

**Published:** 2023-03-08

**Authors:** Jonathan Chung, Joseph S. Capani, Matthias Göhl, Philipp C. Roosen, Christopher D. Vanderwal

**Affiliations:** † Department of Chemistry, University of California, Irvine, 1102 Natural Sciences II, Irvine, California 92697-2025, United States; ‡ Department of Pharmaceutical Sciences, University of California, Irvine, 101 Theory #100, Irvine, California 92617, United States

## Abstract

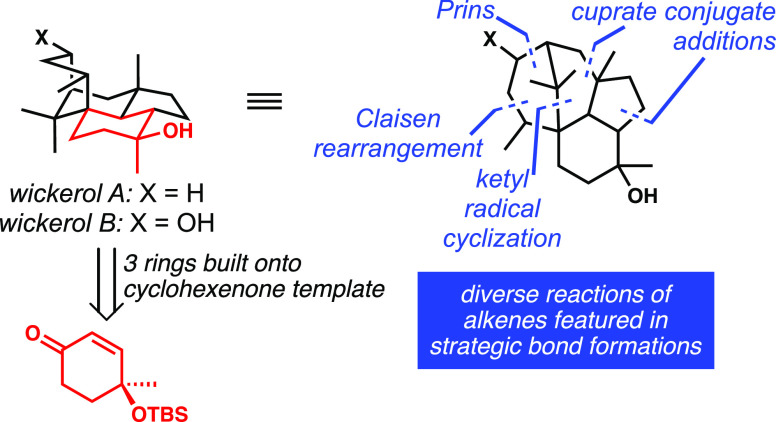

The evolution of a successful strategy for the synthesis of the strained, cage-like antiviral diterpenoids wickerols A and B is described. Initial attempts to access the carbocyclic core were surprisingly challenging and in retrospect, presaged the many detours needed to ultimately arrive at the fully adorned wickerol architecture. In most cases, conditions to trigger desired outcomes with respect to both reactivity and stereochemistry were hard-won. The successful synthesis ultimately leveraged alkenes in virtually all productive bond-forming events. A series of conjugate addition reactions generated the fused tricyclic core, a Claisen rearrangement was used to install an otherwise unmanageable methyl-bearing stereogenic center, and a Prins cyclization closed the strained bridging ring. This final reaction proved enormously interesting because the strain of the ring system permitted diversion of the presumed initial Prins product into several different scaffolds.

## Introduction

**Figure 1:**
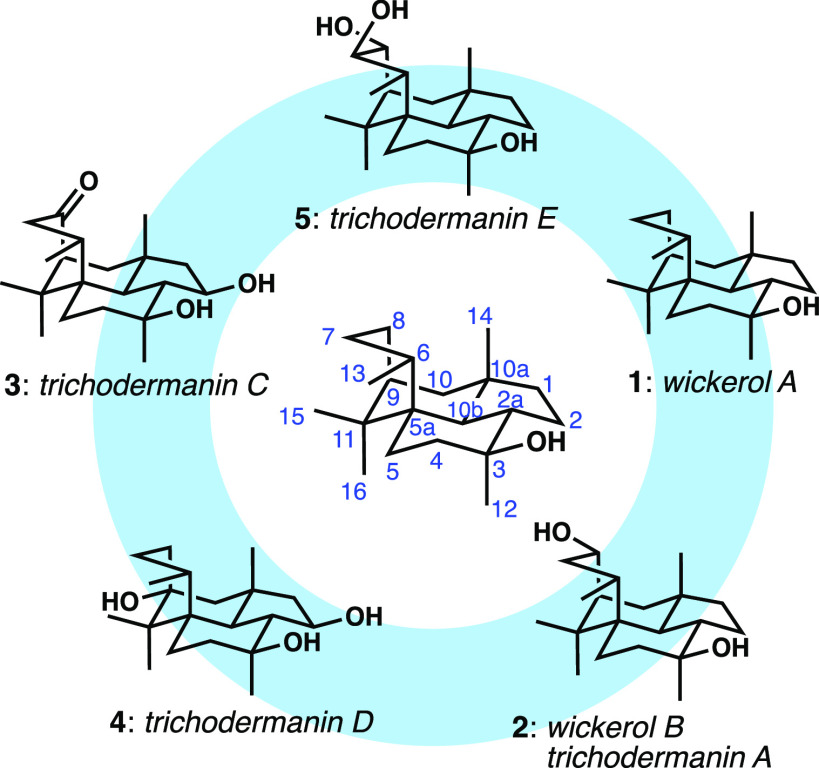
Wickerols and trichodermanins, and the numbering system put forth by O̅mura.

In 2009, O̅mura and colleagues reported the structure and potent antiinfluenza activity of wickerol A (**1**, Figure 1, at that time called wickerol) in a patent; this tetracyclic compound was isolated from a strain of the filamentous fungus *Trichoderma atroviride*.^1^ A closely related structure—differing only by the addition of a second hydroxyl group—was reported independently the following year by Qin and co-workers, and was given the name trichodermanin A (**2**).^2^ In O̅mura’s first nonpatent publication, both wickerol A and B were reported, and the latter had an identical structure to trichodermanin A.^3^ Other, more highly oxidized trichodermanins from a different species of *Trichoderma* fungus were reported by Yamada and colleagues in 2017 (**3**–**5**, and others not shown).^4,5^ Wickerol A demonstrates potent antiviral activity against influenza A H1N1 strains A/PR/8/34 and A/WSN/33 with IC_50_ values of 0.07 μg/mL; the diol wickerol B, however, showed depressed antiinfluenza activity, with an IC_50_ value of only 5 μg/mL against the A/PR/8/34 strain.^3^ Owing to the small quantities of these compounds isolated from natural sources, an expedient synthetic route to the wickerols would permit more in-depth investigations of their biological activities, including potential mechanism of action studies.

At the outset of our efforts, trichodermanins C–E had not been reported, and the only studies toward the synthesis of these compounds came from the Richard group in the form of the construction of a stripped-down tricyclic scaffold containing the three six-membered rings.^6^ The wickerol architecture poses some obvious challenges to synthesis, most notably the congested bridging six-membered ring. The boat conformation of this ring, as shown in Figure 1, follows from the X-ray structure reported by the O̅mura group,^3^ which clearly shows how this conformation alleviates the extreme nonbonded interaction between C7 and C14 that arise when this ring even approaches a chair conformation. This conformation keeps C7 and C15 in close proximity, highlighting the little room available to maneuver in the installation of the bridging ring. The 1,3-diaxial interactions between the bridging ring (C6 and C8) and the axial C14 methyl group further exacerbate the situation. Additionally, three quaternary carbons (two of which are stereogenic) and the *trans*-hydrindane ring junctions are each potentially problematic.

During the course of our multiyear effort toward the wickerols, two successful approaches were reported. The first was by Trauner and Liu in 2017, who successfully applied a sterically demanding Diels–Alder cycloaddition and a remarkable intramolecular enolate alkylation to unite two neopentylic centers, ultimately arriving at wickerol A in ∼26 steps.^7^ The second was accomplished in 2020 by Gui and co-workers who, by judicious use of the chiral pool (sitolactone, a microbial degradation product of steroids), as well as cleverly orchestrated C–C bond-forming events, were able to access wickerols A and B in 16 and 15 steps, respectively.^8^

This article describes the development of our synthesis design that ultimately leveraged the versatile reactivity of alkenes, which are used in virtually all key C–C bond-forming events (see below). During the implementation of our strategy, we unearthed fascinating trends in conjugate addition stereoselectivity, gained valuable insights into reductive ketone/alkene cyclizations, and overcame significant challenges in the context of C6 methyl group installation and the closure of the bridging ring via Prins cyclizations, each of which is a transformation built on the utility of alkenes. Many of these lessons are expected to be of broad value to synthetic chemists.

## Results and Discussion

### Synthesis Plan

**Scheme 1:**
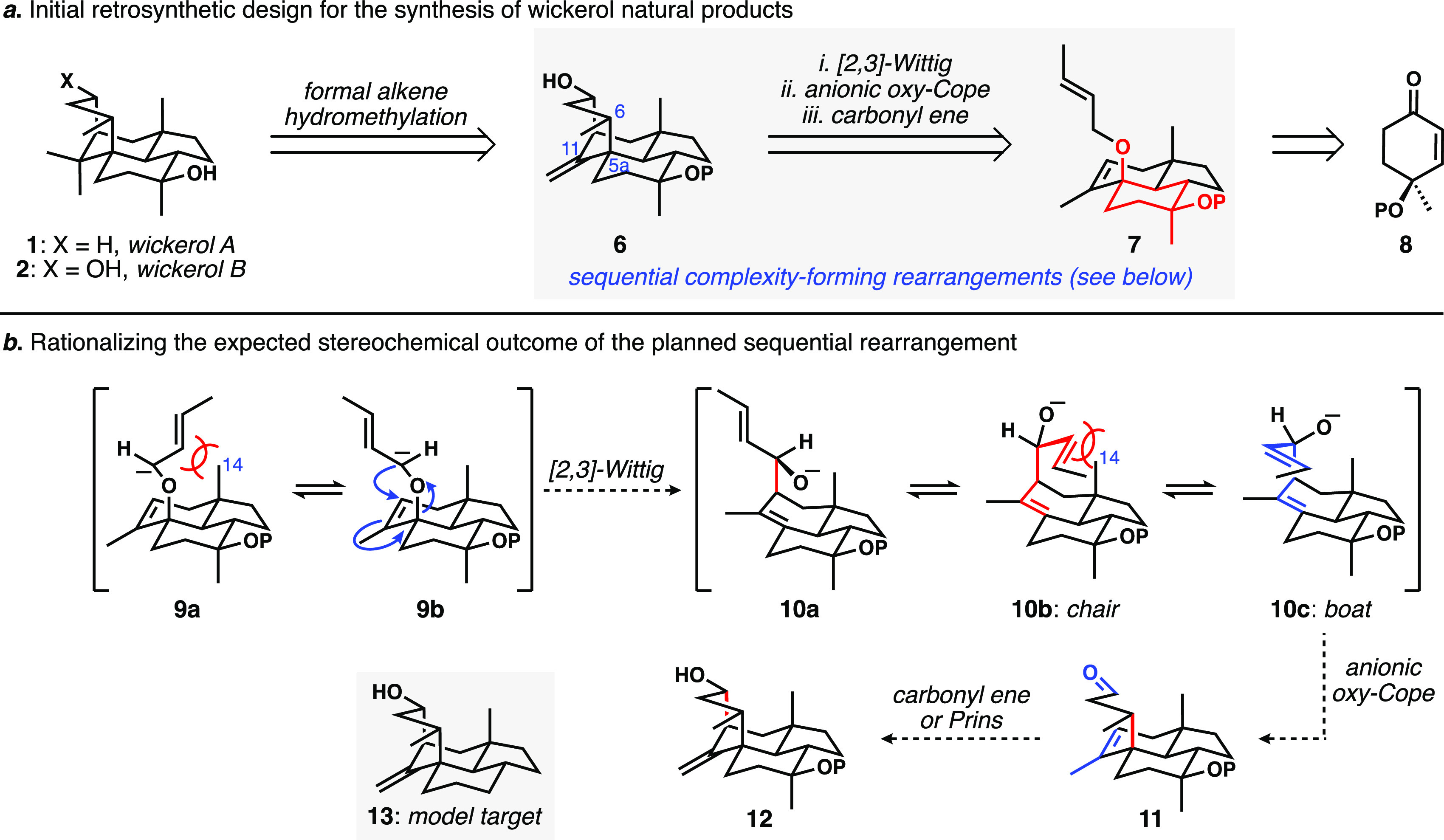
(a) General Synthesis Plan for the Wickerols; (b) Details of a Wittig Rearrangement/Anionic Oxy-Cope Cascade

We initially planned a pericyclic cascade of [2,3]-Wittig rearrangement/anionic oxy-Cope rearrangement/carbonyl ene reaction to install the key bridging ring (Scheme 1), wherein the last step could equally be a Prins cyclization. In more detail, we considered that the more potently active wickerol A might be generated by selective deoxygenation of wickerol B, which would arise from formal hydromethylation of the exocyclic alkene of Prins/carbonyl ene product **6**. The key insight was that the lack of the quaternary center at C11 should facilitate bridging ring construction. Installation of the C5a quaternary stereocenter and its adjacent C6 methyl-bearing center were expected to arise from the [2,3]-Wittig, anionic oxy-Cope cascade shown in detail in Scheme 1b. Ultimately, this process serves to replace what we anticipated would be an easily accessed ether linkage with the more concerning vicinal quaternary/tertiary stereogenic array. Key to the stereochemical outcome of the cascade was the anticipation that the same steric encumbrance that forces the wickerol bridging ring into the boat conformation (the C14 axial methyl group) should drive the reactive conformations of each step of the sequence; for example, the anionic oxy-Cope rearrangement of the *E*-configured allylic alkoxide should proceed via a boat topology. To test this general plan, at our peril, we initially targeted the simplified tetracyclic model system **13** (Scheme 1b inset).

### Toward a Model Tetracyclic Core

**Scheme 2:**
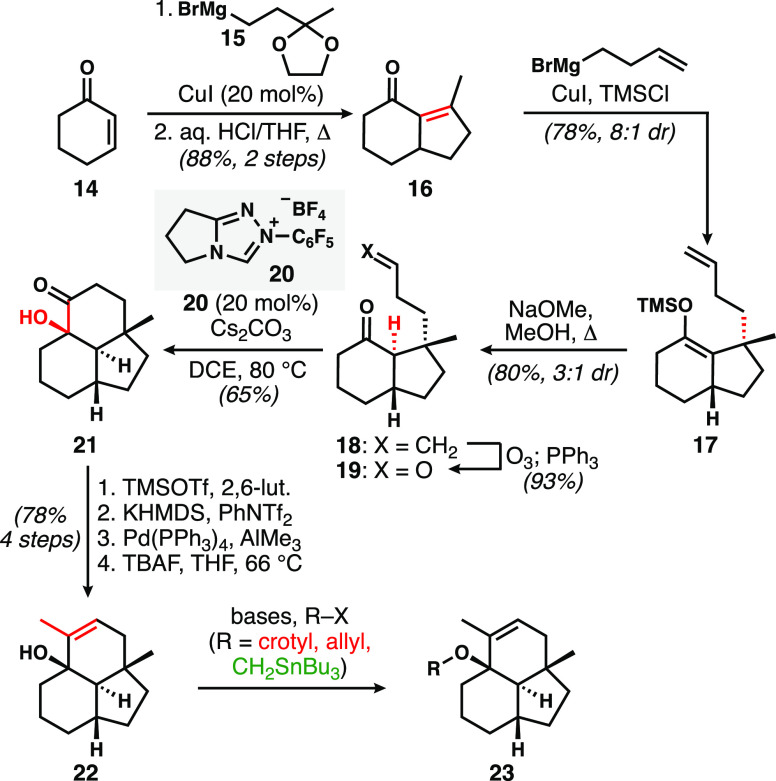
Synthesis of Tertiary Allylic Alcohol 22 from Cyclohexenone

The synthesis of the fused tricyclic model system, as a prelude to consideration of the bridging ring, is described in Scheme 2. Copper-catalyzed conjugate addition of the Grignard reagent **15** derived from methyl vinyl ketone to cyclohexenone, followed by intramolecular aldol condensation under acidic conditions, afforded hydrindenone **16** in high yield.^9^ A second conjugate addition uneventfully introduced the butenyl group with concurrent enoxysilane formation and with good stereochemical control to yield **17**. Enoxysilane methanolysis under conditions that establish the thermodynamic ratio of hydrindanones favored the desired trans ring junction of **18** (3:1 diastereomeric ratio (dr)).^10^ The undesired cis isomer could be resubjected to basic equilibration conditions to aid with material throughput. Ozonolysis of **18** set up for an intramolecular benzoin reaction of **19** employing Rovis’s precatalyst **20**,^11^ which proceeded with excellent diastereoselectivity. Ketol **21** was elaborated to trisubstituted alkene **22***via* a high-yielding sequence of alcohol silylation, alkenyl triflate formation, palladium-catalyzed methylation, and silyl ether cleavage. The resulting tertiary allylic alcohol required only appendage of an *E* crotyl group before the desired pericyclic cascade could be evaluated.

**Figure 2:**
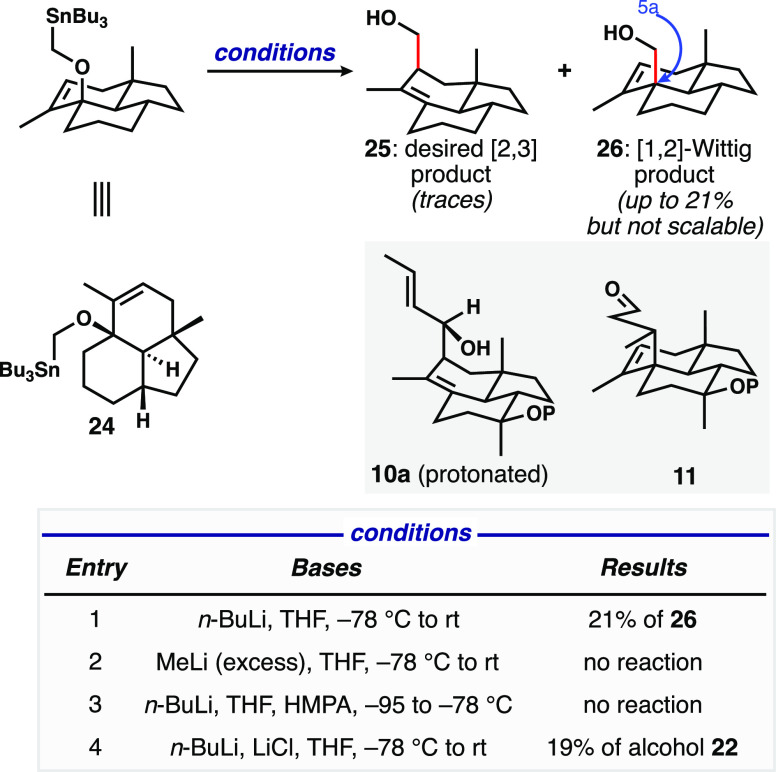
Attempts at the Still variant of the [2,3]-Wittig rearrangement using stannylmethyl ether **24**.

The etherification of the tertiary carbinol proved much more difficult than anticipated. We were unable to install the desired crotyl group using a range of conditions. Bases (NaH, KH, and *n*-BuLi), solvents (tetrahydrofuran (THF), dimethylformamide (DMF)), additives (tetrabutylammonium iodide (TBAI), KI, 18-crown-6), and temperatures (room temperature (rt) to 120 °C) were all varied, typically resulting in recovered starting material; under more extreme conditions, apparent elimination products were observed. Heating the alcohol in neat crotyl bromide with strong amine bases such as 1,8-diazabicyclo[5.4.0]undec-7-ene (DBU) or 1,1,3,3-tetramethylguanidine (TMG) was equally unsuccessful. Given these results, a variety of electrophiles were screened (see the Supporting Information). To our surprise, iodomethyltri-*n*-butylstannane^12^ was the sole electrophile that successfully alkylated **22**. With the stannyl ether derivative **24** in hand, we could interrogate the [2,3]-Wittig rearrangement^13^*via* application of the Still variant^14^ of this sigmatropic rearrangement. We found that the reaction did not proceed at the typically applied cryogenic temperatures, whereas warming of the reaction mixture promoted both the competing [1,2]-rearrangement to form **26**(13) or α-elimination to reform alcohol **22** (Figure 2). We propose that our inability to effect this reaction came down to the inflexibility of the 6-6-5 trans fused ring system to adopt the required five-membered transition structure, and we were never able to isolate [2,3]-rearrangement product **25**, which we intended to elaborate to the model system equivalent of **10a** (the anionic oxy-Cope precursor). Interestingly, in the context of the Still variant that only transfers one carbon atom, the [1,2]-rearranged product **26** is potentially more useful than the [2,3]-product because the C5a quaternary center is installed, and chain extension to deliver a simplified version of Prins/carbonyl ene substrate related to **11** is conceivable. In small-scale reactions, up to 21% of this product was obtained; unfortunately, this sequence of alkylation and [1,2]-rearrangement did not prove at all scalable.

**Scheme 3:**
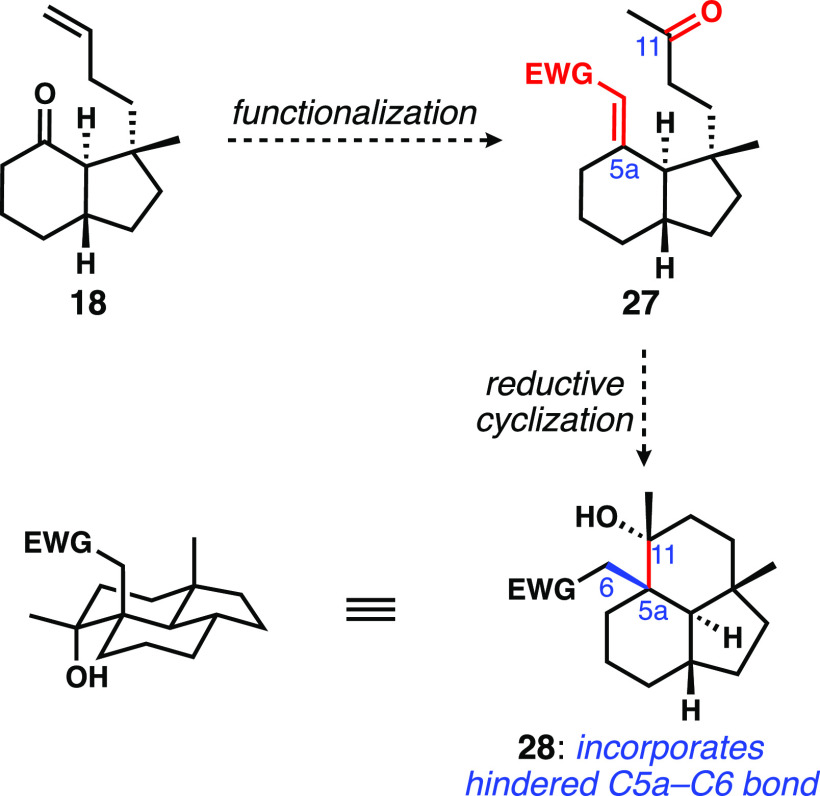
Revised Plan to Target the Tricyclic Ring System with the C5a–C6 Bond in Place

Cognizant that the entire point of the sigmatropic cascade was to replace a hindered C5a–O bond with the C5a–C6 bond and the associated quaternary center, we considered an earlier introduction of that C5a–C6 bond, prior to establishment of the fused tricyclic ring system. A revised plan was hatched to alkenylate the C5a ketone and replace the benzoin reaction with a reductive cyclization to forge the C5a–C11 bond, as shown in Scheme 3.

**Scheme 4:**
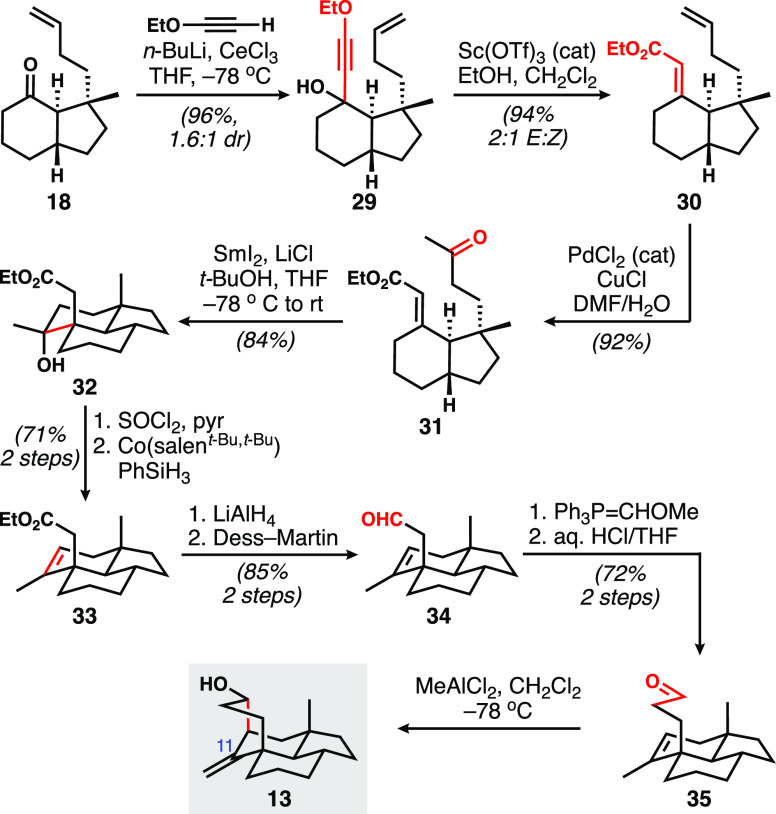
Ketone Alkenylation and Ketyl Radical Anion Cyclization Setup for Installation of the Bridging Ring via Prins Chemistry

To this end, **18** was subjected to a variety of classical alkenylation conditions, mainly focused on Horner–Wadsworth–Emmons and Peterson processes. However, the only outcome from these reactions was epimerization of the trans ring fusion, which indicates the lability of the ring junction. Thus, alternatives that employed more nucleophilic and less basic nucleophiles were sought. In the end, a two-stage procedure was adopted, wherein the alkynylcerium derivative of ethoxyacetylene^15^ was added to form **29**, followed by Meyer–Schuster rearrangement to furnish the desired conjugated enoate ester **30**, in a modification of work by Dudley and co-workers (Scheme 4).^16^ Wacker oxidation delivered **31**, setting up for the key samarium-chloride-induced (SmI_2_/LiCl) ketyl radical anion cyclization,^17^ which proceeded efficiently to afford tricyclic alcohol **32**. Dehydration using thionyl chloride was moderately selective (2.8:1) for the required internal alkene as shown in **33**; the use of Martin’s sulfurane or the Burgess reagent each led to near exclusive formation of the exocyclic isomer. In any case, the crude mixture was directly isomerized using the method of Shenvi^18^ to give predominantly (>10:1) the endocyclic isomer. Reduction of the ester to the aldehyde (**34**) set up for a one-carbon homologation via Wittig chemistry, ultimately giving cyclization precursor **35**. Fortunately, little experimentation was required to find conditions to effect a Lewis-acid-catalyzed cyclization establishing the bridging ring; a mixture of inseparable polycyclic alcohols was formed under the action of MeAlCl_2_, and by our estimation, this mixture contained tetracyclic model target **13**. The isolation of apparent hydride-trapped tetracyclic products when EtAlCl_2_ was used strongly suggested that the cyclization proceeded via cationic (Prins) reactivity, rather than a pericyclic (carbonyl ene) process.^19^ Thus, the general plan toward the tetracyclic architecture of the wickerols was validated. Unfortunately, it would take approximately five years of effort to adapt this general approach to a successful total synthesis of a natural product target.

### Total Synthesis of Wickerols A and B

The key differences between the model target and the natural product wickerol B are (1) the tertiary carbinol at C3, (2) the methyl group borne by C6, and (3) the geminal methyl groups at C11 (eq 1). The last of these we assumed could be introduced from an exocyclic alkene related to **13**; indeed, the Gui synthesis demonstrated exactly this type of reactivity by well-precedented cyclopropanation and cyclopropane hydrogenolysis.^8^ Given the difficulties revealed by Liu and Trauner in the diastereoselective late-stage introduction of the axial methyl group on C3 by 1,2-methylation of a ketone,^7^ we chose to install this stereocenter early in the synthesis. We opted to include the tertiary carbinol in the starting cyclohexenone, which we assumed would have a relatively minor effect on the chemistry we had developed. Finally, it appeared that there were multiple opportunities to bring in the methyl group at C6 either by enolate chemistry or conjugate addition processes. As it would turn out, these assumptions amounted to gross over simplifications.
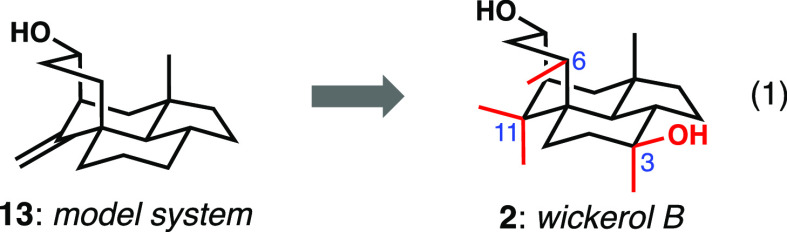
1

**Scheme 5:**
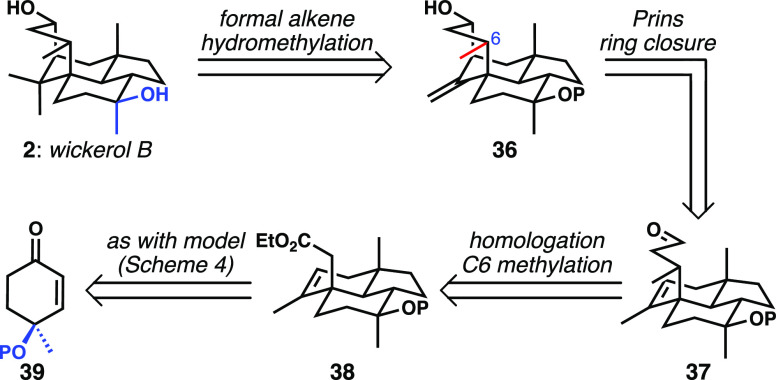
Revised Retrosynthetic Plan toward Wickerol B Showing Differences from Model System Synthesis

Our plan, shown in Scheme 5, crystallized around the use of cyclohexenone **39**, whose chiral center would ideally be used in stereochemical relay to all other centers in the target. Other than this change, the conversion of **38** to **37**, with the introduction of the methyl group to C6, corresponded to the most “uncharted territory” in the synthesis design informed by our successful model study.

**Scheme 6:**
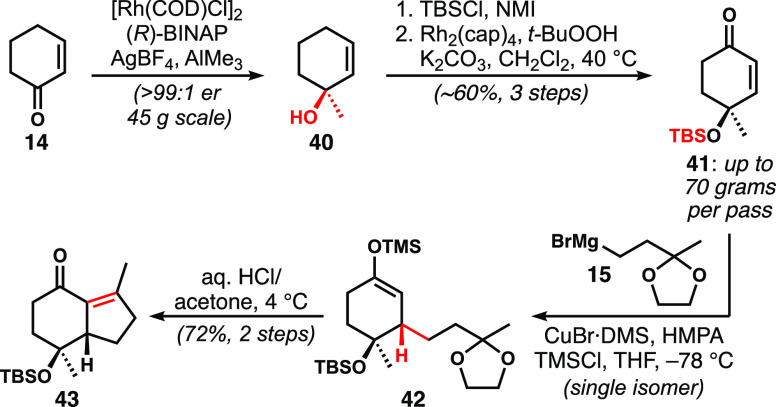
Enantioselective Synthesis of Hydrindenone **43**

Enantioselective methylation of cyclohexenone according to the procedure of von Zezschwitz and co-workers employed Rh(I)/*R*-BINAP catalysis for the addition of trimethylaluminum (Scheme 6).^20^ This procedure, reported on a 1 mmol scale, proved readily scalable to ≥0.5 mol scale reactions with >99:1 enantiomer ratio (er), and the crude product was silylated without purification. Most typical conditions for silylation were ineffective owing to the poor reactivity of the hindered alcohol and its propensity to eliminate. However, the reaction in neat *N*-methylimidazole (NMI) with TBSCl gave **41** with good efficiency.^21^ Allylic oxidation as reported by the Doyle group, using Rh_2_(cap)_4_ with *tert*-butyl hydroperoxide (*t*-BuOOH) as a terminal oxidant, was remarkably effective.^22^ Other conditions employing palladium- or copper-based catalyst systems^23,24^ gave incomplete conversion. Conjugate addition of **15** provided **42** with excellent diastereoselectivity, adding trans to the silyloxy group, as supported by nuclear Overhauser effect (nOe) experiments.^25^ The inclusion of both hexamethylphosphoramide (HMPA) and trimethylsilyl chloride (TMSCl) was required for reactivity at lower temperatures, which was needed to ensure high stereochemical control. Acid-mediated acetal cleavage and intramolecular aldol condensation furnished hydrindenone **43**. This robust sequence routinely procured 10-g batches of this key intermediate.

**Figure 3:**
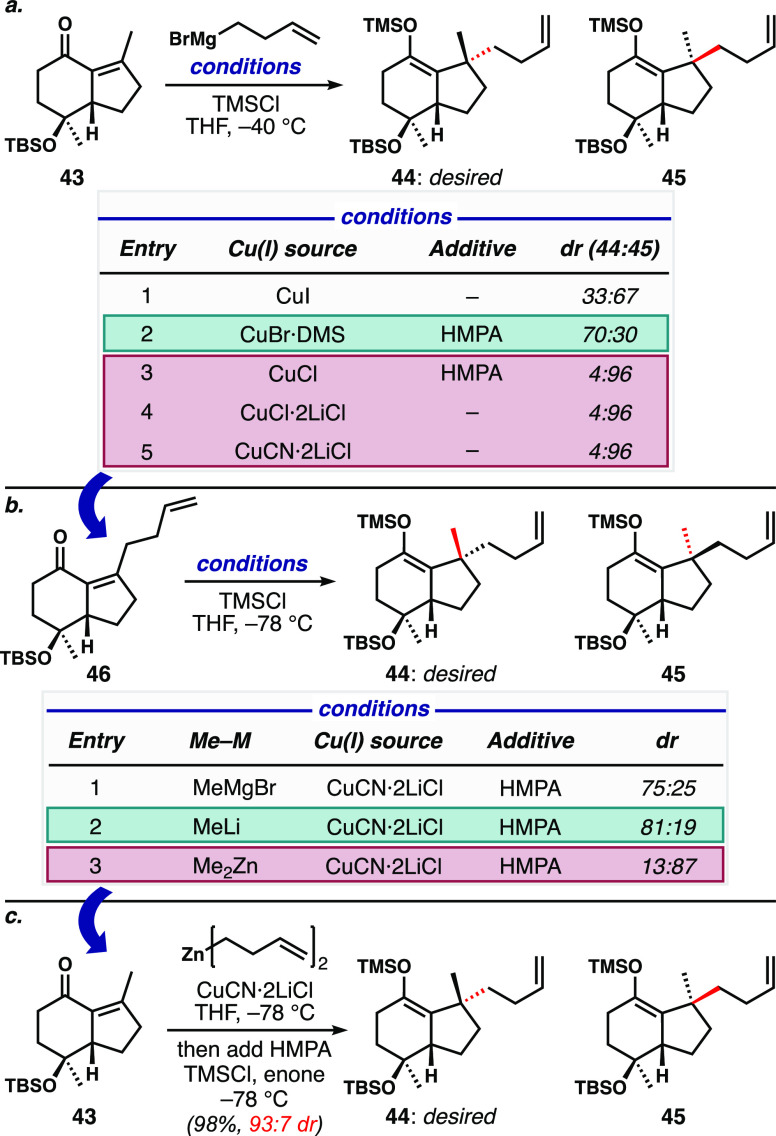
Chronological discovery of a highly diastereoselective conjugate butenylation. (a) Selected results from an extensive screen of conditions for conjugate butenylation of hydrindenone **43**. (b) Conjugate methylation of butenyl-substituted hydrindenone **46** could be controlled to favor either diastereomer. (c) Diorganozinc addition to **43** was efficient and diastereoselective.

Diastereoselective conjugate addition of the butenyl group to **43** (Figure 3a) was the first major roadblock in translating the model system chemistry. Initial experiments using the same conditions from the model system (CuI, TMSCl, giving 8:1 dr) showed modest selectivity for the undesired diastereomer **45** (entry 1). An extensive screen of different copper(I) sources and additives was thus initiated.^25^ Eventually, we found that the combination of the CuBr·DMS complex with HMPA biased the outcome slightly in favor of the desired isomer (entry 2), but further improvement was not achieved at that point. Remarkably, we identified several sets of conditions (entries 3–5) that strongly favored the undesired outcome. The mechanistic basis for these results is not well understood, but it is clear that structural changes in the reactive cuprate cluster led to remarkable shifts in the observed diastereoselectivity.

For reasons dealing with the difficulty in separation of isomers at this and at following stages, we absolutely needed a highly selective formation of **44**. We therefore took these lessons learned and inverted the order of operations for the introduction of the two exocyclic substituents (methyl and butenyl) on the cyclopentane ring (Figure 3b). In other words, we looked to install the methyl group during a conjugate addition to the butenyl-substituted hydrindenone, using conditions similar to those in table entry 5 of Figure 3a. To this end, we synthesized hydrindenone **46** along similar lines to sequence shown in Scheme 6; this chemistry translated reasonably well but was slightly less efficient in the cyclopentene ring annulation sequence. With **46** in hand, and using methylmagnesium bromide as the nucleophile (Figure 3b, entry 1), the diastereoselectivity of the conjugate addition was lower than anticipated (3:1 dr). Interestingly, we found that changing the organometallic nucleophile to methyllithium improved the outcome (entry 2), and the next logical step was the evaluation of other methyl organometallics. Here, the use of dimethylzinc reversed the outcome, significantly favoring the undesired stereoisomer **45** (entry 3). With these results in hand, we surmised that returning to conjugate butenylation of **43** using dialkylzinc—which we had not tried previously—might result in a highly stereocontrolled reaction. Gratifyingly, we found that transmetallation of 3-butenylmagnesium bromide to its organozinc derivative with ZnCl_2_, followed by conjugate addition under the conditions shown in Figure 3b, generated a 93:7 ratio favoring the required stereoisomer **44** (Figure 3c), in almost quantitative yield. This procedure proved robust on a >10 g scale. At this stage, we do not have a good explanation for the complete switch in the stereochemical outcome on going from Grignard/organolithium species to diorganozinc nucleophiles, although the difference in Lewis acidities of the main group metals and the differences in organometallic clusters that result in each case are likely to be key players.

**Scheme 7:**
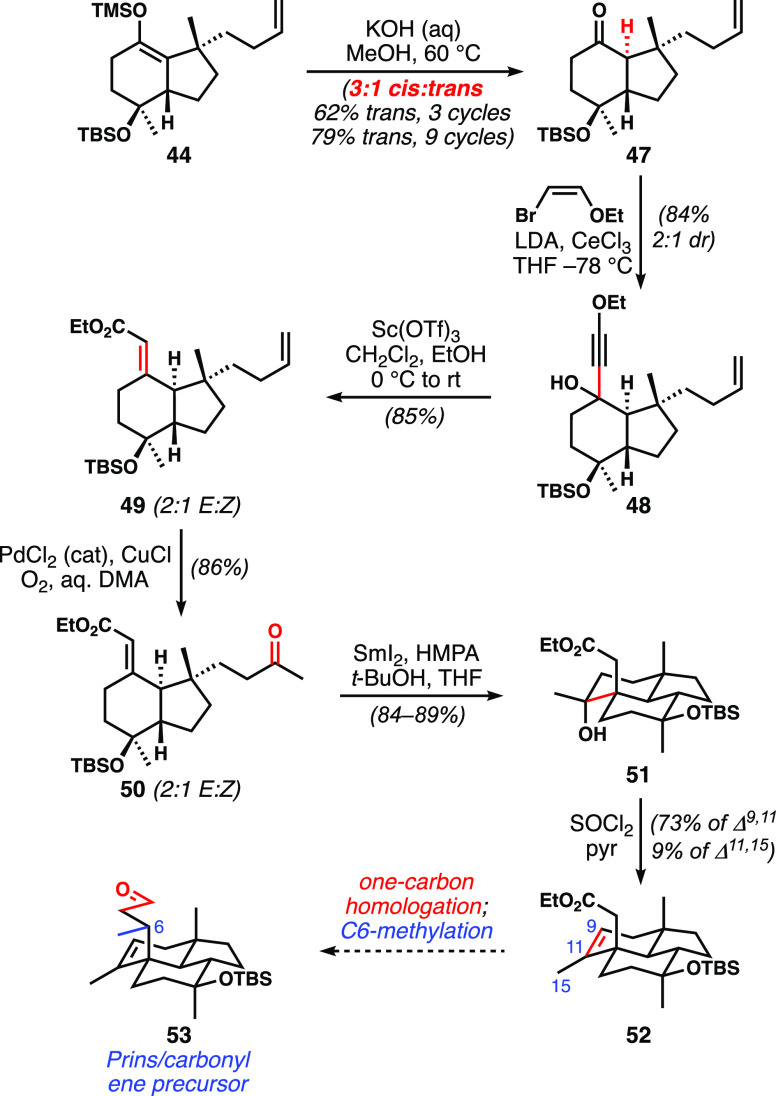
Synthesis of Tricyclic Ester **52** via Reductive Cyclization of Keto-acrylate **50**

Basic hydrolysis of the enoxysilane of **44** yielded *trans-*hydrindanone **47** and its undesired cis diastereomer in a 1:3 ratio (Scheme 7). Mercifully, the diastereomers could be readily separated by column chromatography, and the undesired diastereomer could be equilibrated to the same thermodynamic ratio. Three recycles of material afforded over 60% of the desired stereoisomer; on a 15 g scale, it was even practical to engage in nine separation/equilibration cycles with the desired trans diastereomer obtained in nearly 80% yield. Attention then turned to preparing the substrate for reductive cyclization, and the conditions transferred well with only slight modifications. Ketone **47** was subjected to addition by the organocerium derived from ethoxyacetylene. (*Z*)*-*2-Ethoxyvinylbromide served as a convenient precursor from which to generate lithiated ethoxyacetylene in situ compared to synthesizing ethoxyacetylene discretely.^26^ Meyer–Schuster rearrangement of the carbinol **48** catalyzed by Sc(OTf)_3_^16a^ completed the two-step homologation sequence, giving **49** in excellent yield as a ∼2:1 ratio of alkene geometrical isomers. Wacker oxidation set up for the key reductive cyclization of **50**. Unfortunately, significant optimization was needed to arrive at conditions that were as effective as the SmCl_2_ conditions were for the model system (see below); in this case, SmI_2_ with excess HMPA and careful control of cryogenic temperature delivered tricyclic alcohol **51** with high efficiency. Dehydration was reasonably selective for the endocyclic alkene. At this stage, only one-carbon homologation and C6-methylation were required to get to the key Prins precursor.

**Figure 4:**
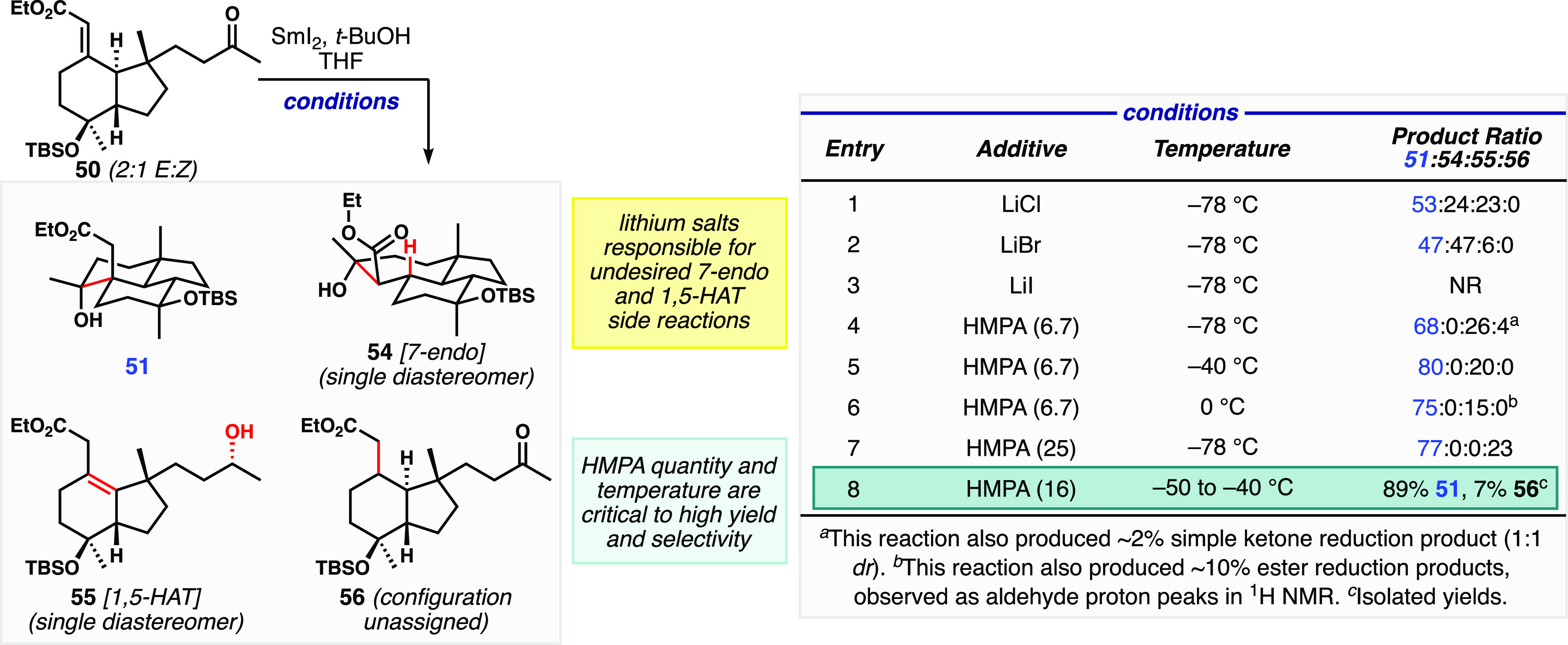
Summary of optimization results for the reductive cyclization of keto-enoate **50**.

A digression on the ketyl radical anion cyclization optimization is warranted. Under the SmCl_2_ conditions (Figure 4, table entry 1) from the model study, we observed two significant side products in addition to the formation of **51**. Cycloheptane **54** arose from competing 7-*endo* cyclization. Deconjugated enoate **55** was also generated, as a single stereoisomer at the secondary carbinol; taken together, the two structural changes in **55** suggest a 1,5 H-atom transfer (HAT) from the γ-position of the enoate to the ketyl radical anion, which might be expected to result in a stereocontrolled net reduction of the ketone. We also found that the *E* isomer of **50** was more reactive under these conditions, with incomplete reactions leading to isolation of starting material that was enriched in *Z* isomer. Undesired 7-*endo* product **54** was inseparable from desired product **51**, so conditions were needed to minimize its formation. We therefore explored the effect of additives on the reaction profile to increase our mechanistic understanding and to develop conditions that minimized impurity formation. Lithium salt additives are known to modulate the reactivity of SmI_2_ by halide metathesis, with redox potential increasing as halide size decreases.^17^ Increasing the redox potential (relative to SmCl_2_ with added LiBr, entry 2) led to increased 7-*endo* cyclization but decreased reduction product that appears to proceed via 1,5 HAT. Unexpectedly, the addition of lithium iodide shut down all reactivity (entry 3).

The addition of HMPA to SmI_2_ solutions is known to make complexes that dramatically impact reduction outcomes in complex settings.^27^ We examined the effects of several different quantities of HMPA, and in all cases, the problem of the formation of cycloheptane **54** was resolved. At lower quantities of HMPA (expected to make [Sm(HMPA)_4_(THF)_2_]^2+^, entries 4–6),^27^ we observed significant amounts of reduction product **55**, along with traces of simple ketone reduction product (not shown), which proved difficult to separate from the desired product. Increasing the temperature increased the amount of the desired product relative to **55**; however, at 0 °C, we observed ∼10% of other reduction products (tentatively aldehyde variants of **51** and **55**). We then increased the equivalents of HMPA relative to SmI_2_^27^ to generate [Sm(HMPA)_6_]^2+^ and found that HAT-based reduction products were not formed (entry 7). However, we identified the formation of 1,4 reduction product **56** with the pendent ketone intact. At −78 °C, significant amounts of this new reduction impurity were observed, while raising the temperature to between −50 and −40 °C and somewhat decreasing the amount of HMPA, we observed suppression of **56** formation. This procedure was scalable and the small amount of **56** was readily separated; these conditions (entry 8) that permitted isolation upward of 85% of the desired tricyclic product **51** were deemed optimized. A full mechanistic discussion can be found in the Supporting Information.

Dehydration of the tertiary alcohol of **51** proceeded smoothly with thionyl chloride and pyridine (Scheme 7). Compared with our results on the model system, we found that cooling the reaction to −40 °C further pushed the regioselectivity in our favor (7:1 *endo*:*exo*, compared to 2.8:1). As with the model system, the undesired alkene isomer could be reequilibrated under HAT conditions.^18^

**Scheme 8:**
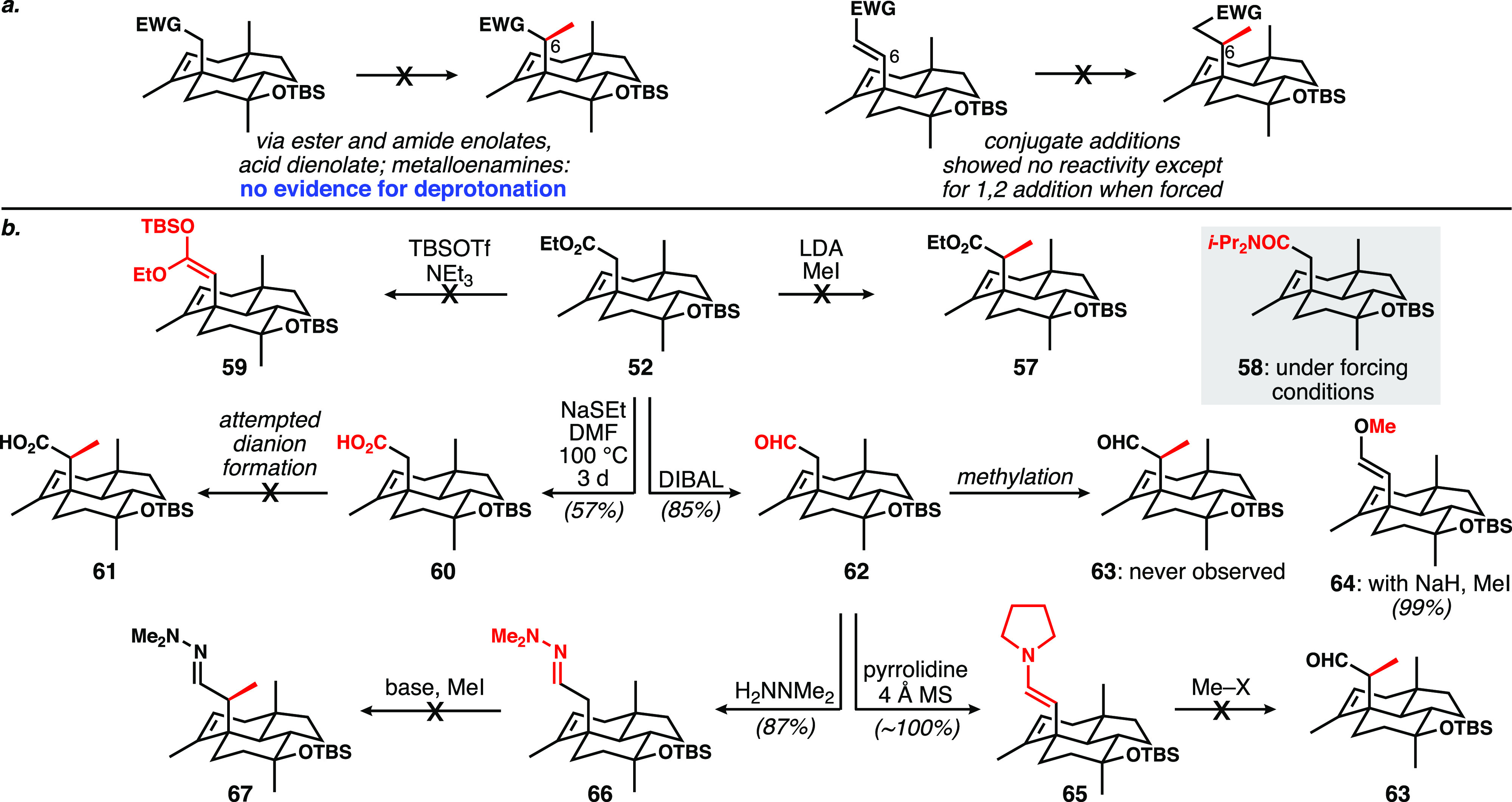
(a) General Strategies for C6-Methylation; (b) Summary of Failed Attempts at Electrophilic Methylation at C6

In a further differentiation from the model system, attention turned to the installation of the C6 methyl substituent, initially via methylation of a C6 nucleophile (enolate or equivalent) and later by conjugate methylation of a homologated system (Scheme 8). Treatment of ester **52** with lithium diisopropyl amide (LDA) and MeI led to clean recovery of starting material; no **57** was ever observed. In an attempt to probe the feasibility of deprotonation, treatment with excess LDA followed by the addition of D_2_O provided only products of amidation (**58**). Multiple conditions for soft enolization to form **59** also failed. Carboxylic acid **60** was made with the intent of generating a highly nucleophilic dianion for methylation, but this reactivity was never realized. Reduction of the ester in **52** produced aldehyde **62** cleanly. Various attempts to *C*-methylate its enolate to form **63** did not work; however, the combined use of sodium hydride and methyl iodide at room temperature led to O-methylation (**64**) as the sole outcome. Enamine **65** could be cleanly generated, but methylation could not be effected; similarly, the metalloenamine derived from hydrazone **66** was not reactive. Clearly, this compilation of results is a testament to the high steric encumbrance around the neopentylic α-carbon in these systems. While homologation of aldehyde **62** to conjugate acceptors (α,β-unsaturated ester, nitrile, and aldehyde) was feasible, all attempts at conjugate methylation were fruitless.

**Scheme 9:**
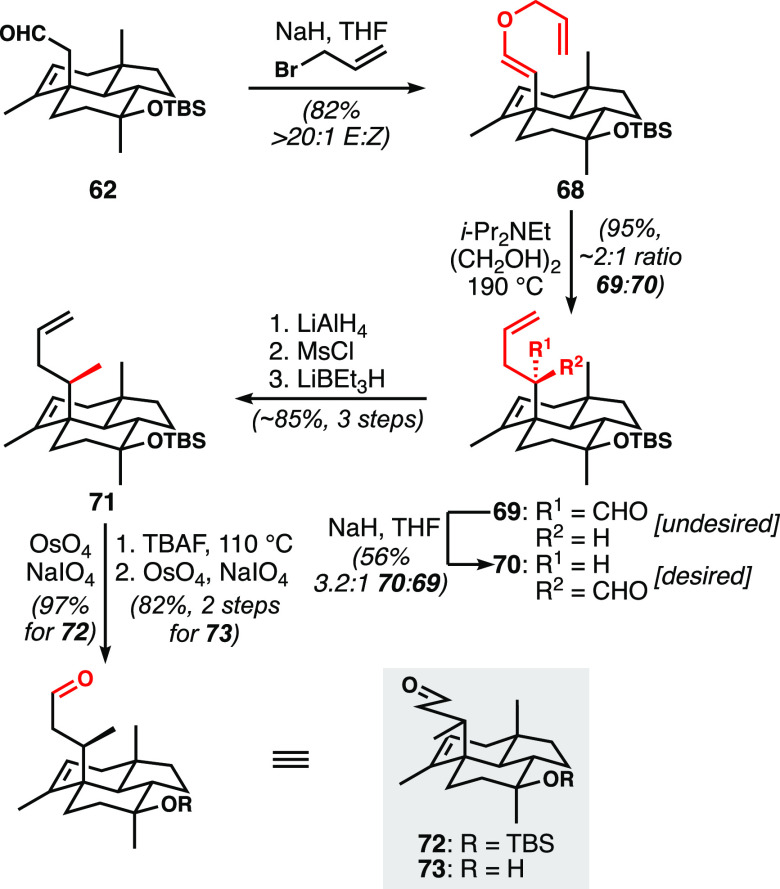
Access to Potential Prins Cyclization Substrates **72** and **73** Enabled by a Claisen Rearrangement to Address the C6 Methyl-Bearing Stereogenic Center

The feasibility of O-alkylation proved to be the one bright spot of the studies in Scheme 8b. We sought to leverage this reactivity in a Claisen-rearrangement-based approach to formally methylate C6 (Scheme 9). We thus targeted alkenyl allyl ether **68**. An important point involving the execution of the Claisen-based strategy emerged: the *Z*-configured alkenyl ether was unreactive in the sigmatropic rearrangement, presumably due to steric interactions between the allyl chain and the tricyclic ring system that preclude adoption of the requisite six-membered transition structure. We found that the diastereoselectivity of the O-allylation exhibited a strong solvent dependence. When DMF was employed, *E*/*Z* ratios in the range of 2.5:1–3:1 were obtained, with slight improvement at reduced temperatures. However, when THF was employed as the solvent, the *E*/*Z* ratio improved to >20:1. This striking solvent effect enabled us to selectively prepare sufficient quantities of *E*-alkenyl ether **68**. However, and despite extensive screening of reaction times and temperatures, variable and generally poor diastereoselectivity was observed in the sigmatropic rearrangement. With optimized conditions, we observed diastereomeric ratios slightly favoring undesired diastereomer **70**. Attempts to perform the Claisen rearrangement in the presence of π-acid catalysts such as PdCl_2_^28^ led to reversion to aldehyde **62**. Diisobutylaluminum hydride and triisobutylaluminum are known to promote Claisen rearrangements with subsequent hydride transfer to the incipient aldehyde in situ, resulting in the formation of the corresponding primary alcohol;^29^ full conversion was observed with these reagents, but with a ratio of 1:3 favoring the undesired diastereomer, (and with expected reduction to the alcohol).

We confirmed the structure of the desired diastereomer of the Claisen rearrangement product (**70**) via X-ray crystallography,^25^ and found that we could reliably equilibrate the undesired isomer to a ∼3:1 ratio favoring the desired isomer. We turned our attention to reduction of the aldehyde group to the corresponding methyl group. Initial efforts surveying Wolff–Kishner, Caglioti,^30^ Yamamura’s modified Clemmensen,^31^ and B(C_6_F_5_)_3_-catalyzed silane-mediated reduction^32^ were not successful. However, we found that a three-step process involving reduction to the alcohol, mesylation, and further reduction with excess Super-Hydride at room temperature allowed clean conversion to **71** in good yield. Selective oxidative cleavage of the monosubstituted alkene proceeded in moderate yield under modified Johnson–Lemieux conditions affording the corresponding aldehyde **72**.^33^ As it would happen, our initial studies of the final ring closure using this aldehyde (see below) led us to evaluate the desilylated compound **73**. It was prepared by desilylation—requiring dissolution of **71** in 1 M tetra-*n*-butylammonium fluoride (TBAF)/THF and heating to 110 °C in a sealed microwave tube—followed by the same modified Johnson–Lemieux oxidation, which proved to be a more efficient sequence. While several steps were ultimately needed to introduce the C6-methyl group, the strategic use of the Claisen rearrangement simultaneously resolved the need for chain homologation, rendering the net process relatively efficient.

**Figure 5:**
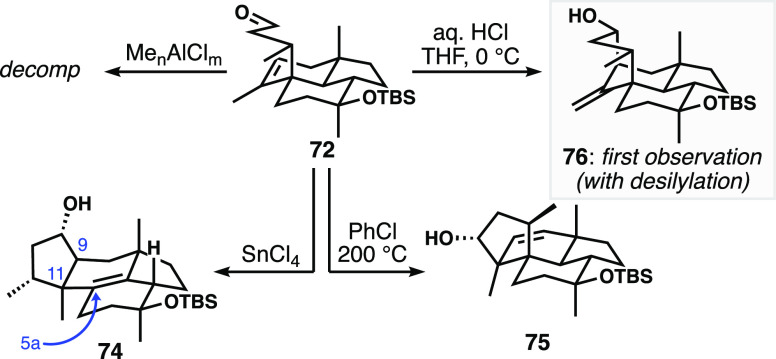
Attempted Prins or carbonyl ene reactivity to forge the fourth ring of the wickerols.

With alkene/aldehyde **72** in hand, we began explorations into the closure of the final ring of wickerol B (Figure 5). We found that application of the conditions that were effective with the model system (MeAlCl_2_) led to decomposition, as did a range of other aluminum-based Lewis acids (Me_2_AlCl and Me_3_Al_2_Cl_3_). When SnCl_4_ was used, we observed fused cyclopentane **74**, which presumably arises from the desired π-cyclization, but with a subsequent strain-relieving alkyl shift from C5a to C11, followed by proton elimination. This was the first hint that the strained ring system might induce undesired reactivity. Other Lewis acids such as Sc(OTf)_3_, BF_3_·OEt_2_, and TMSOTf were unproductive. Interestingly, we found that heating **72** led to a carbonyl ene reaction exclusively engaging the allylic methylene hydrogen atom rather than one from the methyl group, thus producing **75**. Finally, when HCl was employed, traces of the desired product **76** were observed alongside significant decomposition of the substrate, presumably in part through acid-mediated desilylation.

**Figure 6:**
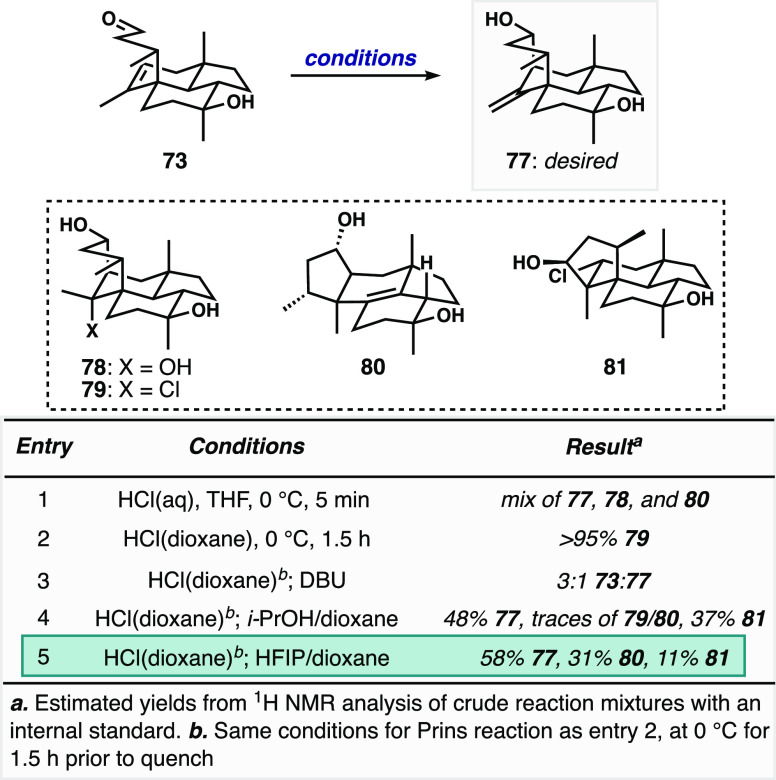
Selected experiments designed to optimize the Prins cyclization of **73**.

To mitigate substrate decomposition related to partial silyl ether cleavage, we evaluated the Prins cyclization reactivity of desilylated aldehyde **73**, focusing on Brønsted–Lowry acids owing to the promising reactivity observed with **72**. When **73** was treated with aqueous HCl, the desired Prins product **77** was obtained as a minor product alongside hydration product **78** and carbocation rearrangement products **80** and **81**, which were major components (Figure 6). We hypothesize that these products arise via divergent strain-relieving Wagner–Meerwein rearrangements with subsequent elimination or chloride trapping, respectively. The facility with which these rearrangement products are formed again speaks to the highly strained nature of the wickerol carbocyclic skeleton and a strong driving force for its relief by removal of the bridging ring. A variety of Brønsted–Lowry acidic, nonaqueous conditions were screened for the Prins cyclization (not shown^25^); milder conditions led predominantly to the formation of Wagner–Meerwein rearrangement product **80**, and harsher conditions caused complete decomposition of the substrate, likely via ionization of the tertiary alcohol. We then turned our attention to optimizing reaction conditions using HCl. In an effort to reduce the generation of hydration product **78**, we employed anhydrous HCl. To our surprise, when HCl in dioxane was used and the reaction mixture was concentrated in vacuo rather than subjected to standard basic workup (entry 2), we observed efficient formation of chlorinative Prins product **79** (“chloro-norwickerol B”). Encouraged by these results, we sought to perform a controlled dehydrohalogenation of **79** to afford **77**, in the face of potentially facile reversion to starting material via Grob fragmentation, or formation of **80** and **81***via* Wagner–Meerwein rearrangements. When **79** was treated with DBU (entry 3), **73** was observed almost exclusively, reinforcing our hypothesis that regeneration of starting material observed in some of our other experiments proceeds via Grob fragmentation. To that end, we investigated various quenching conditions that might promote ionization of the tertiary alkyl chloride. When crude **79** was subjected to aqueous mild basic or mild acidic conditions (not shown), Grob fragmentation to form **73** was predominantly observed. Based on these results, we sought milder conditions to promote ionization. We hypothesized that a solvent exchange to place **79** in a polar protic environment would facilitate ionization without promoting rapid Grob fragmentation. To our delight, when **79** was treated with a mixture of *i*-PrOH and 1,4-dioxane, **77** was the dominant product. Further optimization led us to conditions employing a mixture of hexafluoroisopropanol (HFIP)^34^ and 1,4-dioxane, which when introduced to crude chloride **79** led to the formation of the desired product as 55–60% of the crude mass balance, with the remainder going to rearrangement products **80** and **81**.

**Scheme 10:**
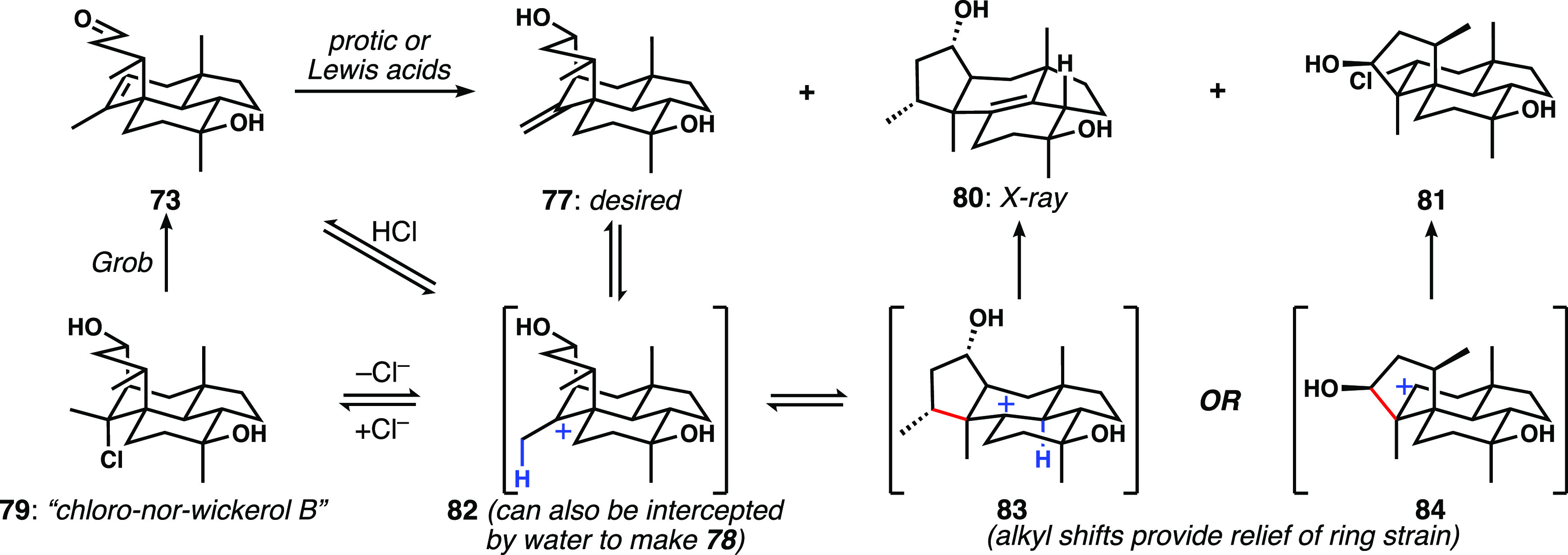
Plausible Mechanistic Details Underpinning the Diversity of Prins Reaction Products

Our current understanding of the landscape of intermediates and products in these Prins equilibria is shown in Scheme 10, which shows clearly why carefully controlled conditions are required to effect dehydrohalogenation of **79**. Avoidance of the Grob fragmentation requires neutral conditions, and ionization of the tertiary chloride sets up for a facile, strain-relieving alkyl shift, leading to **80**/**81** that can in many instances compete with proton loss to afford the desired bridged tetracycle **77**. The identity of **80** was secured by X-ray crystallography, and the proposed structure of **81** was supported by NMR experiments, including ^13^C NMR shift predictions.^25^

**Scheme 11:**
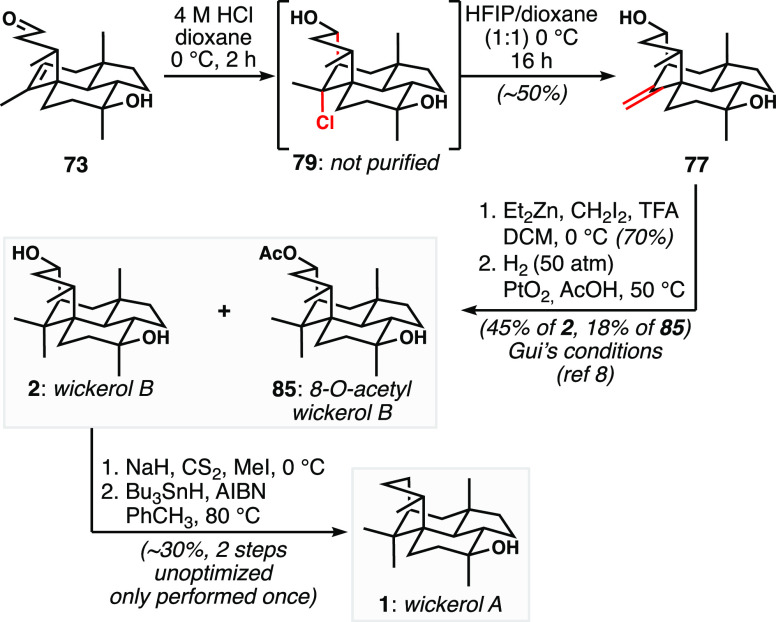
Completion of the Synthesis of Wickerols A and B

With our optimized Prins cyclization conditions, we could reliably obtain **77** in ∼50% isolated yield (Scheme 11), intercepting Gui’s intermediate^8^ and achieving the formal total synthesis of wickerol B. We sought to improve the efficiency of Gui’s endgame by attempting a one-step alkene hydromethylation. Unfortunately, both Baran’s and Frederich’s hydromethylation protocols^35,36^ proved unsuccessful in our system, indicating the strain incorporated into the system by introduction of the geminal dimethyl group. Ultimately, we elected to reproduce Gui’s protocol.^8^ Modified Simmons–Smith cyclopropanation conditions^37^ were employed to afford the spiro-cyclopropane, which was then subjected to hydrogenolysis by the action of Adam’s catalyst, affording wickerol B (**2**). As was the case for Gui and co-workers, the hydrogenolysis reaction afforded varying but significant amounts of 8-*O-*acetyl wickerol B (**85**), a recently reported natural product.^5^ Reductive cleavage of the ester allowed for the isolation of more wickerol B (not shown).

**Figure 7:**
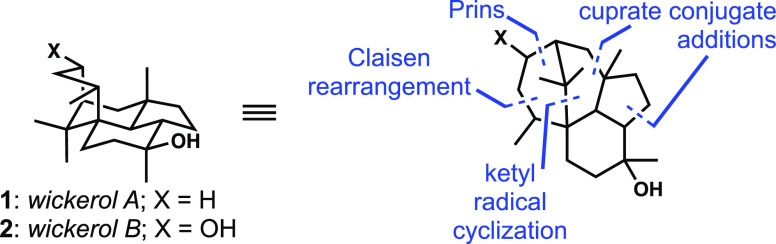
Key alkene-based strategic bond constructions used in our wickerol synthesis.

Finally, we executed the planned deoxygenation of the C8 hydroxyl group of wickerol B that had permitted the installation of the strained bridging ring via Prins cyclization. Application of Barton–McCombie conditions using the intermediate xanthate ester^38^ proceeded uneventfully to afford wickerol A (**1**) in modest yield, without any optimization. Our syntheses of wickerols A and B were thus complete, based on a blueprint that featured many different modes of alkene reactivity to forge strategic bonds in the targets (Figure 7).

## Conclusions

We have achieved a de novo synthesis of the strained tetracyclic diterpenoids wickerols A and B, as well as 8-*O-*acetyl wickerol B. Route scouting was carried out on a simplified model system. The general synthetic plan discovered therein was—with significant optimization—successfully implemented in a synthesis of the natural products themselves.

The synthesis of wickerol B was completed in 23 steps from cyclohexenone, which compares favorably to Trauner and Liu’s work, but is longer than that of Gui and co-workers, who cleverly started from sitolactone, which provides the *trans*-hydrindane and one of the quaternary stereogenic centers of the target. Important lessons that transcend this specific synthesis include the following: (1) intriguing and potentially generalizable changes in diastereoselectivity of cuprate conjugate additions to set quaternary stereogenic centers; (2) application of a cerium acetylide ketone addition/Meyer–Schuster rearrangement sequence to alkenylate a very hindered ketone; (3) that judicious additive choice in reductive samarium(II) cyclizations of polyfunctional keto-enoates results in markedly different impurity profiles based on different operative mechanisms; (4) a Claisen-rearrangement-based sequence to install a methyl-bearing stereogenic center with simultaneous formal homologation of an aldehyde that was recalcitrant to traditional enolate methylation; and (5) a Prins cyclization to install the bridging ring that is both sterically congested and strained. Indeed, the strain built into the tetracyclic core led to fascinating rearrangement chemistry in late-stage intermediates, and required significant experimentation and understanding to control, such that the desired, natural product scaffold was favored.

## Supporting Information Available

Supporting discussion of optimization studies and reactivity; experimental procedures for the synthesis of new compounds; tabulated spectral data supporting the assignment of these compounds; NMR spectra for these compounds; HPLC data for compound **40**; X-ray diffraction information for compounds **21** and **80**; and ^13^C NMR chemical shift calculations for rearrangement compounds (PDF)
