# Supplementary material for: Enantioselective Syntheses of Wickerols A and B
Source: J Am Chem Soc. 2023 Mar 8;145(11):6486–97. doi: 10.1021/jacs.3c00448 (PMC10037325; doi:10.1021/jacs.3c00448)
Supplement: Supplementary file 1 — ja3c00448_si_001.pdf [file ja3c00448_si_001.pdf]

Enantioselective Syntheses of Wickerols A and B

Jonathan Chung, Joseph S. Capani, Jr., Matthias Göhl, Philipp C. Roosen, and Christopher D. Vanderwal

Supporting Information

Table of Contents:

I. Supplementary Results and Discussion

II. Experimental Information

Materials and Methods……**S2**

Method for Predicting ^{13}C Chemical Shifts……**S2**

Experimental Procedures and Characterization Data……**S3**

III. References……**S35**

IV. Experimental Data

SFC Traces……**S36**

X-ray Data……**S37**

Predicted ^{13}C Chemical Shifts and Comparisons of Spectral Data……**S62**

1D and 2D NMR Spectra……**S75**

I. Supplementary Results and Discussion

Conjugate butenylation

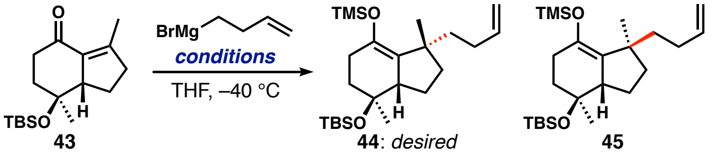

Entry	Cu(I) source	Additive	dr (44:45)
1	CuI	–	33:67
2	CuBr	–	45:55
3	CuCl	–	48:52
4	CuBr·DMS	HMPA	70:30
5	CuCl	DMS	47:53
6	CuCl	HMPA	4:96
7	CuCl·2LiCl	–	4:96
8	CuCN	HMPA	6:94
9	CuCN·2LiCl	HMPA	4:96
10	CuCN·2LiCl	–	4:96

Figure S1. Selected results from an extensive screen of conditions for conjugate butenylation of hydrindenone **43**.

Ketyl radical cyclization

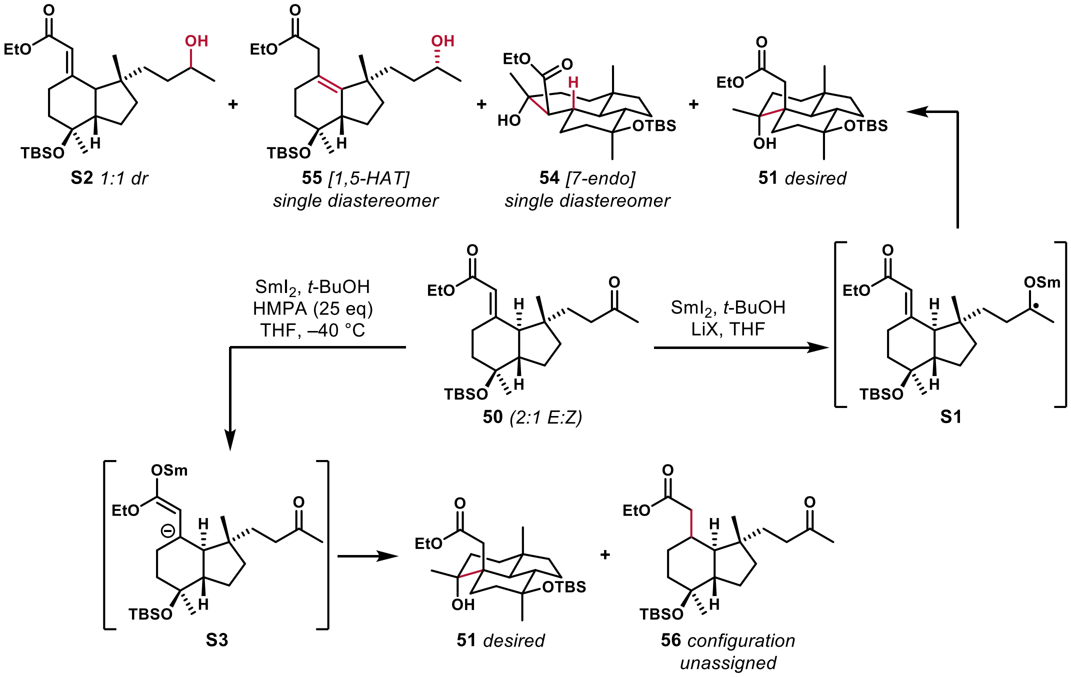

Scheme S1. Plausible divergent mechanistic pathways for the formation of byproducts during ketyl radical cyclization.

We expect that two distinct mechanisms are operative, especially considering the mutually exclusive formation of **55** and **56**. Firstly, **50** can take delivery of an electron from Sm(II) at the ketone, generating ketyl radical anion **S1** that could then undergo intramolecular 6-*exo* Giese addition to give the desired **51**. The observed diastereoselectivity is rationalized by electrostatic repulsion between the ketyl alkoxide and the enoate ester.[1] Compared with the model system, it is conceivable that some steric strain from the

axial methyl group must be overcome, possibly driving ketyl radical anion **S1** down non-productive pathways such as 1,5-HAT to stereoselectively generate **55** or premature reduction to the alcohol **S2**. That hypothesis is also consistent with the observation of 7-*endo* product **54**, which was not observed in the model system.

Our observation that **55** and **56** formation were mutually exclusive suggests that, depending upon the reduction potential of the Sm(II) complex utilized, **50** can accept an electron at the unsaturated ester. Indeed, this anionic reaction pathway for bond formation has been proposed by Proctor and co-workers.[2,3] The presumed dianion **S3** could undergo intramolecular homoaldol addition to generate the desired product **51**, while quenching could lead to generation of **56**. It is clear that modulations in redox potential and additives can have a large impact on the operative reaction pathways.

<u>*Prins cyclization*</u>

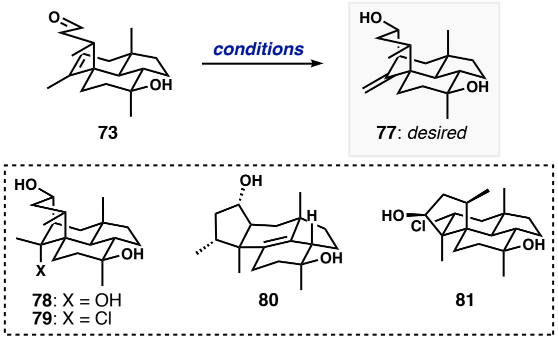

Entry	*Conditions*	*Result*
1	$HCl_{(aq)}$, THF, 0 °C	*29% **77**, 49% **78**, 22% **80***
2	TFA, CH_2Cl_2, rt	*>95% **80***
3	THF, CH_2Cl_2, -40 °C	*>95% **80***
4	THF, CH_2Cl_2, -78 °C	*no reaction*
5	AcOH, CH_2Cl_2, 40 °C	*no reaction*
6	MsOH, CH_2Cl_2, 0 °C	*decomposition*
7	MsOH, THF, 0 °C	*no reaction*
8	MsOH, dioxane, 0 °C	*20% **73**, 35% **77**, 45% **80***
9	HBF_4•OEt_2, CH_2Cl_2, 0 °C	*decomposition*
10	HBF_4•OEt_2, THF, 0 °C	*no reaction*
11	HBF_4•OEt_2, dioxane, 0 °C	*decomposition*
12	HFIP, 50 °C, 24 hr	*56% **73**, 44% **80***
13	Dowex 50WX8-400, CH_2Cl_2, rt	*17% **73**, 9% **77**, 74% **80***
14	Dowex 50WX8-400, THF, rt	*31% **73**, 24% **77**, 45% **80***
15	SiO_2•$NaHSO_4$, CH_2Cl_2, rt	*decomposition*
16	SiO_2, CH_2Cl_2, rt	*no reaction*
17	SiO_2, THF, rt	*no reaction*
18	SiO_2, MeOH, rt	*no reaction*
19	MK-10 clay, CH_2Cl_2, rt	*decomposition*
20	MK-10 clay, THF, 40 °C	*no reaction*
21	TMSCl, MeOH, -40 °C	*decomposition, MeOH incorporation*
22	$HCl_{(dioxane)}$, 0 °C	*>95% **79***
23	$HCl_{(ether)}$, 0 °C	*8% **77**, 22% **80**, 70% **81***
24	$HCl_{(dioxane)}$; DBU	*75% **73**, 25% **79***
25	$HCl_{(dioxane)}$; $NaHCO_{3(aq)}$	*52% **73**, 15% **77**, 24% **78**, 9% **81***
26	$HCl_{(ether)}$; $NaHCO_{3(aq)}$	*42% **73**, 9% **77**, 12% **79**, 12% **80**, 25% **81***
27	$HCl_{(dioxane)}$; $NH_4Cl_{(aq)}$	*65% **73**, 14% **77**, 8% **80**, 13% **81***

28	$HCl_{(dioxane)}$; SiO_2, dioxane, rt	*4% **73**, 43% **77**, 8% **79**, 23% **80**, 19% **81***
29	$HCl_{(dioxane)}$; *i*-PrOH, 0 °C	*21% **77**, 24% **79**, 2% **80**, 53% **81***
30	$HCl_{(dioxane)}$; *i*-PrOH, rt	*2% **73**, 48% **77**, 7% **79**, 7% **80**, 37% **81***
31	$HCl_{(dioxane)}$; HFIP, 0 °C	*decomposition*
32	$HCl_{(dioxane)}$; HFIP/THF, °C	*decomposition*
33	$HCl_{(dioxane)}$; HFIP/CH_2Cl_2, °C	*decomposition*
34	$HCl_{(dioxane)}$; HFIP/Et_2O, °C	*3% **73**, 37% **77**, 44% **80**, 16% **81***
35	$HCl_{(dioxane)}$; HFIP/dioxane, 0 °C	*58% **77**, 31% **80**, 11% **81***

Figure S2. Selected results from an extensive screen of conditions for Prins cyclization of aldehyde **73**.

II. Experimental Information

Materials and Methods

All reactions were carried out in flame-dried glassware under an atmosphere of argon with dry solvents unless otherwise noted. Microwave reactions were performed in a CEM Discover Microwave. Reaction solvents including tetrahydrofuran (THF), diethyl ether (Et_2O), dichloromethane (CH_2Cl_2), toluene, benzene (C_6H_6), acetonitrile (MeCN) and dimethylformamide (DMF) were dried by percolation through a column packed with neutral alumina and a column packed with Q5 reactant (a supported copper catalyst for scavenging oxygen) under a positive pressure of Ar. Diisopropylamine (*i*-Pr_2NH), diisopropylethylamine (*i*-Pr_2NEt), triethylamine (NEt_3), pyridine (py), Trimethylsilyl chloride (TMSCl), 1,3-dimethyltetrahydropyrimidin-2(1H)-one (DMPU), and hexamethylphosphoramide (HMPA) were purified by distillation from CaH_2. *N*-Bromosuccinimide (NBS) was purified by crystallization from water. Methyl vinyl ketone (MVK) was purified by distillation. Thionyl chloride ($SOCl_2$) was purified by distillation. Copper iodide was purified by extracting impurities with THF *via* Soxlet and dried in vacuo at 100 °C. 2,6-lutidine was passed over a plug of basic alumina immediately prior to use. Carbon disulfide was purified by distillation immediately prior to use. Tributyltin hydride (Bu_3SnH) was purified by distillation. All other commercially available solvents and reagents were used as received, unless otherwise indicated.

Solvents for workup and chromatography were: hexanes (Fisher or EMD, ACS Grade), pentane (Fisher, ACS Grade), EtOAc (Fisher, ACS Grade), chloroform ($CHCl_3$, Fisher, ACS Grade), dichloromethane (CH_2Cl_2, Fisher, ACS Grade), and diethyl ether (Et_2O, Fisher, ACS Grade). Analytical thin layer chromatography was performed on 0.25 mm EMD glass-backed TLC plates impregnated with a fluorescent dye and visualized with UV light (254 or 210 nm) and $KMnO_4$ in K_2CO_3/NaOH/H_2O, *p*-anisaldehyde in ethanol/aqueous H_2SO_4/AcOH, or phosphomolybdic acid (PMA) staining solutions. Forced flow (flash) chromatography was performed on EMD Silica 60, mesh 0.04-0.063 silica gel. Yields refer to chromatographically and spectroscopically homogeneous materials, unless otherwise stated.

NMR spectra were recorded on Bruker 500 MHz and Bruker 600 MHz instruments, obtained at 298 K unless otherwise noted and referenced to residual chloroform (7.26 ppm, ^1H) or to $CDCl_3$ (77.16 ppm, ^{13}C). Chemical shifts are reported in ppm with the following abbreviations to explain multiplicities: s = singlet, d = doublet, t = triplet, q = quartet, quin = quintuplet, bs = broad singlet, m = multiplet. All coupling constants are apparent *J* values measured at the indicated field strengths and reported in Hertz (Hz). FT-IR spectra were recorded on a Perkin-Elmer spectrum RX1 spectrometer. High-resolution mass spectra (HRMS) were recorded on a Waters LCT Premier spectrometer using ESI-TOF (electrospray ionization-time of flight) or a Waters GCT Premier Micromass GC-MS (chemical ionization), and data are reported in the form of (m/z). Melting points were measured on a MEL-TEMP II capillary apparatus and stand uncorrected.

Method for Predicting ^{13}C Chemical shifts

All calculations on structures **78**, **79**, **80**, and **81** were performed using Spartan 18 in the gas phase. First, a distribution of conformers was calculated with Molecular Mechanics MMFF. All conformers exceeding 13 kcal/mol were discarded. The remaining conformers were subjected to an equilibrium geometry calculation using HF/3-21G and then assigned an alignment score. Any duplicates (conformers that shared an align score and relative energy) were discarded as well as any conformers exceeding 5 kcal/mol. All remaining conformers were refined using a single point energy calculation with ωB97X-D/6-31G* and all conformers exceeding 3 kcal/mol were discarded. Remaining conformers were subject to final energy refinement with ωB97XD/6-311+G(2df,2p)[6-311G*]

with NMR prediction using ωB97X-D/6-31G*. Each of the conformers were assigned a Boltzmann weight and the Boltzmann averaged ^{13}C shifts are reported.

Experimental Procedures and Characterization Data

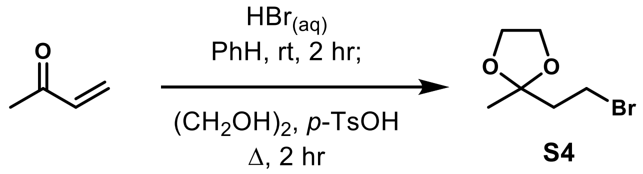

To a 3 L round bottom flask equipped with a stir bar was added benzene (1.20 L), butenone (100 mL, 1.20 mol), and $HBr_{(aq)}$ (48% w/v, 404 mL, 2.40 mol). The reaction mixture was allowed to stir vigorously at room temperature for 2 hours, then transferred to a separatory funnel and washed with saturated $NaHCO_{3(aq)}$ (3 x 750 mL) and brine (1 x 750 mL). The organic extracts were dried over $MgSO_4$ and filtered into a 3 L round bottom flask equipped with stir bar. Neat ethylene glycol (70.5 mL, 1.26 mol) and *p*-TsOH (5.70 g, 30.0 mmol) were added to the reaction vessel, which was then equipped with a Dean–Stark apparatus and heated to reflux *via* oil bath. After stirring at reflux for two hours, the reaction mixture was allowed to cool to room temperature, then quenched with saturated $NaHCO_{3(aq)}$ (750 mL), diluted with pentane (1 L), and partitioned. The organic extracts were washed with water (7 x 600 mL) and brine (2 x 600 mL), dried over $MgSO_4$, filtered, and gently concentrated *in vacuo*. The crude product was purified by distillation under reduced pressure (10 torr, 62 °C) affording **bromoketal S4** (46.8 g, 20%) as a colorless liquid. The characterization data obtained are in good agreement with those previously reported.[4]

bromoketal S4
^{1}H NMR (500 MHz, $CDCl_3$) δ 3.98-3.83 (m, 4H), 3.42-3.31 (m, 2H), 2.29-2.17 (m, 2H), 1.29 (s, 3H) ppm; **^{13}C NMR** (125 MHz, $CDCl_3$) δ 109.1, 64.9, 42.8, 27.0, 24.1 ppm; **HRMS** (CI) calculated for $C_6H_{11}BrO_2$ $[M+H]^+$ 195.0021 found 195.0023.

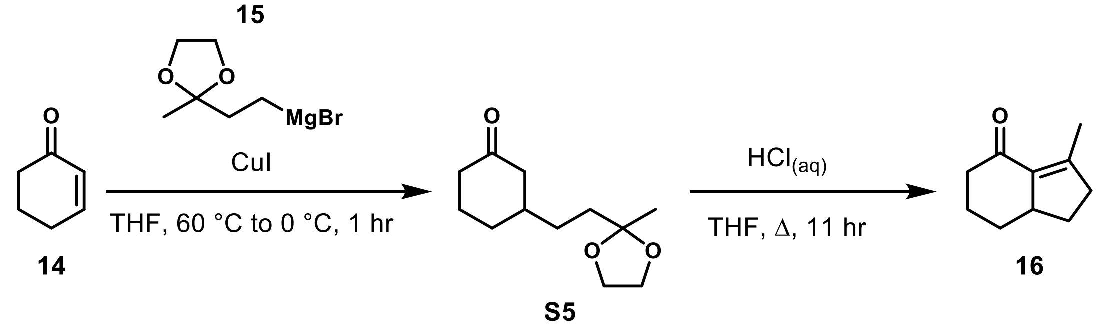

To a 3-necked 500 mL round bottom flask equipped with a stir bar, an addition funnel, and an internal thermometer was added magnesium powder (20-230 mesh, 8.81 g, 362 mmol). The flask was then flame-dried under high vacuum while stirring and allowed to cool to room temperature under an atmosphere of argon. THF (120 mL) and dibromoethane (0.6 mL) were added. The resultant slurry was sonicated for one minute, heated slightly with a heat gun, then stirred vigorously at room temperature until a gray precipitate was observed (ca. 30 min). The reaction vessel was placed in a room temperature water bath. A solution of **bromoketal S4** (24.0 g, 123 mmol) and dibromoethane (1.8 mL) in THF (36 mL) was added dropwise *via* addition funnel at a rate sufficient to maintain an internal temperature of 20 °C. Ice was added to the bath as necessary to maintain the internal temperature at 20 °C throughout the course of the addition. The reaction mixture was then allowed to stir for 30 minutes at an internal temperature of 20 °C. Stirring was halted, and the precipitate was allowed to settle (ca. 15 min). The supernatant containing **Grignard reagent 15** was transferred *via* cannula into a flame dried 2-necked 50 mL round bottom flask equipped with stir bar, rinsing with THF (2 x 5 mL). The solution was then cooled to –30 °C (internal temperature) *via* $MeOH/H_2O/CO_{2(s)}$ bath and freshly purified CuI (229 mg, 1.20 mmol) was added in one portion. The reaction mixture was allowed to stir for 30 minutes at –30 °C (internal temperature). The reaction mixture was then cooled to –60 °C (internal temperature) *via* $Me_2CO/CO_{2(s)}$ bath. A solution of **cyclohexanone 14** (7.02 mL, 72.5 mmol) in THF (24 mL)

was added dropwise *via* addition funnel at a rate sufficient to maintain an internal temperature below –60 °C. A color change from white to yellow to orange was observed over the course of the addition. Upon completion of the addition, the reaction vessel was then allowed to stir while slowly warming to 0 °C over a period of 1 hour. During this period, a color change from orange to black was observed. After 1 hour, the reaction was quenched at 0 °C by the addition of 4:1 $NH_4Cl_{(aq)}$:1 M $NaOH_{(aq)}$ (300 mL) then allowed to warm to room temperature. The mixture was diluted with water until precipitated salts were dissolved, and extracted with Et_2O (480 mL, then 120 mL). The organic extracts were washed with water (2 x 180 mL). The water layers were back extracted with Et_2O (120 mL). The organic extracts were washed with brine, dried over $MgSO_4$, filtered, and concentrated *in vacuo*. Crude **keto dioxolane S5** was carried forward without further purification.

To a 2 L round bottom flask equipped with stir bar containing crude **keto dioxolane S5** was added THF (362 mL) followed by 2 M $HCl_{(aq)}$ (362 mL). The reaction vessel was heated to 75 °C *via* oil bath and allowed to stir at 75 °C for 11 hours. Upon consumption of starting material as indicated by TLC analysis, the reaction vessel was allowed to cool to room temperature. The phases were partitioned, and the aqueous layer was extracted with Et_2O (540 mL) and EtOAc (180 mL). The organic extracts were washed with saturated $NaHCO_{3(aq)}$ (240 mL) and brine (180 mL), dried over $MgSO_4$, filtered, and concentrated *in vacuo*. The crude product was purified by column chromatography (SiO_2, 5% to 10% Et_2O in hexanes, stepped gradient) affording **hydrindenone 16** (9.59 g, 88% overall) as a colorless oil.

hydrindenone 16
^{1}H NMR (500 MHz, $CDCl_3$) δ 2.81–2.70 (m, 1H), 2.53–2.39 (m, 2H), 2.30-2.18 (m, 2H), 2.18-2.10 (m, 1H), 2.10-2.05 (s, 3H), 2.05-2.00 (m, 1H), 2.00-1.93, (m, 1H), 1.80-1.69 (m, 1H), 1.46 (quint, 1H, *J* = 10.6 Hz), 1.22 (qd, 1H, *J* = 20.7, 3.0) ppm; **^{13}C NMR** (125 MHz, $CDCl_3$) δ 200.8, 153.1, 135.9, 47.3, 41.5, 38.7, 32.4, 31.5, 24.4, 16.4 ppm; **IR** (thin film) ν_{max} 2926, 2859, 1677, 1619, 1260 cm^{-1}; **HRMS** (ESI+) calculated for $C_{10}H_{14}O$ [M+H]$^+$ 151.1123 found 151.1117.

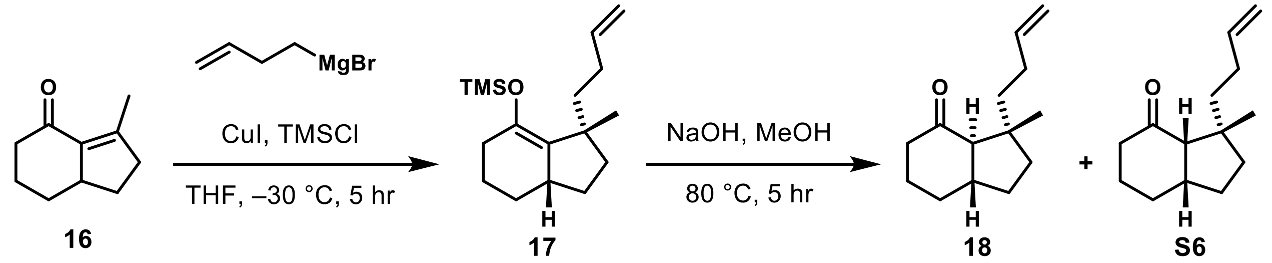

To a 25 mL round bottom flask equipped with a stir bar was added magnesium powder (20-230 mesh, 255 mg, 105 mmol). The flask was then flame dried under high vacuum and allowed to cool to room temperature under an atmosphere of argon. THF (5 mL) and dibromoethane (20 μL) were added. The resultant slurry was sonicated for one minute, then stirred vigorously until a gray precipitate was observed (ca. 30 min). Neat 4-bromobutene (3.55 mL, 350 mmol) was added slowly until an exotherm was observed indicating formation of the desired Grignard reagent. THF (30 mL) was then added, followed by dropwise addition of the remaining 4-bromobutene. The flask was then submerged in a 10–15 °C water/ice bath to cool the exotherm. Upon completion of the addition, the slurry was stirred for a further 1 hour at room temperature. Stirring was halted, and the precipitate was allowed to settle (ca. 15 min). The resulting solution of 4-butenylmagnesium bromide was titrated, and a concentration of 0.75 M was observed. To a 25 mL round bottom flask equipped with stir bar was added THF (5.3 mL) and 4-butenylmagnesium bromide (0.75 M, 2.7 mL, 2.0 mmol). The reaction vessel was cooled to –30 °C *via* $Me_2CO/CO_{2(s)}$ bath. Freshly purified CuI (190 mg, 1.0 mmol) was added to the reaction vessel. The reaction was allowed to stir at –30 °C for 30 minutes. The reaction vessel was then cooled to –78 °C *via* $Me_2CO/CO_{2(s)}$ bath. Neat TMSCl (0.20 mL, 1.6 mmol) was added dropwise *via* syringe to the reaction vessel. A solution of **hydrindenone 16** (100 mg, 0.667 mmol) in THF (1 mL) was then added dropwise *via* syringe to the reaction vessel. The reaction vessel was allowed to warm to –30 °C and allowed to stir at –30 °C for 5 hours. The vessel was then allowed to warm to 0 °C by submersion in an ice bath and the reaction was quenched at 0 °C by the dropwise addition of 2:1 $NH_4Cl_{(aq)}$:1 M $NaOH_{(aq)}$ (12 mL). The mixture was allowed to warm to room temperature, stirring open to air until a clear solution was observed (ca. 15 minutes). The aqueous layer was extracted with EtOAc (10 mL). The organic extracts were washed with 2:1 $NH_4Cl_{(aq)}$:1 M $NaOH_{(aq)}$ (3 mL), water (3 mL), and brine (3 mL), dried over $MgSO_4$, filtered, and concentrated *in vacuo*. The crude material was filtered through a plug of NEt_3 treated Florisil®, rinsing with hexanes/NEt_3

(99:1 v/v ratio). The filtrate was concentrated *in vacuo*, affording crude **enoxysilane 17** (145 mg, 78%) as a mixture of diastereomers (8:1 *dr*). The crude material was carried forward without further purification.

To a 10 mL round bottom flask equipped with stir bar was added crude **enoxysilane 17** (140 mg, 0.50 mmol) and 0.13 M $NaOH_{(MeOH)}$ (5 mL, 0.65 mmol). The flask was equipped with a reflux condenser then heated to 80 °C *via* oil bath. The reaction mixture was allowed to stir at 80 °C for 5 hours then allowed to cool to room temperature. The reaction mixture was concentrated *in vacuo*. The crude residue was dissolved in EtOAc (10 mL) and 1:1 $NH_4Cl_{(aq)}$:water (5 mL) and the layers were partitioned. The aqueous layer was extracted with EtOAc (5 mL). The organic extracts were washed with brine (3 mL), dried over $MgSO_4$, filtered, and concentrated *in vacuo*. The crude product was purified by column chromatography (SiO_2, 1% Et_2O in hexanes) affording ***trans*-hydrindanone 18** (54 mg, 51%, >20:1 dr) as a colorless oil and ***cis*-hydrindanone S6** (30 mg, 29%, 2:1 dr) as a colorless oil.

***trans*-hydrindanone 18**

^{1}H NMR (500 MHz, $CDCl_3$ at 7.27 ppm) δ 5.80 (ddt, 1H, *J* = 17.1, 10.3, 6.6), 4.98 (dq, 1H, *J* = 17.1, 1.7), 4.90 (d, 1H, *J* = 10.3), 2.26 (d, 1H, *J* = 14.4 Hz), 2.22-2.13 (m, 1H), 2.13-1.95 (m, 5H), 1.91-1.82 (m, 2H), 1.74 (td, 1H, *J* = 18.8, 5.0 Hz), 1.71-1.60 (m, 1H), 1.57-1.50 (m, 1H), 1.41-1.20 (m, 4H), 1.09 (s, 3H) ppm; **^{13}C NMR** (125 MHz, $CDCl_3$) δ 210.9, 139.8, 113.9, 64.9, 45.0, 42.6, 42.3, 41.8, 37.8, 31.9, 29.9, 29.2, 27.1, 22.2 ppm; **IR** (thin film) ν_{max} 3076, 2932, 2865, 1711, 1640 cm^{-1}; **HRMS** (ESI+) calculated for $C_{14}H_{22}O$ $[M+NH_4]^+$ 224.2014 found 224.2010.

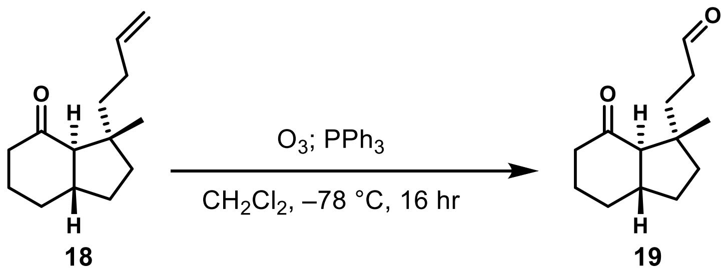

To a 100 mL round bottom flask equipped with a stir bar was added a solution of ***trans*-hydrindanone 18** (847 mg, 4.11 mmol) in CH_2Cl_2 (41 mL). The reaction vessel was cooled to –78 °C *via* $Me_2CO/CO_{2(s)}$ bath. Ozone gas was then bubbled through the solution until saturation. A color change to blue was observed. O_2 was then bubbled through the solution until the blue color dissipated. Triphenylphosphine (1.18 g, 4.52 mmol) was added to the reaction vessel. The vessel was then allowed to warm to room temperature. The reaction mixture was allowed to stir at room temperature for 16 hours, then passed through a plug of SiO_2 rinsing with Et_2O. The filtrate was concentrated *in vacuo*. The crude product was purified by column chromatography (SiO_2, 30% Et_2O in hexanes) affording **keto aldehyde 19** (787 mg, 92%) as a colorless oil.

keto aldehyde 19

^{1}H NMR (500 MHz, $CDCl_3$) δ 9.75 (t, 1H, *J* = 1.7 Hz), 2.55 (dddd, 1H, *J* = 17.2, 10.3, 5.4, 1.7 Hz), 2.42 (dddd, 1H, *J* = 17.2, 10.3, 5.4, 1.7 Hz), 2.30-2.23 (m, 1H), 2.17 (tdd, 1H, *J* = 13.3, 6.2, 1.0), 2.10-2.00 (m, 3H), 1.95 (ddd, 1H, *J* = 13.8, 10.3, 5.8), 1.87 (dt, 1H, *J* = 12.1, 8.0), 1.70-1.57 (m, 2H), 1.53-1.46 (m, 1H), 1.43-1.29 (m, 3H), 1.03 (s, 3H) ppm; **^{13}C NMR** (126 MHz, $CDCl_3$) δ 211.1, 203.4, 65.1, 45.5, 42.8, 42.1, 41.0, 38.5, 34.7, 32.0, 29.1, 27.3, 22.0 ppm; **IR** (thin film) ν_{max} 3175, 2937, 2866, 2719, 1708 cm^{-1} (aldehyde and ketone are overlapping); **HRMS** (ESI+) calculated for $C_{13}H_{20}O_2Na$ $[M+Na]^+$ 231.1361 found 231.1365.

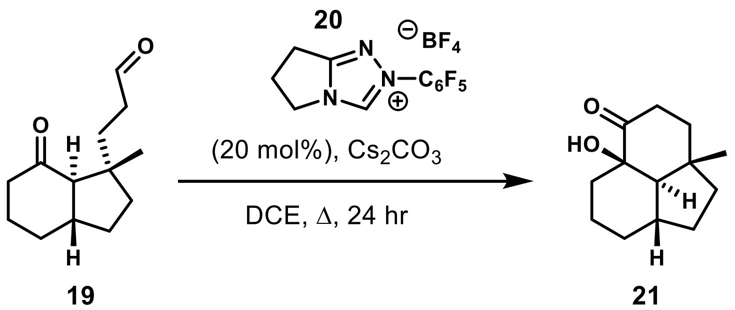

To a 3-necked 500 mL round bottom flask equipped with a stir bar and a reflux condenser was added Rovis's precatalyst **20**[5] (182 mg, 0.500 mmol), Cs_2CO_3 (160 mg, 0.500 mmol), and DCE (83 mL). The mixture was allowed to stir at room temperature for 30 minutes. A color change from yellow to orange was observed. A solution of **keto aldehyde 19** (348 mg, 1.67 mmol) in DCE (42 mL) was added to the reaction mixture. The reaction mixture was heated to reflux *via* oil bath. The reaction mixture was allowed to stir while heated for 24 hours. Upon consumption of starting material as indicated by TLC analysis, the reaction mixture was allowed to cool to room temperature, then quenched by the addition of $NH_4Cl_{(s)}$. The quenched reaction mixture was filtered through SiO_2, rinsing with Et_2O. The filtrate was concentrated *in vacuo*. The crude product was purified by column chromatography (SiO_2, 20% to 30% Et_2O in hexanes, stepped gradient) affording **hydroxyketone 21** (240 mg, 69%) as a white solid. **Hydroxyketone 21** was crystallized ***conditions to afford X-ray quality crystals.

In some cases, an inseparable impurity co-elutes with the product. If this is the case, the impurity can be removed *via* trituration with cold hexanes to afford pure **hydroxyketone 21**.

hydroxyketone 21
^{1}H NMR (500 MHz, $CDCl_3$), δ 3.0 (dd, 1H, *J* = 15.0, 14.0, 6.1 Hz), 2.20 (dq, 1H, *J* = 15.0, 2.1 Hz), 2.11-1.92 (m, 4H), 1.73-1.64 (m, 2H), 1.64-1.51 (m, 3H), 1.45-1.37 (m, 1H) ppm; **^{13}C NMR** (126 MHz, $CDCl_3$) δ 211.1, 76.8, 70.0, 39.8, 39.6, 35.2, 33.5, 32.9, 32.7, 28.7, 22.0, 20.0 ppm; **IR** (thin film) ν_{max} 3452, 2931, 2862, 1708 cm^{-1}; **m.p.** = 84–85 °C; **HRMS** (–ESI) calculated for $C_{13}H_{19}O_2$ [M–H]$^-$ 207.1385 found [M-H]$^-$ = 207.1379.

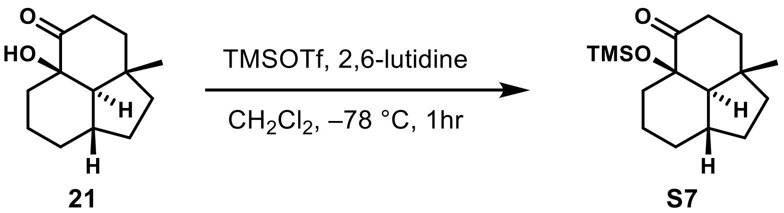

To a 50 mL round bottom flask equipped with a stir bar was added a solution of **hydroxyketone 21** (191 mg, 0.92 mmol) in CH_2Cl_2 (9.2 mL). The reaction vessel was cooled to –78 °C *via* $Me_2CO/CO_{2(s)}$ bath. 2,6-lutidine (540 µL, 4.59 mmol) and TMSOTf (420 µL, 2.29 mmol) were added to the reaction vessel at –78 °C. the reaction mixture was allowed to stir at –78 °C for 1 hour. Upon consumption of starting material as indicated by TLC analysis, the reaction was quenched by the addition of a solution of NEt_3 in MeOH (20 v/v%, 1 mL) at –78 °C. The reaction mixture was allowed to warm to room temperature then diluted with saturated NH_4Cl (10 mL) and CH_2Cl_2 (10 mL). The layers were partitioned, and the aqueous layer was extracted with CH_2Cl_2 (20 mL). The organic extracts were dried over $MgSO_4$, filtered, and concentrated *in vacuo*. The crude product was purified by column chromatography (SiO_2, 2% Et_2O in hexanes) affording **TMS-protected alcohol S7** (245 mg, 95%) as a colorless oil.

TMS-protected alcohol S7
^{1}H NMR (500 MHz, $CDCl_3$), δ 2.99 (td, 1H, *J* = 21.0, 6.0 Hz), 2.15 (ddd, 1H, *J* = 14.1, 4.2, 2.4 Hz), 2.10-2.004 (m, 1H), 1.98-1.87 (m, 3H), 1.78 (dt, 1H, *J* = 14.1, 2.9), 1.66-1.46 (m, 3H), 1.42-1.31 (m, 2H), 1.30-1.17 (m, 2H), 1.16 (s, 3H), 0.88 (qd, 1H, *J* = 21.0, 3.7 Hz), 0.85 (d, 1H, 12.4 Hz), 0.07 (s, 9H) ppm; **^{13}C NMR** (126 MHz, $CDCl_3$) *δ* 213.8, 79.8, 63.0, 40.6, 39.7, 39.2, 35.6, 33.5, 33.0, 31.7, 28.8, 22.2, 20.0,

1.7 ppm; **IR** (thin film) ν_{max} 2933, 2864, 1746 cm^{-1}; **HRMS** (ESI+) calculated for $C_{16}H_{28}O_2SiNa$ [M+Na]$^+$ 303.1756 found [M+H]$^+$ = 303.1747.

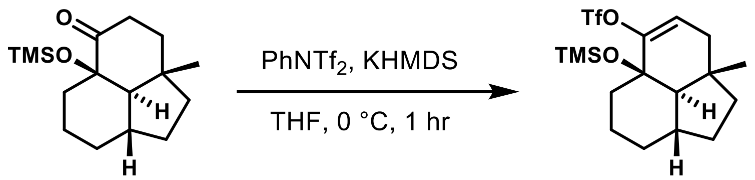

S7 **S8**

To a 100 mL round bottom flask equipped with a stir bar was added a solution of **TMS-protected alcohol S7** (245 mg, 0.875 mmol) and $PhNTf_2$ (626 mg, 1.75 mmol) in THF (20.2 mL). The reaction vessel was cooled to 0 °C *via* ice bath. KHMDS (0.44 M in PhMe, 2.97 mL, 1.31 mmol) was added dropwise *via* syringe to the reaction vessel. The reaction mixture was allowed to stir at 0 °C for 1 hour. Upon consumption of starting material as indicated by TLC analysis, the reaction was quenched by the addition of saturated $NaHCO_{3(aq)}$ (20 mL). The aqueous layer was extracted with Et_2O (3 x 40 mL). The organic extracts were dried over $MgSO_4$, filtered, and concentrated *in vacuo*. The crude product was purified by column chromatography (SiO_2, hexanes) affording **alkenyl triflate S8** (336 mg, 93%) as a colorless oil.

alkenyl triflate S8

^{1}H NMR (600 MHz, $CDCl_3$) δ 5.77 (dd, 1H, *J* = 5.6, 2.4 Hz), 2.28 (dd, 1H, *J* = 17.6, 5.6 Hz), 2.15 (dd, 1H, *J* = 17.6, 2.0 Hz), 2.07-1.88 (m, 4H), 1.69-1.60 (m, 2H), 1.55 (ddd, 1H, *J* = 12.8, 9.2, 3.6 Hz), 1.42 (td, 1H, *J* = 12.8, 6.1 Hz), 1.31-1.23 (m, 2H), 1.16 (d, 1H, 12.8 Hz), 1.06-0.97 (m, 1H), 0.96 (s, 3H), 0.12 (s, 9H) ppm; **^{13}C NMR** (126 MHz, $CDCl_3$) δ 152.8, 120.8, 118.6 (q, *J* = 318 Hz), 73.2, 60.7 ,40.3, 39.8, 37.5, 34.4, 37.5, 33.2, 33.2, 29.0, 22.1, 21.7, 1.6 ppm; **IR** (thin film) ν_{max} 3456, 3404, 2955, 2937, 2867, 1651, 1456, 1417, 1341, 1249, 1144; **HRMS** (ESI+) calculated for $C_{17}H_{27}F_3O_4SSiNa$ [M+Na]$^+$ 435.1249 found [M+Na]$^+$ 435.1252.

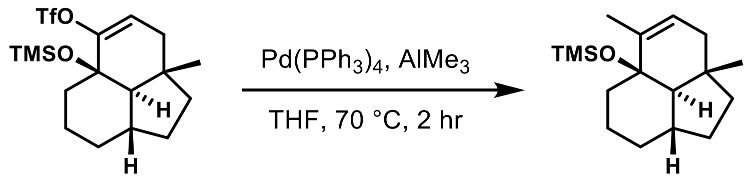

S8 **S9**

To a 100 mL round bottom flask equipped with a stir bar was added **alkenyl triflate S8** (815 mg, 1.98 mmol) and degassed THF (9.9 mL). The reaction vessel was cooled to 0 °C *via* ice bath. A solution of $Pd(PPh_3)_4$ in degassed THF (9.9 mL) was added to the reaction mixture at 0 °C. The reaction mixture was allowed to stir at 0 °C for 5 minutes, then $AlMe_3$ (2.0 M in heptane, 4.94 mL, 9.88 mmol) was added dropwise *via* syringe at 0 °C. The reaction mixture was allowed to stir at 0 °C for 5 minutes, then allowed to warm to room temperature. Upon warming to room temperature, the reaction vessel was equipped with a reflux condenser. The reaction vessel was then heated to 70 °C *via* oil bath. The reaction mixture was allowed to stir at 70 °C for 2 hours. Upon consumption of starting material as indicated by TLC analysis, the reaction vessel was cooled to 0 °C. The reaction was quenched at 0 °C by the dropwise addition of saturated $NaHCO_{3(aq)}$ (25 mL). The mixture was allowed to warm to room temperature, and saturated Rochelle's salt (25 mL) was added. The mixture was allowed to stir vigorously at room temperature for 2 hours, then extracted with Et_2O (3 x 100 mL). The organic extracts were dried over $MgSO_4$, filtered, and concentrated *in vacuo*. The crude product was purified by column chromatography (SiO_2, hexanes) affording **alkene S9** (479 mg, 87%) as a colorless oil.

alkene S9

^{1}H NMR (600 MHz, $CDCl_3$), δ 5.36 (tq, 1H, *J* = 3.6, 1.4 Hz), 2.10 (ddq, 1H, *J* = 17.2, 5.1, 1.4 Hz), 2.00-1.80 (m, 5H), 1.75 (quint, 1H, *J* = 1.4 Hz), 1.66 (qt, 1H, *J* = 13.0, 3.9 Hz), 1.58 (dtt, 1H, *J* = 13.3, 2.3, 4.5 Hz), 1.49 (ddd, 1H, *J* = 12.5, 9.0, 3.6 Hz), 1.34 (td, 1H, *J* = 12.5, 6.0 Hz), 1.16 (tdd, 1H, *J* = 12.5, 8.0, 3.6 Hz), 1.03 (td, 1H, 13.3, 4.5), 0.95 (qd, 1H, *J* = 12.5, 4.5 Hz), 0.89 (s, 3H), 0.09 (s, 9H) ppm; **^{13}C NMR** (150

MHz, $CDCl_3$),δ 136.8, 125.9, 74.1, 59.8, 42.7, 40.8, 37.6, 36.1, 33.8, 33.7, 28.6, 22.8, 22.1, 18.0, 2.4 ppm; **IR** (thin film) ν_{max} 3405 (br), 3010, 2945, 2863, 2671, 2359, 2339, 2091, 1644 cm^{-1}; **HRMS** (CI) calculated for $C_{17}H_{30}OSi$ $[M]^+$ 278.2066 found $[M]^+$ 278.2060.

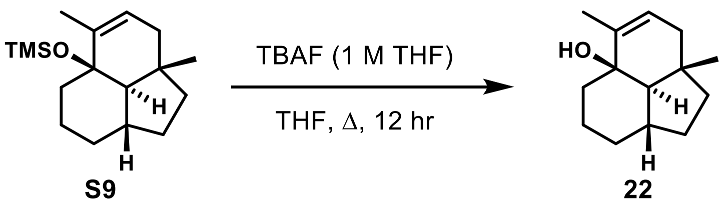

To a 50 mL round bottom flask equipped with a stir bar was added a solution of **alkene S9** (479 mg, 1.72 mmol) in THF (8.6 mL) followed by TBAF (1.0 M in THF, 8.6 mL, 8.6 mmol). The reaction mixture was heated to reflux *via* oil bath. The reaction mixture was allowed to stir while at reflux for 12 hours. Upon consumption of starting material as indicated by TLC analysis, the reaction vessel was cooled to room temperature. The reaction mixture was filtered through a plug of SiO_2, rinsing with Et_2O. The filtrate was concentrated *in vacuo*. The crude product was purified by column chromatography (SiO_2, 6% Et_2O in hexanes) affording **allylic alcohol 22** (356 mg, 99%) as a colorless oil.

allylic alcohol 22

^{1}H NMR (600 MHz, $CDCl_3$), δ 5.38 (tq, 1H, *J* = 3.6, 1.4 Hz), 2.12 (ddq, 1H, *J* = 17.0, 5.5, 1.2 Hz), 2.04-1.87 (m, 4H), 1.84 (ddd, 1H, *J* = 13.2, 3.7, 3.0 Hz), 1.74 (quint, 3H, *J* = 1.3 Hz), 1.69 (dtd, 1H, *J* = 12.7, 3.7, 1.0), 1.66 (dtt, 1H, *J* = 13.8, 2.8, 4.7 Hz), 1.54 (ddd, 1H, *J* = 12.7, 9.1, 3.7), 1.40 (td, 1H, *J* = 12.7, 6.1), 1.22 (tdd, 1H, *J* = 12.7, 8.0, 3.7 Hz) ppm; **^{13}C NMR** (150 MHz, $CDCl_3$), δ 138.1, 124.7, 71.6, 58.7, 42.2, 40.6, 37.6, 36.0, 33.8, 33.7, 28.5, 22.6, 21.6, 16.5 ppm; **IR** (thin film) ν_{max} 3625, 3499 (br), 3009, 2933, 2862, 1714, 1642 cm^{-1}; **HRMS** (ESI+) calculated for $C_{14}H_{21}$ $[M–OH]^+$ 189.1643 found $[M–OH]^+$ 189.1637.

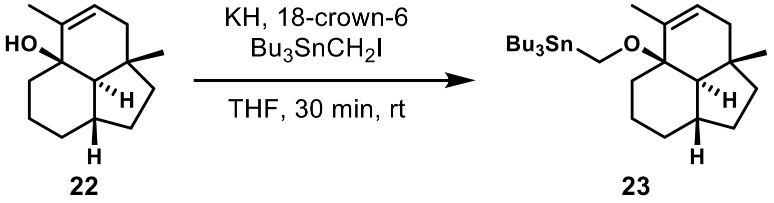

To a 2-dram vial equipped with a stir bar was added KH (30 wt % in mineral oil, 40 mg, 0.296 mmol). The solids were slurried in hexanes (1 mL) then allowed to settle to the bottom of the vessel and the supernatant was removed *via* syringe. This process was repeated three times to ensure the removal of mineral oil from the reaction vessel. THF (1 mL) was added to the reaction vessel and the mixture was allowed to stir at room temperature until a suspension was formed. The reaction vessel was cooled to 0 °C *via* ice bath. A solution of **allylic alcohol 22** (30.5 mg, 0.148 mmol) in THF (250 µL) was added to the reaction vessel dropwise *via* syringe at 0 °C. The reaction mixture was allowed to warm to room temperature. 18-crown-6 (46.9 mg, 0.178 mmol) was added to the reaction mixture at room temperature, and the reaction mixture was allowed to stir at room temperature for 1 hour. A solution of Bu_3SnCH_2I (63.8 mg, 0.148 mmol) in THF (250 µL) was added dropwise *via* syringe to the reaction vessel, and the reaction mixture was allowed to stir at room temperature for 30 minutes. Upon consumption of starting material as indicated by TLC analysis, the reaction was quenched at room temperature by the addition of saturated $NH_4Cl_{(aq)}$ (3 mL). The mixture was extracted with Et_2O (3 x 6 mL). The organic extracts were dried over $MgSO_4$, filtered, and concentrated *in vacuo*. The crude product was purified by column chromatography (SiO_2, hexanes) affording **stannylmethyl ether 23** (67.1 mg, 89%) as a colorless oil.

stannylmethyl ether 23

^{1}H NMR (600 MHz, $CDCl_3$), δ 5.46 (ddd, 1H, *J* = 4.5, 2.5, 1.5), 3.67 (td, 1H, *J* = 11.9, 8.9 Hz), 3.58 (q, 1H, *J* = 8.9 Hz), 2.31 (dt, 1H, *J* = 13.8, 3.9 Hz), 2.10 (ddq, 1H, *J* = 17.4, 4.5, 1.5), 2.04-1.96 (m, 1H), 1.96-1.88 (m, 2H), 1.86 (quint, 3H, *J* = 1.0 Hz), 1.85-1.80 (m, 1H), 1.59-1.40 (m, 8H), 1.35-1.24 (m, 6H), 1.13 (tdd, 1H, *J* = 12.5, 8.0, 4.0), 1.05-0.69 (m, 15H) ppm; **^{13}C NMR** (150 MHz, $CDCl_3$) δ 136.1, 127.7, 75.9, 59.9, 52.9, 43.1, 40.8, 37.5, 33.8, 29.9, 29.4, 28.7, 27.6, 23.2, 22.8, 20.9, 13.9, 8.9 ppm; **IR** (thin film) ν_{max} 2954, 2925, 2869, 2853, 1457 cm^{-1}; **HRMS** (CI+) calculated for $C_{23}H_{41}OSn$ $[M–Bu]^+$ 453.2184 found $[M–Bu]^+$ 453.2168.

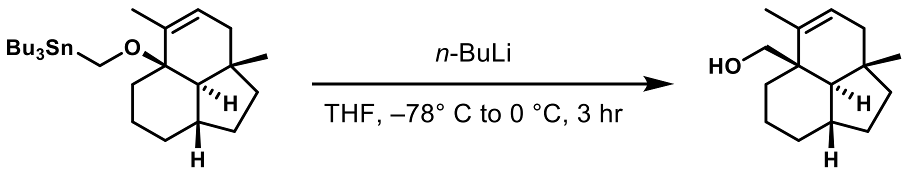

23
26

To a 1-dram vial equipped with a stir bar was added a solution of **stannylmethyl ether 23** (23.5 mg, 0.046 mmol) in THF (300 μL). The solution was cooled to –78 °C *via* $Me_2CO/CO_{2(s)}$ bath. *n*-BuLi (2.5 M in hexane, 22 μL, 0.055 mmol) was added dropwise *via* syringe to the reaction vessel at –78 °C. The reaction mixture was allowed to warm slowly to 0 °C while stirring for 3 hours. The reaction was then quenched by the addition of saturated $NH_4Cl_{(aq)}$ (1 mL). The resultant slurry was extracted with Et_2O (3 x 2 mL). The organic extracts were dried over $MgSO_4$, filtered, and concentrated *in vacuo*. The crude product was purified by column chromatography (SiO_2, 0% to 4% Et_2O in hexanes, stepped gradient) affording **alcohol 26** (2.1 mg 21%) as a white solid.

alcohol 26
^{1}H NMR (600 MHz, $CDCl_3$) δ 5.34-5.31 (m, 1H), 3.91 (d, 1H, *J* = 11.0 Hz), 3.83 (d, 1H, *J* = 11 Hz), 2.20 (dt, 1H, *J* = 12.9, 3.3 Hz), 2.10-1.98 (m, 2H), 1.93-1.81 (m, 2H), 1.77 (dt, 3H, *J* = 2.5, 1.4 Hz), 1.70-1.65 (m, 1H), 1.60-1.50 (m, 2H)***,** 1.45 (qt, 1H, *J* = 13.5, 3.8 Hz), 1.40-1.33 (m, 1H), 1.32-1.15 (m, 2H)* 1.04-0.87 (m, 3H), 0.85 (s, 3H) ppm; **^{13}C NMR** (600 MHz, $CDCl_3$) δ 140.3, 123.3, 64.9, 59.8, 43.0, 42.5, 41.9, 37.8, 34.6, 33.9, 33.6, 29.9, 28.9, 23.2, 23.0, 19.5, 14.3 ppm; **IR** (thin film) ν_{max} 3369, 2923, 2862 cm^{-1}; **HRMS** (ESI+) calculated for $C_{15}H_{24}ONa$ $[M+Na]^+$ 243.1725 found $[M+Na]^+$ 243.1716.

*Due to the small amount of material available, integration of multiplets in these regions were difficult to determine.

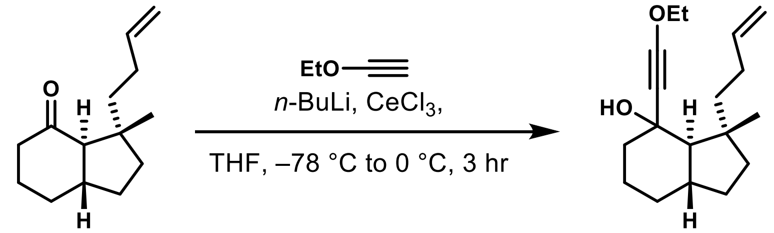

18
29

To a 25 mL round bottom flask equipped with a stir bar was added a solution of ethoxyacetylene (183 μL, 2.09 mmol) in THF (5.3 mL). The solution was cooled to –78 °C *via* $Me_2CO/CO_{2(s)}$ bath. *n*-BuLi (2.6 M in hexane, 540 μL, 1.40 mmol) was added dropwise *via* syringe at –78 °C to the reaction vessel. The reaction mixture was allowed to stir at –78 °C for 30 minutes, then allowed to warm to 0 °C by submersion in an ice bath. The reaction mixture was allowed to stir at 0 °C for 30 minutes, then cooled to –78 °C. Anhydrous $CeCl_3$ (413 mg, 1.67 mmol) was added to the reaction mixture in a single portion under a stream of argon. The reaction mixture was allowed to stir at –78 °C for 30 minutes. A solution of ***trans*-hydrindanone 18** (57.6 mg, 0.279 mmol) in THF (200 μL) was added dropwise *via* syringe to the reaction vessel at –78 °C, rinsing with THF (100 μL). The reaction mixture was allowed to stir at –78 °C, then warmed slowly to 0 °C while stirring over the course of 3 hours. A color change from white to light brown was observed. After 3 hours, the reaction mixture was allowed to warm to room temperature. The reaction mixture was allowed to stir at room temperature for 30 minutes, then quenched at room temperature by the addition of $NH_4Cl_{(s)}$. The resultant slurry was allowed to stir vigorously at room temperature for 15 minutes, then diluted with hexanes (10 mL) and filtered through a plug of SiO_2, rinsing with Et_2O. The filtrate was concentrated *in vacuo*. The crude product was purified by column chromatography (SiO_2, 15% to 20% Et_2O in hexanes, stepped gradient) affording **alkynyl ether 29** (265 mg, 96%) as a colorless oil (inconsequential 1.6:1 mixture of diastereomers).

Note: individual diastereomers were isolated for characterization purposes only. Otherwise, the diastereomeric mixture was isolated together and taken onto the next step.

major diastereomer of alkynyl ether 29

^{1}H NMR (500 MHz, $CDCl_3$) δ 5.84 (ddt, 1H, *J* = 17.0, 10.3, 6.6 Hz), 5.02 (ddd, 1H, *J* = 17.0, 3.1, 1.4 Hz), 4.90 (ddd, 1H, *J* = 10.3, 1.7, 1.0 Hz), 4.06 (q, 2H, *J* = 7.1 Hz), 2.08 (dt, 2H, *J* = 8.6, 7.4 Hz), 1.93-1.84 (m, 3H), 1.71 (dddd, 1H, *J* = 12.2, 8.7, 6.2, 2.4 Hz), 1.67-1.49 (m, 5H), 1.35 (t, 3H, *J* = 7.1 Hz), 1.27-1.19 (m, 2H), 1.14 (s, 3H), 1.11 (d, 1H, *J* = 12.1 Hz), 1.05-0.91 (m, 2H) ppm; **^{13}C NMR** (126 MHz, $CDCl_3$) δ 140.1, 113.7, 93.2, 74.3, 68.3, 60.1, 44.0, 43.3, 42.8, 38.1, 38.1, 32.8, 30.0, 29.8, 25.2, 21.6, 14.6 ppm; **IR** (thin film) ν_{max} 3478, 3075, 2974, 2933, 2860, 2260, 1722, 1639 cm^{-1}; **HRMS** (ESI+) calculated for $C_{18}H_{27}O$ $[M–OH]^+$ 259.2062 found 259.2059, calculated for $C_{18}H_{29}O_2$ $[M+H]^+$ 277.2168 found 277.2163.

minor diastereomer of alkynyl ether 29

^{1}H NMR (500 MHz, $CDCl_3$) δ 5.83 (ddt, 1H, *J* = 17.0, 10.3, 6.6 Hz), 4.99 (dq, 1H, *J* = 17.0, 1.5 Hz), 4.89 (d, 1H, *J* = 10.3), 4.07 (q, 2H, *J* = 7.1 Hz), 2.11 (ddd, 1H, *J* = 18.8, 13.0, 6.0 Hz), 2.04 (ddd, 1H, *J* = 18.8, 13.0, 6.0 Hz), 1.88-1.71 (m, 5H), 1.71-1.64 (m, 1H), 1.64-1.54 (m, 2H), 1.46 (td, 1H, *J* = 12.3, 4.0 Hz), 1.42 (td, 1H, *J* = 12.7, 4.8 Hz), 1.36 (t, 3H, 7.1 Hz), 1.27 (dt, 1H, *J* = 13.3, 8.3 Hz), 1.20 (s, 3H), 1.15 (d, 1H, 11.6 Hz), 1.15-1.04 (m, 1H), 0.92 (qd, 1H, *J* = 12.6, 4.3 Hz) ppm; **^{13}C NMR** (126 MHz, $CDCl_3$) δ 140.2, 113.7, 96.3, 74.3, 72.7, 62.5, 45.7, 44.1, 41.7, 41.4, 38.4, 32.7, 29.9, 29.8, 24.8, 24.0, 14.7 ppm; **IR** (thin film) 3478, 3074, 2972, 2932, 2859, 2258, 1640 cm^{-1}; **HRMS** (ESI+) calculated for $C_{18}H_{28}O_2Na$ $[M+Na]^+$ 299.1987 found 299.1986.

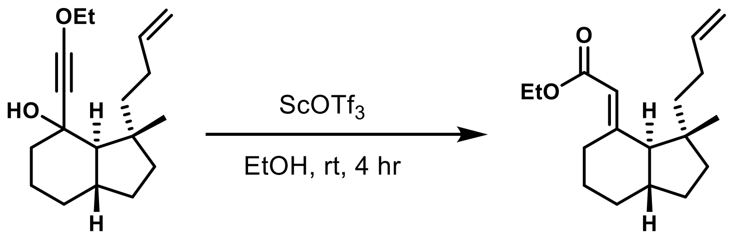

29 30

To a 50 mL round bottom flask equipped with a stir bar was added **alkynyl ether 29** (246.7 mg, 0.892 mmol), CH_2Cl_2 (14.9 mL), EtOH (0.52 mL, 8.92 mmol), and $Sc(OTf)_3$ (43.9 mg, 0.0892 mmol). The reaction mixture was allowed to stir at room temperature for 4 hours, then diluted with hexanes (20 mL) and filtered through a plug of pH 7 SiO_2, rinsing with Et_2O. The filtrate was concentrated *in vacuo*. The crude product was purified by column chromatography (SiO_2, 2% Et_2O in hexanes) affording **enoate ester 30** (231 mg, 94%) as a colorless oil (inconsequential 2:1 mixture of diastereomers).

Note: individual diastereomers were isolated for characterization purposes only. Otherwise, the diastereomeric mixture was isolated together and taken onto the next step.

major diastereomer of enoate ester 30

^{1}H NMR (500 MHz, $CDCl_3$) δ 5.82 (ddt, 1H, *J* = 17.0, 10.2, 6.5 Hz), 5.78 (t, 1H, *J* = 1.5 Hz), 4.99 (ddd, 1H, *J* = 17.0, 1.7, 1.4 Hz), 4.89 (d, 1H, *J* = 10.2 Hz), 4.14 (dq, 1H, *J* = 10.9, 7.1 Hz), 4.06 (dq, 1H, *J* = 10.9, 7.1 Hz), 2.76 (dt, 1H, *J* = 12.5, 1.4 Hz), 2.41 (tdd, 1H, *J* = 12.2, 8.0, 1.4 Hz), 2.14 (dddd, 1H, *J* = 25.1, 12.5, 5.8, 3.3 Hz), 2.08-1.96 (m, 3H), 1.95-1.85 (m, 1H), 1.78 (q, 2H, *J* = 12.6 Hz), 1.66 - 1.59 (m, 2H), 1.49-1.41 (m, 2H), 1.26 (t, 3H, *J* = 7.1 Hz), 1.18-1.07 (m, 2H), 0.87 (s, 3H) ppm; **^{13}C NMR** (126 MHz, $CDCl_3$) δ 167.5, 160.7, 140.1, 116.6, 113.7, 59.8, 56.0, 45.6, 42.5, 40.4, 37.9, 34.9, 29.9, 28.3, 24.8, 24.1, 23.0, 14.4 ppm; **IR** (thin film) ν_{max} 3075, 2928, 2862, 1720, 1639 cm^{-1}; **HRMS** (ESI+) calculated for $C_{18}H_{29}O_2$ $[M+H]^+$ 277.2168 found 277.2172.

minor diastereomer of enoate ester 30

^{1}H NMR (500 MHz, $CDCl_3$) δ 5.83 (ddt, 1H, *J* = 17.0, 10.2, 6.5 Hz), 5.69 (s, 1H), 5.01 (d, 1H, *J* = 17.0 Hz), 4.92 (d, 1H, *J* = 10.2 Hz), 4.14 (q, 2H, *J* = 7.1 Hz), 3.92 (dt, 1H, *J* = 13.7, 2.8 Hz), 2.18-2.08 (m, 1H), 2.08-1.94 (m, 2H), 1.93 -1.76 (m, 4H), 1.71-1.61 (m, 2H), 1.55 - 1.50 (m, 1H), 1.47-1.33 (m, 3H), 1.28 (t, 3H, *J* = 7.1 Hz), 1.26-1.16 (m, 2H), 1.06 (s, 3H) ppm; **^{13}C NMR** (126 MHz, $CDCl_3$) δ 167.2, 162.2, 139.6,

114.0, 111.9, 59.7, 59.6, 44.2, 41.2, 43.1, 39.0, 33.5, 30.4, 29.4, 27.8, 27.2, 20.9, 14.4 ppm; **IR** (thin film) ν_{max} 3075, 2929, 2863, 1717, 1641 cm^{-1}; **HRMS** (ESI+) calculated for $C_{18}H_{29}O_2$ $[M+H]^+$ 277.2168 found 277.2169

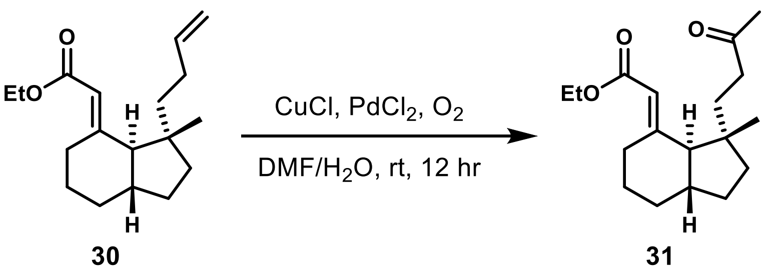

To a 50 mL round bottom flask equipped with a stir bar was added **enoate ester 30** (209.4 mg, 0.752 mmol), a mixture of DMF/H_2O (10:1, 7.52 mL), CuCl (112 mg, 1.13 mmol), and $PdCl_2$ (33.3 mg, 0.188 mmol). The reaction mixture was sparged with $O_{2(g)}$ while stirring at room temperature for 30 minutes, then allowed to stir at room temperature under an atmosphere of $O_{2(g)}$ for 12 hours. Upon consumption of starting material as indicated by TLC analysis, the reaction mixture was filtered through Celite, rinsing with Et_2O (30 mL). The filtrate was washed with 1:1 brine:water (3 x 10 mL), brine (1 x 10 mL), dried over $MgSO_4$, filtered, and concentrated *in vacuo*. The crude product was purified by column chromatography (SiO_2, 20% Et_2O in hexanes) affording **keto ester 31** (202 mg, 92%) as a colorless oil (inconsequential 2:1 mixture of diastereomers).

major diastereomer of keto ester 31

^{1}H NMR (500 MHz, $CDCl_3$) δ 5.78 (t, 1H, *J* = 1.8 Hz), 4.09 (tq, 2H, *J* = 10.8, 3.7 Hz, this is more likely 2 dq's, one for each methylene hydrogen of the ethyl ester which is split by 10.8 Hz), 2.78 (dt, 1H, *J* = 12.5, 1.3 Hz), 2.61 (ddd, 1H, *J* = 16.3, 8.6, 7.6 Hz), 2.45-2.31 (m, 2H), 2.15 (s, 3H), 2.14-2.07 (m, 1H), 1.99 (ddd, 1H, *J* = 12.7, 6.5, 1.5 Hz), 1.94-1.85 (m, 1H), 1.80 (tdd, 1H, *J* = 10.8, 3.0, 1.7 Hz), 1.72-1.56 (m, 4H), 1.43 (dt, 1H, *J* = 13.3, 8.7 Hz), 1.26 (t, 3H, *J* = 7.1 Hz), 1.23-1.03 (m, 2H), 0.87 (s, 3H) ppm; **^{13}C NMR** (126 MHz, $CDCl_3$) δ 210.1, 167.5, 160.8, 116.7, 59.9, 54.6, 44.8, 40.7, 40.0, 37.8, 36.3, 34.8, 30.0, 28.8, 24.7, 24.2, 23.9, 14.3 ppm; **IR** (thin film) ν_{max} 2944, 2864, 1716, 1638 cm^{-1}; **HRMS** (ESI+) calculated for $C_{18}H_{29}O_3$ $[M+H]^+$ 293.2117 found 293.2130.

Note: The ^{1}H NMR spectra of this compound is contaminated with ~2% of an aldehyde tentatively assigned from anti-Markovnikov oxidation.

minor diastereomer of keto ester 31

^{1}H NMR (500 MHz, $CDCl_3$) δ 5.67 (t, 1H, *J* = 1.5 Hz), 4.131 (q, 1H, *J* = 7.1 Hz), 4.128 (q, 1H, *J* = 7.1 Hz), 3.93 (td, 1H, *J* = 14.0, 3.0 Hz), 2.52 (ddd, 1H, *J* = 16.7, 11.8, 4.9 Hz), 2.38 (dddd, 1H, *J* = 16.7, 11.8, 4.9 Hz), 2.16 (s, 3H), 2.04-1.94 (m, 2H), 1.91-1.77 (m, 2H), 1.69-1.53 (m, 3H), 1.51-1.32 (m, 2H), 1.27 (t, 3H, *J* = 7.1 Hz), 1.28-1.67 (m, 2H), 1.05 (s, 3H) ppm; **^{13}C NMR** (126 MHz, $CDCl_3$) δ 209.4, 167.2, 161.8, 112.1, 59.8, 59.7, 44.3, 42.6, 39.8, 39.0, 34.6, 33.5, 30.4, 30.1, 27.8, 27.2, 20.9, 14.5 ppm; **IR** (thin film) ν_{max} 2931, 2864, 2359, 2339, 1714, 1643 cm^{-1}; **HRMS** (ESI+) calculated for $C_{18}H_{28}O_3Na$ $[M+Na]^+$ 315.1936 found 315.1929.

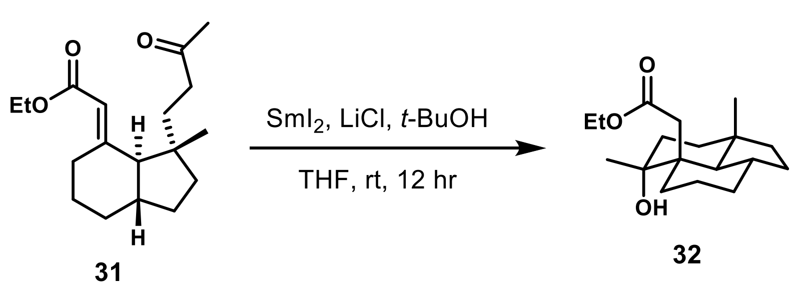

To a 2-dram vial equipped with a stir bar in a glove box was added anhydrous LiCl (40.0 mg, 0.944 mmol). The vessel was removed from the glovebox and placed under an atmosphere of argon. SmI_2[6] (0.1 M in THF, 1.89 mL, 0.189 mmol) was added to the reaction vessel. The mixture was allowed to stir vigorously for 30 minutes at room temperature. A color change to dark green was observed.

The reaction mixture was then cooled to –78 °C *via* $Me_2CO/CO_{2(s)}$ bath. A solution of **keto ester 31** (18.4 mg, 0.0629 mmol) and *t*-BuOH (6.6 µL, 0.0692 mmol) in degassed THF (1.79 mL) was added dropwise *via* syringe to the reaction mixture at –78 °C, rinsing with degassed THF (0.2 mL). The reaction vessel was allowed to warm to room temperature, then allowed to stir at room temperature for 12 hours. Upon consumption of starting material as indicated by TLC analysis, the reaction was quenched at room temperature by the addition of 1:1 saturated $NaHCO_{3(aq)}$:brine (3 mL). The resultant slurry was allowed to stir vigorously at room temperature for 20 minutes, then extracted with Et_2O (3 x 6 mL). The organic extracts were dried over $MgSO_4$, filtered, and concentrated *in vacuo*. The crude product was purified by column chromatography (SiO_2, 30% Et_2O in hexanes) affording **hydroxyester 32** (15.6 mg, 84%) as a colorless oil.

hydroxyester 32

^{1}H NMR (500 MHz, C_6D_6) δ 3.96 (qd, 1H, *J* = 10.8, 7.1 Hz), 3.95 (qd, 1H, *J* = 10.8, 7.1 Hz), 2.45 (d, 1H, *J* = 15.5 Hz), 2.29 (dt, 1H, *J* = 12.7, 3.0 Hz), 2.26 (d, 1H, *J* = 15.5 Hz), 1.91 (dq, 1H, *J* = 12.1, 3.4 Hz), 1.82-1.45 (m, 7H), 1.40 (d, 1H, *J* = 13.0 Hz), 1.37 (dt, 1H, *J* = 12.1, 3.4 Hz), 1.31-1.19 (m, 3H), 1.23 (s, 3H), 1.18-1.10 (m, 1H), 0.99 (t, 3H, *J* = 7.1 Hz), 0.97-0.91 (m, 1H), 0.67 (s, 3H); **^{13}C NMR** (126 MHz, C_6D_6) δ 172.3, 73.5, 59.8, 54.5, 45.2, 41.9, 40.0, 35.5, 35.2, 34.6, 34.4, 34.1, 28.4, 27.6, 26.0, 23.6, 20.8, 14.2 ppm; **IR** (thin film) 3527, 2977, 2935, 2864, 1735, 1714 cm^{-1}; **HRMS** (ESI+) calculated for $C_{18}H_{30}O_3Na$ $[M+Na]^+$ 317.2093 found 317.2094.

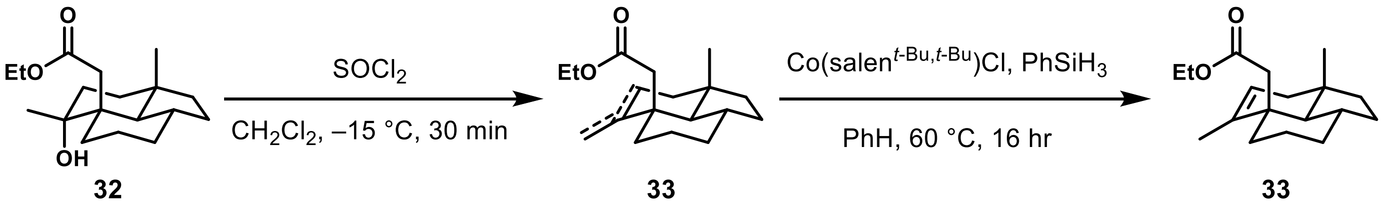

To a 2-dram vial equipped with a stir bar was added **hydroxyester 32** (26.1 mg, 0.0893 mmol), pyridine (72.0 µL, 0.893 mmol), and CH_2Cl_2 (0.72 mL). The mixture was cooled to –15 °C *via* $MeOH/H_2O/CO_{2(s)}$ bath. A solution of $SOCl_2$ (7.8 µL, 0.107 mmol) in CH_2Cl_2 (0.1 mL) was added dropwise *via* syringe to the reaction vessel at –15 °C. The reaction mixture was allowed to stir at –15 °C for 30 minutes. Upon consumption of starting material as indicated by TLC analysis, the reaction was quenched at –15 °C by the addition of saturated $NaHCO_{3(aq)}$ (1 mL). The layers were partitioned, and the aqueous layer was extracted with CH_2Cl_2 (3 x 2 mL). The organic extracts were dried over $MgSO_4$, filtered, and concentrated *in vacuo*, affording crude **alkene 33** (2.8:1 mixture of *endo:exo*). The crude mixture was carried forward without further purification.

To a 2-dram vial equipped with stir bar was added the crude mixture of **alkenes 33**, Co(salen^{tBu,tBu})Cl (1.47 mg, 2.31 µmol), degassed benzene (0.67 mL), and a solution of $PhSiH_3$ (0.57 µL, 4.63 µmol) in degassed benzene (0.1 mL). The vial was sealed under a stream of argon, then heated to 60 °C *via* oil bath. The reaction mixture was allowed to stir at 60 °C for 16 hours, then allowed to cool to room temperature. The reaction mixture was concentrated *in vacuo*. The resultant residue was purified by column chromatography (SiO_2, 2% Et_2O in hexanes) affording **alkene 33** (17.7 mg, 71%) as a colorless oil.

Note: The α-ester methylene and ethyl ester methylene protons are distinct owing to slow equilibration of rotamers.

alkene 33

^{1}H NMR (500 MHz, $CDCl_3$) δ 5.28-5.25 (m, 1H), 4.13 (dq, 1H, *J* = 10.8, 7.1 Hz), 4.07 (dq, 1H, *J* = 10.8, 7.1 Hz), 4.03 (dt, 1H, *J* = 10.8, 7.1 Hz, rotamer), 3.96 (dt, 1H, *J* = 10.8, 7.1 Hz, rotamer), 2.71 (d, 1H, *J* = 13.4 Hz, rotamer), 2.70 (d, 1H, *J* = 13.4 Hz), 2.44 (dd, 1H, *J* = 13.4, 0.7 Hz, rotamer), 2.43 (dd, 1H, *J* = 13.4, 0.7 Hz), 2.26 (dt, 1H, *J* = 13.0, 3.2 Hz), 2.08 (ddq, 1H, *J* = 17.5, 4.8, 1.5 Hz), 2.05-1.96 (m, 2H), 1.91 (dtd, 1H, *J* = 13.0, 9.3, 7.2 Hz), 1.87-1.77 (m, 1H), 1.70-1.60 (m, 3H), 1.53 (ddd, 1H, *J* = 12.5, 9.2, 3.3 Hz), 1.37 (td, 1H, *J* = 12.0, 7.3 Hz), 1.25 (t, 3H, *J* = 7.1 Hz), 1.24-1.17 (m, 1H), 1.18 (d, 1H, *J* = 12.8 Hz), 1.05-0.91 (m, 2H), 0.89 (s, 3H) ppm; **^{13}C NMR** (126 MHz, $CDCl_3$) 173.7, 141.1, 122.9, 60.9, 60.2, 42.6, 42.2, 41.0, 38.0, 37.4, 34.7, 33.8, 28.8, 23.3, 23.0, 18.7, 14.3 ppm; **IR** (thin film) ν_{max} 2936, 2865, 2831, 1731 cm^{-1}; **HRMS** (ESI+) calculated for $C_{18}H_{28}O_2Na$ $[M+Na]^+$ 299.1987 found 299.1994.

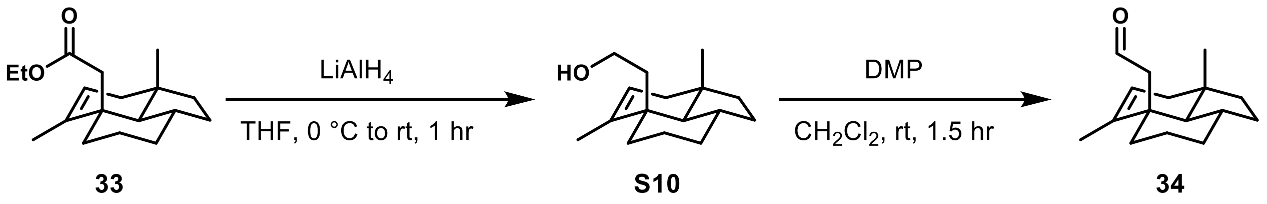

To a 2-dram vial equipped with a stir bar was added THF (1 mL) and $LiAlH_4$ (15.8 mg, 0.417 mmol). The slurry was cooled to 0 °C *via* ice bath. A solution of **alkene 33** (38.4 mg, 0.139 mmol) in THF (0.2 mL) was added dropwise *via* syringe at 0 °C to the reaction vessel, rinsing with THF (0.2 mL). The reaction mixture was allowed to stir at 0 °C for 30 minutes, warmed to room temperature, and allowed to stir at room temperature for 30 minutes. Upon consumption of starting material as indicated by TLC analysis, the reaction mixture was cooled to 0 °C. H_2O (16 µL), of 15 w/v% $NaOH_{(aq)}$ (16 µL) and H_2O (48 µL) were added to the reaction mixture at 0 °C. The resultant slurry was allowed to warm to room temperature then allowed to stir at room temperature for 20 minutes. $MgSO_4$ was added to the mixture, and the resultant slurry was filtered through $MgSO_4$, rinsing with Et_2O. The filtrate was concentrated *in vacuo*, affording crude **alcohol S10**, which was carried forward without further purification.

To a 2-dram vial equiped with stir bar was added crude **alcohol S10** (0.139 mmol), CH_2Cl_2 (1.4 mL) which had been previously shaken with distilled H_2O, and Dess-Martin periodinane (83.9 mg, 0.198 mmol). The reaction mixture was allowed to stir at room temperature for 1.5 hours. Upon consumption of starting material as indicated by TLC analysis, the reaction mixture was diluted with pentane (1.4 mL), dried over $MgSO_4$, filtered, and concentrated *in vacuo*. The crude product was purified by column chromatography (SiO_2, 3% Et_2O in hexanes) affording **aldehyde 34** (27.4 mg, 85%) as a colorless oil.

aldehyde 34

^{1}H NMR (500 MHz, $CDCl_3$) δ 9.95 (t, 1H, *J* = 3.3 Hz), 5.32 (s, 1H), 2.67 (dd, 1H, *J* = 15.2, 3.2 Hz), 2.50 (dd, 1H, *J* = 15.2, 3.2 Hz), 2.12-1.97 (m, 4H), 1.97-1.79 (m, 2H), 1.70 (s, 3H), 1.69-1.62 (m, 1H), 1.58-1.46 (m, 2H), 1.39 (td, 1H, *J* = 12.2, 7.1 Hz), 1.27-1.18 (m, 1H), 1.16 (d, 12.7 Hz), 1.09 (td, 1H, *J* = 13.2, 4.0 Hz), 1.01 (td, 1H, *J* = 12.2, 4.0 Hz), 0.83 (s, 3H) ppm; **^{13}C NMR** (126 MHz, $CDCl_3$) δ 204.7, 139.7, 123.8, 60.7, 469., 42.5, 41.8, 40.9, 38.1, 36.0, 34.4, 33.8, 28.8, 22.9, 22.6, 19.0 ppm; **IR** (thin film) ν_{max} 3007, 2930, 2865, 2831, 2736, 1716 cm^{-1}; **HRMS** (ESI+) calculated for $C_{16}H_{24}ONa$ $[M+Na]^+$ 255.1725 found 255.1728.

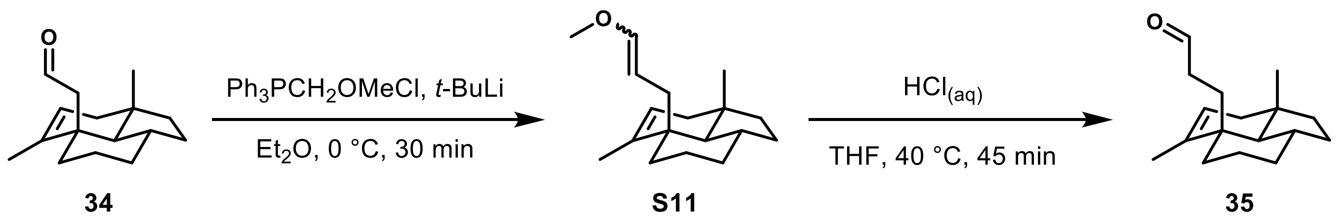

To a 1-dram vial equipped with a stir bar was added Ph_3PCH_2OMeCl (20.1 mg, 58.7 µmol) and Et_2O (0.17 mL). The mixture was cooled to 0 °C *via* ice bath. *t*-BuLi (31.4 µL, 53.4 µmol, 1.7 M in pentane) was added dropwise *via* syringe at 0 °C to the reaction mixture. A color change to red was observed. The mixture was allowed to warm to room temperature and allowed to stir at room temperature for 15 minutes. The reaction mixture was then cooled to 0 °C. **Aldehyde 34** (6.2 mg, 26.7 µmol) was added dropwise *via* syringe at 0 °C to the reaction vessel. The reaction mixture was allowed to stir at 0 °C for 30 minutes. Upon consumption of starting material as indicated by TLC analysis, the reaction was quenched at 0 °C by the addition of saturated $NH_4Cl_{(aq)}$ (1 mL) then allowed to warm to room temperature. The resultant slurry was extracted with Et_2O (3 x 2 mL). The organic extracts were dried over $MgSO_4$, filtered, and concentrated *in vacuo*, affording crude **methyl alkenyl ether S11** (1:1 E:Z) as a yellow oil. The crude product was carried forward without further purification.

To a 1-dram vial equipped with a stir bar was added crude **methyl alkenyl ether S11**, THF (0.2 mL), and 10% (w/v%) $HCl_{(aq)}$ (0.2 mL). The mixture was heated to 40 °C *via* oil bath and allowed to stir at 40 °C for 45 minutes. Upon consumption of starting material as indicated by TLC analysis, the reaction vessel was cooled to 0 °C *via* ice bath. The reaction was quenched at 0 °C by the addition of saturated $NaHCO_{3(aq)}$ (1 mL), then allowed to warm to room temperature. The resultant slurry was extracted with Et_2O (3 x 2 mL). The

organic extracts were dried over $MgSO_4$, filtered, and concentrated *in vacuo*. The crude product was purified by column chromatography (SiO_2, 2% Et_2O in hexanes) affording **aldehyde 35** (5.2 mg, 72%) as a colorless oil.

aldehyde 35

^{1}H NMR (500 MHz, $CDCl_3$) δ 9.76 (t, 1H, *J* = 1.5 Hz), 5.24 (s, 1H), 2.60 (dddd, 1H, *J* = 17.0, 12.2, 5.0, 1.7 Hz), 2.52 (dddd, 1H, *J* = 17.0, 12.2, 5.0, 1.7 Hz), 2.10-1.95 (m, 4H), 1.95-1.83 (m, 3H), 1.79 (ddd, 1H, *J* = 16.6, 11.8, 5.0 Hz), 1.70 (s, 3H, coupling constants too small to determine), 1.63 (dquint, 1H, *J* = 14.1, 3.5 Hz), 1.56-1.50 (m, 1H), 1.41-1.29 (m, 2H), 1.26-1.13 (m, 1H), 1.17 (d, 1H, *J* = 12.5 Hz), 1.03-0.91 (m, 2H), 0.90 (s, 3H) ppm; **^{13}C NMR** (126 MHz, $CDCl_3$) δ 203.0, 141.4, 123.1, 60.9, 42.7, 42.2, 41.2, 39.6, 38.2, 34.48, 34.46, 33.9, 28.8, 24.3, 23.6, 23.0, 19.9 ppm; **IR** (thin film) 2926, 2864, 2804, 2713, 1758 cm^{-1}; **HRMS** (ESI+) calculated for $C_{17}H_{26}ONa$ $[M+Na]^+$ 269.1881 found 269.1888.

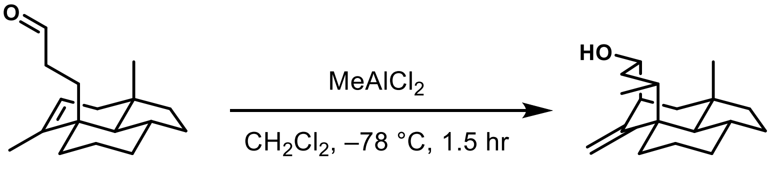

35 **13**

To a 25 mL round bottom flask equipped with a stir bar was added **aldehyde 35** (6.4 mg, 0.026 mmol) and CH_2Cl_2 (5.5 mL). The vessel was cooled to –78 °C *via* $Me_2CO/CO_{2(s)}$ bath, and $MeAlCl_2$ (1 M in hexane) (40 µL, 0.040 mmol) was added dropwise. The reaction mixture was allowed to stir at –78 °C for 1.5 hours. Upon consumption of starting material as indicated by TLC analysis, the reaction was quenched at 78 °C by the dropwise addition of citric acid (2.5 mL), then allowed to warm to room temperature. The phases were partitioned, and the aqueous layer was extracted with DCM (2 x 5 mL). The organic extracts were dried over Mg_2SO_4, filtered, and concentrated *in vacuo*. The crude material was purified by column chromatography (SiO_2, 7.5% to 15% to 30% Et_2O in hexanes, stepped gradient) affording an inseparable 3:1 mixture of hydrocarbon alcohols (3.4 mg). The spectral data for the minor component of this mixture are consistent with those expected for the desired Prins cyclization product **13**.

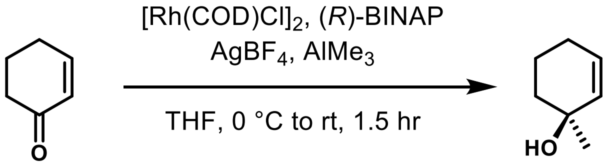

14 **40**

To a 3-necked 2 L round bottom flask equipped with a stir bar and internal thermometer was added $[Rh(COD)Cl]_2$ (1.63 g, 3.30 mmol), (*R*)-BINAP (4.93 g, 7.92 mmol), and THF (700 mL). The mixture was allowed to stir at room temperature for 1 hour, then a solution of $AgBF_4$ (2.18 g, 11.2 mmol) in THF (10 mL) was added, rinsing with THF (2 x 1 mL). A color change from red to orange was observed. Neat **cyclohexenone 14** (48.1 g, 500 mmol) was then added to the reaction vessel. The mixture was cooled to 5 °C (internal temperature) *via* ice bath, and neat $AlMe_3$ (57.5 mL, 600 mmol) was added at a rate sufficient to maintain the internal temperature below 10 °C (*ca.* 45 minutes). A color change from orange to brown was observed. Following completion of the addition, the mixture was allowed to stir at 0 °C for 30 minutes, then allowed to warm to room temperature. Upon consumption of starting material as indicated by TLC analysis (*ca.* 1 hour), the reaction mixture was decanted into a 4 L beaker equipped with stir bar containing pentane (1 L) (pre-cooled to 0 °C *via* ice bath). After vigorous stirring at 0 °C for 10 minutes, the mixture was allowed to warm to room temperature, and activated charcoal (120 g) followed by $Na_2SO_{4(s)}$ (16 g) were added. The suspension was allowed to stir vigorously for 5 minutes, then filtered through a plug of Celite®, rinsing with pentane (3 x 200 mL). The filtrate was concentrated *in vacuo* to afford crude **alcohol 40** (46.7 g, 83%) as a colorless liquid. Crude **alcohol 40** was carried forward without further purification. The characterization data obtained are in good agreement with those previously reported.[7]

alcohol 40

^{1}H NMR (500 MHz, $CDCl_3$) δ 5.62 (dt, *J* = 10.0, 3.7 Hz, 1H), 5.52 (appar. d, *J* = 12 Hz, 1H) 2.19 (bs, 1H), 1.97-1.88 (m, 1H), 1.82 (dddd, J = 17.3, 7.8, 5.5, 2.6 Hz, 1H), 1.69-1.58 (m, 2H), 1.56-1.48 (m, 2H), 1.18 (s, 3H) ppm; **^{13}C NMR** (125 MHz, $CDCl_3$) δ 133.89, 128.47, 67.76, 37.75, 29.26, 24.95, 19.43 ppm; $[\alpha]_D^{22.4}$ = –69.8 (*c* 1.00, $CHCl_3$); **FTIR** (film) ν_{max} 3341 (br), 3019, 2933, 2872 cm^{-1}; **SFC** analysis (Chiracel AD, 0.1% *i*-PrOH, 3 mL/min, 210 nm) indicated >99% ee: t_R (desired enantiomer) = 4.0 minutes, t_R (undesired enantiomer) = 4.6 minutes.

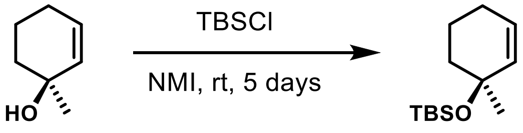

To a 500 mL round bottom flask equipped with a stir bar was added TBSCl (62.7 g, 416 mmol). The vessel was evacuated and backfilled with argon three times. *N*-Methylimidazole (60 mL, 820 mmol) was added, followed by neat crude **alcohol 40** (46.7 g, 416 mmol), rinsing with additional *N*-methylimidazole (3 x 5 mL). The reaction mixture was allowed to stir vigorously at room temperature for 5 days. Upon consumption of starting material as indicated by TLC analysis, the reaction mixture was transferred to a separatory funnel and the phases were partitioned. The organic phase was washed with water (4 x 25 mL), dried over $MgSO_4$, filtered, and concentrated *in vacuo* to afford crude **silyl ether S12** (78.3 g, 83%) as a colorless liquid. The crude product was carried forward without further purification.

silyl ether S12

^{1}H NMR (600 MHz, $CDCl_3$) δ 5.66 (dt, *J* = 10.0, 3.6 Hz, 1H), 5.61 (appar. d, *J* = 10.1 Hz, 1H), 2.04-1.96 (m, 1H), 1.90 (dddd, *J* = 18.7, 8.2, 5.3, 2.8 Hz, 1H), 1.81-1.72 (m, 2H), 1.60-1.48 (m, 2H), 1.25 (s, 3H), 0.85 (s, 9H), 0.07 (s, 3H), 0.05 (s, 3H) ppm; **^{13}C NMR** (125 MHz, $CDCl_3$) δ 134.8, 127.9, 70.4, 39.0, 30.9, 26.0, 25.3, 19.7, –1.9, –2.1 ppm; $[\alpha]_D^{22.4}$ = –84.4 (*c* 1.00, $CHCl_3$); **FTIR** (film) ν_{max} 3021, 2930, 2857 cm^{-1}; **HRMS** (CI) calculated for $C_{13}H_{26}OSi$ $[M–H_2+H]^+$ 225.1675 found 225.1664.

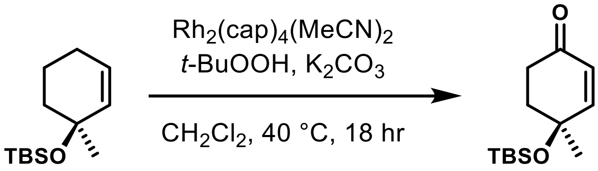

To a 1 L round bottom flask equipped with a stir bar was added CH_2Cl_2 (280 mL) and **silyl ether S12** (17.2 g, 75.8 mmol). The mixture was stirred until dissolved, followed by the addition of ignited K_2CO_3 (5.24 g, 37.9 mmol) and freshly prepared $Rh_2(cap)_4(MeCN)_2$[8] (558 mg, 0.758 mmol). The reaction mixture was heated to 40 °C *via* oil bath, and TBHP (69 mL, 5.5 M in nonane) was added over 30 seconds. Gas evolution was observed. After 1.5 hours, the reaction mixture was charged with additional $Rh_2(cap)_4$ (279 mg, 0.379 mmol) and TBHP (69 mL, 5.5 M in nonane) over 30 seconds. At 3 hours, the addition of $Rh_2(cap)_4$ (279 mg, 0.379 mmol) and TBHP (69 mL, 5.5 M in nonane) was repeated. Upon consumption of starting material as indicated by TLC analysis (*ca*. 18 hours), the reaction mixture was allowed to cool to room temperature and concentrated *in vacuo* leaving a crude slurry in nonane, which was then partitioned between Et_2O (400 mL) and water (200 mL). The organic extracts were washed with H_2O (2 x 200 mL), and brine (100 mL), dried over Na_2SO_4, filtered, and concentrated *in vacuo* yielding a solution of crude product in nonane. The crude solution was then purified by column chromatography (SiO_2, 0% to 6% Et_2O in pentane, stepped gradient) to afford **enone 41** (13.38 g, 76%) as a colorless oil.

enone 41

^{1}H NMR (600 MHz, $CDCl_3$) δ 6.74 (d, *J* = 10.1 Hz, 1H), 5.83 (d, *J* = 10.1 Hz, 1H), 2.63 (ddd, *J* = 17.1, 7.7, 4.9 Hz, 1H), 2.37 (ddd, *J* = 17.1, 9.1, 4.9 Hz, 1H), 2.16 (ddd, *J* = 13.4, 9.1, 4.9 Hz, 1H), 2.03 (ddd, *J* = 13.4, 7.7, 4.9 Hz, 1H), 1.43 (s, 3H), 0.86 (s, 9H), 0.11 (s, 3H), 0.09 (s,

3H) ppm; **^{13}C NMR** (150 MHz, $CDCl_3$) δ 199.5, 155.6, 127.5, 70.6, 38.3, 35.1, 28.4, 25.8, 18.1, –1.9, –2.0 ppm; $[\alpha]_D^{21.6}$ = –82.8 (*c* 2.28, CH_2Cl_2); **FTIR** (film) ν_{max} 2955, 2929, 2857, 1686 cm^{-1}; **HRMS** (CI) calculated for $C_{13}H_{24}O_2Si$ $[M+H]^+$ 241.1624 found 241.1615.

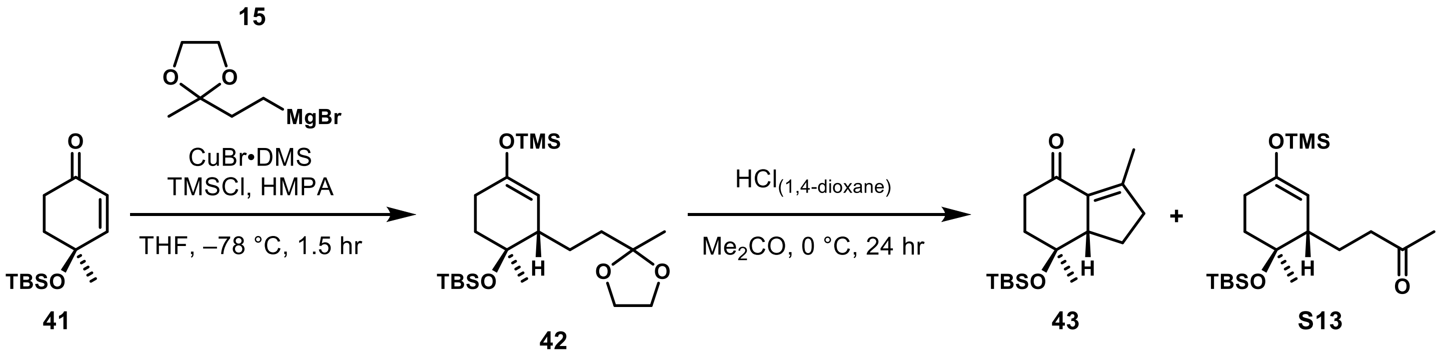

To a 3-necked 500 mL round bottom flask equipped with a stir bar and internal thermometer was added magnesium powder (20-230 mesh, 6.95 g, 286 mmol). The flask was then flame dried under high vacuum and allowed to cool to room temperature under an atmosphere of argon. THF (150 mL) and dibromoethane (220 µL) were added. The resultant slurry was sonicated for one minute, then stirred vigorously until a gray precipitate was observed (ca. 30 min). The reaction mixture was cooled to 10 °C (internal temperature) *via* ice bath, and a solution of **bromoketal S4** (22.3 g, 114 mmol) in THF (80 mL) was added dropwise at a rate sufficient to maintain an internal temperature of 10–20 °C. The flask containing the bromide solution was then rinsed with THF (2 x 35 mL) and transferred to the reaction mixture, ensuring quantitative transfer. The reaction mixture was then stirred for 30 minutes at an internal temperature of 15 °C. Stirring was halted, and the precipitate was allowed to settle (ca. 15 min). The supernatant containing **Grignard reagent 15** was transferred *via* cannula into a flame dried 3-necked 500 mL round bottom flask equipped with stir bar, rinsing with THF (2 x 35 mL). The solution was then cooled to –60 °C (internal temperature) *via* $Me_2CO/CO_{2(s)}$ bath and freshly prepared CuBr·DMS[9] (11.8 g, 57.2 mmol) was added in one portion. The reaction mixture was warmed to –40 °C (internal temperature) *via* $MeCN/CO_{2(s)}$ bath and allowed to stir for 30 minutes, during which time a color change from gray to brown was observed. The reaction mixture was then cooled to –78 °C (internal temperature) *via* $Me_2CO/CO_{2(s)}$ bath. HMPA (19.9 mL, 114 mol) and TMSCl (14.5 mL, 114 mmol) were added dropwise to the reaction mixture. A solution of **enone 41** (13.8 g, 57.2 mmol) in THF (40 mL) was then added dropwise *via* cannula, rinsing with THF (2 x 25 mL). The resulting mixture was stirred at –78 °C (internal temperature) for an additional 1.5 hours, then quenched with 4:1 $NH_4Cl_{(aq)}$:1 M NaOH (350 mL) at –78 °C and allowed to warm to room temperature. The mixture was diluted with water until precipitated salts were dissolved, and extracted with Et_2O (3 x 600 mL). The organic extracts were washed with water (2 x 300 mL), 4:1 $NH_4Cl_{(aq)}$:1 M NaOH (300 mL), and brine (150 mL), dried over $MgSO_4$, filtered, and concentrated *in vacuo*. The crude **enoxysilane 42** was carried forward without further purification.

To a 1 L round bottom flask equipped with a stir bar was added crude **enoxysilane 42** and Me_2CO (380 mL). The mixture was stirred at room temperature until dissolved, then cooled to 0 °C *via* ice bath. Hydrochloric acid in 1,4-dioxane (38 mL, 4 M) was added and the resulting mixture was allowed to sit in a freezer (ca. –20 °C) for 24 hours. The reaction mixture was quenched with saturated $NaHCO_{3(aq)}$ (300 mL) at 0 °C, allowed to warm to room temperature, and extracted with hexanes (3 x 600 mL). The organic extracts were dried over $MgSO_4$, filtered, and concentrated *in vacuo*. The resultant crude product was purified by column chromatography (SiO_2, 3% Et_2O in hexanes). Unreacted **diketone S13** (2.38 g) was recovered by eluting with 30% EtOAc in hexanes and resubjected to the reaction conditions (76.3 mL Me_2CO, 7.63 mL 4 M HCl in 1,4-dioxane). **Hydrindenone 43** (12.1 g, 72% overall) was obtained as a yellow oil.

hydrindenone 43
^{1}H NMR (600 MHz, $CDCl_3$) δ 3.08 (t, *J* = 8.5 Hz, 1H), 2.48-2.37 (m, 2H), 2.34-2.25 (m, 2H), 2.07 (s, 3H), 2.02-1.88 (m, 3 H), 1.7 (dq, *J* = 12.9, 9.5 Hz, 1H), 1.16 (s, 3H), 0.86 (s, 9H), 0.09 (s, 3H), 0.08 (s, 3H) ppm; **^{13}C NMR** (150 MHz, $CDCl_3$) δ 199.3, 155.8, 133.5, 74.7, 58.1, 39.7, 38.8, 38.7, 25.9, 24.4, 21.7, 18.2, 16.4, –1.80, –1.83 ppm; $[\alpha]_D^{22.4}$ = –3.7 (*c* 1.22, CH_2Cl_2); **FTIR** (film) ν_{max} 2955, 2929, 2856, 1681, 1618 cm^{-1}; **HRMS** (CI) calculated for $C_{17}H_{30}O_2Si$ $[M+H]^+$ 295.2093 found 295.2094.

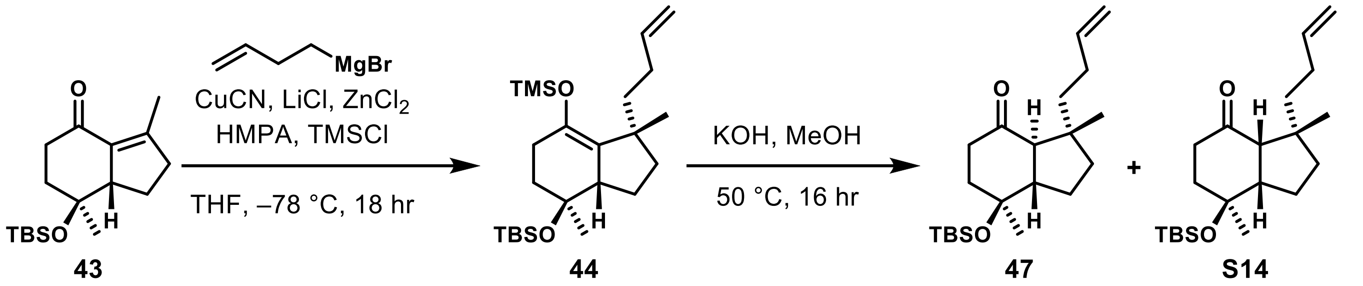

To a 1 L round bottom flask equipped with a stir bar was added magnesium powder (20-230 mesh, 25.5 g, 1.05 mol). The flask was then flame dried under high vacuum and allowed to cool to room temperature under an atmosphere of argon. THF (50 mL) and dibromoethane (150 µL) were added. The resultant slurry was sonicated for one minute, then stirred vigorously until a gray precipitate was observed (ca. 30 min). Neat 4-bromobutene (35.5 mL, 0.35 mol) was added slowly until an exotherm was observed indicating formation of the desired Grignard reagent. THF (300 mL) was then added, followed by dropwise addition of the remaining 4-bromobutene. The flask was then submerged in a 10–15 °C water/ice bath to cool the exotherm. Upon completion of the addition, the slurry was stirred for a further 1 hour at room temperature. Stirring was halted, and the precipitate was allowed to settle (ca. 15 min). The resulting solution of 4-butenylmagnesium bromide was titrated, and a concentration of 1.0 M was observed. To a separate 1 L round bottom flask was added previously refused $ZnCl_2$ (15.4 g, 113 mmol). The flask was then flame dried under high vacuum until melting of the $ZnCl_2$ was observed then allowed to cool to room temperature under an atmosphere of argon. THF (320 mL) was added, and the mixture was sonicated at room temperature until dissolved. The resulting solution was cooled to –78 °C *via* $Me_2CO/CO_{2(s)}$ bath, and freshly prepared 4-butenylmagnesium bromide (1.0 M in THF, 221 mL, 221 mmol) was added dropwise *via* cannula. The reaction mixture was allowed to warm to room temperature, then allowed to stir at room temperature for 1 hour. The reaction mixture was then cooled to –78 °C *via* $Me_2CO/CO_{2(s)}$ bath. CuCN (10.2 g, 114 mmol) and LiCl (9.82 g, 232 mmol) were added, and the resulting slurry was allowed to stir at –78 °C for 1 hour. HMPA (37.6 mL, 216 mmol) and TMSCl (20.1 mL, 158 mmol) were added dropwise, and the reaction mixture was then allowed to stir at –78 °C for 30 minutes. A solution of **enone 43** (15.5 g, 52.7 mmol) in THF (85 mL) was then added dropwise to the reaction mixture. The reaction mixture was allowed to stir at –78 °C for 18 hours. Upon consumption of starting material as indicated by TLC analysis, the reaction mixture was quenched at –78 °C with a 4:1 $NH_4Cl_{(aq)}$:1 M NaOH solution (500 mL), allowed to warm to room temperature, and extracted with hexanes (3 x 250 mL). The organic extracts were washed with 4:1 $NH_4Cl_{(aq)}$:1 M NaOH (250 mL), water (250 mL), and brine (150 mL), dried over $MgSO_4$, filtered, and concentrated *in vacuo*. Crude **enoxysilane 44** (93:7 *dr* by crude ^{1}H-NMR integration) was carried forward without further purification.

To a 1 L round bottom flask containing crude **enoxysilane 44** (23.0 g, 52.7 mmol) and equipped with a stir bar was added MeOH (298 mL) and KOH (1 M in MeOH, 132 mL). The reaction mixture was heated to 50 °C *via* oil bath and allowed to stir for 16 hours. The reaction mixture was then cooled to room temperature and concentrated *in vacuo*, yielding a crude yellow residue. The residue was partitioned between Et_2O (500 mL) and sat. aqueous NH_4Cl (500 mL). The aqueous layer was then extracted with Et_2O (2 x 500 mL). The organic extracts were dried over $MgSO_4$, filtered, and concentrated *in vacuo*. The crude hydrindanone (1:3 *dr* by ^{1}H NMR integration of the unpurified sample) was purified by column chromatography (SiO_2, 1% Et_2O in hexanes) to afford ***cis*-hydrindanone S14** and ***trans*-hydrindanone 47**. The ***cis*-hydrindanone S14** was resubject to the above conditions 9 times, affording ***trans*-hydrindanone 47** (14.5 g, 79% overall) as a colorless oil.

***trans*-hydrindanone 47**

^{1}H NMR (500 MHz, $CDCl_3$) δ 5.79 (ddt, *J* = 17.0, 10.2, 6.4 Hz, 1H), 4.97 (d, *J* = 17.0 Hz, 1H), 4.89 (d, *J* = 10.2 Hz, 1H), 2.36-2.17 (m, 3H), 2.14-2.03 (m, 1H), 2.02-1.82 (m, 4H), 1.82-1.64 (m, 2H), 1.53-1.42 (m, 2H), 1.41-1.29 (m, 2H), 1.35 (s, 3H), 1.06 (s, 3H), 0.85 (s, 9H), 0.09 (s, 3H), 0.07 (s, 3H) ppm; **^{13}C DEPTQ** (125 MHz, $CDCl_3$) δ 209.8, 139.6, 114.0, 74.9, 59.7, 53.0, 42.5, 41.8, 41.6, 40.5, 37.3, 29.8, 25.9, 21.5, 20.1, –1.81, –1,84 ppm; $[\alpha]_D^{22.4}$ = +22.4 (*c* 1.99, CH_2Cl_2); **FTIR** (film) ν_{max} 3076, 2955, 2930, 2856, 1716, 1641 cm^{-1}; **HRMS** (CI) calculated for $C_{21}H_{38}O_2Si$ [M–(*t*-Bu)+H]$^+$ 293.1937 found 293.1927.

***cis*-hydrindanone S14**

^{1}H NMR (500 MHz, $CDCl_3$) δ 5.75 (ddt, *J* = 17.0, 10.3, 6.5 Hz, 1H), 4.96 (dq, *J* = 17.0, 1.6 Hz, 1H), 4.88 (d, *J* = 10.3, 1H), 2.57-2.43 (m, 3H), 2.12-1.99 (m, 3H), 1.98-1.89 (m, 1H), 1.88-1.78 (m, 1H), 1.78-1.66 (m, 2H), 1.49-1.32 (m, 3H), 1.30-1.18 (m, 1H), 1.26 (s, 3H), 1.07 (s, 3H), 0.81 (s, 9H), 0.07 (s, 6H); **^{13}C DEPTQ** (125 MHz, $CDCl_3$) δ 214.0, 139.4, 114.2, 114.0, 74.4, 60.7, 53.1, 47.1, 37.7, 36.9, 36.2, 32.9, 27.9, 27.0, 25.9, 18.4, –1.9, –2.1 ppm; $[\alpha]_D^{22.5}$ = –15.4 (*c* 2.02, CH_2Cl_2); **FTIR** (film) ν_{max} 3075, 2953, 2929, 2856, 1694, 1641 cm^{-1}; **HRMS** (CI) calculated for $C_{21}H_{38}O_2Si$ $[M+H]^+$ 351.2719 found 351.2734.

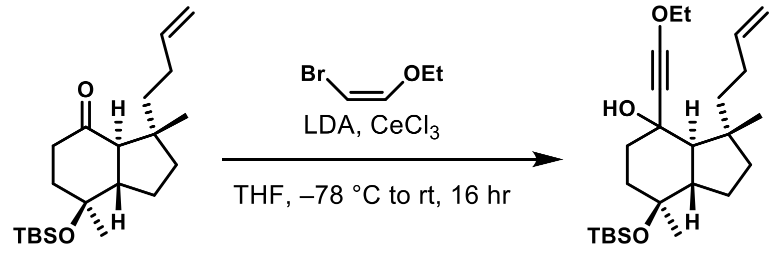

47 **48**

To a 3-necked 1 L round bottom flask equipped with a stir bar was added added THF (220 mL) and *i*-Pr_2NH (22.6 mL, 161 mmol). The solution was cooled to –78 °C *via* $Me_2CO/CO_{2(s)}$ bath, and *n*-BuLi (2.75 M, 57.6 mL, 158 mmol) was added dropwise *via* addition funnel. The mixture was allowed to warm to 0 °C in an ice bath, then allowed to stir for 30 minutes. The mixture was then cooled to –78 °C *via* $Me_2CO/CO_{2(s)}$ bath, and 1-bromo-2-ethoxyethene (8.59 mL, 80.7 mmol) was added dropwise *via* addition funnel. The mixture was allowed to warm to 0 °C in an ice bath, then allowed to stir for 2.5 hours. The mixture was then cooled to –78 °C *via* $Me_2CO/CO_{2(s)}$ bath, and anhydrous $CeCl_3$ (21.5 g, 87.1 mmol) was added in one portion. The resulting slurry was allowed to stir at –78 °C for 30 minutes. **Hydrindanone 47** (11.1 g, 31.7 mmol) was then added as a solution in THF (20 mL) at –78 °C, rinsing with THF (10 mL). The reaction mixture was allowed to warm to room temperature, then allowed to stir for 16 hours. Upon consumption of starting material as indicated by TLC analysis, the reaction mixture was quenched at –78 °C with solid NH_4Cl. The resulting slurry was allowed to warm to room temperature, stirring vigorously for 15 minutes. The mixture was then filtered through a pad of silica, rinsing with Et_2O, and the filtrate was concentrated *in vacuo*. The crude material was purified by column chromatography (SiO_2, 5% Et_2O in hexanes) to afford **alkynyl ether 48** (10.8 g, 84%) as a colorless oil (inconsequential mixture of diastereomers).

Note: individual diastereomers were isolated for characterization purposes only. Otherwise, the diastereomeric mixture was isolated together and taken onto the next step.

less polar diastereomer of alkynyl ether 48

^{1}H NMR (500 MHz, $CDCl_3$) δ 5.83 (ddt, *J* = 17.0, 10.3, 6.5 Hz, 1H), 5.00 (dq, *J* = 17.0, 1.5 Hz, 1H), 4.91 (dquin, *J* = 10.3, 1.0 Hz, 1H), 4.06 (q, *J* = 7.0 Hz, 2H), 2.19 (ddd, *J* = 13.2, 10.3, 7.4 Hz, 1H), 2.14-2.00 (m, 2H), 1.19-1.80 (m, 3H), 1.70-1.47 (m, 6H), 1.35 (t, *J* = 7.0 Hz, 3H), 1.28-1.20 (m, 3H), 1.17 (s, 3H), 1.14 (s, 3H), 0.84 (s, 9H), 0.07 (s, 3H), 0.06 (s, 3H) ppm; **^{13}C NMR** (125 MHz, $CDCl_3$) δ 140.0, 113.8, 93.4, 75.9, 74.3, 67.6, 56.4, 48.5, 43.6, 42.9, 42.5, 42.4, 38.1, 37.8, 29.7, 26.0, 24.5, 22.9, 20.3, 18.3, 14.7, –1.71, –1.75 ppm; $[\alpha]_D^{22.7}$ = +32.2 (*c* 1.28, CH_2Cl_2); **FTIR** (film) ν_{max} 3505 (br), 2954, 2929, 2856, 2260, 1640 cm^{-1} **HRMS** (ESI) calculated for $C_{25}H_{44}O_3Si$ $[M+Na]^+$ 443.2957 found 443.2944.

more polar diastereomer of alkynyl ether 48

^{1}H NMR (500 MHz, $CDCl_3$) δ 5.82 (ddt, *J* = 17.1, 10.3, 6.5 Hz, 1H), 4.99 (dq, *J* = 17.1, 1.7 Hz, 1H), 4.89 (dquin, *J* = 10.3, 1.0 Hz, 1H), 4.09 (q, *J* = 7.1 Hz, 2H), 2.17-1.97 (m, 3H), 1.88 (td, *J* = 13.5, 3.8 Hz, 1H), 1.78 (s, 1H), 1.77-1.59 (m, 5H), 1.49 (ddd, *J* = 12.4, 9.5, 5.0 Hz, 1H), 1.43-1.24 (m, 5H), 1.39 (t, *J* = 7.1 Hz, 3H), 1.21 (s, 3H), 1.17 (s, 3H), 0.85 (s, 9H), 0.08 (s, 3H), 0.07 (s, 3H) ppm; **^{13}C NMR** (125 MHz, $CDCl_3$) δ 140.1, 113.8, 96.5, 75.9, 74.3, 72.9, 57.9, 51.5, 44.5, 43.81, 43.76, 41.0, 40.7, 38.6, 31.7, 25.9, 23.1, 22.9, 22.8, 21.1, 18.2, 14.7, 14.3, –1.7, –1.8 ppm; $[\alpha]_D^{22.4}$ = –11.2 (*c* 2.41, CH_2Cl_2); **FTIR** (film) ν_{max} 3451 (br), 2952, 2928, 2856, 2257, 1640 cm^{-1}; **HRMS** (ESI) calculated for $C_{25}H_{44}O_3Si$ $[M+Na]^+$ 443.2957 found 443.2953.

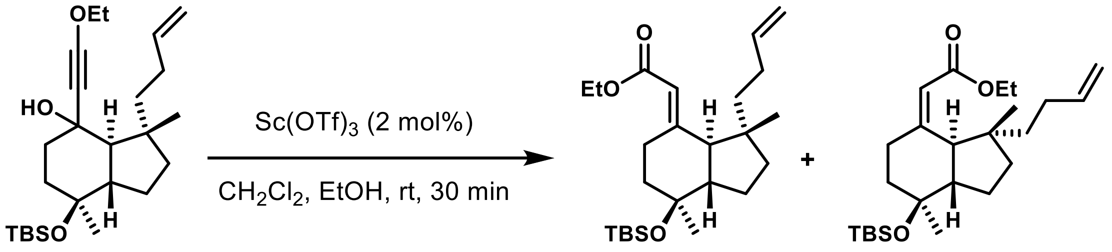

To a 500 mL round bottom flask equipped with a stir bar was added **alkynyl ether 48** (12.0 g, 29.4 mmol), and a 30:1 mixture of CH_2Cl_2/EtOH (200 mL). The mixture was allowed to stir at room temperature until dissolved, then cooled to 0 °C *via* ice bath. $Sc(OTf)_3$ (290 mg, 0.589 mmol) was added, and the reaction mixture was allowed to warm to room temperature and stir for 30 minutes. Upon consumption of starting material as indicated by TLC analysis, the reaction mixture was diluted with hexanes (300 mL) and filtered through a plug of silica, rinsing with Et_2O. The filtrate was then concentrated *in vacuo*. The crude product was purified by column chromatography (SiO_2, 2% to 5% Et_2O in hexanes, stepped gradient) affording a mixture of ***E*-enoate ester 49** and ***Z*-enoate ester S15** (10.2 g, 85%) as a colorless oil (inconsequential 2:1 mixture of diastereomers).

Note: individual diastereomers were isolated for characterization purposes only. Otherwise, the diastereomeric mixture was isolated together and taken onto the next step.

***Z*-enoate ester S15**
^{1}H NMR (600 MHz, $CDCl_3$) δ 5.81 (ddt, *J* = 16.8, 10.3, 6.5 Hz, 1H), 5.77 (bs, 1H), 4.99 (d, *J* = 17.1 Hz, 1H), 4.89 (d, *J* = 10.2 Hz, 1H), 4.15 (dq, *J* = 10.9, 7.3 Hz, 1H), 4.05 (dq, *J* = 10.9, 7.3 Hz, 1H), 2.94 (d, *J* = 13.4 Hz, 1H), 2.71-2.61 (m, 1H), 2.42 (td, *J* = 12.8, 5.7 Hz, 1H), 2.11-1.96 (m, 4H), 1.76 (appar. t, *J* = 11.6 Hz, 1H), 1.69-1.59 (m, 2H), 1.50-1.37 (m, 3H), 1.32-1.23 (m, 1H), 1.26 (t, *J* = 7.2 Hz, 3H) 1.12 (s, 3H), 0.89 (s, 3H), 0.86 (s, 9H), 0.08 (s, 3H), 0.06 (s, 3H) ppm; **^{13}C NMR** (125 MHz, $CDCl_3$) δ 167.3, 159.7, 140.0, 116.6, 113.8, 74.4, 59.8, 51.1, 51.0, 45.8, 43.8, 42.6, 30.7, 35.5, 29.8, 26.0, 24.8, 23.2, 23.0, 18.2, 14.4, –1.8, –1.9 ppm; $[\alpha]_D^{22.4}$ = +74.9 (*c* 1.00, $CHCl_3$); **FTIR** (film) ν_{max} 2956, 2930, 2856, 1720, 1641 cm^{-1}; **HRMS** (CI) calculated for $C_{25}H_{44}O_3Si$ $[M]^+$ 420.3060 found 420.3047.

***E*-enoate ester 49**
^{1}H NMR (600 MHz, $CDCl_3$) δ 5.86-5.78 (m, 1H), 5.69 (bs, 1H), 5.01 (d, *J* = 17.1 Hz, 1H), 4.93 (d, *J* = 10.0 Hz, 1H), 4.15 (q, *J* = 7.3 Hz, 2H), 3.90-3.84 (m, 1H), 2.16-2.06 (m, 2H), 2.00 (tt, *J* = 12.6, 7.2 Hz, 1H), 1.87-1.75 (m, 4H), 1.72-1.56 (m, 2H), 1.54-1.48 (m, 1H), 1.45-1.39 (m, 2H), 1.36 (td, *J* = 12.6, 4.8 Hz, 1H), 1.29 (t, *J* = 7.1 Hz, 2H), 1.25 (s, 3H), 1.06 (s, 3H), 0.83 (s, 9H), 0.07 (s, 3H) 0.05 (s, 3H) ppm; **^{13}C NMR** (125 MHz, $CDCl_3$) δ 167.2, 160.9, 139.6, 114.2, 112.6, 75.5, 59.9, 55.5, 53.4, 43.5, 42.9, 40.6, 39.3, 29.9, 29.5, 28.1, 25.9, 21.2, 20.3, 20.1, 18.2, 14.5, –1.78, –1.80 ppm; $[\alpha]_D^{22.4}$ = –30.2 (*c* 1.00, $CHCl_3$); **FTIR** (film) ν_{max} 2955, 2928, 2855, 1717, 1644 cm^{-1}; **HRMS** (CI) calculated for $C_{25}H_{44}O_3Si$ $[M]^+$ 420.3060 found 420.3062.

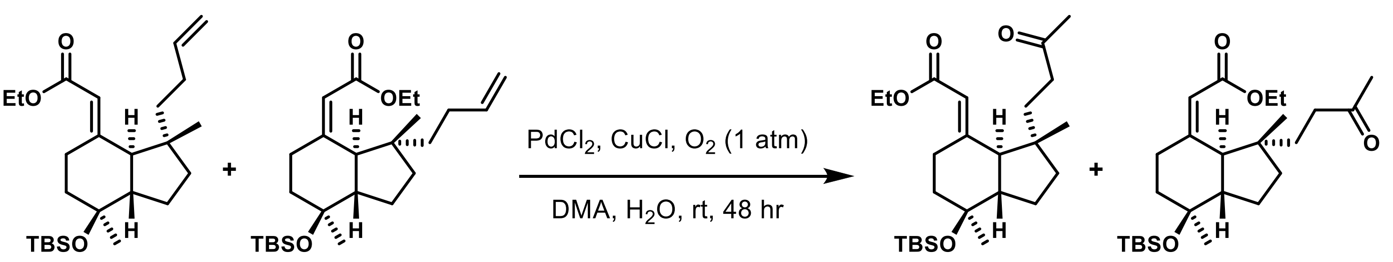

To a 250 mL round bottom flask equipped with a stir bar was added a mixture of ***E*-enoate ester 49** and ***Z*-enoate ester S15** (3.23 g, 7.68 mmol), DMA (70 mL), and water (7 mL). The mixture was allowed to stir at room temperature until dissolved, then freshly purified CuCl (3.19 g, 32.2 mmol) and $PdCl_2$ (1.02 g, 5.76 mmol) were added. The reaction mixture was sparged with O_2 for 15 min, then allowed

to stir under an atmosphere of O_2 for 48 hours. The reaction mixture was then filtered through Celite®, rinsing with Et_2O (400 mL). The filtrate was washed with H_2O (5 x 100 mL) and brine (100 mL), dried over $MgSO_4$, filtered, and concentrated *in vacuo*. The crude product was purified by column chromatography (SiO_2, 10% Et_2O in hexanes) affording ***E*-ketoester 50** and ***Z*-ketoester S16** (2.88 g, 86%) as a yellow oil (inconsequential 2:1 mixture of diastereomers).

Note: individual diastereomers were isolated for characterization purposes only. Otherwise, the diastereomeric mixture was isolated together and taken onto the next step.

***Z*-ketoester S16**

^{1}H NMR (600 MHz, $CDCl_3$) δ 5.78 (bs, 1H), 4.18-4.03 (m, 2H), 2.93 (d, *J* = 13.4 Hz, 1H), 2.71-2.55 (m, 2H), 2.46-2.31 (m, 2H), 2.15 (s, 3H), 2.07 (dd, *J* = 13.7, 7.2 Hz, 1H), 2.00 (dd, *J* = 12.7, 5.5 Hz, 1H), 1.72-1.54 (m, 5H), 1.44 (dt, *J* = 13.2, 9.1 Hz, 1H), 1.31-1.20 (m, 1H), 1.26 (s, 3H), 1.12 (s, 3H), 0.89 (s, 3H), 0.86 (s, 9H), 0.08 (s, 3H), 0.06 (s, 3H) ppm; **^{13}C NMR** (125 MHz, $CDCl_3$) δ 209.9, 167.2, 159.7, 116.8, 74.2, 59.9, 51.3, 49.6, 45.0, 43.7, 40.0, 36.9, 36.4, 35.5, 30.0, 26.0, 24.8, 24.4, 23.4, 18.2, 14.3, –1.8, –1.9 ppm; $[\alpha]_D^{22.5}$ = +27.2 (*c* 1.23, CH_2Cl_2); **FTIR** (film) ν_{max} 2955, 2929, 2856, 1716, 1643 cm^{-1}; **HRMS** (ESI) calculated for $C_{25}H_{44}O_4Si$ $[M+Na]^+$ 459.2906 found 459.2919.

***E*-ketoester 50**

^{1}H NMR (600 MHz, $CDCl_3$) δ 5.67 (bs, 1H), 4.13 (q, *J* = 7.1 Hz, 2H), 3.91-3.84 (m, 1H), 2.50 (ddd, *J* = 16.6, 11.7, 4.8 Hz, 1H), 2.38 (ddd, *J* = 16.6, 11.6, 4.8 Hz, 1H), 2.16 (s, 3H), 2.13-2.06 (m, 1H), 2.01 (ddd, *J* = 14.0, 11.8, 4.8 Hz, 1H), 1.87-1.74 (m, 3H), 1.66 (dq, *J* = 13.8, 8.9 Hz, 1H), 1.59-1.47 (m, 3H), 1.45-1.30 (m, 2H), 1.27 (t, *J* = 7.1 Hz, 3H), 1.24 (s, 3H), 1.05 (s, 3H), 0.82 (s, 9H), 0.06 (s, 3H), 0.05 (s, 3H) ppm; **^{13}C NMR** (125 MHz, $CDCl_3$) δ 209.2, 167.0, 160.4, 112.7, 75.5, 59.9, 55.6, 53.5, 42.9, 42.8, 39.7, 39.2, 34.8, 30.1, 28.0, 25.9, 21.1, 20.3, 19.8, 18.2, 14.5, –1.79, –1.81 ppm; $[\alpha]_D^{22.7}$ = –91.5 (*c* 1.94, CH_2Cl_2); **FTIR** (film) ν_{max} 2955, 2929, 2856, 1714, 1645 cm^{-1}; **HRMS** (CI) calculated for $C_{25}H_{44}O_4Si$ $[M]^+$ 436.3009 found 436.2993.

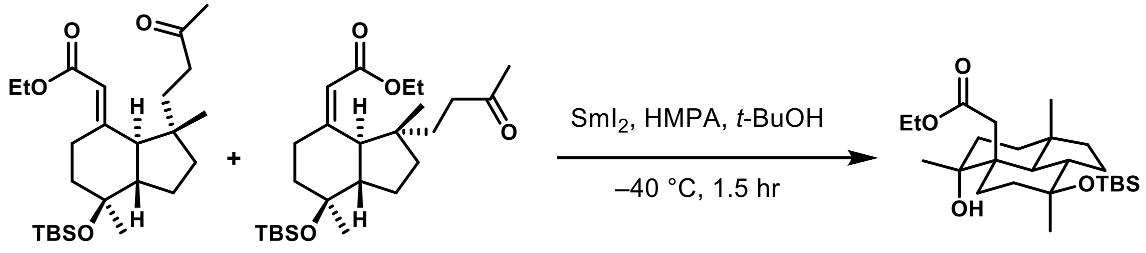

To a 500 mL round bottom flask equipped with a stir bar was added freshly synthesized SmI_2[6] (0.0975 M in THF, 106 mL, 10.3 mmol) and HMPA (28.8 mL, 165 mmol). The mixture was allowed to stir at room temperature for 30 minutes. THF (61 mL) was added, and the mixture was cooled to –40 °C *via* MeCN/$CO_{2(s)}$ bath. A solution of **ketoesters 50**, **S16** (1.80 g, 4.13 mmol) and *t*-BuOH (830 µL, 8.68 mmol) in THF (25 mL) was added dropwise, rinsing with THF (10 mL). The reaction mixture was allowed to stir at –40 °C for 1.5 hours, then quenched at –40 °C with a solution of 1:1 $NaHCO_{3(aq)}$: $Na_2S_2O_{3(aq)}$ (100 mL) and allowed to warm to room temeperature. The resulting mixture was extracted with Et_2O (3 x 75 mL). The organic extracts were dried over $MgSO_4$, filtered, and concentrated *in vacuo*. The resulting solution of crude product with HMPA was dissolved in Et_2O (200 mL), then washed with water (5 x 100 mL) and brine (75 mL). The organic extract was dried over $MgSO_4$, filtered, and concentrated *in vacuo*. The crude product was purified by column chromatography (SiO_2, 6% EtOAc in hexanes) affording **hydroxyester 51** (1.59 g, 88%) as a colorless oil.

hydroxyester 51

^{1}H NMR (600 MHz, $CDCl_3$) δ 4.14 (dq, *J* = 10.8, 7.3 Hz, 1H), 4.07 (dq, *J* = 10.8, 7.3 Hz, 1H), 2.79 (d, *J* = 13.5 Hz, 1H), 2.40 (d, *J* = 13.5 Hz, 1H), 2.20 (dt, *J* = 13.5, 3.6 Hz, 1H), 2.11-2.04 (m, 2H), 1.96 (dt, *J* = 17.6, 2.3 Hz, 1H), 1.87 (td, *J* = 13.5, 3.6 Hz, 1H), 1.78-1.71 (m, 1H), 1.69 (s, 3H), 1.65 (dt, *J* = 13.5, 3.6 Hz, 1H), 1.64-1.60 (m, 1H), 1.56 (ddd, *J* = 12.2, 8.8, 1.3 Hz, 1H), 1.28 (d, *J* = 14.0 Hz, 1H), 1.25 (t, 3H, *J* = 7.3 Hz), 1.21 (q, *J* = 11.3 Hz, 1H), 1.16 (s, 3H), 1.01 (td, *J* = 13.5, 3.6 Hz, 1H), 0.89 (s, 3H), 0.85 (s, 9H), 0.09 (s, 3H), 0.08 (s, 3H) ppm; **^{13}C NMR** (150 MHz, $CDCl_3$) δ 173.5, 141.1, 122.7, 77.1, 60.3, 56.6, 44.9, 43.0, 42.3, 40.7, 39.8, 38.4, 37.2, 33.3, 25.9, 22.3, 22.2, 21.3,

19.0, 18.2, 14.3, –1.7, –1.8 ppm; $[\alpha]_D^{22.4}$ = +14.1 (*c* 1.68, CH_2Cl_2); **FTIR** (film) ν_{max} 3536 (br), 2953, 2929, 2856, 1722 cm^{-1}; **HRMS** (CI) calculated for $C_{25}H_{46}O_4Si$ $[M+H]^+$ 439.3244 found 439.3266.

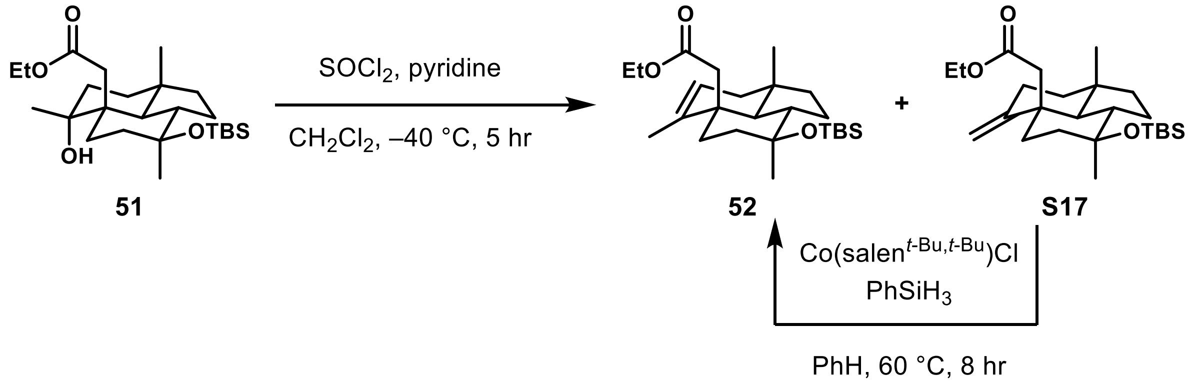

To a 250 mL round bottom flask equipped with a stir bar was added **hydroxyester 51** (1.37 g, 3.13 mmol), pyridine (31 mL), and CH_2Cl_2 (31 mL). The mixture was allowed to stir at room temperature until dissolved, then cooled to –40 °C *via* MeCN/$CO_{2(s)}$ bath. $SOCl_2$ (250 µL, 3.44 mmol) was added dropwise, and the reaction mixture was allowed to stir at –40 °C for 5 hours. The reaction mixture was the quenched at –40 °C with saturated $NaHCO_{3(aq)}$ (100 mL) and allowed to warm to room temperature. The organic layer was separated, and the aqueous phase was extracted with CH_2Cl_2 (4 x 200 mL). The organic extracts were dried over $MgSO_4$, filtered, and concentrated *in vacuo*. The crude product was purified by column chromatography (SiO_2, 0.5% to 2% Et_2O in hexanes, stepped gradient) affording **endocyclic alkene 52** (963 mg, 73%) and **exocyclic alkene S17** (124 mg, 9%) as colorless oils.

The exocyclic alkene isomer can be isomerized to the *endo* isomer with the following procedure:

To a 25 mL round bottom flask equipped with stir bar was added **exocyclic alkene S17** (327 mg, 0.775 mmol) and Co(salen$^{tBu,\ tBu}$)Cl (14.7 mg, 0.0231 mmol). The reaction vessel was evacuated and backfilled with argon three times. Degassed PhH (7.8 mL) was added, followed by $PhSiH_3$ (5.7 µL, 0.0465 mmol). The reaction mixture was heated to 60 °C *via* oil bath, and allowed to stir for 8 hours. The reaction mixture was then allowed to cool to room temperature, and concentrated *in vacuo*. The crude residue (5.4:1 *endocyclic*:*exocyclic*, based on ^{1}H NMR integration of the crude sample) was purified by column chromatography (SiO_2, 0.5% to 2% Et_2O in hexanes, stepped gradient) affording **endocyclic alkene 52** (241 mg, 74%) and **exocyclic alkene S17** (72.5 mg, 22%) as colorless oils.

endocyclic alkene 52

^{1}H NMR (600 MHz, $CDCl_3$) δ 5.25 (s, 1H), 4.15 (dq, *J* = 10.8, 7.3 Hz, 1H), 4.08 (dq, *J* = 10.8, 7.3 Hz, 1H), 2.80 (d, *J* = 13.6 Hz, 1H), 2.40 (d, *J* = 13.6 Hz, 1H), 2.20 (dt, *J* = 13.6, 3.4 Hz, 1H), 2.11-2.04 (m, 2H), 1.97 (dt, *J* = 17.7, 2.0 Hz, 1H), 1.87 (td, *J* = 13.6, 3.4 Hz, 1H), 1.74 (dq, *J* = 14.0, 9.4 Hz, 1H), 1.69 (s, 3H), 1.65 (dt, *J* = 13.6, 3.4 Hz, 1H), 1.64-1.60 (m, 1H), 1.57 (dd, *J* = 12.2, 8.8 Hz, 1H), 1.29 (d, *J* = 13.6 Hz, 1H), 1.26 (t, *J* = 7.3 Hz, 3H), 1.21 (q, *J* = 11.4 Hz, 1H), 1.16 (s, 3H), 1.02 (td, *J* = 13.6, 3.4 Hz, 1H), 0.89 (s, 3H), 0.86 (s, 9H), 0.09 (s, 3H), 0.08 (s, 3H) ppm; **^{13}C NMR** (150 MHz, $CDCl_3$) δ 173.5, 141.1, 122.8, 77.1, 60.3, 56.6, 44.9, 43.0, 42.3, 40.7, 39.9, 38.5, 37.2, 33.3, 26.0, 22.3, 22.2, 21.3, 19.0, 18.2, 14.3, –1.7, –1.8 ppm; $[\alpha]_D^{22.6}$ = +48.1 (*c* 1.19, CH_2Cl_2); **FTIR** (film) ν_{max} 2954, 2928, 2900, 2857 cm^{-1}; **HRMS** (CI) calculated for $C_{25}H_{44}O_3Si$ $[M+H]^+$ 421.3138 found 421.3124.

exocyclic alkene S17

^{1}H NMR (600 MHz, $CDCl_3$) δ 4.81 (s, 1H), 4.58 (s, 1H), 4.04 (q, *J* = 7.2 Hz, 2H), 2.61-2.51 (m, 3H), 2.17 (dt, *J* = 14.5, 2.5 Hz, 1H), 2.01 (dt, *J* = 14.5, 3.1 Hz, 1H), 1.98 (ddd, *J* = 14.5, 10.8, 6.3 Hz, 1H), 1.84-1.76 (m, 2H), 1.73 (dq, *J* = 14.5, 9.3 Hz, 1H), 1.67 (dt, *J* = 13.5, 3.1 Hz, 1H), 1.61-1.57 (m, 1H), 1.40 (dd, *J* = 11.7, 9.2, 1H), 1.29 (dd, *J* = 14.5, 3.1 Hz, 1H), 1.24 (dd, *J* = 14.5, 3.1 Hz, 1H), 1.20 (t, *J* = 7.2 Hz, 3H), 1.14 (s, 3H), 1.06 (q, *J* = 10.8 Hz, 1H), 1.00 (s, 3H), 0.94 (d, *J* = 9.3 Hz, 1H), 0.85 (s, 9H), 0.09 (s, 3H), 0.08 (s, 3H) ppm; **^{13}C NMR** (125 MHz, $CDCl_3$) δ 172.2, 154.4, 108.5, 76.8, 60.0, 58.1, 44.7, 42.6, 42.4, 41.1, 40.6, 39.4, 34.8, 32.9, 30.5, 26.0, 22.2, 21.1, 20.0, 18.2, 14.4, –1.69, –1.72 ppm; $[\alpha]_D^{22.5}$ = –16.4 (*c* 1.24, CH_2Cl_2); **FTIR** (film) ν_{max} 3081, 2955, 2929, 2855, 1734, 1640 cm^{-1}; **HRMS** (ESI) calculated for $C_{25}H_{44}O_3Si$ $[M+Na]^+$ 443.2957 found 443.2959.

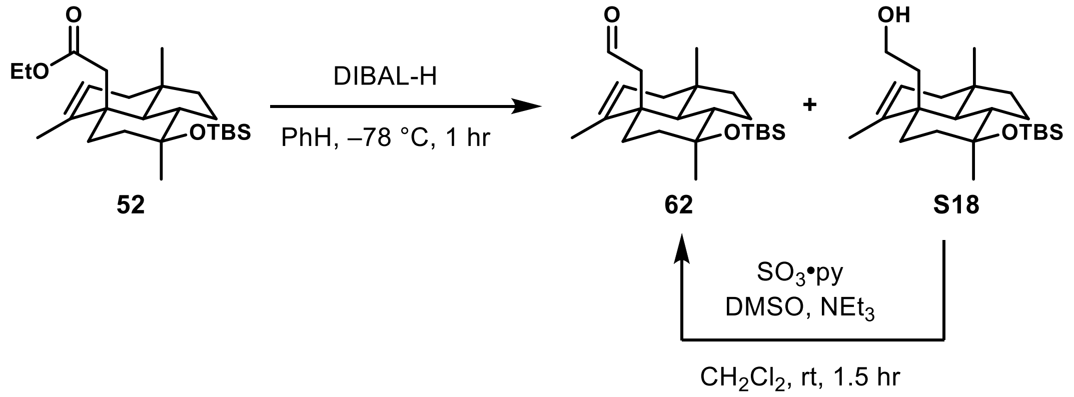

To a 100 mL round bottom flask equipped with a stir bar was added **endocyclic alkene 52** (1.04 g, 2.48 mmol) and PhMe (25 mL). The mixture was stirred at room temperature until dissolved, then cooled to –78 °C *via* $Me_2CO/CO_{2(s)}$ bath. Neat DIBAL-H (486 µL, 2.73 mmol) was added dropwise. The reaction mixture was allowed to stir at –78 °C for 1 hour, then quenched at –78 °C by dropwise addition of MeOH (200 µL). The quenched reaction mixture was allowed to warm to room temperature, then saturated Rochelle's salt (30 mL) and Et_2O (15 mL) were added. The resulting slurry was allowed to stir at room temperature for 1 hour. The organic layer was separated, and the aqueous phase was extracted with Et_2O (4 x 15 mL). The organic extracts were dried over $MgSO_4$, filtered, and concentrated *in vacuo*. The crude product was purified by column chromatography (SiO_2, 2% to 20% Et_2O in hexanes, stepped gradient) affording **aldehyde 62** (794 mg, 85%) and overreduced **alcohol S18** (113 mg, 12%) as colorless oils.

The alcohol can be oxidized to the desired aldehyde *via* Parikh–Doering oxidation:

To a 15 mL round bottom flask equipped with a stir bar was added **alcohol S18** (140 mg, 0.370 mmol), NEt_3 (260 µL, 1.85 mmol), DMSO (930 µL) and CH_2Cl_2 (3.70 mL). The mixture was allowed to stir until dissolved at room temperature, then cooled to 0 °C *via* ice bath. SO_3·py (176 mg, 1.10 mmol) was added, and the reaction mixture was allowed to warm to room temperature and stir for 1.5 hours. Water (5 mL) was added, and the organic layer was separated. The aqueous layer was extracted with CH_2Cl_2 (3 x 10 mL). The organic extracts were dried over $MgSO_4$, filtered, and concentrated *in vacuo*. The crude product was purified by column chromatography (SiO_2, 2% Et_2O in hexanes) affording **aldehyde 62** (135 mg, 97%) as a colorless oil.

aldehyde 62

^{1}H NMR (500 MHz, $CDCl_3$) δ 9.95 (s, 1H), 5.32 (s, 1H), 2.71 (d, *J* = 15.2 Hz, 1H), 2.52 (d, *J* = 15.2 Hz, 1H), 2.12-2.04 (m, 2H), 1.99 (s, 1H), 1.96 (s, 1H), 1.81-1.61 (m, 4H), 1.70 (s, 3H), 1.27 (d, *J* = 14.1 Hz, 1H), 1.23 (q, *J* = 11.4 Hz, 1H), 1.18 (s, 3H), 1.14 (dd, *J* = 13.1, 4.5 Hz, 1H), 0.85 (s, 9H), 0.84 (s, 3H), 0.08 (s, 6H) ppm; **^{13}C NMR** (125 MHz, $CDCl_3$) δ 204.1, 139.5, 123.9, 77.0, 56.3, 46.2, 45.1, 42.6, 42.2, 40.7, 39.5, 38.5, 34.7, 25.9, 22.3, 21.9, 21.2, 19.2, 18.2, –1.71, –1.74 ppm; $[\alpha]_D^{22.4}$ **=** +76.0 (*c* 1.68, CH_2Cl_2); **FTIR** (film) ν_{max} 2953, 2928, 2856, 1717 cm^{-1}; **HRMS** (CI) calculated for $C_{23}H_{40}O_2Si$ $[M+H]^+$ 377.2876 found 377.2877.

alcohol S18

^{1}H NMR (500 MHz, $CDCl_3$) δ 5.21 (ddq, *J* = 3.8, 2.8, 1.5 Hz, 1H), 3.87 (ddd, *J* = 11.4, 10.4, 5.6 Hz, 1H), 3.77 (ddd, *J* = 11.4, 10.4, 5.3 Hz, 1H), 2.15 (tdd, *J* = 13.3, 11.4, 5.6 Hz, 1H), 2.06 (ddd, *J* = 17.7, 4.5, 1.3 Hz, 1H), 1.96 (dt, *J* = 17.7, 2.4 Hz, 1H), 1.80-1.70 (m, 3H), 1.70 (s, 3H), 1.66-1.54 (m, 5H), 1.22-1.18 (m, 2H), 1.16 (s, 3H), 1.01-0.94 (m, 1H), 0.92 (s, 3H), 0.86 (s, 9H), 0.08 (s, 6H) ppm; **^{13}C NMR** (125 MHz, $CDCl_3$) δ 141.6, 122.7, 77.0, 61.1, 56.8, 45.0, 43.0, 42.5, 40.2, 39.5, 38.6, 35.5, 33.2, 26.0, 22.5, 22.4, 21.1, 20.0, 18.2, –1.67, –1.69 ppm; $[\alpha]_D^{22.6}$ **=** +52.7 (*c* 1.83, CH_2Cl_2); **FTIR** (film) ν_{max} 3324 (br), 2953, 2927, 2856 cm^{-1}; **HRMS** (CI) calculated for $C_{23}H_{42}O_2Si$ $[M+H]^+$ 379.3032 found 379.3033.

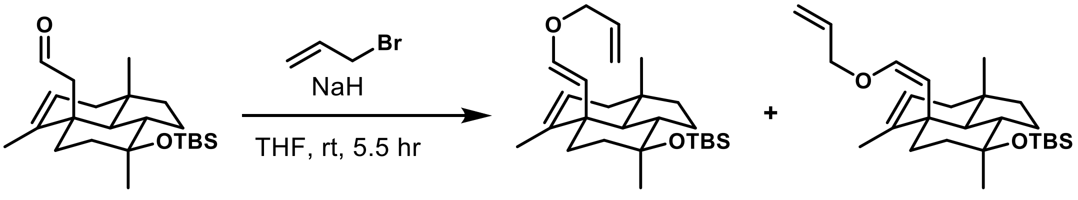

62 **68** **S19**

To a 100 mL round bottom flask equipped with a stir bar was added NaH (60% dispersion in mineral oil, 400 mg, 10.0 mmol). The solids were slurried in hexanes (10 mL) then allowed to settle to the bottom of the vessel and the supernatant was removed *via* syringe. This process was repeated three times to ensure the removal of mineral oil from the reaction vessel. THF (9.5 mL) was added to the reaction vessel and the mixture was allowed to stir at room temperature until a suspension was formed. A solution of **aldehyde 62** (754 mg, 2.00 mmol) in THF (7.5 mL) was added to the reaction vessel at room temperature, rinsing with THF (3 x 1 mL). The reaction mixture was allowed to stir at room temperature for 5.5 hours. Neat allyl bromide (518 µL, 6.00 mmol) was added to the reaction vessel, and the reaction mixture was allowed to stir at room temperature for 18 hours. The reaction mixture was then cooled to 0 °C *via* ice bath and quenched with saturated $NaHCO_{3(aq)}$ (40 mL). The mixture was allowed to warm to room temperature and extracted with Et_2O (3 x 120 mL). The organic extracts were dried over $MgSO_4$, filtered, and concentrated *in vacuo*. The crude product was purified by column chromatography (SiO_2, hexanes) affording ***E*-alkenyl ether 68** (681 mg, 82%) and ***Z*-alkenyl ether S19** (19 mg, 2%) as colorless oils.

***E*-alkenyl ether 68**

^{1}H NMR (500 MHz, $CDCl_3$) δ 6.09 (d, *J* = 12.9 Hz, 1H), 5.94 (ddt, *J* = 16.6, 10.7, 5.4 Hz, 1H), 5.34-5.31 (m, 1H), 5.30 (d, *J* = 0.7 Hz, 1H), 5.22 (dd, *J* = 10.5, 1.3 Hz, 1H), 5.14 (d, *J* = 12.9 Hz, 1H), 4.23 (dd, *J* = 13.3, 5.4 Hz, 1H) 4.20 (dd, *J* = 13.6, 5.4 Hz, 1H), 2.15-2.03 (m, 2H), 1.95 (appar d, *J* = 17.5 Hz, 1H), 1.85 (td, *J* = 13.7, 4.0 Hz, 1H), 1.77-1.67 (m, 2H), 1.66 (d, *J* = 1.2 Hz, 3H), 1.64-1.56 (m, 2H), 1.26-1.17 (m, 1H), 1.16 (s, 3H), 0.88 (s, 3H), 0.85 (s, 9H), 0.08 (s, 6H) ppm; **^{13}C NMR** (125 MHz, $CDCl_3$) δ 146.9, 138.5, 134.0, 123.7, 117.4, 105.8, 77.4, 70.4, 55.8, 44.7, 42.8, 42.2, 41.4, 39.5, 38.9, 35.7, 25.9, 22.5, 21.7, 21.2, 18.2, 18.1, –1.8, –1.9 ppm; $[\alpha]_D^{22.4}$ = +109.7 (*c* 1.00, $CHCl_3$); **FTIR** (film) ν_{max} 2953, 2928, 2857, 1643 cm^{-1}; **HRMS** (CI) calculated for $C_{26}H_{44}O_2Si$ [M–(*t*-Bu)]$^+$ 359.2406 found 359.2402.

***Z*-alkenyl ether S19**

^{1}H NMR (600 MHz, $CDCl_3$) δ 5.95-5.82 (m, 2H), 5.30 (d, *J* = 17.2 Hz, 1H), 5.18 (d, *J* = 10.2 Hz, 1H), 5.18 (s, 1H), 4.38 (d, *J* = 7.5 Hz, 1H), 4.24 (dd, *J* = 13.5, 5.1 Hz, 1H), 4.17 (dd, *J* = 13.5, 5.2 Hz, 1H), 2.63 (dt, *J* = 12.8, 3.4 Hz, 1H), 2.09 (ddd, *J* = 13.7, 10.7, 6.0 Hz, 1H), 2.03 (dd, *J* = 17.0, 4.8 Hz, 1H), 1.92 (appar. d, *J* = 17.1 Hz, 1H), 1.81 (td, *J* = 13.4, 3.6 Hz, 1H), 1.76-1.67 (m, 1H), 1.73 (bs, 3H), 1.66-1.55 (m, 3H), 1.21-1.12 (m, 2H), 1.15 (s, 3H), 0.98-0.90 (m, 1H), 0.94 (s, 3H), 0.85 (s, 9H), 0.07 (s, 6H) ppm; **^{13}C NMR** (150 MHz, $CDCl_3$) δ 144.3, 140.5, 134.2, 121.5, 117.0, 105.6, 77.3, 73.0, 56.4, 45.4, 43.8, 42.7, 40.6, 38.9, 34.1, 26.0, 22.5, 21.4, 21.3, 18.8, 18.2, –1.7, –1.8 ppm; $[\alpha]_D^{22.4}$ = +192.6 (*c* 1.00, $CHCl_3$); **FTIR** (film) ν_{max} 2954, 2930, 2856, 2827, 1639 cm^{-1}; **HRMS** (CI) calculated for $C_{26}H_{44}O_2Si$ [M]$^+$ 416.3111 found 416.3091.

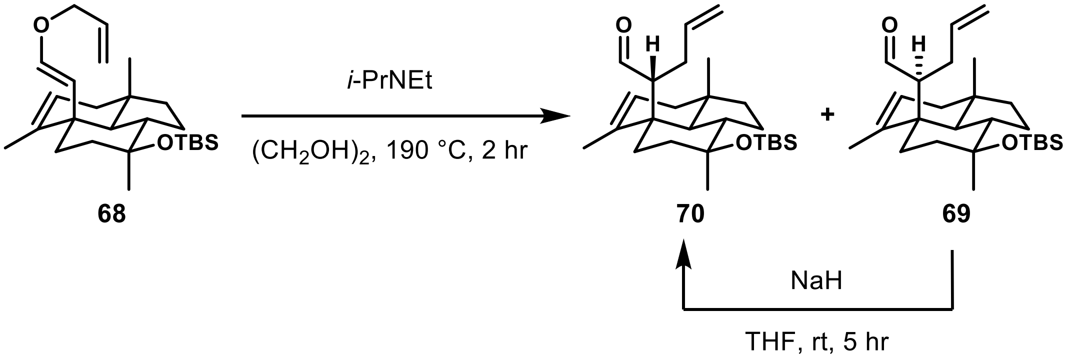

To a 250 mL round bottom flask equipped with a stir bar was added ***E*-alkenyl ether 68** (681 mg, 1.63 mmol) and ethylene glycol (33 mL). The mixture was sparged with argon under sonication for 30 minutes, then degassed *i*-Pr_2NEt (854 µL, 4.90 mmol) was added. The reaction mixture was heated to 190 °C *via* oil bath and allowed to stir at 190 °C for 2 hours. Upon consumption of starting material

as indicated by TLC analysis, the reaction mixture was allowed to cool to room temperature, diluted with brine (150 mL), and partitioned. The aqueous layer was extracted with hexanes (4 x 300 mL). The organic extracts were dried over $MgSO_4$, filtered, and concentrated *in vacuo*. The crude product was purified by column chromatography (SiO_2, 10% CH_2Cl_2 in hexanes) affording ***R*-allyl aldehyde 70** (239 mg, 26%) and ***S*-allyl aldehyde 69** (473 mg, 69%) as colorless oils.

***S*-allyl aldehyde 69** can be epimerized according to the following protocol:

To a 1-dram vial equipped with a stir bar was added NaH (60% dispersion in mineral oil, 10 mg, 0.25 mmol). The solids were slurried in hexanes (1 mL) then allowed to settle to the bottom of the vessel and the supernatant was removed *via* syringe. This process was repeated three times to ensure the removal of mineral oil from the reaction vessel. A solution of ***S*-allyl-aldehyde 69** (5 mg, 0.0120 mmol) in THF (120 µL) was added in one portion. The reaction mixture was allowed to stir at room temperature for 5 hours. Upon consumption of starting material as indicated by TLC analysis, the reaction mixture was cooled to –50 °C *via* $MeCN/Me_2CO/CO_{2(s)}$ bath and quenched by dropwise addition of MeOH (30 µL). The quenched reaction mixture was allowed to warm to room temperature, diluted with water (0.5 mL), and extracted with Et_2O (3 x 2 mL). The organic extracts were purified via column chromatography (SiO_2, 10% CH_2Cl_2 in hexanes) to afford ***R*-allyl aldehyde 70** (2.1 mg, 43%) and ***S*-allyl aldehyde 69** (0.7 mg, 13%) as colorless oils.

***R*-allyl-aldehyde 70**
^{1}H NMR (600 MHz, $CDCl_3$) δ 9.73 (d, *J* = 5.4 Hz, 1H), 5.58 (dddd, *J* = 17.6, 9.8, 7.6, 5.9 Hz, 1H), 5.43 (ddq, *J* = 4.1, 2.8, 1.1 Hz, 1H), 4.98 (d, 5.9 Hz, 1H), 4.96 (s, 1H), 2.69 (ddd, *J* = 11.3, 5.9, 1.1 Hz, 1H), 2.60 (ddd, *J* = 15.8, 5.9, 1.1 Hz, 1H), 2.28 (ddd, *J* = 15.8, 11.3, 7.6 Hz, 1H), 2.16-2.09 (m, 2H), 2.06 (dt, *J* = 18.9, 2.0 Hz, 1H), 1.96 (dt, *J* = 14.5, 3.5 Hz, 1H), 1.81-1.72 (m, 1H), 1.75, (s, 3H), 1.68 (dd, *J* = 10.0, 5.1 Hz, 1H), 1.66-1.62 (m, 1H), 1.61-1.56 (m, 2H), 1.52 (dt, *J* = 13.6, 3.5 Hz, 1H), 1.35 (d, *J* = 13.6 Hz, 1H), 1.17 (s, 3H), 1.16-1.09 (m, 2H), 0.92 (s, 3H), 0.85 (s, 9H), 0.09 (s, 3H), 0.08 (s, 3H) ppm; **^{13}C NMR** (150 MHz, $CDCl_3$) δ 203.3, 138.0, 135.8, 126.0, 116.8, 76.7, 57.9, 51.0, 44.7, 44.1, 43.5, 41.6, 39.1, 37.8, 33.4, 32.8, 25.9, 22.4, 21.7, 21.1, 20.5, 18.2, –1.7, –1.8 ppm; $[\alpha]_D^{22.4}$ = +61.0 (*c* 1.48, CH_2Cl_2); **FTIR** (film) ν_{max} 2953, 2927, 2876, 2856, 2707, 1723, 1641 cm^{-1} **HRMS** (ESI) calculated for $C_{26}H_{44}O_2Si$ $[M+Na]^+$ 439.3008 found 439.2994.

***S*-allyl-aldehyde 69**
^{1}H NMR (500 MHz, $CDCl_3$) δ 9.50 (d, *J* = 4.8 Hz, 1H), 5.65 (dddd, *J* = 17.9, 10.8, 8.8, 7.0 Hz, 1H), 5.48 (ddq, *J* = 4.2, 2.8, 1.3 Hz, 1H), 5.03 (s, 1H), 5.00 (dq, *J* = 7.0, 1.3 Hz, 1H), 2.63 (ddd, *J* = 11.6, 4.7, 3.1 Hz, 1H), 2.58 (ddq, *J* = 14.3, 6.5, 1.3 Hz, 1H), 2.33 (ddd, *J* = 14.3, 11.6, 7.0 Hz, 1H), 2.18-2.09 (m, 2H), 2.05 (ddq, *J* = 18.0, 4.2, 1.3 Hz, 1H), 1.99 (dquin, *J* = 18.0, 2.3 Hz, 1H), 1.77 (q, *J* = 1.3 Hz, 3H), 1.75-1.54 (m, 6H), 1.43 (d, *J* = 13.8 Hz, 1H), 1.20 (s, 3H), 1.16 (q, *J* = 11.0 Hz, 1H), 1.06 (td, *J* = 14.2, 3.7 Hz, 1H), 0.85 (s, 9H), 0.81 (s, 3H), 0.08 (s, 3H), 0.07 (s, 3H) ppm; **^{13}C NMR** (125 MHz, $CDCl_3$) δ 204.3, 137.5, 136.0, 126.1, 117.0, 76.8, 57.5, 51.2, 44.6, 44.2, 41.83, 41.76, 40.3, 37.8, 34.5, 32.5, 25.9, 22.54, 22.53, 21.1, 20.6, 18.2, –1.67, –1.72 ppm; $[\alpha]_D^{22.5}$ = +125.2 (*c* 1.80, CH_2Cl_2); **FTIR** (film) ν_{max} 2953, 2928, 2882, 1717, 1640 cm^{-1}; **HRMS** (CI) calculated for $C_{26}H_{44}O_2Si$ $[M+H]^+$ 417.3189 found 417.3191.

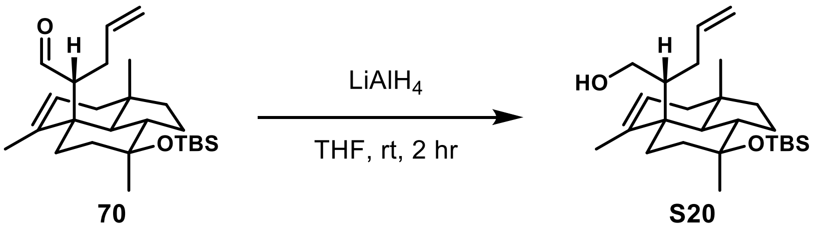

70 **S20**

To a 25 mL round bottom flask equipped with a stir bar was added a solution of ***R*-allyl-aldehyde 70** (356 mg, 0.856 mmol) in THF (7.6 mL). The vessel was cooled to 0 °C *via* ice bath. $LiAlH_4$ (145 mg, 3.83 mmol) was added portionwise at 0 °C. The reaction mixture was allowed to warm to room temperature and stir for 2 hours. Upon consumption of starting material as indicated by TLC analysis, the reaction mixture was cooled to 0 °C *via* ice bath and quenched with saturated Rochelle's salt (20 mL). Et_2O (20 mL) was added and the resulting mixture was stirred vigorously for 1 hour, then partitioned. The aqueous layer was extracted with Et_2O (2 x 20 mL). The organic extracts were dried over $MgSO_4$, filtered, and concentrated *in vacuo* to afford crude **alcohol S20** which was used without further purification.

alcohol S20

^{1}H NMR (500 MHz, $CDCl_3$) δ 5.81 (dtd, *J* = 17.0, 9.7, 4.3 Hz, 1H), 5.28 (bs, 1H), 5.09 (d, *J* = 17.1 Hz, 1H), 5.00 (d, *J* = 10.2 Hz, 1H), 3.93-3.81 (m, 2H), 2.61 (d, *J* = 15.3 Hz, 1H), 2.18-1.98 (m, 5H), 1.93-1.87 (m, 1H), 1.80-1.61 (m, 8H), 1.56 (dd, *J* = 11.5, 8.0 Hz, 1H), 1.40 (d, *J* = 13.7 Hz, 1H), 1.18 (s, 3H), 1.16-1.00 (m, 2H), 0.96 (s, 3H), 0.85 (s, 9H), 0.07 (s, 3H), 0.06 (s, 3H) ppm; **^{13}C NMR** (125 MHz, $CDCl_3$) δ 140.8, 140.2, 124.5, 115.7, 77.1, 63.7, 57.9, 45.0, 44.7, 43.1, 41.9, 41.4, 38.1, 37.8, 35.9, 33.9, 26.0, 22.8, 21.8, 20.8, 20.4, 18.2, –1.67, –1.70 ppm; $[\alpha]_D^{22.6}$ = +82.4 (*c* 0.41, CH_2Cl_2); **FTIR** (film) ν_{max} 3406 (br), 2952, 2928, 2894, 2856, 1638 cm^{-1}; **HRMS** (CI) calculated for $C_{26}H_{46}O_2Si$ $[M+H]^+$ 419.3345 found 419.3336.

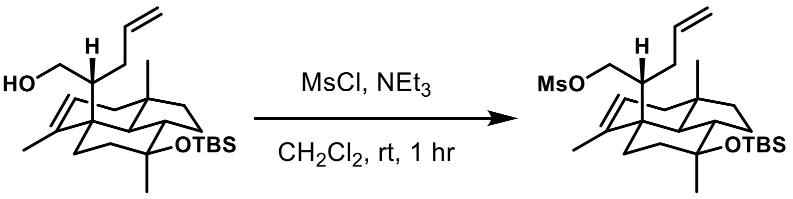

S20 **S21**

To a 25 mL round bottom flask equipped with a stir bar was added NEt_3 (600 µL, 4.29 mmol) and a solution of crude **alcohol S20** in CH_2Cl_2 (8.6 mL). The mixture was cooled to 0 °C *via* ice bath, and MsCl (166 µL, 2.14 mmol) was added dropwise. The reaction mixture was allowed to warm to room temperature and stir for 1 hour. Upon consumption of starting material as indicated by TLC analysis, the reaction mixture was cooled to 0 °C *via* ice bath and quenched with saturated $NaHCO_{3(aq)}$ (10 mL). The resulting mixture was allowed to warm to room temperature and partitioned. The aqueous layer was extracted with CH_2Cl_2 (2 x 10 mL). The organic extracts were dried over $MgSO_4$, filtered, and concentrated *in vacuo*. The crude product was purified by column chromatography (SiO_2, 5% Et_2O in hexanes) affording **mesylate S21** (420 mg, 99% over two steps) as a colorless oil.

mesylate S21

^{1}H NMR (500 MHz, $CDCl_3$) δ 5.71 (dddd, *J* = 17.4, 10.7, 8.5, 4.2 Hz, 1H), 5.33 (s, 1H), 5.09 (d, *J* = 10.7, 1H), 5.07 (quin, *J* = 1.7 Hz, 1H), 4.47 (dt, *J* = 10.7, 1.7 Hz, 1H), 4.39 (dd, *J* = 10.1, 2.9 Hz, 1H), 3.00 (s, 3H), 2.6 (d, *J* = 14.8 Hz, 1H), 2.13-2.02 (m, 5H), 1.96 (ddd, *J* = 14.0, 10.1, 4.6 Hz, 1H), 1.77 (dt, *J* = 13.6, 3.5 Hz, 1H), 1.79-1.71 (m, 1H), 1.68 (s, 3H), 1.69-1.63 (m, 1H), 1.61-1.52 (m, 3H), 1.44 (d, *J* = 13.6 Hz, 1H), 1.19 (s, 3H), 1.19-1.09 (m, 2H), 0.97 (s, 3H), 0.85 (s, 9H), 0.08 (s, 3H), 0.07 (s, 3H) ppm; **^{13}C NMR** (125 MHz, $CDCl_3$) δ 139.7, 137.8, 125.5, 117.3, 76.9, 69.5, 57.7, 44.9, 44.8, 42.7, 41.8, 41.2, 37.8, 37.4, 36.1, 34.5, 33.8, 26.0, 22.8, 21.7, 20.6, 20.2, 18.2, –1.6 ppm; $[\alpha]_D^{22.6}$ = +65.5 (*c* 1.04, CH_2Cl_2); **FTIR** (film) ν_{max} 2952, 2923, 2892, 2856, 1639 cm^{-1}; **HRMS** (ESI) calculated for $C_{27}H_{48}O_4SSi$ $[M+Na]^+$ 519.2940 found 519.2948.

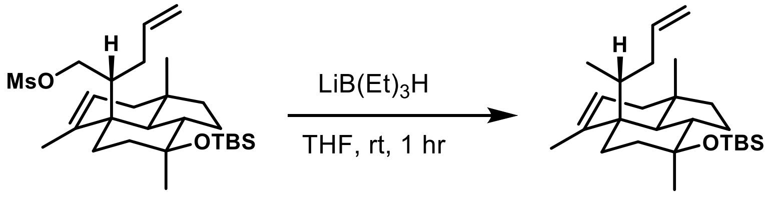

S21 **71**

To a 50 mL round bottom flask equipped with a stir bar was added **mesylate S21** (394 mg, 0.794 mmol) and $LiB(Et)_3H$ (1.0 M in THF, 15.9 mL, 15.9 mmol). The reaction mixture was allowed to stir at room temperature for 1 hour. Upon consumption of starting material as indicated by TLC analysis, the reaction mixture was cooled to 0 °C *via* ice bath and quenched by dropwise addition of methanol (0.5 mL). The mixture was allowed to warm to room temperature, diluted with water (20 mL) and hexanes (20 mL), and partitioned. The aqueous layer was extracted with hexanes (2 x 20 mL). The organic extracts were dried over $MgSO_4$, filtered, and concentrated *in vacuo*. The crude product was purified by column chromatography (SiO_2, hexanes) affording **diene 71** (275 mg, 86%) as a colorless oil.

diene 71

^{1}H NMR (500 MHz, $CDCl_3$) δ 5.74 (dtd, *J* = 18.2, 8.9, 4.5 Hz, 1H), 5.26 (ddt, *J* = 4.5, 2.8, 1.5 Hz, 1H), 4.99 (d, *J* = 5.2 Hz, 1H), 4.97 (s, 1H), 2.69 (dd, *J* = 15.0, 3.7 Hz, 1H), 2.12-2.03 (m, 4H), 1.84-1.51 (m, 10H), 1.39 (d, *J* = 13.6 Hz, 1H), 1.19 (s, 3H), 1.09 (d, *J* = 7.0 Hz, 3H), 1.03-0.97 (m, 1H), 0.96 (s, 3H), 0.86 (s, 9H), 0.08 (s, 3H), 0.07 (s, 3H) ppm; **^{13}C NMR** (125 MHz, $CDCl_3$) δ 141.0, 139.8, 123.8, 115.3, 77.0, 57.8, 45.2, 44.6, 42.9, 41.5, 41.4, 41.3, 37.7, 34.2, 30.2, 26.0, 22.7, 22.0, 21.0, 20.8, 18.2, 18.1, –1.67, –1.71 ppm; $[\alpha]_D^{22.6}$ = +87.0 (*c* 1.02, CH_2Cl_2); **FTIR** (film) ν_{max} 3076, 2955, 2928, 2895, 2856, 1639 cm^{-1}; **HRMS** (CI) calculated for $C_{26}H_{46}OSi$ $[M]^+$ 402.3318 found 402.3305.

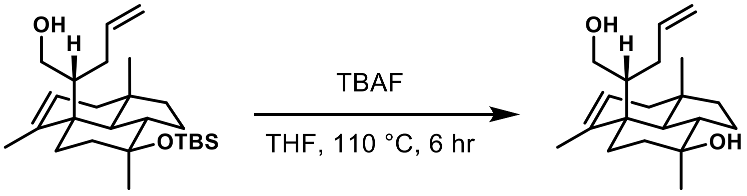

S20 S22

To a 5 mL microwave tube equipped with stir bar was added crude **alcohol S20** (10.0 mg, 23.9 μmol) and TBAF (1.0 M in THF, 1.20 mL, 1.20 mmol). The mixture was sparged with argon under sonication for 30 minutes, then subject to microwave irradiation with stirring at 110 °C for 6 hours. The mixture was allowed to cool to room temperature, diluted with saturated $NH_4Cl_{(aq)}$ (3 mL), and extracted with Et_2O (3 x 3 mL). The organic extracts were washed with brine (1 x 3 mL), dried over $MgSO_4$, filtered, and concentrated *in vacuo*. The crude product was purified by column chromatography (SiO_2, 30% to 50% EtOAc in hexanes, stepped gradient) affording **diol S22** (5.2 mg, 85%) as a white solid. **Diol S22** was dissolved in a mixture of Et_2O and hexanes, then crystallized by slow evaporation to afford X-ray quality crystals.

diol S22

^{1}H NMR (500 MHz, $CDCl_3$) δ 5.79 (dtd, *J* = 17.4, 9.9, 4.3 Hz, 1H), 5.32-5.29 (m, 1H), 5.09 (dt, *J* = 17.4, 1.8 Hz, 1H), 5.01 (dt, *J* = 9.9, 1.8 Hz, 1H), 3.89 (dd, *J* = 11.4, 4.5 Hz, 1H), 3.86 (dd, *J* = 11.4, 4.5 Hz, 1H), 2.62 (d, *J* = 15.9 Hz, 1H), 2.18-2.08 (m, 4H), 2.05 (ddd, *J* = 15.3, 10.8, 4.8 Hz, 1H), 1.94-1.89 (m, 1H), 1.84-1.65 (m, 7H), 1.62 (dd, *J* = 11.6, 8.1 Hz, 1H), 1.44 (d, *J* = 13.5 Hz, 1H), 1.22 (s, 3H), 1.16 (q, *J* = 10.7 Hz, 1H), 1.10 (td, *J* = 14.0, 4.8 Hz, 1H), 0.98 (s, 3H) ppm; **^{13}C NMR** (125 MHz, $CDCl_3$) δ 140.6, 140.1, 124.5, 115.8, 74.5, 63.9, 58.4, 45.1, 44.1, 43.1, 41.5, 41.2, 38.0, 37.8, 35.9, 33.9, 22.2, 21.8, 20.5, 20.3 ppm; $[\alpha]_D^{22.7}$ = +96.6 (*c* 0.50, CH_2Cl_2); **FTIR** (film) ν_{max} 3362 (br), 3072, 2945, 2873, 2829, 1647 cm^{-1}; **m.p.** = 149 °C; **HRMS** (ESI) calculated for $C_{20}H_{32}O_2$ $[M+Na]^+$ 327.2300 found 327.2290.

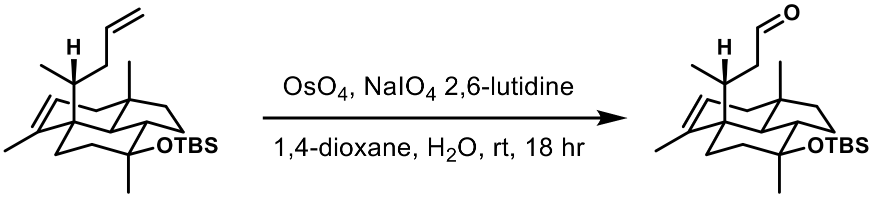

71 72

To a 1-dram vial equipped with a stir bar was added a 3:1 mixture of 1,4-dioxane/water (2.0 mL), **diene 71** (43.1 mg, 0.107 mmol), $NaIO_4$ (69 mg, 0.324 mmol), 2,6-lutidine (87 μL, 0.751 mmol), and OsO_4 (2.5 wt% in *t*-BuOH, 110 μL, 8.56 μmol). The reaction mixture was allowed to stir at room temperature for 18 hours. Upon consumption of starting material as indicated by TLC analysis, the reaction mixture was cooled to 0 °C *via* ice bath and quenched with saturated $Na_2S_2O_{3(aq)}$ (10 mL). The mixture was allowed to warm to room temperature and extracted with pentane (3 x 20 mL). The organic extracts were washed with brine (1 x 20 mL), dried over $MgSO_4$, filtered, and concentrated *in vacuo*. The crude product was purified by column chromatography (SiO_2, 3% EtOAc in hexanes) affording **aldehyde 72** (42 mg, 97%) as a colorless oil.

aldehyde 72

^{1}H NMR (600 MHz, $CDCl_3$) δ 9.72 (d, *J* = 2.3 Hz, 1H), 5.29 (bs, 1H), 3.10 (d, *J* = 18.4 Hz, 1H), 2.66-2.49 (m, 1H), 2.23-2.09 (m, 2H), 2.07 (bs, 3H), 1.78 – 1.69 (m, 1H), 1.69-1.62 (m, 3H), 1.64 (s, 3H), 1.56 (dd, *J* = 11.5, 8.3 Hz, 1H), 1.40 (d, *J* = 13.6 Hz, 1H), 1.18 (s, 3H), 1.15-1.08 (m, 1H), 1.10 (d, *J* = 7.0 Hz, 3H), 1.07-0.98 (m, 1H), 0.86 (s, 9H), 0.86 (s, 3H), 0.09 (s, 3H), 0.08 (s, 3H) ppm; **^{13}C NMR** (150 MHz, $CDCl_3$) δ 203.4, 140.9, 124.3, 76.8, 57.4, 52.3, 45.1, 44.5, 42.5, 41.5, 41.4, 37.6, 33.8, 26.0, 24.3, 22.6, 22.4, 20.8, 20.6, 20.3, 18.2, –1.68, –1.69 ppm; $[\alpha]_D^{22.4}$ = +66.5 (*c* 1.0, $CHCl_3$); **FTIR** (film) ν_{max} 2953, 2857, 1725, 1462, 1379, 1254, 1148, 1116, 1053, 1033 cm^{-1}; **HRMS** (CI) calculated for $C_{25}H_{44}O_2Si$ $[M–(t\text{-Bu})]^+$ 347.2406 found 347.2398.

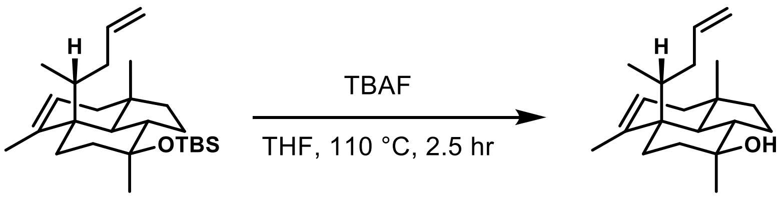

71 **S23**

To a 5 mL microwave tube equipped with a stir bar was added a solution of **diene 71** (50 mg, 0.124 mmol) in THF (400 µL), and TBAF (1.0 M in THF, 4.00 mL, 4.00 mmol). The mixture was sparged with argon under sonication for 30 minutes, then subject to microwave irradiation with stirring at 110 °C for 2.5 hours. The mixture was allowed to cool to room temperature, diluted with saturated $NH_4Cl_{(aq)}$ (20 mL), and extracted with Et_2O (3 x 40 mL). The organic extracts were washed with brine (1 x 40 mL), dried over $MgSO_4$, filtered, and concentrated *in vacuo*. The crude product was purified by column chromatography (SiO_2, 6% EtOAc in hexanes) affording **alcohol S23** (31 mg, 92%) as a colorless oil.

alcohol S23

^{1}H NMR (500 MHz, $CDCl_3$) δ 5.76 – 5.63 (m, 1H), 5.27 (dd, *J* = 4.9, 3.5 Hz, 1H), 5.00-4.97 (m, 1H), 4.97-4.93 (m, 1H), 2.67 (dd, *J* = 15.1, 2.1 Hz, 1H), 2.14 (dt, *J* = 13.9, 3.3 Hz, 1H), 2.12-2.01 (m, 3H), 1.86-1.72 (m, 2H), 1.72-1.52 (m, 8H), 1.43 (s, 1H), 1.40 (bs, 1H), 1.21 (s, 3H), 1.14 (q, *J* = 10.5 Hz, 1H), 1.08 (d, *J* = 6.9 Hz, 3H), 1.03 (td, *J* = 13.4, 4.2 Hz, 1H), 0.97 (s, 3H) ppm; **^{13}C NMR** (125 MHz, $CDCl_3$) δ 140.8, 139.6, 115.4, 74.4, 58.2, 45.2, 44.0, 42.9, 41.23, 41.15, 41.15, 37.6, 34.3, 30.1, 22.1, 21.9, 20.8, 20.6, 18.1 ppm; $[\alpha]_D^{22.4}$ = +67.2 (*c* 1.00, $CHCl_3$); **FTIR** (film) ν_{max} 3348 (br), 3074, 2957, 2938, 2874, 2828, 1639 cm^{-1}; **HRMS** (CI) calculated for $C_{20}H_{32}O$ $[M]^+$ 288.2453 found 288.2459.

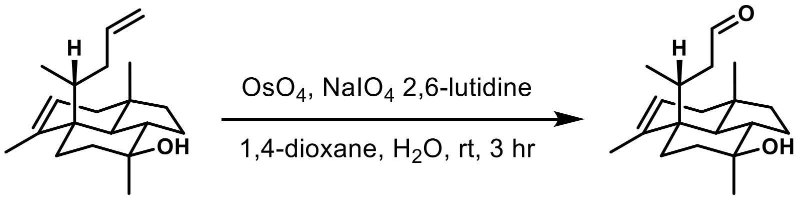

S23 **73**

To a 25 mL round bottom flask equipped with a stir bar was added a 3:1 mixture of 1,4-dioxane/water (2.2 mL), **alcohol S23** (31 mg, 0.108 mmol), $NaIO_4$ (69 mg, 0.324 mmol), 2,6-lutidine (88 µL, 0.756 mmol), and OsO_4 (2.5 wgt% in *t*-BuOH, 111 µL, 8.64 µmol). The reaction mixture was allowed to stir at room temperature for 3 hours. Upon consumption of starting material as indicated by TLC analysis, the reaction mixture was cooled to 0 °C *via* ice bath and quenched with saturated $Na_2S_2O_{3(aq)}$ (10 mL). The mixture was allowed to warm to room temperature and extracted with pentane (3 x 20 mL). The organic extracts were washed with brine (1 x 20 mL), dried over $MgSO_4$, filtered, and concentrated *in vacuo*. The crude product was purified by column chromatography (SiO_2, 15% EtOAc in hexanes) affording **aldehyde 73** (28 mg, 89%) as a colorless oil.

aldehyde 73

^{1}H NMR (600 MHz, $CDCl_3$) δ 9.71 (d, *J* = 2.3 Hz, 1H), 5.31 (appar. d, *J* = 1.3 Hz, 1H), 3.11 (d, *J* = 18.6 Hz, 1H), 2.60 (tt, *J* = 14.0, 7.0 Hz, 1H), 2.19 (ddd, *J* = 18.7, 10.3, 2.4 Hz, 1H), 2.16-2.07 (m, 4H), 1.79 (dtd, *J* = 14.0, 10.9, 8.2 Hz, 1H), 1.74-1.67 (m, 2H), 1.67-1.59 (m, 5H),

1.43 (d, *J* = 13.6 Hz, 1H), 1.21 (s, 3H), 1.16 (q, *J* = 10.8 Hz, 1H), 1.10 (d, *J* = 7.1 Hz, 3H), 1.07 (td, *J* = 14.0, 3.8 Hz), 0.85 (s, 3H) ppm; **^{13}C NMR** (150 MHz, $CDCl_3$) δ 203.2, 140.7, 124.3, 74.3, 58.0, 52.3, 45.1, 44.0, 42.5, 41.3, 41.1, 37.5, 33.9, 24.2, 22.3, 22.0, 20.6, 20.43, 20.40 ppm; $[\alpha]_D^{22.4}$ = +84.4 (*c* 1.00, $CHCl_3$); **FTIR** (film) ν_{max} 3388 (br), 2938, 2876, 2827, 2711, 1722 cm^{-1}; **HRMS** (CI) calculated for $C_{19}H_{30}O_2$ $[M]^+$ 290.2246 found 290.2244.

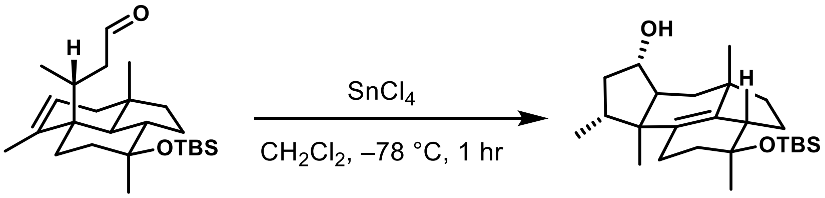

72 74

To a 1-dram vial equipped with stir bar was added **aldehyde 72** (3.6 mg, 8.9 μmol) and CH_2Cl_2 (100 μL). The reaction vessel was cooled to –78 °C *via* $Me_2CO/CO_{2(s)}$ bath. $SnCl_4$ (1 M in hexane) (2.2 μL, 2.2 μmol) was added to the reaction vessel. The reaction mixture was allowed to stir at –78 °C for 1 hour. Upon consumption of starting material as indicated by TLC analysis, the reaction mixture was quenched at –78 °C with saturated $NaHCO_{3(aq)}$ (1 mL). The mixture was allowed to warm to room temperature and extracted with Et_2O (3x2 mL). The oganic extracts were dried over Na_2SO_4, filtered, and concentrated *in vacuo*. Analysis of the crude reaction mixture revealed the presence of rearranged **tetrasubstituted alkene 74**.

tetrasubstituted alkene 74

^{1}H NMR (500 MHz, $CDCl_3$) δ 4.16 (td, *J* = 9.9, 5.6 Hz, 1 H), 2.60 (d, *J* = 11.0 Hz, 1H), 2.16-1.99 (m, 3H), 1.96 (appar. d, *J* = 14.1 Hz, 1H), 1.89-1.74 (m, 2H), 1.74-1.57 (m, 5H), 1.42 (dd, *J* = 14.2, 5.0 Hz, 1H), 1.20 (appar. q, *J* = 10.8 Hz, 1H), 1.08 (s, 3H), 1.04 (d, *J* = 7.3 Hz, 3H), 1.01-0.97 (m, 1H), 0.96 (s, 3H), 0.94 (s, 3H), 0.85 (s, 9H), 0.07 (s, 3H), 0.06 (s, 3H) ppm; **^{13}C NMR** (125 MHz, $CDCl_3$) δ 138.5, 131.0, 88.6, 74.9, 73.4, 56.2, 48.5, 43.8, 43.6, 42.9, 40.6, 39.0, 38.2, 33.8, 25.9, 24.2, 22.4, 22.3, 20.0, 18.3, 17.6, –1.7, –1.8 ppm.

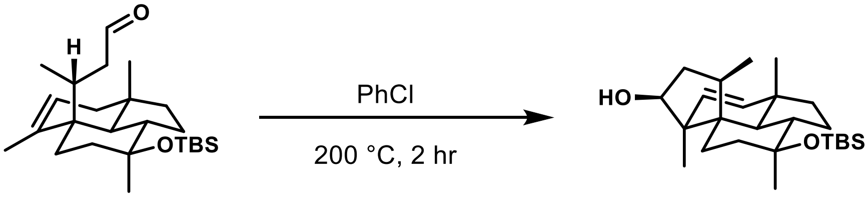

72 75

To a 10 mL microwave vial equipped with a stir bar was added **aldehyde 72** (2.9 mg, 7.4 μmol) and PhCl (1 mL). The reaction vessel was sealed and subject to microwave irradiation at 200 °C with stirring for 2 hours. The reaction vessel was then allowed to cool to room temperature, and the reaction mixture was concentrated *in vacuo*. Analysis of the crude residue revealed the presence of **carbonyl ene product 75**.

carbonyl ene product 75

^{1}H NMR (500 MHz, $CDCl_3$) δ 6.06 (d, *J* = 10.1 Hz, 1H), 5.28 (d, *J* = 10.2 Hz, 1H), 3.78 (d, *J* = 10.4, 5.7 Hz, 1H), 2.27 (quin.d, *J* = 7.2, 2.0 Hz, 1H), 2.20-2.10 (m, 1H), 2.10-2.03 (m, 1H), 1.97 (ddd, *J* = 14.3, 10.3, 6.2 Hz, 1H), 1.84-1.72 (m, 3H), 1.70-1.60 (m, 4 H), 1.36 (dd, *J* = 11.3, 8.8 Hz, 1H), 1.17 (s, 3H), 1.08 (d, *J* = 6.8 Hz, 3H), 1.03 (s, 3H), 0.98 (s, 3H), 0.85 (s, 9H), 0.08-0.05 (m, 6H) ppm; **^{13}C NMR** (125 MHz, $CDCl_3$) δ 138.7, 131.2, 79.3, 76.4, 52.5, 51.8, 45.3, 44.2, 42.4, 42.2, 41.1, 39.6, 30.4, 29.6, 26.0, 22.3, 21.2, 20.8, 20.3, 19.7, 18.3, –1.6, –1.7 ppm.

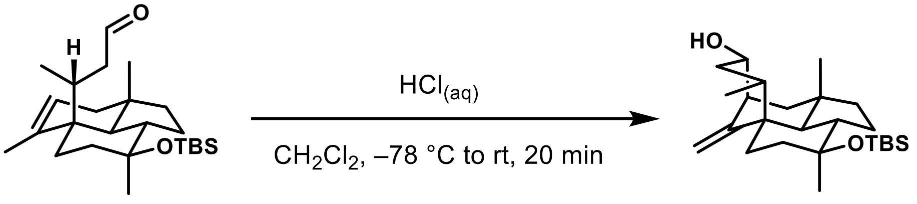

To a 1-dram vial equipped with a stir bar was added **aldehyde 72** (2.9 mg, 7.2 μmol) and CH_2Cl_2 (400 μL). The reaction vessel was cooled to –78 °C *via* $Me_2CO/CO_{2(s)}$ bath. Concentrated $HCl_{(aq)}$ (10 μL) was added to the reaction vessel. The cooling bath was removed, and the reaction mixture was allowed to warm to room temperature, stirring for 20 minutes. Upon consumption of starting material as indicated by TLC analysis, the reaction mixture was cooled to –78 °C and quenched with saturated $NaHCO_{3(aq)}$ (1 mL). The mixture was allowed to warm to room temperature and extracted with CH_2Cl_2 (3x2 mL). The organic extracts were dried over Na_2SO_4, filtered, and concentrated *in vacuo*. NMR spectra of the crude product are consistent with the presence of small quantities of expected **Prins product 76**.

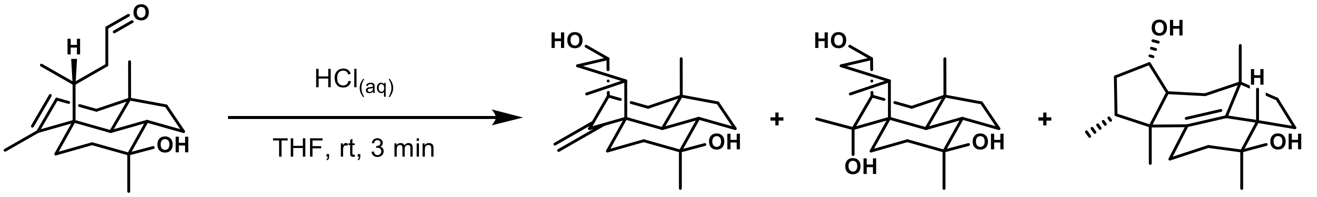

To a 2-dram vial equipped with a stir bar was added **aldehyde 73** (9 mg, 31 μmol), THF (900 μL), and concentrated $HCl_{(aq)}$ (900 μL). The reaction mixture was allowed to stir vigorously at room temperature for 3 minutes. Upon consumption of starting material as indicated by TLC analysis, the reaction was quenched by addition of saturated $NaHCO_{3(aq)}$ (1.5 mL) followed by $NaHCO_{3(s)}$ until the evolution of gas subsided. The mixture was extracted with Et_2O (3x3 mL). The organic extracts were dried over $MgSO_4$, filtered, and concentrated *in vacuo*. The crude product was purified by column chromatography (SiO_2, 15% to 20% EtOAc in hexanes, stepped gradient; SiO_2, 15% EtOAc in 1:1 hexanes:CH_2Cl_2) affording **tetrasubstituted alkene 80** as a white solid, desired **diol 77** as a white solid, and **triol 78** as a white solid. Characterization data obtained for the desired **diol 77** are in good agreement with those previously reported.[10] **Tetrasubstituted alkene 80** was dissolved in a mixture of MeOH/hexanes then crystallized by slow evaporation to produce X-ray quality crystals.

tetrasubstituted alkene 80

^{1}H NMR (600 MHz, $CDCl_3$) δ 4.16 (td, *J* = 10.0, 5.8 Hz, 1H), 2.60 (d, *J* = 10.8 Hz, 1H), 2.15 (ddt, *J* = 17.6, 6.7, 1.7 Hz, 1H), 2.11-2.03 (m, 2H), 1.99 (dd, *J* = 14.1, 2.2 Hz, 1H), 1.91-1.80 (m, 2H), 1.74-1.64 (m, 3H), 1.64-1.57 (m, 2H), 1.44 (dd, *J* = 14.1, 5.6 Hz, 1H), 1.22-1.17 (m, 1H), 1.10 (s, 3H), 1.05 (d, *J* = 7.1 Hz, 3H), 1.04-0.99 (m, 1H) 0.98 (s, 6H) ppm; **^{13}C NMR** (150 MHz, $CDCl_3$) δ 138.2, 131.4, 73.3, 72.3, 56.1, 47.9, 43.8, 43.6, 42.9, 40.6, 38.9, 38.1, 33.7, 25.8, 24.1, 22.4, 21.9, 19.5, 17.6 ppm; **HRMS** (CI) calculated for $C_{19}H_{30}O_2$ $[M]^+$ 290.2246 found 290.2251.

diol 77

^{1}H NMR (500 MHz, $CDCl_3$) δ 4.77 (s, 1H), 4.63 (s, 1H), 4.26 (t, *J* = 7.3 Hz, 1H), 2.35 (dd, *J* = 4.9, 2.2 Hz, 1H), 2.23-2.11 (m, 2H), 2.04 (dd, *J* = 13.1, 2.4 Hz, 1H), 2.00-1.93 (m, 1H), 1.93-1.87 (m, 1H), 1.86-1.78 (m, 1H), 1.72 (dt, *J* = 12.9, 3.4 Hz, 1H), 1.63-1.59 (m, 1H), 1.54-1.39 (m, 4H), 1.33-1.27 (m, 2H), 1.19 (s, 3H), 1.03 (s, 3H), 1.01-0.96 (m, 1H), 0.94 (d, *J* = 6.2 Hz, 3H), 0.91-0.86 (m, 2H) ppm; **^{13}C NMR** (125 MHz, $CDCl_3$) δ 154.7, 106.0, 73.9, 73.4, 59.5, 52.1, 45.8, 44.5, 43.3, 43.1, 41.5, 41.3, 39.9, 30.4, 27.8, 21.7, 20.8, 20.52, 20.46 ppm; $[\alpha]_D^{22.4}$ = –3.1 (*c* 1.00, $CHCl_3$); **FTIR** (film) ν_{max} 3350 (br), 2926, 2852, 1640 cm^{-1}; **HRMS** (CI) calculated for $C_{19}H_{30}O_2$ $[M]^+$ 290.2246 found 290.2241.

triol 78

^{1}H NMR (500 MHz, $CDCl_3$) δ 4.26 (dd, *J* = 9.7, 6.7 Hz, 1H), 2.52 (dt, *J* = 16.0, 9.3 Hz, 1H), 2.14-2.07 (m, 1H), 1.93-1.72 (m, 3H), 1.71-1.40 (m, 9H), 1.37 (s, 3H), 1.28-1.23 (m, 1H), 1.22 (s, 3H), 1.11-1.07 (m, 1H), 1.05 (d, *J* = 7.1 Hz, 3H), 0.91 (s, 3H) ppm; **^{13}C NMR** (125 MHz, $CDCl_3$) δ 73.9, 73.8, 72.2, 52.3, 50.2, 43.4, 41.2, 41.1, 40.8, 40.7, 38.9, 27.1, 25.8, 24.8, 21.9, 21.6, 20.6, 19.8 ppm; $[\alpha]_D^{22.4}$ = +1.8 (*c* 0.50, $CHCl_3$); **FTIR** (film) ν_{max} 3393 (br), 2927, 1463, 1383, 1099 cm^{-1}; **HRMS** (CI) calculated for $C_{19}H_{32}O_3Na$ $[M+Na]^+$ 331.2249 found 331.2250.

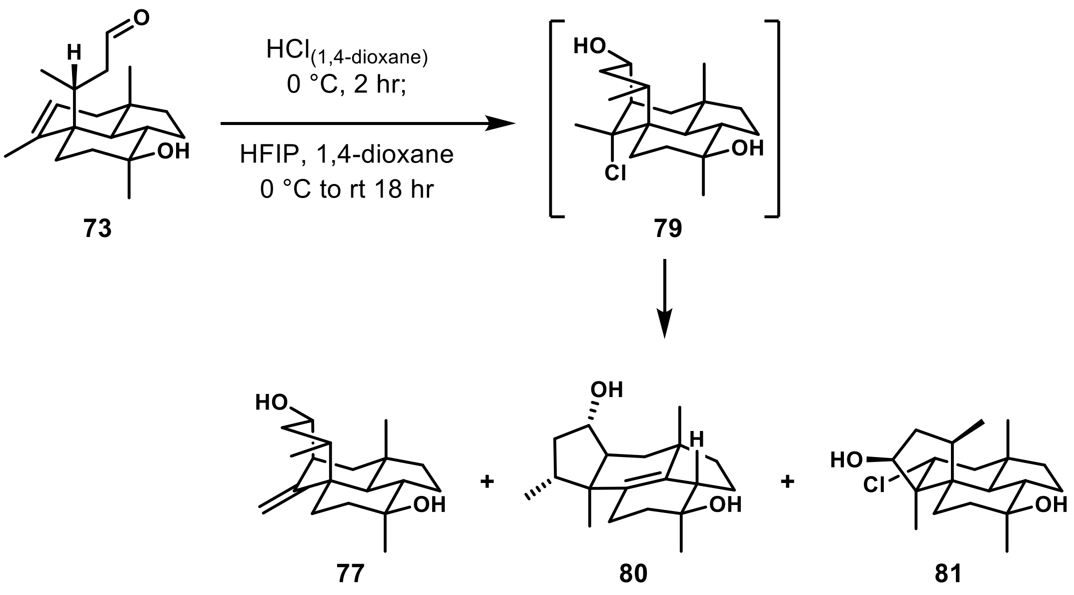

To a 2-dram vial equipped with stir bar was added **aldehyde 73** (14.6 mg, 50 μmol). The reaction vessel was cooled to 0 °C *via* ice bath, and HCl (4 M in 1,4-dioxane, 600 μL, 2.4 mmol) was added. The reaction mixture was allowed to stir at 0 °C for 2 hours, then allowed to warm to room temperature and concentrated *in vacuo*. Analysis of the concentrated reaction mixture revealed the presence of **chlorinated intermediate 79**. The resulting residue was cooled to 0 °C *via* ice bath, and a 1:1 mixture of HFIP/1,4-dioxane (1.2 mL) was added streamwise down the sides of the reaction vessel. The reaction mixture was allowed to warm slowly to room temperature, stirring for 18 hours, then concentrated *in vacuo*. The crude product was purified by column chromatography (SiO_2, 8% EtOAc in 1:1 hexanes/DCM followed by 15% EtOAc in hexanes) affording **diol 77** (6.8 mg, 48%) as a white solid, **chloride 81** as a colorless oil (1.5 mg, 9%), and **tetrasubstituted alkene 80** (4.1 mg, 28%) as a white solid.

chlorinated intermediate 79

^{1}H NMR (600 MHz, $CDCl_3$) δ 4.34 (dd, *J* = 9.8, 6.2 Hz, 1H), 2.59 (dt, *J* = 16.1, 9.7 Hz, 1 H), 2.22-2.13 (m, 2H), 2.11-2.08 (m, 1H), 1.83 (s, 3H), 1.82-1.55 (m, 8H), 1.48-1.40 (m, 2H), 1.31 (ddd, *J* = 15.4, 9.2, 6.2 Hz, 1H), 1.23 (s, 3H), 1.15-1.11 (m, 1H), 1.09 (d, *J* = 6.9 Hz, 3H), 0.93 (s, 3H). **^{13}C NMR** (125 MHz, $CDCl_3$) δ 86.2, 73.9, 72.5, 54.7, 52.0, 44.2, 43.3, 42.5, 42.0, 41.0, 40.6, 38.9, 28.5, 27.8, 27.0, 22.2, 21.6, 20.6, 19.5 ppm; **HRMS** (CI) calculated for $C_{19}H_{31}ClO_2$ $[M]^+$ 326.2013 found 326.2010.

chloride 81

^{1}H NMR (500 MHz, $CDCl_3$) δ 4.21-4.09 (m, 2H), 2.57-2.41 (m, 2H), 2.19-2.06 (m, 2H), 1.88-1.68 (m, 4H), 1.68-1.58 (m, 2H), 1.52-1.31 (m, 3H), 1.18 (m, 8H), 1.06 (s, 3H), 1.05 (s, 3H) ppm; **^{13}C NMR** (150 MHz, $CDCl_3$) δ 75.3, 74.2, 66.2, 57.5, 51.2, 49.4, 46.6, 44.5, 43.3, 42.8, 42.0, 40.8, 35.9, 27.8, 21.5, 20.8, 20.3, 19.8, 9.3 ppm; **HRMS** (CI) calculated for $C_{29}H_{31}ClO_2$ $[M]^+$ 326.2013 found 326.2020.

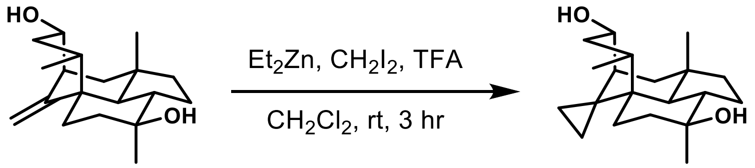

77 **S24**

To a 2-dram vial equipped with a stir bar was added CH_2Cl_2 (150 µL) and Et_2Zn (1.0 M in hexanes, 103 µL, 103 µmol). The solution was cooled to 0 °C *via* ice bath. A solution of TFA (3.9 µL, 52 µmol) in CH_2Cl_2 (150 µL) was added dropwise to the reaction vessel, and the mixture was allowed to stir at 0 °C for 10 minutes. A solution of CH_2I_2 (4.1 µL, 52 µmol) in CH_2Cl_2 (150 µL) was added dropwise to the rection vessel, and the mixture was allowed to stir at 0 °C for 20 minutes. A solution of **diol 77** (3.0 mg, 103 µmol) was added dropwise to the reaction vessel. The reaction mixture was allowed to warm to room temperature and stir for 3 hours. Upon consumption of starting material as indicated by TLC analysis, The reaction mixture was cooled to 0 °C *via* ice bath, quenched with saturated $NaHCO_{3(aq)}$ (1 mL), allowed to warm to room temperature, and extracted with EtOAc (3 x 2 mL). The organic extracts were washed with brine (1 x 1 mL), dried over $MgSO_4$, filtered, and concentrated *in vacuo*. The crude product was purified by column chromatography (SiO_2, 20% EtOAc in hexanes) affording **cyclopropane S24** (2.2 mg, 70%) as a white solid. The characterization data obtained are in good agreement with those previously reported.[10]

cyclopropane S24

^{1}H NMR (600 MHz, $CDCl_3$) δ 4.25 (appar t, *J* = 8.4 Hz, 1H), 2.19 (dt, *J* = 13.8, 6.9 Hz, 1H), 2.15-2.06 (m, 1H), 1.90-1.73 (m, 3H), 1.63-1.57 (m, 2H), 1.54-1.37 (m, 6H), 1.18 (s, 3H), 1.07-1.01 (m, 2H), 1.00 (d, *J* = 6.7 Hz, 3H), 0.97 (s, 3H), 0.66 (td, *J* = 14.0, 3.2 Hz, 1H), 0.64-0.60 (m, 1H), 0.56 (dt, *J* = 9.3, 4.7 Hz, 1H), 0.38 (dt, *J* = 9.5, 4.9 Hz, 1H), -0.16 (ddd, *J* = 9.3, 5.9, 5.0 Hz, 1H) ppm; **^{13}C NMR** (150 MHz, $CDCl_3$) δ 73.8, 72.2, 57.5, 52.4, 44.6, 43.6, 43.5, 42.4, 41.3, 40.4, 36.4, 27.5, 26.5, 25.7, 21.5, 21.3, 20.7, 20.5, 8.2, 4.3 ppm; $[\alpha]_D^{22.4}$ = +4.5 (*c* 0.50, $CHCl_3$); **FTIR** (film) ν_{max} 3347 (br), 2952, 2924 cm^{-1}; **HRMS** (CI) calculated for $C_{20}H_{32}O_2$ $[M–H_2O]^+$ 286.2297 found 286.2288.

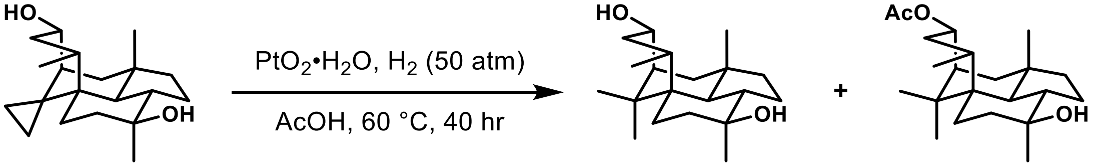

S24 *wickerol B (**2**)* *8-O-acetyl wickerol B (**85**)*

To a 2-dram vial equipped with a stir bar was added **cyclopropane S24** (2.2 mg, 7.2 µmol), PtO_2•H_2O (3.3 mg, 250 wgt%), and AcOH (1 mL). The reaction vessel was placed in a pressure reactor, which was then sealed, flushed with H_2 three times, and pressurized to 50 atm H_2. The reactor was heated to 60 °C (bath temperature) *via* oil bath. The reaction mixture was allowed to stir at 60 °C for 40 hours, allowed to cool to room temperature, then filtered through a pad of Celite rinsing with EtOAc (5 x 2 mL). The filtrate was concentrated *in vacuo*. The crude product was purified by column chromatography (SiO_2, 50% $CHCl_3$ in hexanes) affording **wickerol B** (**2**, 1.0 mg, 45%) as a white solid, and **8-*O*-acetyl wickerol B** (**85**, 0.4 mg, 18%) as a colorless oil. The characterization data obtained are in good agreement with those previously reported.[10]

wickerol B (2)

^{1}H NMR (600 MHz, $CDCl_3$) δ 4.18 (dd, *J* = 10.0, 6.3 Hz, 1H), 2.62 (dt, *J* = 16.1, 9.8 Hz, 1H), 2.07-1.97 (m, 1H), 1.90-1.70 (m, 4H), 1.67-1.58 (m, 3H), 1.49-1.37 (m, 4H), 1.29 (d, *J* = 13.0 Hz, 1H), 1.25-1.20 (m, 1H), 1.18 (s, 3H), 1.14 (s, 3H), 1.08 (d, *J* = 7.0 Hz, 1H), 0.98 (s, 3H), 0.92 (s, 3H) ppm; **^{13}C NMR** (151 MHz, $CDCl_3$) δ 73.91, 72.8, 52.6, 52.0, 44.3, 43.6, 41.9, 41.2, 41.1, 39.4, 39.3, 39.0, 26.6, 26.2, 26.1, 25.1, 22.5, 21.6, 20.6, 19.8 ppm; $[\alpha]_D^{22.0}$ = +17.6 (*c* 0.10, MeOH); **FTIR** (film) ν_{max} 3328 (br), 2957, 2926, 2881, 1463, 1383, 1103, 1014 cm^{-1}; **HRMS** (CI) calculated for $C_{20}H_{34}O_2$ $[M]^+$ 306.2559 found 306.2567.

8-*O*-acetyl wickerol B (85)

^{1}H NMR (500 MHz, $CDCl_3$) δ 5.13 (dd, *J* = 10.2, 6.1 Hz, 1H), 2.65 – 2.55 (m, 1H), 2.04 (d, *J* = 8.0 Hz, 1H), 2.01 (s, 2H), 1.88 – 1.66 (m, 4H), 1.61 (dd, *J* = 13.2, 9.6 Hz, 2H), 1.50 – 1.38 (m, 3H), 1.38 – 1.27 (m, 3H), 1.18 (s, 2H), 1.08 (d, *J* = 9.1 Hz, 5H), 1.03 (dd, *J* = 18.7, 9.7 Hz, 1H), 0.97 (d, *J* = 3.2 Hz, 4H) ppm; **^{13}C NMR** (151 MHz, $CDCl_3$) δ 170.9, 76.1, 73.9, 52.0, 48.8, 44.3, 43.6, 41.0, 40.6, 39.3, 39.0, 38.9, 37.9, 26.3, 26.2, 25.8, 24.9, 22.5, 21.8, 21.6, 20.6, 19.8 ppm; $[\alpha]_D^{22.4}$ = +20.6 (*c* 0.10, $CHCl_3$); **FTIR** (film) ν_{max} 2918, 2851, 1731 cm^{-1}; **HRMS** (CI) calculated for $C_{22}H_{36}O_3$ [M–AcOH]$^+$ 288.2453 found 288.2446.

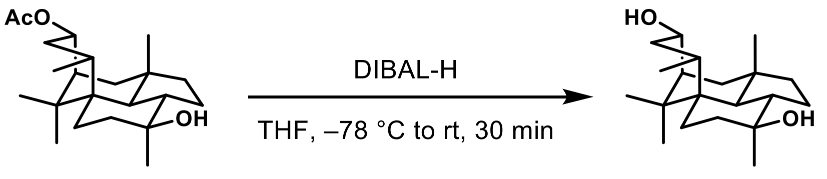

*8-O-acetyl wickerol B (**85**)*   *wickerol B (**2**)*

To a 1-dram vial equipped with a stir bar was added **8-*O*-acetyl wickerol B** (**85**, 1.7 mg, 4.8 μmol) and THF (200 μL). The solution was cooled to –78 °C *via* $Me_2CO/CO_{2(s)}$ bath. Neat DIBAL-H (20 μL, 0.11 mmol) was added dropwise to the reaction vessel. The cooling bath was removed, and the reaction allowed to stir warming to room temperature over 30 minutes. Upon consumption of starting material as indicated by TLC analysis, the reaction was quenched at –78 °C by dropwise addition of MeOH (50 μL). The quenched reaction mixture was allowed to warm to room temperature, then saturated Rochelle's salt (1 mL) and EtOAc (1 mL) were added. The resulting slurry was allowed to stir at room temperature for 1 hour. The organic layer was separated, and the aqueous layer was extracted with EtOAc (3 x 1 mL). The organic extracts were dried over Na_2SO_4, filtered, and concentrated *in vacuo*. The crude product was purified by column chromatography (SiO_2, 25% EtOAc in hexanes) affording **wickerol B** (**2**, 1.0 mg, 59%) as a white residue.

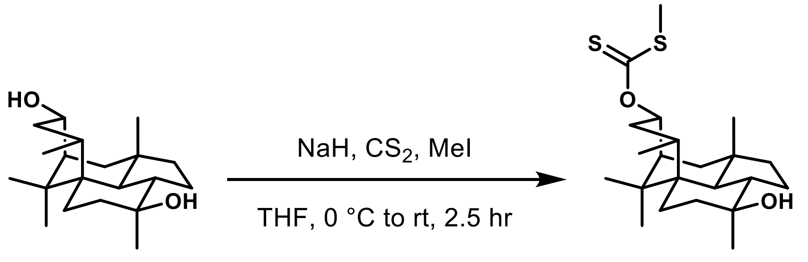

*wickerol B (**2**)*   **S25**

To a 1-dram vial equipped with a stir bar was added dry NaH (1.0 mg, 0.042 mmol) followed by THF (50 μL) The mixture was allowed to stir vigorously at room temperature until formation of a suspension, then cooled to 0 °C *via* ice bath. **Wickerol B** (**2**, 1.3 mg, 4.2 μmol) was added to the reaction vessel as a solution in THF (50 μL), rinsing with THF (2 x 50 μL). The mixture was allowed to stir at 0 °C for 30 minutes. Carbon disulfide (neat) (5.1 μL, 0.085 mmol) was added to the reaction vessel. The mixture was allowed to stir at 0 °C for 30 minutes. Methyl iodide (neat) (5.3 μL, 0.085 mmol) was added to the reaction vessel. The mixture was allowed to warm to room temperature, then allowed to stir at room temperature for 2.5 hours. Upon consumption of starting material as indicated by TLC analysis, the reaction was quenched at room temperature by dropwise addition of saturated $NH_4Cl_{(aq)}$ (0.5 mL). The quenched reaction mixture was diluted with water (0.5 mL). The aqueous layer was extracted with EtOAc (3 x 2 mL). The organic extracts were washed with brine (1 x 2 mL), dried over Na_2SO_4, filtered, and concentrated *in vacuo*. The crude product was purified by column chromatography (SiO_2, 20% EtOAc in hexanes) affording **xanthate ester S25** (0.7 mg, 42%) as a colorless residue.

xanthate ester S25

^{1}H NMR (500 MHz, $CDCl_3$) δ 5.92 (dd, *J* = 10.3, 6.1 Hz, 1H), 2.74 (dt, *J* = 16.6, 10.1 Hz, 1H), 2.53 (s, 3H), 2.11-2.05 (m, 1H), 1.92 (dd, *J* = 13.7, 2.8 Hz, 1H), 1.88-1.69 (m, 4H), 1.69-1.58 (m, 4H), 1.50-1.42 (m, 2H), 1.31 (d, *J* = 12.9 Hz, 1H), 1.24-1.20 (m, 1H), 1.19 (s, 3H), 1.12 (s, 3H), 1.11 (d, *J* = 7.1 Hz, 3H), 1.09-1.02 (m, 1H), 0.98 (s, 3H), 0.97 (s, 3H) ppm.

This material was prepared only once and was not characterized beyond ^{1}H NMR spectroscopy.

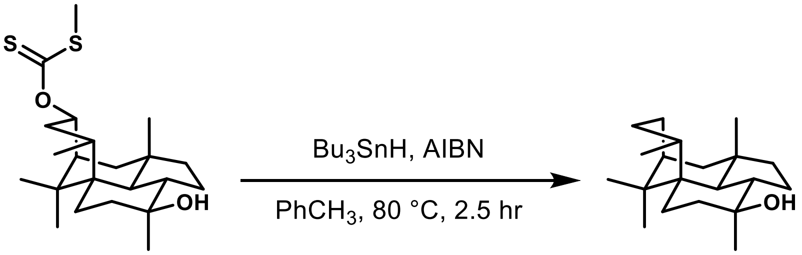

S25 *wickerol A (**1**)*

To a 1-dram vial equipped with a stir bar was added **xanthate ester S25** (1.8 mg, 4.5 µmol) and toluene (350 µL). AIBN (0.75 µg, 0.45 µmol) was added as a stock solution in toluene (50 µL). Bu_3SnH (2.0 mg, 6.8 µmol) was added as a stock solution in toluene (50 µL). The reaction mixture was heated to 80 °C *via* oil bath and allowed to stir for 2.5 hours. Upon consumption of starting material as indicated by TLC analysis, the reaction vessel was allowed to cool to room temperature, then filtered through a plug of K_2CO_3/SiO_2 (10 w/w%), rinsing with DCM.[11] The filtrate was concentrated *in vacuo*. The crude product was purified by column chromatography (SiO_2, 10% to 15% to 25% EtOAc in hexanes, stepped gradient) affording **wickerol A** (**1**) as a white solid (0.87 mg, 67%). The characterization data obtained are in good agreement with those previously reported.[10]

wickerol A (1)
^{1}H NMR (600 MHz, $CDCl_3$) δ 2.16-2.05 (m, 2H), 2.05-1.96 (m, 1H), 1.90-1.83 (m, 1H), 1.83-1.74 (m, 1H), 1.72-1.65 (m, 2H), 1.65-1.56 (m, 3H), 1.52-1.43 (m, 4H), 1.41 (d, *J* = 11.1 Hz, 1H), 1.27 (d, *J* = 13.6 Hz, H), 1.24-1.18 (m, 1H), 1.17 (s, 3H), 1.052 (s, 3H), 1.048 (s, 3H), 1.02 (d, *J* = 6.9 Hz, 3H), 1.01-0.97 (m, 1H), 0.94 (s, 3H) ppm; **^{13}C NMR** (151 MHz, $CDCl_3$) δ 74.1, 52.2, 44.5, 44.1, 43.1, 41.2, 41.0, 39.4, 38.9, 38.8, 29.0, 26.8, 26.5, 25.84, 25.76, 24.8, 23.1, 21.7, 20.6, 20.0 ppm; $[\alpha]_D^{22.4}$ **=** +10.7 (*c* 0.23, $CHCl_3$); **FTIR** (film) ν_{max} 3347, 2955, 2923, 2878, 1457, 1381, 1098 cm^{-1}.

III. References

1. Harb, H. Y.; Procter, D. J. *Synlett* **2012**, *2012* (01), 6–20.

2. Hutton, T. K.; Muir, K. W.; Procter, D. J. *Org. Lett.* **2002**, *4* (14), 2345–2347.

3. Hutton, T. K.; Muir, K. W.; Procter, D. J. *Org. Lett.* **2003**, *5* (25), 4811–4814.

4. Singh, S.; Guiry, P. J. *J. Org. Chem.* **2009**, *74* (15), 5758–5761.

5. Kerr, M. S.; Read de Alaniz, J.; Rovis, T. *J. Org. Chem.* **2005**, *70*, 5725–5728.

6. Szostak, M.; Spain, M.; Procter, D. J. *J. Org. Chem.* **2012**, *77* (7), 3049–3059.

7. Kolb, A.; Zuo, W.; Siewert, J.; Harms, K.; Von Zezschwitz, P. *Chem. A Eur. J.* **2013**, *19* (48), 16366–16373.

8. Ratnikov, M. O.; Goldmann, P. L.; McLaughlin, E. C.; Doyle, M. P. *Org. Synth.* **2012**, *89*, 19-33.

9. Wuts, P. G. M. *Synth. Commun.* **1981**, *11* (2), 139–140.

10. Deng, J.; Ning, Y.; Tian, H.; Gui, J. *J. Am. Chem. Soc.* **2020**, *142* (10), 4690–4695.

11. Harrowven, D. C.; Curran, D. P.; Kostiuk, S. L.; Wallis-Guy, I. L.; Whiting, S.; Stenning, K. J.; Tang, B.; Packard, E.; Nanson, L. *Chem. Commun.* **2010**, *46* (34), 6335–6337.

IV. Experimental Data

SFC Data

Racemic 1-methylcyclohex-2-en-1-ol

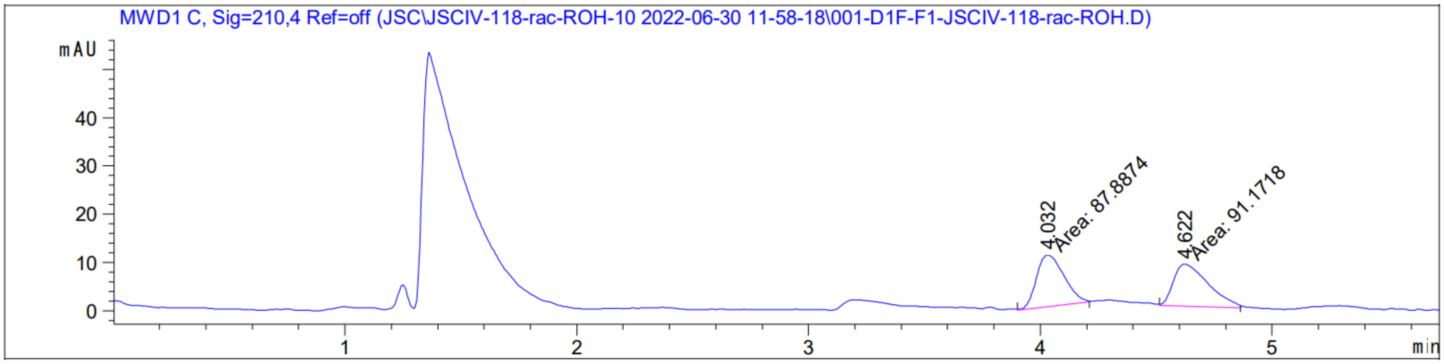

Signal 3: MWD1 C, Sig=210,4 Ref=off

Peak #	RetTime [min]	Type	Width [min]	Area [mAU*s]	Height [mAU]	Area %
1	4.032	MM	0.1371	87.88737	10.68692	49.0829
2	4.622	MM	0.1749	91.17184	8.68892	50.9171
Totals :				179.05921	19.37584	

Compound 40

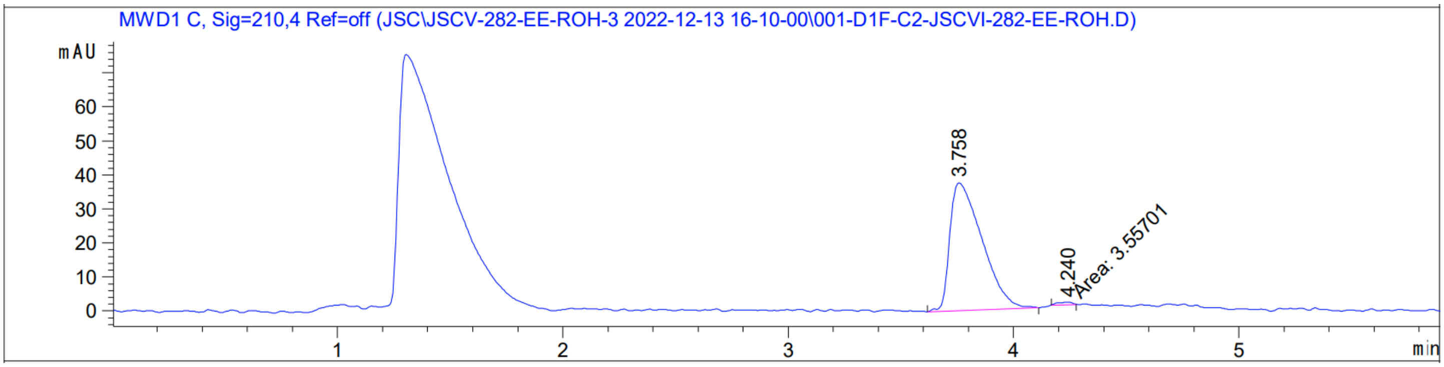

Signal 3: MWD1 C, Sig=210,4 Ref=off

Peak #	RetTime [min]	Type	Width [min]	Area [mAU*s]	Height [mAU]	Area %
1	3.758	VB R	0.1495	372.07651	37.57992	99.0531
2	4.240	MM	0.0766	3.55701	7.73441e-1	0.9469
Totals :				375.63352	38.35336	

Please note that the difference in retention times between the racemic and enantioenriched samples is due to a six-month interval between data collection.

X-ray Diffraction Data

X-ray Data Collection, Structure Solution and Refinement for **21**.

A colorless crystal of approximate dimensions 0.108 x 0.347 x 0.405 mm was mounted on a glass fiber and transferred to a Bruker SMART APEX II diffractometer system. The APEX2[1] program package was used to determine the unit-cell parameters and for data collection (20 sec/frame scan time). The raw frame data was processed using SAINT[2] and SADABS[3] to yield the reflection data file. Subsequent calculations were carried out using the SHELXTL[4] program package. The diffraction symmetry was 2/*m* and the systematic absences were consistent with the monoclinic space groups $P2_1$ and $P2_1/m$. It was later determined that space group $P2_1$ was correct.

The structure was solved by direct methods and refined on F^2 by full-matrix least-squares techniques. The analytical scattering factors[5] for neutral atoms were used throughout the analysis. Hydrogen atom H(1) was located from a difference-Fourier map and refined (x,y,z and U_{iso}). The remaining hydrogen atoms were included using a riding model. The complex was refined as a two-component twin.

Least-squares analysis yielded wR2 = 0.0683 and Goof = 1.047 for 142 variables refined against 2866 data (0.74 Å), R1 = 0.0282 for those 2813 data with $I > 2.0\sigma(I)$. The absolute structure was assigned according to the synthetic method employed. Refinement of the Flack[6] parameter was ambiguous due to the lack of an atom heavier than oxygen and the radiation (Mo) used.

References.

1. APEX2 Version 2014.11-0, Bruker AXS, Inc.; Madison, WI 2014.

2. SAINT Version 8.34a, Bruker AXS, Inc.; Madison, WI 2013.

3. Sheldrick, G. M. SADABS, Version 2014/5, Bruker AXS, Inc.; Madison, WI 2014.

4. Sheldrick, G. M. SHELXTL, Version 2014/7, Bruker AXS, Inc.; Madison, WI 2014.

5. International Tables for Crystallography 1992, Vol. C., Dordrecht: Kluwer Academic Publishers.

6. Parsons, S., Flack, H. D., Wagner, T. Acta. Cryst. B69, 249-259, 2013.

Definitions:

$wR2 = [\Sigma[w(F_o^2-F_c^2)^2] / \Sigma[w(F_o^2)^2]]^{1/2}$

$R1 = \Sigma||F_o|-|F_c|| / \Sigma|F_o|$

Goof = $S = [\Sigma[w(F_o^2-F_c^2)^2] / (n-p)]^{1/2}$ where n is the number of reflections and p is the total number of parameters refined.

The thermal ellipsoid plot is shown at the 50% probability level.

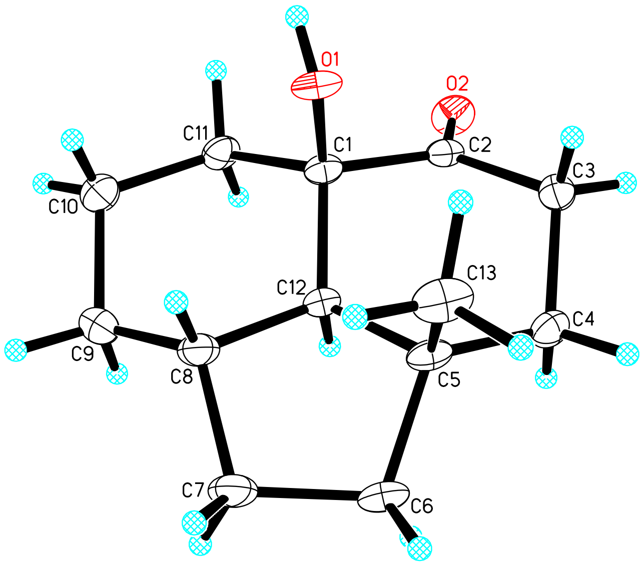

Table 1. Crystal data and structure refinement for **21**.

Identification code	cdv41 (Jonathan Chung)
Empirical formula	C_{13} H_{20} O_2
Formula weight	208.29
Temperature	93(2) K
Wavelength	0.71073 Å
Crystal system	Monoclinic
Space group	$P2_1$
Unit cell dimensions	a = 8.4288(10) Å [illegible] = 90°.
	b = 5.9423(7) Å [illegible] = 90.0207(15)°.
	c = 11.5281(13) Å [illegible] = 90°.
Volume	577.40(12) $Å^3$
Z	2
Density (calculated)	1.198 Mg/m^3
Absorption coefficient	0.079 mm^{-1}
F(000)	228
Crystal color	colorless
Crystal size	0.405 x 0.347 x 0.108 mm^3
Theta range for data collection	1.766 to 28.953°
Index ranges	$-11 \leq h \leq 11$, $-8 \leq k \leq 8$, $-15 \leq l \leq 15$
Reflections collected	12947
Independent reflections	2866 [R(int) = 0.0237]
Completeness to theta = 25.242°	100.0 %
Absorption correction	Semi-empirical from equivalents
Max. and min. transmission	0.8621 and 0.8359
Refinement method	Full-matrix least-squares on F^2
Data / restraints / parameters	2866 / 1 / 142
Goodness-of-fit on F^2	1.047
Final R indices [I>2sigma(I) = 2813 data]	R1 = 0.0282, wR2 = 0.0675
R indices (all data, 0.74 Å)	R1 = 0.0291, wR2 = 0.0683
Largest diff. peak and hole	0.176 and -0.141 e.$Å^{-3}$

Table 2. Atomic coordinates (x 10^4) and equivalent isotropic displacement parameters (Å^2 x 10^3) for cdv41. U(eq) is defined as one third of the trace of the orthogonalized U^{ij} tensor.

	x	y	z	U(eq)
O(1)	5839(2)	3236(2)	3435(1)	18(1)
O(2)	6941(2)	7081(2)	5370(1)	23(1)
C(1)	6217(2)	5587(3)	3475(2)	15(1)
C(2)	7315(2)	5974(3)	4529(2)	16(1)
C(3)	8935(2)	4873(3)	4445(2)	19(1)
C(4)	9843(2)	5560(3)	3333(2)	19(1)
C(5)	8831(2)	5076(3)	2252(2)	16(1)
C(6)	9313(2)	6292(3)	1114(2)	20(1)
C(7)	7739(2)	6629(3)	413(2)	24(1)
C(8)	6364(2)	6082(3)	1260(2)	20(1)
C(9)	4905(3)	7588(4)	1263(2)	28(1)
C(10)	3846(2)	6956(4)	2299(2)	27(1)
C(11)	4727(2)	7075(3)	3468(2)	19(1)
C(12)	7214(2)	6213(3)	2423(2)	14(1)
C(13)	8773(3)	2521(3)	2021(2)	24(1)

Table 3. Bond lengths [Å] and angles [°] for cdv41.

O(1)-C(1)	1.4340(19)
O(1)-H(1)	0.82(3)
O(2)-C(2)	1.213(2)
C(1)-C(12)	1.521(2)
C(1)-C(11)	1.536(2)
C(1)-C(2)	1.544(3)
C(2)-C(3)	1.518(2)
C(3)-C(4)	1.548(2)
C(3)-H(3A)	0.9900
C(3)-H(3B)	0.9900
C(4)-C(5)	1.537(3)
C(4)-H(4A)	0.9900
C(4)-H(4B)	0.9900
C(5)-C(12)	1.534(3)
C(5)-C(13)	1.542(2)
C(5)-C(6)	1.552(3)
C(6)-C(7)	1.566(3)
C(6)-H(6A)	0.9900
C(6)-H(6B)	0.9900
C(7)-C(8)	1.550(3)
C(7)-H(7A)	0.9900
C(7)-H(7B)	0.9900
C(8)-C(9)	1.521(3)
C(8)-C(12)	1.522(3)
C(8)-H(8A)	1.0000
C(9)-C(10)	1.537(3)
C(9)-H(9A)	0.9900
C(9)-H(9B)	0.9900
C(10)-C(11)	1.540(3)
C(10)-H(10A)	0.9900
C(10)-H(10B)	0.9900
C(11)-H(11A)	0.9900
C(11)-H(11B)	0.9900
C(12)-H(12A)	1.0000
C(13)-H(13A)	0.9800
C(13)-H(13B)	0.9800

C(13)-H(13C)	0.9800
C(1)-O(1)-H(1)	107.1(16)
O(1)-C(1)-C(12)	109.59(14)
O(1)-C(1)-C(11)	112.25(14)
C(12)-C(1)-C(11)	107.90(15)
O(1)-C(1)-C(2)	107.66(14)
C(12)-C(1)-C(2)	105.07(14)
C(11)-C(1)-C(2)	114.09(15)
O(2)-C(2)-C(3)	121.22(18)
O(2)-C(2)-C(1)	123.64(17)
C(3)-C(2)-C(1)	115.15(15)
C(2)-C(3)-C(4)	112.58(15)
C(2)-C(3)-H(3A)	109.1
C(4)-C(3)-H(3A)	109.1
C(2)-C(3)-H(3B)	109.1
C(4)-C(3)-H(3B)	109.1
H(3A)-C(3)-H(3B)	107.8
C(5)-C(4)-C(3)	110.34(15)
C(5)-C(4)-H(4A)	109.6
C(3)-C(4)-H(4A)	109.6
C(5)-C(4)-H(4B)	109.6
C(3)-C(4)-H(4B)	109.6
H(4A)-C(4)-H(4B)	108.1
C(12)-C(5)-C(4)	107.84(13)
C(12)-C(5)-C(13)	115.32(16)
C(4)-C(5)-C(13)	110.00(16)
C(12)-C(5)-C(6)	97.85(15)
C(4)-C(5)-C(6)	116.90(15)
C(13)-C(5)-C(6)	108.70(15)
C(5)-C(6)-C(7)	105.88(15)
C(5)-C(6)-H(6A)	110.6
C(7)-C(6)-H(6A)	110.6
C(5)-C(6)-H(6B)	110.6
C(7)-C(6)-H(6B)	110.6
H(6A)-C(6)-H(6B)	108.7
C(8)-C(7)-C(6)	106.34(15)
C(8)-C(7)-H(7A)	110.5

C(6)-C(7)-H(7A)	110.5
C(8)-C(7)-H(7B)	110.5
C(6)-C(7)-H(7B)	110.5
H(7A)-C(7)-H(7B)	108.7
C(9)-C(8)-C(12)	110.37(16)
C(9)-C(8)-C(7)	118.90(16)
C(12)-C(8)-C(7)	101.11(15)
C(9)-C(8)-H(8A)	108.7
C(12)-C(8)-H(8A)	108.7
C(7)-C(8)-H(8A)	108.7
C(8)-C(9)-C(10)	109.15(17)
C(8)-C(9)-H(9A)	109.9
C(10)-C(9)-H(9A)	109.9
C(8)-C(9)-H(9B)	109.9
C(10)-C(9)-H(9B)	109.9
H(9A)-C(9)-H(9B)	108.3
C(9)-C(10)-C(11)	112.87(17)
C(9)-C(10)-H(10A)	109.0
C(11)-C(10)-H(10A)	109.0
C(9)-C(10)-H(10B)	109.0
C(11)-C(10)-H(10B)	109.0
H(10A)-C(10)-H(10B)	107.8
C(1)-C(11)-C(10)	111.86(16)
C(1)-C(11)-H(11A)	109.2
C(10)-C(11)-H(11A)	109.2
C(1)-C(11)-H(11B)	109.2
C(10)-C(11)-H(11B)	109.2
H(11A)-C(11)-H(11B)	107.9
C(1)-C(12)-C(8)	115.46(15)
C(1)-C(12)-C(5)	119.06(15)
C(8)-C(12)-C(5)	106.40(15)
C(1)-C(12)-H(12A)	104.8
C(8)-C(12)-H(12A)	104.8
C(5)-C(12)-H(12A)	104.8
C(5)-C(13)-H(13A)	109.5
C(5)-C(13)-H(13B)	109.5
H(13A)-C(13)-H(13B)	109.5
C(5)-C(13)-H(13C)	109.5

H(13A)-C(13)-H(13C)	109.5
H(13B)-C(13)-H(13C)	109.5

Table 4. Anisotropic displacement parameters ($Å^2 \times 10^3$) for cdv41. The anisotropic displacement factor exponent takes the form: $-2\pi^2[h^2 a^{*2}U^{11} + ... + 2 h k a^* b^* U^{12}]$

	U^{11}	U^{22}	U^{33}	U^{23}	U^{13}	U^{12}
O(1)	21(1)	14(1)	20(1)	0(1)	9(1)	-5(1)
O(2)	23(1)	27(1)	18(1)	-7(1)	4(1)	-1(1)
C(1)	15(1)	13(1)	16(1)	-1(1)	4(1)	-1(1)
C(2)	16(1)	15(1)	17(1)	2(1)	4(1)	-2(1)
C(3)	17(1)	22(1)	17(1)	2(1)	1(1)	2(1)
C(4)	14(1)	20(1)	22(1)	-1(1)	4(1)	1(1)
C(5)	17(1)	12(1)	19(1)	-2(1)	8(1)	0(1)
C(6)	21(1)	20(1)	20(1)	-1(1)	10(1)	-4(1)
C(7)	25(1)	33(1)	15(1)	1(1)	5(1)	-9(1)
C(8)	20(1)	26(1)	14(1)	1(1)	2(1)	-7(1)
C(9)	22(1)	43(1)	21(1)	12(1)	0(1)	0(1)
C(10)	15(1)	40(1)	26(1)	9(1)	3(1)	2(1)
C(11)	13(1)	21(1)	22(1)	1(1)	5(1)	3(1)
C(12)	14(1)	13(1)	14(1)	0(1)	4(1)	-2(1)
C(13)	28(1)	14(1)	30(1)	-5(1)	12(1)	1(1)

Table 5. Hydrogen coordinates (x 10^4) and isotropic displacement parameters (Å^2 x 10^3) for cdv41.

	x	y	z	U(eq)
H(1)	5110(30)	3030(40)	3890(20)	22(6)
H(3A)	8804	3218	4455	22
H(3B)	9573	5300	5132	22
H(4A)	10102	7184	3365	22
H(4B)	10850	4709	3285	22
H(6A)	10074	5365	667	24
H(6B)	9812	7762	1288	24
H(7A)	7655	8199	133	29
H(7B)	7709	5605	-264	29
H(8A)	6018	4493	1127	24
H(9A)	4308	7393	530	34
H(9B)	5230	9184	1325	34
H(10A)	3438	5410	2185	32
H(10B)	2924	7988	2322	32
H(11A)	5036	8653	3625	22
H(11B)	4005	6583	4095	22
H(12A)	7464	7845	2529	16
H(13A)	9849	1962	1878	36
H(13B)	8108	2224	1341	36
H(13C)	8324	1752	2698	36

Table 6. Torsion angles [°] for cdv41.

O(1)-C(1)-C(2)-O(2)	-114.43(18)
C(12)-C(1)-C(2)-O(2)	128.82(19)
C(11)-C(1)-C(2)-O(2)	10.9(2)
O(1)-C(1)-C(2)-C(3)	66.01(18)
C(12)-C(1)-C(2)-C(3)	-50.74(17)
C(11)-C(1)-C(2)-C(3)	-168.71(14)
O(2)-C(2)-C(3)-C(4)	-124.6(2)
C(1)-C(2)-C(3)-C(4)	54.98(19)
C(2)-C(3)-C(4)-C(5)	-54.7(2)
C(3)-C(4)-C(5)-C(12)	53.10(18)
C(3)-C(4)-C(5)-C(13)	-73.44(18)
C(3)-C(4)-C(5)-C(6)	162.01(16)
C(12)-C(5)-C(6)-C(7)	-35.06(17)
C(4)-C(5)-C(6)-C(7)	-149.69(16)
C(13)-C(5)-C(6)-C(7)	85.1(2)
C(5)-C(6)-C(7)-C(8)	11.88(19)
C(6)-C(7)-C(8)-C(9)	137.84(19)
C(6)-C(7)-C(8)-C(12)	16.94(19)
C(12)-C(8)-C(9)-C(10)	-54.6(2)
C(7)-C(8)-C(9)-C(10)	-170.70(18)
C(8)-C(9)-C(10)-C(11)	55.9(2)
O(1)-C(1)-C(11)-C(10)	-68.1(2)
C(12)-C(1)-C(11)-C(10)	52.78(19)
C(2)-C(1)-C(11)-C(10)	169.11(15)
C(9)-C(10)-C(11)-C(1)	-56.3(2)
O(1)-C(1)-C(12)-C(8)	67.4(2)
C(11)-C(1)-C(12)-C(8)	-55.1(2)
C(2)-C(1)-C(12)-C(8)	-177.15(16)
O(1)-C(1)-C(12)-C(5)	-61.1(2)
C(11)-C(1)-C(12)-C(5)	176.45(15)
C(2)-C(1)-C(12)-C(5)	54.37(19)
C(9)-C(8)-C(12)-C(1)	57.5(2)
C(7)-C(8)-C(12)-C(1)	-175.77(15)
C(9)-C(8)-C(12)-C(5)	-168.02(14)
C(7)-C(8)-C(12)-C(5)	-41.28(18)
C(4)-C(5)-C(12)-C(1)	-57.88(19)

C(13)-C(5)-C(12)-C(1)	65.5(2)
C(6)-C(5)-C(12)-C(1)	-179.49(16)
C(4)-C(5)-C(12)-C(8)	169.58(15)
C(13)-C(5)-C(12)-C(8)	-67.1(2)
C(6)-C(5)-C(12)-C(8)	47.97(17)

Table 7. Hydrogen bonds for cdv41 [Å and °].

D-H...A	d(D-H)	d(H...A)	d(D...A)	<(DHA)
O(1)-H(1)...O(2)#1	0.82(3)	2.01(3)	2.804(2)	165(2)

Symmetry transformations used to generate equivalent atoms:

#1 -x+1,y-1/2,-z+1

X-ray Data Collection, Structure Solution and Refinement for **80**.

A colorless crystal of approximate dimensions 0.051 x 0.052 x 0.192 mm was mounted in a cryoloop and transferred to a Bruker SMART APEX II diffractometer system. The APEX3[1] program package was used to determine the unit-cell parameters and for data collection (4-10 sec/frame scan time). The raw frame data was processed using SAINT[2] and SADABS[3] to yield the reflection data file. Subsequent calculations were carried out using the SHELXTL[4] program package. Thee systematic absences were consistent with the hexagonal space groups *R*3 and $R\bar{3}$. The chiral space group *R*3 was assigned and later determined to be correct.

The structure was solved by direct methods and refined on F^2 by full-matrix least-squares techniques. The analytical scattering factors[5] for neutral atoms were used throughout the analysis. Hydrogen atoms were located from a difference-Fourier map and refined (x,y,z and U_{iso})

Least-squares analysis yielded wR2 = 0.0658 and Goof = 1.050 for 310 variables refined against 4016 data (0.83 Å), R1 = 0.0255 for those 3931 data with I > 2.0σ(I). The absolute structure was assigned by refinement of the Flack[6] parameter.

There were high residuals present in the final difference-Fourier map. It was not possible to determine the nature of the residuals although it was probable that acetonitrile solvent was present. The SQUEEZE[7a] routine in the PLATON[7b] program package was used to account for the electrons in the solvent accessible voids.

References.

7. APEX3 Version 2018.1-0, Bruker AXS, Inc.; Madison, WI 2018.

8. SAINT Version 8.38a, Bruker AXS, Inc.; Madison, WI 2013.

9. Sheldrick, G. M. SADABS, Version 2014/5, Bruker AXS, Inc.; Madison, WI 2014.

10. Sheldrick, G. M. SHELXTL, Version 2014/7, Bruker AXS, Inc.; Madison, WI 2014.

11. International Tables for Crystallography 1992, Vol. C., Dordrecht: Kluwer Academic Publishers.

12. Parsons, S., Flack, H. D., Wagner, T. Acta Cryst. B69, 249-259, 2013. **(Check CIF for correct reference)**

13. (a) Spek, A.L. SQUEEZE, Acta Cryst. 2015, C71, 9-19., (b) Spek, A. L. PLATON, Acta. Cryst. 2009, D65, 148-155

Definitions:

$wR2 = [\Sigma[w(F_o^2-F_c^2)^2] / \Sigma[w(F_o^2)^2]]^{1/2}$

$R1 = \Sigma||F_o|-|F_c|| / \Sigma|F_o|$

Goof = S = $[\Sigma[w(F_o^2-F_c^2)^2] / (n-p)]^{1/2}$ where n is the number of reflections and p is the total number of parameters refined.

The thermal ellipsoid plot is shown at the 50% probability level.

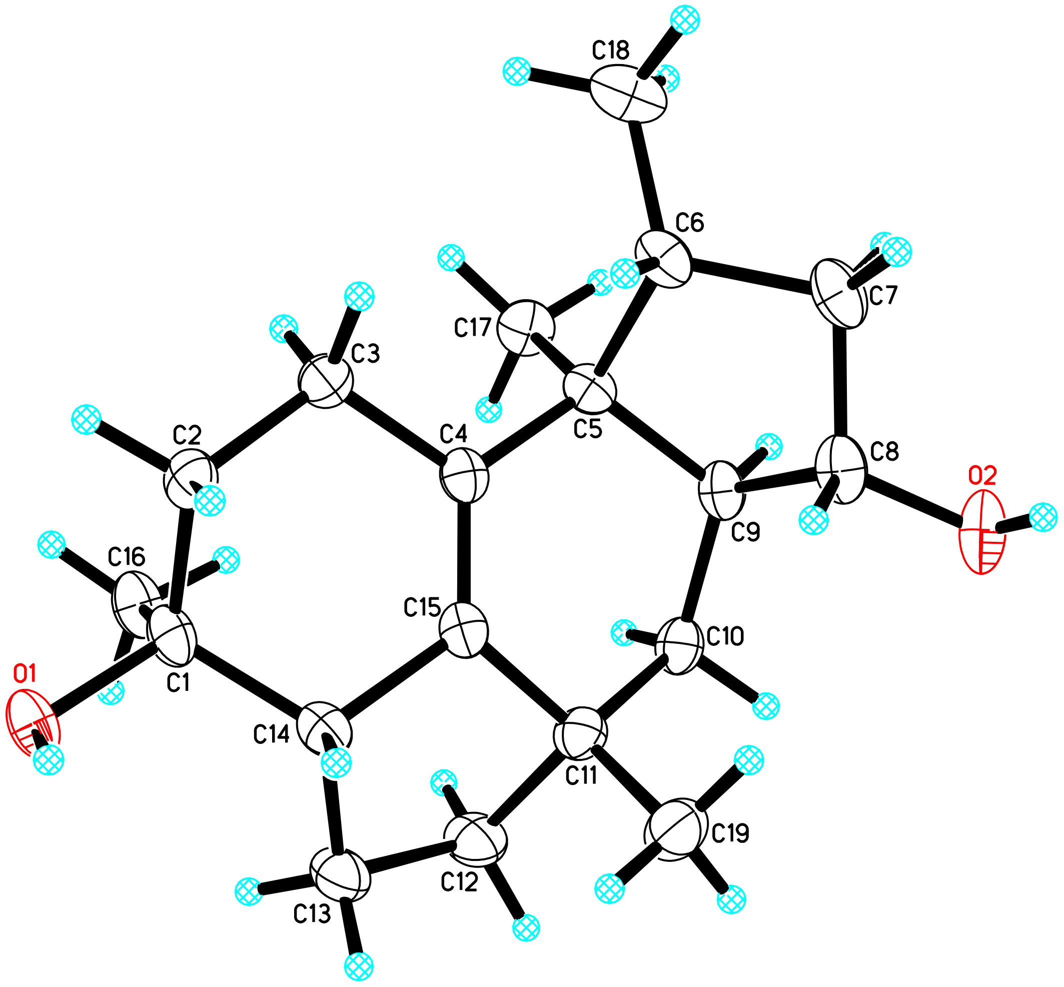

Table 1. Crystal data and structure refinement for **80**.

Identification code	cdv89 (Joseph Capani Jr.)	
Empirical formula	$C_{19} H_{30} O_2$	
Formula weight	290.43	
Temperature	93(2) K	
Wavelength	1.54178 Å	
Crystal system	Trigonal	
Space group	*R*3	
Unit cell dimensions	a = 29.9115(19) Å	⍰ = 90°.
	b = 29.9115(19) Å	⍰ = 90°.
	c = 6.2915(4) Å	⍰ = 120°.
Volume	4874.8(7) $Å^3$	
Z	9	
Density (calculated)	0.890 Mg/m^3	
Absorption coefficient	0.431 mm^{-1}	
F(000)	1440	
Crystal color	colorless	
Crystal size	0.192 x 0.052 x 0.051 mm^3	
Theta range for data collection	2.955 to 68.957°	
Index ranges	$-35 \le h \le 36$, $-36 \le k \le 36$, $-7 \le l \le 7$	
Reflections collected	25740	
Independent reflections	4016 [R(int) = 0.0406]	
Completeness to theta = 67.679°	100.0 %	
Absorption correction	Semi-empirical from equivalents	
Max. and min. transmission	0.8643 and 0.7908	
Refinement method	Full-matrix least-squares on F^2	
Data / restraints / parameters	4016 / 1 / 310	
Goodness-of-fit on F^2	1.050	
Final R indices [I>2sigma(I) = 3931 data]	R1 = 0.0255, wR2 = 0.0653	
R indices (all data, ? Å)	R1 = 0.0262, wR2 = 0.0658	
Absolute structure parameter	-0.02(8)	
Largest diff. peak and hole	0.103 and -0.123 e.$Å^{-3}$	

Table 2. Atomic coordinates (x 10^4) and equivalent isotropic displacement parameters ($Å^2$ x 10^3) for cdv89. U(eq) is defined as one third of the trace of the orthogonalized U^{ij} tensor.

	x	y	z	U(eq)
O(1)	6380(1)	6760(1)	5204(2)	25(1)
O(2)	6626(1)	9672(1)	5997(2)	32(1)
C(1)	6179(1)	7108(1)	4996(2)	23(1)
C(2)	5863(1)	7069(1)	6961(2)	24(1)
C(3)	5606(1)	7403(1)	6754(3)	25(1)
C(4)	5946(1)	7921(1)	5730(2)	20(1)
C(5)	5715(1)	8272(1)	5480(2)	21(1)
C(6)	5565(1)	8396(1)	7740(2)	24(1)
C(7)	5854(1)	8987(1)	7891(2)	28(1)
C(8)	6336(1)	9141(1)	6581(3)	25(1)
C(9)	6114(1)	8809(1)	4605(2)	22(1)
C(10)	6489(1)	8806(1)	2964(2)	24(1)
C(11)	6765(1)	8516(1)	3674(2)	24(1)
C(12)	6894(1)	8262(1)	1811(3)	30(1)
C(13)	6962(1)	7832(1)	2845(3)	29(1)
C(14)	6619(1)	7670(1)	4874(2)	23(1)
C(15)	6410(1)	8041(1)	4953(2)	20(1)
C(16)	5843(1)	6927(1)	3009(3)	28(1)
C(17)	5252(1)	8015(1)	3942(3)	27(1)
C(18)	4991(1)	8154(1)	8223(3)	37(1)
C(19)	7267(1)	8870(1)	4902(3)	33(1)

Table 3. Bond lengths [Å] and angles [°] for cdv89.

O(1)-C(1)	1.4430(17)
O(1)-H(1)	0.90(2)
O(2)-C(8)	1.4242(19)
O(2)-H(2)	0.76(3)
C(1)-C(16)	1.524(2)
C(1)-C(2)	1.526(2)
C(1)-C(14)	1.533(2)
C(2)-C(3)	1.539(2)
C(2)-H(2A)	0.97(2)
C(2)-H(2B)	1.00(2)
C(3)-C(4)	1.507(2)
C(3)-H(3A)	0.99(2)
C(3)-H(3B)	0.99(2)
C(4)-C(15)	1.341(2)
C(4)-C(5)	1.5264(19)
C(5)-C(17)	1.544(2)
C(5)-C(9)	1.546(2)
C(5)-C(6)	1.590(2)
C(6)-C(18)	1.524(2)
C(6)-C(7)	1.533(2)
C(6)-H(6A)	1.010(19)
C(7)-C(8)	1.520(2)
C(7)-H(7A)	1.02(2)
C(7)-H(7B)	0.99(2)
C(8)-C(9)	1.521(2)
C(8)-H(8A)	1.00(2)
C(9)-C(10)	1.528(2)
C(9)-H(9A)	0.986(18)
C(10)-C(11)	1.533(2)
C(10)-H(10A)	1.00(2)
C(10)-H(10B)	1.02(2)
C(11)-C(15)	1.512(2)
C(11)-C(19)	1.544(2)
C(11)-C(12)	1.546(2)
C(12)-C(13)	1.543(2)
C(12)-H(12A)	1.01(2)

C(12)-H(12B)	0.97(2)
C(13)-C(14)	1.556(2)
C(13)-H(13A)	0.95(2)
C(13)-H(13B)	0.97(2)
C(14)-C(15)	1.521(2)
C(14)-H(14A)	1.02(2)
C(16)-H(16A)	1.01(2)
C(16)-H(16B)	0.95(2)
C(16)-H(16C)	0.94(2)
C(17)-H(17A)	0.98(2)
C(17)-H(17B)	0.96(2)
C(17)-H(17C)	1.00(3)
C(18)-H(18A)	0.99(2)
C(18)-H(18B)	0.98(3)
C(18)-H(18C)	0.99(2)
C(19)-H(19A)	0.93(3)
C(19)-H(19B)	0.96(3)
C(19)-H(19C)	1.00(2)

C(1)-O(1)-H(1)	108.7(12)
C(8)-O(2)-H(2)	104.7(17)
O(1)-C(1)-C(16)	104.93(12)
O(1)-C(1)-C(2)	109.77(11)
C(16)-C(1)-C(2)	111.28(13)
O(1)-C(1)-C(14)	110.90(12)
C(16)-C(1)-C(14)	113.56(13)
C(2)-C(1)-C(14)	106.44(12)
C(1)-C(2)-C(3)	111.98(12)
C(1)-C(2)-H(2A)	109.1(13)
C(3)-C(2)-H(2A)	110.6(12)
C(1)-C(2)-H(2B)	108.1(11)
C(3)-C(2)-H(2B)	110.1(12)
H(2A)-C(2)-H(2B)	106.8(17)
C(4)-C(3)-C(2)	113.80(13)
C(4)-C(3)-H(3A)	109.5(11)
C(2)-C(3)-H(3A)	108.9(11)
C(4)-C(3)-H(3B)	109.3(12)
C(2)-C(3)-H(3B)	107.7(12)

H(3A)-C(3)-H(3B)	107.4(16)
C(15)-C(4)-C(3)	120.46(13)
C(15)-C(4)-C(5)	122.81(13)
C(3)-C(4)-C(5)	116.55(13)
C(4)-C(5)-C(17)	108.84(12)
C(4)-C(5)-C(9)	111.93(12)
C(17)-C(5)-C(9)	109.17(12)
C(4)-C(5)-C(6)	110.23(12)
C(17)-C(5)-C(6)	112.91(13)
C(9)-C(5)-C(6)	103.73(12)
C(18)-C(6)-C(7)	112.77(14)
C(18)-C(6)-C(5)	116.74(13)
C(7)-C(6)-C(5)	105.18(12)
C(18)-C(6)-H(6A)	107.6(10)
C(7)-C(6)-H(6A)	107.1(10)
C(5)-C(6)-H(6A)	107.0(10)
C(8)-C(7)-C(6)	102.66(12)
C(8)-C(7)-H(7A)	110.1(12)
C(6)-C(7)-H(7A)	110.6(12)
C(8)-C(7)-H(7B)	113.6(11)
C(6)-C(7)-H(7B)	110.8(11)
H(7A)-C(7)-H(7B)	109.0(16)
O(2)-C(8)-C(7)	115.84(13)
O(2)-C(8)-C(9)	109.90(13)
C(7)-C(8)-C(9)	101.86(13)
O(2)-C(8)-H(8A)	108.3(12)
C(7)-C(8)-H(8A)	107.9(12)
C(9)-C(8)-H(8A)	113.1(11)
C(8)-C(9)-C(10)	118.25(13)
C(8)-C(9)-C(5)	104.23(12)
C(10)-C(9)-C(5)	114.91(12)
C(8)-C(9)-H(9A)	103.2(10)
C(10)-C(9)-H(9A)	107.0(10)
C(5)-C(9)-H(9A)	108.3(10)
C(9)-C(10)-C(11)	114.27(12)
C(9)-C(10)-H(10A)	112.0(12)
C(11)-C(10)-H(10A)	108.9(12)
C(9)-C(10)-H(10B)	106.1(11)

C(11)-C(10)-H(10B)	108.5(11)
H(10A)-C(10)-H(10B)	106.7(16)
C(15)-C(11)-C(10)	111.35(12)
C(15)-C(11)-C(19)	111.35(13)
C(10)-C(11)-C(19)	111.78(13)
C(15)-C(11)-C(12)	99.92(12)
C(10)-C(11)-C(12)	113.17(13)
C(19)-C(11)-C(12)	108.70(14)
C(13)-C(12)-C(11)	104.98(13)
C(13)-C(12)-H(12A)	111.5(12)
C(11)-C(12)-H(12A)	106.3(13)
C(13)-C(12)-H(12B)	113.0(13)
C(11)-C(12)-H(12B)	111.6(13)
H(12A)-C(12)-H(12B)	109.3(17)
C(12)-C(13)-C(14)	105.35(12)
C(12)-C(13)-H(13A)	110.3(13)
C(14)-C(13)-H(13A)	108.9(12)
C(12)-C(13)-H(13B)	113.5(13)
C(14)-C(13)-H(13B)	111.1(13)
H(13A)-C(13)-H(13B)	107.7(18)
C(15)-C(14)-C(1)	110.98(12)
C(15)-C(14)-C(13)	104.27(12)
C(1)-C(14)-C(13)	116.98(13)
C(15)-C(14)-H(14A)	107.8(11)
C(1)-C(14)-H(14A)	107.5(11)
C(13)-C(14)-H(14A)	108.9(11)
C(4)-C(15)-C(11)	126.31(13)
C(4)-C(15)-C(14)	124.37(13)
C(11)-C(15)-C(14)	108.46(12)
C(1)-C(16)-H(16A)	110.8(13)
C(1)-C(16)-H(16B)	110.7(14)
H(16A)-C(16)-H(16B)	111.6(19)
C(1)-C(16)-H(16C)	111.9(13)
H(16A)-C(16)-H(16C)	109.1(19)
H(16B)-C(16)-H(16C)	102.3(19)
C(5)-C(17)-H(17A)	110.2(12)
C(5)-C(17)-H(17B)	112.2(13)
H(17A)-C(17)-H(17B)	107.7(18)

C(5)-C(17)-H(17C)	107.7(14)
H(17A)-C(17)-H(17C)	108.8(19)
H(17B)-C(17)-H(17C)	110(2)
C(6)-C(18)-H(18A)	112.5(14)
C(6)-C(18)-H(18B)	110.9(15)
H(18A)-C(18)-H(18B)	108(2)
C(6)-C(18)-H(18C)	106.7(14)
H(18A)-C(18)-H(18C)	111(2)
H(18B)-C(18)-H(18C)	108(2)
C(11)-C(19)-H(19A)	105.5(16)
C(11)-C(19)-H(19B)	111.7(14)
H(19A)-C(19)-H(19B)	110(2)
C(11)-C(19)-H(19C)	111.1(13)
H(19A)-C(19)-H(19C)	112(2)
H(19B)-C(19)-H(19C)	107.2(19)

Table 4. Anisotropic displacement parameters (Å^2 x 10^3) for cdv89. The anisotropic displacement factor exponent takes the form: -2π^2[h^2 a*^{2}U^{11} + ... + 2 h k a* b* U^{12}]

	U^{11}	U^{22}	U^{33}	U^{23}	U^{13}	U^{12}
O(1)	36(1)	26(1)	22(1)	-2(1)	-5(1)	22(1)
O(2)	49(1)	19(1)	27(1)	-4(1)	-11(1)	15(1)
C(1)	31(1)	23(1)	21(1)	1(1)	-2(1)	18(1)
C(2)	28(1)	22(1)	22(1)	5(1)	1(1)	14(1)
C(3)	28(1)	23(1)	26(1)	4(1)	7(1)	13(1)
C(4)	24(1)	20(1)	16(1)	-2(1)	-2(1)	12(1)
C(5)	23(1)	25(1)	16(1)	0(1)	-1(1)	14(1)
C(6)	30(1)	28(1)	21(1)	-2(1)	-1(1)	19(1)
C(7)	40(1)	30(1)	22(1)	-4(1)	-6(1)	24(1)
C(8)	34(1)	23(1)	23(1)	-1(1)	-8(1)	17(1)
C(9)	28(1)	20(1)	20(1)	0(1)	-5(1)	14(1)
C(10)	28(1)	20(1)	22(1)	4(1)	0(1)	12(1)
C(11)	24(1)	23(1)	25(1)	5(1)	3(1)	11(1)
C(12)	34(1)	34(1)	26(1)	8(1)	11(1)	20(1)
C(13)	33(1)	32(1)	29(1)	5(1)	10(1)	20(1)
C(14)	26(1)	25(1)	22(1)	1(1)	1(1)	16(1)
C(15)	26(1)	20(1)	16(1)	0(1)	-1(1)	13(1)
C(16)	37(1)	26(1)	25(1)	-3(1)	-6(1)	20(1)
C(17)	25(1)	30(1)	25(1)	-1(1)	-4(1)	14(1)
C(18)	35(1)	51(1)	31(1)	-4(1)	3(1)	27(1)
C(19)	25(1)	28(1)	42(1)	4(1)	-1(1)	10(1)

Table 5. Hydrogen coordinates (x 10^4) and isotropic displacement parameters ($Å^2$ x 10^3) for cdv89.

	x	y	z	U(eq)
H(1)	6607(8)	6869(7)	6290(30)	23(4)
H(2)	6722(9)	9815(9)	7040(40)	34(6)
H(2A)	6085(9)	7170(8)	8210(40)	32(5)
H(2B)	5594(8)	6700(8)	7160(30)	29(5)
H(3A)	5282(8)	7209(8)	5920(30)	23(4)
H(3B)	5509(8)	7455(8)	8200(30)	29(5)
H(6A)	5719(7)	8268(7)	8850(30)	17(4)
H(7A)	5642(8)	9131(8)	7220(30)	30(5)
H(7B)	5927(7)	9100(7)	9390(30)	26(5)
H(8A)	6566(8)	9054(7)	7420(30)	26(5)
H(9A)	5925(7)	8961(7)	3910(30)	17(4)
H(10A)	6752(9)	9162(9)	2520(30)	32(5)
H(10B)	6274(8)	8628(8)	1650(30)	28(5)
H(12A)	6585(9)	8115(8)	830(40)	34(5)
H(12B)	7197(9)	8512(8)	1040(30)	32(5)
H(13A)	7312(9)	7964(8)	3260(30)	29(5)
H(13B)	6866(8)	7540(8)	1920(40)	32(5)
H(14A)	6847(7)	7738(7)	6170(30)	21(4)
H(16A)	5719(9)	7174(9)	2630(40)	40(6)
H(16B)	5564(9)	6585(10)	3190(40)	40(6)
H(16C)	6019(9)	6892(8)	1840(40)	33(5)
H(17A)	5111(8)	8244(8)	3690(30)	28(5)
H(17B)	4978(9)	7694(9)	4490(30)	37(5)
H(17C)	5380(10)	7956(10)	2570(40)	44(6)
H(18A)	4808(9)	7772(10)	8160(40)	39(5)
H(18B)	4824(10)	8277(10)	7220(40)	48(6)
H(18C)	4963(9)	8270(10)	9660(40)	44(6)
H(19A)	7482(11)	9121(11)	3930(40)	49(7)
H(19B)	7431(9)	8684(9)	5410(40)	42(6)
H(19C)	7196(8)	9026(8)	6160(40)	33(5)

Table 6. Torsion angles [°] for cdv89.

O(1)-C(1)-C(2)-C(3)	-175.85(13)
C(16)-C(1)-C(2)-C(3)	-60.15(17)
C(14)-C(1)-C(2)-C(3)	64.06(16)
C(1)-C(2)-C(3)-C(4)	-40.08(18)
C(2)-C(3)-C(4)-C(15)	4.9(2)
C(2)-C(3)-C(4)-C(5)	-179.85(13)
C(15)-C(4)-C(5)-C(17)	110.70(16)
C(3)-C(4)-C(5)-C(17)	-64.48(16)
C(15)-C(4)-C(5)-C(9)	-10.1(2)
C(3)-C(4)-C(5)-C(9)	174.77(12)
C(15)-C(4)-C(5)-C(6)	-124.96(15)
C(3)-C(4)-C(5)-C(6)	59.86(17)
C(4)-C(5)-C(6)-C(18)	-109.94(15)
C(17)-C(5)-C(6)-C(18)	12.03(19)
C(9)-C(5)-C(6)-C(18)	130.07(15)
C(4)-C(5)-C(6)-C(7)	124.21(13)
C(17)-C(5)-C(6)-C(7)	-113.82(14)
C(9)-C(5)-C(6)-C(7)	4.23(15)
C(18)-C(6)-C(7)-C(8)	-159.23(13)
C(5)-C(6)-C(7)-C(8)	-30.96(15)
C(6)-C(7)-C(8)-O(2)	165.63(13)
C(6)-C(7)-C(8)-C(9)	46.42(14)
O(2)-C(8)-C(9)-C(10)	63.57(17)
C(7)-C(8)-C(9)-C(10)	-173.09(12)
O(2)-C(8)-C(9)-C(5)	-167.41(12)
C(7)-C(8)-C(9)-C(5)	-44.07(13)
C(4)-C(5)-C(9)-C(8)	-94.67(14)
C(17)-C(5)-C(9)-C(8)	144.77(12)
C(6)-C(5)-C(9)-C(8)	24.16(14)
C(4)-C(5)-C(9)-C(10)	36.34(17)
C(17)-C(5)-C(9)-C(10)	-84.22(15)
C(6)-C(5)-C(9)-C(10)	155.17(12)
C(8)-C(9)-C(10)-C(11)	72.12(17)
C(5)-C(9)-C(10)-C(11)	-51.75(17)
C(9)-C(10)-C(11)-C(15)	37.25(18)
C(9)-C(10)-C(11)-C(19)	-88.00(16)

C(9)-C(10)-C(11)-C(12)	148.87(13)
C(15)-C(11)-C(12)-C(13)	-39.98(16)
C(10)-C(11)-C(12)-C(13)	-158.46(14)
C(19)-C(11)-C(12)-C(13)	76.73(16)
C(11)-C(12)-C(13)-C(14)	27.72(17)
O(1)-C(1)-C(14)-C(15)	-172.42(12)
C(16)-C(1)-C(14)-C(15)	69.72(16)
C(2)-C(1)-C(14)-C(15)	-53.07(15)
O(1)-C(1)-C(14)-C(13)	68.15(16)
C(16)-C(1)-C(14)-C(13)	-49.71(18)
C(2)-C(1)-C(14)-C(13)	-172.50(13)
C(12)-C(13)-C(14)-C(15)	-3.95(17)
C(12)-C(13)-C(14)-C(1)	119.00(15)
C(3)-C(4)-C(15)-C(11)	172.65(14)
C(5)-C(4)-C(15)-C(11)	-2.3(2)
C(3)-C(4)-C(15)-C(14)	4.6(2)
C(5)-C(4)-C(15)-C(14)	-170.44(13)
C(10)-C(11)-C(15)-C(4)	-11.3(2)
C(19)-C(11)-C(15)-C(4)	114.19(17)
C(12)-C(11)-C(15)-C(4)	-131.11(16)
C(10)-C(11)-C(15)-C(14)	158.37(12)
C(19)-C(11)-C(15)-C(14)	-76.15(16)
C(12)-C(11)-C(15)-C(14)	38.55(15)
C(1)-C(14)-C(15)-C(4)	21.0(2)
C(13)-C(14)-C(15)-C(4)	147.80(15)
C(1)-C(14)-C(15)-C(11)	-148.89(12)
C(13)-C(14)-C(15)-C(11)	-22.11(16)

Table 7. Hydrogen bonds for cdv89 [Å and °].

D-H...A	d(D-H)	d(H...A)	d(D...A)	<(DHA)
O(1)-H(1)...O(1)#1	0.90(2)	1.86(2)	2.7494(12)	168.3(18)
O(2)-H(2)...O(2)#2	0.76(3)	1.89(3)	2.6416(12)	169(2)

Symmetry transformations used to generate equivalent atoms:

#1 -x+y+2/3,-x+4/3,z+1/3 #2 -y+5/3,x-y+4/3,z+1/3

*Cartesian coordinates for lowest energy conformer of **78** optimized at HF/3-21G*

C	0.712290	0.212055	-0.256925
C	-0.430796	-0.695766	1.942692
C	1.982721	-0.185822	1.971342
C	0.702918	-0.185510	2.829970
C	1.873739	0.656141	0.677644
C	-0.520378	0.137606	0.666366
C	-1.898414	-0.190947	0.067627
C	-2.754855	0.013231	1.349082
C	-1.892920	-0.627454	2.497805
C	0.462168	1.183020	3.478896
C	0.990237	-1.137926	-1.008791
C	0.431448	-1.213117	-2.470140
C	-0.702250	-0.255346	-2.920166
C	-0.924058	0.967986	-2.021420
C	-2.139493	0.823204	-1.062616
C	0.366908	1.347867	-1.267926
C	2.483367	-1.537394	-1.116979
C	1.502404	1.736241	-2.227987
C	-2.138628	-1.648858	-0.382662
H	-0.179842	-1.725937	1.726173
H	-0.638927	1.158236	0.998329
O	0.837797	-1.184877	3.876632
O	0.091022	2.532264	-0.458463
O	-0.352539	0.296263	-4.225334
H	2.197956	-1.221449	1.742517
H	2.811823	0.191463	2.566628
H	2.823325	0.624669	0.160738
H	1.686684	1.687650	0.936970
H	-3.736030	-0.440109	1.275241
H	-2.881067	1.074296	1.533130
H	-2.249449	-1.618779	2.741577
H	-1.952922	-0.032498	3.396363
H	-0.357445	1.121326	4.181148
H	0.248440	1.950483	2.749870
H	1.349835	1.483174	4.029672
H	0.511142	-1.913426	-0.430219
H	0.136822	-2.240612	-2.660430
H	1.252626	-1.007101	-3.142088
H	-1.635058	-0.792854	-3.019427
H	-1.156632	1.770562	-2.713036
H	-2.306950	1.794407	-0.614894
H	-3.020785	0.568746	-1.644917
H	2.559908	-2.473947	-1.661443
H	3.050091	-0.790061	-1.658974
H	2.942273	-1.684729	-0.150929
H	1.792925	0.935398	-2.882292

H	1.168811	2.553379	-2.860031
H	2.357370	2.073443	-1.657428
H	-3.178339	-1.748698	-0.680977
H	-1.528661	-1.954373	-1.213295
H	-1.964470	-2.341090	0.431327
H	1.535531	-0.928336	4.495387
H	-0.045996	3.296446	-1.034274
H	-0.275679	-0.412540	-4.879062

wB97X-D/6-311+G(2DF,2P) [6-311G*] = –968.0016618 hartrees

*Cartesian coordinates for lowest energy conformer of **79** optimized at HF/3-21G*

C	-0.303169	0.699692	-0.305893
C	0.476866	-0.370139	1.979281
C	-0.122397	2.020105	1.916426
C	-0.145246	0.757571	2.799546
C	-0.865284	1.855748	0.569110
C	-0.232833	-0.519327	0.633992
C	0.240343	-1.879674	0.090400
C	-0.045043	-2.736423	1.356300
C	0.418117	-1.825313	2.552399
C	-1.548678	0.474952	3.348868
C	-1.280993	0.298910	-1.458689
C	-0.769601	-0.998332	-2.121028
C	-0.626401	-2.197462	-1.138588
C	1.105421	1.047548	-0.928825
C	1.402649	0.444611	-2.343092
C	0.532822	-0.705789	-2.897949
C	-1.569783	1.406745	-2.483965
Cl	-2.987877	-0.096861	-0.788575
C	1.419864	2.560541	-1.051145
C	1.749758	-2.036813	-0.198573
O	0.218820	-0.268465	-4.251303
O	0.766811	0.948923	3.913923
H	1.511070	-0.082736	1.846259
H	-1.265971	-0.708814	0.893238
H	-1.507588	-1.290665	-2.860329
H	0.919120	2.266915	1.759784
H	-0.571237	2.846961	2.462291
H	-1.904654	1.645331	0.775551
H	-0.837768	2.794266	0.033865
H	0.474895	-3.686385	1.344090
H	-1.109101	-2.930087	1.431588
H	1.390393	-2.126394	2.916633
H	-0.275089	-1.901620	3.376046
H	-1.930358	1.364137	3.843523
H	-2.248794	0.201341	2.573298
H	-1.504941	-0.318479	4.081777
H	-1.619210	-2.467666	-0.803068
H	-0.229060	-3.045691	-1.689376

H	1.832372	0.644260	-0.239852
H	2.445334	0.150982	-2.370505
H	1.302566	1.227527	-3.078842
H	1.107447	-1.620469	-2.959087
H	-0.685230	1.677801	-3.030248
H	-1.976659	2.276359	-1.987420
H	-2.298765	1.052303	-3.199586
H	1.432439	3.062069	-0.095476
H	0.708523	3.059876	-1.696702
H	2.405476	2.675348	-1.491604
H	2.341784	-1.812734	0.679252
H	2.105586	-1.423847	-1.005606
H	1.937968	-3.073263	-0.462946
H	-0.218872	-0.975215	-4.744823
H	0.434158	1.637840	4.505659

wB97X-D/6-311+G(2DF,2P) [6-311G*] = -1353.3907037 hartrees

*Cartesian coordinates for lowest energy conformer of **80** optimized at HF/3-21G*

C	-0.721238	-0.092673	0.041788
C	0.020449	0.981721	2.205998
C	-2.386543	0.694578	1.830536
C	-1.268315	0.454157	2.844791
C	-2.168072	-0.108818	0.536749
C	0.231982	0.391465	0.816076
C	1.720335	0.318785	0.553560
C	2.245272	-0.089022	1.956065
C	1.363171	0.705305	2.959819
C	-0.450354	-0.766277	-1.312957
C	1.062911	-0.720011	-1.713996
C	2.029090	-0.735920	-0.517012
C	2.278897	1.719962	0.213861
C	-1.152256	0.051963	-2.452122
C	-0.126489	1.167309	-2.739810
C	1.233927	0.454817	-2.708699
C	-0.918723	-2.237861	-1.232921
C	-1.444910	-0.760472	-3.728736
C	-1.200042	-1.015369	3.272849
O	-1.631831	1.275824	3.983065
O	1.545349	-0.163980	-3.989598
H	-0.121295	2.053496	2.124754
H	-2.410497	1.756542	1.618351
H	-3.333766	0.429110	2.284172
H	-2.833142	0.274694	-0.226820
H	-2.460326	-1.138904	0.709639
H	3.301945	0.117857	2.077401
H	2.082853	-1.151538	2.097406
H	1.838731	1.639265	3.228561
H	1.216504	0.141647	3.871400
H	1.273492	-1.597448	-2.309323

H	3.049327	-0.631283	-0.873838
H	1.952667	-1.713822	-0.052041
H	1.781343	2.151246	-0.641850
H	3.342543	1.657359	0.005335
H	2.140461	2.397371	1.047319
H	-2.081839	0.474623	-2.099160
H	-0.161554	1.901772	-1.944851
H	-0.306990	1.670701	-3.683025
H	2.042944	1.116286	-2.438604
H	-1.991460	-2.312481	-1.106193
H	-0.440772	-2.729035	-0.391853
H	-0.650234	-2.772245	-2.134487
H	-1.893196	-0.105528	-4.470612
H	-0.531483	-1.158241	-4.142144
H	-2.145003	-1.564634	-3.538495
H	-0.497135	-1.150953	4.087005
H	-0.894509	-1.648558	2.452603
H	-2.176116	-1.328126	3.624244
H	-0.973727	1.186321	4.685800
H	1.617623	0.509025	-4.679999

wB97X-D/6-311+G(2DF,2P) [6-311G*] = –892.2940182 hartrees

*Cartesian coordinates for lowest energy conformer of **81** optimized at HF/3-21G*

C	0.657626	0.200745	-0.377015
C	-0.421871	-0.701693	1.817323
C	1.686787	0.611416	1.986348
C	0.374327	0.248952	2.716505
C	1.513606	1.129147	0.532682
C	-0.608869	-0.048906	0.447397
C	-1.862685	-0.686112	-0.178845
C	-2.792368	-0.617313	1.066944
C	-1.879927	-1.055800	2.257801
C	-0.378197	1.510421	3.161757
C	-1.759101	-2.171036	-0.592050
C	-2.317367	0.186728	-1.382864
C	-1.306649	1.293303	-1.788875
C	0.195506	0.914303	-1.726268
C	1.416523	-1.110083	-0.857345
C	1.656021	-0.912402	-2.369020
C	0.406978	-0.172851	-2.822906
C	2.729292	-1.525750	-0.167524
O	0.696607	-0.540185	3.892940
O	0.421238	0.346848	-4.160293
Cl	-1.684217	2.834168	-0.830181
C	1.049836	2.136501	-2.115848
H	-0.959027	0.946941	0.673688
H	0.167246	-1.607059	1.749408
H	2.300501	-0.273192	2.008777
H	2.208921	1.376237	2.557396

H	1.022651	2.092359	0.578428
H	2.495528	1.296465	0.107744
H	-3.668165	-1.247164	0.966448
H	-3.120723	0.405852	1.210864
H	-1.965662	-2.120989	2.425089
H	-2.160755	-0.564100	3.176308
H	0.271776	2.105180	3.798007
H	-1.252756	1.246409	3.739346
H	-0.678437	2.129758	2.329591
H	-2.756422	-2.543355	-0.806595
H	-1.343122	-2.777269	0.202681
H	-1.163405	-2.319105	-1.480943
H	-2.469887	-0.438646	-2.254868
H	-3.266872	0.661730	-1.176078
H	-1.507208	1.592436	-2.805608
H	0.730277	-1.933863	-0.745904
H	1.789826	-1.853430	-2.888237
H	2.540895	-0.303898	-2.531999
H	-0.418127	-0.862606	-2.815511
H	3.176587	-2.335269	-0.736693
H	2.566243	-1.890993	0.835987
H	3.443694	-0.713041	-0.134662
H	1.171682	-0.000964	4.540122
H	1.276976	0.742365	-4.368070
H	0.713590	2.515855	-3.071836
H	0.964196	2.933296	-1.396162
H	2.096888	1.869660	-2.197382

wB97X-D/6-311+G(2DF,2P) [6-311G*] = –1353.0854026 hartrees

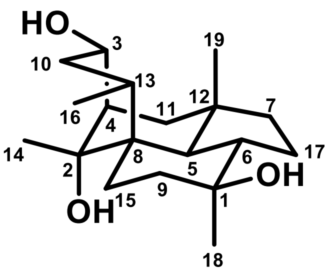

Table S1. Isolated experimental and calculated ^{13}C chemical shifts for **78**. Carbons are numbered according to structural assignment performed *via* 2D-NMR analysis.

Carbon	Isolated Exp.	ωB97X-D/6-31G*	Δ	|Δ|
1	73.9	76.4	2.5	2.5
2	73.8	78.5	4.7	4.7
3	72.2	74.8	2.6	2.6
4	52.3	50.0	-2.3	2.3
5	50.2	48.3	-1.9	1.9
6	43.4	43.3	-0.1	0.1
7	41.2	42.1	0.9	0.9
8	41.1	41.6	0.5	0.5
9	41.1	39.2	-1.9	1.9
10	40.8	39.7	-1.1	1.1
11	40.7	37.9	-2.8	2.8
12	38.9	39.2	0.3	0.3
13	27.1	27.7	0.6	0.6
14	25.8	24.5	-1.3	1.3
15	24.8	25.1	0.3	0.3
16	21.9	20.7	-1.2	1.2
17	21.6	22.9	1.3	1.3
18	20.6	19.1	-1.5	1.5
19	19.8	18.0	-1.8	1.8

AVERAGE |Δ|: 1.6
MAX |Δ|: 4.7

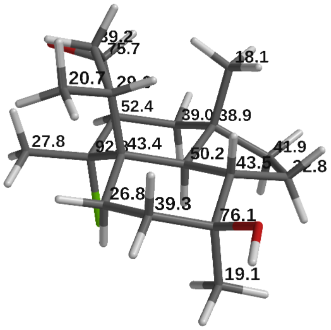

Table S2. Isolated experimental and calculated ^{13}C chemical shifts for **79**. Carbons are numbered according to decreasing chemical shift.

Carbon	Isolated Exp.	ωB97X-D/6-31G*	Δ	|Δ|
1	86.2	92.8	6.6	6.6
2	73.9	76.1	2.2	2.2
3	72.5	75.7	3.2	3.2
4	54.7	52.4	-2.3	2.3
5	52.0	50.2	-1.8	1.8
6	44.2	43.5	-0.7	0.7
7	43.3	43.4	0.1	0.1
8	42.5	41.9	-0.6	0.6
9	42.0	39.3	-2.7	2.7
10	41.0	39.2	-1.8	1.8
11	40.6	39.0	-1.6	1.6
12	38.9	38.9	0.0	0.0
13	28.5	29.0	0.5	0.5
14	27.8	27.8	0.0	0.0
15	27.0	26.8	-0.2	0.2
16	22.2	22.8	0.6	0.6
17	21.6	20.7	-0.9	0.9
18	20.6	19.1	-1.5	1.5
19	19.5	18.1	-1.4	1.4

AVERAGE |Δ|: 1.5
MAX |Δ|: 6.6

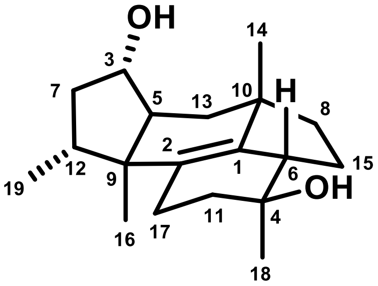

Table S3. Isolated experimental and calculated ^{13}C chemical shifts for **80**. Carbons are numbered according to structural assignment performed *via* 2D-NMR analysis.

Carbon	Isolated Exp.	ωB97X-D/6-31G*	Δ	|Δ|
1	138.2	140.9	2.7	2.7
2	131.4	128.8	-2.6	2.6
3	73.3	80.8	7.5	7.5
4	72.3	74.2	1.9	1.9
5	56.1	55.0	-1.1	1.1
6	47.9	48.2	0.3	0.3
7	43.8	39.6	-4.2	4.2
8	43.6	41.1	-2.5	2.5
9	42.9	47.5	4.6	4.6
10	40.6	41.2	0.6	0.6
11	38.8	37.1	-1.7	1.7
12	38.1	41.0	2.9	2.9
13	33.7	35.1	1.4	1.4
14	25.8	25.0	-0.8	0.8
15	24.1	23.3	-0.8	0.8
16	22.4	22.9	0.5	0.5
17	21.9	25.3	3.5	3.5
18	19.5	19.3	-0.2	0.2
19	17.6	16.0	-1.6	1.6

AVERAGE |Δ|: 2.2
MAX |Δ|: 7.5

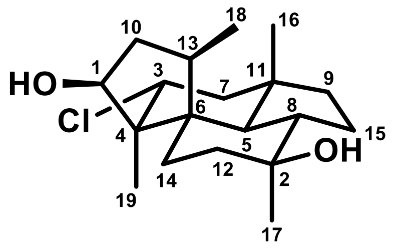

Table S4. Isolated experimental and calculated ^{13}C chemical shifts for **81**. Carbons are numbered according to structural assignment performed *via* 2D-NMR analysis.

**Carbon**	**Isolated Exp.**	**ωB97X-D/6-31G***	**Δ**	**|Δ|**
1	75.3	76.0	0.7	0.7
2	74.2	77.2	3.0	3.0
3	66.2	71.6	5.4	5.4
4	57.5	54.5	-3.0	3.0
5	51.2	48.6	-2.6	2.6
6	49.4	45.2	-4.2	4.2
7	46.6	40.4	-6.2	6.2
8	44.5	42.6	-1.9	1.9
9	43.3	43.7	0.4	0.4
10	42.8	39.7	-3.1	3.1
11	42.0	38.5	-3.5	3.5
12	40.8	38.6	-2.2	2.2
13	35.9	32.8	-3.1	3.1
14	27.8	30.4	2.6	2.6
15	21.5	23.2	1.7	1.7
16	20.8	22.9	2.1	2.1
17	20.3	19.6	-0.7	0.7
18	19.8	17.4	-2.4	2.4
19	9.3	15.6	6.3	6.3

AVERAGE |Δ|: 2.9
MAX |Δ|: 6.3

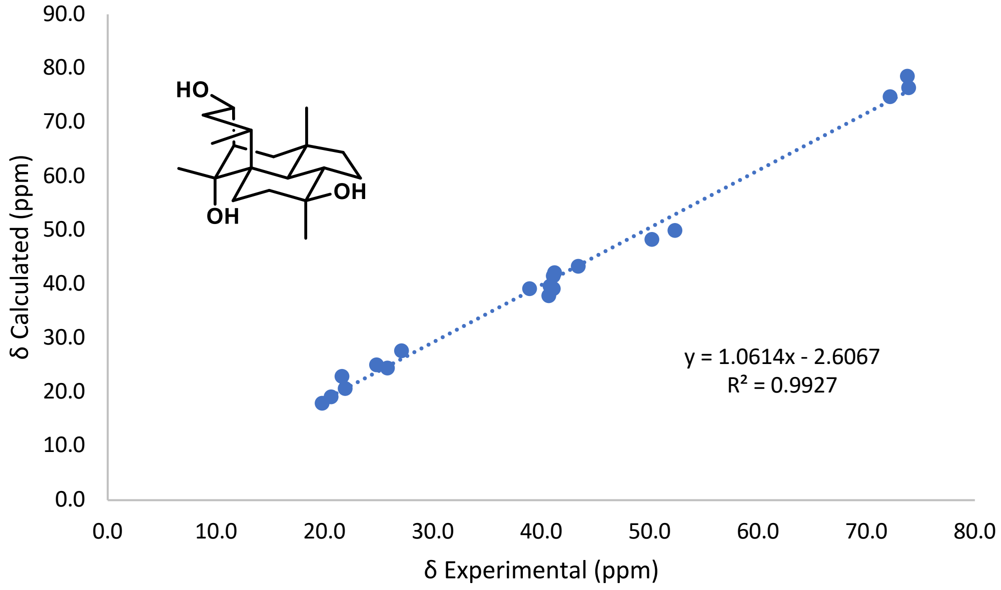

Figure S3. Linear correlation plot for **78** based on data from Table **S1**.

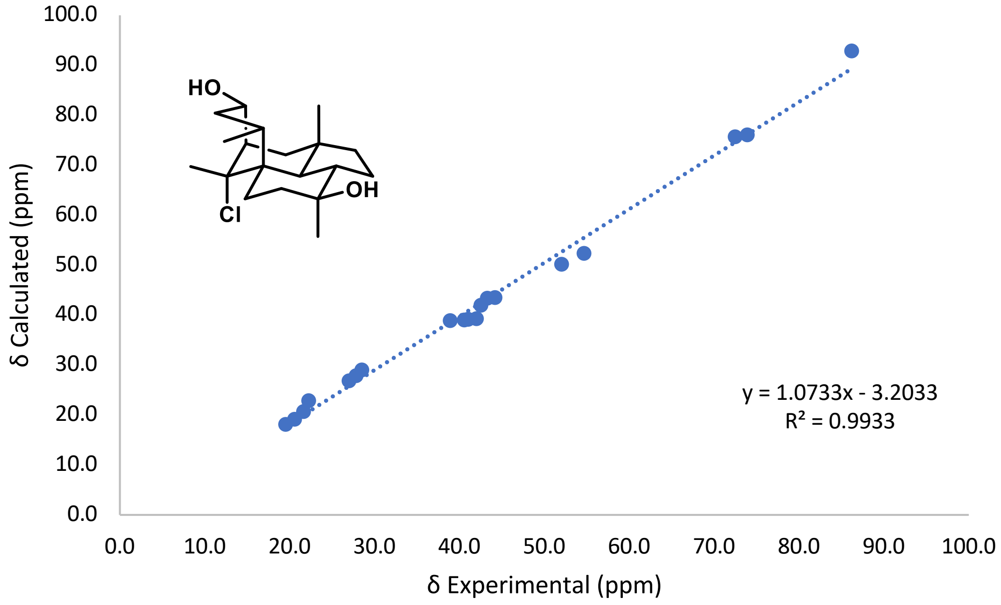

Figure S4. Linear correlation plot for **79** based on data from Table **S2**.

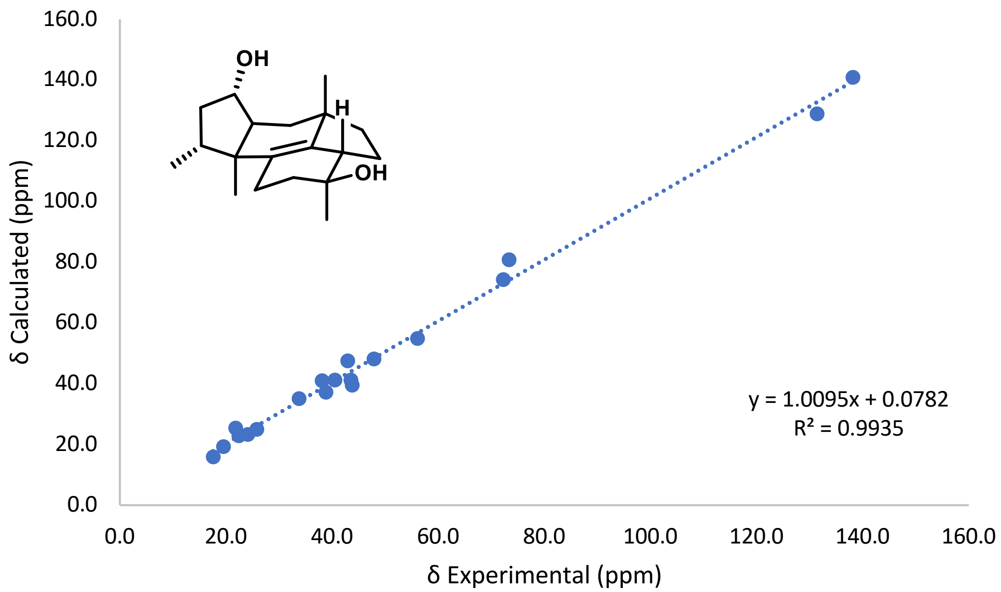

Figure S5. Linear correlation plot for **80** based on data from Table **S3**.

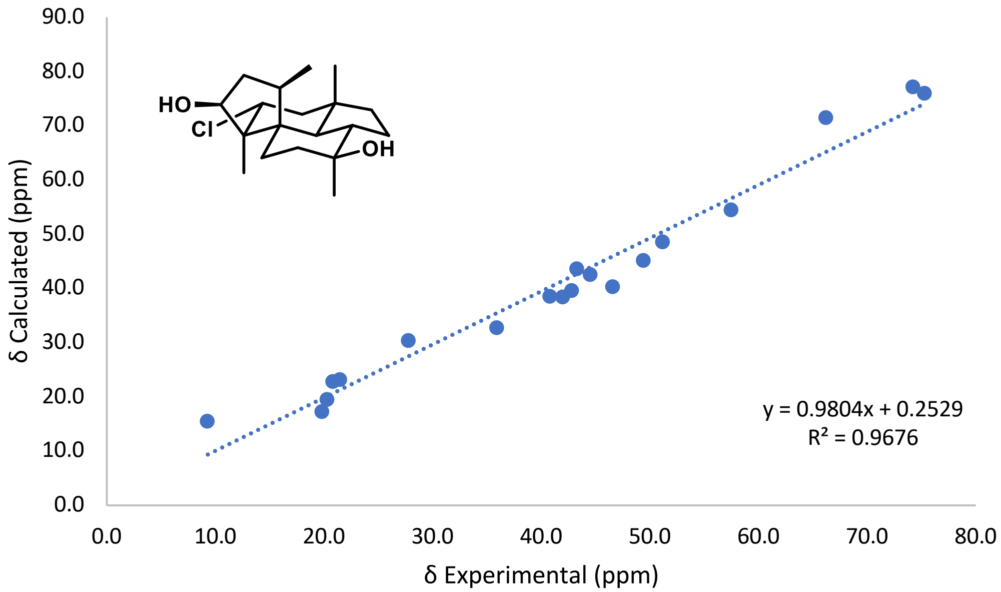

Figure S6. Linear correlation plot for **81** based on data from Table **S4**.

```
Current Data Parameters
NAME      MG 1-1 dest Fr. 4
EXPNO                     1
PROCNO                    1

F2 - Acquisition Parameters
Date_              20170310
Time                  19.16
INSTRUM             cryo500
PROBHD    5 mm CPTCI 1H-
PULPROG                zg30
TD                    32048
SOLVENT               CDCl3
NS                        8
DS                        0
SWH                8012.820 Hz
FIDRES             0.250026 Hz
AQ                1.9997952 sec
RG                      3.6
DW                   62.400 usec
DE                     6.00 usec
TE                    298.0 K
D1               0.10000000 sec
MCREST                    0 sec
MCWRK            0.01500000 sec

======== CHANNEL f1 ========
NUC1                     1H
P1                     7.50 usec
PL1                    1.60 dB
SFO1            500.2235015 MHz

F2 - Processing parameters
SI                    65536
SF              500.2200317 MHz
WDW                      EM
SSB                       0
LB                     0.30 Hz
GB                        0
PC                     1.00
```

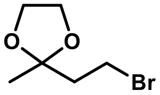

S4

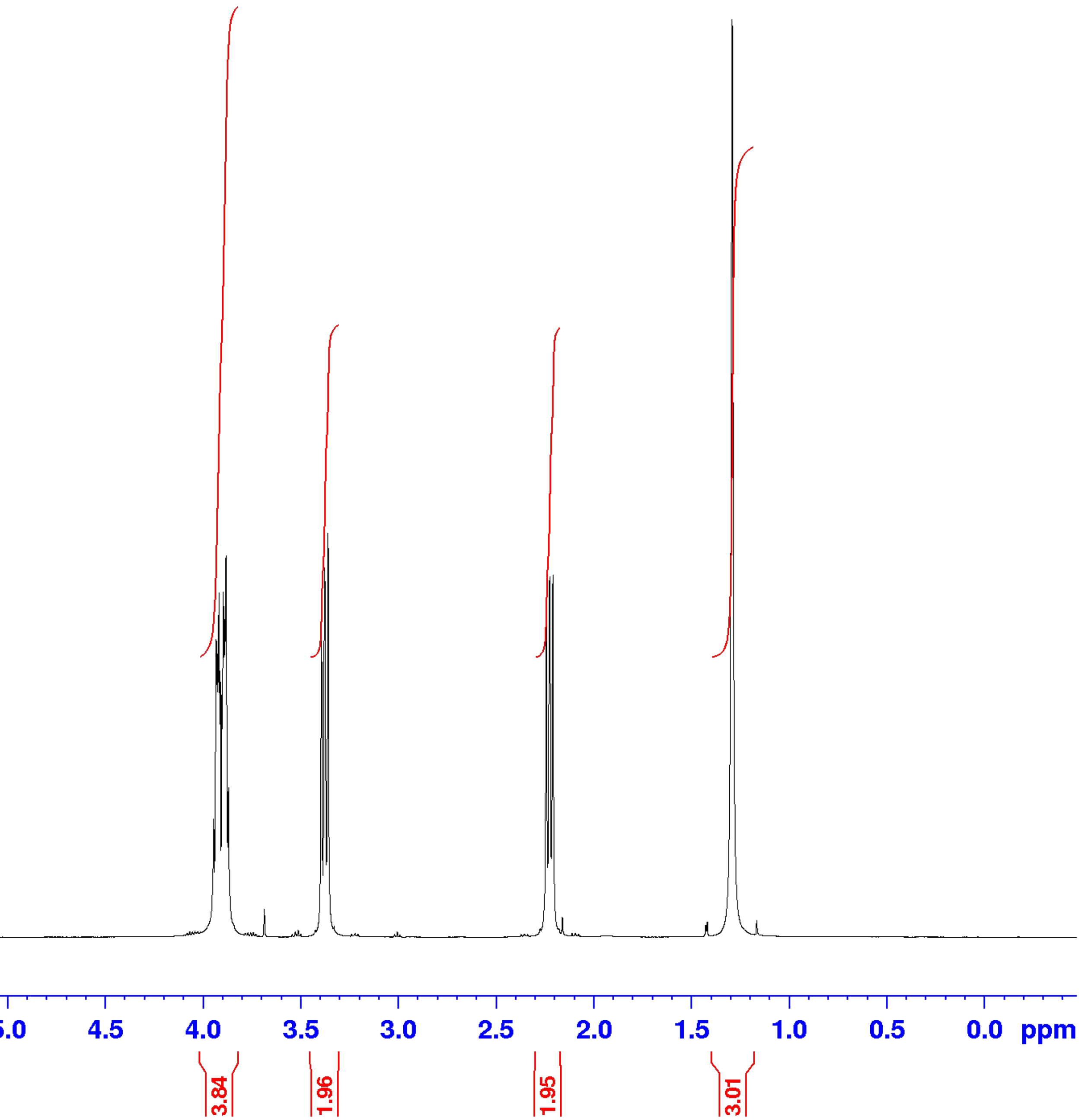

```
Current Data Parameters
NAME      MG 1-1 dest Fr. 4
EXPNO                   2
PROCNO                  1

F2 - Acquisition Parameters
Date_           20170310
Time               19.20
INSTRUM          cryo500
PROBHD   5 mm CPTCI 1H-
PULPROG       deptqgpsp
TD                 65536
SOLVENT            CDCl3
NS                   329
DS                     8
SWH            30303.031 Hz
FIDRES          0.462388 Hz
AQ             1.0813440 sec
RG                  2048
DW                16.500 usec
DE                  6.00 usec
TE                 298.0 K
CNST2        145.0000000
CNST12         1.5000000
D1            1.00000000 sec
d2            0.00344828 sec
d12           0.00002000 sec
D16           0.00020000 sec
DELTA         0.00001707 sec
DELTA1        0.00228023 sec
DELTA2        0.00226483 sec
DELTA3        0.00224828 sec
MCREST                 0 sec
MCWRK         0.01500000 sec

======== CHANNEL f1 ========
NUC1                 13C
P1                 16.55 usec
P12              2000.00 usec
PL0               120.00 dB
PL1                -1.00 dB
SFO1         125.7942548 MHz
SP2                 2.70 dB
SPNAM[2]     Crp60comp.4
SPOFF2                 0 Hz

======== CHANNEL f2 ========
CPDPRG[2         waltz16
NUC2                  1H
p0                 11.55 usec
P3                  7.70 usec
p4                 15.40 usec
PCPD2             100.00 usec
PL2                 1.60 dB
PL12               24.50 dB
SFO2         500.2225011 MHz

====== GRADIENT CHANNEL =====
GPNAM[1]        SINE.100
GPNAM[2]        SINE.100
GPNAM[3]        SINE.100
GPX1                   0 %
GPX2                   0 %
GPX3                   0 %
GPY1                   0 %
GPY2                   0 %
GPY3                   0 %
GPZ1               31.00 %
GPZ2               31.00 %
GPZ3               31.00 %
P16              1000.00 usec

F2 - Processing parameters
SI                 65536
SF           125.7804165 MHz
WDW                   EM
SSB                    0
LB                  1.00 Hz
GB                     0
PC                  2.00
```

S4

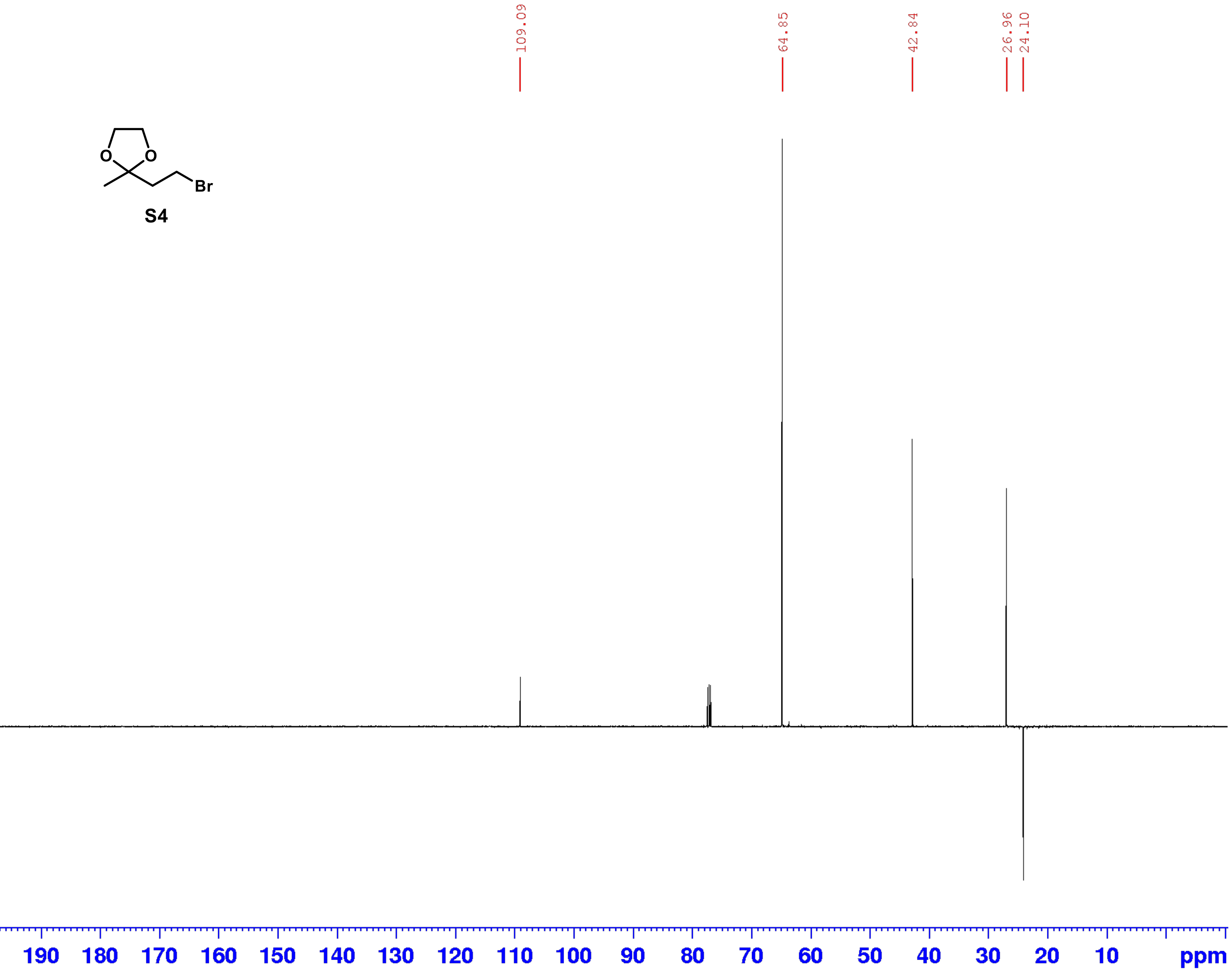

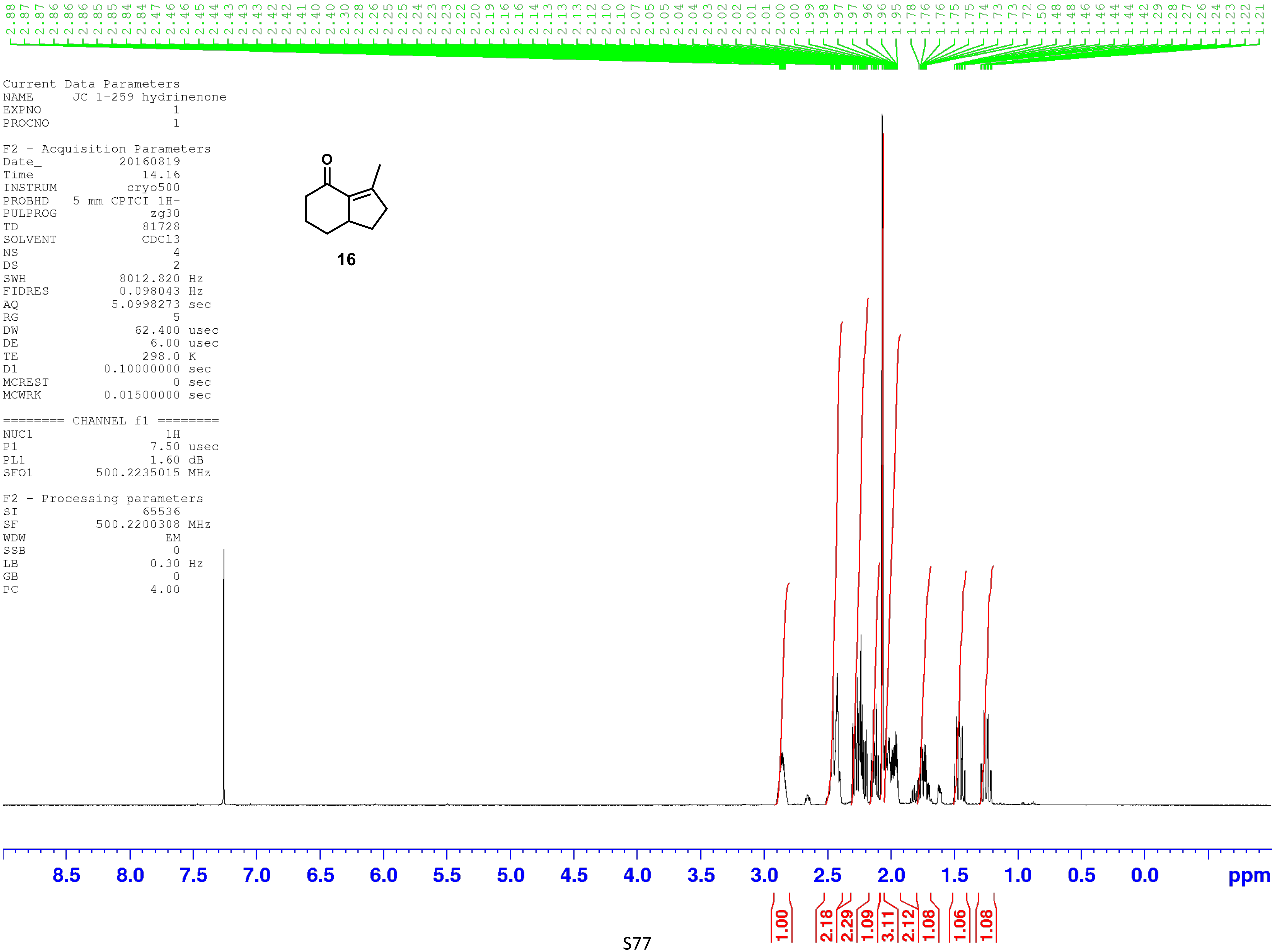
2.88 2.87 2.87 2.86 2.86 2.86 2.85 2.85 2.84 2.84 2.47 2.46 2.46 2.45 2.44 2.43 2.43 2.43 2.42 2.42 2.41 2.40 2.40 2.30 2.28 2.26 2.25 2.25 2.24 2.23 2.23 2.22 2.20 2.19 2.16 2.16 2.14 2.13 2.13 2.12 2.10 2.10 2.07 2.05 2.04 2.04 2.03 2.02 2.02 2.01 2.01 2.00 2.00 1.99 1.98 1.97 1.97 1.96 1.96 1.95 1.78 1.76 1.76 1.75 1.75 1.74 1.73 1.73 1.72 1.50 1.48 1.48 1.46 1.46 1.44 1.44 1.42 1.29 1.28 1.27 1.26 1.24 1.23 1.22 1.21
Current Data Parameters
NAME JC 1-259 hydrinenone
EXPNO 1
PROCNO 1
F2 - Acquisition Parameters
Date_ 20160819
Time 14.16
INSTRUM cryo500
PROBHD 5 mm CPTCI 1H-
PULPROG zg30
TD 81728
SOLVENT CDCl3
NS 4
DS 2
SWH 8012.820 Hz
FIDRES 0.098043 Hz
AQ 5.0998273 sec
RG 5
DW 62.400 usec
DE 6.00 usec
TE 298.0 K
D1 0.10000000 sec
MCREST 0 sec
MCWRK 0.01500000 sec
======== CHANNEL f1 ========
NUC1 1H
P1 7.50 usec
PL1 1.60 dB
SFO1 500.2235015 MHz
F2 - Processing parameters
SI 65536
SF 500.2200308 MHz
WDW EM
SSB 0
LB 0.30 Hz
GB 0
PC 4.00
8.5 8.0 7.5 7.0 6.5 6.0 5.5 5.0 4.5 4.0 3.5 3.0 2.5 2.0 1.5 1.0 0.5 0.0 ppm
1.00 2.18 2.29 1.09 3.11 2.12 1.08 1.06 1.08

16

```
Current Data Parameters
NAME     JC 1-259 hydrinenone
EXPNO                 2
PROCNO                1

F2 - Acquisition Parameters
Date_          20160819
Time              14.17
INSTRUM         cryo500
PROBHD   5 mm CPTCI 1H-
PULPROG  SpinEchopg30gp.prd
TD                65536
SOLVENT           CDCl3
NS                   35
DS                   16
SWH           30303.031 Hz
FIDRES         0.462388 Hz
AQ            1.0813440 sec
RG               7298.2
DW               16.500 usec
DE                 6.00 usec
TE                298.0 K
D1           0.25000000 sec
d11          0.03000000 sec
D16          0.00020000 sec
d17          0.00019600 sec
MCREST                0 sec
MCWRK        0.01500000 sec
P2                33.10 usec

======== CHANNEL f1 ========
NUC1                13C
P1                16.55 usec
P11              500.00 usec
P12             2000.00 usec
PL0              120.00 dB
PL1               -1.00 dB
SFO1        125.7942548 MHz
SP1                2.70 dB
SP2                2.70 dB
SPNAM[1] Crp60,0.5,20.1
SPNAM[2]     Crp60comp.4
SPOFF1                0 Hz
SPOFF2                0 Hz

======== CHANNEL f2 ========
CPDPRG[2         waltz16
NUC2                 1H
PCPD2            100.00 usec
PL2                1.60 dB
PL12              24.50 dB
SFO2        500.2225011 MHz

====== GRADIENT CHANNEL =====
GPNAM[1]       SINE.100
GPNAM[2]       SINE.100
GPX1                  0 %
GPX2                  0 %
GPY1                  0 %
GPY2                  0 %
GPZ1              30.00 %
GPZ2              50.00 %
p15              500.00 usec
p16             1000.00 usec

F2 - Processing parameters
SI                65536
SF          125.7804080 MHz
WDW                  EM
SSB                   0
LB                 1.00 Hz
GB                    0
PC                 2.00
```

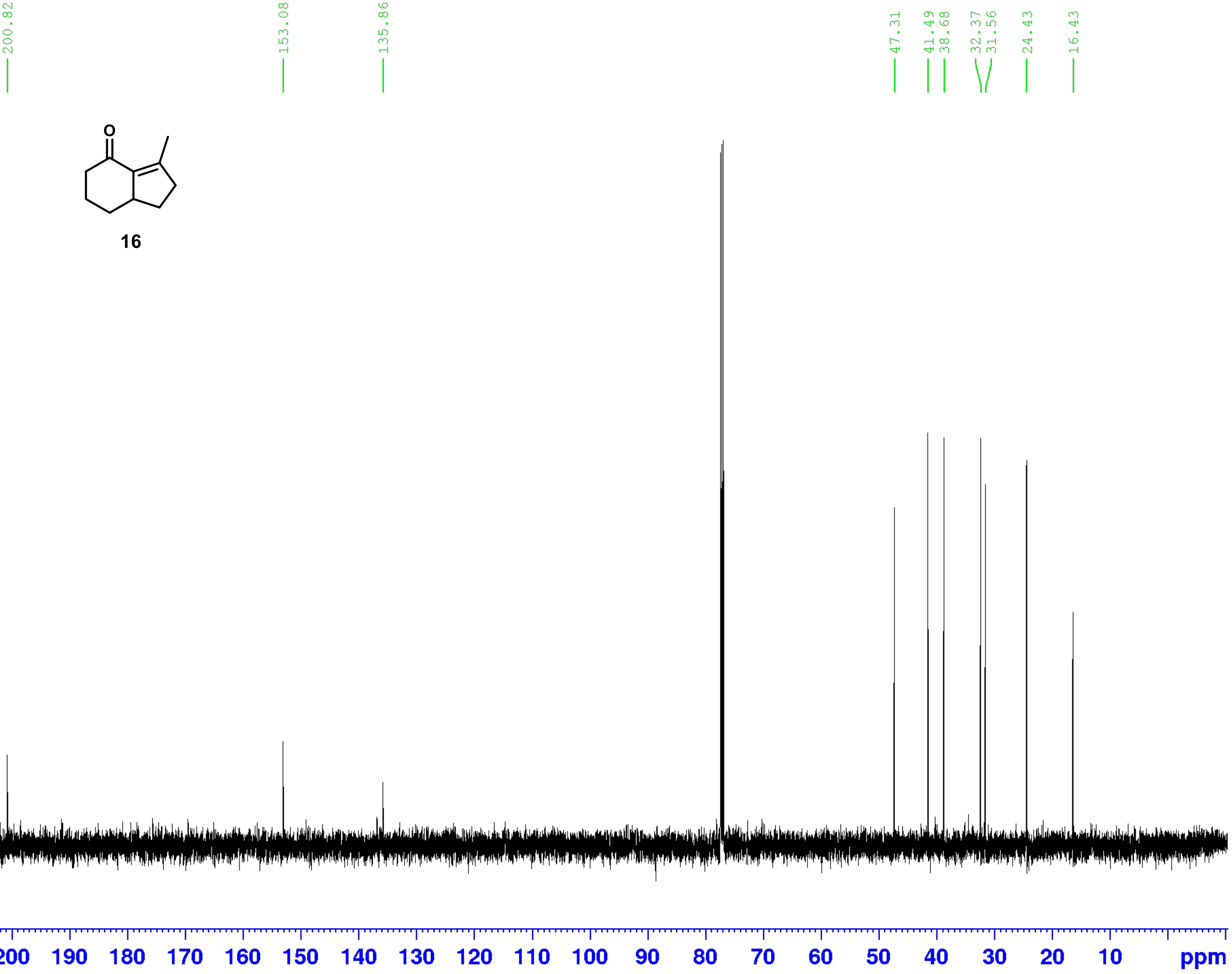

```
Current Data Parameters
NAME       JC 1-141 CuI butene
EXPNO                    1
PROCNO                   1

F2 - Acquisition Parameters
Date_             20160525
Time                 22.18
INSTRUM              gn500
PROBHD    5 mm broadband
PULPROG               zg30
TD                   81728
SOLVENT              CDCl3
NS                       8
DS                       2
SWH               8012.820 Hz
FIDRES            0.098043 Hz
AQ               5.0998273 sec
RG                   574.7
DW                  62.400 usec
DE                    6.00 usec
TE                   298.2 K
D1              0.10000000 sec
MCREST                   0 sec
MCWRK           0.01500000 sec

======== CHANNEL f1 ========
NUC1                    1H
P1                   12.00 usec
PL1                  -5.80 dB
SFO1           499.0734935 MHz

F2 - Processing parameters
SI                   65536
SF             499.0700314 MHz
WDW                     EM
SSB                      0
LB                    0.30 Hz
GB                       0
PC                    1.00
```

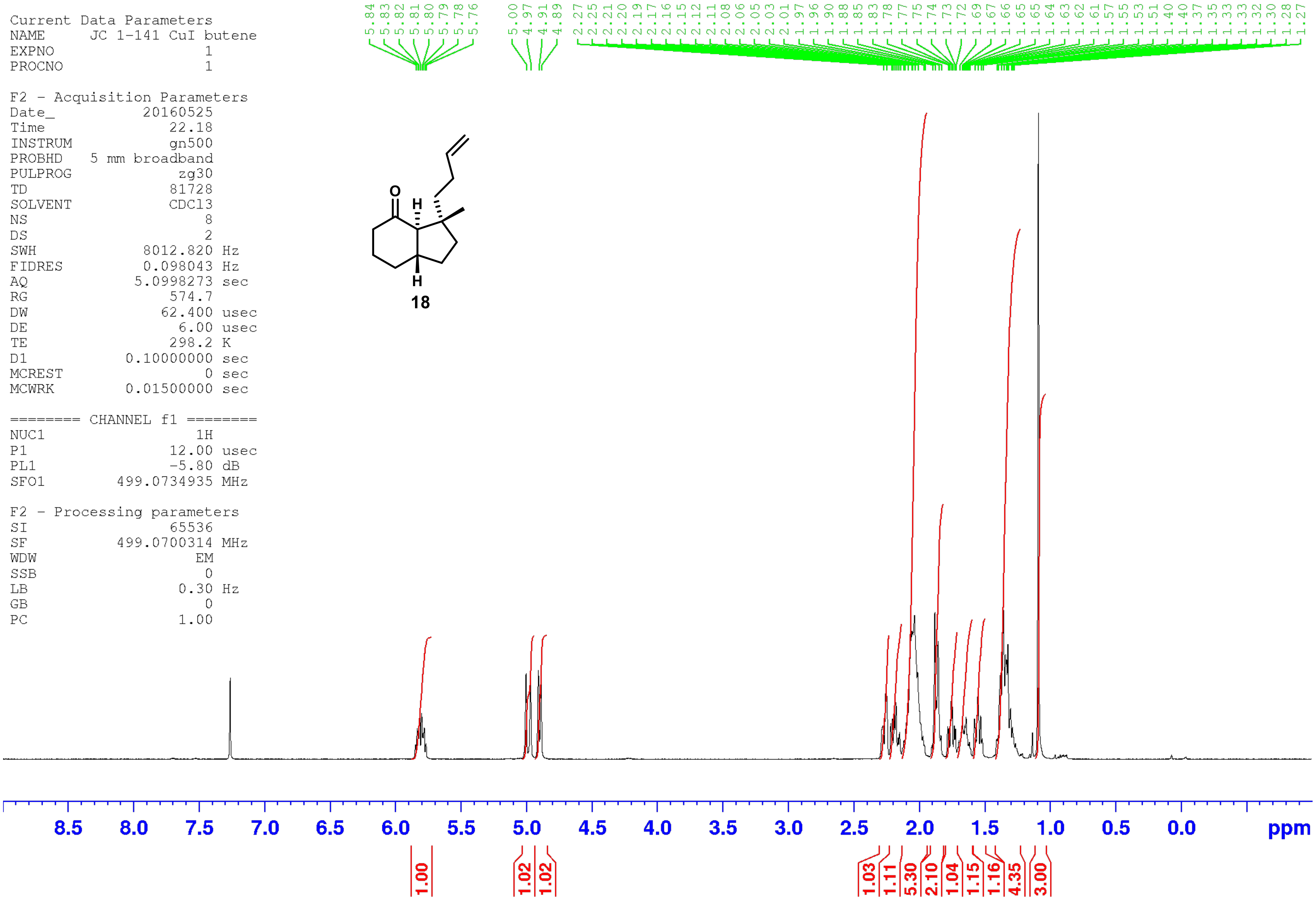

Current Data Parameters
NAME JC 1-028-6 NaOH
EXPNO 2
PROCNO 1

F2 - Acquisition Parameters
Date_ 20160302
Time 16.42
INSTRUM cryo500
PROBHD 5 mm CPTCI 1H-
PULPROG SpinEchopg30gp.prd
TD 65536
SOLVENT CDCl3
NS 128
DS 16
SWH 30303.031 Hz
FIDRES 0.462388 Hz
AQ 1.0813440 sec
RG 7298.2
DW 16.500 usec
DE 6.00 usec
TE 298.0 K
D1 0.25000000 sec
d11 0.03000000 sec
D16 0.00020000 sec
d17 0.00019600 sec
MCREST 0 sec
MCWRK 0.01500000 sec
P2 33.10 usec

======== CHANNEL f1 ========
NUC1 13C
P1 16.55 usec
P11 500.00 usec
P12 2000.00 usec
PL0 120.00 dB
PL1 -1.00 dB
SFO1 125.7942548 MHz
SP1 2.70 dB
SP2 2.70 dB
SPNAM[1] Crp60,0.5,20.1
SPNAM[2] Crp60comp.4
SPOFF1 0 Hz
SPOFF2 0 Hz

======== CHANNEL f2 ========
CPDPRG[2 waltz16
NUC2 1H
PCPD2 100.00 usec
PL2 1.60 dB
PL12 24.50 dB
SFO2 500.2225011 MHz

====== GRADIENT CHANNEL =====
GPNAM[1] SINE.100
GPNAM[2] SINE.100
GPX1 0 %
GPX2 0 %
GPY1 0 %
GPY2 0 %
GPZ1 30.00 %
GPZ2 50.00 %
p15 500.00 usec
p16 1000.00 usec

F2 - Processing parameters
SI 65536
SF 125.7804092 MHz
WDW EM
SSB 0
LB 1.00 Hz
GB 0
PC 2.00

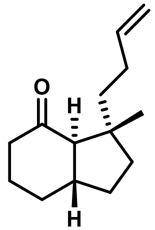

18

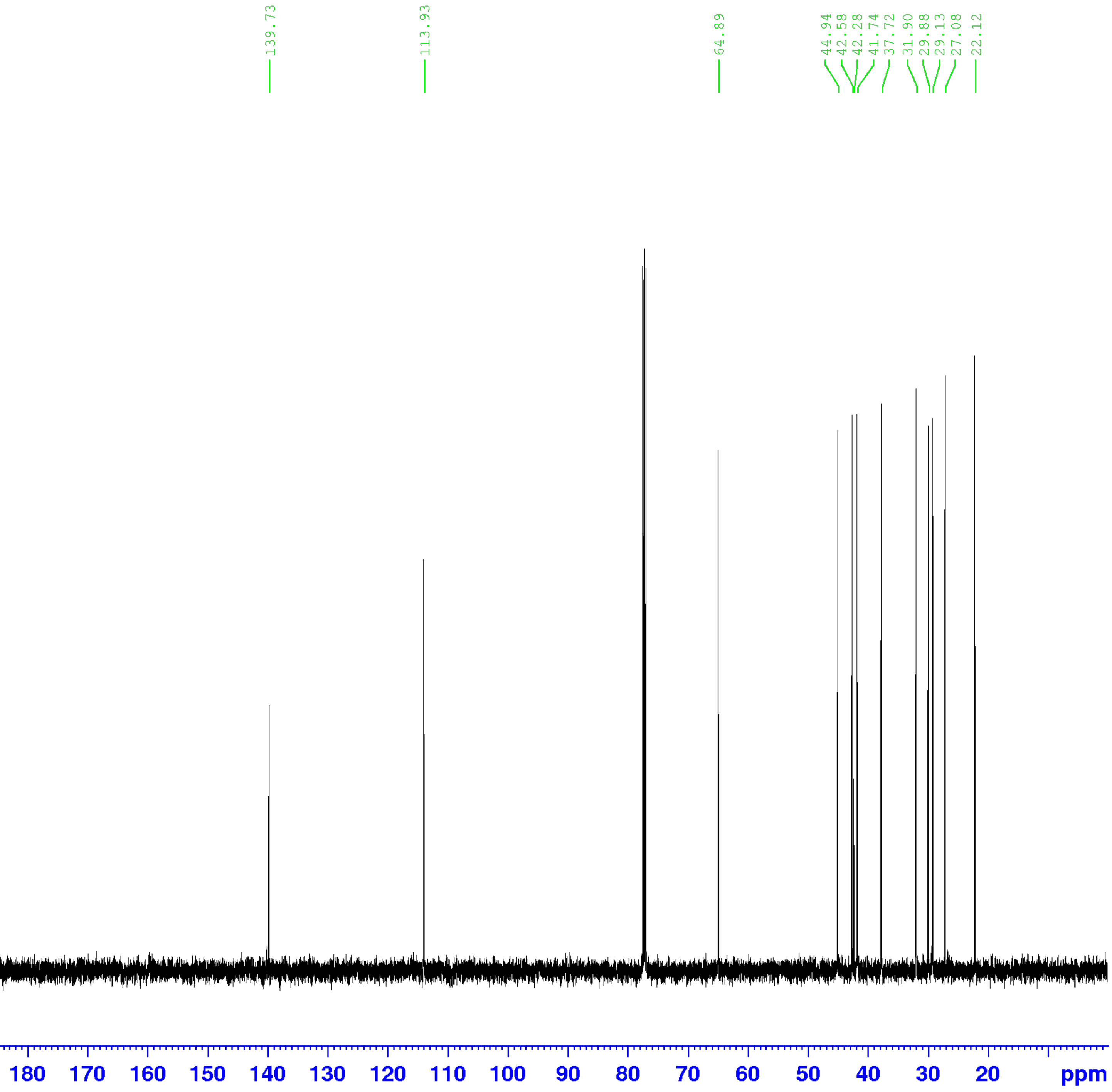

```
Current Data Parameters
NAME      JC 1-056-12 aldehyde
EXPNO                    1
PROCNO                   1

F2 - Acquisition Parameters
Date_            20160324
Time                16.27
INSTRUM           cryo500
PROBHD   5 mm CPTCI 1H-
PULPROG              zg30
TD                  81728
SOLVENT             CDCl3
NS                      8
DS                      2
SWH              8012.820 Hz
FIDRES           0.098043 Hz
AQ              5.0998273 sec
RG                      5
DW                 62.400 usec
DE                   6.00 usec
TE                  298.0 K
D1             0.10000000 sec
MCREST                  0 sec
MCWRK          0.01500000 sec

======== CHANNEL f1 ========
NUC1                   1H
P1                   7.50 usec
PL1                  1.60 dB
SFO1          500.2235015 MHz

F2 - Processing parameters
SI                  65536
SF            500.2200315 MHz
WDW                    EM
SSB                     0
LB                   0.30 Hz
GB                      0
PC                   4.00
```

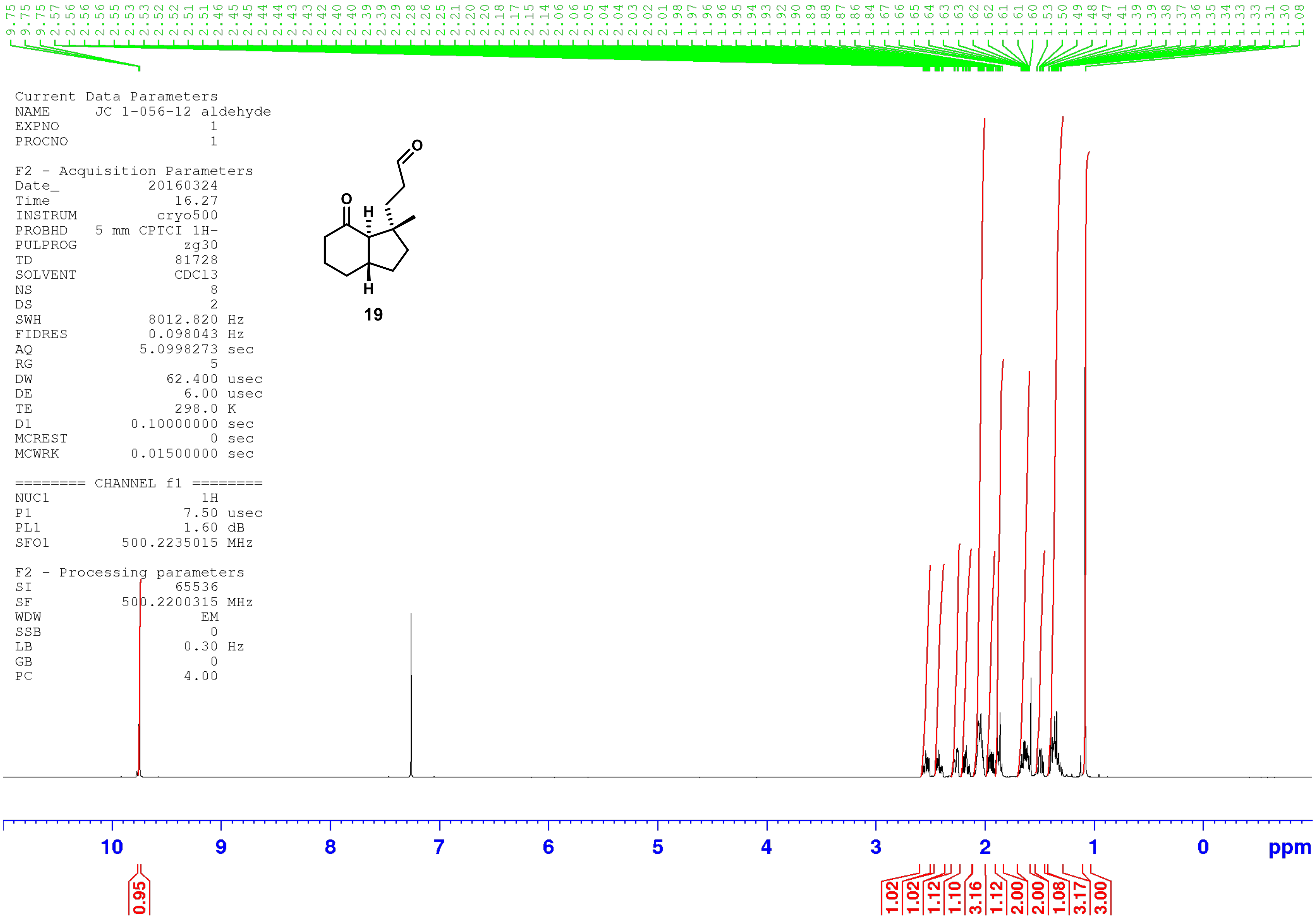

19

```
Current Data Parameters
NAME     JC 1-056-12 aldehyde
EXPNO                   2
PROCNO                  1

F2 - Acquisition Parameters
Date_          20160324
Time              16.29
INSTRUM         cryo500
PROBHD   5 mm CPTCI 1H-
PULPROG  SpinEchopg30gp.prd
TD                65536
SOLVENT           CDCl3
NS                   55
DS                   16
SWH           30303.031 Hz
FIDRES         0.462388 Hz
AQ            1.0813440 sec
RG               7298.2
DW               16.500 usec
DE                 6.00 usec
TE                298.0 K
D1           0.25000000 sec
d11          0.03000000 sec
D16          0.00020000 sec
d17          0.00019600 sec
MCREST                0 sec
MCWRK        0.01500000 sec
P2                33.10 usec

======== CHANNEL f1 ========
NUC1                13C
P1                16.55 usec
P11              500.00 usec
P12             2000.00 usec
PL0              120.00 dB
PL1               -1.00 dB
SFO1        125.7942548 MHz
SP1                2.70 dB
SP2                2.70 dB
SPNAM[1] Crp60,0.5,20.1
SPNAM[2]    Crp60comp.4
SPOFF1                0 Hz
SPOFF2                0 Hz

======== CHANNEL f2 ========
CPDPRG[2        waltz16
NUC2                 1H
PCPD2            100.00 usec
PL2                1.60 dB
PL12              24.50 dB
SFO2        500.2225011 MHz

====== GRADIENT CHANNEL =====
GPNAM[1]       SINE.100
GPNAM[2]       SINE.100
GPX1                  0 %
GPX2                  0 %
GPY1                  0 %
GPY2                  0 %
GPZ1              30.00 %
GPZ2              50.00 %
p15              500.00 usec
p16             1000.00 usec

F2 - Processing parameters
SI                65536
SF          125.7804081 MHz
WDW                  EM
SSB                   0
LB                 1.00 Hz
GB                    0
PC                 2.00
```

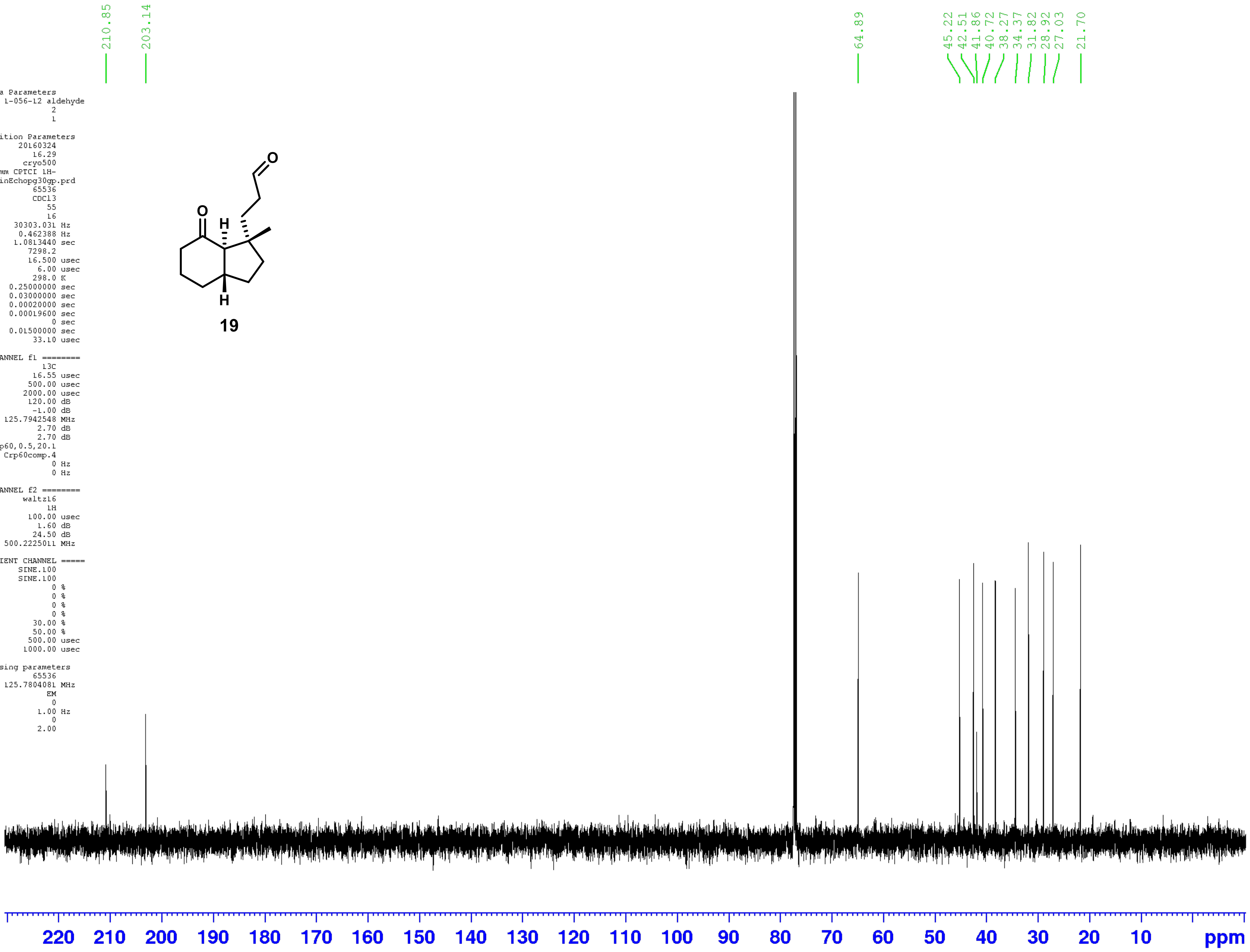

```
Current Data Parameters
NAME      JC 1-284 NHC cyclise recryst
EXPNO                    1
PROCNO                   1

F2 - Acquisition Parameters
Date_             20160912
Time                 19.15
INSTRUM              av600
PROBHD    5 mm CPBBO BB-
PULPROG               zg30
TD                   57690
SOLVENT              CDCl3
NS                       8
DS                       0
SWH               9615.385 Hz
FIDRES            0.166673 Hz
AQ               2.9998801 sec
RG                      10
DW                  52.000 usec
DE                   13.70 usec
TE                   298.0 K
D1              0.10000000 sec
TD0                      1

======== CHANNEL f1 ========
SFO1           600.1342009 MHz
NUC1                    1H
P1                   12.00 usec
PLW1           20.00000000 W

F2 - Processing parameters
SI                   65536
SF             600.1300357 MHz
WDW                     EM
SSB                      0
LB                    0.30 Hz
GB                       0
PC                    1.00
```

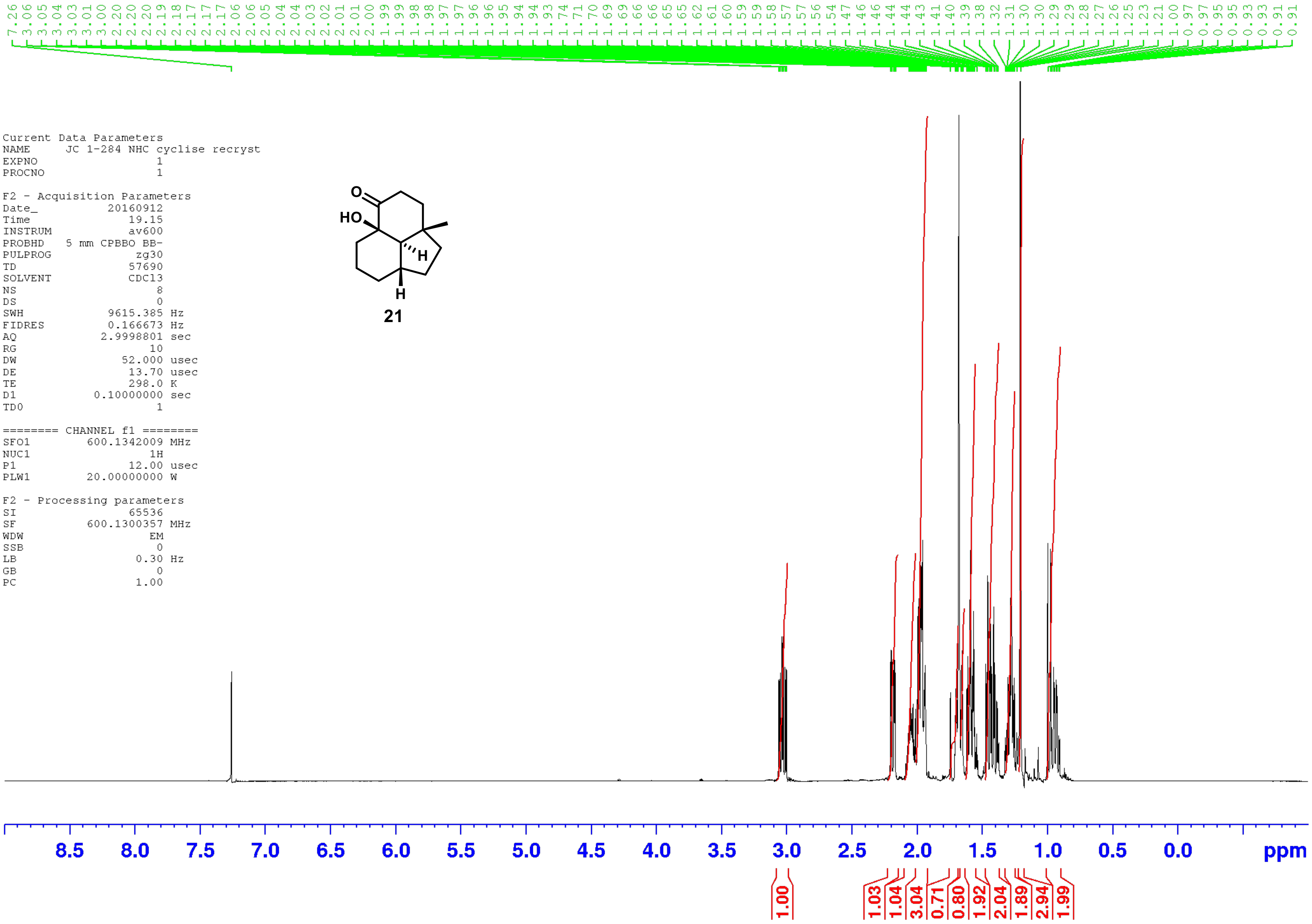

21

```
Current Data Parameters
NAME      JC 1-284 NHC cyclise recryst
EXPNO                  2
PROCNO                 1

F2 - Acquisition Parameters
Date_           20160912
Time               19.20
INSTRUM            av600
PROBHD    5 mm CPBBO BB-
PULPROG          zgdc30
TD                 65536
SOLVENT            CDCl3
NS                    79
DS                     4
SWH           36231.883 Hz
FIDRES         0.552855 Hz
AQ            0.9043968 sec
RG                  2050
DW               13.800 usec
DE                19.65 usec
TE                298.0 K
D1           0.40000001 sec
D11          0.03000000 sec
TD0                    1

======== CHANNEL f1 ========
SFO1        150.9194075 MHz
NUC1                 13C
P1                 10.00 usec
PLW1         64.00000000 W

======== CHANNEL f2 ========
SFO2        600.1330010 MHz
NUC2                  1H
CPDPRG[2         waltz16
PCPD2              80.00 usec
PLW2         20.00000000 W
PLW12         0.36000001 W

F2 - Processing parameters
SI                 65536
SF           150.9027969 MHz
WDW                   EM
SSB                    0
LB                  1.00 Hz
GB                     0
PC                  1.00
```

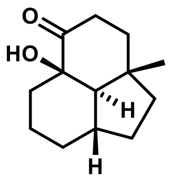

21

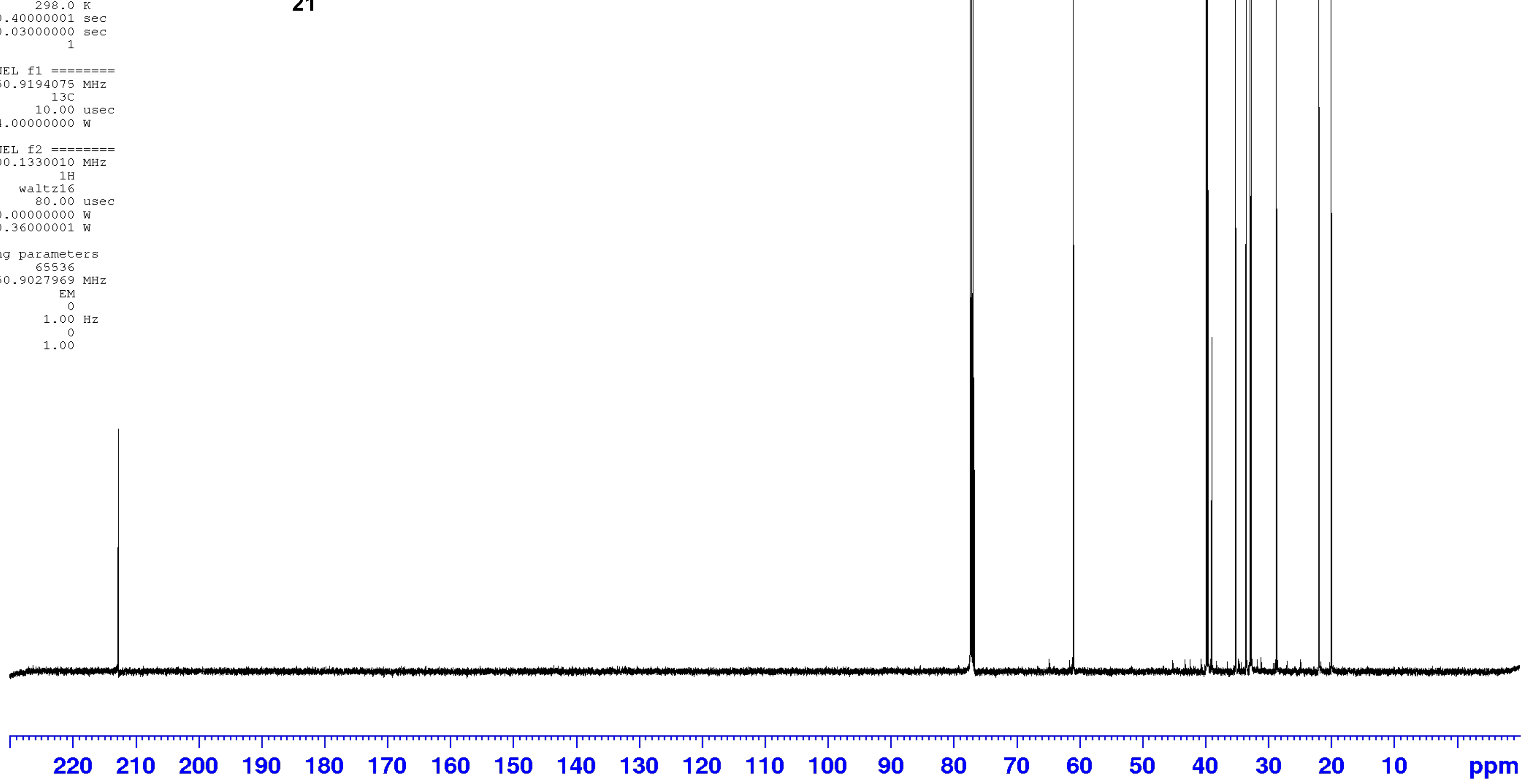

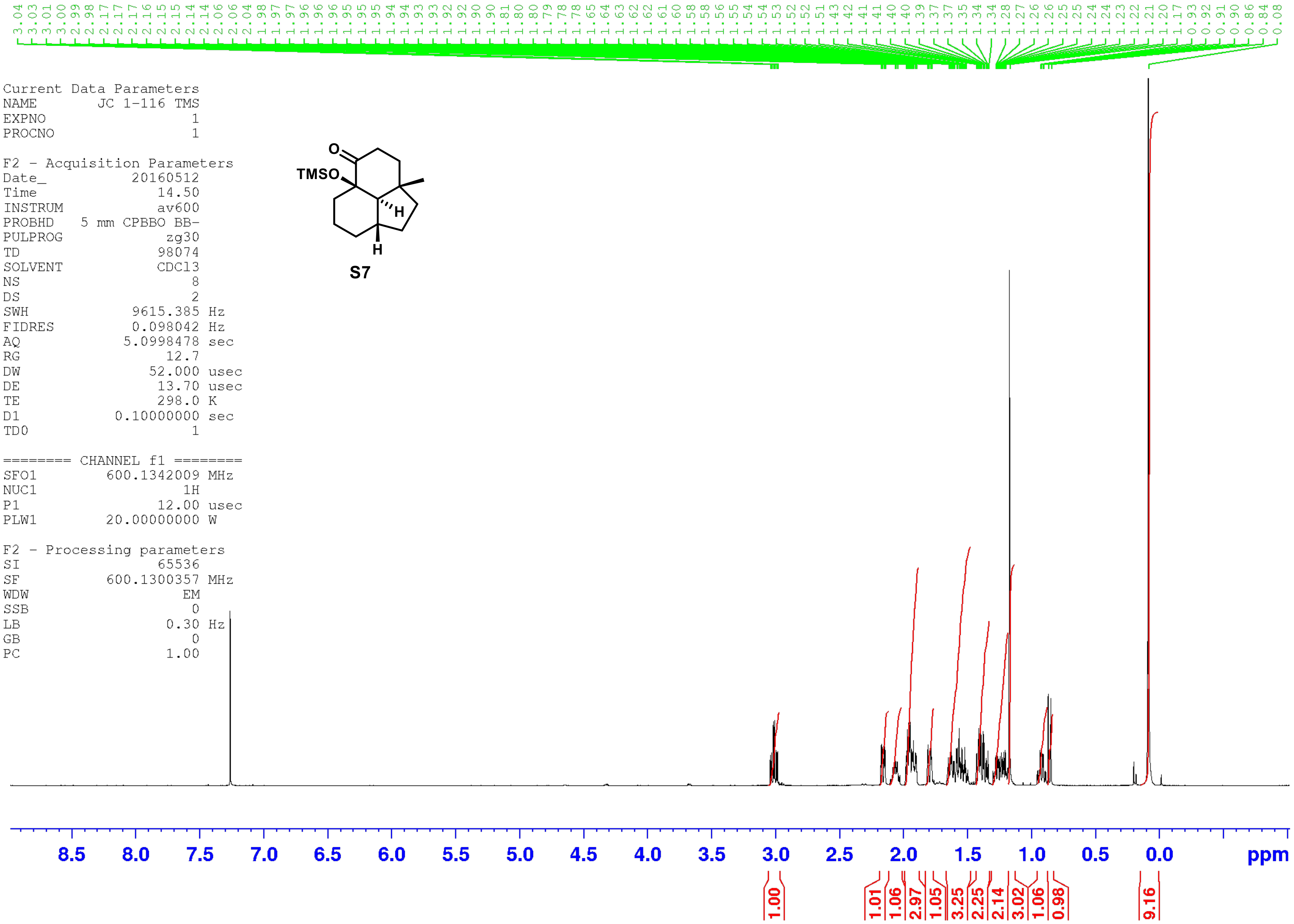
3.04 3.03 3.01 3.00 2.99 2.98 2.17 2.17 2.16 2.15 2.15 2.14 2.06 2.06 2.04 1.98 1.97 1.97 1.96 1.96 1.95 1.95 1.95 1.94 1.93 1.93 1.92 1.90 1.90 1.81 1.80 1.80 1.79 1.78 1.78 1.65 1.64 1.63 1.62 1.62 1.61 1.60 1.58 1.58 1.56 1.55 1.54 1.54 1.53 1.52 1.52 1.51 1.43 1.42 1.41 1.41 1.40 1.40 1.39 1.37 1.37 1.35 1.34 1.34 1.28 1.27 1.26 1.26 1.25 1.25 1.24 1.24 1.23 1.22 1.21 1.20 1.17 0.93 0.92 0.91 0.90 0.86 0.84 0.08
Current Data Parameters
NAME JC 1-116 TMS
EXPNO 1
PROCNO 1
F2 - Acquisition Parameters
Date_ 20160512
Time 14.50
INSTRUM av600
PROBHD 5 mm CPBBO BB-
PULPROG zg30
TD 98074
SOLVENT CDCl3
NS 8
DS 2
SWH 9615.385 Hz
FIDRES 0.098042 Hz
AQ 5.0998478 sec
RG 12.7
DW 52.000 usec
DE 13.70 usec
TE 298.0 K
D1 0.10000000 sec
TD0 1
======== CHANNEL f1 ========
SFO1 600.1342009 MHz
NUC1 1H
P1 12.00 usec
PLW1 20.00000000 W
F2 - Processing parameters
SI 65536
SF 600.1300357 MHz
WDW EM
SSB 0
LB 0.30 Hz
GB 0
PC 1.00
O
TMSO
H
H
S7
8.5 8.0 7.5 7.0 6.5 6.0 5.5 5.0 4.5 4.0 3.5 3.0 2.5 2.0 1.5 1.0 0.5 0.0 ppm
1.00 1.01 1.06 2.97 1.05 3.25 2.25 2.14 3.02 1.06 0.98 9.16

Current Data Parameters
NAME JC 1-116 TMS
EXPNO 2
PROCNO 1

F2 - Acquisition Parameters
Date_ 20160512
Time 14.52
INSTRUM av600
PROBHD 5 mm CPBBO BB-
PULPROG zgdc30
TD 65536
SOLVENT CDCl3
NS 38
DS 4
SWH 36231.883 Hz
FIDRES 0.552855 Hz
AQ 0.9043968 sec
RG 2050
DW 13.800 usec
DE 19.65 usec
TE 298.0 K
D1 0.40000001 sec
D11 0.03000000 sec
TD0 1

======== CHANNEL f1 ========
SFO1 150.9194075 MHz
NUC1 13C
P1 10.00 usec
PLW1 64.00000000 W

======== CHANNEL f2 ========
SFO2 600.1330010 MHz
NUC2 1H
CPDPRG[2 waltz16
PCPD2 80.00 usec
PLW2 20.00000000 W
PLW12 0.36000001 W

F2 - Processing parameters
SI 65536
SF 150.9027931 MHz
WDW EM
SSB 0
LB 1.00 Hz
GB 0
PC 1.00

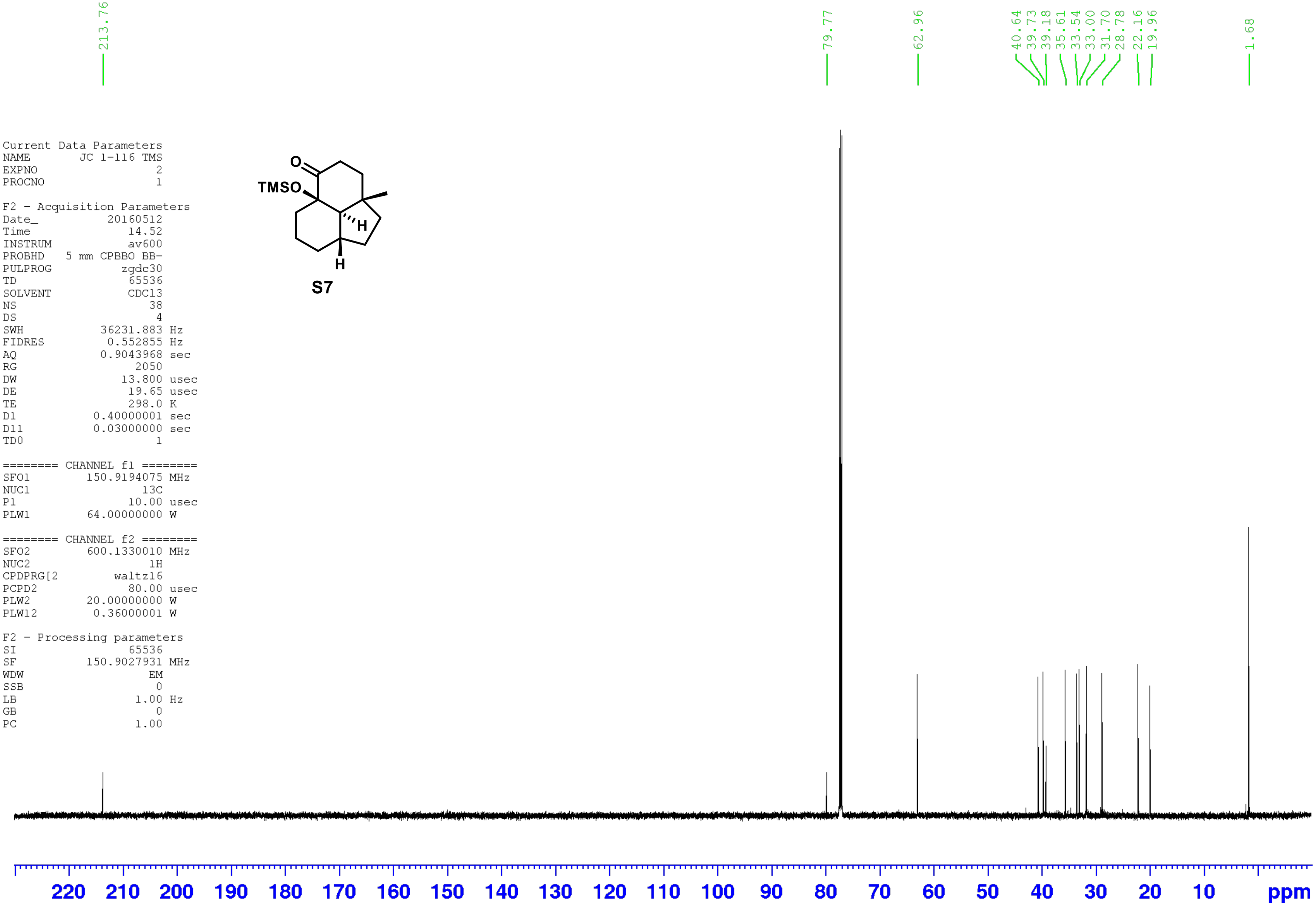

Current Data Parameters
NAME JC 1-112 OTf KHMDS
EXPNO 1
PROCNO 1

F2 - Acquisition Parameters
Date_ 20160510
Time 22.54
INSTRUM gn500
PROBHD 5 mm broadband
PULPROG zg30
TD 81728
SOLVENT CDCl3
NS 8
DS 2
SWH 8012.820 Hz
FIDRES 0.098043 Hz
AQ 5.0998273 sec
RG 812.7
DW 62.400 usec
DE 6.00 usec
TE 298.0 K
D1 0.10000000 sec
MCREST 0 sec
MCWRK 0.01500000 sec

======== CHANNEL f1 ========
NUC1 1H
P1 12.00 usec
PL1 -5.80 dB
SFO1 499.0734935 MHz

F2 - Processing parameters
SI 65536
SF 499.0700313 MHz
WDW EM
SSB 0
LB 0.30 Hz
GB 0
PC 1.00

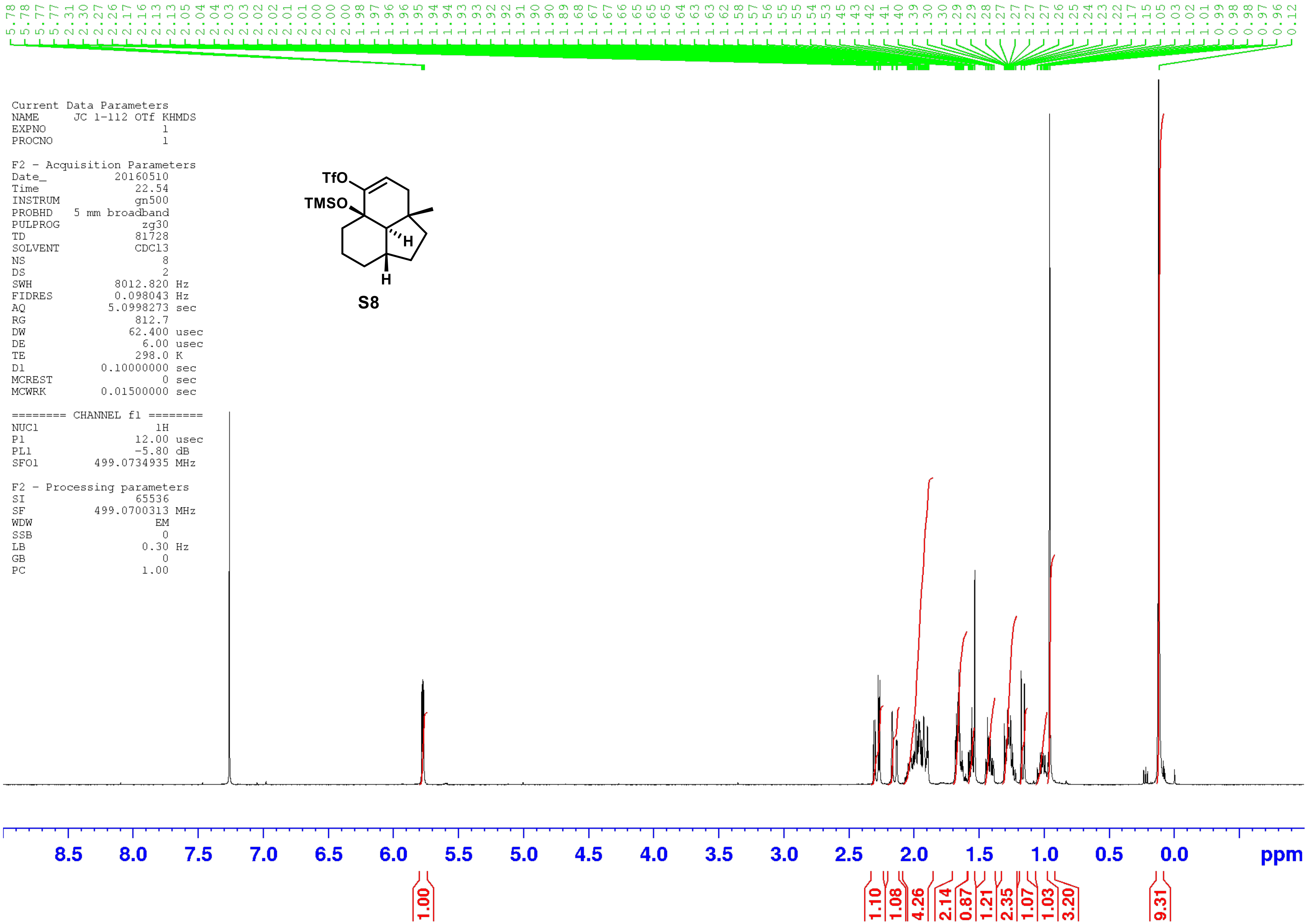

Current Data Parameters
NAME JC 1-111 OTf pub
EXPNO 2
PROCNO 1

F2 - Acquisition Parameters
Date_ 20160820
Time 1.45
INSTRUM av600
PROBHD 5 mm CPBBO BB-
PULPROG zgdc30
TD 65536
SOLVENT CDCl3
NS 277
DS 4
SWH 36231.883 Hz
FIDRES 0.552855 Hz
AQ 0.9043968 sec
RG 2050
DW 13.800 usec
DE 19.65 usec
TE 298.0 K
D1 2.00000000 sec
D11 0.03000000 sec
TD0 1

======== CHANNEL f1 ========
SFO1 150.9194075 MHz
NUC1 13C
P1 10.00 usec
PLW1 64.00000000 W

======== CHANNEL f2 ========
SFO2 600.1330010 MHz
NUC2 1H
CPDPRG[2 waltz16
PCPD2 80.00 usec
PLW2 20.00000000 W
PLW12 0.36000001 W

F2 - Processing parameters
SI 65536
SF 150.9027921 MHz
WDW EM
SSB 0
LB 1.00 Hz
GB 0
PC 1.00

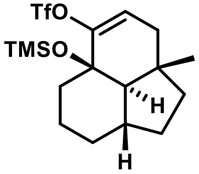

S8

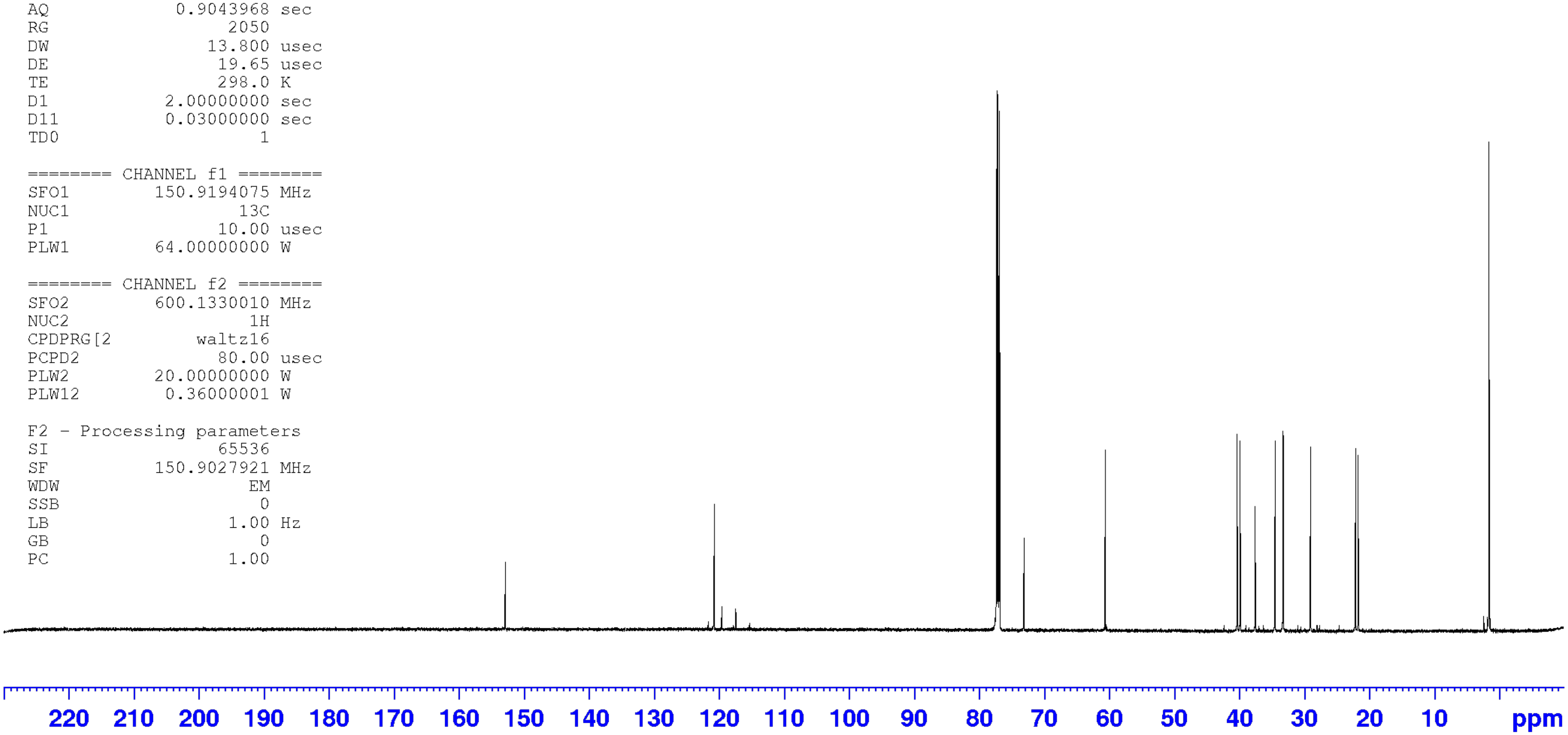

Current Data Parameters
NAME JC 1-126 A1
EXPNO 1
PROCNO 1

F2 - Acquisition Parameters
Date_ 20160517
Time 19.53
INSTRUM cryo500
PROBHD 5 mm CPTCI 1H-
PULPROG zg30
TD 81728
SOLVENT CDCl3
NS 8
DS 2
SWH 8012.820 Hz
FIDRES 0.098043 Hz
AQ 5.0998273 sec
RG 6.3
DW 62.400 usec
DE 6.00 usec
TE 298.0 K
D1 0.10000000 sec
MCREST 0 sec
MCWRK 0.01500000 sec

======== CHANNEL f1 ========
NUC1 1H
P1 7.50 usec
PL1 1.60 dB
SFO1 500.2235015 MHz

F2 - Processing parameters
SI 65536
SF 500.2200319 MHz
WDW EM
SSB 0
LB 0.30 Hz
GB 0
PC 4.00

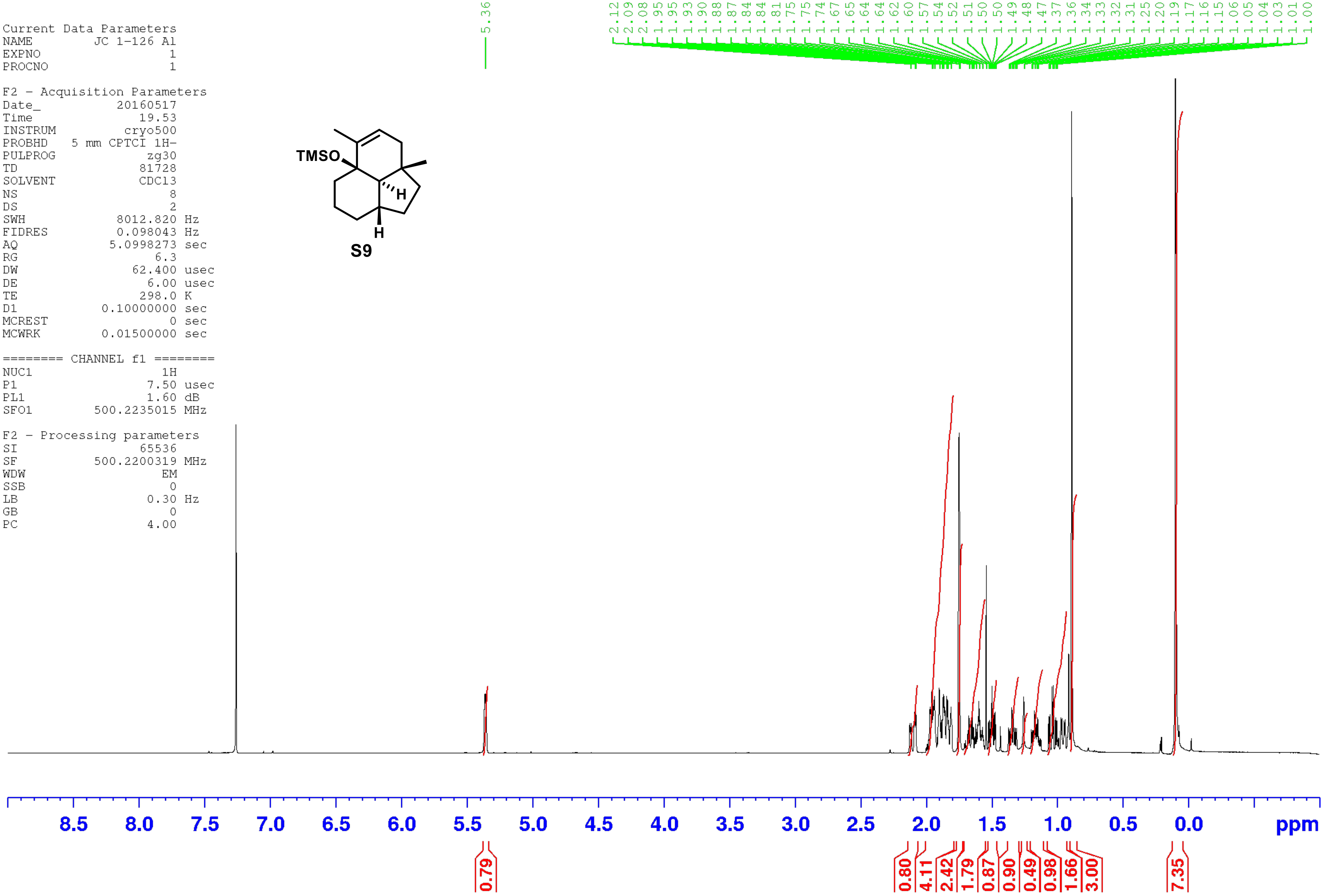

Current Data Parameters
NAME JC 1-126 A1
EXPNO 2
PROCNO 1

F2 - Acquisition Parameters
Date_ 20160517
Time 19.55
INSTRUM cryo500
PROBHD 5 mm CPTCI 1H-
PULPROG SpinEchopg30gp.prd
TD 65536
SOLVENT CDCl3
NS 45
DS 16
SWH 30303.031 Hz
FIDRES 0.462388 Hz
AQ 1.0813440 sec
RG 2298.8
DW 16.500 usec
DE 6.00 usec
TE 298.0 K
D1 1.00000000 sec
d11 0.03000000 sec
D16 0.00020000 sec
d17 0.00019600 sec
MCREST 0 sec
MCWRK 0.01500000 sec
P2 33.10 usec

======== CHANNEL f1 ========
NUC1 13C
P1 16.55 usec
P11 500.00 usec
P12 2000.00 usec
PL0 120.00 dB
PL1 -1.00 dB
SFO1 125.7942548 MHz
SP1 2.70 dB
SP2 2.70 dB
SPNAM[1] Crp60,0.5,20.1
SPNAM[2] Crp60comp.4
SPOFF1 0 Hz
SPOFF2 0 Hz

======== CHANNEL f2 ========
CPDPRG[2 waltz16
NUC2 1H
PCPD2 100.00 usec
PL2 1.60 dB
PL12 24.50 dB
SFO2 500.2225011 MHz

====== GRADIENT CHANNEL =====
GPNAM[1] SINE.100
GPNAM[2] SINE.100
GPX1 0 %
GPX2 0 %
GPY1 0 %
GPY2 0 %
GPZ1 30.00 %
GPZ2 50.00 %
p15 500.00 usec
p16 1000.00 usec

F2 - Processing parameters
SI 65536
SF 125.7804074 MHz
WDW EM
SSB 0
LB 1.00 Hz
GB 0
PC 2.00

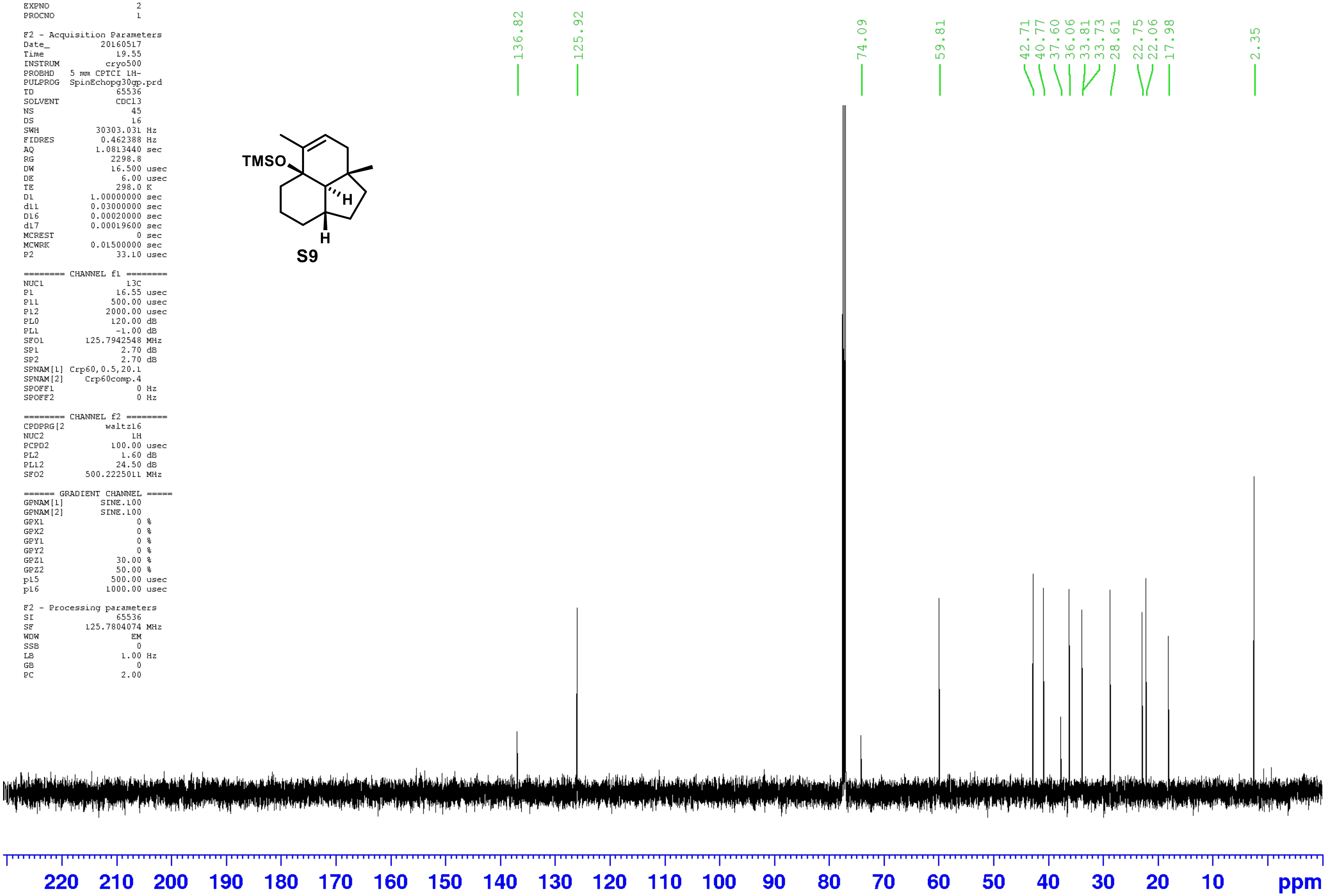

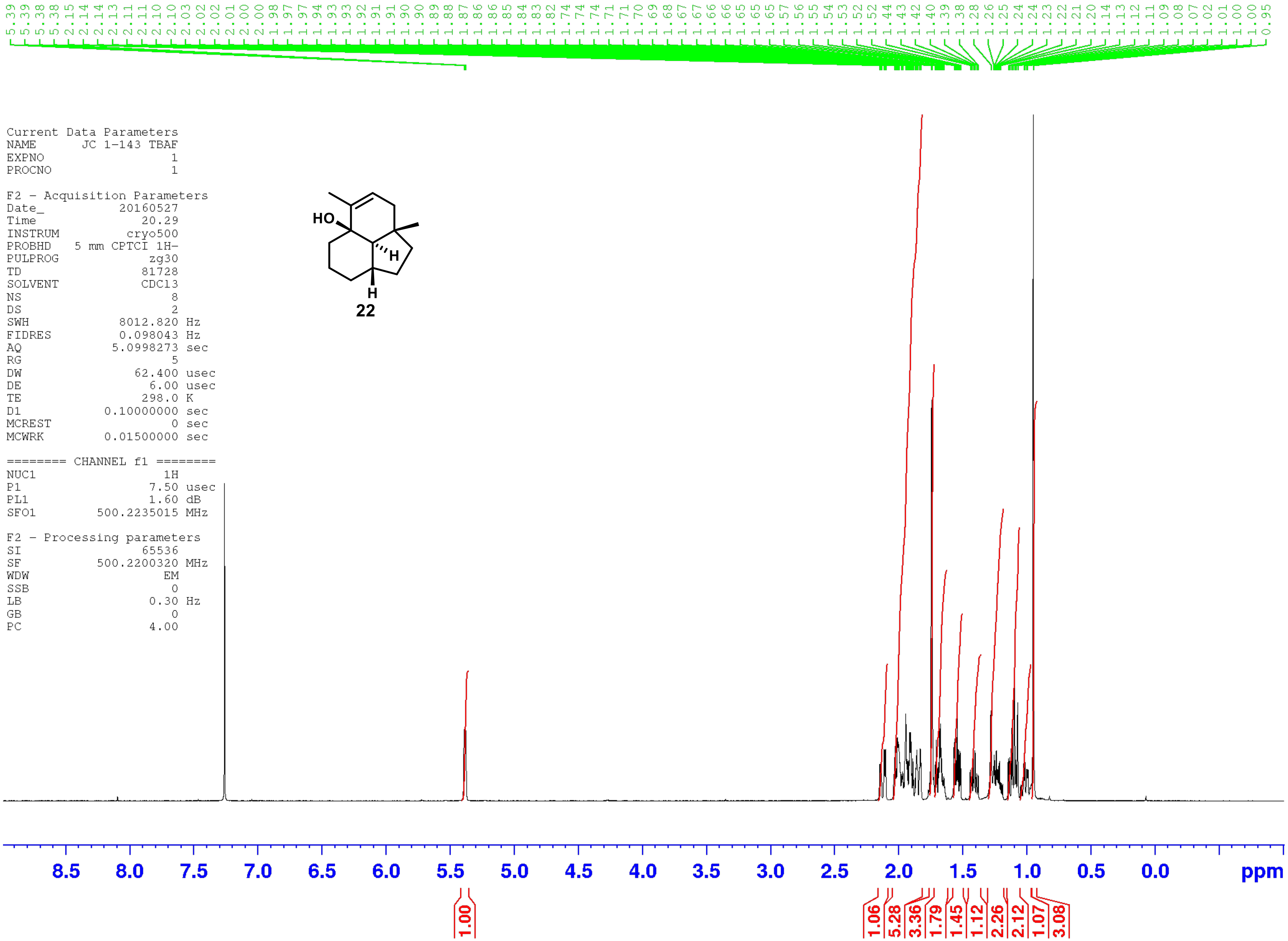

5.39 5.39 5.38 5.38 2.15 2.14 2.13 2.11 2.11 2.10 2.10 2.03 2.02 2.02 2.01 2.00 2.00 1.98 1.97 1.97 1.94 1.93 1.93 1.92 1.91 1.91 1.90 1.90 1.89 1.88 1.87 1.86 1.85 1.84 1.83 1.82 1.74 1.74 1.74 1.71 1.70 1.69 1.68 1.67 1.67 1.66 1.65 1.65 1.65 1.57 1.56 1.55 1.54 1.53 1.52 1.52 1.44 1.43 1.42 1.40 1.39 1.38 1.28 1.26 1.25 1.24 1.24 1.23 1.22 1.21 1.20 1.14 1.13 1.12 1.11 1.09 1.08 1.07 1.02 1.01 1.00 0.95
Current Data Parameters
NAME JC 1-143 TBAF
EXPNO 1
PROCNO 1
F2 - Acquisition Parameters
Date_ 20160527
Time 20.29
INSTRUM cryo500
PROBHD 5 mm CPTCI 1H-
PULPROG zg30
TD 81728
SOLVENT CDCl3
NS 8
DS 2
SWH 8012.820 Hz
FIDRES 0.098043 Hz
AQ 5.0998273 sec
RG 5
DW 62.400 usec
DE 6.00 usec
TE 298.0 K
D1 0.10000000 sec
MCREST 0 sec
MCWRK 0.01500000 sec
======== CHANNEL f1 ========
NUC1 1H
P1 7.50 usec
PL1 1.60 dB
SFO1 500.2235015 MHz
F2 - Processing parameters
SI 65536
SF 500.2200320 MHz
WDW EM
SSB 0
LB 0.30 Hz
GB 0
PC 4.00
HO
H
H
22
8.5 8.0 7.5 7.0 6.5 6.0 5.5 5.0 4.5 4.0 3.5 3.0 2.5 2.0 1.5 1.0 0.5 0.0 ppm
1.00
1.06 5.28 3.36 1.79 1.45 1.12 2.26 2.12 1.07 3.08

```
Current Data Parameters
NAME       JC 1-179 TBAF
EXPNO                  2
PROCNO                 1

F2 - Acquisition Parameters
Date_           20160622
Time               18.54
INSTRUM          cryo500
PROBHD   5 mm CPTCI 1H-
PULPROG  SpinEchopg30gp.prd
TD                 65536
SOLVENT            CDC13
NS                    55
DS                    16
SWH            30303.031 Hz
FIDRES          0.462388 Hz
AQ             1.0813440 sec
RG                  3251
DW                16.500 usec
DE                  6.00 usec
TE                 298.0 K
D1            0.25000000 sec
d11           0.03000000 sec
D16           0.00020000 sec
d17           0.00019600 sec
MCREST                 0 sec
MCWRK         0.01500000 sec
P2                 33.10 usec

======== CHANNEL f1 ========
NUC1                 13C
P1                 16.55 usec
P11               500.00 usec
P12              2000.00 usec
PL0               120.00 dB
PL1                -1.00 dB
SFO1         125.7942548 MHz
SP1                 2.70 dB
SP2                 2.70 dB
SPNAM[1] Crp60,0.5,20.1
SPNAM[2]     Crp60comp.4
SPOFF1                 0 Hz
SPOFF2                 0 Hz

======== CHANNEL f2 ========
CPDPRG[2         waltz16
NUC2                  1H
PCPD2             100.00 usec
PL2                 1.60 dB
PL12               24.50 dB
SFO2         500.2225011 MHz

====== GRADIENT CHANNEL =====
GPNAM[1]        SINE.100
GPNAM[2]        SINE.100
GPX1                   0 %
GPX2                   0 %
GPY1                   0 %
GPY2                   0 %
GPZ1               30.00 %
GPZ2               50.00 %
p15               500.00 usec
p16              1000.00 usec

F2 - Processing parameters
SI                 65536
SF           125.7804075 MHz
WDW                   EM
SSB                    0
LB                  1.00 Hz
GB                     0
PC                  2.00
```

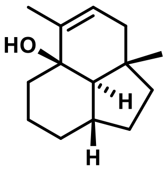

22

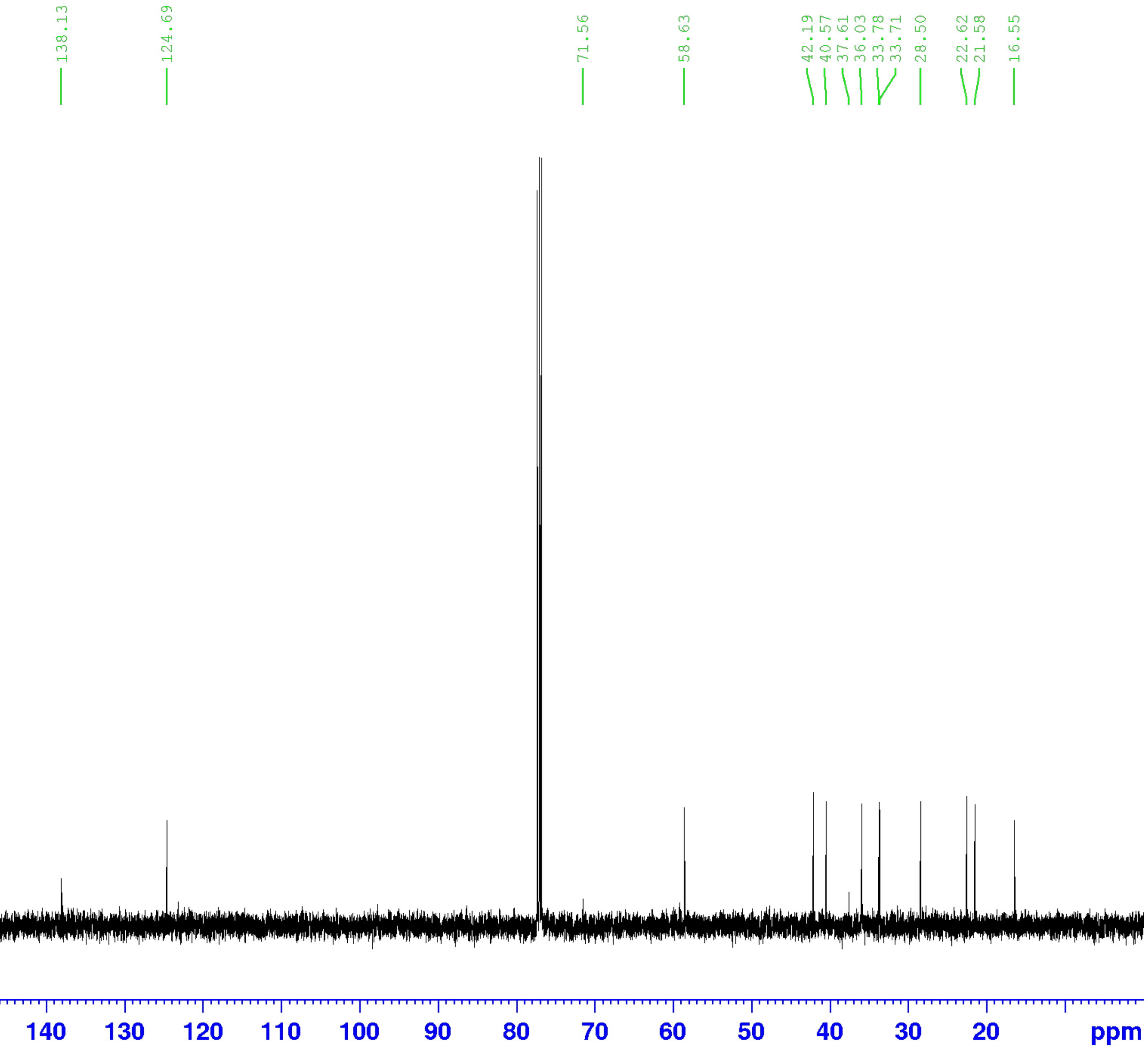

Current Data Parameters
NAME JC 1-194 Sn
EXPNO 1
PROCNO 1

F2 - Acquisition Parameters
Date_ 20160709
Time 16.03
INSTRUM cryo500
PROBHD 5 mm CPTCI 1H-
PULPROG zg30
TD 81728
SOLVENT CDCl3
NS 8
DS 2
SWH 8012.820 Hz
FIDRES 0.098043 Hz
AQ 5.0998273 sec
RG 6.3
DW 62.400 usec
DE 6.00 usec
TE 298.0 K
D1 0.10000000 sec
MCREST 0 sec
MCWRK 0.01500000 sec

======== CHANNEL f1 ========
NUC1 1H
P1 7.50 usec
PL1 1.60 dB
SFO1 500.2235015 MHz

F2 - Processing parameters
SI 65536
SF 500.2200313 MHz
WDW EM
SSB 0
LB 0.30 Hz
GB 0
PC 4.00

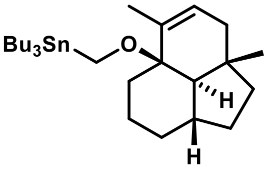

23

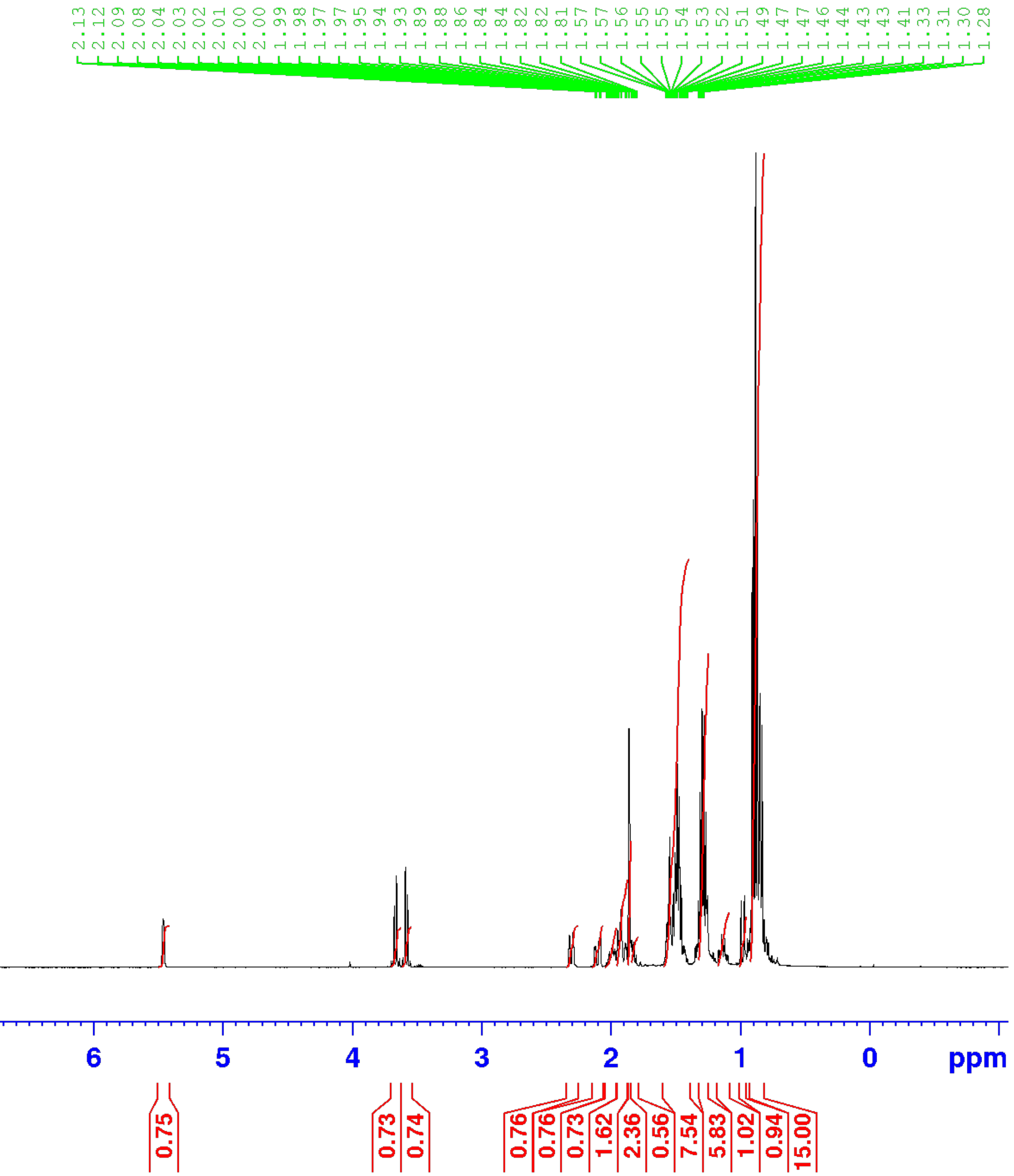

Current Data Parameters
NAME JC 1-194 Sn
EXPNO 2
PROCNO 1

F2 - Acquisition Parameters
Date_ 20160709
Time 16.06
INSTRUM cryo500
PROBHD 5 mm CPTCI 1H-
PULPROG SpinEchopg30gp.prd
TD 65536
SOLVENT CDCl3
NS 163
DS 16
SWH 30303.031 Hz
FIDRES 0.462388 Hz
AQ 1.0813440 sec
RG 5792.6
DW 16.500 usec
DE 6.00 usec
TE 298.0 K
D1 0.25000000 sec
d11 0.03000000 sec
D16 0.00020000 sec
d17 0.00019600 sec
MCREST 0 sec
MCWRK 0.01500000 sec
P2 33.10 usec

======== CHANNEL f1 ========
NUC1 13C
P1 16.55 usec
P11 500.00 usec
P12 2000.00 usec
PL0 120.00 dB
PL1 -1.00 dB
SFO1 125.7942548 MHz
SP1 2.70 dB
SP2 2.70 dB
SPNAM[1] Crp60,0.5,20.1
SPNAM[2] Crp60comp.4
SPOFF1 0 Hz
SPOFF2 0 Hz

======== CHANNEL f2 ========
CPDPRG[2 waltz16
NUC2 1H
PCPD2 100.00 usec
PL2 1.60 dB
PL12 24.50 dB
SFO2 500.2225011 MHz

====== GRADIENT CHANNEL =====
GPNAM[1] SINE.100
GPNAM[2] SINE.100
GPX1 0 %
GPX2 0 %
GPY1 0 %
GPY2 0 %
GPZ1 30.00 %
GPZ2 50.00 %
p15 500.00 usec
p16 1000.00 usec

F2 - Processing parameters
SI 65536
SF 125.7804070 MHz
WDW EM
SSB 0
LB 1.00 Hz
GB 0
PC 2.00

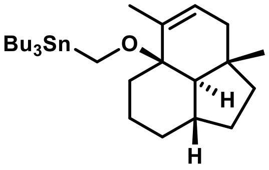

23

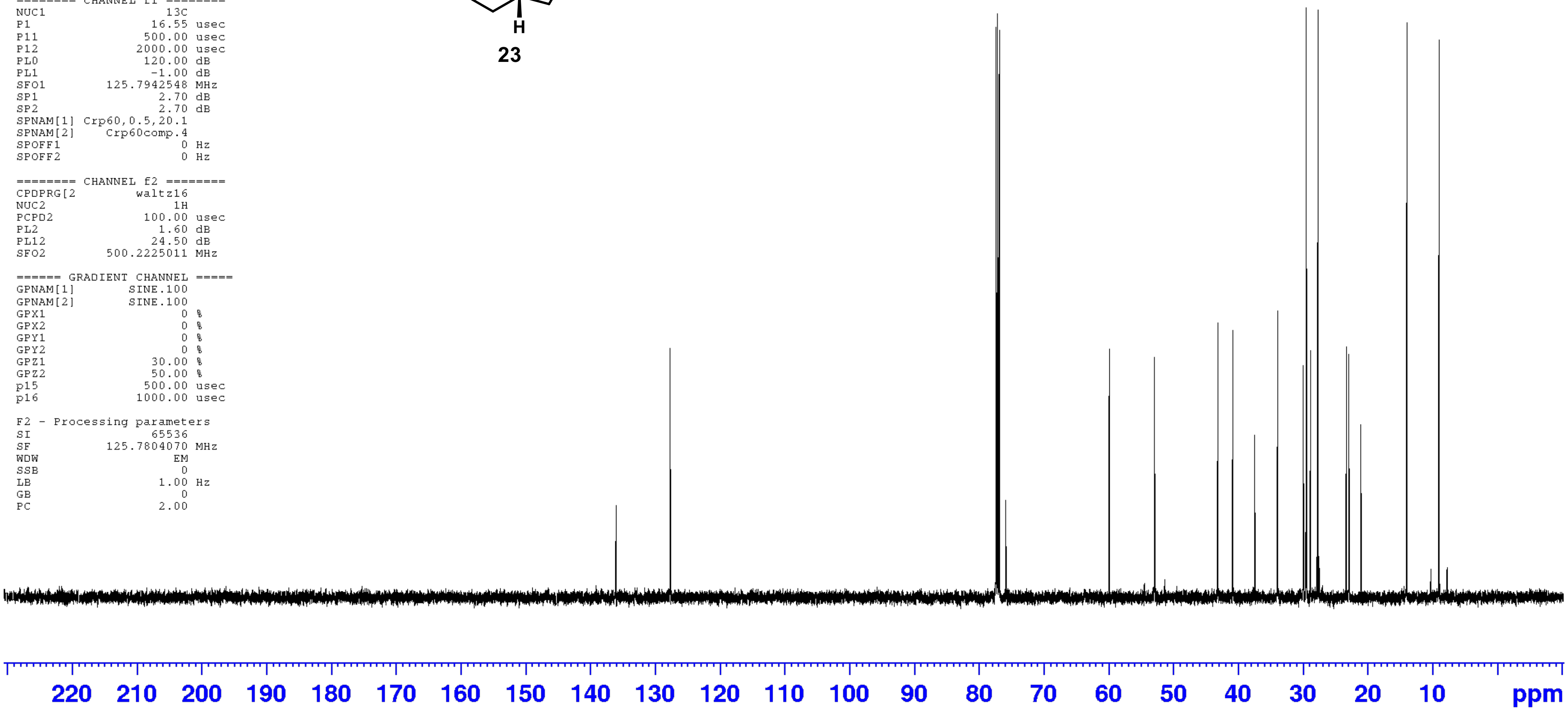

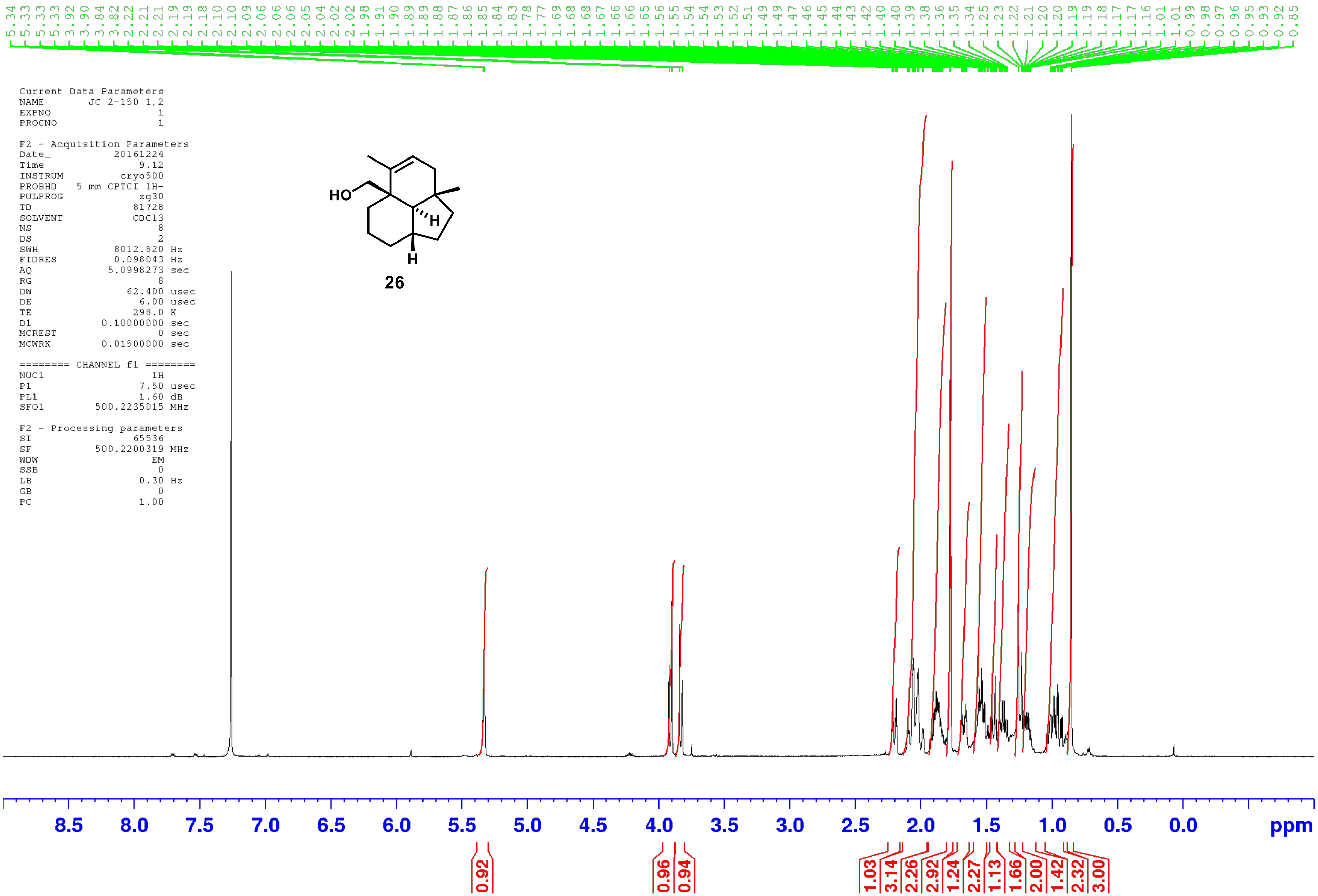
5.34 5.33 5.33 5.33 3.92 3.90 3.84 3.82 2.22 2.21 2.21 2.19 2.19 2.18 2.10 2.10 2.09 2.06 2.06 2.06 2.05 2.04 2.02 2.02 1.98 1.91 1.90 1.89 1.89 1.88 1.87 1.86 1.85 1.84 1.83 1.78 1.77 1.69 1.68 1.67 1.66 1.66 1.65 1.56 1.55 1.54 1.54 1.53 1.52 1.51 1.49 1.49 1.47 1.46 1.45 1.44 1.43 1.42 1.40 1.40 1.39 1.38 1.36 1.35 1.34 1.25 1.23 1.22 1.21 1.20 1.19 1.19 1.18 1.17 1.17 1.16 1.01 1.01 0.99 0.98 0.97 0.96 0.95 0.93 0.92 0.85
Current Data Parameters
NAME JC 2-150 1,2
EXPNO 1
PROCNO 1
F2 - Acquisition Parameters
Date_ 20161224
Time 9.12
INSTRUM cryo500
PROBHD 5 mm CPTCI 1H-
PULPROG zg30
TD 81728
SOLVENT CDCl3
NS 8
DS 2
SWH 8012.820 Hz
FIDRES 0.098043 Hz
AQ 5.0998273 sec
RG 8
DW 62.400 usec
DE 6.00 usec
TE 298.0 K
D1 0.10000000 sec
MCREST 0 sec
MCWRK 0.01500000 sec
======== CHANNEL f1 ========
NUC1 1H
P1 7.50 usec
PL1 1.60 dB
SFO1 500.2235015 MHz
F2 - Processing parameters
SI 65536
SF 500.2200319 MHz
WDW EM
SSB 0
LB 0.30 Hz
GB 0
PC 1.00
HO
H
H
26
8.5 8.0 7.5 7.0 6.5 6.0 5.5 5.0 4.5 4.0 3.5 3.0 2.5 2.0 1.5 1.0 0.5 0.0 ppm
0.92
0.96 0.94
1.03 3.14 2.26 2.92 1.24 2.27 1.13 1.66 2.00 1.42 2.32 3.00

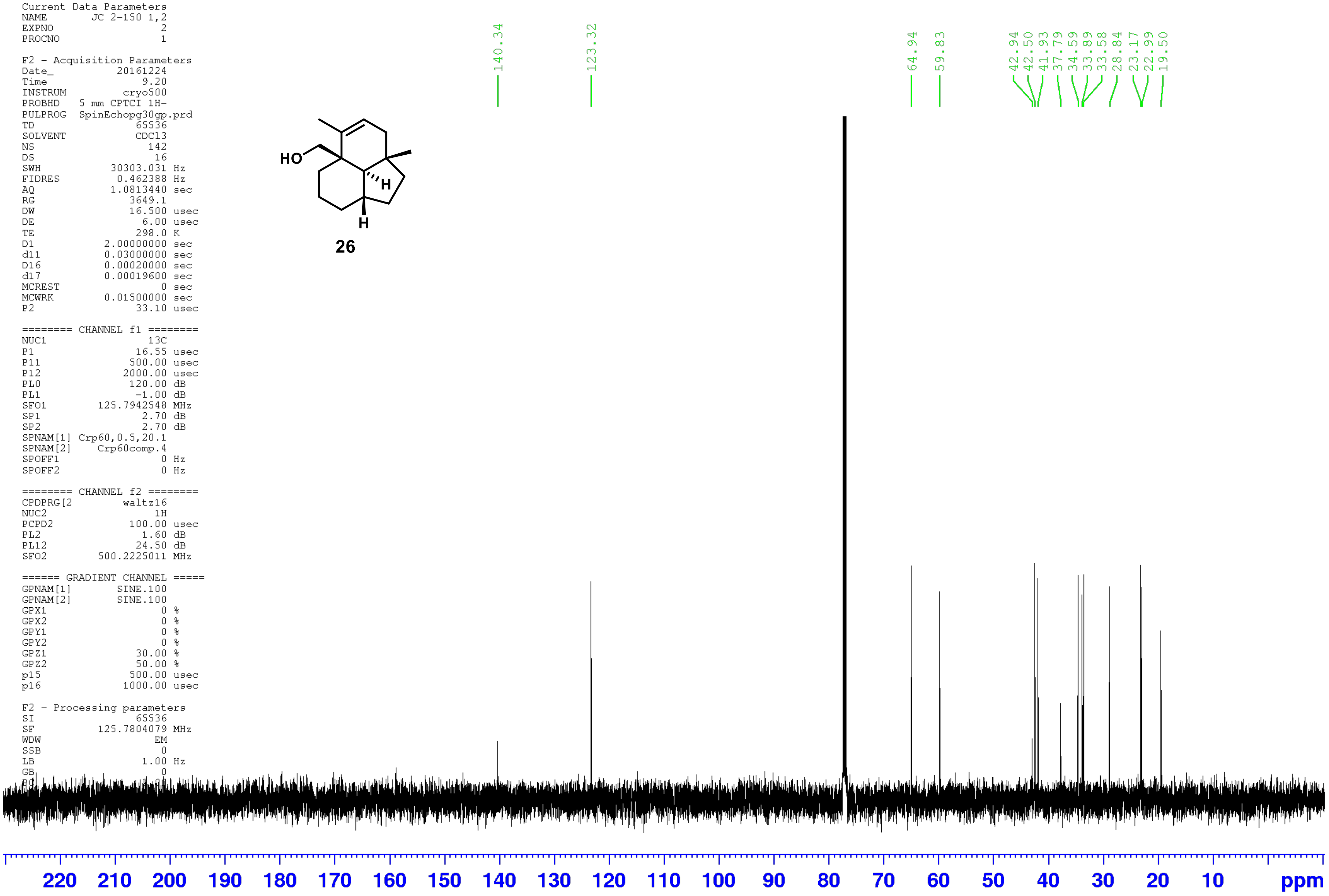
Current Data Parameters
NAME JC 2-150 1,2
EXPNO 2
PROCNO 1
F2 - Acquisition Parameters
Date_ 20161224
Time 9.20
INSTRUM cryo500
PROBHD 5 mm CPTCI 1H-
PULPROG SpinEchopg30gp.prd
TD 65536
SOLVENT CDCl3
NS 142
DS 16
SWH 30303.031 Hz
FIDRES 0.462388 Hz
AQ 1.0813440 sec
RG 3649.1
DW 16.500 usec
DE 6.00 usec
TE 298.0 K
D1 2.00000000 sec
d11 0.03000000 sec
D16 0.00020000 sec
d17 0.00019600 sec
MCREST 0 sec
MCWRK 0.01500000 sec
P2 33.10 usec
======== CHANNEL f1 ========
NUC1 13C
P1 16.55 usec
P11 500.00 usec
P12 2000.00 usec
PL0 120.00 dB
PL1 -1.00 dB
SFO1 125.7942548 MHz
SP1 2.70 dB
SP2 2.70 dB
SPNAM[1] Crp60,0.5,20.1
SPNAM[2] Crp60comp.4
SPOFF1 0 Hz
SPOFF2 0 Hz
======== CHANNEL f2 ========
CPDPRG[2 waltz16
NUC2 1H
PCPD2 100.00 usec
PL2 1.60 dB
PL12 24.50 dB
SFO2 500.2225011 MHz
====== GRADIENT CHANNEL =====
GPNAM[1] SINE.100
GPNAM[2] SINE.100
GPX1 0 %
GPX2 0 %
GPY1 0 %
GPY2 0 %
GPZ1 30.00 %
GPZ2 50.00 %
p15 500.00 usec
p16 1000.00 usec
F2 - Processing parameters
SI 65536
SF 125.7804079 MHz
WDW EM
SSB 0
LB 1.00 Hz
GB 0
HO
H
H
26
140.34
123.32
64.94
59.83
42.94
42.50
41.93
37.79
34.59
33.89
33.58
28.84
23.17
22.99
19.50
220
210
200
190
180
170
160
150
140
130
120
110
100
90
80
70
60
50
40
30
20
10
ppm

```
Current Data Parameters
NAME      JC 2-104 top CeCl3
EXPNO                    1
PROCNO                   1

F2 - Acquisition Parameters
Date_             20161208
Time                 23.39
INSTRUM            cryo500
PROBHD    5 mm CPTCI 1H-
PULPROG               zg30
TD                   81728
SOLVENT              CDCl3
NS                       8
DS                       2
SWH               8012.820 Hz
FIDRES            0.098043 Hz
AQ               5.0998273 sec
RG                     6.3
DW                  62.400 usec
DE                    6.00 usec
TE                   298.0 K
D1              0.10000000 sec
MCREST                   0 sec
MCWRK           0.01500000 sec

======== CHANNEL f1 ========
NUC1                    1H
P1                    7.50 usec
PL1                   1.60 dB
SFO1           500.2235015 MHz

F2 - Processing parameters
SI                   65536
SF             500.2200317 MHz
WDW                     EM
SSB                      0
LB                    0.30 Hz
GB                       0
PC                    1.00
```

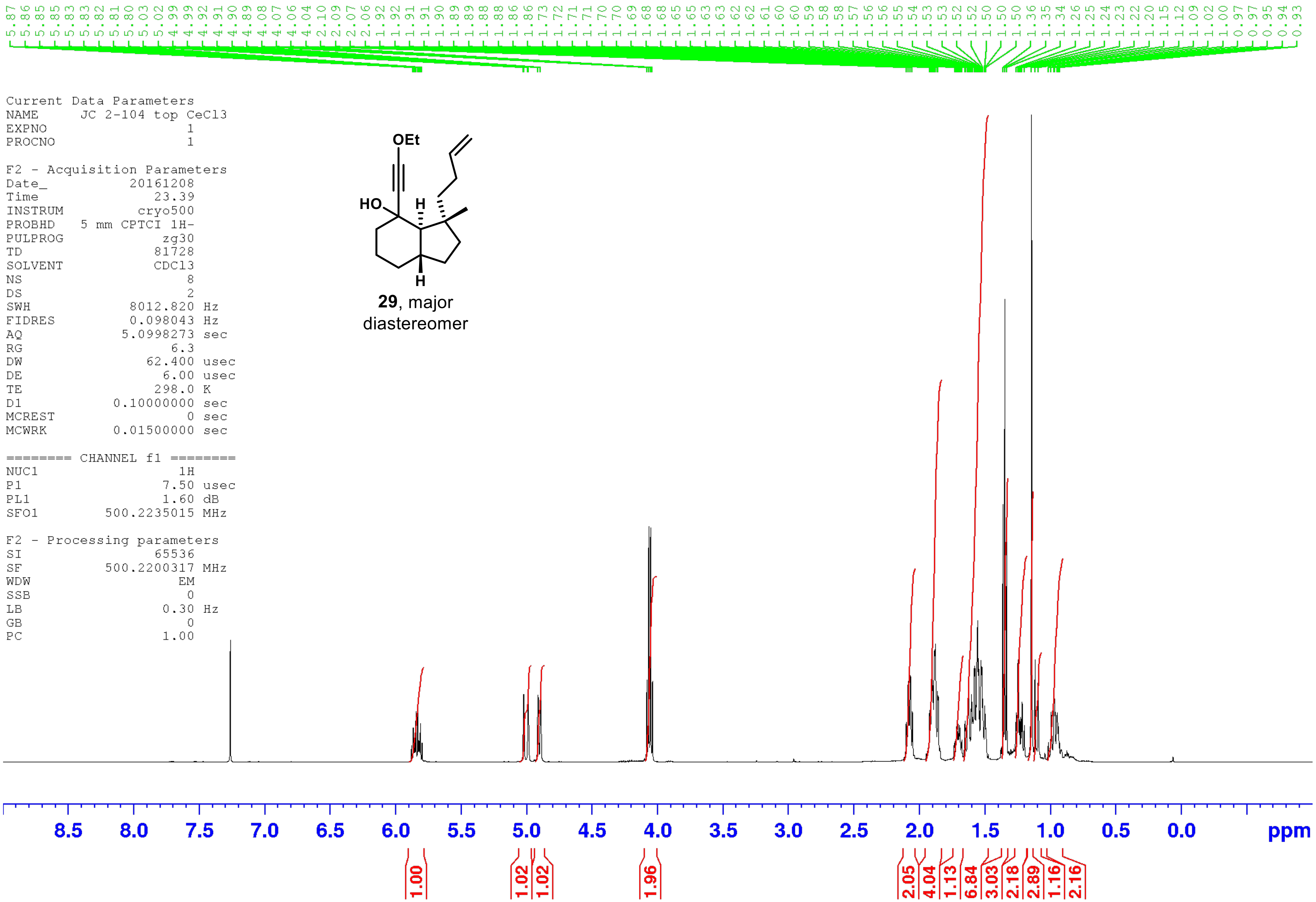

29, major diastereomer

```
Current Data Parameters
NAME      JC 2-104 top CeCl3
EXPNO              2
PROCNO             1

F2 - Acquisition Parameters
Date_          20161208
Time              23.42
INSTRUM         cryo500
PROBHD   5 mm CPTCI 1H-
PULPROG  SpinEchopg30gp.prd
TD                65536
SOLVENT           CDCl3
NS                   61
DS                   16
SWH           30303.031 Hz
FIDRES         0.462388 Hz
AQ            1.0813440 sec
RG               7298.2
DW               16.500 usec
DE                 6.00 usec
TE                298.0 K
D1           1.00000000 sec
d11          0.03000000 sec
D16          0.00020000 sec
d17          0.00019600 sec
MCREST                0 sec
MCWRK        0.01500000 sec
P2                33.10 usec

======== CHANNEL f1 ========
NUC1                13C
P1                16.55 usec
P11              500.00 usec
P12             2000.00 usec
PL0              120.00 dB
PL1               -1.00 dB
SFO1        125.7942548 MHz
SP1                2.70 dB
SP2                2.70 dB
SPNAM[1] Crp60,0.5,20.1
SPNAM[2]    Crp60comp.4
SPOFF1                0 Hz
SPOFF2                0 Hz

======== CHANNEL f2 ========
CPDPRG[2        waltz16
NUC2                 1H
PCPD2            100.00 usec
PL2                1.60 dB
PL12              24.50 dB
SFO2        500.2225011 MHz

====== GRADIENT CHANNEL =====
GPNAM[1]       SINE.100
GPNAM[2]       SINE.100
GPX1                  0 %
GPX2                  0 %
GPY1                  0 %
GPY2                  0 %
GPZ1              30.00 %
GPZ2              50.00 %
p15              500.00 usec
p16             1000.00 usec

F2 - Processing parameters
SI                65536
SF          125.7804084 MHz
WDW                  EM
SSB                   0
LB                 1.00 Hz
GB                    0
PC                 2.00
```

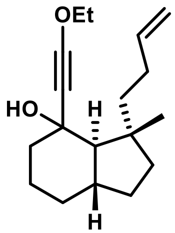

29, major diastereomer

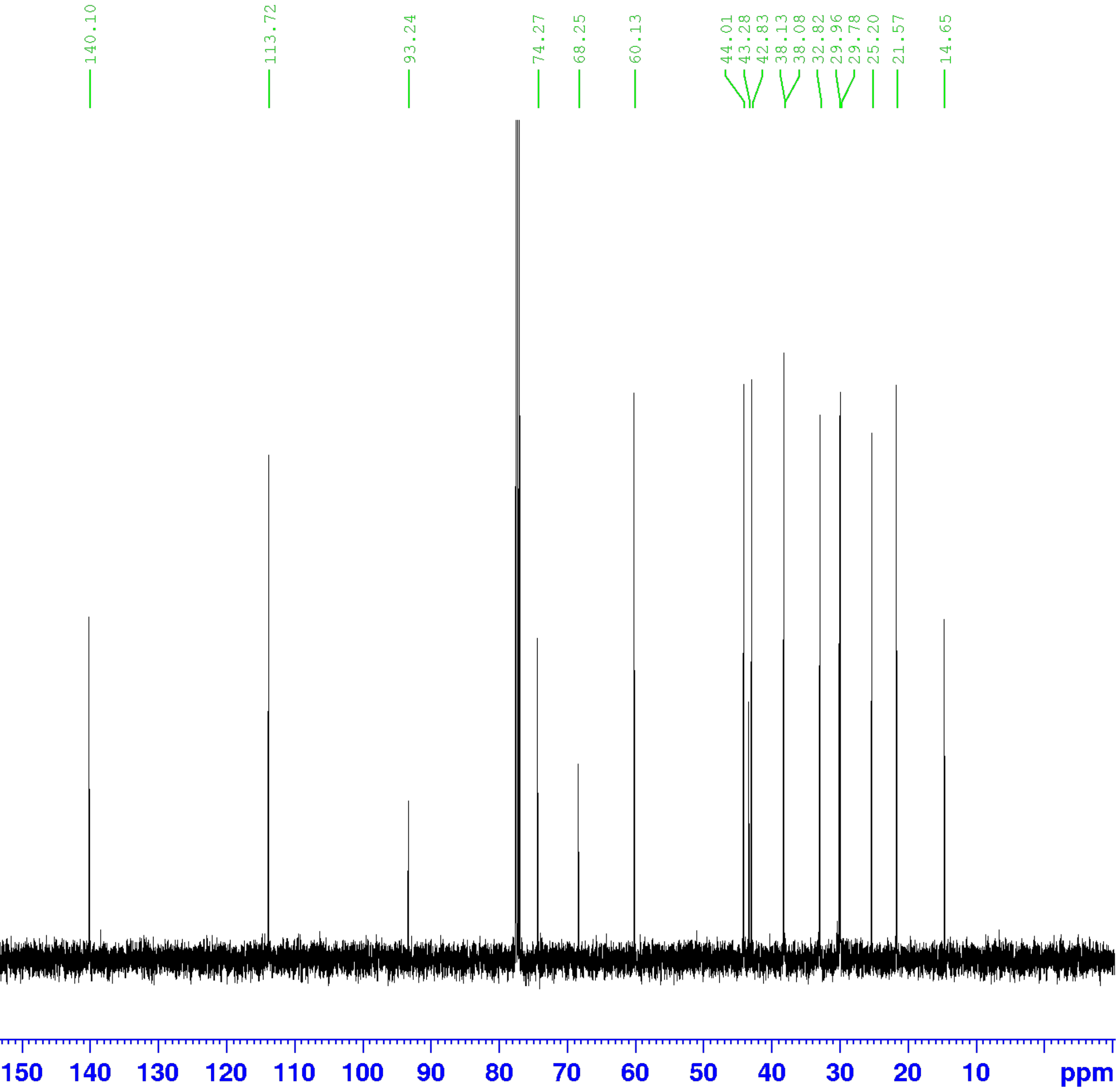

Current Data Parameters
NAME JC 2-104 bot CeCl3
EXPNO 1
PROCNO 1

F2 - Acquisition Parameters
Date_ 20161208
Time 23.56
INSTRUM cryo500
PROBHD 5 mm CPTCI 1H-
PULPROG zg30
TD 81728
SOLVENT CDCl3
NS 8
DS 2
SWH 8012.820 Hz
FIDRES 0.098043 Hz
AQ 5.0998273 sec
RG 5.7
DW 62.400 usec
DE 6.00 usec
TE 298.0 K
D1 0.10000000 sec
MCREST 0 sec
MCWRK 0.01500000 sec

======== CHANNEL f1 ========
NUC1 1H
P1 7.50 usec
PL1 1.60 dB
SFO1 500.2235015 MHz

F2 - Processing parameters
SI 65536
SF 500.2200319 MHz
WDW EM
SSB 0
LB 0.30 Hz
GB 0
PC 1.00

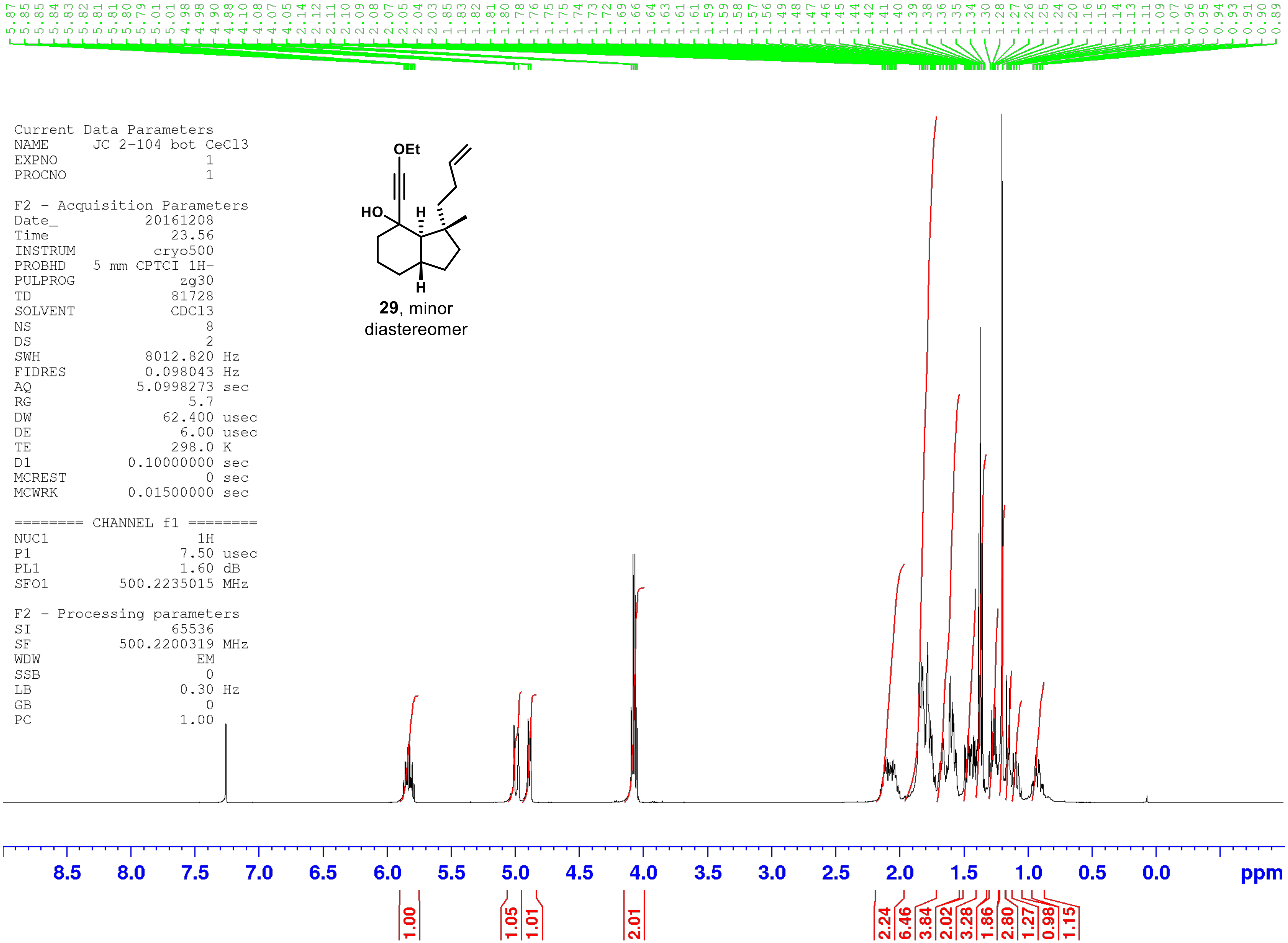

29, minor diastereomer

```
Current Data Parameters
NAME      JC 2-104 bot CeCl3
EXPNO              2
PROCNO             1

F2 - Acquisition Parameters
Date_          20161208
Time              23.59
INSTRUM        cryo500
PROBHD   5 mm CPTCI 1H-
PULPROG  SpinEchopg30gp.prd
TD                65536
SOLVENT           CDCl3
NS                   39
DS                   16
SWH          30303.031 Hz
FIDRES        0.462388 Hz
AQ           1.0813440 sec
RG                 6502
DW               16.500 usec
DE                 6.00 usec
TE                298.0 K
D1           1.00000000 sec
d11          0.03000000 sec
D16          0.00020000 sec
d17          0.00019600 sec
MCREST                0 sec
MCWRK        0.01500000 sec
P2                33.10 usec

======== CHANNEL f1 ========
NUC1                13C
P1                16.55 usec
P11              500.00 usec
P12             2000.00 usec
PL0              120.00 dB
PL1               -1.00 dB
SFO1        125.7942548 MHz
SP1                2.70 dB
SP2                2.70 dB
SPNAM[1] Crp60,0.5,20.1
SPNAM[2]    Crp60comp.4
SPOFF1                0 Hz
SPOFF2                0 Hz

======== CHANNEL f2 ========
CPDPRG[2        waltz16
NUC2                 1H
PCPD2            100.00 usec
PL2                1.60 dB
PL12              24.50 dB
SFO2        500.2225011 MHz

====== GRADIENT CHANNEL =====
GPNAM[1]       SINE.100
GPNAM[2]       SINE.100
GPX1                  0 %
GPX2                  0 %
GPY1                  0 %
GPY2                  0 %
GPZ1              30.00 %
GPZ2              50.00 %
p15              500.00 usec
p16             1000.00 usec

F2 - Processing parameters
SI                65536
SF          125.7804090 MHz
WDW                  EM
SSB                   0
LB                 1.00 Hz
GB                    0
PC                 2.00
```

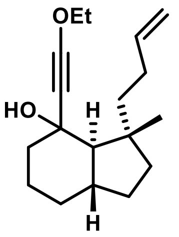

29, minor diastereomer

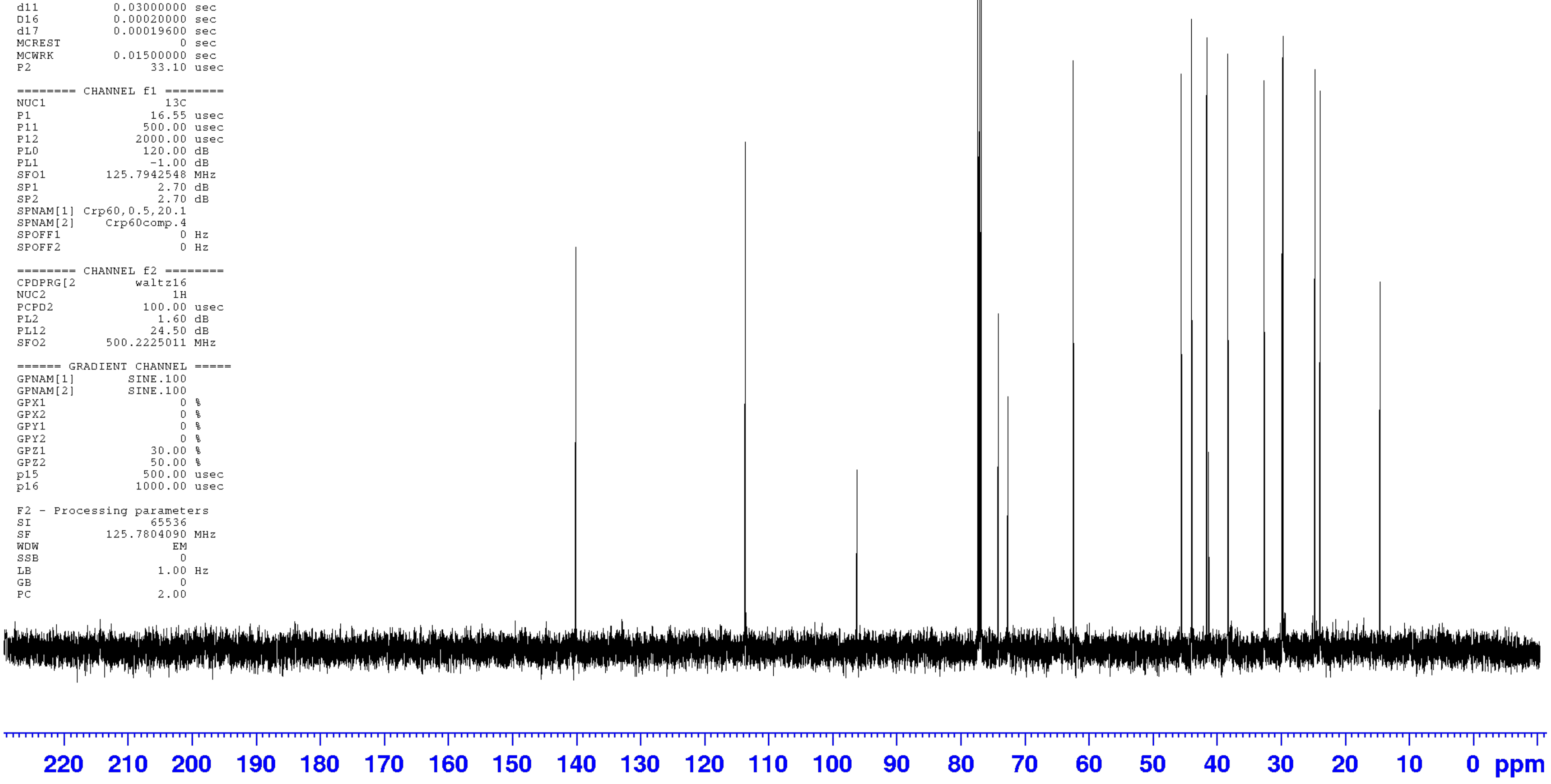

Current Data Parameters
NAME JC 2-110 top Sc(OTf)
EXPNO 1
PROCNO 1

F2 - Acquisition Parameters
Date_ 20161208
Time 23.28
INSTRUM cryo500
PROBHD 5 mm CPTCI 1H-
PULPROG zg30
TD 81728
SOLVENT CDCl3
NS 8
DS 2
SWH 8012.820 Hz
FIDRES 0.098043 Hz
AQ 5.0998273 sec
RG 6.3
DW 62.400 usec
DE 6.00 usec
TE 298.0 K
D1 0.10000000 sec
MCREST 0 sec
MCWRK 0.01500000 sec

======== CHANNEL f1 ========
NUC1 1H
P1 7.50 usec
PL1 1.60 dB
SFO1 500.2235015 MHz

F2 - Processing parameters
SI 65536
SF 500.2200317 MHz
WDW EM
SSB 0
LB 0.30 Hz
GB 0
PC 1.00

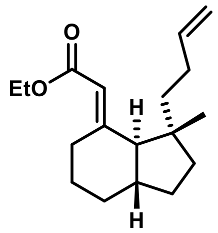

30, major diastereomer

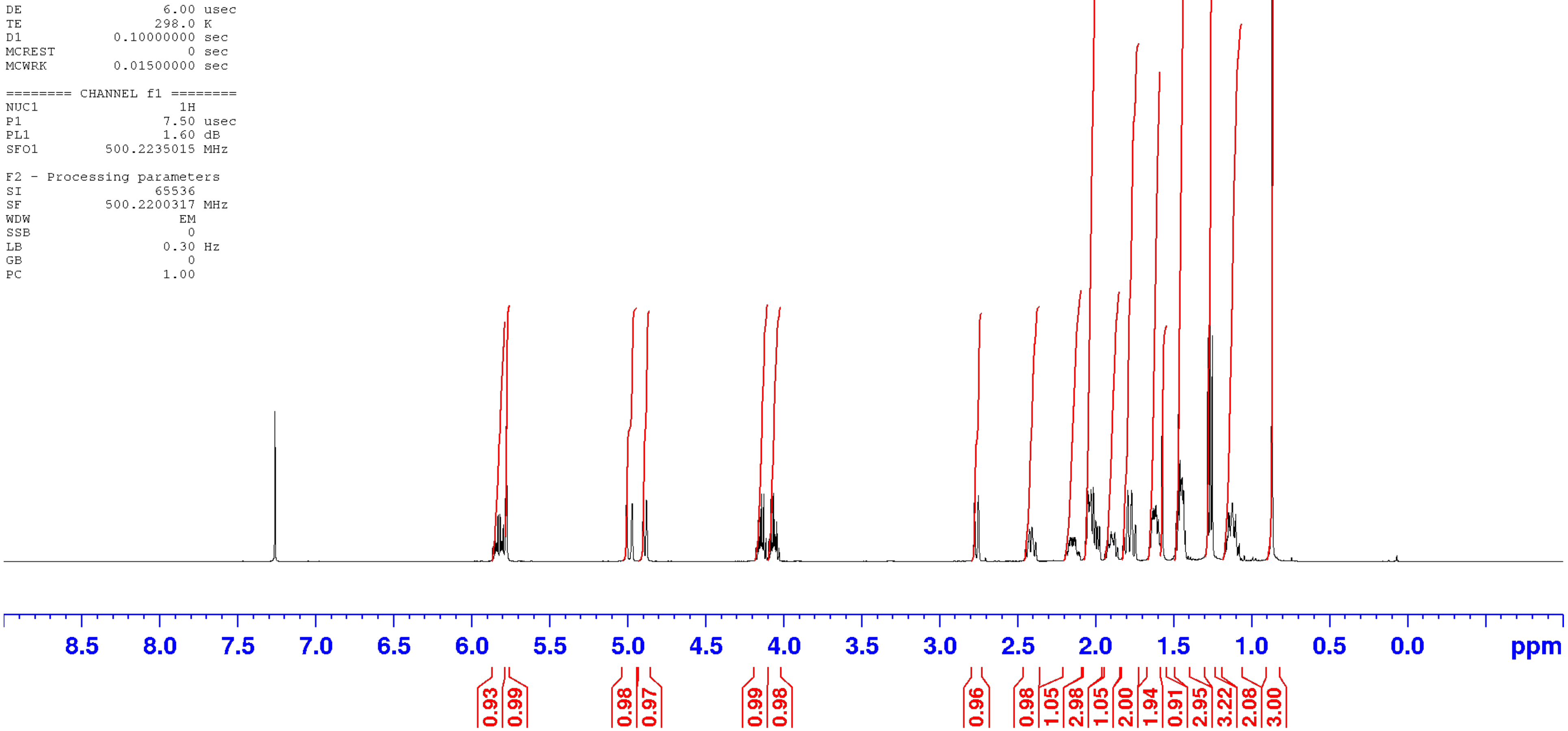

```
Current Data Parameters
NAME     JC 2-110 top Sc(OTf)
EXPNO               2
PROCNO              1

F2 - Acquisition Parameters
Date_          20161208
Time              23.31
INSTRUM         cryo500
PROBHD   5 mm CPTCI 1H-
PULPROG  SpinEchopg30gp.prd
TD                65536
SOLVENT           CDCl3
NS                   62
DS                   16
SWH           30303.031 Hz
FIDRES         0.462388 Hz
AQ            1.0813440 sec
RG                 4096
DW               16.500 usec
DE                 6.00 usec
TE                298.0 K
D1           1.00000000 sec
d11          0.03000000 sec
D16          0.00020000 sec
d17          0.00019600 sec
MCREST                0 sec
MCWRK        0.01500000 sec
P2                33.10 usec

======== CHANNEL f1 ========
NUC1                13C
P1                16.55 usec
P11              500.00 usec
P12             2000.00 usec
PL0              120.00 dB
PL1               -1.00 dB
SFO1        125.7942548 MHz
SP1                2.70 dB
SP2                2.70 dB
SPNAM[1] Crp60,0.5,20.1
SPNAM[2]    Crp60comp.4
SPOFF1                0 Hz
SPOFF2                0 Hz

======== CHANNEL f2 ========
CPDPRG[2        waltz16
NUC2                 1H
PCPD2            100.00 usec
PL2                1.60 dB
PL12              24.50 dB
SFO2        500.2225011 MHz

====== GRADIENT CHANNEL =====
GPNAM[1]       SINE.100
GPNAM[2]       SINE.100
GPX1                  0 %
GPX2                  0 %
GPY1                  0 %
GPY2                  0 %
GPZ1              30.00 %
GPZ2              50.00 %
p15              500.00 usec
p16             1000.00 usec

F2 - Processing parameters
SI                65536
SF          125.7804081 MHz
WDW                  EM
SSB                   0
LB                 1.00 Hz
GB                    0
PC                 2.00
```

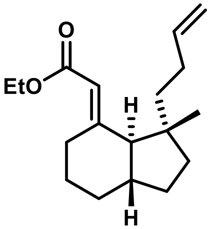

30, major diastereomer

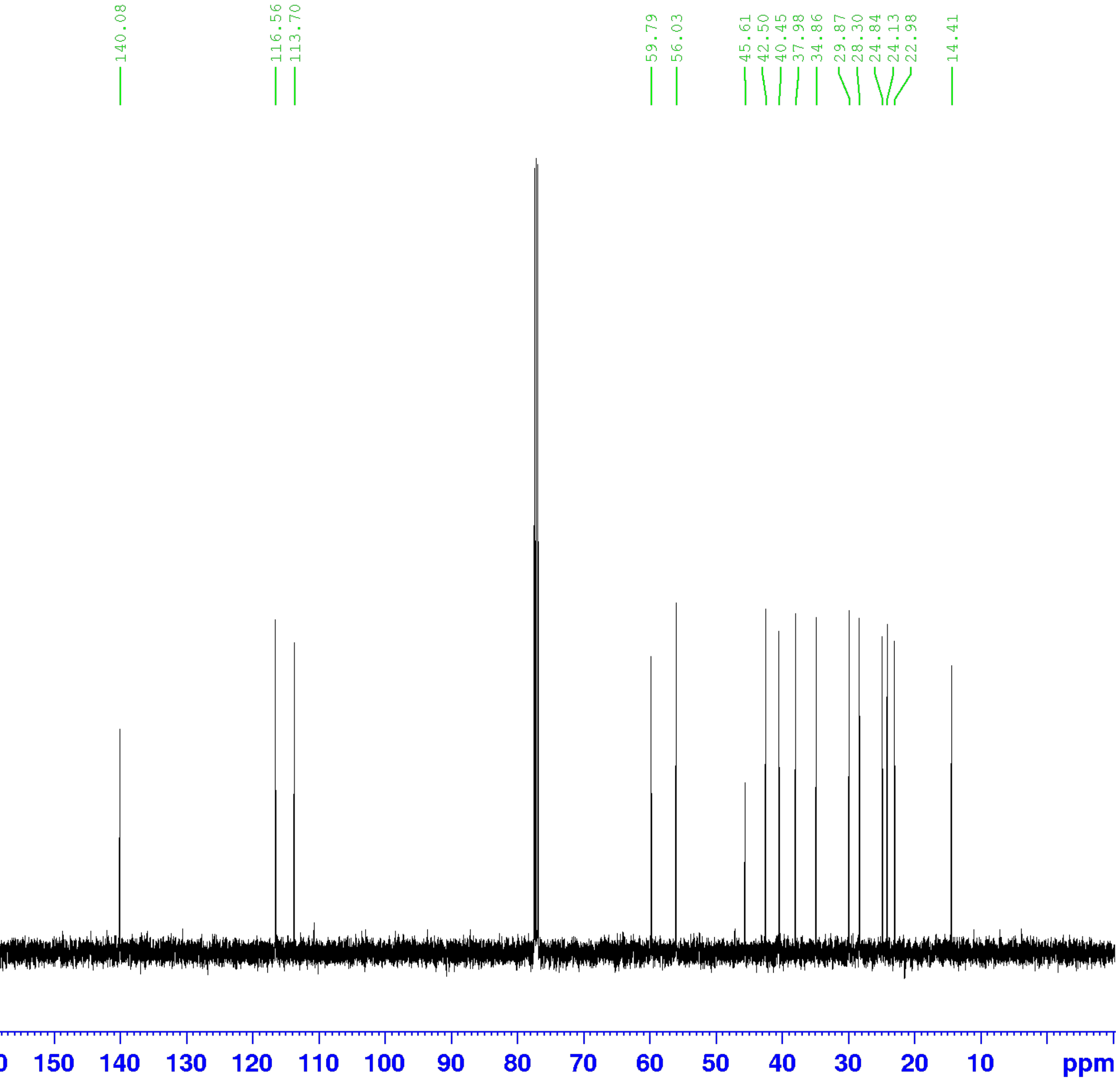

Current Data Parameters
NAME JC 2-110 bot Sc(OTf)
EXPNO 1
PROCNO 1

F2 - Acquisition Parameters
Date_ 20161208
Time 23.15
INSTRUM cryo500
PROBHD 5 mm CPTCI 1H-
PULPROG zg30
TD 81728
SOLVENT CDCl3
NS 8
DS 2
SWH 8012.820 Hz
FIDRES 0.098043 Hz
AQ 5.0998273 sec
RG 6.3
DW 62.400 usec
DE 6.00 usec
TE 298.0 K
D1 0.10000000 sec
MCREST 0 sec
MCWRK 0.01500000 sec

======== CHANNEL f1 ========
NUC1 1H
P1 7.50 usec
PL1 1.60 dB
SFO1 500.2235015 MHz

F2 - Processing parameters
SI 65536
SF 500.2200322 MHz
WDW EM
SSB 0
LB 0.30 Hz
GB 0
PC 1.00

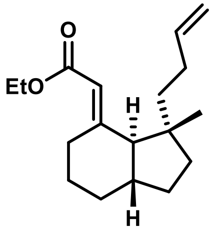

30, minor diastereomer

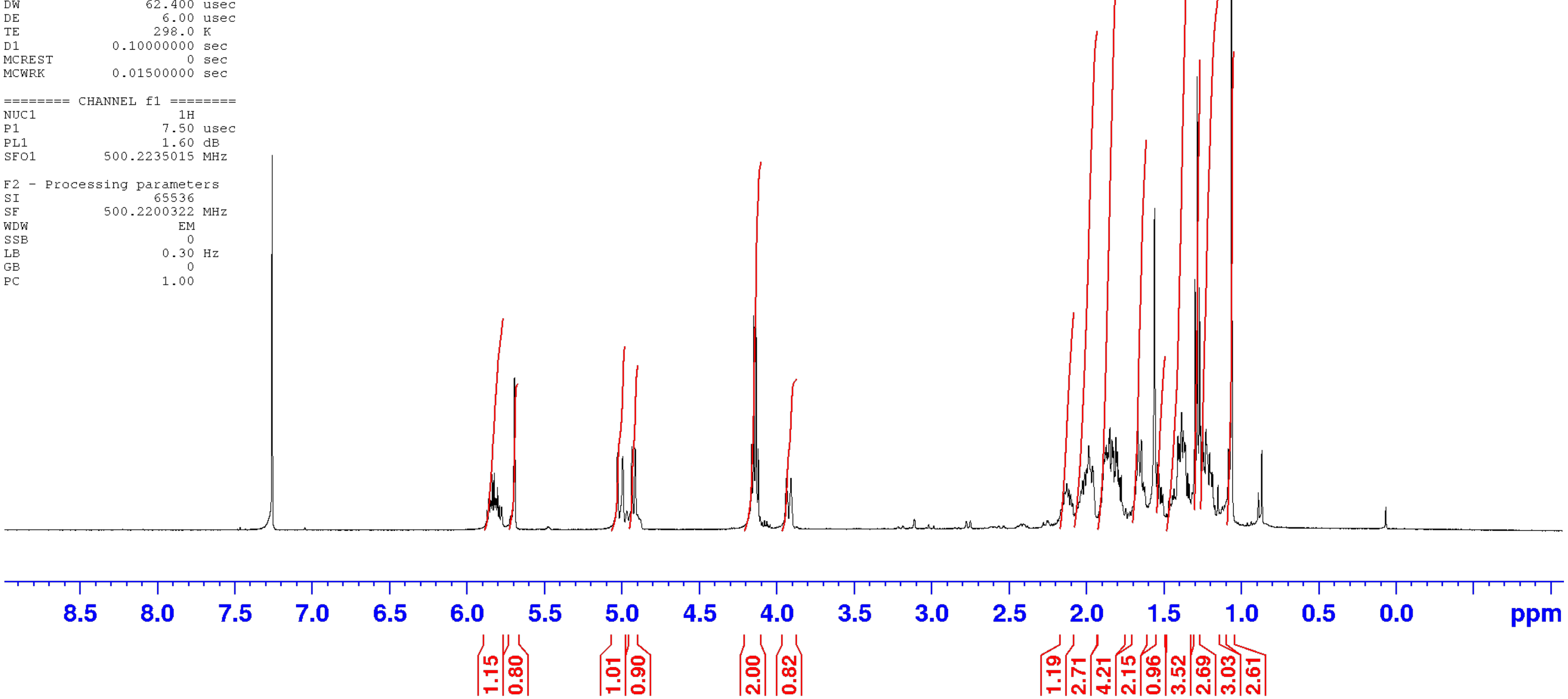

```
Current Data Parameters
NAME      JC 2-110 bot Sc(OTf)
EXPNO                    2
PROCNO                   1

F2 - Acquisition Parameters
Date_           20161208
Time               23.19
INSTRUM          cryo500
PROBHD   5 mm CPTCI 1H-
PULPROG  SpinEchopg30gp.prd
TD                 65536
SOLVENT            CDCl3
NS                    60
DS                    16
SWH            30303.031 Hz
FIDRES          0.462388 Hz
AQ             1.0813440 sec
RG                  8192
DW                16.500 usec
DE                  6.00 usec
TE                 298.0 K
D1            1.00000000 sec
d11           0.03000000 sec
D16           0.00020000 sec
d17           0.00019600 sec
MCREST                 0 sec
MCWRK         0.01500000 sec
P2                 33.10 usec

======== CHANNEL f1 ========
NUC1                 13C
P1                 16.55 usec
P11               500.00 usec
P12              2000.00 usec
PL0               120.00 dB
PL1                -1.00 dB
SFO1         125.7942548 MHz
SP1                 2.70 dB
SP2                 2.70 dB
SPNAM[1] Crp60,0.5,20.1
SPNAM[2]     Crp60comp.4
SPOFF1                 0 Hz
SPOFF2                 0 Hz

======== CHANNEL f2 ========
CPDPRG[2         waltz16
NUC2                  1H
PCPD2             100.00 usec
PL2                 1.60 dB
PL12               24.50 dB
SFO2         500.2225011 MHz

====== GRADIENT CHANNEL =====
GPNAM[1]        SINE.100
GPNAM[2]        SINE.100
GPX1                   0 %
GPX2                   0 %
GPY1                   0 %
GPY2                   0 %
GPZ1               30.00 %
GPZ2               50.00 %
p15               500.00 usec
p16              1000.00 usec

F2 - Processing parameters
SI                 65536
SF           125.7804079 MHz
WDW                   EM
SSB                    0
LB                  1.00 Hz
GB                     0
PC                  2.00
```

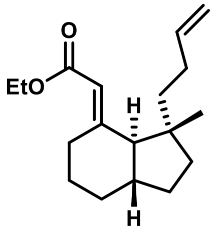

30, minor diastereomer

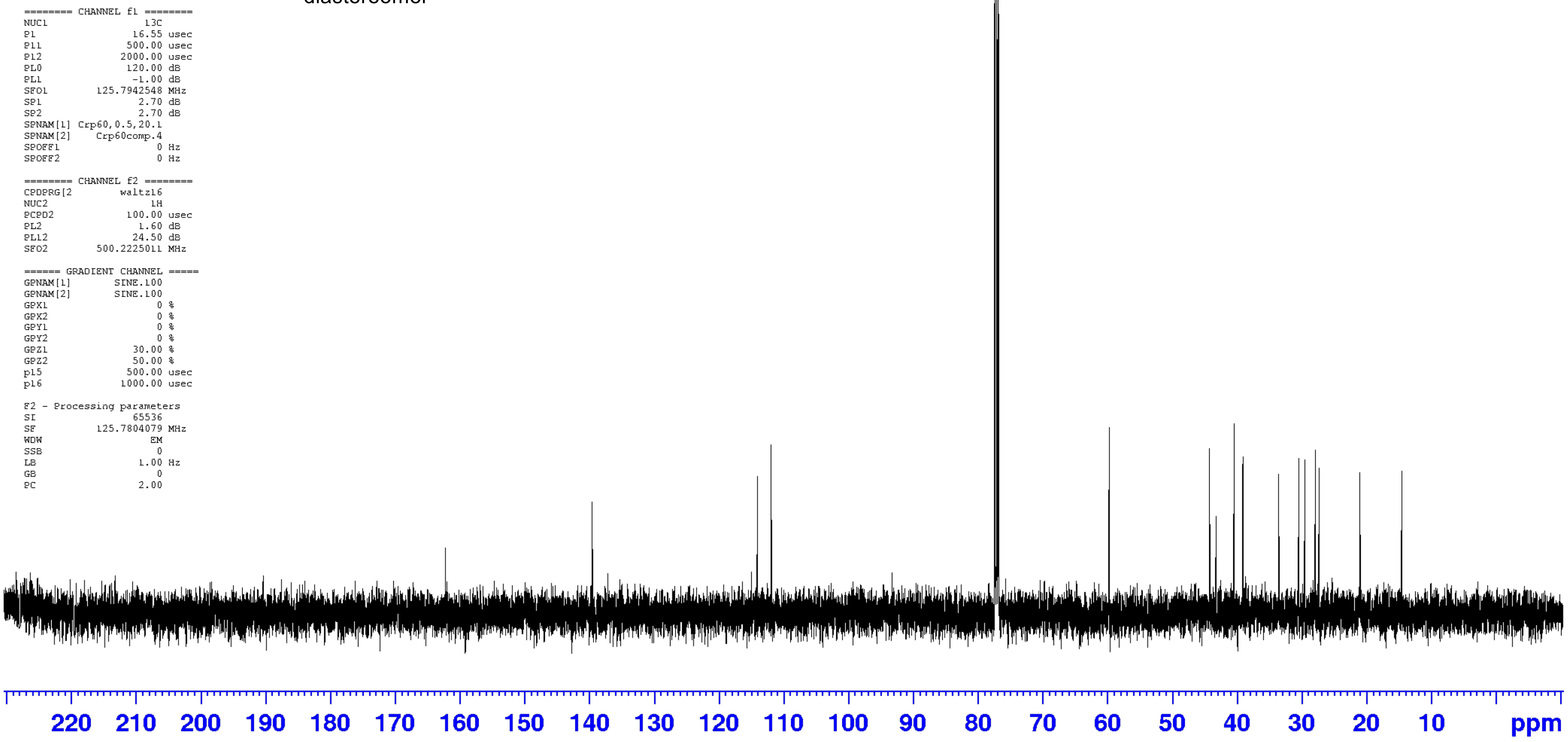

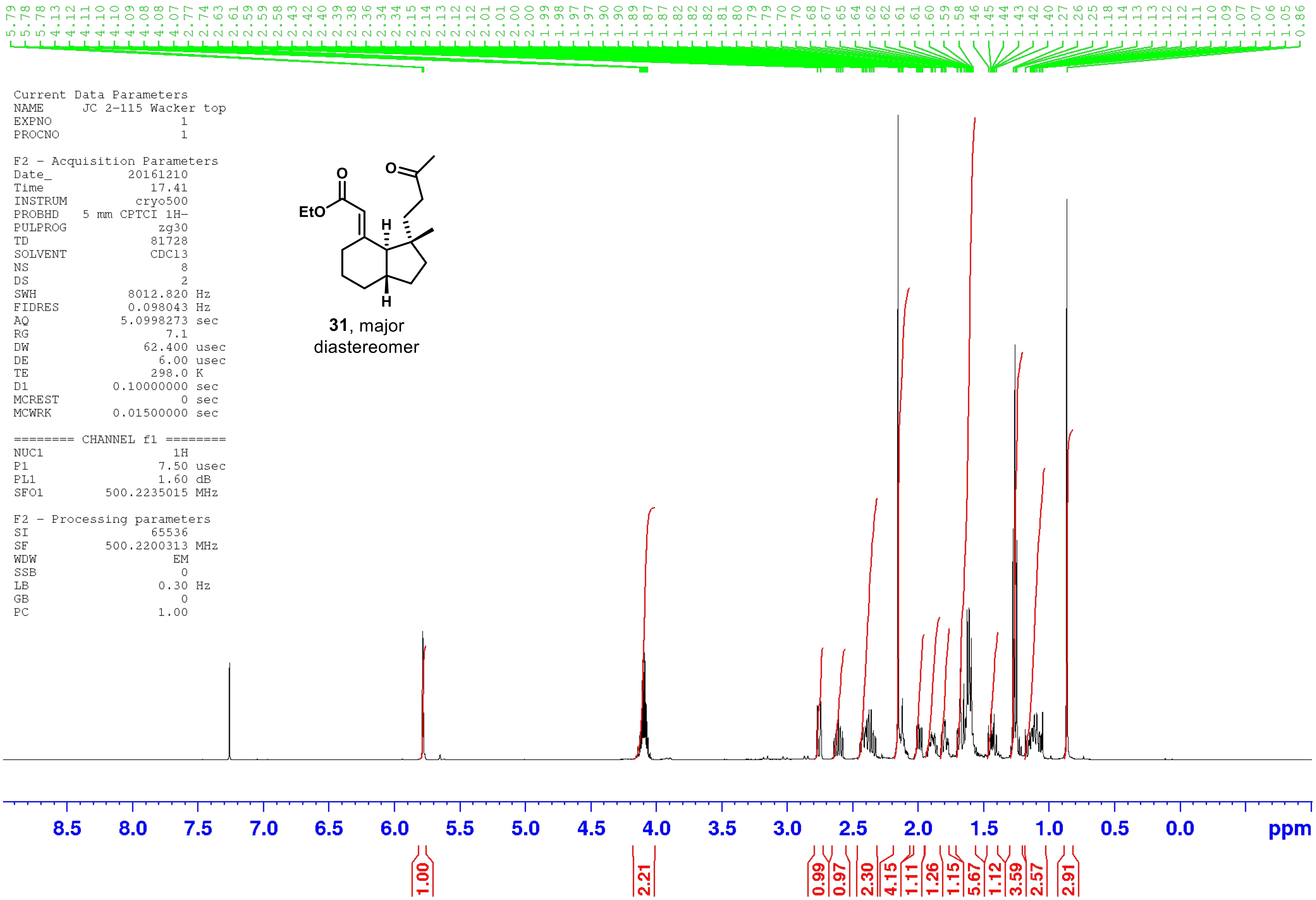
5.79 5.78 5.78 4.13 4.12 4.11 4.10 4.10 4.09 4.08 4.08 4.07 2.77 2.74 2.63 2.61 2.59 2.59 2.58 2.43 2.42 2.40 2.39 2.38 2.36 2.34 2.15 2.14 2.13 2.12 2.12 2.01 2.01 2.00 2.00 1.99 1.98 1.97 1.97 1.90 1.90 1.89 1.87 1.87 1.82 1.82 1.81 1.80 1.79 1.79 1.70 1.70 1.68 1.67 1.65 1.64 1.62 1.62 1.61 1.61 1.60 1.59 1.58 1.46 1.45 1.44 1.43 1.42 1.40 1.27 1.26 1.25 1.18 1.14 1.13 1.12 1.12 1.11 1.10 1.09 1.07 1.07 1.06 1.05 0.86
Current Data Parameters
NAME JC 2-115 Wacker top
EXPNO 1
PROCNO 1
F2 - Acquisition Parameters
Date_ 20161210
Time 17.41
INSTRUM cryo500
PROBHD 5 mm CPTCI 1H-
PULPROG zg30
TD 81728
SOLVENT CDCl3
NS 8
DS 2
SWH 8012.820 Hz
FIDRES 0.098043 Hz
AQ 5.0998273 sec
RG 7.1
DW 62.400 usec
DE 6.00 usec
TE 298.0 K
D1 0.10000000 sec
MCREST 0 sec
MCWRK 0.01500000 sec
======== CHANNEL f1 ========
NUC1 1H
P1 7.50 usec
PL1 1.60 dB
SFO1 500.2235015 MHz
F2 - Processing parameters
SI 65536
SF 500.2200313 MHz
WDW EM
SSB 0
LB 0.30 Hz
GB 0
PC 1.00
O
O
EtO
H
H
31, major diastereomer
8.5 8.0 7.5 7.0 6.5 6.0 5.5 5.0 4.5 4.0 3.5 3.0 2.5 2.0 1.5 1.0 0.5 0.0 ppm
1.00 2.21 0.99 0.97 2.30 4.15 1.11 1.26 1.15 5.67 1.12 3.59 2.57 2.91

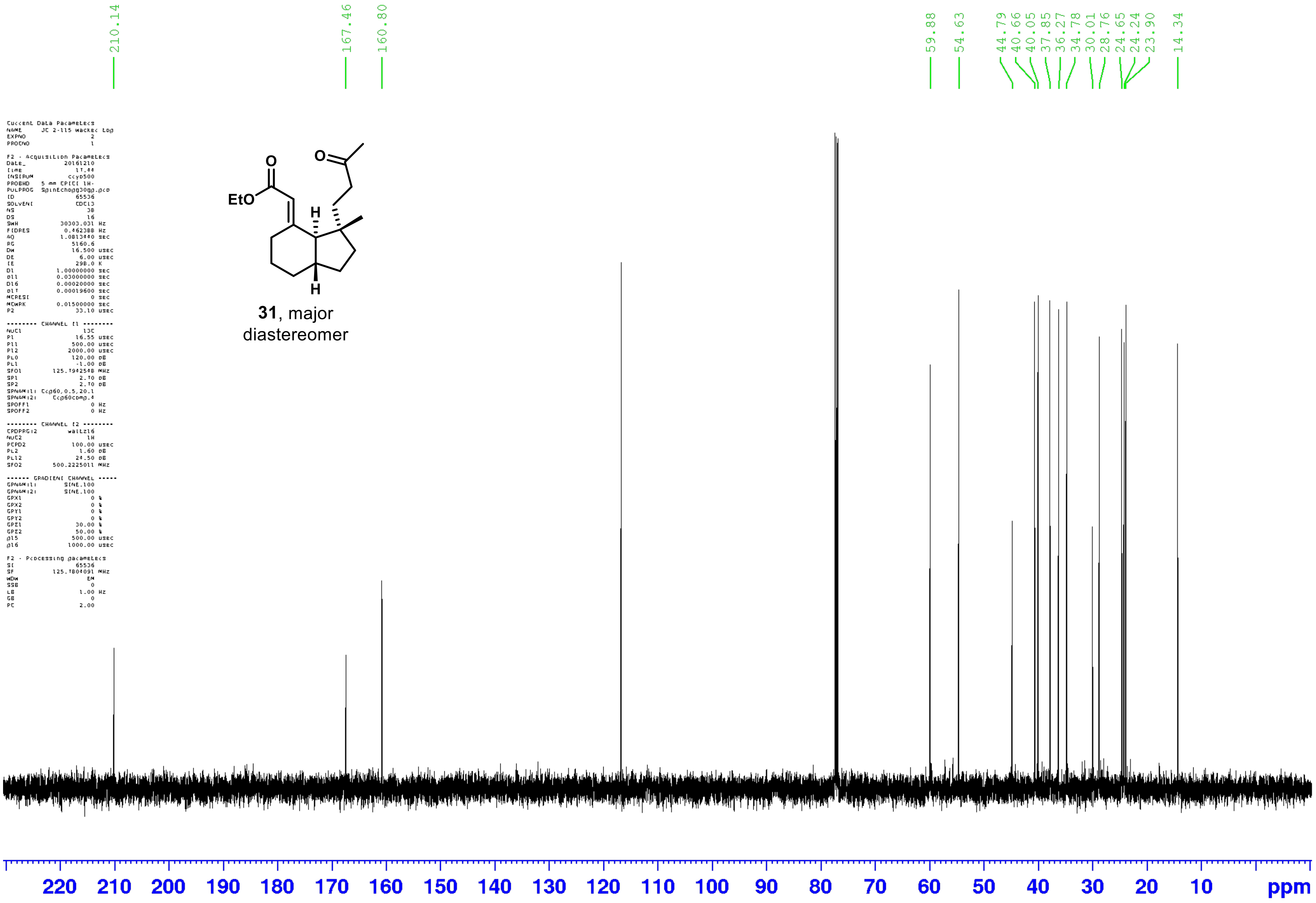

31, major diastereomer

```
Current Data Parameters
NAME      JC 2-115 Wacker bot
EXPNO                   1
PROCNO                  1

F2 - Acquisition Parameters
Date_            20161210
Time                17.32
INSTRUM           cryo500
PROBHD   5 mm CPTCI 1H-
PULPROG              zg30
TD                  81728
SOLVENT             CDCl3
NS                      8
DS                      2
SWH              8012.820 Hz
FIDRES           0.098043 Hz
AQ              5.0998273 sec
RG                    6.3
DW                 62.400 usec
DE                   6.00 usec
TE                  298.0 K
D1             0.10000000 sec
MCREST                  0 sec
MCWRK          0.01500000 sec

======== CHANNEL f1 ========
NUC1                   1H
P1                   7.50 usec
PL1                  1.60 dB
SFO1          500.2235015 MHz

F2 - Processing parameters
SI                  65536
SF            500.2200318 MHz
WDW                    EM
SSB                     0
LB                   0.30 Hz
GB                      0
PC                   1.00
```

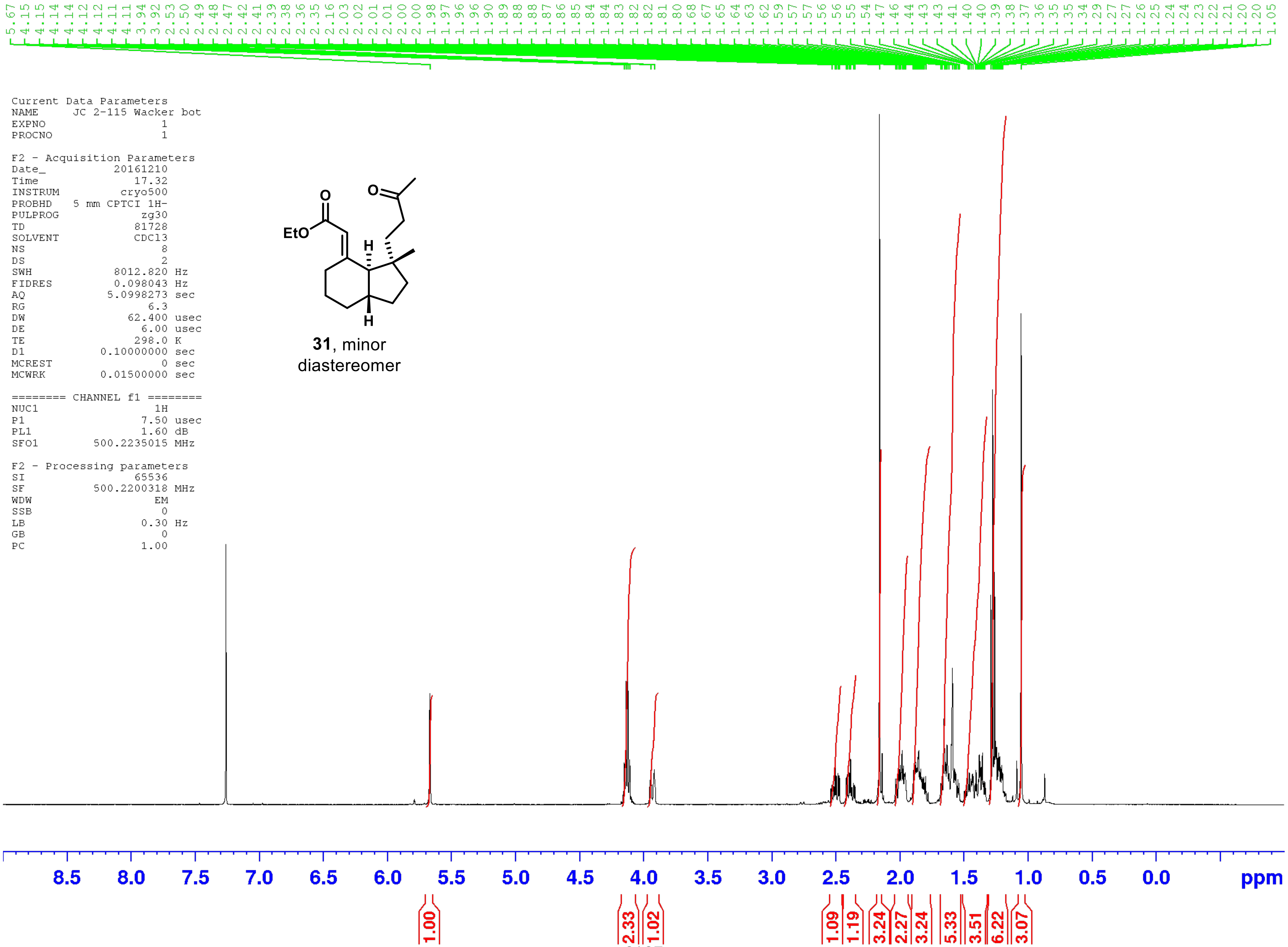

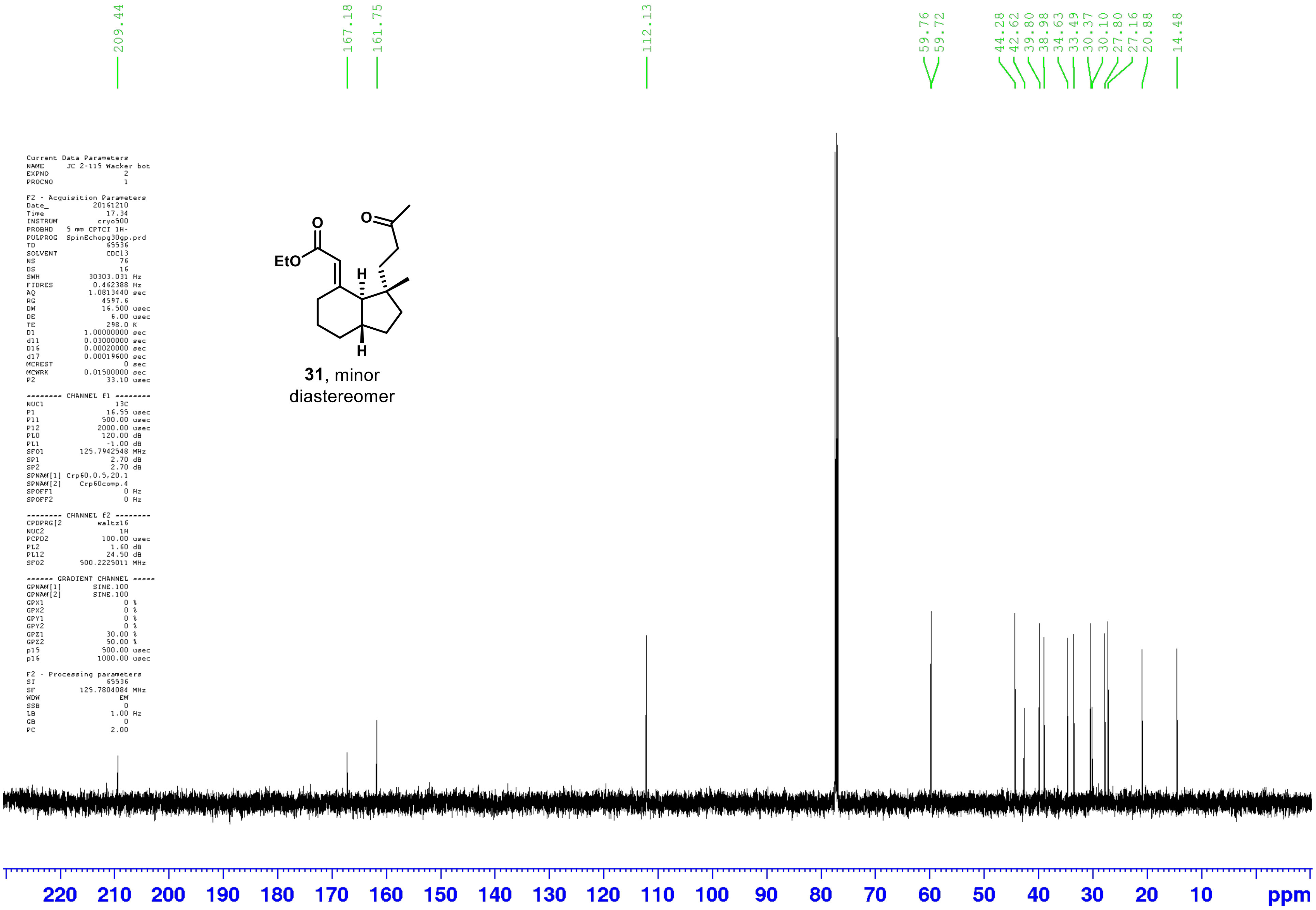
209.44
167.18
161.75
112.13
59.76
59.72
44.28
42.62
39.80
38.98
34.63
33.49
30.37
30.10
27.80
27.16
20.88
14.48
Current Data Parameters
NAME JC 2-115 Wacker bot
EXPNO 2
PROCNO 1
F2 - Acquisition Parameters
Date_ 20161210
Time 17.34
INSTRUM cryo500
PROBHD 5 mm CPTCI 1H-
PULPROG SpinEchopg30gp.prd
TD 65536
SOLVENT CDCl3
NS 76
DS 16
SWH 30303.031 Hz
FIDRES 0.462388 Hz
AQ 1.0813440 sec
RG 4597.6
DW 16.500 usec
DE 6.00 usec
TE 298.0 K
D1 1.00000000 sec
d11 0.03000000 sec
D16 0.00020000 sec
d17 0.00019600 sec
MCREST 0 sec
MCWRK 0.01500000 sec
P2 33.10 usec
-------- CHANNEL f1 --------
NUC1 13C
P1 16.55 usec
P11 500.00 usec
P12 2000.00 usec
PL0 120.00 dB
PL1 -1.00 dB
SFO1 125.7942548 MHz
SP1 2.70 dB
SP2 2.70 dB
SPNAM[1] Crp60,0.5,20.1
SPNAM[2] Crp60comp.4
SPOFF1 0 Hz
SPOFF2 0 Hz
-------- CHANNEL f2 --------
CPDPRG[2 waltz16
NUC2 1H
PCPD2 100.00 usec
PL2 1.60 dB
PL12 24.50 dB
SFO2 500.2225011 MHz
------ GRADIENT CHANNEL -----
GPNAM[1] SINE.100
GPNAM[2] SINE.100
GPX1 0 %
GPX2 0 %
GPY1 0 %
GPY2 0 %
GPZ1 30.00 %
GPZ2 50.00 %
p15 500.00 usec
p16 1000.00 usec
F2 - Processing parameters
SI 65536
SF 125.7804084 MHz
WDW EM
SSB 0
LB 1.00 Hz
GB 0
PC 2.00
EtO
O
O
H
H
31, minor
diastereomer
220
210
200
190
180
170
160
150
140
130
120
110
100
90
80
70
60
50
40
30
20
10
ppm

Current Data Parameters
NAME JC 2-133 SmI2 140 mg
EXPNO 1
PROCNO 1

F2 - Acquisition Parameters
Date_ 20161219
Time 23.08
INSTRUM cryo500
PROBHD 5 mm CPTCI 1H-
PULPROG zg30
TD 81728
SOLVENT CDCl3
NS 8
DS 2
SWH 8012.820 Hz
FIDRES 0.098043 Hz
AQ 5.0998273 sec
RG 6.3
DW 62.400 usec
DE 6.00 usec
TE 298.0 K
D1 0.10000000 sec
MCREST 0 sec
MCWRK 0.01500000 sec

======== CHANNEL f1 ========
NUC1 1H
P1 7.50 usec
PL1 1.60 dB
SFO1 500.2235015 MHz

F2 - Processing parameters
SI 65536
SF 500.2200318 MHz
WDW EM
SSB 0
LB 0.30 Hz
GB 0
PC 1.00

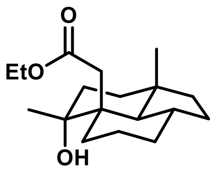

32

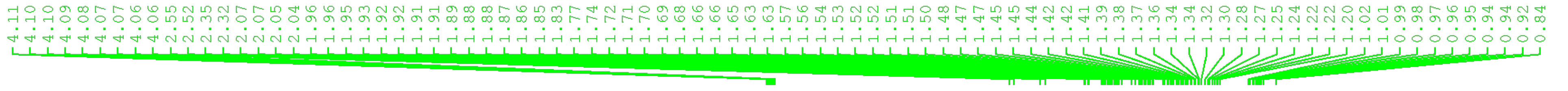

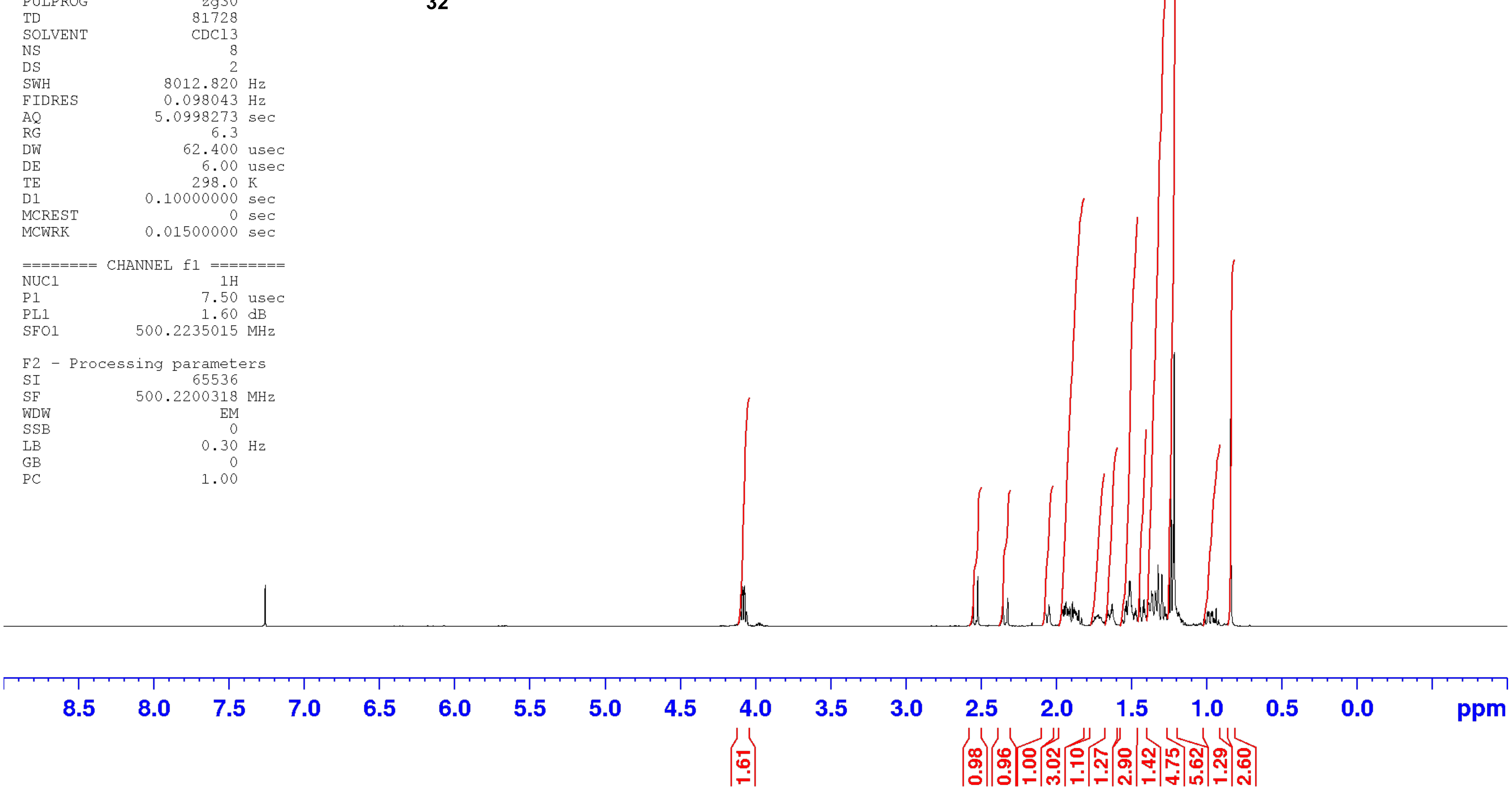

```
Current Data Parameters
NAME      JC 2-133 SmI2 140 mg
EXPNO                2
PROCNO               1

F2 - Acquisition Parameters
Date_         20161219
Time             23.10
INSTRUM        cryo500
PROBHD    5 mm CPTCI 1H-
PULPROG  SpinEchopg30gp.prd
TD               65536
SOLVENT          CDC13
NS                  51
DS                  16
SWH          30303.031 Hz
FIDRES        0.462388 Hz
AQ           1.0813440 sec
RG              7298.2
DW              16.500 usec
DE                6.00 usec
TE               298.0 K
D1          0.25000000 sec
d11         0.03000000 sec
D16         0.00020000 sec
d17         0.00019600 sec
MCREST               0 sec
MCWRK       0.01500000 sec
P2               33.10 usec

======== CHANNEL f1 ========
NUC1               13C
P1               16.55 usec
P11             500.00 usec
P12            2000.00 usec
PL0             120.00 dB
PL1              -1.00 dB
SFO1       125.7942548 MHz
SP1               2.70 dB
SP2               2.70 dB
SPNAM[1] Crp60,0.5,20.1
SPNAM[2]     Crp60comp.4
SPOFF1               0 Hz
SPOFF2               0 Hz

======== CHANNEL f2 ========
CPDPRG[2         waltz16
NUC2                  1H
PCPD2           100.00 usec
PL2               1.60 dB
PL12             24.50 dB
SFO2       500.2225011 MHz

====== GRADIENT CHANNEL =====
GPNAM[1]        SINE.100
GPNAM[2]        SINE.100
GPX1                0 %
GPX2                0 %
GPY1                0 %
GPY2                0 %
GPZ1            30.00 %
GPZ2            50.00 %
p15            500.00 usec
p16           1000.00 usec

F2 - Processing parameters
SI               65536
SF         125.7804084 MHz
WDW                 EM
SSB                  0
LB                1.00 Hz
GB                   0
PC                2.00
```

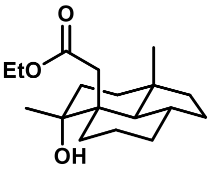

32

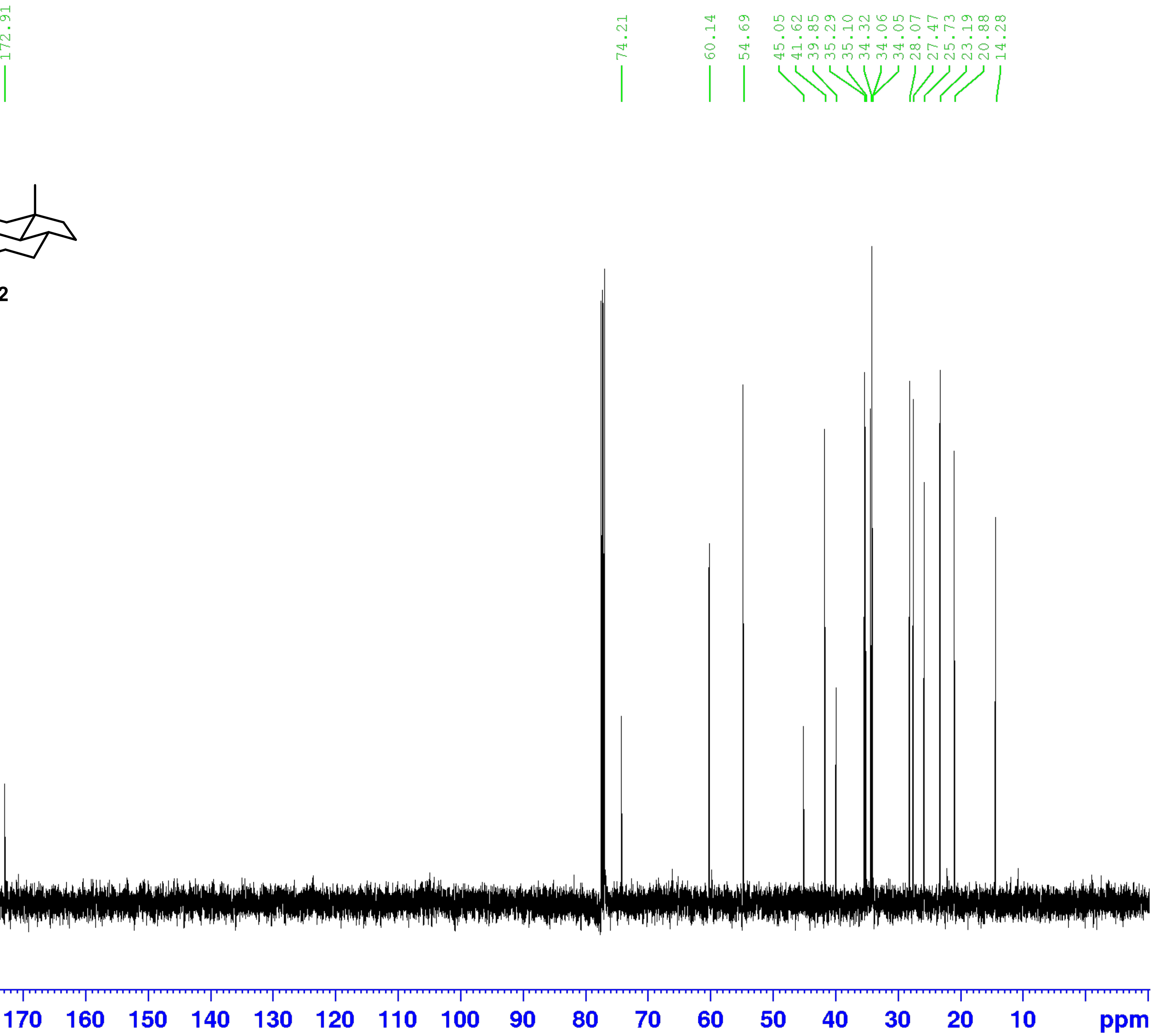

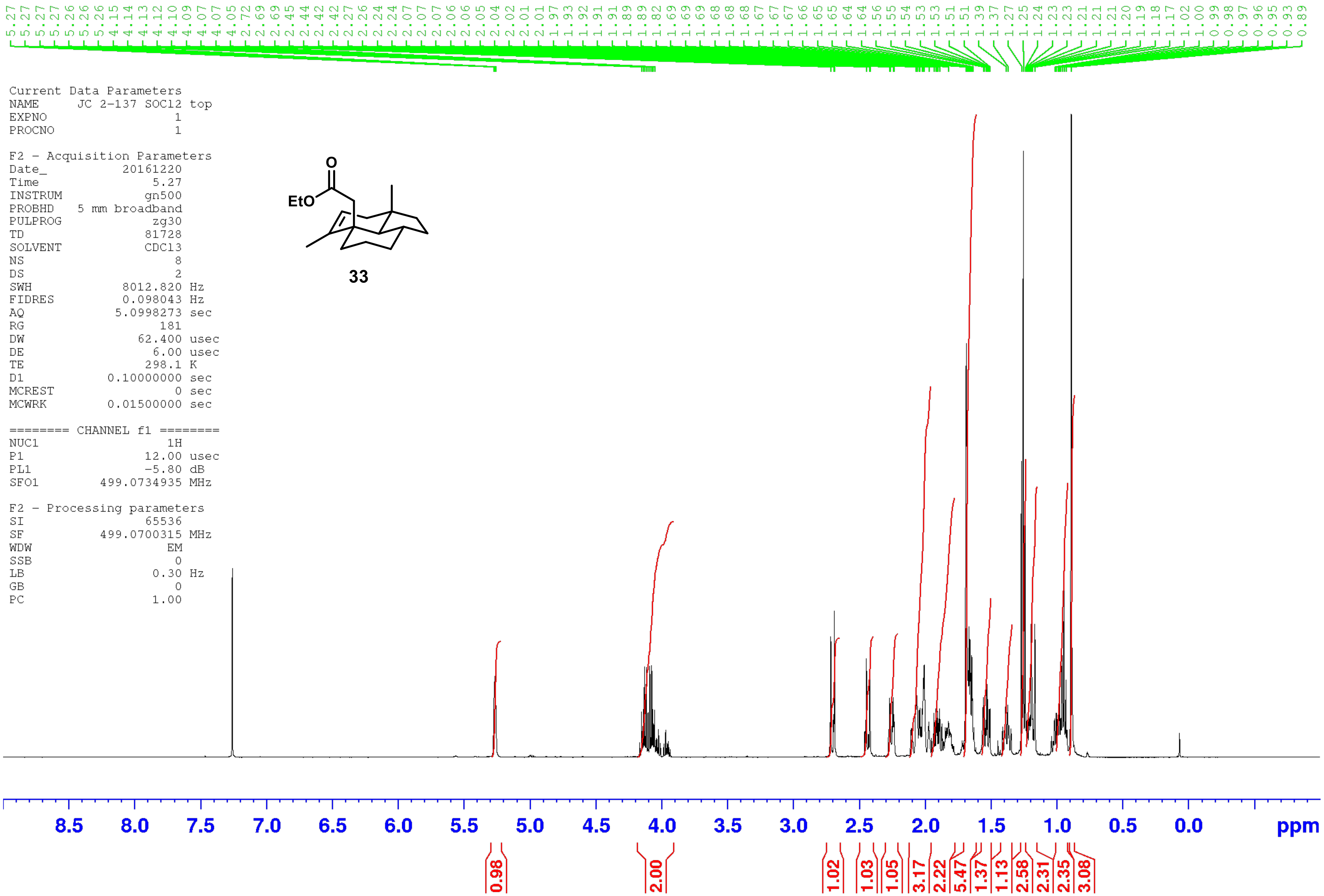
Current Data Parameters
NAME JC 2-137 SOCl2 top
EXPNO 1
PROCNO 1
F2 - Acquisition Parameters
Date_ 20161220
Time 5.27
INSTRUM gn500
PROBHD 5 mm broadband
PULPROG zg30
TD 81728
SOLVENT CDCl3
NS 8
DS 2
SWH 8012.820 Hz
FIDRES 0.098043 Hz
AQ 5.0998273 sec
RG 181
DW 62.400 usec
DE 6.00 usec
TE 298.1 K
D1 0.10000000 sec
MCREST 0 sec
MCWRK 0.01500000 sec
======== CHANNEL f1 ========
NUC1 1H
P1 12.00 usec
PL1 -5.80 dB
SFO1 499.0734935 MHz
F2 - Processing parameters
SI 65536
SF 499.0700315 MHz
WDW EM
SSB 0
LB 0.30 Hz
GB 0
PC 1.00
O
EtO
33
8.5 8.0 7.5 7.0 6.5 6.0 5.5 5.0 4.5 4.0 3.5 3.0 2.5 2.0 1.5 1.0 0.5 0.0
ppm
0.98 2.00 1.02 1.03 1.05 3.17 2.22 5.47 1.37 1.13 2.58 2.31 2.35 3.08

Current Data Parameters
NAME JC 2-137 SOCl2 top
EXPNO 2
PROCNO 1

F2 - Acquisition Parameters
Date_ 20161220
Time 5.24
INSTRUM gn500
PROBHD 5 mm broadband
PULPROG zgdc30
TD 65536
SOLVENT CDCl3
NS 42
DS 4
SWH 30303.031 Hz
FIDRES 0.462388 Hz
AQ 1.0813440 sec
RG 14596.5
DW 16.500 usec
DE 4.50 usec
TE 298.1 K
D1 2.00000000 sec
d11 0.03000000 sec
MCREST 0 sec
MCWRK 0.01500000 sec

======== CHANNEL f1 ========
NUC1 13C
P1 9.00 usec
PL1 -0.60 dB
SFO1 125.5050550 MHz

======== CHANNEL f2 ========
CPDPRG[2 waltz16
NUC2 1H
PCPD2 80.00 usec
PL2 -3.00 dB
PL12 12.80 dB
SFO2 499.0724953 MHz

F2 - Processing parameters
SI 65536
SF 125.4912384 MHz
WDW EM
SSB 0
LB 1.00 Hz
GB 0
PC 2.00

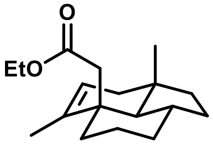

33

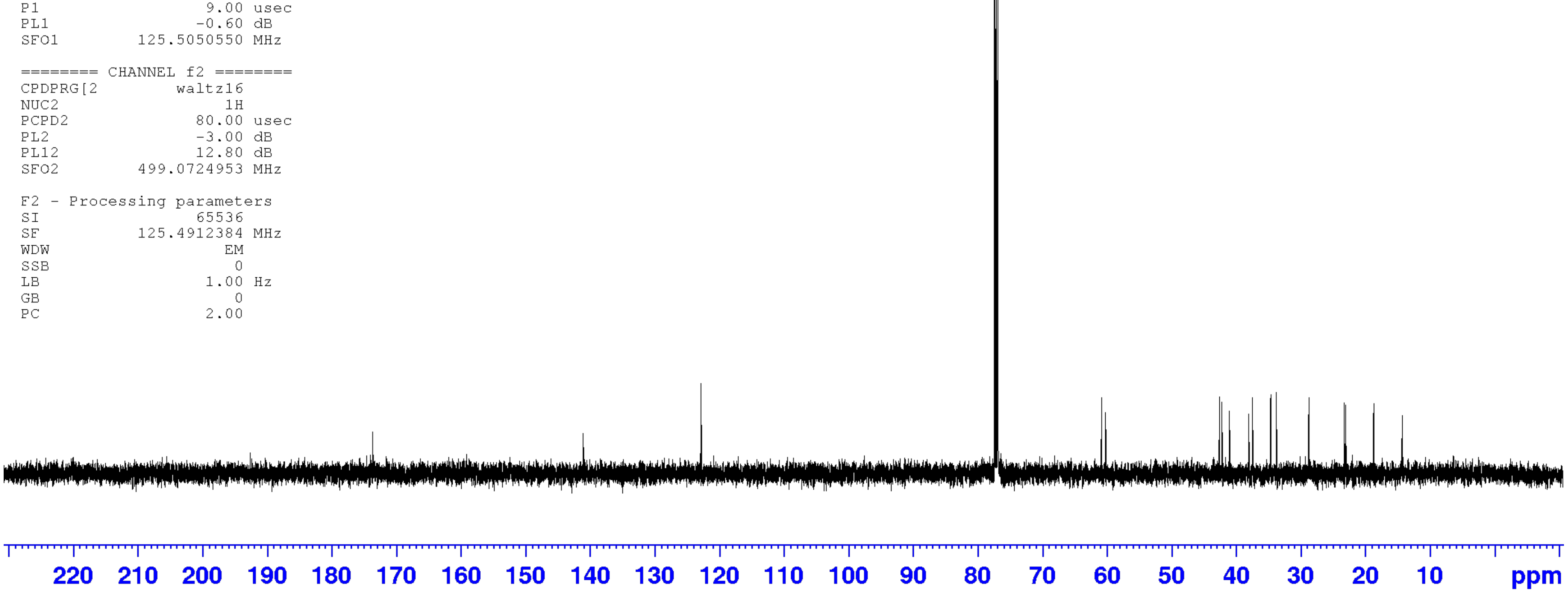

```
Current Data Parameters
NAME      JC 2-144 DMP col
EXPNO                    1
PROCNO                   1

F2 - Acquisition Parameters
Date_             20161222
Time                 21.11
INSTRUM            cryo500
PROBHD    5 mm CPTCI 1H-
PULPROG               zg30
TD                   81728
SOLVENT              CDCl3
NS                       8
DS                       2
SWH               8012.820 Hz
FIDRES            0.098043 Hz
AQ               5.0998273 sec
RG                     5.7
DW                  62.400 usec
DE                    6.00 usec
TE                   298.0 K
D1              0.10000000 sec
MCREST                   0 sec
MCWRK           0.01500000 sec

======== CHANNEL f1 ========
NUC1                    1H
P1                    7.50 usec
PL1                   1.60 dB
SFO1           500.2235015 MHz

F2 - Processing parameters
SI                   65536
SF             500.2200320 MHz
WDW                     EM
SSB                      0
LB                    0.30 Hz
GB                       0
PC                    1.00
```

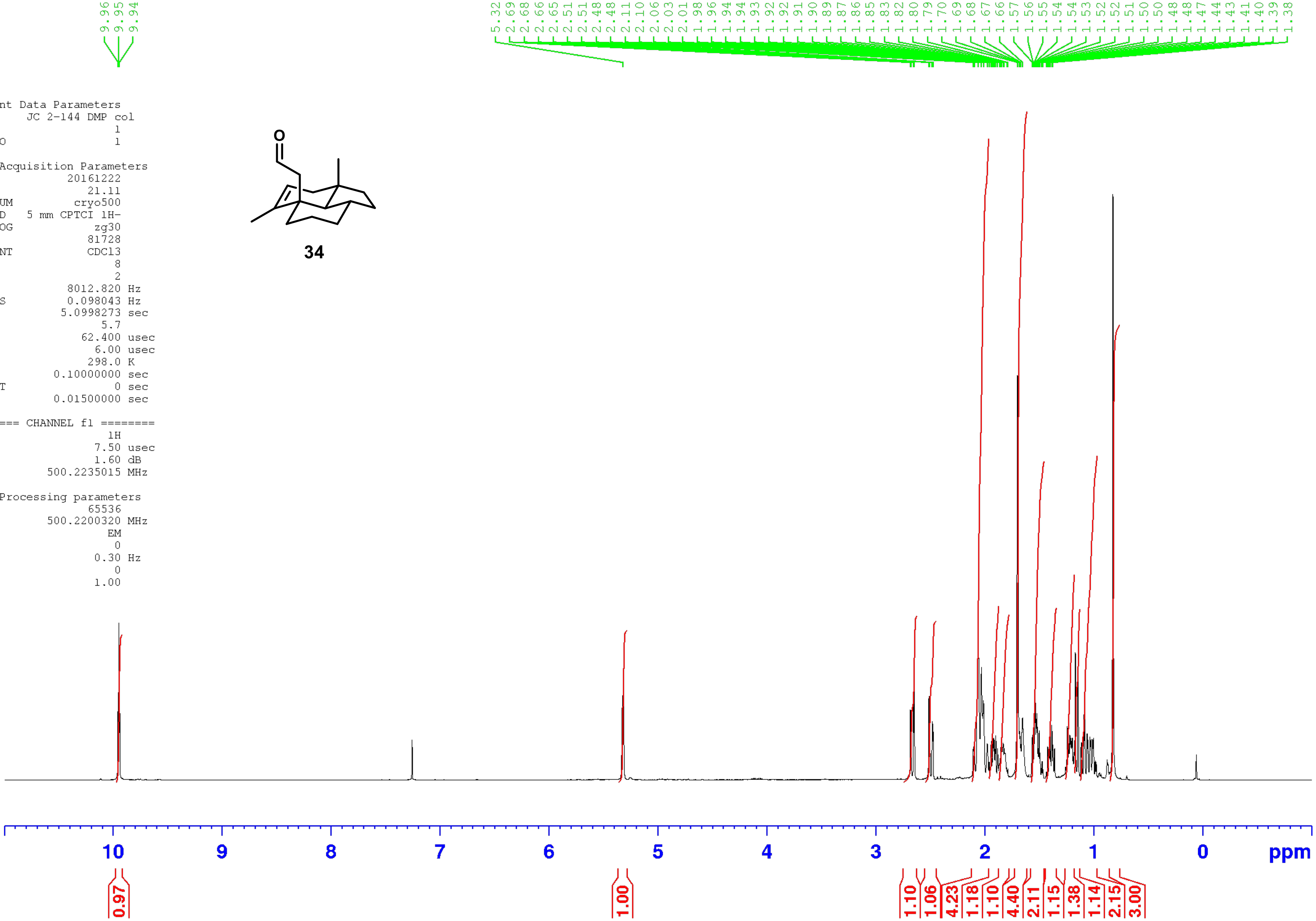

```
Current Data Parameters
NAME      JC 2-144 DMP col
EXPNO                    2
PROCNO                   1

F2 - Acquisition Parameters
Date_          20161222
Time              21.13
INSTRUM         cryo500
PROBHD   5 mm CPTCI 1H-
PULPROG  SpinEchopg30gp.prd
TD                65536
SOLVENT           CDCl3
NS                   32
DS                   16
SWH           30303.031 Hz
FIDRES         0.462388 Hz
AQ            1.0813440 sec
RG               7298.2
DW               16.500 usec
DE                 6.00 usec
TE                298.0 K
D1           1.00000000 sec
d11          0.03000000 sec
D16          0.00020000 sec
d17          0.00019600 sec
MCREST                0 sec
MCWRK        0.01500000 sec
P2                33.10 usec

======== CHANNEL f1 ========
NUC1                13C
P1                16.55 usec
P11              500.00 usec
P12             2000.00 usec
PL0              120.00 dB
PL1               -1.00 dB
SFO1        125.7942548 MHz
SP1                2.70 dB
SP2                2.70 dB
SPNAM[1] Crp60,0.5,20.1
SPNAM[2]    Crp60comp.4
SPOFF1                0 Hz
SPOFF2                0 Hz

======== CHANNEL f2 ========
CPDPRG[2        waltz16
NUC2                 1H
PCPD2            100.00 usec
PL2                1.60 dB
PL12              24.50 dB
SFO2        500.2225011 MHz

====== GRADIENT CHANNEL =====
GPNAM[1]       SINE.100
GPNAM[2]       SINE.100
GPX1                  0 %
GPX2                  0 %
GPY1                  0 %
GPY2                  0 %
GPZ1              30.00 %
GPZ2              50.00 %
p15              500.00 usec
p16             1000.00 usec

F2 - Processing parameters
SI                65536
SF          125.7804101 MHz
WDW                  EM
SSB                   0
LB                 1.00 Hz
GB                    0
PC                 2.00
```

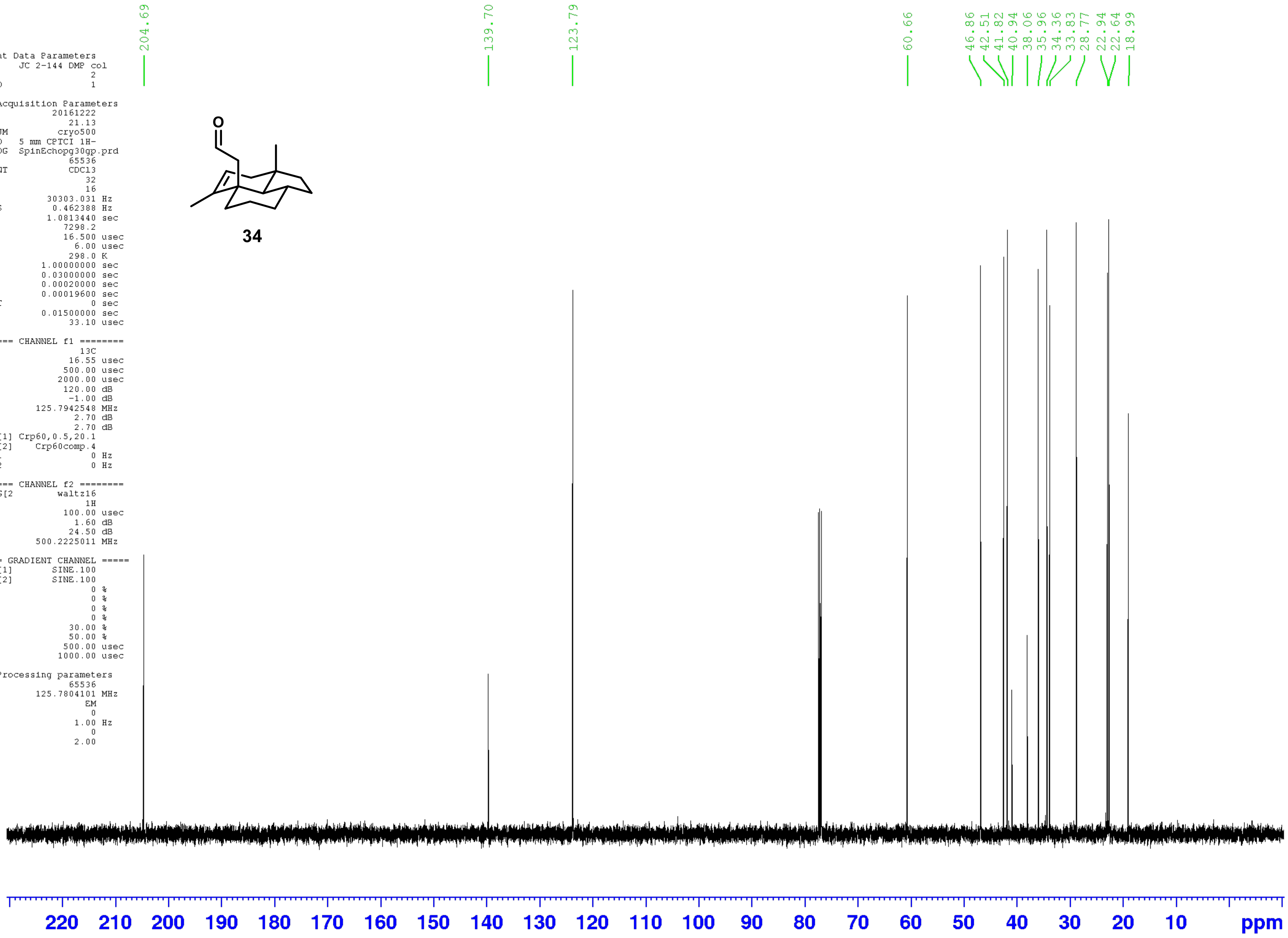

Current Data Parameters
NAME JC 2-148 HCl col
EXPNO 1
PROCNO 1

F2 - Acquisition Parameters
Date_ 20161224
Time 9.00
INSTRUM cryo500
PROBHD 5 mm CPTCI 1H-
PULPROG zg30
TD 81728
SOLVENT CDCl3
NS 8
DS 2
SWH 8012.820 Hz
FIDRES 0.098043 Hz
AQ 5.0998273 sec
RG 7.1
DW 62.400 usec
DE 6.00 usec
TE 298.0 K
D1 0.10000000 sec
MCREST 0 sec
MCWRK 0.01500000 sec

======== CHANNEL f1 ========
NUC1 1H
P1 7.50 usec
PL1 1.60 dB
SFO1 500.2235015 MHz

F2 - Processing parameters
SI 65536
SF 500.2200315 MHz
WDW EM
SSB 0
LB 0.30 Hz
GB 0
PC 1.00

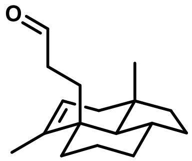

35

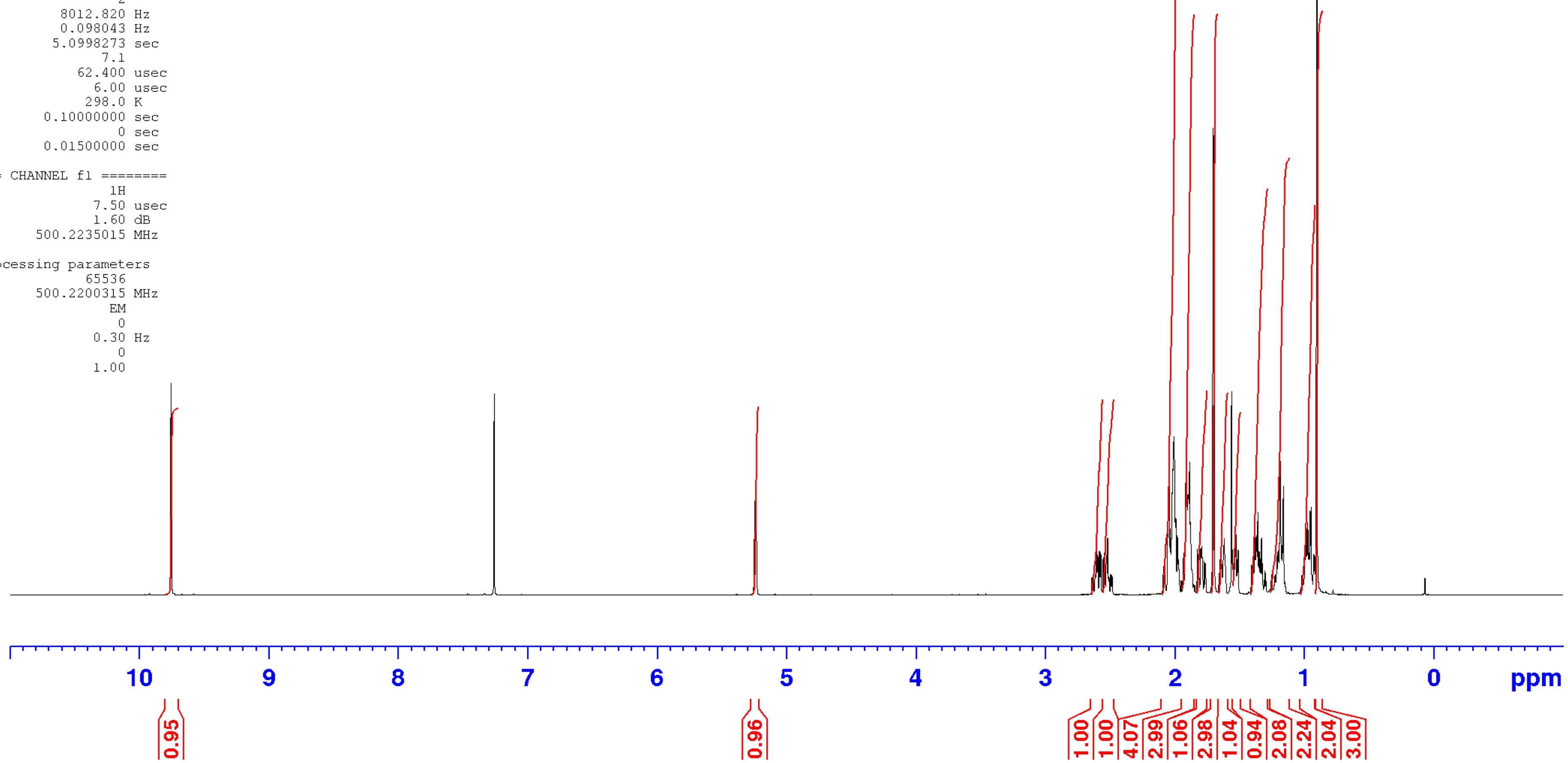

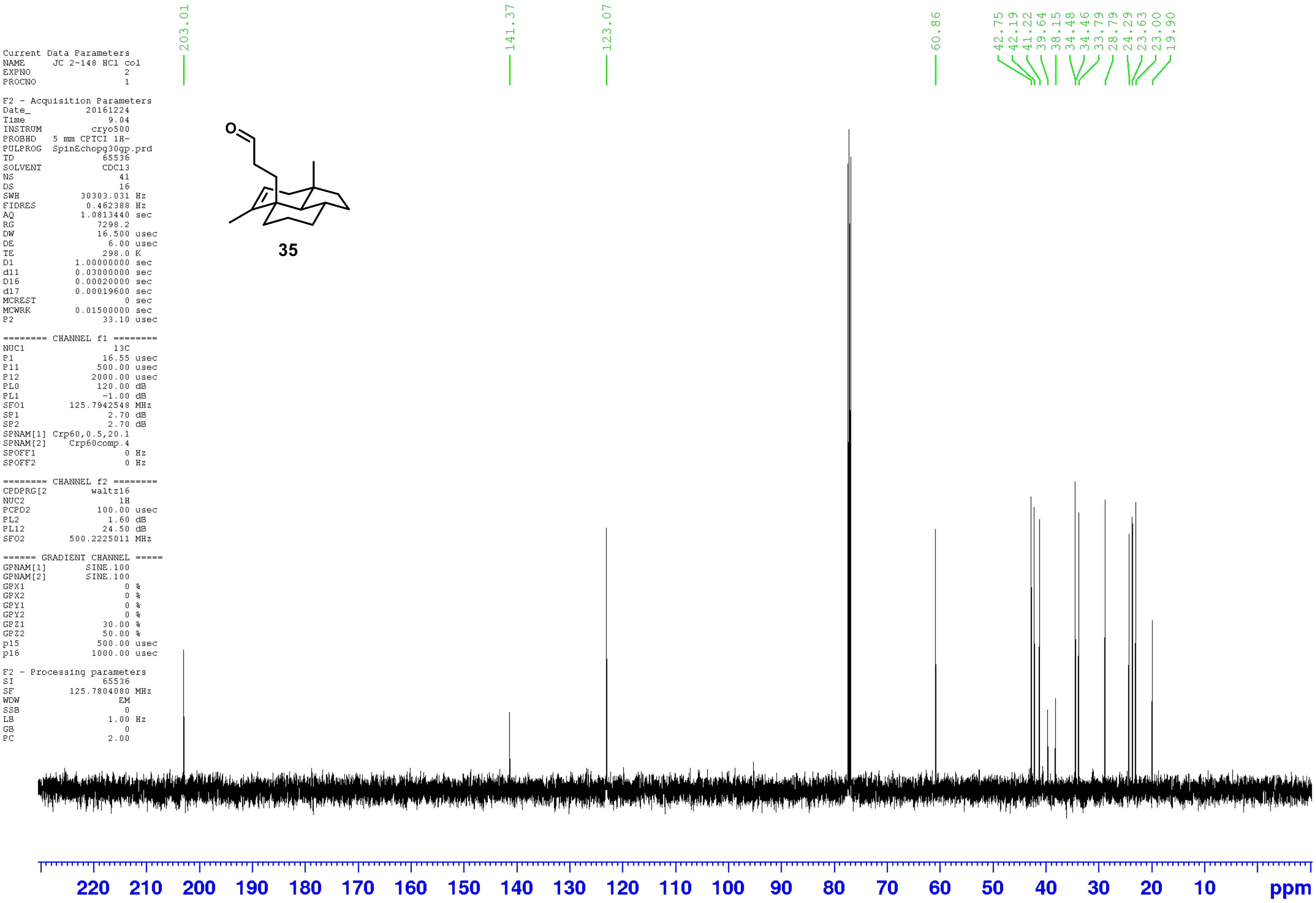
35
Current Data Parameters
NAME JC 2-148 HCl col
EXPNO 2
PROCNO 1
F2 - Acquisition Parameters
Date_ 20161224
Time 9.04
INSTRUM cryo500
PROBHD 5 mm CPTCI 1H-
PULPROG SpinEchopg30gp.prd
TD 65536
SOLVENT CDCl3
NS 41
DS 16
SWH 30303.031 Hz
FIDRES 0.462388 Hz
AQ 1.0813440 sec
RG 7298.2
DW 16.500 usec
DE 6.00 usec
TE 298.0 K
D1 1.00000000 sec
d11 0.03000000 sec
D16 0.00020000 sec
d17 0.00019600 sec
MCREST 0 sec
MCWRK 0.01500000 sec
P2 33.10 usec
======== CHANNEL f1 ========
NUC1 13C
P1 16.55 usec
P11 500.00 usec
P12 2000.00 usec
PL0 120.00 dB
PL1 -1.00 dB
SFO1 125.7942548 MHz
SP1 2.70 dB
SP2 2.70 dB
SPNAM[1] Crp60,0.5,20.1
SPNAM[2] Crp60comp.4
SPOFF1 0 Hz
SPOFF2 0 Hz
======== CHANNEL f2 ========
CPDPRG[2 waltz16
NUC2 1H
PCPD2 100.00 usec
PL2 1.60 dB
PL12 24.50 dB
SFO2 500.2225011 MHz
====== GRADIENT CHANNEL =====
GPNAM[1] SINE.100
GPNAM[2] SINE.100
GPX1 0 %
GPX2 0 %
GPY1 0 %
GPY2 0 %
GPZ1 30.00 %
GPZ2 50.00 %
p15 500.00 usec
p16 1000.00 usec
F2 - Processing parameters
SI 65536
SF 125.7804080 MHz
WDW EM
SSB 0
LB 1.00 Hz
GB 0
PC 2.00
203.01
141.37
123.07
60.86
42.75
42.19
41.22
39.64
38.15
34.48
34.46
33.79
28.79
24.29
23.63
23.00
19.90
220 210 200 190 180 170 160 150 140 130 120 110 100 90 80 70 60 50 40 30 20 10 ppm

```
Current Data Parameters
NAME      MG 1-128 Fr 16-15
EXPNO                   1
PROCNO                  1

F2 - Acquisition Parameters
Date_            20171031
Time                19.41
INSTRUM             av600
PROBHD    5 mm CPBBO BB-
PULPROG              zg30
TD                  38460
SOLVENT             CDCl3
NS                      8
DS                      0
SWH              9615.385 Hz
FIDRES           0.250010 Hz
AQ              1.9999200 sec
RG                   25.4
DW                 52.000 usec
DE                  13.70 usec
TE                  298.2 K
D1             0.10000000 sec
TD0                     1

======== CHANNEL f1 ========
SFO1         600.1342009 MHz
NUC1                  1H
P1                  12.00 usec
PLW1          20.00000000 W

F2 - Processing parameters
SI                  65536
SF            600.1300000 MHz
WDW                    EM
SSB                     0
LB                   0.30 Hz
GB                      0
PC                   1.00
```

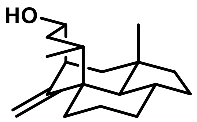

13, tentative structure assigned to minor component

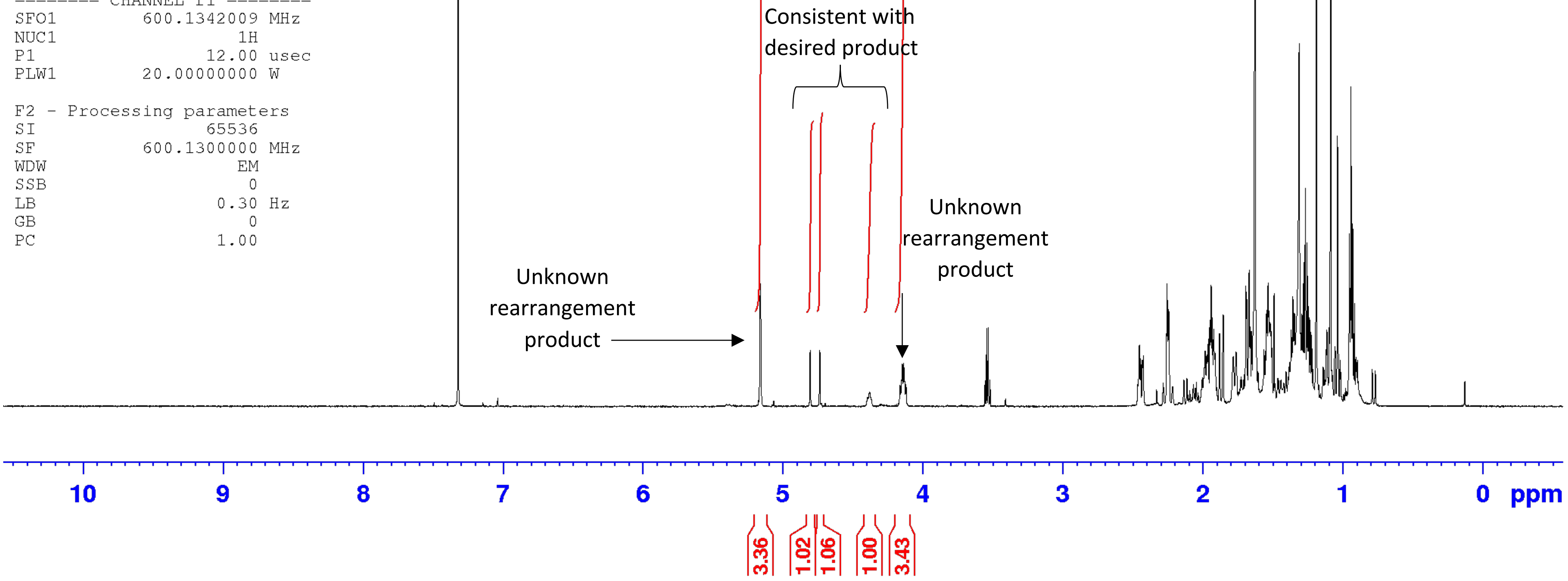

Current Data Parameters
NAME MG 1-103 Fr 9-19 AlMe3
EXPNO 1
PROCNO 1

F2 - Acquisition Parameters
Date_ 20170822
Time 16.45
INSTRUM cryo500
PROBHD 5 mm CPTCI 1H-
PULPROG zg30
TD 32048
SOLVENT CDCl3
NS 8
DS 0
SWH 8012.820 Hz
FIDRES 0.250026 Hz
AQ 1.9997952 sec
RG 2.5
DW 62.400 usec
DE 6.00 usec
TE 298.0 K
D1 0.10000000 sec
MCREST 0 sec
MCWRK 0.01500000 sec

======== CHANNEL f1 ========
NUC1 1H
P1 7.50 usec
PL1 1.60 dB
SFO1 500.2235015 MHz

F2 - Processing parameters
SI 65536
SF 500.2200307 MHz
WDW EM
SSB 0
LB 0.30 Hz
GB 0
PC 1.00

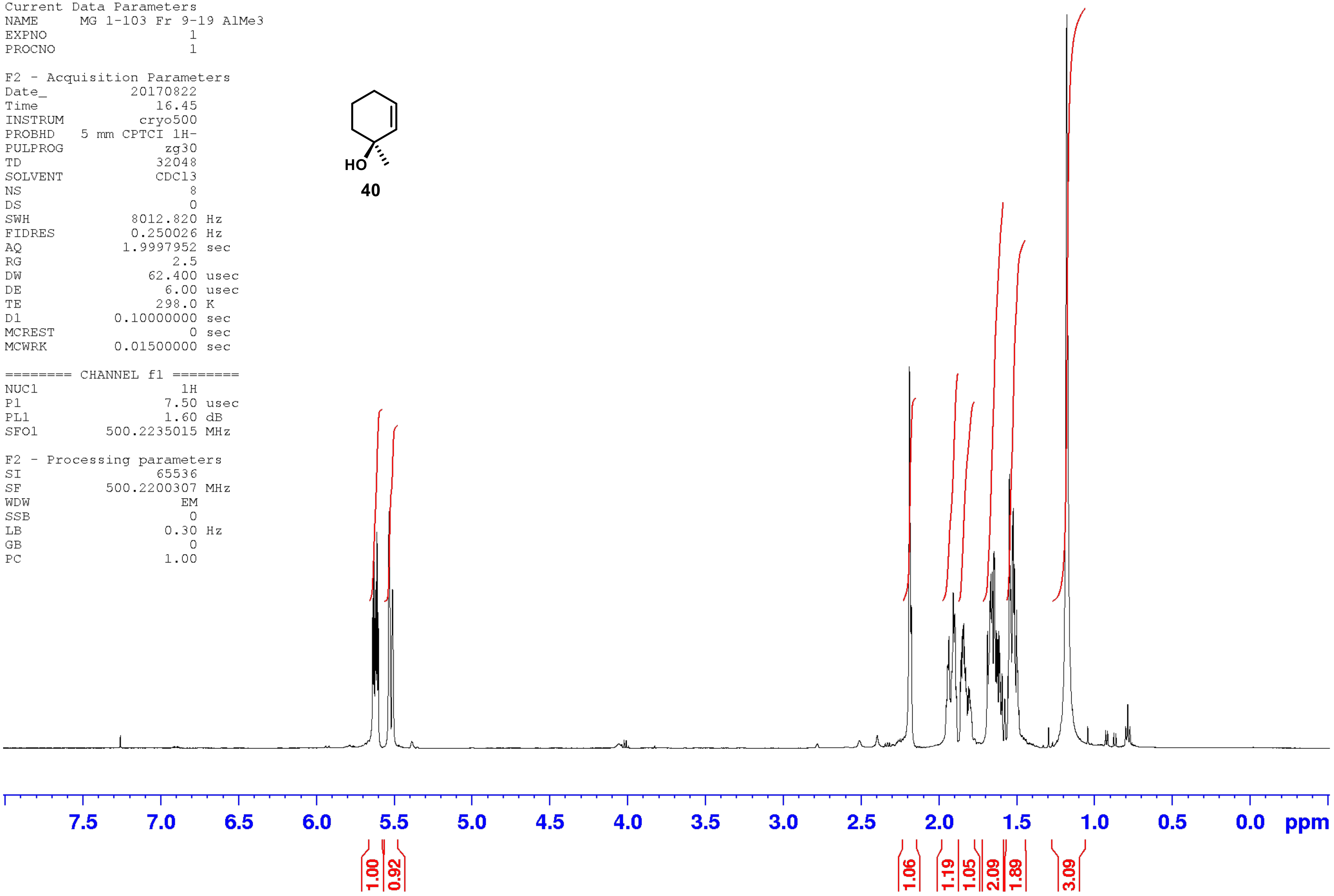

```
Current Data Parameters
NAME      MG 1-103 Fr 9-19 AlMe3
EXPNO                    2
PROCNO                   1

F2 - Acquisition Parameters
Date_            20170822
Time                16.47
INSTRUM           cryo500
PROBHD    5 mm CPTCI 1H-
PULPROG         deptqgpsp
TD                  65536
SOLVENT              CD3OD
NS                     59
DS                      0
SWH            30303.031 Hz
FIDRES          0.462388 Hz
AQ             1.0813440 sec
RG                   6502
DW                 16.500 usec
DE                   6.00 usec
TE                  298.0 K
CNST2         145.0000000
CNST12          1.5000000
D1             1.00000000 sec
d2             0.00344828 sec
d12            0.00002000 sec
D16            0.00020000 sec
DELTA          0.00001707 sec
DELTA1         0.00228023 sec
DELTA2         0.00226483 sec
DELTA3         0.00224828 sec
MCREST                  0 sec
MCWRK          0.01500000 sec

======== CHANNEL f1 ========
NUC1                  13C
P1                  16.55 usec
P12               2000.00 usec
PL0                120.00 dB
PL1                 -1.00 dB
SFO1          125.7942548 MHz
SP2                  2.70 dB
SPNAM[2]      Crp60comp.4
SPOFF2                  0 Hz

======== CHANNEL f2 ========
CPDPRG[2           waltz16
NUC2                   1H
p0                  11.55 usec
P3                   7.70 usec
p4                  15.40 usec
PCPD2              100.00 usec
PL2                  1.60 dB
PL12                24.50 dB
SFO2          500.2225011 MHz

====== GRADIENT CHANNEL =====
GPNAM[1]         SINE.100
GPNAM[2]         SINE.100
GPNAM[3]         SINE.100
GPX1                    0 %
GPX2                    0 %
GPX3                    0 %
GPY1                    0 %
GPY2                    0 %
GPY3                    0 %
GPZ1                31.00 %
GPZ2                31.00 %
GPZ3                31.00 %
P16               1000.00 usec

F2 - Processing parameters
SI                  65536
SF            125.7804264 MHz
WDW                    EM
SSB                     0
LB                   1.00 Hz
GB                      0
PC                   2.00
```

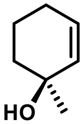

40

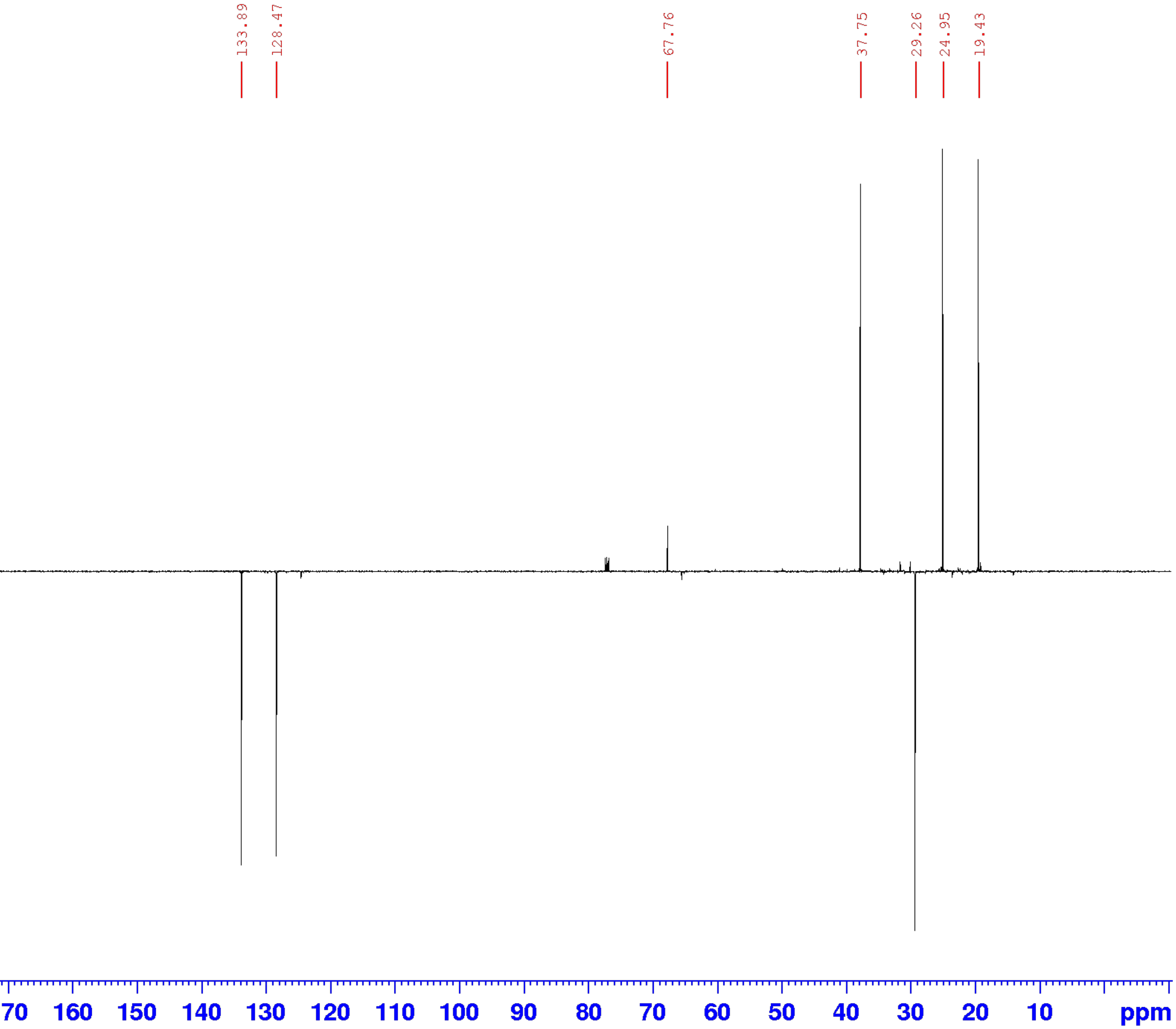

```
Current Data Parameters
NAME      MG 2-254 crude TBSCl
EXPNO                    1
PROCNO                   1

F2 - Acquisition Parameters
Date_             20180412
Time                 13.29
INSTRUM              av600
PROBHD    5 mm CPBBO BB-
PULPROG               zg30
TD                   38460
SOLVENT              CDCl3
NS                       8
DS                       0
SWH               9615.385 Hz
FIDRES            0.250010 Hz
AQ               1.9999200 sec
RG                      10
DW                  52.000 usec
DE                   14.07 usec
TE                   298.0 K
D1              0.10000000 sec
TD0                      1

======== CHANNEL f1 ========
SFO1          600.1342009 MHz
NUC1                    1H
P1                   10.25 usec
PLW1           20.00000000 W

F2 - Processing parameters
SI                   65536
SF            600.1300360 MHz
WDW                     EM
SSB                      0
LB                    0.30 Hz
GB                       0
PC                    1.00
```

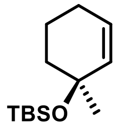

S12

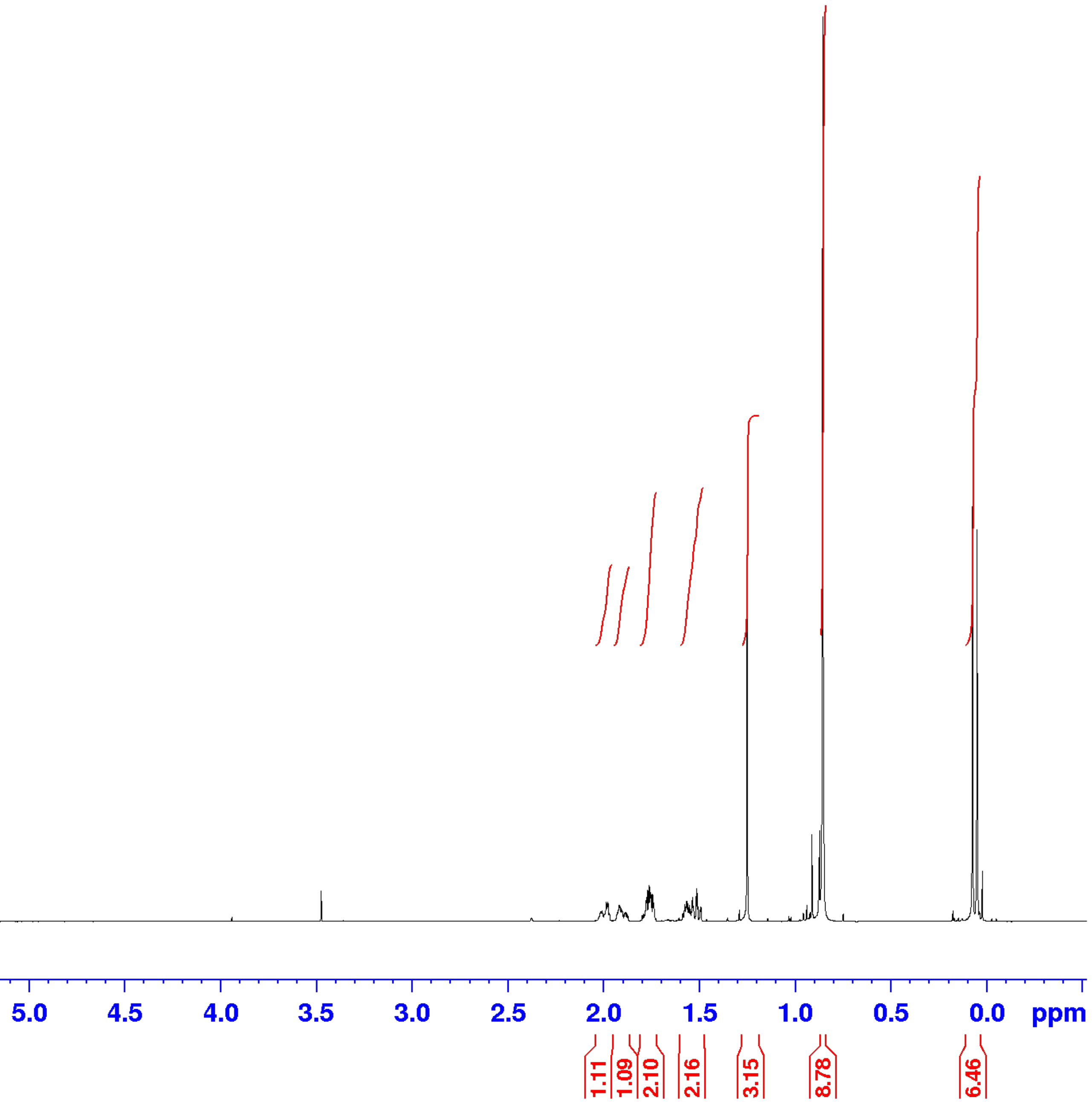

```
Current Data Parameters
NAME       MG 2-254 crude TBSCl
EXPNO                     2
PROCNO                    1

F2 - Acquisition Parameters
Date_              20180412
Time                  13.37
INSTRUM               av600
PROBHD    5 mm CPBBO BB-
PULPROG          deptqgpsp
TD                    65536
SOLVENT               CDCl3
NS                       72
DS                        0
SWH              36231.883 Hz
FIDRES            0.552855 Hz
AQ               0.9043968 sec
RG                     2050
DW                   13.800 usec
DE                    18.00 usec
TE                    298.0 K
CNST2           145.0000000
CNST12            1.5000000
D1               2.00000000 sec
D2               0.00344828 sec
D12              0.00002000 sec
D16              0.00020000 sec
TD0                       1

======== CHANNEL f1 ========
SFO1          150.9194080 MHz
NUC1                    13C
P1                    10.00 usec
P13                 2000.00 usec
PLW0                      0 W
PLW1            64.00000000 W
SPNAM[5]       Crp60comp.4
SPOAL5                0.500
SPOFFS5                   0 Hz
SPW5            11.50000000 W

======== CHANNEL f2 ========
SFO2          600.1330010 MHz
NUC2                     1H
CPDPRG[2             waltz16
P0                    15.38 usec
P3                    10.25 usec
P4                    20.50 usec
PCPD2                 80.00 usec
PLW2            20.00000000 W
PLW12            0.33113000 W

====== GRADIENT CHANNEL =====
GPNAM[1]        SMSQ10.100
GPNAM[2]        SMSQ10.100
GPNAM[3]        SMSQ10.100
GPZ1                  31.00 %
GPZ2                  31.00 %
GPZ3                  31.00 %
P16                 1000.00 usec

F2 - Processing parameters
SI                    65536
SF           150.9028085 MHz
WDW                      EM
SSB                       0
LB                     1.00 Hz
GB                        0
PC                     1.00
```

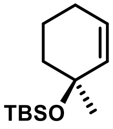

S12

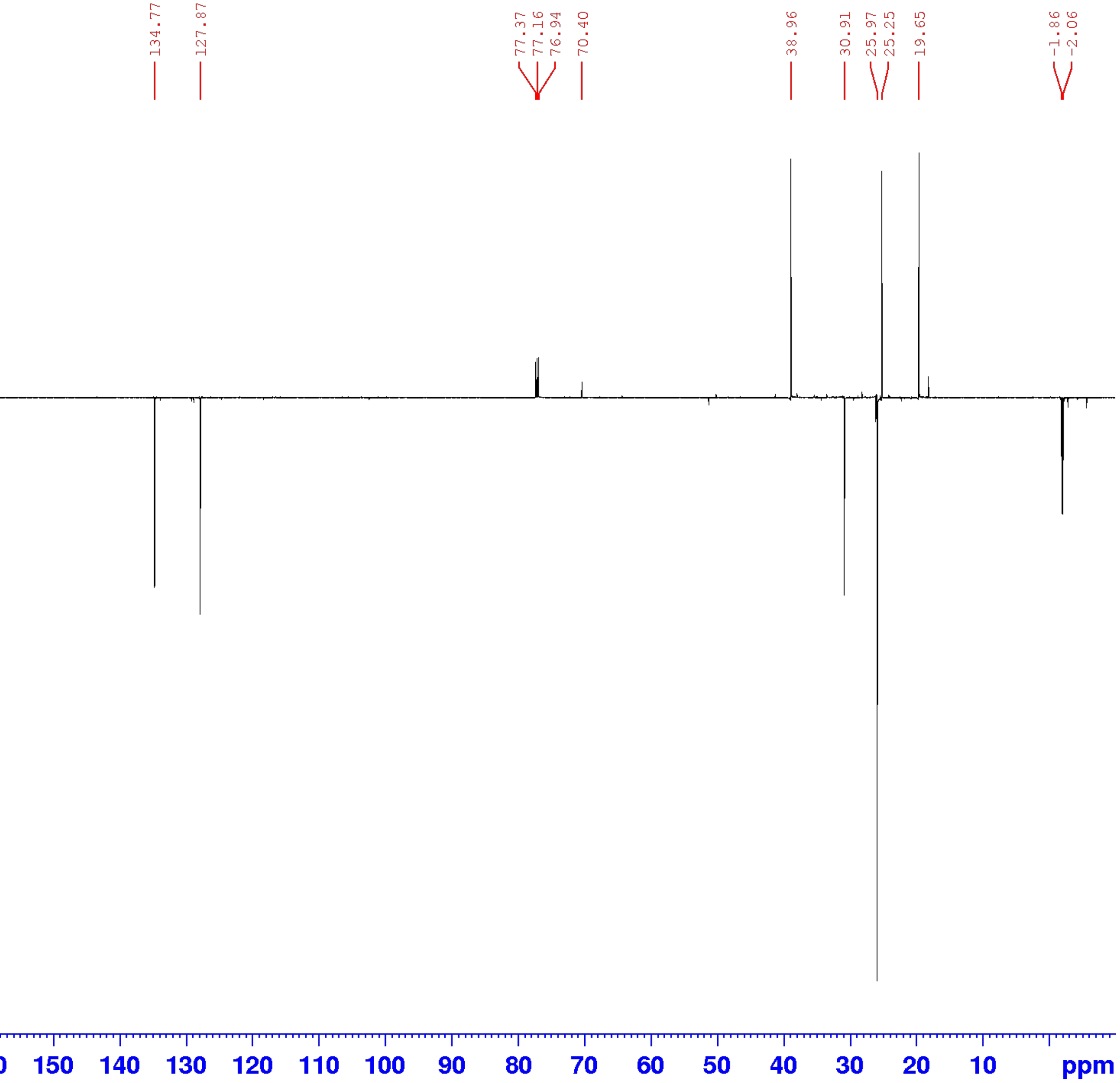

```
Current Data Parameters
NAME      JC 3-188 Rh col3 PUB
EXPNO                    1
PROCNO                   1

F2 - Acquisition Parameters
Date_            20201010
Time                 2.37
INSTRUM             av600
PROBHD   5 mm CPBBO BB-
PULPROG              zg30
TD                  98074
SOLVENT             CDCl3
NS                      8
DS                      2
SWH              9615.385 Hz
FIDRES           0.098042 Hz
AQ              5.0998478 sec
RG                     10
DW                 52.000 usec
DE                  13.70 usec
TE                  298.0 K
D1             0.10000000 sec
TD0                     1

======== CHANNEL f1 ========
SFO1          600.1342009 MHz
NUC1                   1H
P1                  12.00 usec
PLW1          28.00000000 W

F2 - Processing parameters
SI                  65536
SF            600.1300349 MHz
WDW                    EM
SSB       0
LB                   0.30 Hz
GB        0
PC                   1.00
```

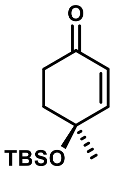

41

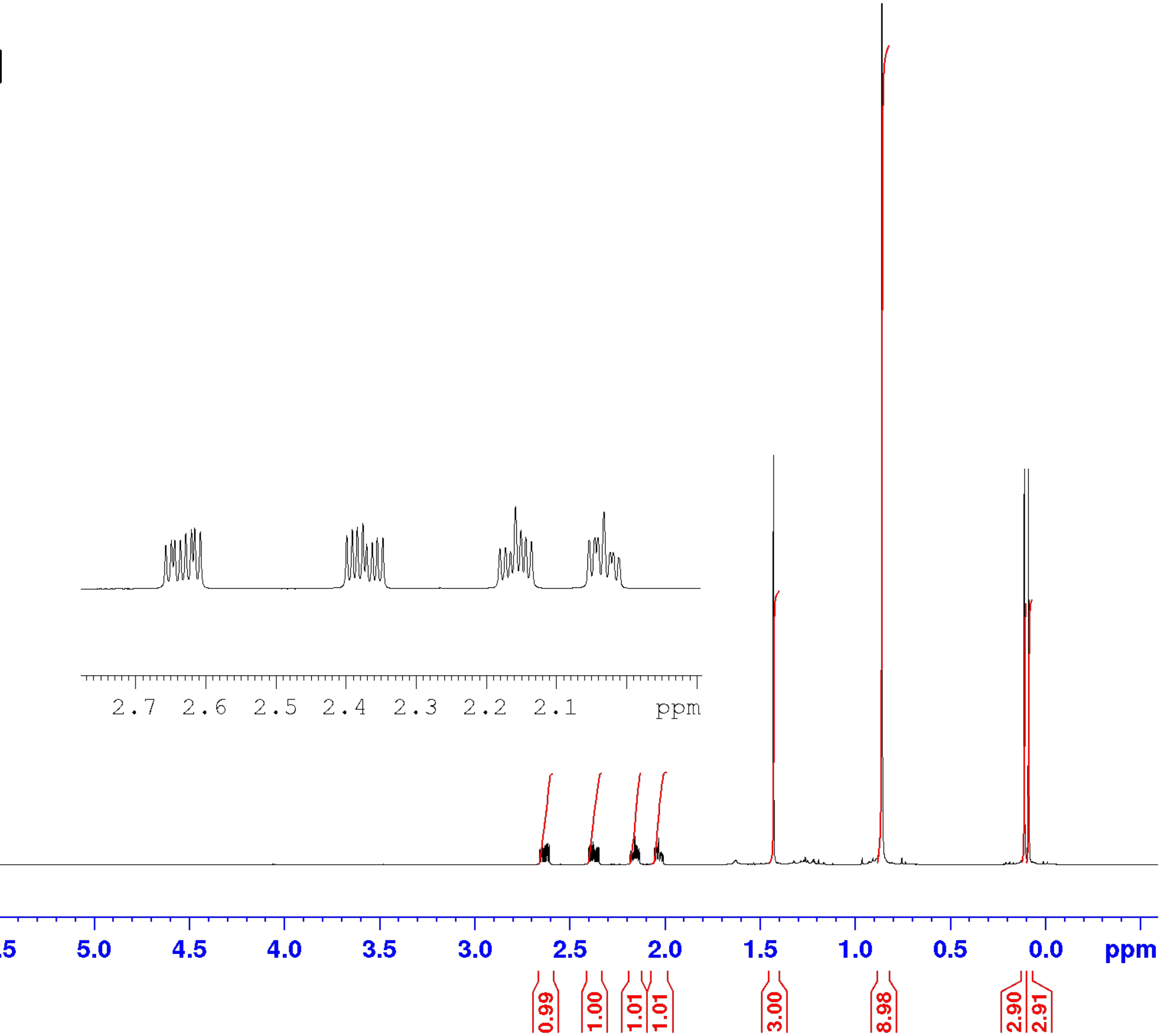

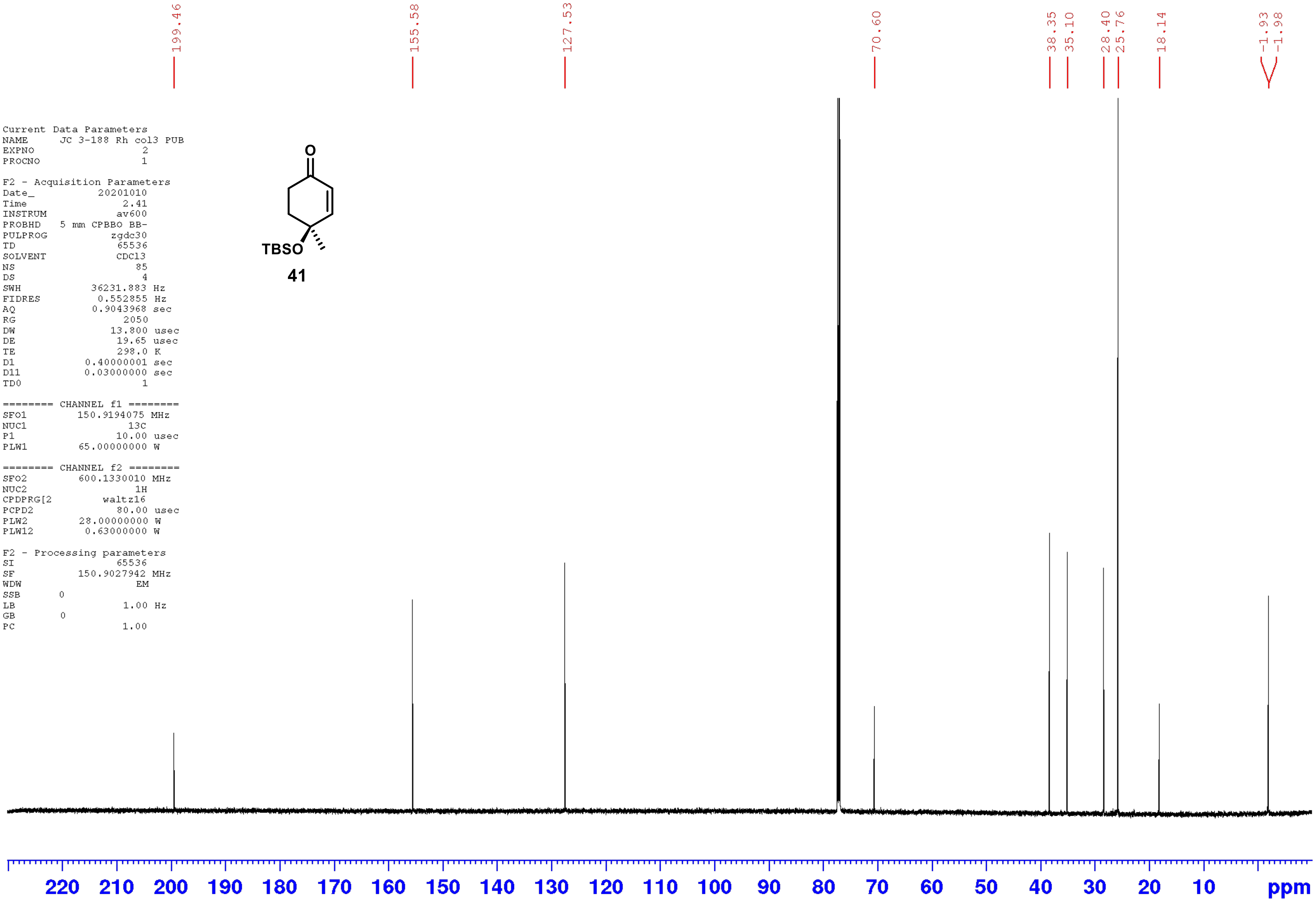


```
Current Data Parameters
NAME      JC 3-188 Rh col3 PUB
EXPNO                2
PROCNO               1

F2 - Acquisition Parameters
Date_           20201010
Time                2.41
INSTRUM            av600
PROBHD   5 mm CPBBO BB-
PULPROG          zgdc30
TD                65536
SOLVENT           CDCl3
NS                   85
DS                    4
SWH           36231.883 Hz
FIDRES         0.552855 Hz
AQ            0.9043968 sec
RG                 2050
DW               13.800 usec
DE                19.65 usec
TE                298.0 K
D1           0.40000001 sec
D11          0.03000000 sec
TD0                   1

======== CHANNEL f1 ========
SFO1        150.9194075 MHz
NUC1                13C
P1                10.00 usec
PLW1        65.00000000 W

======== CHANNEL f2 ========
SFO2        600.1330010 MHz
NUC2                 1H
CPDPRG[2         waltz16
PCPD2             80.00 usec
PLW2        28.00000000 W
PLW12        0.63000000 W

F2 - Processing parameters
SI                65536
SF          150.9027942 MHz
WDW                  EM
SSB       0
LB                 1.00 Hz
GB        0
PC                 1.00
```


41

```
Current Data Parameters
NAME      JC 3-225 top col
EXPNO                    1
PROCNO                   1

F2 - Acquisition Parameters
Date_          20201023
Time               4.05
INSTRUM           av600
PROBHD   5 mm CPBBO BB-
PULPROG            zg30
TD                98074
SOLVENT           CDCl3
NS                    8
DS                    2
SWH           9615.385 Hz
FIDRES        0.098042 Hz
AQ           5.0998478 sec
RG                    8
DW               52.000 usec
DE                13.70 usec
TE                298.0 K
D1           0.10000000 sec
TD0                   1

======== CHANNEL f1 ========
SFO1        600.1342009 MHz
NUC1                  1H
P1                12.00 usec
PLW1        28.00000000 W

F2 - Processing parameters
SI                65536
SF          600.1300345 MHz
WDW                  EM
SSB      0
LB                 0.30 Hz
GB       0
PC                 1.00
```

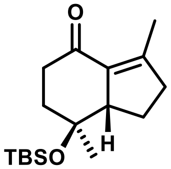

43

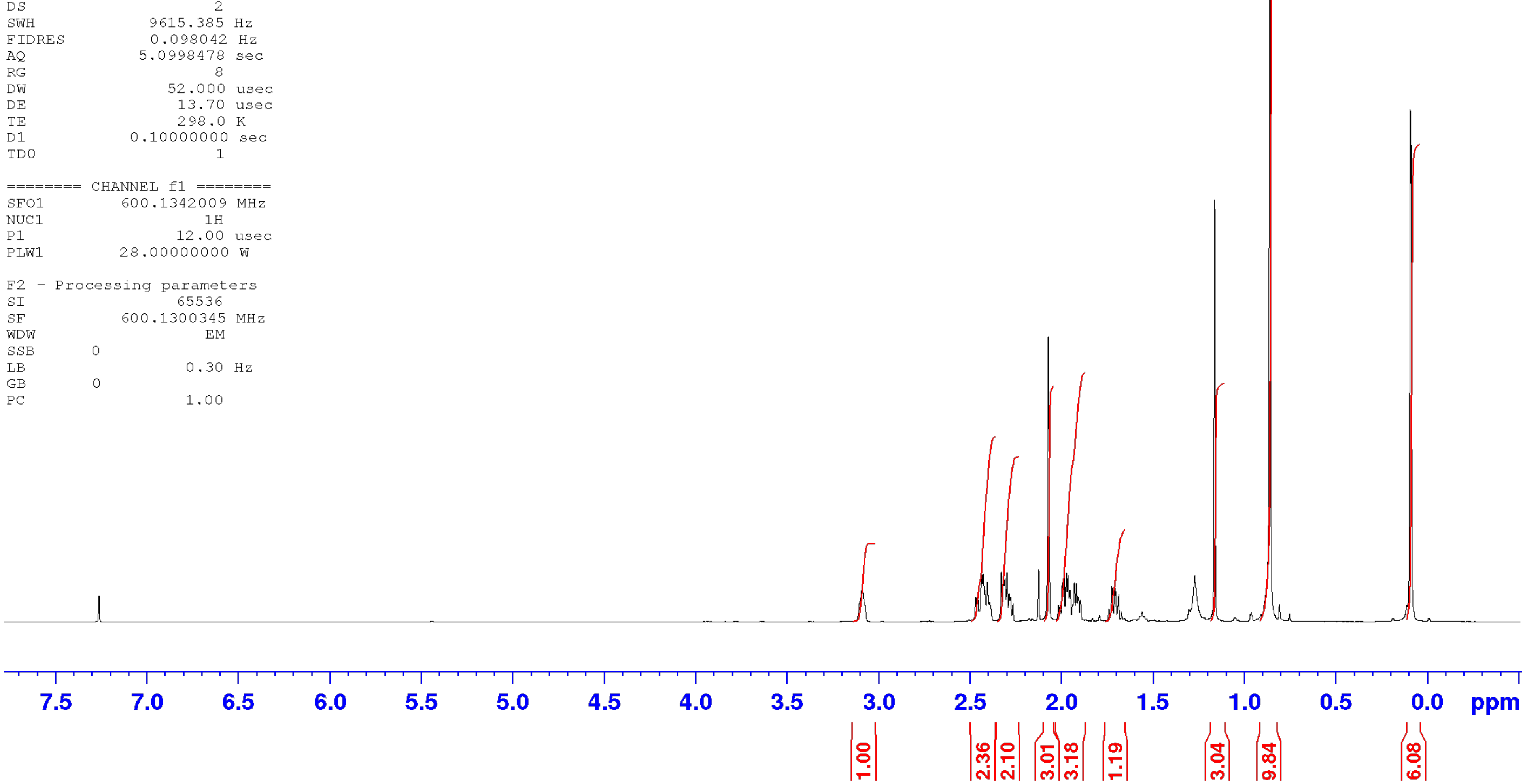

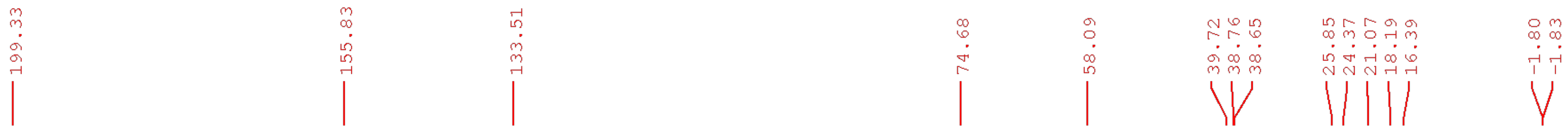


```
Current Data Parameters
NAME      JC 3-225 top col
EXPNO                    2
PROCNO                   1

F2 - Acquisition Parameters
Date_            20201023
Time                 4.09
INSTRUM             av600
PROBHD   5 mm CPBBO BB-
PULPROG           zgdc30
TD                 65536
SOLVENT            CDCl3
NS                   127
DS                     4
SWH            36231.883 Hz
FIDRES          0.552855 Hz
AQ             0.9043968 sec
RG                  2050
DW                13.800 usec
DE                 19.65 usec
TE                 298.0 K
D1            0.40000001 sec
D11           0.03000000 sec
TD0                    1

======== CHANNEL f1 ========
SFO1         150.9194075 MHz
NUC1                 13C
P1                 10.00 usec
PLW1         65.00000000 W

======== CHANNEL f2 ========
SFO2         600.1330010 MHz
NUC2                  1H
CPDPRG[2          waltz16
PCPD2              80.00 usec
PLW2         28.00000000 W
PLW12         0.63000000 W

F2 - Processing parameters
SI                 65536
SF           150.9027946 MHz
WDW                   EM
SSB      0
LB                  1.00 Hz
GB       0
PC                  1.00
```

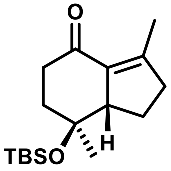

43

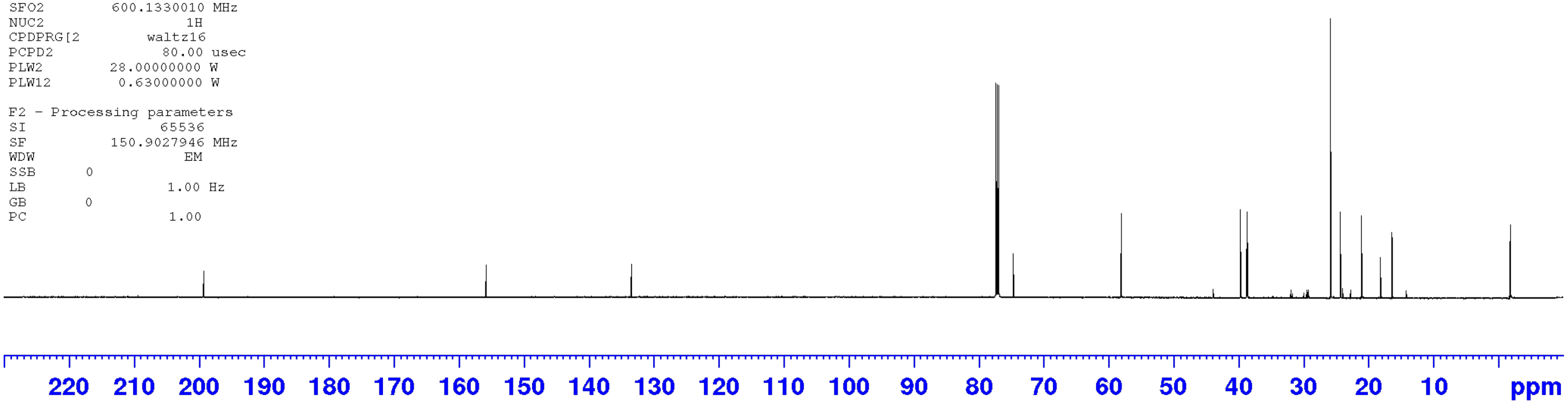

```
Current Data Parameters
NAME      JC 3-234 top spot
EXPNO                    1
PROCNO                   1

F2 - Acquisition Parameters
Date_             20201113
Time                 22.36
INSTRUM            cryo500
PROBHD    5 mm CPTCI 1H-
PULPROG               zg30
TD                   16022
SOLVENT              CDCl3
NS                       8
DS                       2
SWH               8012.820 Hz
FIDRES            0.500114 Hz
AQ               0.9997728 sec
RG                     3.2
DW                  62.400 usec
DE                    6.00 usec
TE                   298.0 K
D1              0.10000000 sec
MCREST    0 sec
MCWRK           0.01500000 sec

======== CHANNEL f1 ========
NUC1                    1H
P1                    7.50 usec
PL1                   1.60 dB
SFO1           500.2235015 MHz

F2 - Processing parameters
SI                   65536
SF             500.2200312 MHz
WDW                     EM
SSB       0
LB                    0.30 Hz
GB        0
PC                    1.00
```

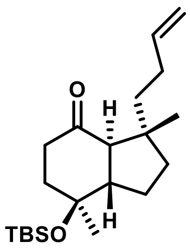

47

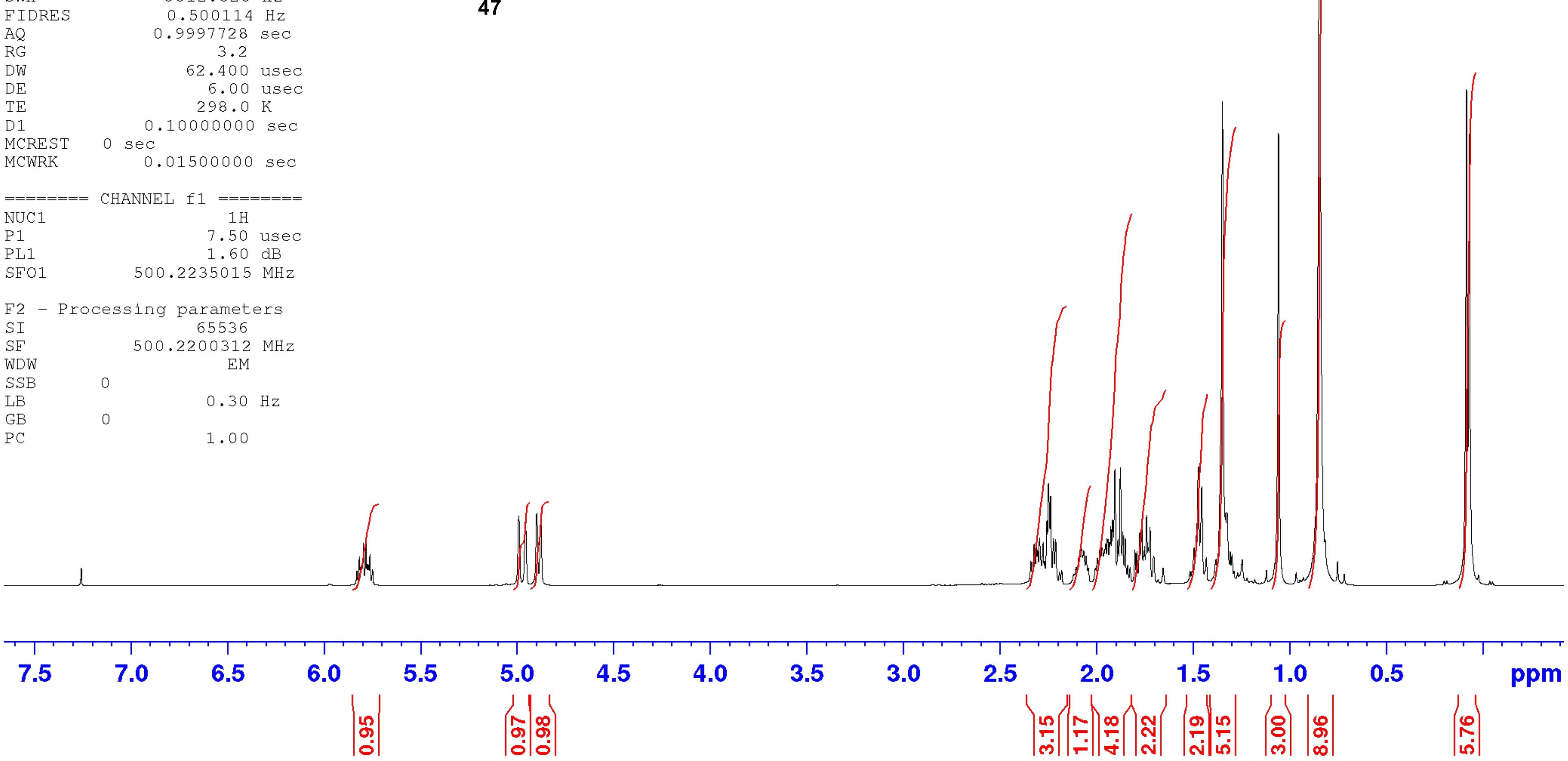

```
Current Data Parameters
NAME      JC 3-234 top spot
EXPNO                  2
PROCNO                 1

F2 - Acquisition Parameters
Date_           20201113
Time               22.39
INSTRUM          cryo500
PROBHD   5 mm CPTCI 1H-
PULPROG        deptqgpsp
TD                 65536
SOLVENT            CDCl3
NS                   125
DS                     8
SWH            30303.031 Hz
FIDRES          0.462388 Hz
AQ             1.0813440 sec
RG                  2048
DW                16.500 usec
DE                  6.00 usec
TE                 298.0 K
CNST2        145.0000000
CNST12         1.5000000
D1            1.00000000 sec
d2            0.00344828 sec
d12           0.00002000 sec
D16           0.00020000 sec
DELTA         0.00001707 sec
DELTA1        0.00228023 sec
DELTA2        0.00226483 sec
DELTA3        0.00224828 sec
MCREST    0 sec
MCWRK         0.01500000 sec

======== CHANNEL f1 ========
NUC1                 13C
P1                 16.55 usec
P12              2000.00 usec
PL0               120.00 dB
PL1                -1.00 dB
SFO1         125.7942548 MHz
SP2                 2.70 dB
SPNAM[2]    Crp60comp.4
SPOFF2    0 Hz

======== CHANNEL f2 ========
CPDPRG[2         waltz16
NUC2                  1H
p0                 11.55 usec
P3                  7.70 usec
p4                 15.40 usec
PCPD2             100.00 usec
PL2                 1.60 dB
PL12               24.50 dB
SFO2         500.2225011 MHz

====== GRADIENT CHANNEL =====
GPNAM[1]        SINE.100
GPNAM[2]        SINE.100
GPNAM[3]        SINE.100
GPX1      0 %
GPX2      0 %
GPX3      0 %
GPY1      0 %
GPY2      0 %
GPY3      0 %
GPZ1               31.00 %
GPZ2               31.00 %
GPZ3               31.00 %
P16              1000.00 usec

F2 - Processing parameters
SI                 65536
SF           125.7804078 MHz
WDW                   EM
SSB       0
LB                  1.00 Hz
GB        0
PC                  2.00
```

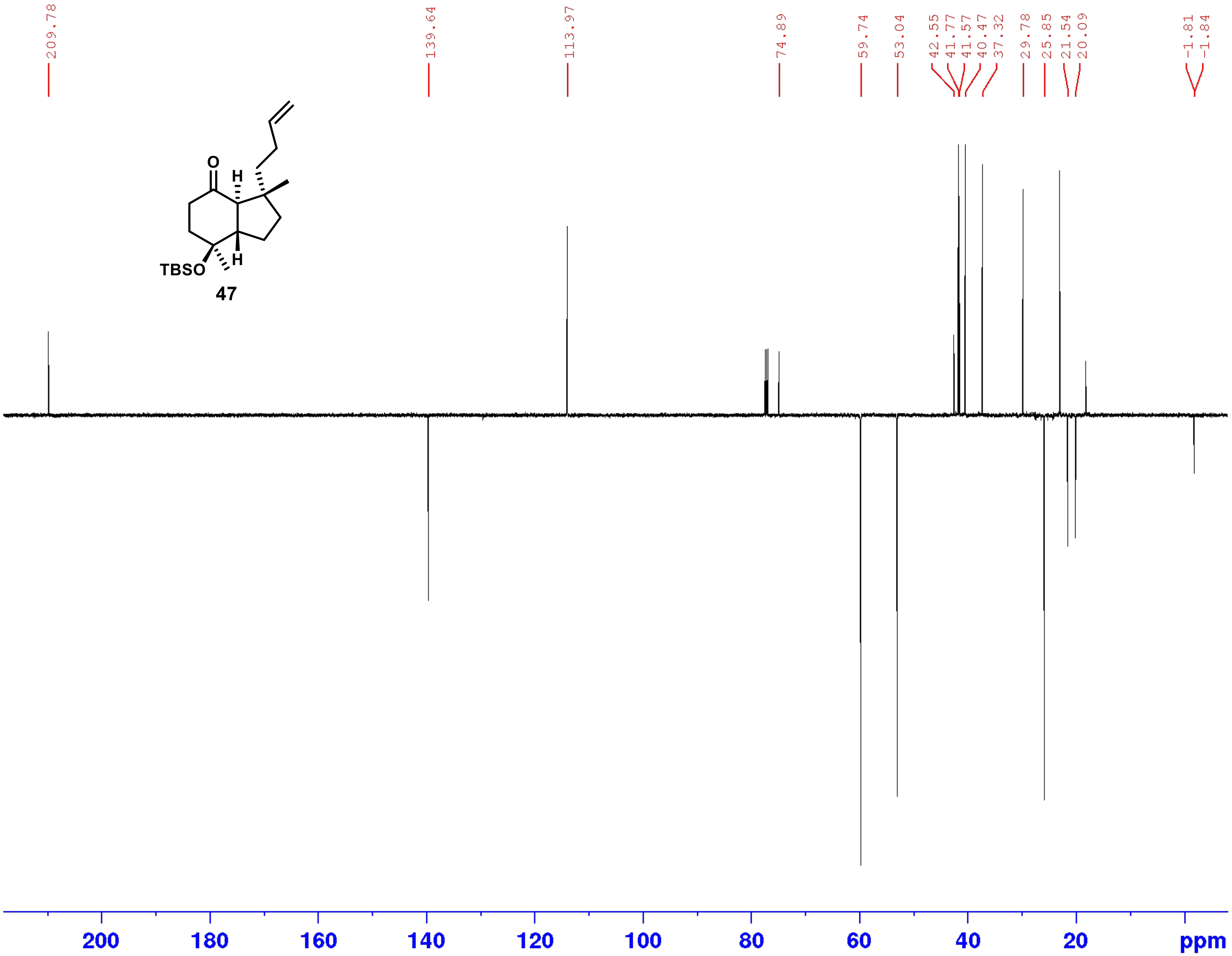

```
Current Data Parameters
NAME      JC 3-234 bot cis
EXPNO                    1
PROCNO                   1

F2 - Acquisition Parameters
Date_             20201113
Time                 22.44
INSTRUM            cryo500
PROBHD    5 mm CPTCI 1H-
PULPROG               zg30
TD                   16022
SOLVENT              CDCl3
NS                       8
DS                       2
SWH               8012.820 Hz
FIDRES            0.500114 Hz
AQ               0.9997728 sec
RG                       4
DW                  62.400 usec
DE                    6.00 usec
TE                   298.0 K
D1              0.10000000 sec
MCREST    0 sec
MCWRK           0.01500000 sec

======== CHANNEL f1 ========
NUC1                    1H
P1                    7.50 usec
PL1                   1.60 dB
SFO1           500.2235015 MHz

F2 - Processing parameters
SI                   65536
SF             500.2200312 MHz
WDW                     EM
SSB       0
LB                    0.30 Hz
GB        0
PC                    2.00
```

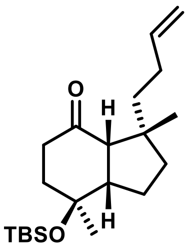

S14

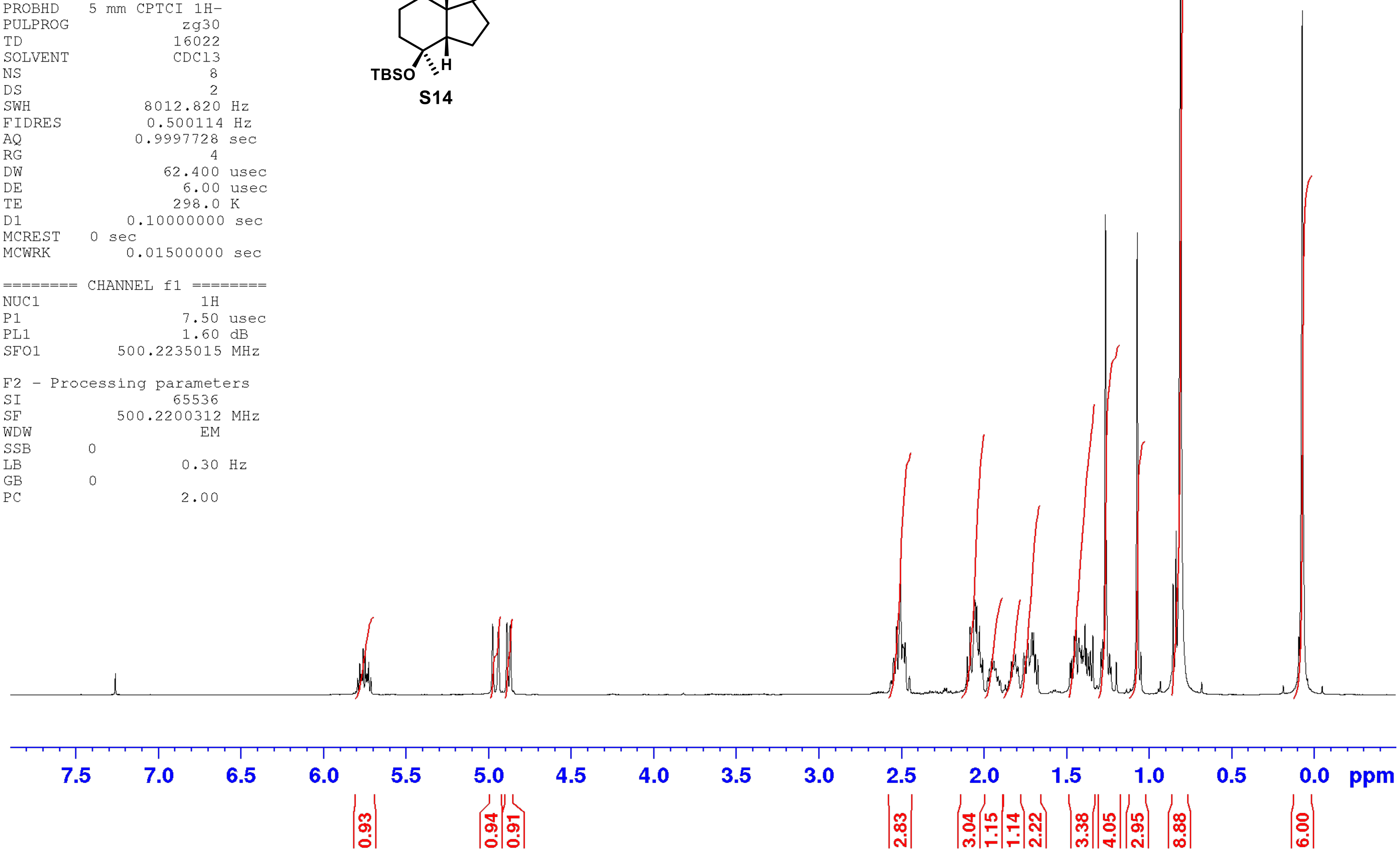

Current Data Parameters
NAME JC 3-234 bot cis
EXPNO 2
PROCNO 1

F2 - Acquisition Parameters
Date_ 20201113
Time 22.45
INSTRUM cryo500
PROBHD 5 mm CPTCI 1H-
PULPROG deptqgpsp
TD 65536
SOLVENT CDCl3
NS 24
DS 8
SWH 30303.031 Hz
FIDRES 0.462388 Hz
AQ 1.0813440 sec
RG 4096
DW 16.500 usec
DE 6.00 usec
TE 298.0 K
CNST2 145.0000000
CNST12 1.5000000
D1 1.00000000 sec
d2 0.00344828 sec
d12 0.00002000 sec
D16 0.00020000 sec
DELTA 0.00001707 sec
DELTA1 0.00228023 sec
DELTA2 0.00226483 sec
DELTA3 0.00224828 sec
MCREST 0 sec
MCWRK 0.01500000 sec

======== CHANNEL f1 ========
NUC1 13C
P1 16.55 usec
P12 2000.00 usec
PL0 120.00 dB
PL1 -1.00 dB
SFO1 125.7942548 MHz
SP2 2.70 dB
SPNAM[2] Crp60comp.4
SPOFF2 0 Hz

======== CHANNEL f2 ========
CPDPRG[2 waltz16
NUC2 1H
p0 11.55 usec
P3 7.70 usec
p4 15.40 usec
PCPD2 100.00 usec
PL2 1.60 dB
PL12 24.50 dB
SFO2 500.2225011 MHz

====== GRADIENT CHANNEL =====
GPNAM[1] SINE.100
GPNAM[2] SINE.100
GPNAM[3] SINE.100
GPX1 0 %
GPX2 0 %
GPX3 0 %
GPY1 0 %
GPY2 0 %
GPY3 0 %
GPZ1 31.00 %
GPZ2 31.00 %
GPZ3 31.00 %
P16 1000.00 usec

F2 - Processing parameters
SI 65536
SF 125.7804090 MHz
WDW EM
SSB 0
LB 1.00 Hz
GB 0
PC 2.00

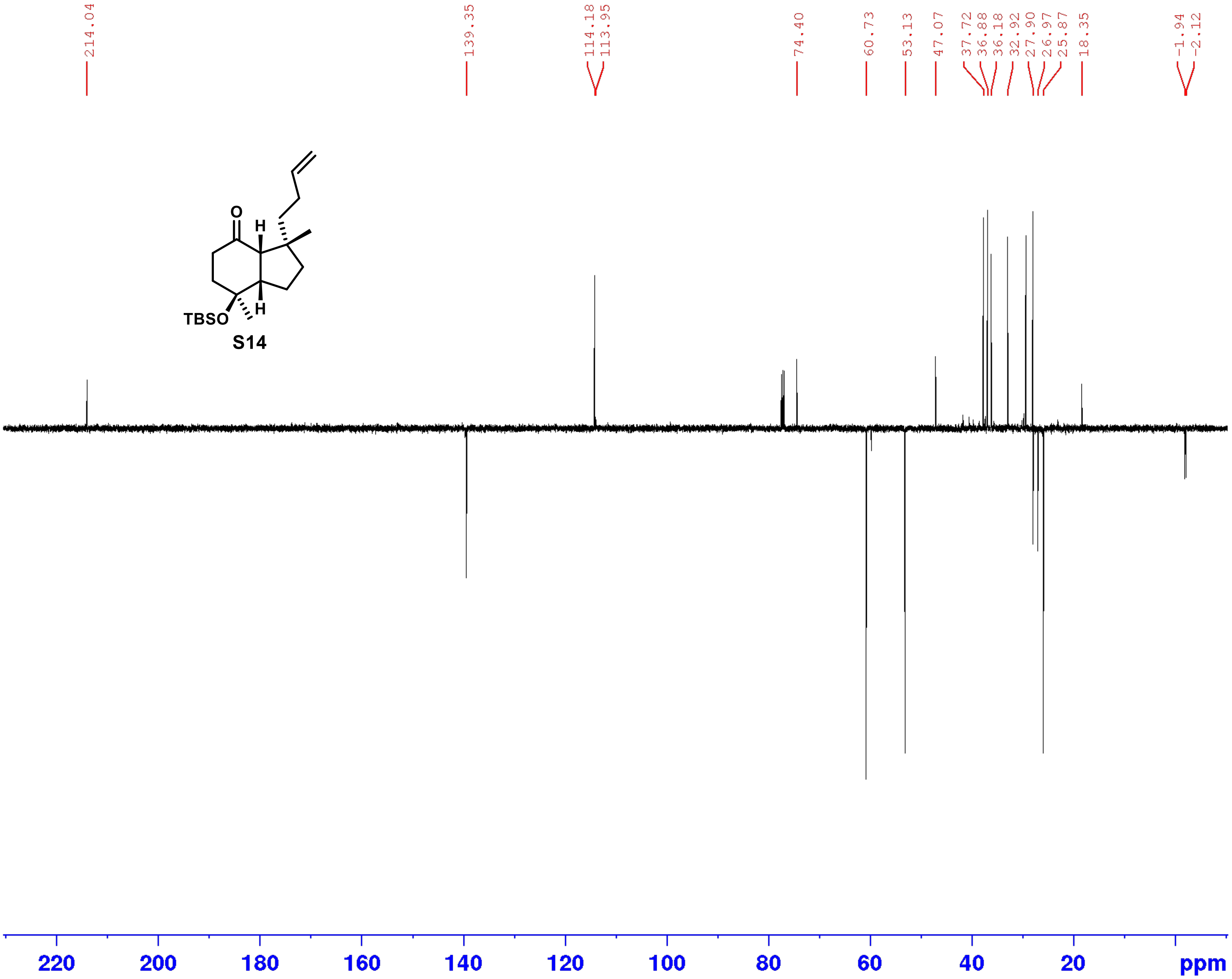

S14

```
Current Data Parameters
NAME      JC 3-242 FRAC 6 col
EXPNO                    2
PROCNO                   1

F2 - Acquisition Parameters
Date_             20201215
Time                  0.47
INSTRUM            cryo500
PROBHD    5 mm CPTCI 1H-
PULPROG               zg30
TD                   16022
SOLVENT              CDCl3
NS                       8
DS                       2
SWH               8012.820 Hz
FIDRES            0.500114 Hz
AQ               0.9997728 sec
RG                       8
DW                  62.400 usec
DE                    6.00 usec
TE                   298.0 K
D1              0.10000000 sec
MCREST    0 sec
MCWRK           0.01500000 sec

======== CHANNEL f1 ========
NUC1                    1H
P1                    9.75 usec
PL1                   1.60 dB
SFO1           500.2235015 MHz

F2 - Processing parameters
SI                   65536
SF             500.2200314 MHz
WDW                     EM
SSB       0
LB                    0.30 Hz
GB        0
PC                    2.00
```

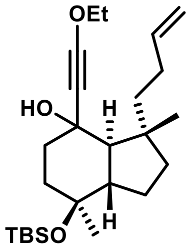

48, less polar diastereomer

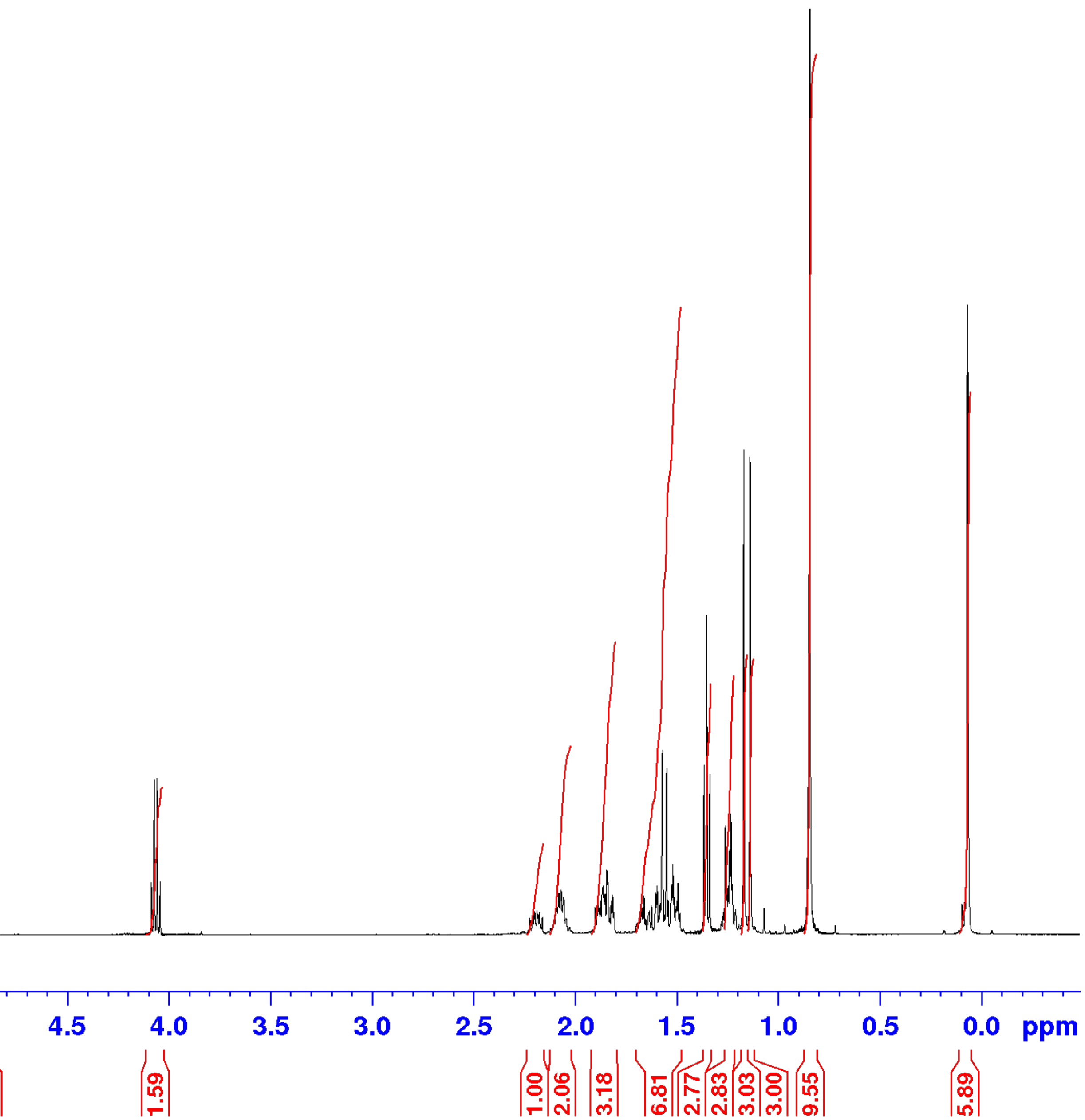

```
Current Data Parameters
NAME      JC 3-242 FRAC 6 col
EXPNO                    3
PROCNO                   1

F2 - Acquisition Parameters
Date_           20201215
Time                0.48
INSTRUM          cryo500
PROBHD   5 mm CPTCI 1H-
PULPROG  SpinEchopg30gp2.prd
TD                 65536
SOLVENT            CDCl3
NS                    68
DS                    16
SWH            30303.031 Hz
FIDRES          0.462388 Hz
AQ             1.0813440 sec
RG                7298.2
DW                16.500 usec
DE                  6.00 usec
TE                 298.0 K
D1            0.25000000 sec
d11           0.03000000 sec
D16           0.00020000 sec
d17           0.00019600 sec
MCREST   0 sec
MCWRK         0.01500000 sec
P2                 37.70 usec

======== CHANNEL f1 ========
NUC1                 13C
P1                 18.85 usec
P12              2000.00 usec
P20               500.00 usec
PL0               120.00 dB
PL1                -1.00 dB
SFO1         125.7942548 MHz
SP2                 1.55 dB
SP4                 1.55 dB
SPNAM[2]     Crp60comp.4
SPNAM[4]  Crp60,0.5,20.1
SPOFF2    0 Hz
SPOFF4    0 Hz

======== CHANNEL f2 ========
CPDPRG[2         waltz16
NUC2                  1H
PCPD2             100.00 usec
PL2                 1.60 dB
PL12               22.00 dB
SFO2         500.2225011 MHz

====== GRADIENT CHANNEL =====
GPNAM[1]       SINE.100
GPNAM[2]       SINE.100
GPX1      0 %
GPX2      0 %
GPY1      0 %
GPY2      0 %
GPZ1               30.00 %
GPZ2               50.00 %
p15               500.00 usec
p16              1000.00 usec

F2 - Processing parameters
SI                 65536
SF           125.7804058 MHz
WDW                   EM
SSB      0
LB                  1.00 Hz
GB       0
PC                  1.40
```

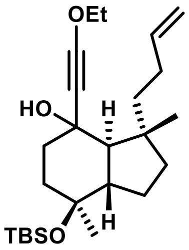

48, less polar diastereomer

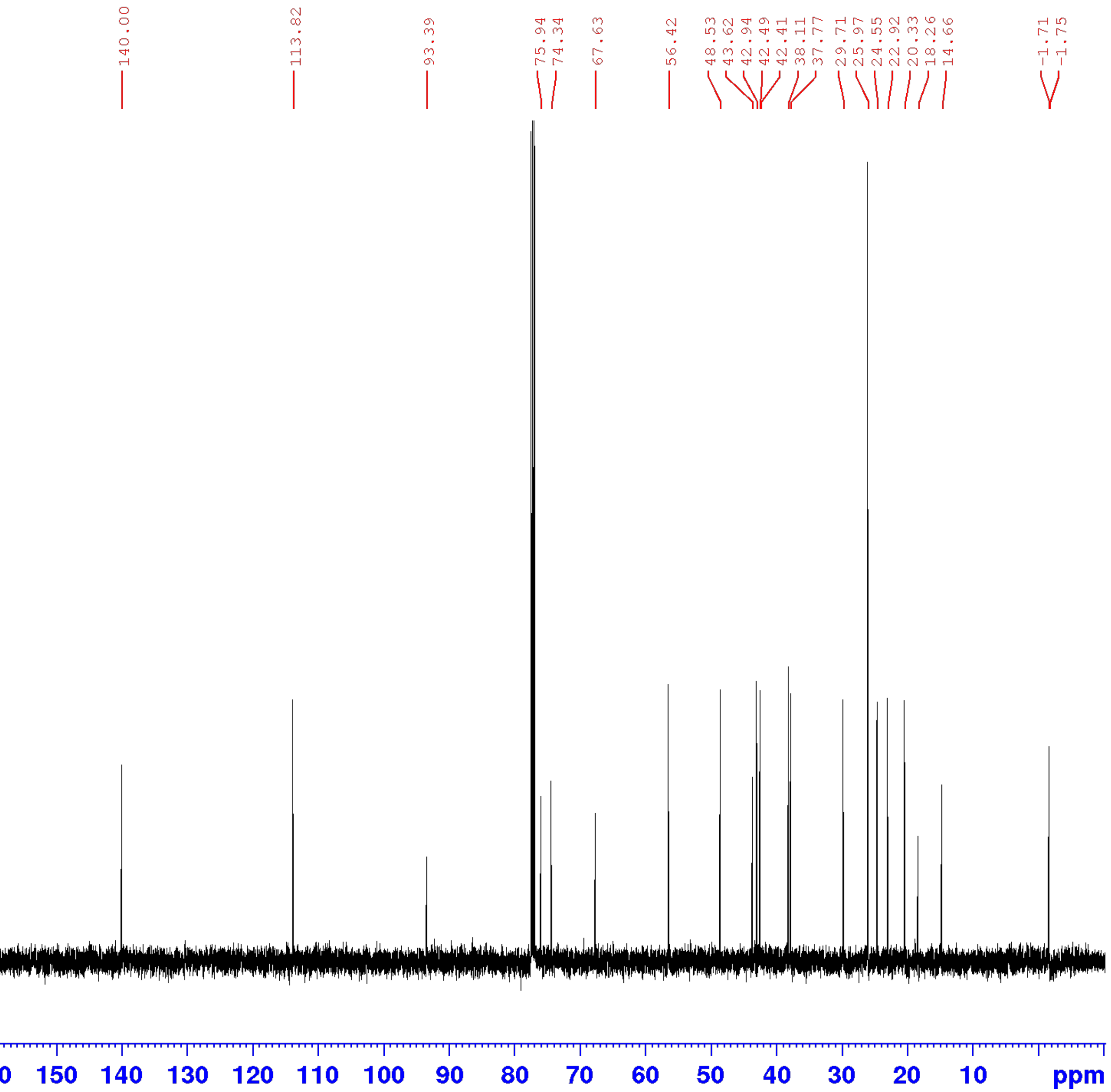

```
Current Data Parameters
NAME      JC 3-242 FRAC 12 col
EXPNO                    1
PROCNO                   1

F2 - Acquisition Parameters
Date_             20201215
Time                  0.54
INSTRUM            cryo500
PROBHD   5 mm CPTCI 1H-
PULPROG               zg30
TD                   16022
SOLVENT              CDCl3
NS                       8
DS                       2
SWH              8012.820 Hz
FIDRES           0.500114 Hz
AQ              0.9997728 sec
RG                     5.7
DW                  62.400 usec
DE                    6.00 usec
TE                   298.0 K
D1              0.10000000 sec
MCREST    0 sec
MCWRK           0.01500000 sec

======== CHANNEL f1 ========
NUC1                    1H
P1                    9.75 usec
PL1                   1.60 dB
SFO1           500.2235015 MHz

F2 - Processing parameters
SI                   65536
SF             500.2200314 MHz
WDW                     EM
SSB       0
LB                    0.30 Hz
GB        0
PC                    2.00
```

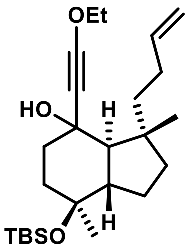

48, more polar diastereomer

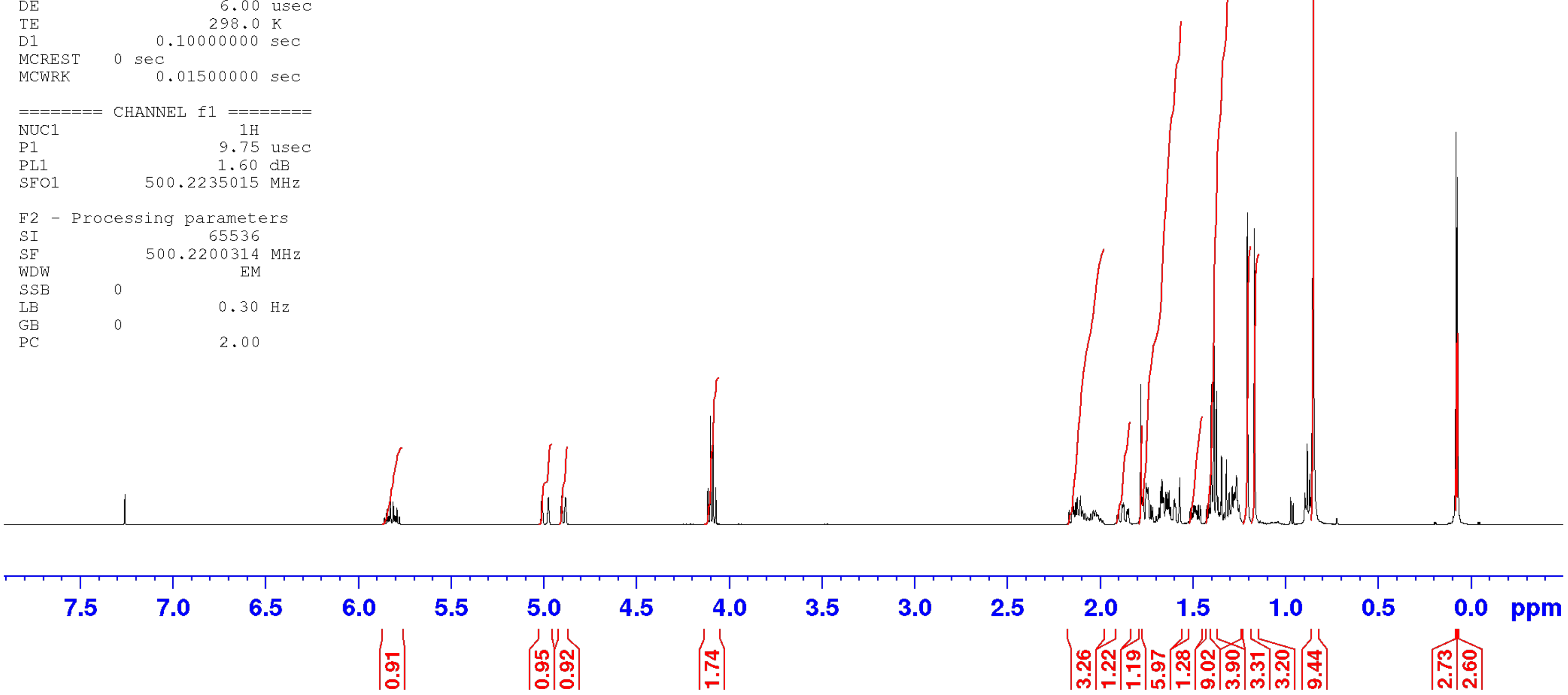

```
Current Data Parameters
NAME     JC 3-242 FRAC 12 col
EXPNO              2
PROCNO             1

F2 - Acquisition Parameters
Date_          20201215
Time               0.56
INSTRUM         cryo500
PROBHD   5 mm CPTCI 1H-
PULPROG  SpinEchopg30gp2.prd
TD                65536
SOLVENT           CDCl3
NS                  105
DS                   16
SWH           30303.031 Hz
FIDRES         0.462388 Hz
AQ            1.0813440 sec
RG                16384
DW               16.500 usec
DE                 6.00 usec
TE                298.0 K
D1           0.25000000 sec
d11          0.03000000 sec
D16          0.00020000 sec
d17          0.00019600 sec
MCREST   0 sec
MCWRK        0.01500000 sec
P2                37.70 usec

======== CHANNEL f1 ========
NUC1                13C
P1                18.85 usec
P12             2000.00 usec
P20              500.00 usec
PL0              120.00 dB
PL1               -1.00 dB
SFO1        125.7942548 MHz
SP2                1.55 dB
SP4                1.55 dB
SPNAM[2]    Crp60comp.4
SPNAM[4] Crp60,0.5,20.1
SPOFF2   0 Hz
SPOFF4   0 Hz

======== CHANNEL f2 ========
CPDPRG[2         waltz16
NUC2                 1H
PCPD2            100.00 usec
PL2                1.60 dB
PL12              22.00 dB
SFO2        500.2225011 MHz

====== GRADIENT CHANNEL =====
GPNAM[1]       SINE.100
GPNAM[2]       SINE.100
GPX1     0 %
GPX2     0 %
GPY1     0 %
GPY2     0 %
GPZ1              30.00 %
GPZ2              50.00 %
p15              500.00 usec
p16             1000.00 usec

F2 - Processing parameters
SI                65536
SF          125.7804062 MHz
WDW                  EM
SSB      0
LB                 1.00 Hz
GB       0
PC                 2.00
```

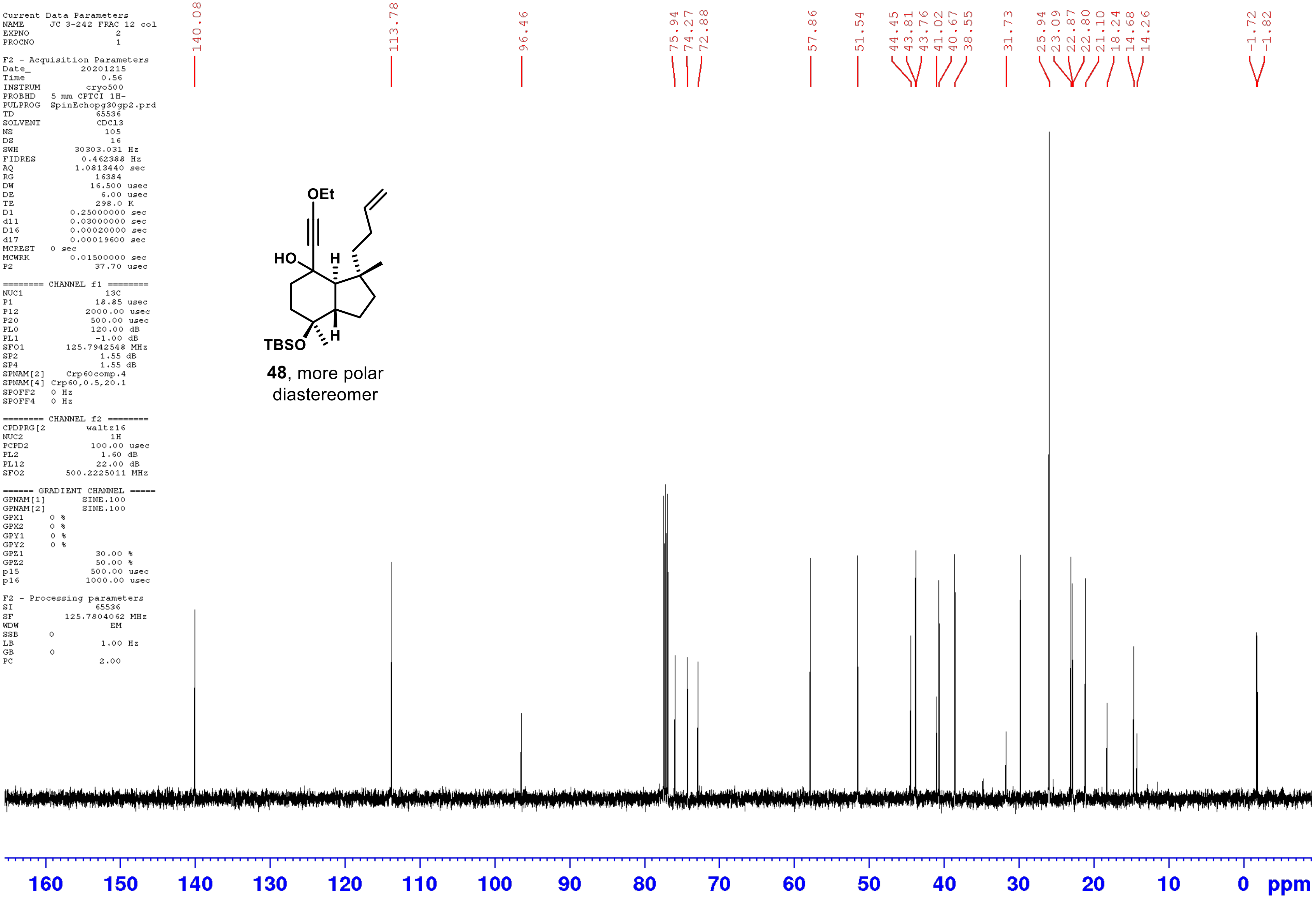

48, more polar diastereomer

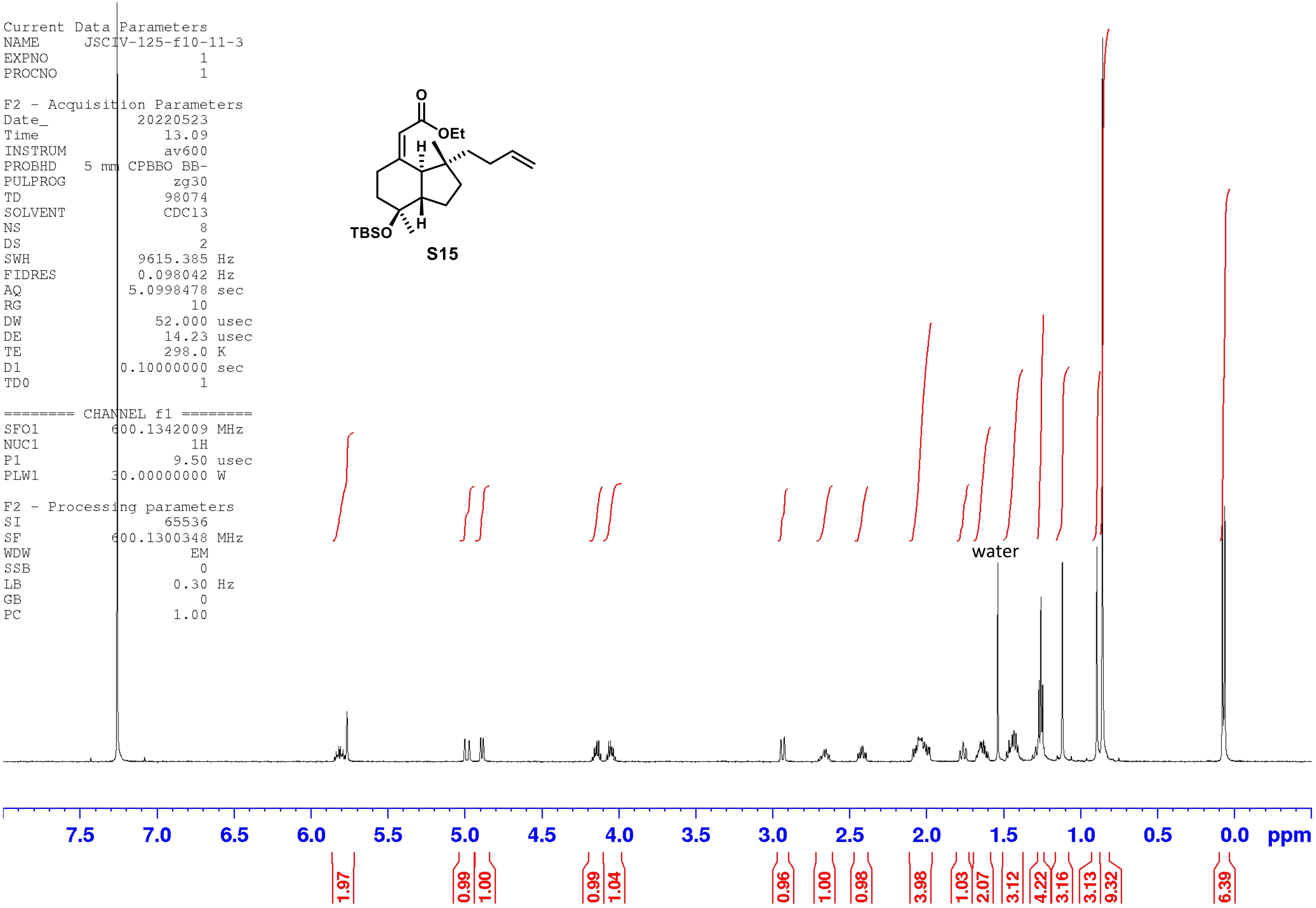

Current Data Parameters
NAME JSCIV-125-f10-11-3
EXPNO 1
PROCNO 1
F2 - Acquisition Parameters
Date_ 20220523
Time 13.09
INSTRUM av600
PROBHD 5 mm CPBBO BB-
PULPROG zg30
TD 98074
SOLVENT CDCl3
NS 8
DS 2
SWH 9615.385 Hz
FIDRES 0.098042 Hz
AQ 5.0998478 sec
RG 10
DW 52.000 usec
DE 14.23 usec
TE 298.0 K
D1 0.10000000 sec
TD0 1
======== CHANNEL f1 ========
SFO1 600.1342009 MHz
NUC1 1H
P1 9.50 usec
PLW1 30.00000000 W
F2 - Processing parameters
SI 65536
SF 600.1300348 MHz
WDW EM
SSB 0
LB 0.30 Hz
GB 0
PC 1.00
O
OEt
H
H
TBSO
S15
water
7.5 7.0 6.5 6.0 5.5 5.0 4.5 4.0 3.5 3.0 2.5 2.0 1.5 1.0 0.5 0.0 ppm
1.97 0.99 1.00 0.99 1.04 0.96 1.00 0.98 3.98 1.03 2.07 3.12 4.22 3.16 3.13 9.32 6.39

Current Data Parameters
NAME JSCIV-125-f10-11-3
EXPNO 2
PROCNO 1

F2 - Acquisition Parameters
Date_ 20220523
Time 13.13
INSTRUM av600
PROBHD 5 mm CPBBO BB-
PULPROG zgdc30
TD 65536
SOLVENT CDCl3
NS 292
DS 4
SWH 36231.883 Hz
FIDRES 0.552855 Hz
AQ 0.9043968 sec
RG 2050
DW 13.800 usec
DE 19.63 usec
TE 298.0 K
D1 0.40000001 sec
D11 0.03000000 sec
TD0 1

======== CHANNEL f1 ========
SFO1 150.9194080 MHz
NUC1 13C
P1 10.10 usec
PLW1 64.00000000 W

======== CHANNEL f2 ========
SFO2 600.1330010 MHz
NUC2 1H
CPDPRG[2 waltz16
PCPD2 80.00 usec
PLW2 30.00000000 W
PLW12 0.37154001 W

F2 - Processing parameters
SI 65536
SF 150.9027930 MHz
WDW EM
SSB 0
LB 1.00 Hz
GB 0
PC 1.00

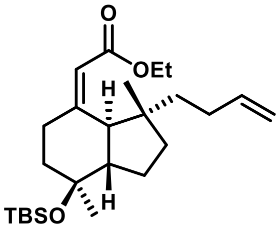

S15

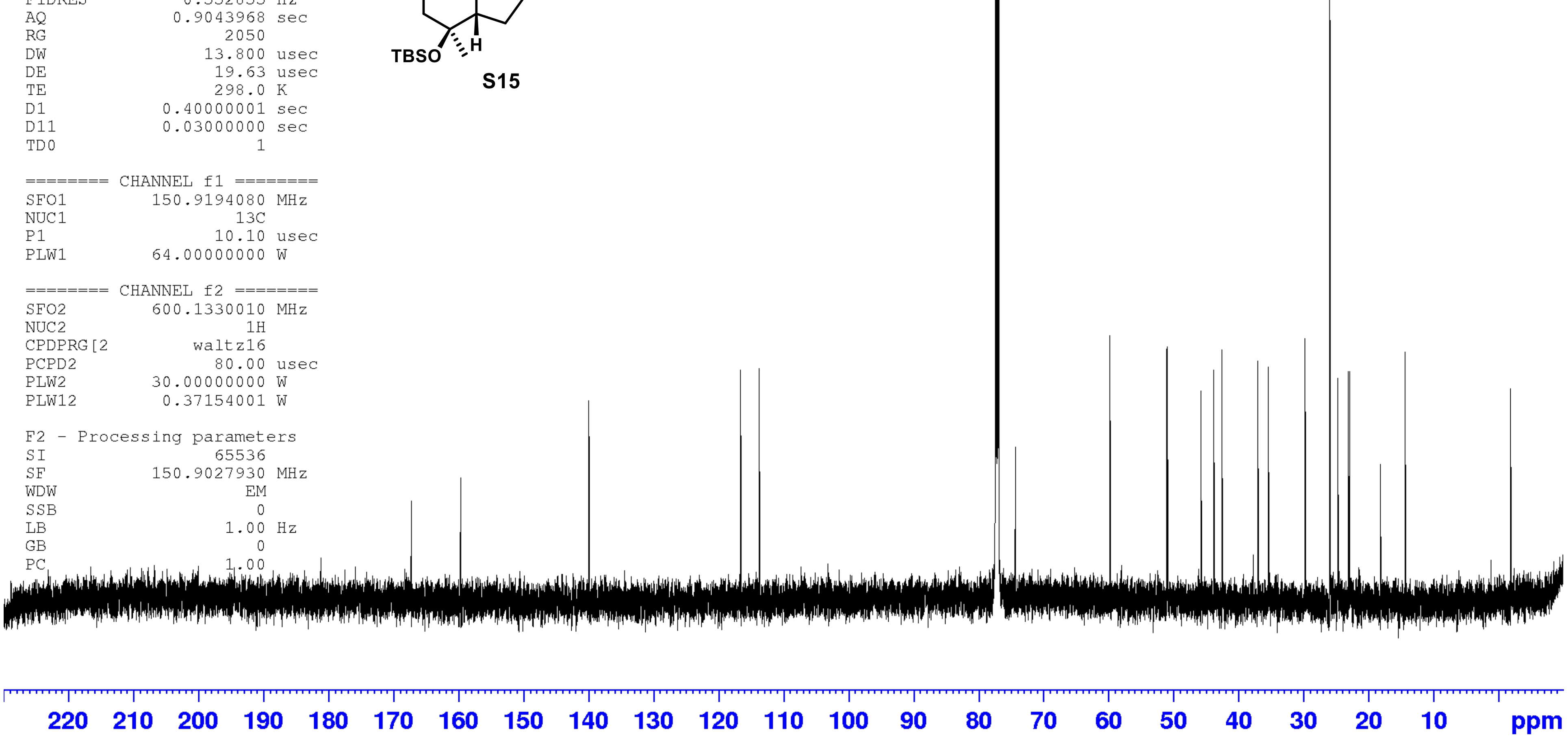

```
Current Data Parameters
NAME     JSCIV-125-f10-11-2
EXPNO                   2
PROCNO                  1

F2 - Acquisition Parameters
Date_          20220523
Time              10.53
INSTRUM         cryo500
PROBHD   5 mm CPTCI 1H-
PULPROG     cosygp60.prd
TD                 2048
SOLVENT           CDCl3
NS                    2
DS                   16
SWH            8012.820 Hz
FIDRES         3.912510 Hz
AQ            0.1277952 sec
RG                  512
DW               62.400 usec
DE                 6.00 usec
TE                298.0 K
d0           0.00000300 sec
D1           1.00000000 sec
d13          0.00000300 sec
D16          0.00020000 sec
IN0          0.00012480 sec

======== CHANNEL f1 ========
NUC1                 1H
P1                 9.75 usec
PL1                1.60 dB
SFO1        500.2235015 MHz

====== GRADIENT CHANNEL =====
GPNAM[1]      SMSQ10.100
GPNAM[2]      SMSQ10.100
GPX1                  0 %
GPX2                  0 %
GPY1                  0 %
GPY2                  0 %
GPZ1              17.00 %
GPZ2              17.00 %
P16             1000.00 usec

F1 - Acquisition parameters
TD                  469
SFO1           500.2235 MHz
FIDRES        34.169811 Hz
SW               16.018 ppm
FnMODE               QF

F2 - Processing parameters
SI                 1024
SF          500.2200300 MHz
WDW                SINE
SSB                   0
LB                    0 Hz
GB                    0
PC                 1.00

F1 - Processing parameters
SI                 1024
MC2                  QF
SF          500.2200314 MHz
WDW                SINE
SSB                   0
LB                    0 Hz
GB                    0
```

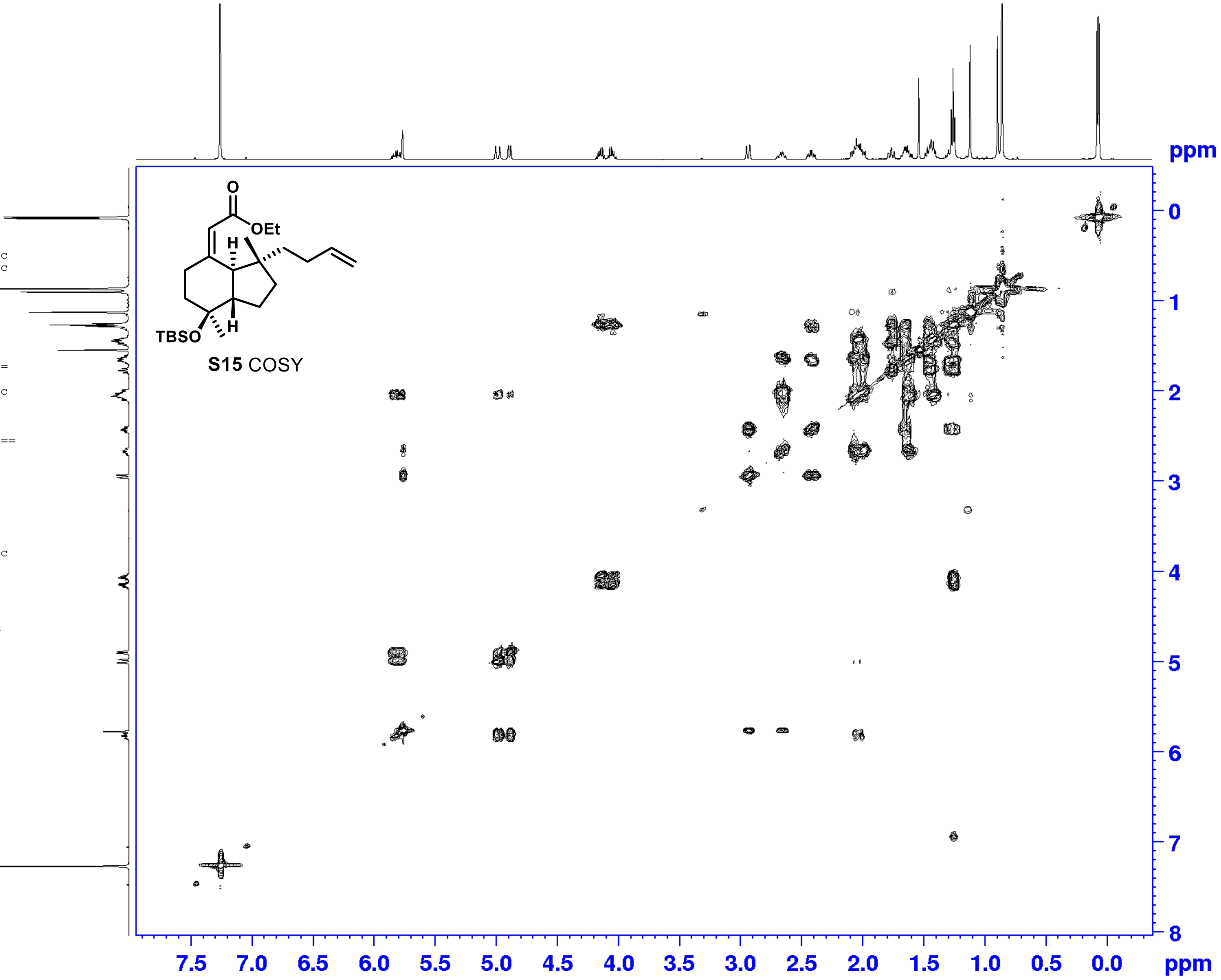

```
Current Data Parameters
NAME      JSCIV-125-f10-11-3
EXPNO                      3
PROCNO                     1

F2 - Acquisition Parameters
Date_               20220523
Time                   13.17
INSTRUM                av600
PROBHD     5 mm CPBBO BB-
PULPROG      hsqcetgpsp.2
TD                      1024
SOLVENT                CDCl3
NS                         2
DS                        16
SWH                 9615.385 Hz
FIDRES              9.390024 Hz
AQ                 0.0532480 sec
RG                      2050
DW                    52.000 usec
DE                     10.00 usec
TE                     298.0 K
CNST2            145.0000000
D0                0.00000300 sec
D1                1.10000002 sec
D4                0.00172414 sec
D11               0.03000000 sec
D16               0.00020000 sec
IN0               0.00001380 sec
ZGOPTNS

======== CHANNEL f1 ========
SFO1             600.1342009 MHz
NUC1                      1H
P1                     10.00 usec
P2                     20.00 usec
P28                  1000.00 usec
PLW1             30.00000000 W

======== CHANNEL f2 ========
SFO2             150.9194083 MHz
NUC2                     13C
CPDPRG[2                garp
P3                     10.10 usec
P14                   500.00 usec
P24                  2000.00 usec
PCPD2                  65.00 usec
PLW0                       0 W
PLW2             64.00000000 W
PLW12             1.47909999 W
SPNAM[3]  Crp60,0.5,20.1
SPOAL3                 0.500
SPOFFS3                    0 Hz
SPW3             10.00000000 W
SPNAM[7]      Crp60comp.4
SPOAL7                 0.500
SPOFFS7                    0 Hz
SPW7             10.00000000 W

====== GRADIENT CHANNEL =====
GPNAM[1]       SMSQ10.100
GPNAM[2]       SMSQ10.100
GPZ1                   80.00 %
GPZ2                   20.10 %
P16                  1000.00 usec

F1 - Acquisition parameters
TD                       256
SFO1                150.9194 MHz
FIDRES            283.061584 Hz
SW                   240.074 ppm
FnMODE        Echo-Antiecho

F2 - Processing parameters
SI                      1024
SF               600.1300278 MHz
WDW                       EM
SSB                        0
LB                      5.00 Hz
GB                         0
PC                      1.00

F1 - Processing parameters
SI                      1024
MC2            echo-antiecho
SF               150.9028143 MHz
WDW                    QSINE
SSB                        3
LB                         0 Hz
GB                         0
```

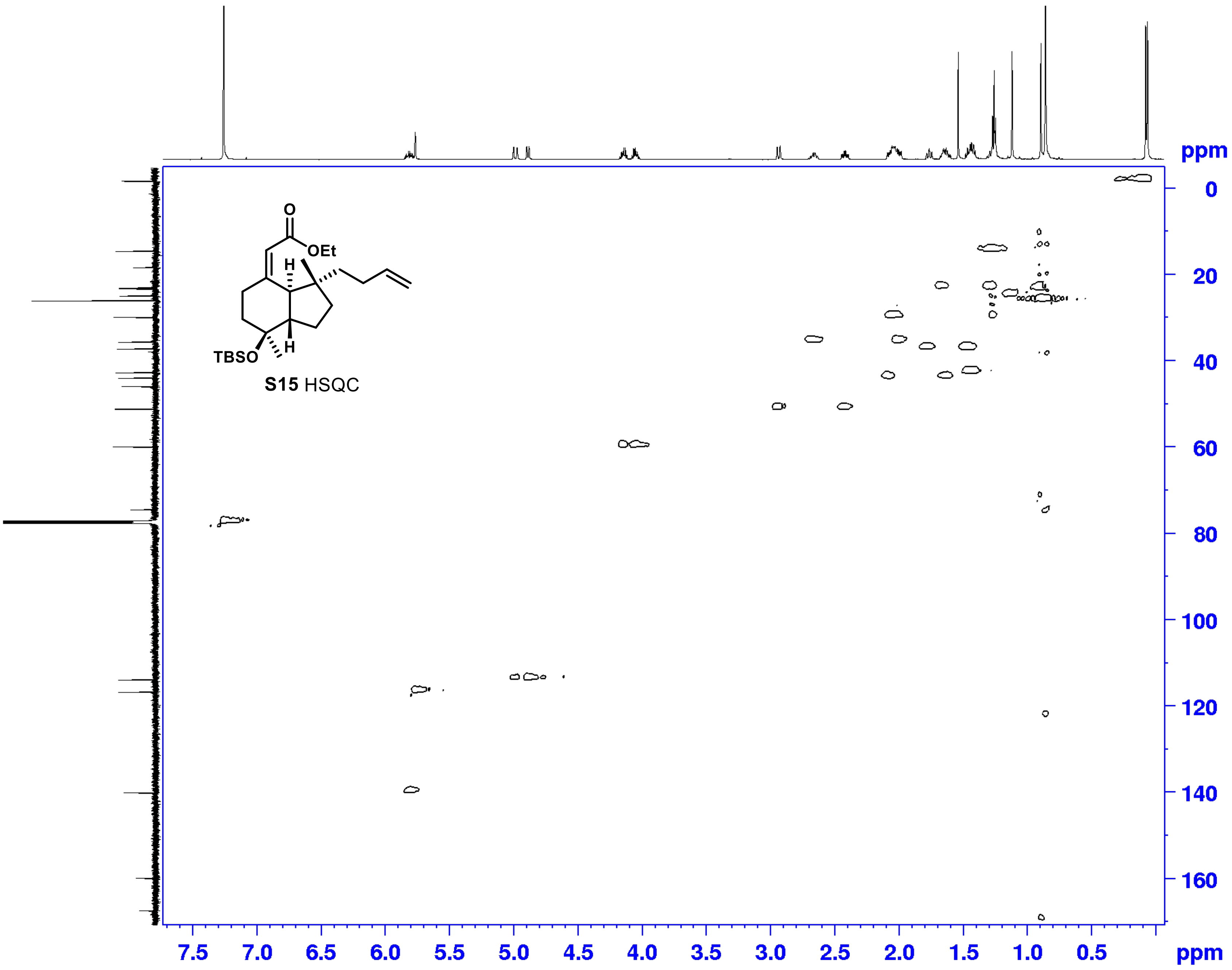

```
Current Data Parameters
NAME      JSCIV-125-f10-11-3
EXPNO                  4
PROCNO                 1

F2 - Acquisition Parameters
Date_           20220523
Time               13.27
INSTRUM            av600
PROBHD   5 mm CPBBO BB-
PULPROG    hmbcgplpndqf
TD                  4096
SOLVENT            CDCl3
NS                     2
DS                    16
SWH            9615.385 Hz
FIDRES         2.347506 Hz
AQ            0.2129920 sec
RG                  2050
DW                52.000 usec
DE                 10.00 usec
TE                 298.0 K
CNST2       145.0000000
CNST13       10.0000000
D0           0.00000300 sec
D1           1.10000002 sec
D2           0.00344828 sec
D6           0.05000000 sec
D16          0.00020000 sec
IN0          0.00001380 sec

======== CHANNEL f1 ========
SFO1        600.1342009 MHz
NUC1                 1H
P1                 9.50 usec
P2                19.00 usec
PLW1        30.00000000 W

======== CHANNEL f2 ========
SFO2        150.9194083 MHz
NUC2                13C
P3                10.10 usec
PLW2        64.00000000 W

====== GRADIENT CHANNEL =====
GPNAM[1]      SMSQ10.100
GPNAM[2]      SMSQ10.100
GPNAM[3]      SMSQ10.100
GPZ1               50.00 %
GPZ2               30.00 %
GPZ3               40.10 %
P16              1000.00 usec

F1 - Acquisition parameters
TD                   256
SFO1            150.9194 MHz
FIDRES        283.061584 Hz
SW               240.074 ppm
FnMODE                QF

F2 - Processing parameters
SI                  2048
SF           600.1300327 MHz
WDW                 SINE
SSB                    0
LB                     0 Hz
GB                     0
PC                  1.00

F1 - Processing parameters
SI                  1024
MC2                   QF
SF           150.9028014 MHz
WDW                 SINE
SSB                    0
LB                     0 Hz
GB                     0
```

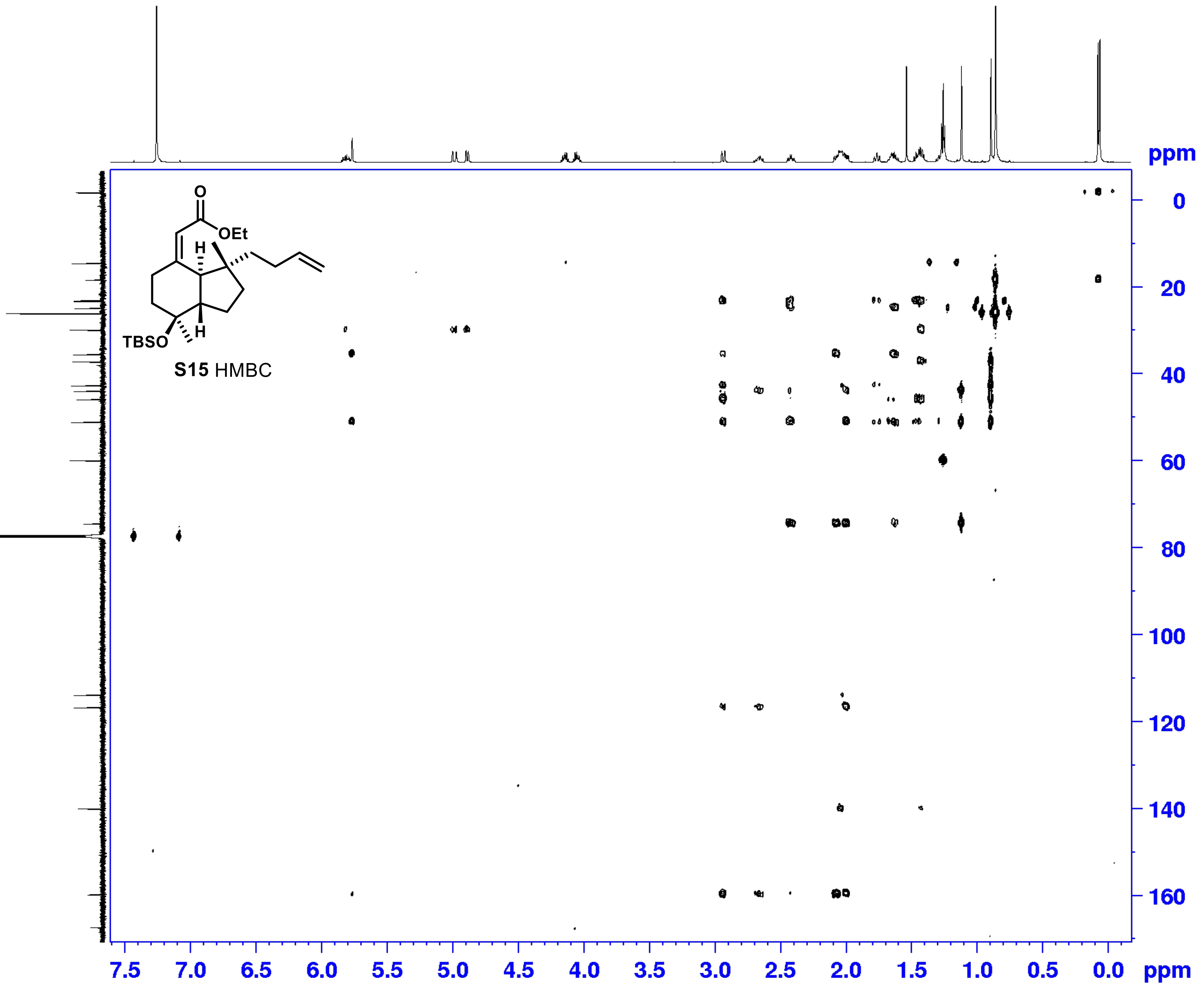

```
Current Data Parameters
NAME      JSCIV-125-f10-11-3
EXPNO                   5
PROCNO                  1

F2 - Acquisition Parameters
Date_            20220523
Time                13.40
INSTRUM             av600
PROBHD   5 mm CPBBO BB-
PULPROG        noesygpph
TD                   2048
SOLVENT             CDCl3
NS                      2
DS                     16
SWH              9615.385 Hz
FIDRES           4.695012 Hz
AQ              0.1064960 sec
RG                     57
DW                 52.000 usec
DE                  16.68 usec
TE                  297.9 K
D0             0.00003990 sec
D1             2.00000000 sec
D8             1.00000000 sec
D16            0.00020000 sec
IN0            0.00010400 sec

======== CHANNEL f1 ========
SFO1       600.1342009 MHz
NUC1                 1H
P1                 9.50 usec
P2                19.00 usec
PLW1        30.00000000 W

====== GRADIENT CHANNEL ====
GPNAM[1]       SMSQ10.100
GPZ1                40.00 %
P16               1000.00 usec

F1 - Acquisition parameters
TD                    256
SFO1             600.1342 MHz
FIDRES          75.120193 Hz
SW                 16.022 ppm
FnMODE        States-TPPI

F2 - Processing parameters
SI                   1024
SF            600.1300238 MHz
WDW                 QSINE
SSB                     2
LB                      0 Hz
GB                      0
PC                   1.00

F1 - Processing parameters
SI                   1024
MC2           States-TPPI
SF            600.1300385 MHz
WDW                 QSINE
SSB                     2
LB                      0 Hz
GB                      0
```

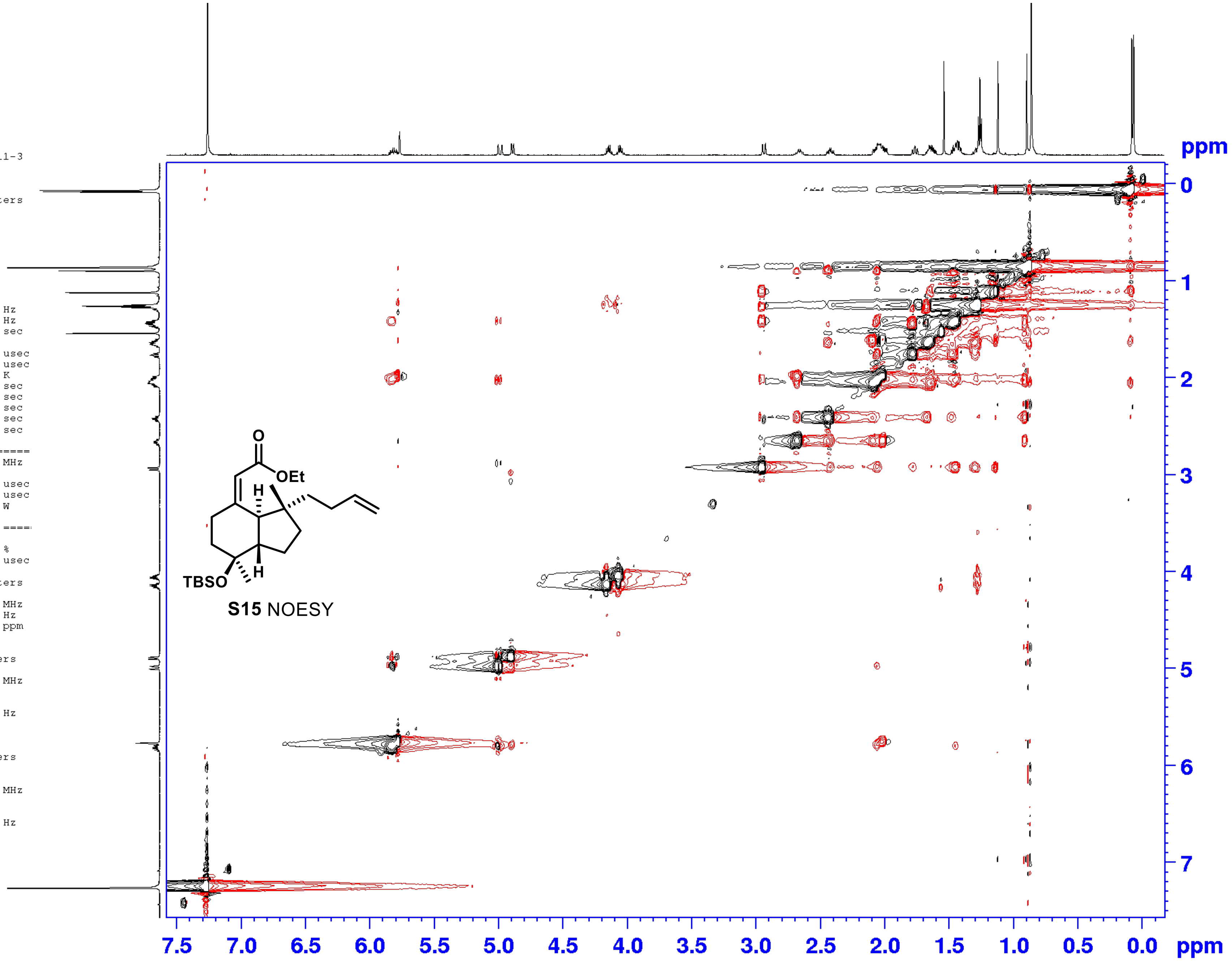

S15 NOESY

```
Current Data Parameters
NAME      JSCIV-125-f19-27-2
EXPNO                  1
PROCNO                 1

F2 - Acquisition Parameters
Date_            20220523
Time                15.26
INSTRUM             av600
PROBHD    5 mm CPBBO BB-
PULPROG              zg30
TD                  98074
SOLVENT             CDCl3
NS                      8
DS                      2
SWH              9615.385 Hz
FIDRES           0.098042 Hz
AQ              5.0998478 sec
RG                     10
DW                 52.000 usec
DE                  14.23 usec
TE                  297.9 K
D1             0.10000000 sec
TD0                     1

======== CHANNEL f1 ========
SFO1         600.1342009 MHz
NUC1                   1H
P1                   9.50 usec
PLW1          30.00000000 W

F2 - Processing parameters
SI                  65536
SF            600.1300353 MHz
WDW                    EM
SSB                     0
LB                   0.30 Hz
GB                      0
PC                   1.00
```

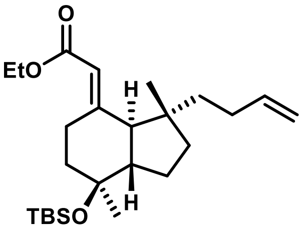

49

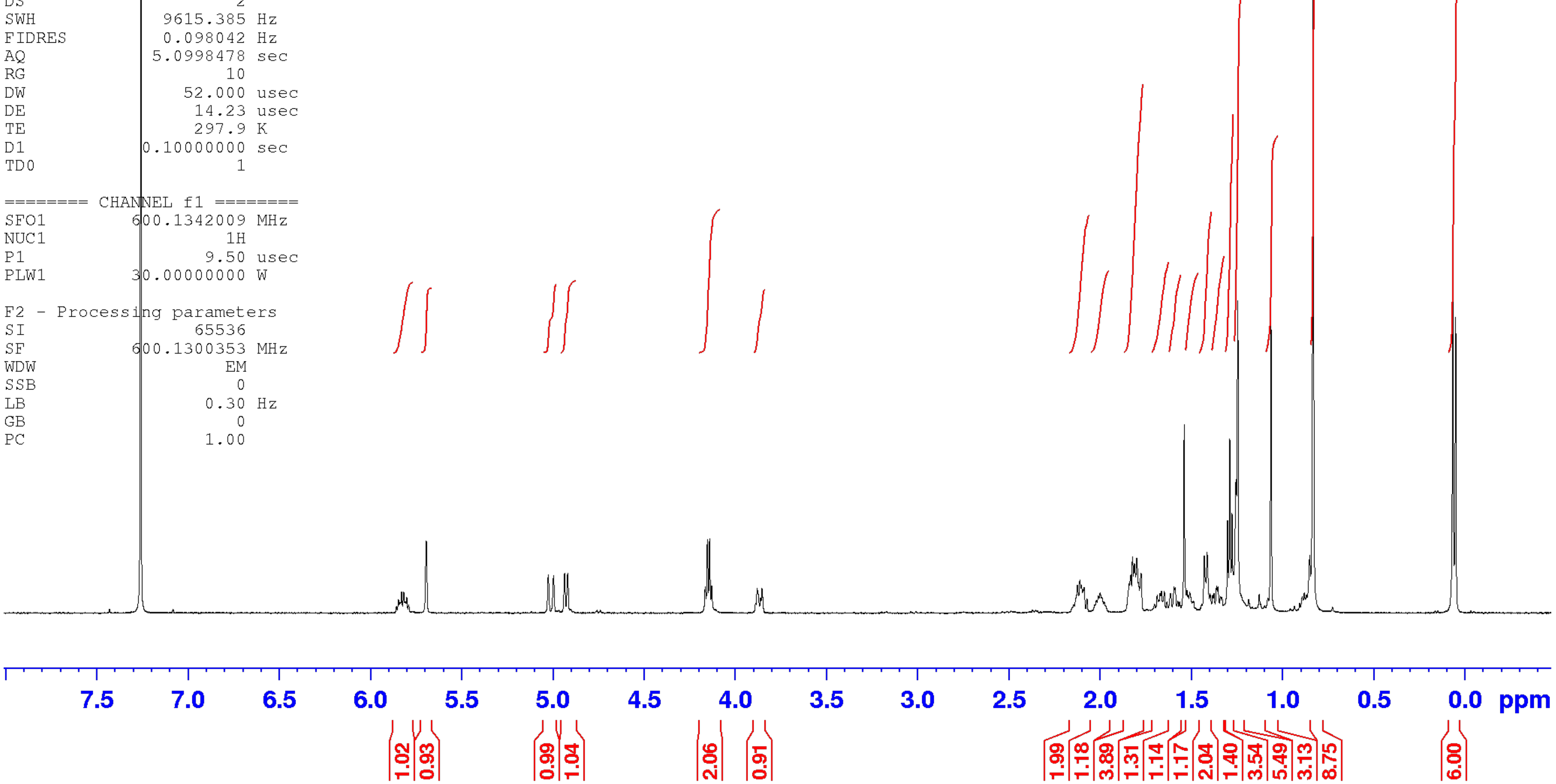

Current Data Parameters
NAME JSCIV-125-f19-27-2
EXPNO 2
PROCNO 1

F2 - Acquisition Parameters
Date_ 20220523
Time 15.28
INSTRUM av600
PROBHD 5 mm CPBBO BB-
PULPROG zgdc30
TD 65536
SOLVENT CDCl3
NS 200
DS 4
SWH 36231.883 Hz
FIDRES 0.552855 Hz
AQ 0.9043968 sec
RG 2050
DW 13.800 usec
DE 19.63 usec
TE 298.0 K
D1 0.40000001 sec
D11 0.03000000 sec
TD0 1

======== CHANNEL f1 ========
SFO1 150.9194080 MHz
NUC1 13C
P1 10.10 usec
PLW1 64.00000000 W

======== CHANNEL f2 ========
SFO2 600.1330010 MHz
NUC2 1H
CPDPRG[2 waltz16
PCPD2 80.00 usec
PLW2 30.00000000 W
PLW12 0.37154001 W

F2 - Processing parameters
SI 65536
SF 150.9027928 MHz
WDW EM
SSB 0
LB 1.00 Hz
GB 0
PC 1.00

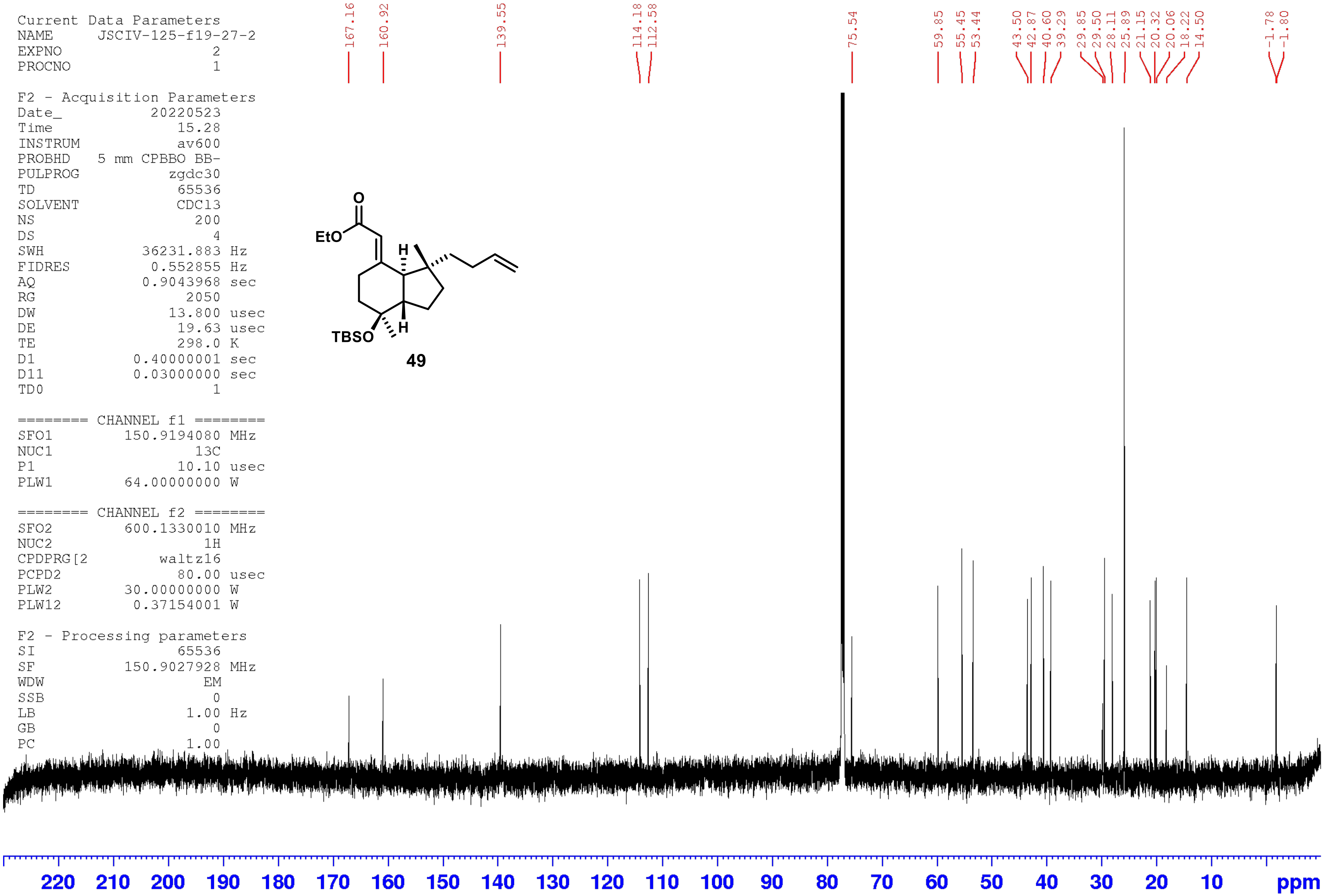

```
Current Data Parameters
NAME      JSCIV-125-f19-27-2
EXPNO                    4
PROCNO                   1

F2 - Acquisition Parameters
Date_           20220523
Time               15.40
INSTRUM            av600
PROBHD    5 mm CPBBO BB-
PULPROG         cosygpqf
TD                  2048
SOLVENT            CDCl3
NS                     1
DS                    16
SWH             9615.385 Hz
FIDRES          4.695012 Hz
AQ             0.1064960 sec
RG                   645
DW                52.000 usec
DE                 10.00 usec
TE                 297.9 K
D0            0.00000300 sec
D1            1.48689198 sec
D13           0.00000400 sec
D16           0.00020000 sec
IN0           0.00010400 sec

======== CHANNEL f1 ========
SFO1         600.1342009 MHz
NUC1                  1H
P0                  9.50 usec
P1                  9.50 usec
PLW1         30.00000000 W

====== GRADIENT CHANNEL =====
GPNAM[1]      SMSQ10.100
GPZ1               10.00 %
P16              1000.00 usec

F1 - Acquisition parameters
TD                   256
SFO1            600.1342 MHz
FIDRES         75.120193 Hz
SW                16.022 ppm
FnMODE                QF

F2 - Processing parameters
SI                  1024
SF           600.1300328 MHz
WDW                 SINE
SSB                    0
LB                     0 Hz
GB                     0
PC                  1.00

F1 - Processing parameters
SI                  1024
MC2                   QF
SF           600.1300336 MHz
WDW                 SINE
SSB                    0
LB                     0 Hz
GB                     0
```

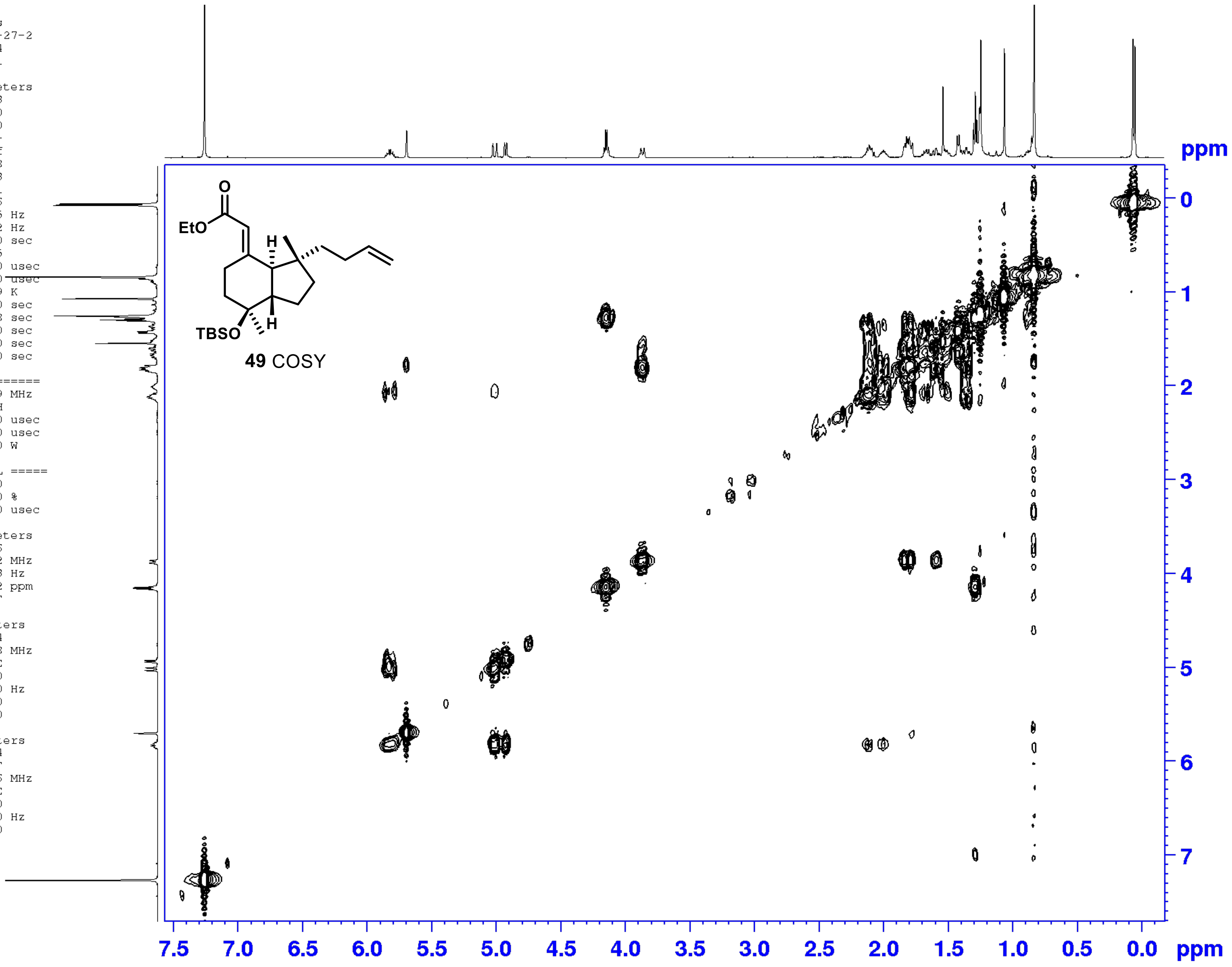

```
Current Data Parameters
NAME     JSCIV-125-f19-27-2
EXPNO                     5
PROCNO                    1

F2 - Acquisition Parameters
Date_              20220523
Time                  15.48
INSTRUM               av600
PROBHD   5 mm CPBBO BB-
PULPROG     hsqcetgpsp.2
TD                     1024
SOLVENT               CDCl3
NS                        2
DS                       16
SWH              9615.385 Hz
FIDRES           9.390024 Hz
AQ              0.0532480 sec
RG                     2050
DW                   52.000 usec
DE                    10.00 usec
TE                    297.9 K
CNST2           145.0000000
D0               0.00000300 sec
D1               1.10000002 sec
D4               0.00172414 sec
D11              0.03000000 sec
D16              0.00020000 sec
IN0              0.00001380 sec
ZGOPTNS

======== CHANNEL f1 ========
SFO1         600.1342009 MHz
NUC1                     1H
P1                    10.00 usec
P2                    20.00 usec
P28                 1000.00 usec
PLW1            30.00000000 W

======== CHANNEL f2 ========
SFO2         150.9194083 MHz
NUC2                    13C
CPDPRG[2               garp
P3                    10.10 usec
P14                  500.00 usec
P24                 2000.00 usec
PCPD2                 65.00 usec
PLW0                      0 W
PLW2            64.00000000 W
PLW12            1.47909999 W
SPNAM[3] Crp60,0.5,20.1
SPOAL3                0.500
SPOFFS3                   0 Hz
SPW3            10.00000000 W
SPNAM[7]     Crp60comp.4
SPOAL7                0.500
SPOFFS7                   0 Hz
SPW7            10.00000000 W

====== GRADIENT CHANNEL =====
GPNAM[1]        SMSQ10.100
GPNAM[2]        SMSQ10.100
GPZ1                  80.00 %
GPZ2                  20.10 %
P16                 1000.00 usec

F1 - Acquisition parameters
TD                      256
SFO1               150.9194 MHz
FIDRES           283.061584 Hz
SW                  240.074 ppm
FnMODE       Echo-Antiecho

F2 - Processing parameters
SI                     1024
SF              600.1300346 MHz
WDW                      EM
SSB                       0
LB                     5.00 Hz
GB                        0
PC                     1.00

F1 - Processing parameters
SI                     1024
MC2          echo-antiecho
SF              150.9028122 MHz
WDW                   QSINE
SSB                       3
LB                        0 Hz
GB                        0
```

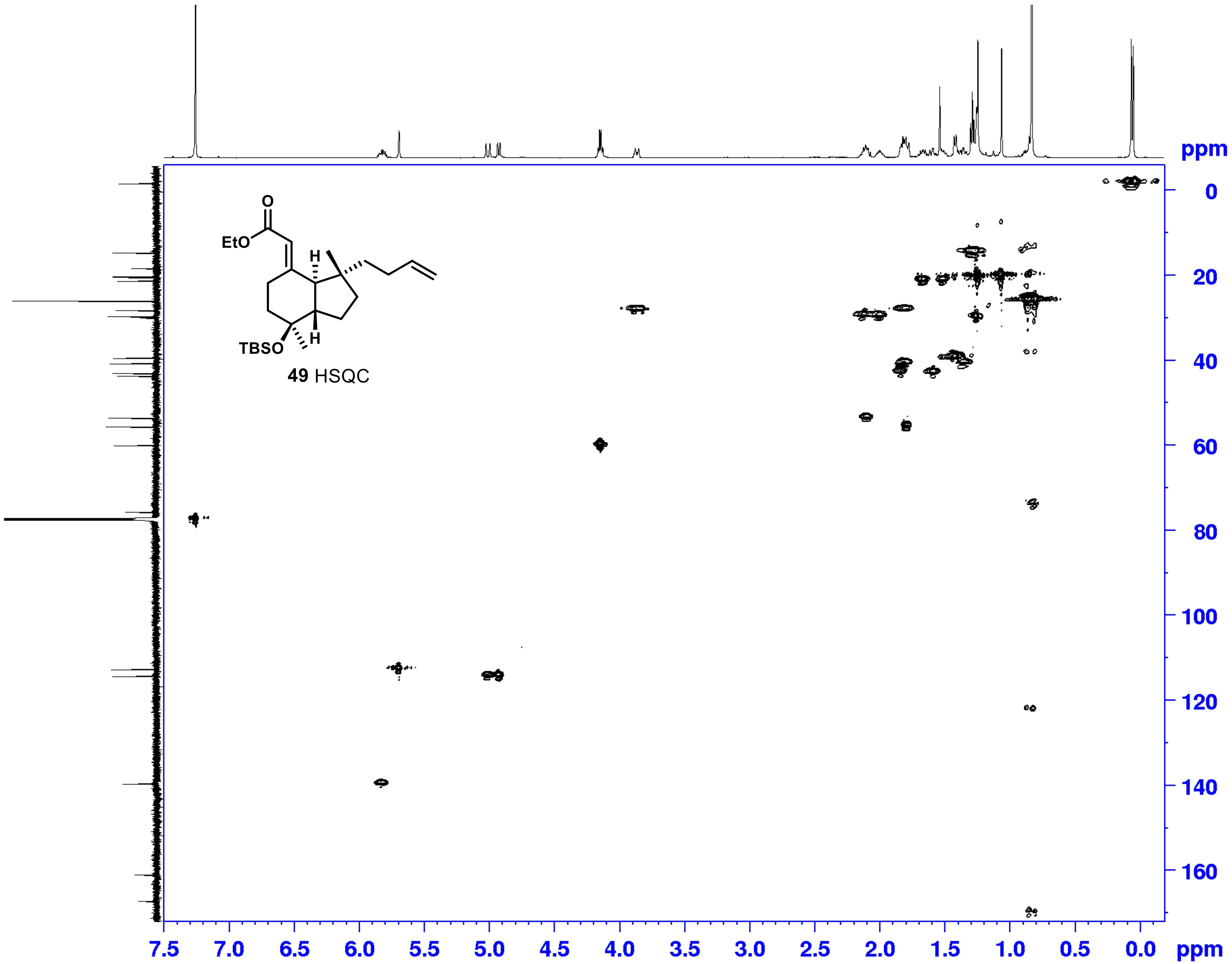

49 HSQC

```
Current Data Parameters
NAME      JSCIV-125-f19-27-2
EXPNO                  6
PROCNO                 1

F2 - Acquisition Parameters
Date_           20220523
Time               15.58
INSTRUM            av600
PROBHD   5 mm CPBBO BB-
PULPROG    hmbcgplpndqf
TD                  4096
SOLVENT            CDCl3
NS                     2
DS                    16
SWH             9615.385 Hz
FIDRES          2.347506 Hz
AQ             0.2129920 sec
RG                  2050
DW                52.000 usec
DE                 10.00 usec
TE                 297.9 K
CNST2        145.0000000
CNST13        10.0000000
D0            0.00000300 sec
D1            1.10000002 sec
D2            0.00344828 sec
D6            0.05000000 sec
D16           0.00020000 sec
IN0           0.00001380 sec

======== CHANNEL f1 ========
SFO1         600.1342009 MHz
NUC1                  1H
P1                  9.50 usec
P2                 19.00 usec
PLW1         30.00000000 W

======== CHANNEL f2 ========
SFO2         150.9194083 MHz
NUC2                 13C
P3                 10.10 usec
PLW2         64.00000000 W

====== GRADIENT CHANNEL =====
GPNAM[1]      SMSQ10.100
GPNAM[2]      SMSQ10.100
GPNAM[3]      SMSQ10.100
GPZ1               50.00 %
GPZ2               30.00 %
GPZ3               40.10 %
P16              1000.00 usec

F1 - Acquisition parameters
TD                   256
SFO1            150.9194 MHz
FIDRES        283.061584 Hz
SW               240.074 ppm
FnMODE                QF

F2 - Processing parameters
SI                  2048
SF           600.1300379 MHz
WDW                 SINE
SSB                    0
LB                     0 Hz
GB                     0
PC                  1.00

F1 - Processing parameters
SI                  1024
MC2                   QF
SF           150.9028170 MHz
WDW                 SINE
SSB                    0
LB                     0 Hz
GB                     0
```

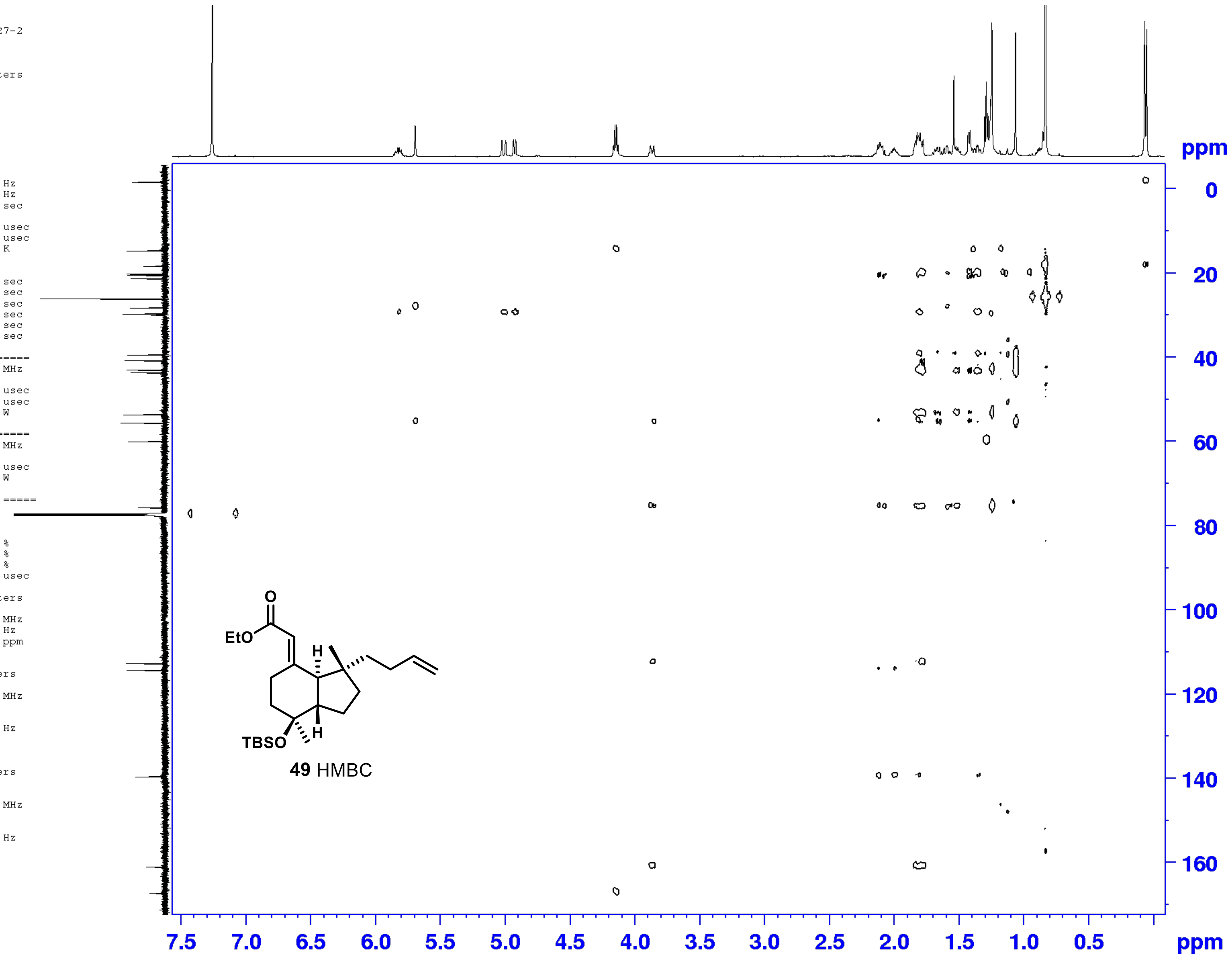

49 HMBC

```
Current Data Parameters
NAME     JSCIV-125-f19-27-2
EXPNO                  7
PROCNO                 1

F2 - Acquisition Parameters
Date_           20220523
Time               16.11
INSTRUM            av600
PROBHD    5 mm CPBBO BB-
PULPROG       noesygpph
TD                  2048
SOLVENT            CDCl3
NS                     2
DS                    16
SWH             9615.385 Hz
FIDRES          4.695012 Hz
AQ             0.1064960 sec
RG                    57
DW                52.000 usec
DE                 16.68 usec
TE                 297.9 K
D0            0.00003990 sec
D1            2.00000000 sec
D8            1.00000000 sec
D16           0.00020000 sec
IN0           0.00010400 sec

======== CHANNEL f1 ========
SFO1         600.1342009 MHz
NUC1                  1H
P1                  9.50 usec
P2                 19.00 usec
PLW1         30.00000000 W

====== GRADIENT CHANNEL =====
GPNAM[1]      SMSQ10.100
GPZ1               40.00 %
P16              1000.00 usec

F1 - Acquisition parameters
TD                   256
SFO1            600.1342 MHz
FIDRES         75.120193 Hz
SW                16.022 ppm
FnMODE       States-TPPI

F2 - Processing parameters
SI                  1024
SF           600.1300339 MHz
WDW                QSINE
SSB                    2
LB                     0 Hz
GB                     0
PC                  1.00

F1 - Processing parameters
SI                  1024
MC2          States-TPPI
SF           600.1300383 MHz
WDW                QSINE
SSB                    2
LB                     0 Hz
GB                     0
```

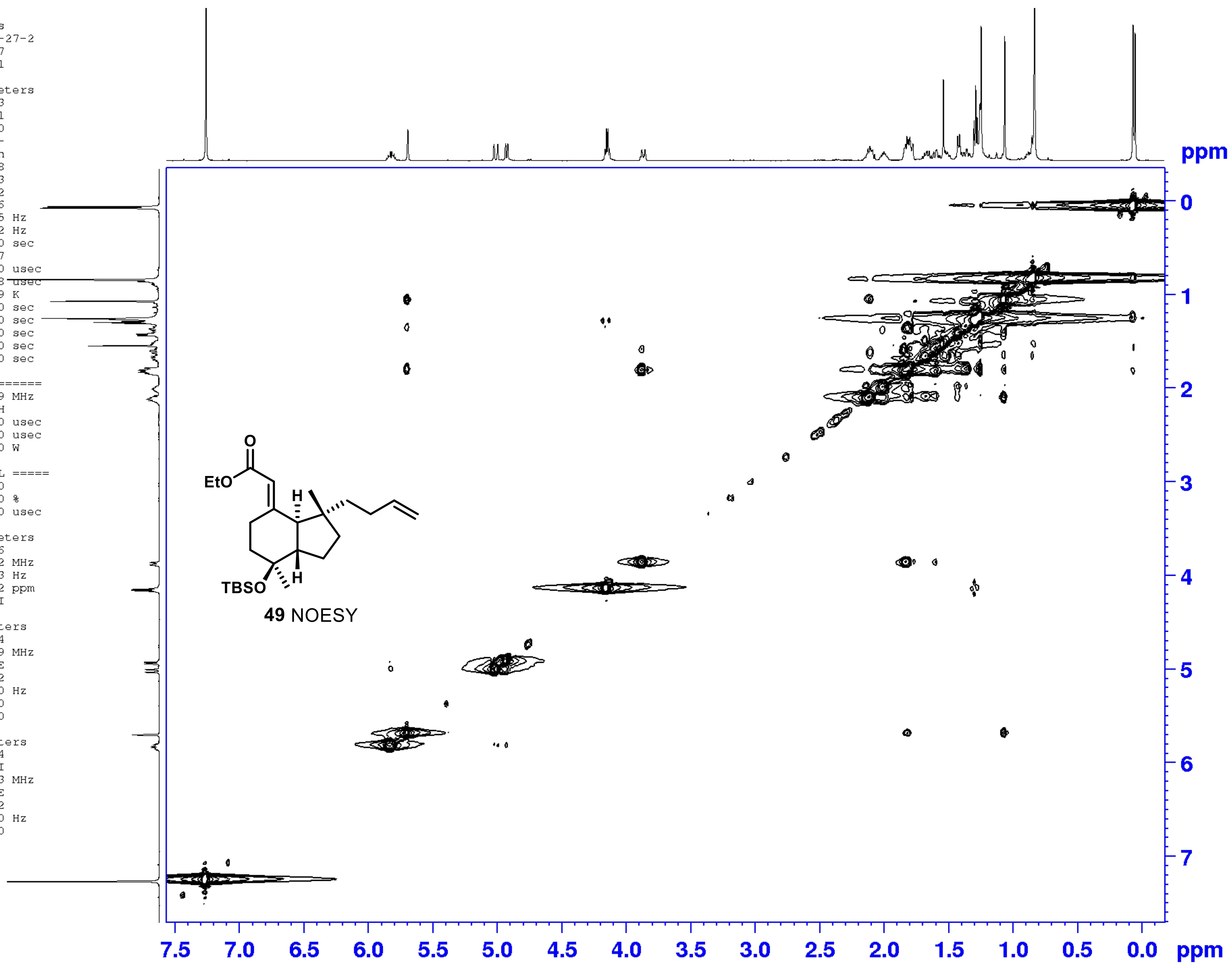

Current Data Parameters
NAME JSCIV-142-f29-38-2
EXPNO 1
PROCNO 1

F2 - Acquisition Parameters
Date_ 20220609
Time 13.55
INSTRUM av600
PROBHD 5 mm CPBBO BB-
PULPROG zg30
TD 98074
SOLVENT CDCl3
NS 8
DS 2
SWH 9615.385 Hz
FIDRES 0.098042 Hz
AQ 5.0998478 sec
RG 10
DW 52.000 usec
DE 14.23 usec
TE 298.0 K
D1 0.10000000 sec
TD0 1

======== CHANNEL f1 ========
SFO1 600.1342009 MHz
NUC1 1H
P1 9.50 usec
PLW1 30.00000000 W

F2 - Processing parameters
SI 65536
SF 600.1300351 MHz
WDW EM
SSB 0
LB 0.30 Hz
GB 0
PC 1.00

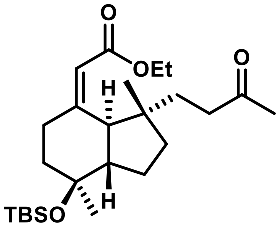

S16

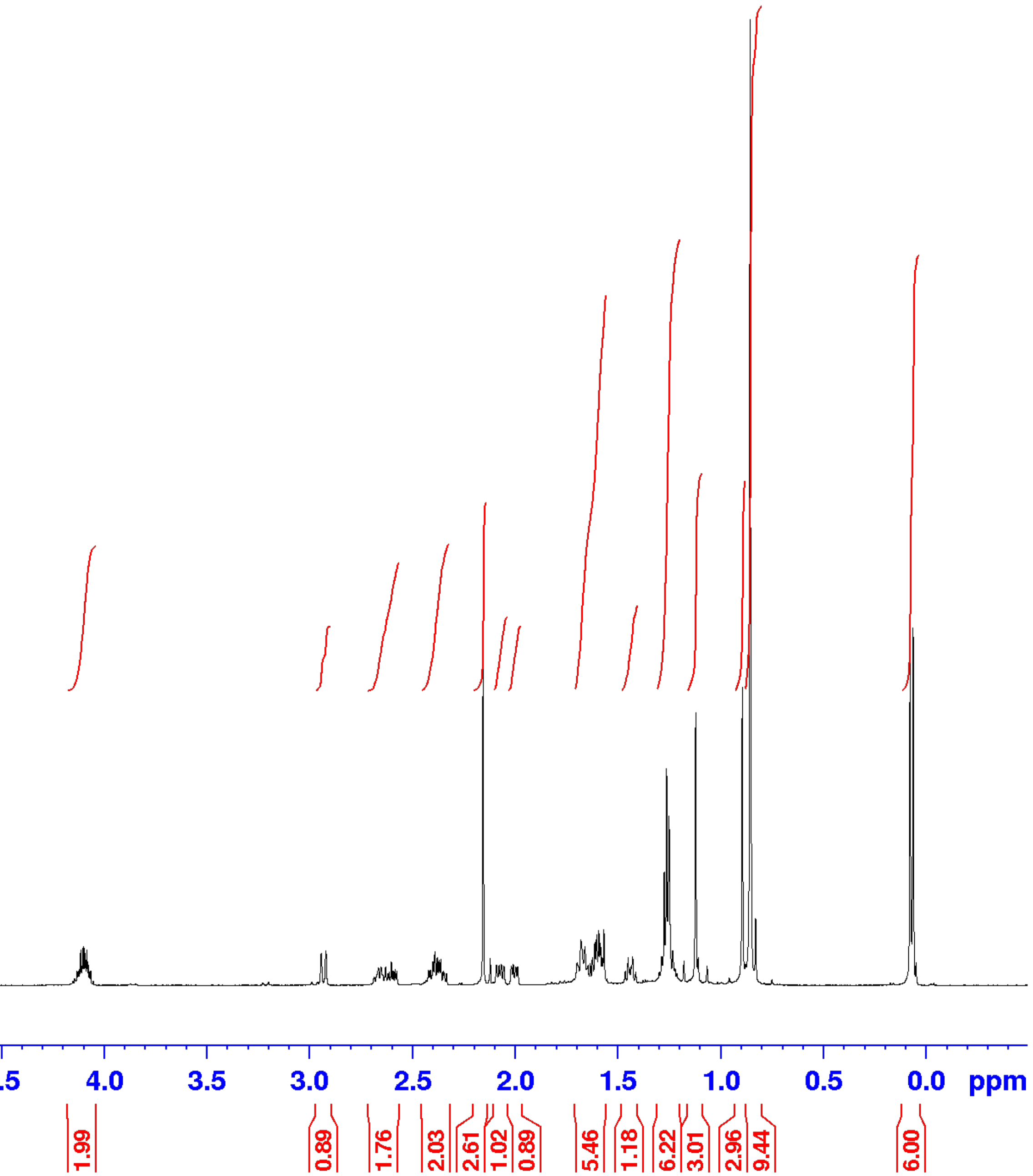

```
Current Data Parameters
NAME      JSCIV-142-f29-38-2
EXPNO                    2
PROCNO                   1

F2 - Acquisition Parameters
Date_             20220609
Time                 13.56
INSTRUM              av600
PROBHD    5 mm CPBBO BB-
PULPROG            zgdc30
TD                   65536
SOLVENT              CDCl3
NS                      65
DS                       4
SWH           36231.883 Hz
FIDRES         0.552855 Hz
AQ           0.9043968 sec
RG                    2050
DW              13.800 usec
DE               19.63 usec
TE                298.0 K
D1          0.40000001 sec
D11         0.03000000 sec
TD0                      1

======== CHANNEL f1 ========
SFO1        150.9194080 MHz
NUC1                   13C
P1               10.10 usec
PLW1        64.00000000 W

======== CHANNEL f2 ========
SFO2        600.1330010 MHz
NUC2                    1H
CPDPRG[2           waltz16
PCPD2            80.00 usec
PLW2        30.00000000 W
PLW12        0.37154001 W

F2 - Processing parameters
SI                   65536
SF          150.9027930 MHz
WDW                     EM
SSB                      0
LB                1.00 Hz
GB                       0
PC                    1.00
```

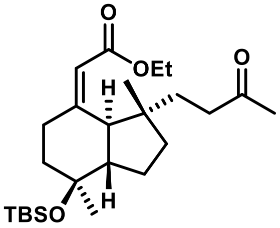

S16

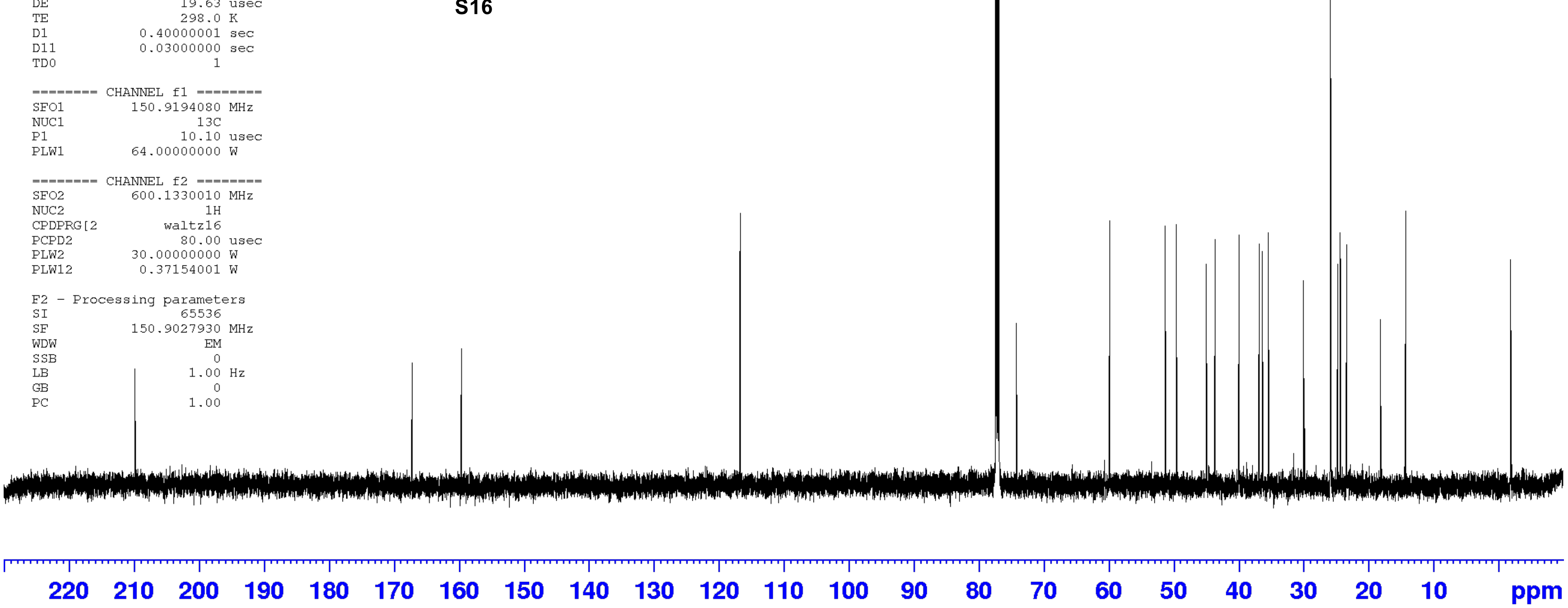

```
Current Data Parameters
NAME      JSCIV-142-f29-38-2
EXPNO                    5
PROCNO                   1

F2 - Acquisition Parameters
Date_             20220609
Time                 14.04
INSTRUM              av600
PROBHD   5 mm CPBBO BB-
PULPROG           cosygpqf
TD                    2048
SOLVENT              CDCl3
NS                       1
DS                      16
SWH               9615.385 Hz
FIDRES            4.695012 Hz
AQ               0.1064960 sec
RG                     256
DW                  52.000 usec
DE                   10.00 usec
TE                   297.9 K
D0              0.00000300 sec
D1              1.48689198 sec
D13             0.00000400 sec
D16             0.00020000 sec
IN0             0.00010400 sec

======== CHANNEL f1 ========
SFO1           600.1342009 MHz
NUC1                    1H
P0                    9.50 usec
P1                    9.50 usec
PLW1           30.00000000 W

====== GRADIENT CHANNEL =====
GPNAM[1]         SMSQ10.100
GPZ1                 10.00 %
P16                1000.00 usec

F1 - Acquisition parameters
TD                     256
SFO1              600.1342 MHz
FIDRES           75.120193 Hz
SW                  16.022 ppm
FnMODE                  QF

F2 - Processing parameters
SI                    1024
SF             600.1300339 MHz
WDW                   SINE
SSB                      0
LB                       0 Hz
GB                       0
PC                    1.00

F1 - Processing parameters
SI                    1024
MC2                     QF
SF             600.1300349 MHz
WDW                   SINE
SSB                      0
LB                       0 Hz
GB                       0
```

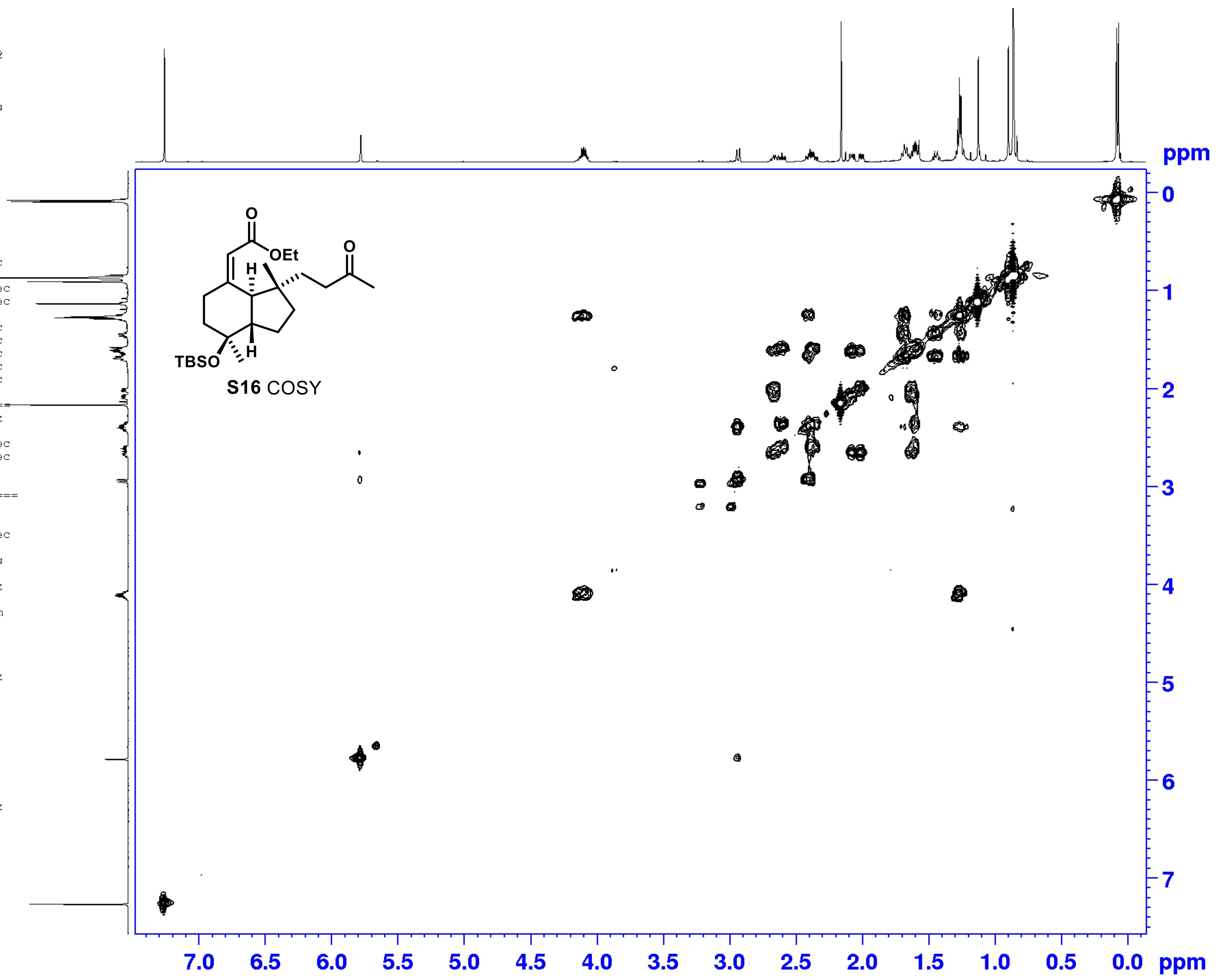

```
Current Data Parameters
NAME       JSCIV-142-f29-38-2
EXPNO                       4
PROCNO                      1

F2 - Acquisition Parameters
Date_                20220609
Time                    14.52
INSTRUM                 av600
PROBHD     5 mm CPBBO BB-
PULPROG      hsqcetgpsp.2
TD                       1024
SOLVENT                 CDCl3
NS                          2
DS                         16
SWH                  9615.385 Hz
FIDRES               9.390024 Hz
AQ                  0.0532480 sec
RG                       2050
DW                     52.000 usec
DE                      10.00 usec
TE                      297.9 K
CNST2             145.0000000
D0                 0.00000300 sec
D1                 1.10000002 sec
D4                 0.00172414 sec
D11                0.03000000 sec
D16                0.00020000 sec
IN0                0.00001380 sec
ZGOPTNS

======== CHANNEL f1 ========
SFO1              600.1342009 MHz
NUC1                       1H
P1                      10.00 usec
P2                      20.00 usec
P28                   1000.00 usec
PLW1              30.00000000 W

======== CHANNEL f2 ========
SFO2              150.9194083 MHz
NUC2                      13C
CPDPRG[2                 garp
P3                      10.10 usec
P14                    500.00 usec
P24                   2000.00 usec
PCPD2                   65.00 usec
PLW0                        0 W
PLW2              64.00000000 W
PLW12              1.47909999 W
SPNAM[3]  Crp60,0.5,20.1
SPOAL3                  0.500
SPOFFS3                     0 Hz
SPW3              10.00000000 W
SPNAM[7]       Crp60comp.4
SPOAL7                  0.500
SPOFFS7                     0 Hz
SPW7              10.00000000 W

====== GRADIENT CHANNEL =====
GPNAM[1]          SMSQ10.100
GPNAM[2]          SMSQ10.100
GPZ1                    80.00 %
GPZ2                    20.10 %
P16                   1000.00 usec

F1 - Acquisition parameters
TD                        256
SFO1                 150.9194 MHz
FIDRES             283.061584 Hz
SW                    240.074 ppm
FnMODE        Echo-Antiecho

F2 - Processing parameters
SI                       1024
SF                600.1300325 MHz
WDW                        EM
SSB                         0
LB                       5.00 Hz
GB                          0
PC                       1.00

F1 - Processing parameters
SI                       1024
MC2             echo-antiecho
SF                150.9028166 MHz
WDW                     QSINE
SSB                         3
LB                          0 Hz
GB                          0
```

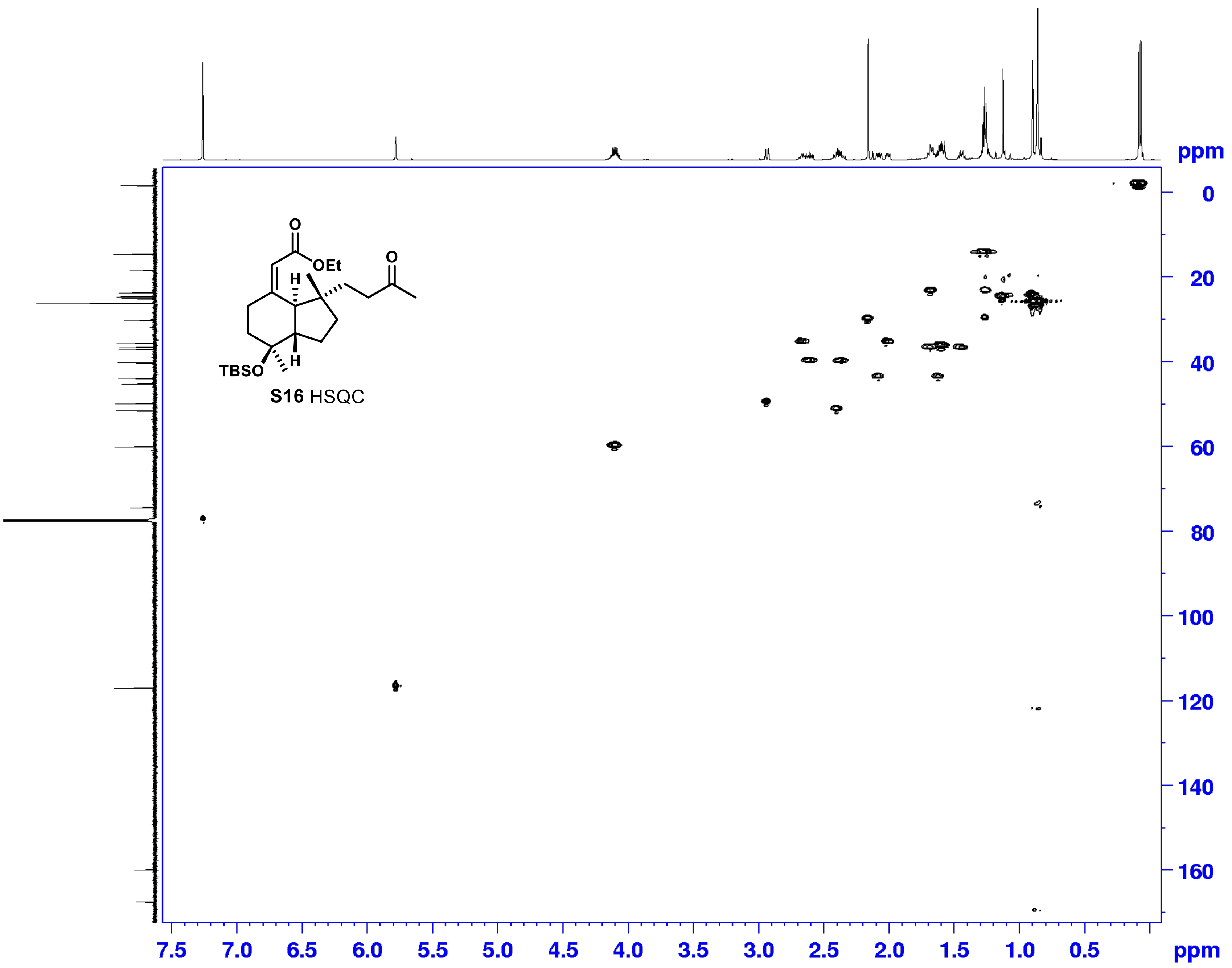

Current Data Parameters
NAME JSCIV-142-f29-38-2
EXPNO 6
PROCNO 1

F2 - Acquisition Parameters
Date_ 20220609
Time 14.12
INSTRUM av600
PROBHD 5 mm CPBBO BB-
PULPROG hmbcgplpndqf
TD 4096
SOLVENT CDCl3
NS 2
DS 16
SWH 9615.385 Hz
FIDRES 2.347506 Hz
AQ 0.2129920 sec
RG 2050
DW 52.000 usec
DE 10.00 usec
TE 298.0 K
CNST2 145.0000000
CNST13 10.0000000
D0 0.00000300 sec
D1 1.10000002 sec
D2 0.00344828 sec
D6 0.05000000 sec
D16 0.00020000 sec
IN0 0.00001380 sec

======== CHANNEL f1 ========
SFO1 600.1342009 MHz
NUC1 1H
P1 9.50 usec
P2 19.00 usec
PLW1 30.00000000 W

======== CHANNEL f2 ========
SFO2 150.9194083 MHz
NUC2 13C
P3 10.10 usec
PLW2 64.00000000 W

====== GRADIENT CHANNEL =====
GPNAM[1] SMSQ10.100
GPNAM[2] SMSQ10.100
GPNAM[3] SMSQ10.100
GPZ1 50.00 %
GPZ2 30.00 %
GPZ3 40.10 %
P16 1000.00 usec

F1 - Acquisition parameters
TD 256
SFO1 150.9194 MHz
FIDRES 283.061584 Hz
SW 240.074 ppm
FnMODE QF

F2 - Processing parameters
SI 2048
SF 600.1300335 MHz
WDW SINE
SSB 0
LB 0 Hz
GB 0
PC 1.00

F1 - Processing parameters
SI 1024
MC2 QF
SF 150.9028115 MHz
WDW SINE
SSB 0
LB 0 Hz
GB 0

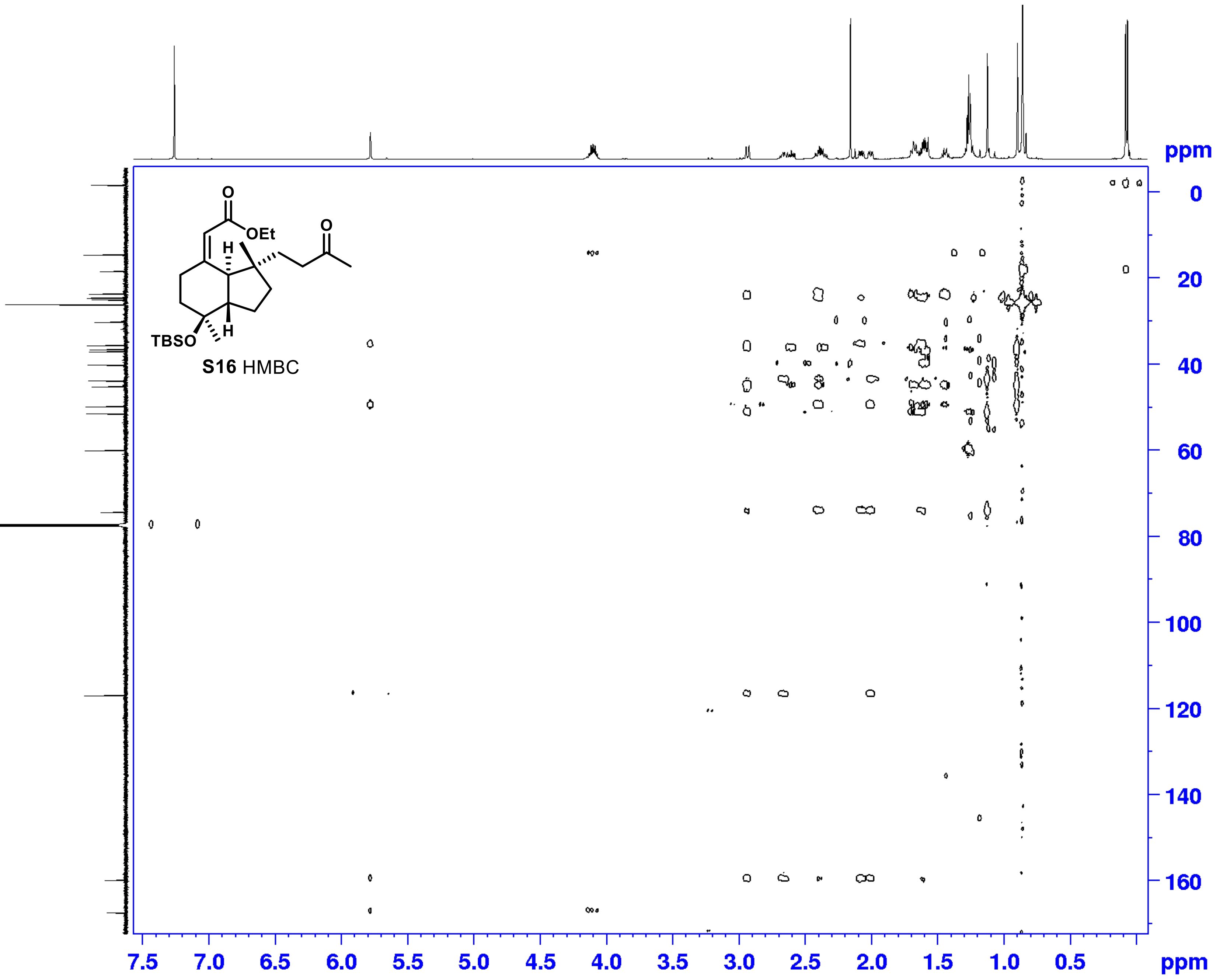

```
Current Data Parameters
NAME      JSCIV-142-f29-38-2
EXPNO                    7
PROCNO                   1

F2 - Acquisition Parameters
Date_           20220609
Time               14.24
INSTRUM            av600
PROBHD   5 mm CPBBO BB-
PULPROG        noesygpph
TD                  2048
SOLVENT            CDCl3
NS                     2
DS                    16
SWH             9615.385 Hz
FIDRES          4.695012 Hz
AQ             0.1064960 sec
RG                    57
DW                52.000 usec
DE                 16.68 usec
TE                 297.9 K
D0            0.00003990 sec
D1            2.00000000 sec
D8            1.00000000 sec
D16           0.00020000 sec
IN0           0.00010400 sec

======== CHANNEL f1 ========
SFO1         600.1342009 MHz
NUC1                  1H
P1                  9.50 usec
P2                 19.00 usec
PLW1         30.00000000 W

====== GRADIENT CHANNEL =====
GPNAM[1]      SMSQ10.100
GPZ1               40.00 %
P16              1000.00 usec

F1 - Acquisition parameters
TD                   256
SFO1            600.1342 MHz
FIDRES         75.120193 Hz
SW                16.022 ppm
FnMODE       States-TPPI

F2 - Processing parameters
SI                  1024
SF           600.1300355 MHz
WDW                QSINE
SSB                    2
LB                     0 Hz
GB                     0
PC                  1.00

F1 - Processing parameters
SI                  1024
MC2          States-TPPI
SF           600.1300355 MHz
WDW                QSINE
SSB                    2
LB                     0 Hz
GB                     0
```

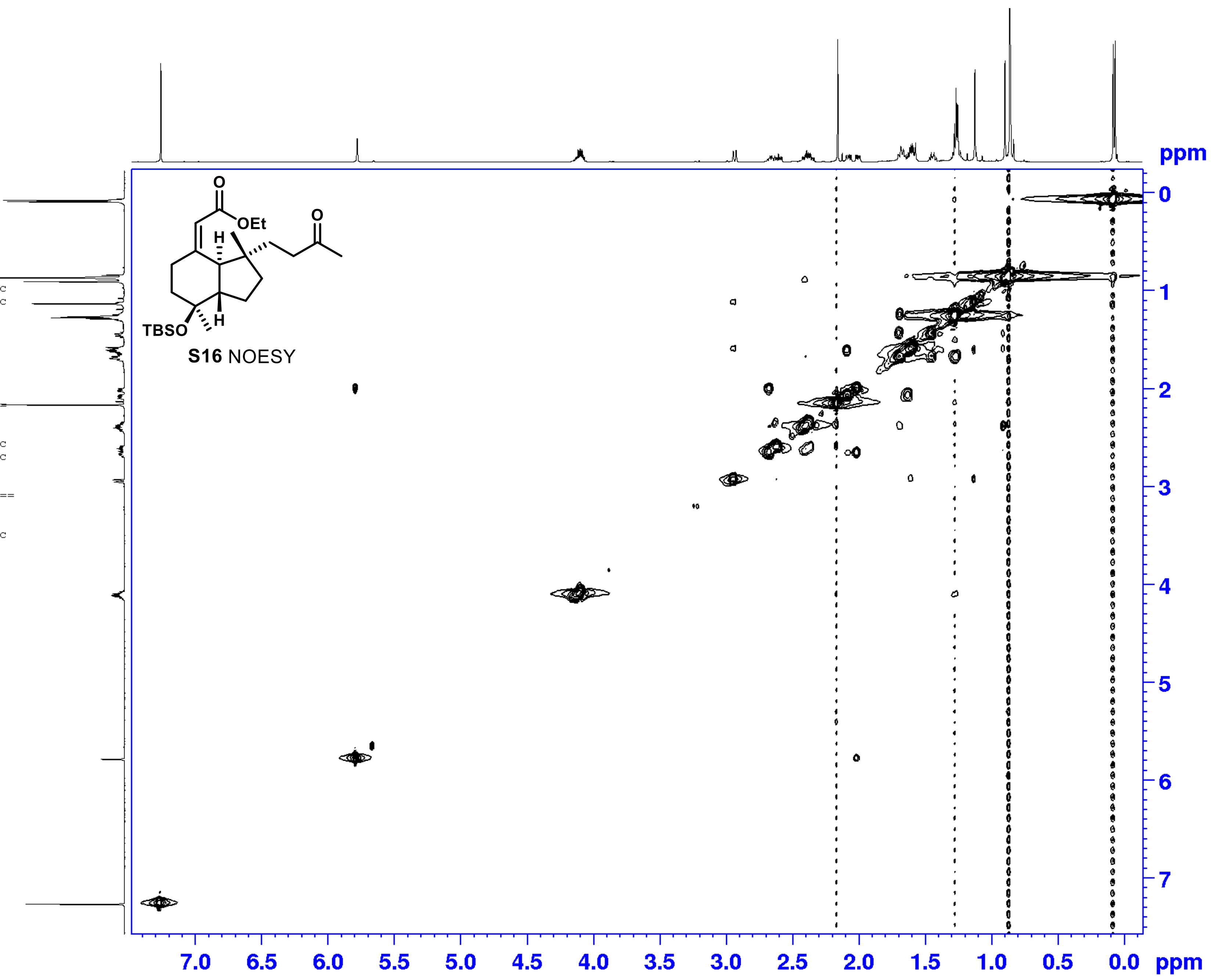

```
Current Data Parameters
NAME      JSCIV-142-f44-60
EXPNO                  1
PROCNO                 1

F2 - Acquisition Parameters
Date_           20210730
Time               20.24
INSTRUM            av600
PROBHD   5 mm CPBBO BB-
PULPROG             zg30
TD                 48074
SOLVENT            CDCl3
NS                    16
DS                     2
SWH             9615.385 Hz
FIDRES          0.200012 Hz
AQ             2.4998479 sec
RG                    10
DW                52.000 usec
DE                 14.23 usec
TE                 298.0 K
D1            4.00000000 sec
TD0                    1

======== CHANNEL f1 ========
SFO1         600.1342009 MHz
NUC1                  1H
P1                  9.50 usec
PLW1         30.00000000 W

F2 - Processing parameters
SI                 65536
SF           600.1300346 MHz
WDW                   EM
SSB                    0
LB                  0.30 Hz
GB                     0
PC                  1.00
```

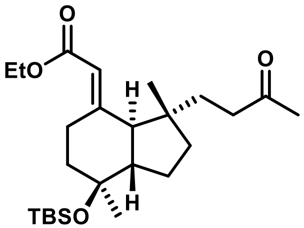

50

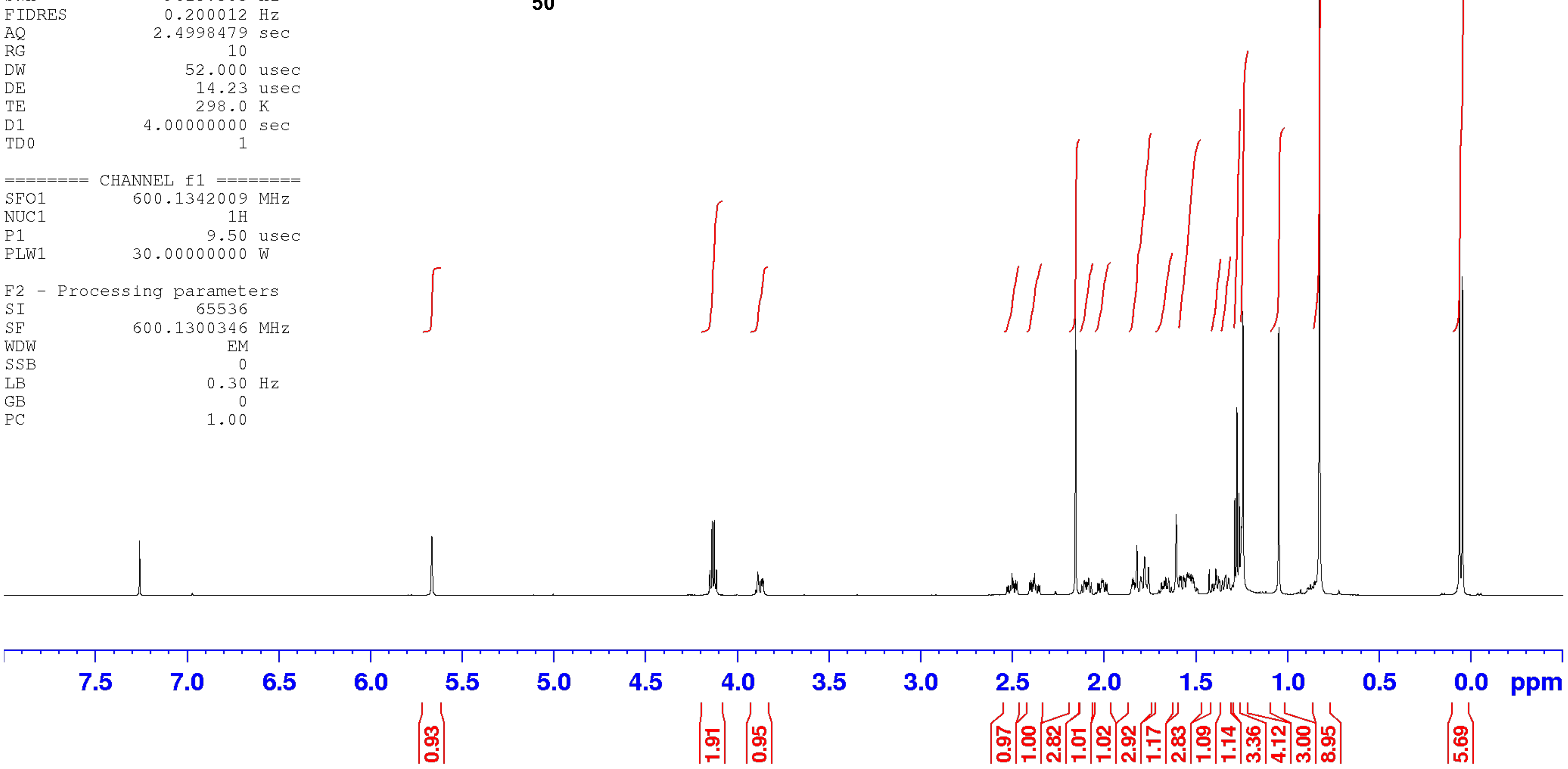

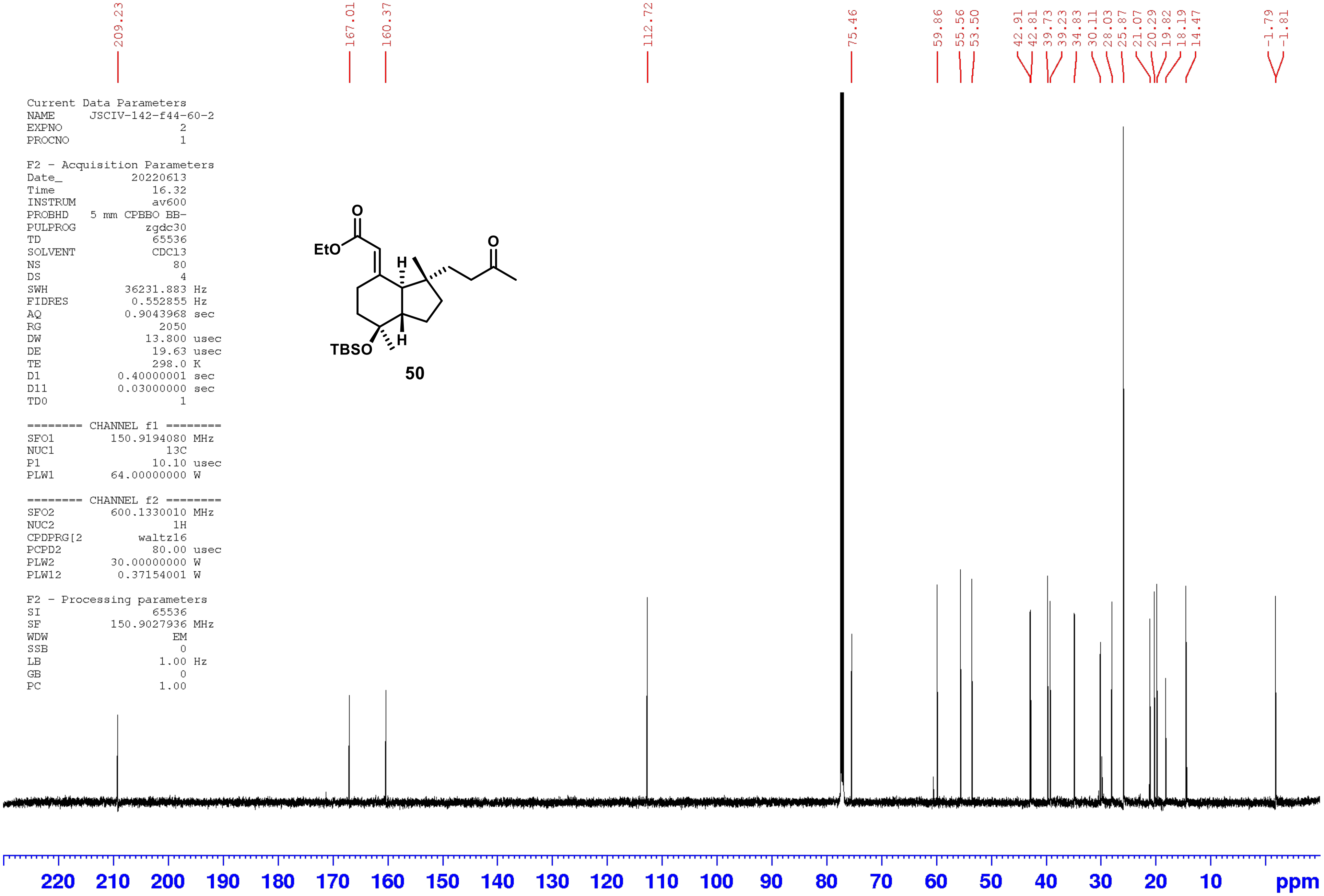
209.23
167.01
160.37
112.72
75.46
59.86
55.56
53.50
42.91
42.81
39.73
39.23
34.83
30.11
28.03
25.87
21.07
20.29
19.82
18.19
14.47
-1.79
-1.81
Current Data Parameters
NAME JSCIV-142-f44-60-2
EXPNO 2
PROCNO 1
F2 - Acquisition Parameters
Date_ 20220613
Time 16.32
INSTRUM av600
PROBHD 5 mm CPBBO BB-
PULPROG zgdc30
TD 65536
SOLVENT CDCl3
NS 80
DS 4
SWH 36231.883 Hz
FIDRES 0.552855 Hz
AQ 0.9043968 sec
RG 2050
DW 13.800 usec
DE 19.63 usec
TE 298.0 K
D1 0.40000001 sec
D11 0.03000000 sec
TD0 1
======== CHANNEL f1 ========
SFO1 150.9194080 MHz
NUC1 13C
P1 10.10 usec
PLW1 64.00000000 W
======== CHANNEL f2 ========
SFO2 600.1330010 MHz
NUC2 1H
CPDPRG[2 waltz16
PCPD2 80.00 usec
PLW2 30.00000000 W
PLW12 0.37154001 W
F2 - Processing parameters
SI 65536
SF 150.9027936 MHz
WDW EM
SSB 0
LB 1.00 Hz
GB 0
PC 1.00
O
EtO
H
O
TBSO
H
50
220 210 200 190 180 170 160 150 140 130 120 110 100 90 80 70 60 50 40 30 20 10 ppm

```
Current Data Parameters
NAME      JSCIV-142-f44-60-2
EXPNO                    5
PROCNO                   1

F2 - Acquisition Parameters
Date_          20220613
Time              16.49
INSTRUM           av600
PROBHD   5 mm CPBBO BB-
PULPROG        cosygpqf
TD                 2048
SOLVENT           CDCl3
NS                    1
DS                   16
SWH            9615.385 Hz
FIDRES         4.695012 Hz
AQ            0.1064960 sec
RG                  114
DW               52.000 usec
DE                10.00 usec
TE                298.0 K
D0           0.00000300 sec
D1           1.48689198 sec
D13          0.00000400 sec
D16          0.00020000 sec
IN0          0.00010400 sec

======== CHANNEL f1 ========
SFO1        600.1342009 MHz
NUC1                 1H
P0                 9.50 usec
P1                 9.50 usec
PLW1        30.00000000 W

====== GRADIENT CHANNEL =====
GPNAM[1]      SMSQ10.100
GPZ1              10.00 %
P16             1000.00 usec

F1 - Acquisition parameters
TD                  256
SFO1           600.1342 MHz
FIDRES        75.120193 Hz
SW               16.022 ppm
FnMODE               QF

F2 - Processing parameters
SI                 1024
SF          600.1300336 MHz
WDW                SINE
SSB                   0
LB                    0 Hz
GB                    0
PC                 1.00

F1 - Processing parameters
SI                 1024
MC2                  QF
SF          600.1300372 MHz
WDW                SINE
SSB                   0
LB                    0 Hz
GB                    0
```

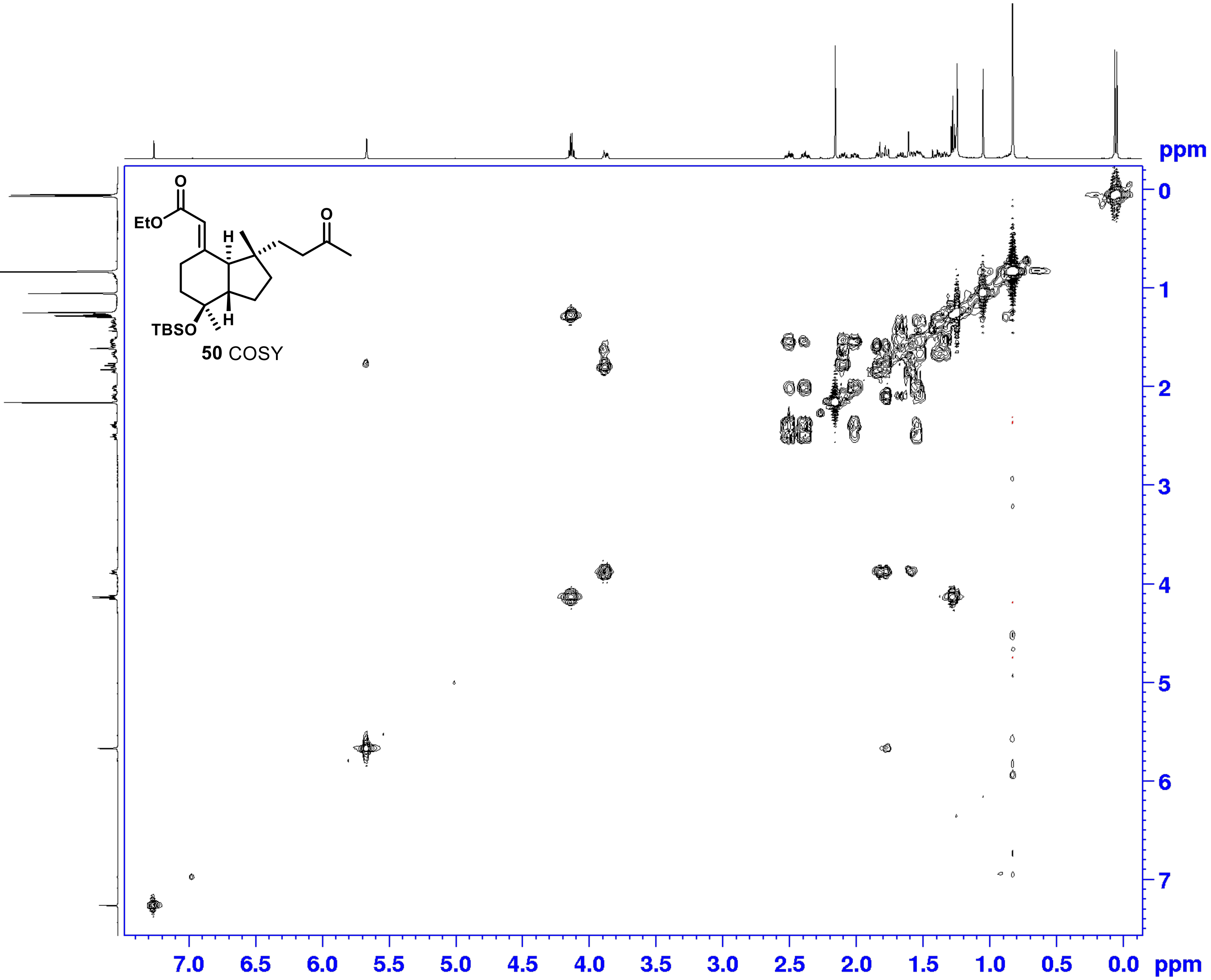

```
Current Data Parameters
NAME       JSCIV-142-f44-60-2
EXPNO                     4
PROCNO                    1

F2 - Acquisition Parameters
Date_              20220613
Time                  16.39
INSTRUM               av600
PROBHD    5 mm CPBBO BB-
PULPROG     hsqcetgpsp.2
TD                     1024
SOLVENT               CDCl3
NS                        2
DS                       16
SWH                9615.385 Hz
FIDRES             9.390024 Hz
AQ                0.0532480 sec
RG                     2050
DW                   52.000 usec
DE                    10.00 usec
TE                    297.9 K
CNST2           145.0000000
D0               0.00000300 sec
D1               1.10000002 sec
D4               0.00172414 sec
D11              0.03000000 sec
D16              0.00020000 sec
IN0              0.00001380 sec
ZGOPTNS

======== CHANNEL f1 ========
SFO1            600.1342009 MHz
NUC1                     1H
P1                    10.00 usec
P2                    20.00 usec
P28                 1000.00 usec
PLW1            30.00000000 W

======== CHANNEL f2 ========
SFO2            150.9194083 MHz
NUC2                    13C
CPDPRG[2               garp
P3                    10.10 usec
P14                  500.00 usec
P24                 2000.00 usec
PCPD2                 65.00 usec
PLW0                      0 W
PLW2            64.00000000 W
PLW12            1.47909999 W
SPNAM[3] Crp60,0.5,20.1
SPOAL3                0.500
SPOFFS3                   0 Hz
SPW3            10.00000000 W
SPNAM[7]     Crp60comp.4
SPOAL7                0.500
SPOFFS7                   0 Hz
SPW7            10.00000000 W

====== GRADIENT CHANNEL =====
GPNAM[1]       SMSQ10.100
GPNAM[2]       SMSQ10.100
GPZ1                  80.00 %
GPZ2                  20.10 %
P16                 1000.00 usec

F1 - Acquisition parameters
TD                      256
SFO1               150.9194 MHz
FIDRES           283.061584 Hz
SW                  240.074 ppm
FnMODE      Echo-Antiecho

F2 - Processing parameters
SI                     1024
SF              600.1300321 MHz
WDW                      EM
SSB                       0
LB                     5.00 Hz
GB                        0
PC                     1.00

F1 - Processing parameters
SI                     1024
MC2           echo-antiecho
SF              150.9028034 MHz
WDW                   QSINE
SSB                       3
LB                        0 Hz
GB                        0
```

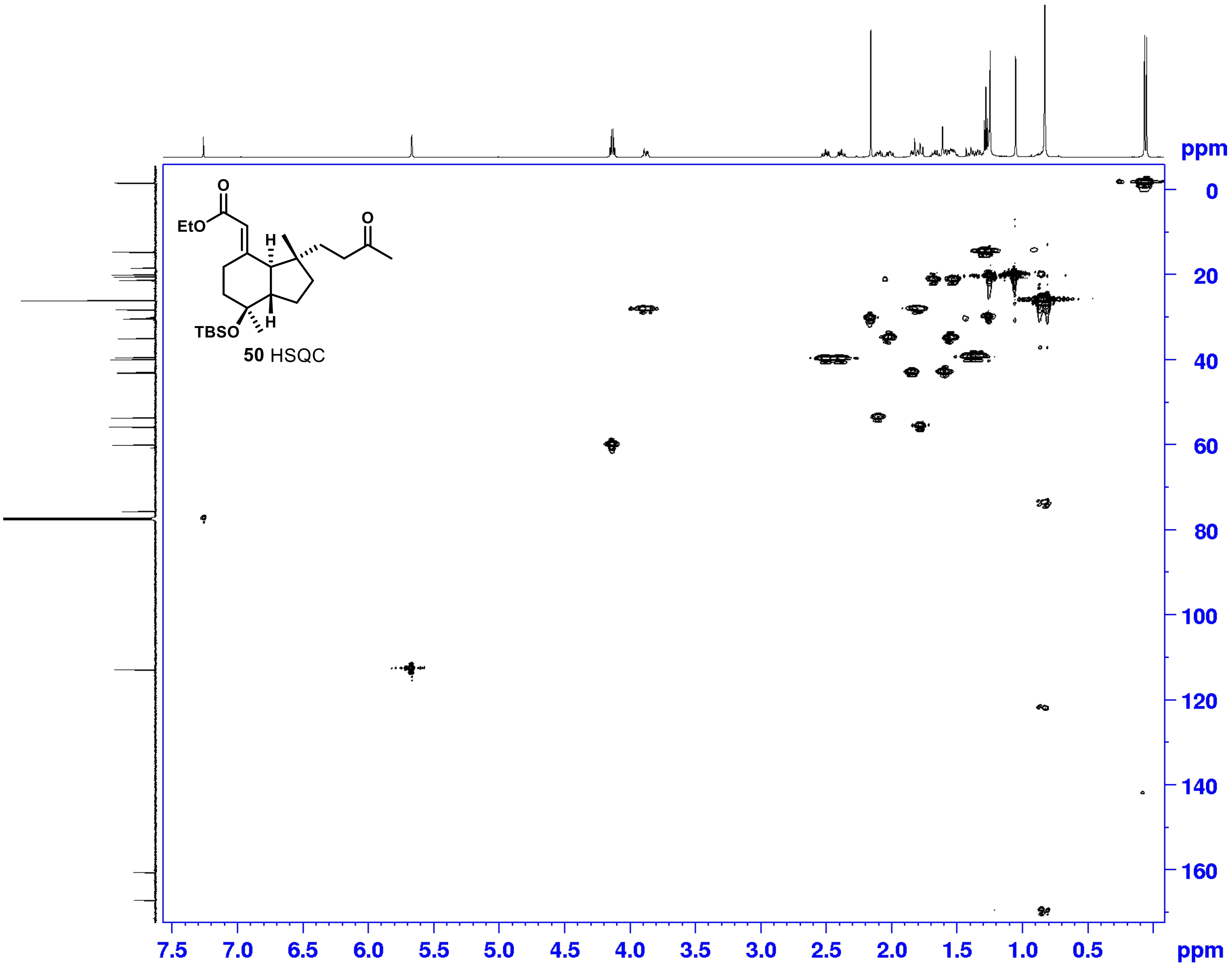

```
Current Data Parameters
NAME       JSCIV-142-f44-60-2
EXPNO                    6
PROCNO                   1

F2 - Acquisition Parameters
Date_            20220613
Time                16.57
INSTRUM             av600
PROBHD    5 mm CPBBO BB-
PULPROG      hmbcgplpndqf
TD                   4096
SOLVENT             CDCl3
NS                      2
DS                     16
SWH              9615.385 Hz
FIDRES           2.347506 Hz
AQ              0.2129920 sec
RG                   2050
DW                 52.000 usec
DE                  10.00 usec
TE                  297.9 K
CNST2         145.0000000
CNST13         10.0000000
D0             0.00000300 sec
D1             1.10000002 sec
D2             0.00344828 sec
D6             0.05000000 sec
D16            0.00020000 sec
IN0            0.00001380 sec

======== CHANNEL f1 ========
SFO1          600.1342009 MHz
NUC1                   1H
P1                   9.50 usec
P2                  19.00 usec
PLW1          30.00000000 W

======== CHANNEL f2 ========
SFO2          150.9194083 MHz
NUC2                  13C
P3                  10.10 usec
PLW2          64.00000000 W

====== GRADIENT CHANNEL =====
GPNAM[1]       SMSQ10.100
GPNAM[2]       SMSQ10.100
GPNAM[3]       SMSQ10.100
GPZ1                50.00 %
GPZ2                30.00 %
GPZ3                40.10 %
P16               1000.00 usec

F1 - Acquisition parameters
TD                    256
SFO1             150.9194 MHz
FIDRES         283.061584 Hz
SW                240.074 ppm
FnMODE                 QF

F2 - Processing parameters
SI                   2048
SF            600.1300327 MHz
WDW                  SINE
SSB                     0
LB                      0 Hz
GB                      0
PC                   1.00

F1 - Processing parameters
SI                   1024
MC2                    QF
SF            150.9028003 MHz
WDW                  SINE
SSB                     0
LB                      0 Hz
GB                      0
```

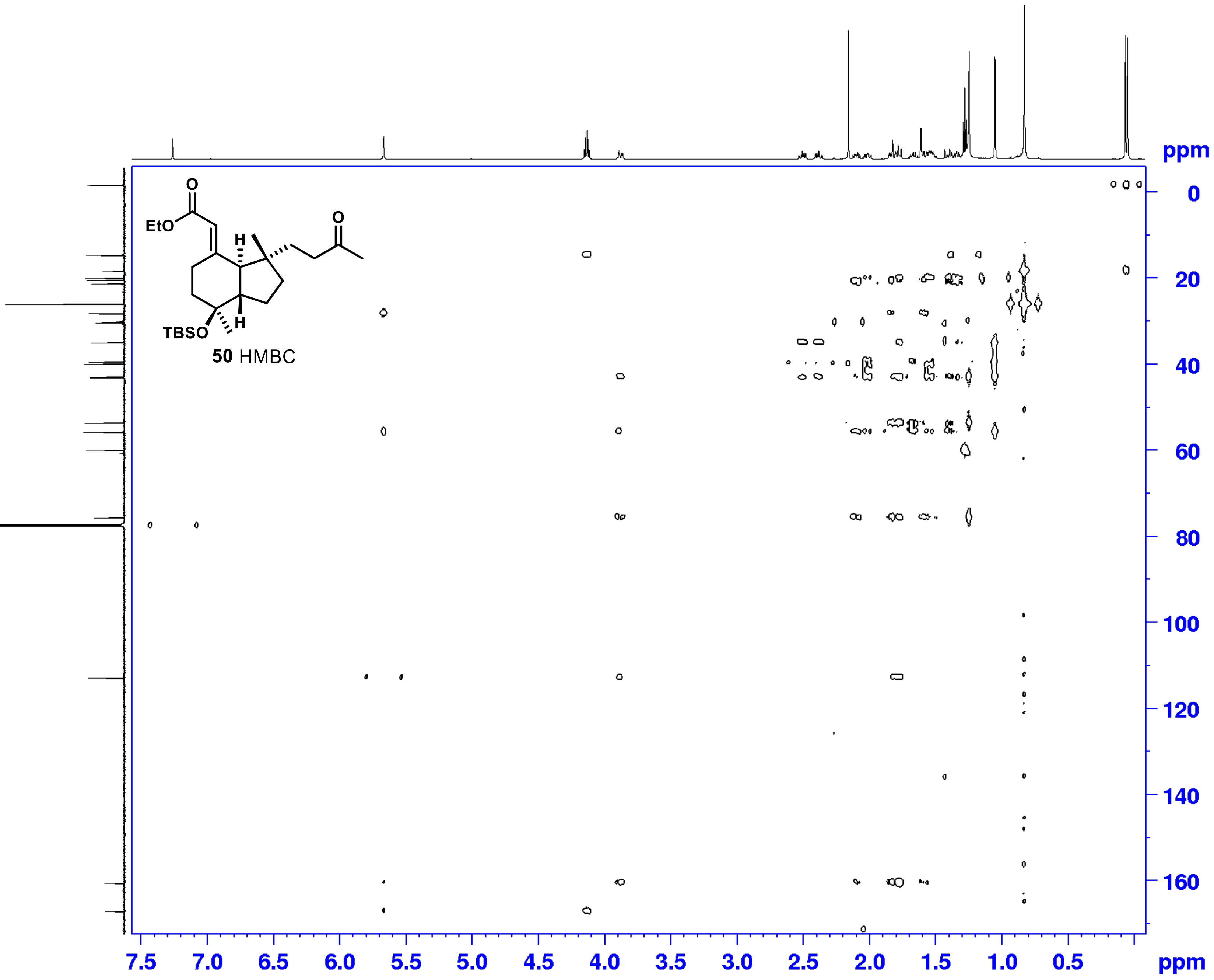

```
Current Data Parameters
NAME      JSCIV-142-f44-60-2
EXPNO                    7
PROCNO                   1

F2 - Acquisition Parameters
Date_          20220613
Time              17.10
INSTRUM           av600
PROBHD    5 mm CPBBO BB-
PULPROG      noesygpph
TD                2048
SOLVENT          CDCl3
NS                   2
DS                  16
SWH           9615.385 Hz
FIDRES        4.695012 Hz
AQ           0.1064960 sec
RG                  57
DW              52.000 usec
DE               16.68 usec
TE               297.9 K
D0          0.00003990 sec
D1          2.00000000 sec
D8          1.00000000 sec
D16         0.00020000 sec
IN0         0.00010400 sec

======== CHANNEL f1 ========
SFO1       600.1342009 MHz
NUC1                1H
P1                9.50 usec
P2               19.00 usec
PLW1       30.00000000 W

====== GRADIENT CHANNEL =====
GPNAM[1]      SMSQ10.100
GPZ1             40.00 %
P16            1000.00 usec

F1 - Acquisition parameters
TD                 256
SFO1          600.1342 MHz
FIDRES       75.120193 Hz
SW              16.022 ppm
FnMODE     States-TPPI

F2 - Processing parameters
SI                1024
SF         600.1300333 MHz
WDW              QSINE
SSB                  2
LB                   0 Hz
GB                   0
PC                1.00

F1 - Processing parameters
SI                1024
MC2        States-TPPI
SF         600.1300314 MHz
WDW              QSINE
SSB                  2
LB                   0 Hz
GB                   0
```

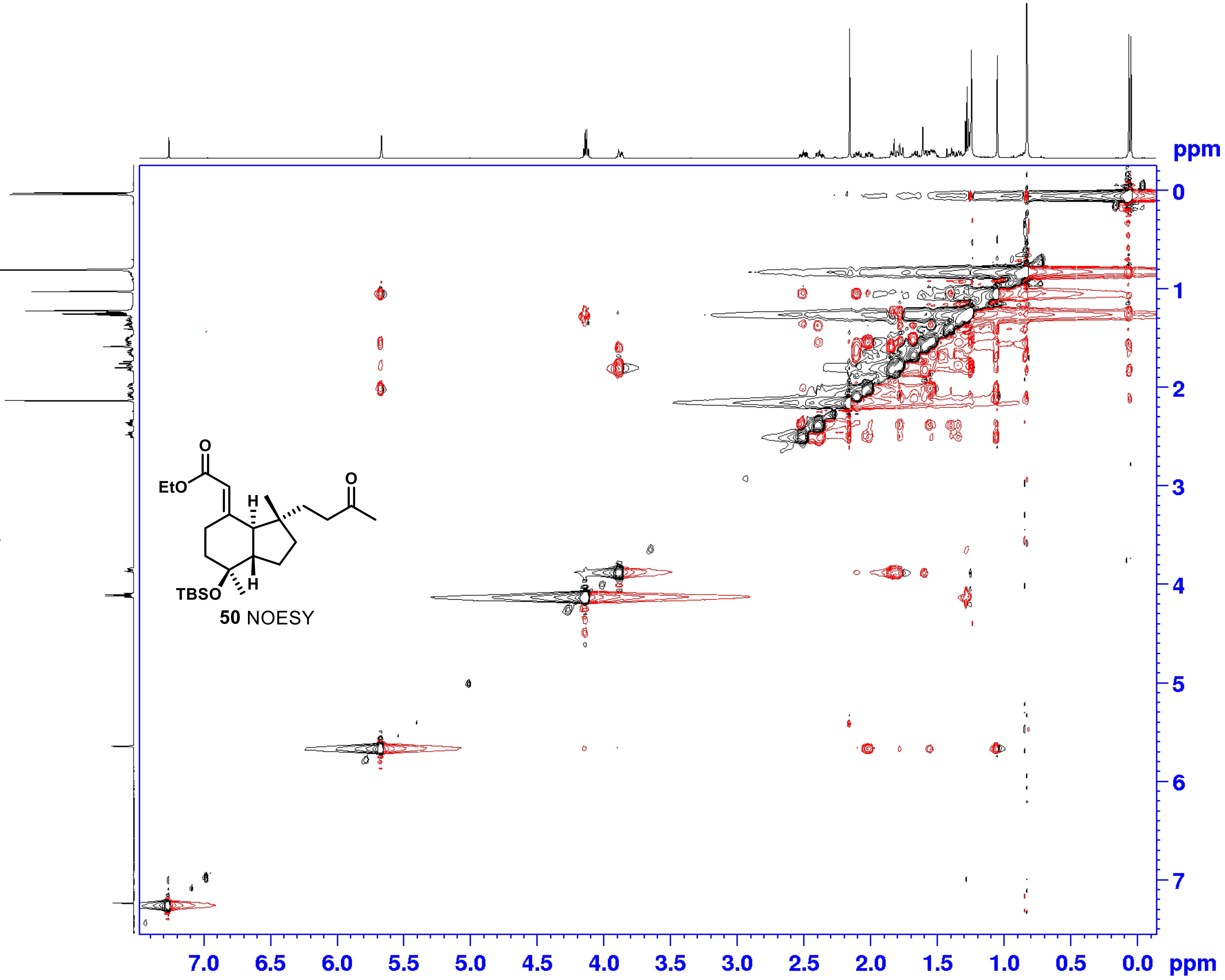

```
Current Data Parameters
NAME          JC 3-269 col
EXPNO                    1
PROCNO                   1

F2 - Acquisition Parameters
Date_             20210228
Time                 19.35
INSTRUM              av600
PROBHD    5 mm CPBBO BB-
PULPROG               zg30
TD                   98074
SOLVENT              CDCl3
NS                       8
DS                       2
SWH               9615.385 Hz
FIDRES            0.098042 Hz
AQ               5.0998478 sec
RG                       8
DW                  52.000 usec
DE                   13.70 usec
TE                   298.1 K
D1              0.10000000 sec
TD0                      1

======== CHANNEL f1 ========
SFO1           600.1342009 MHz
NUC1                    1H
P1                   12.00 usec
PLW1           28.00000000 W

F2 - Processing parameters
SI                   65536
SF             600.1300350 MHz
WDW                     EM
SSB      0
LB                    0.30 Hz
GB       0
PC                    1.00
```

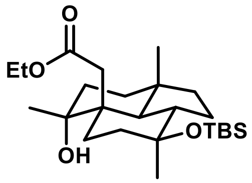

51

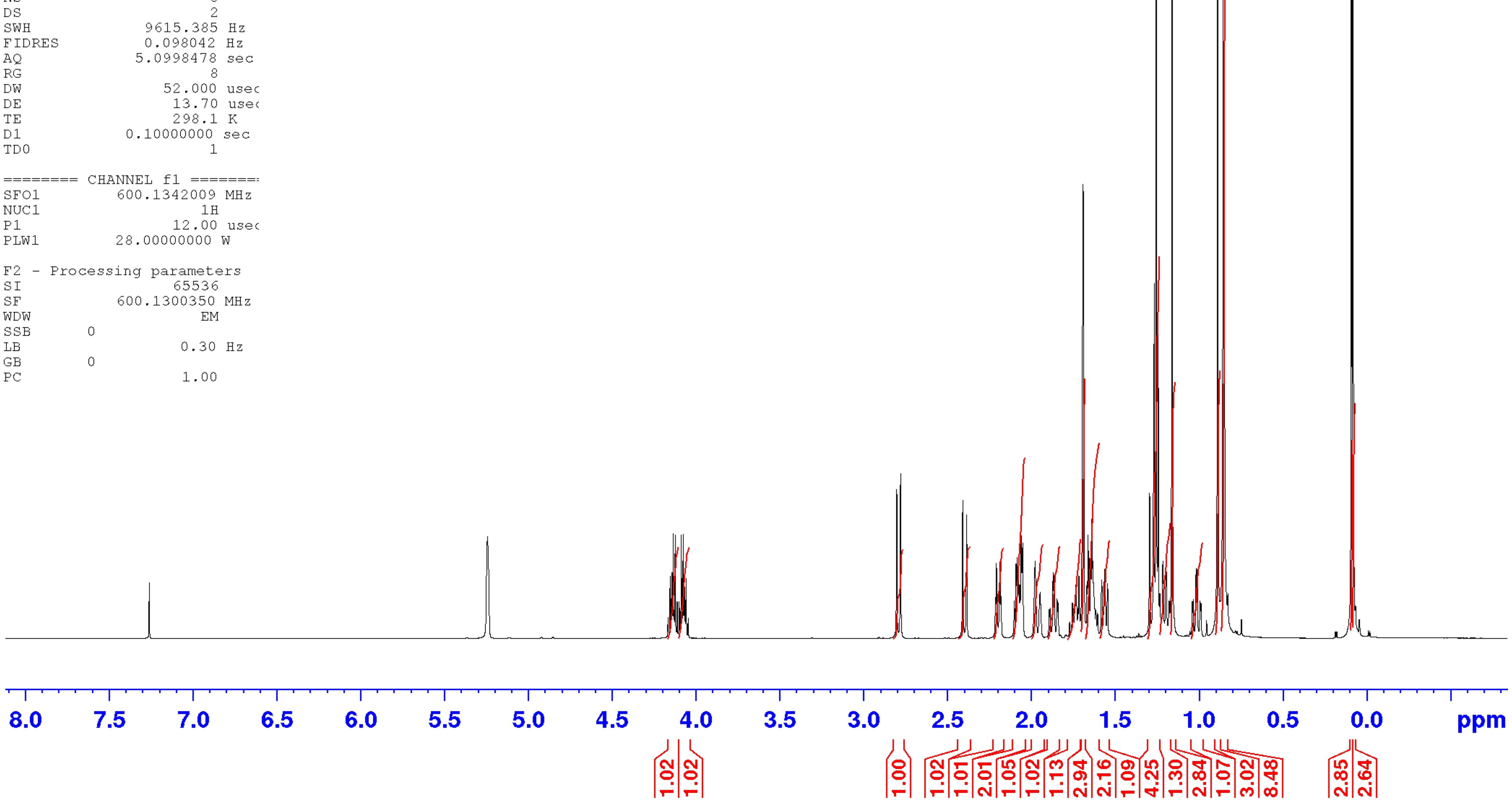

Current Data Parameters
NAME JC 3-269 col
EXPNO 2
PROCNO 1

F2 - Acquisition Parameters
Date_ 20210228
Time 19.40
INSTRUM av600
PROBHD 5 mm CPBBO BB-
PULPROG zgdc30
TD 65536
SOLVENT CDCl3
NS 191
DS 4
SWH 36231.883 Hz
FIDRES 0.552855 Hz
AQ 0.9043968 sec
RG 2050
DW 13.800 usec
DE 19.65 usec
TE 298.1 K
D1 0.40000001 sec
D11 0.03000000 sec
TD0 1

======== CHANNEL f1 ========
SFO1 150.9194075 MHz
NUC1 13C
P1 10.00 usec
PLW1 65.00000000 W

======== CHANNEL f2 ========
SFO2 600.1330010 MHz
NUC2 1H
CPDPRG[2 waltz16
PCPD2 80.00 usec
PLW2 28.00000000 W
PLW12 0.63000000 W

F2 - Processing parameters
SI 65536
SF 150.9027940 MHz
WDW EM
SSB 0
LB 1.00 Hz
GB 0
PC 1.00

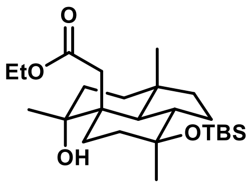

51

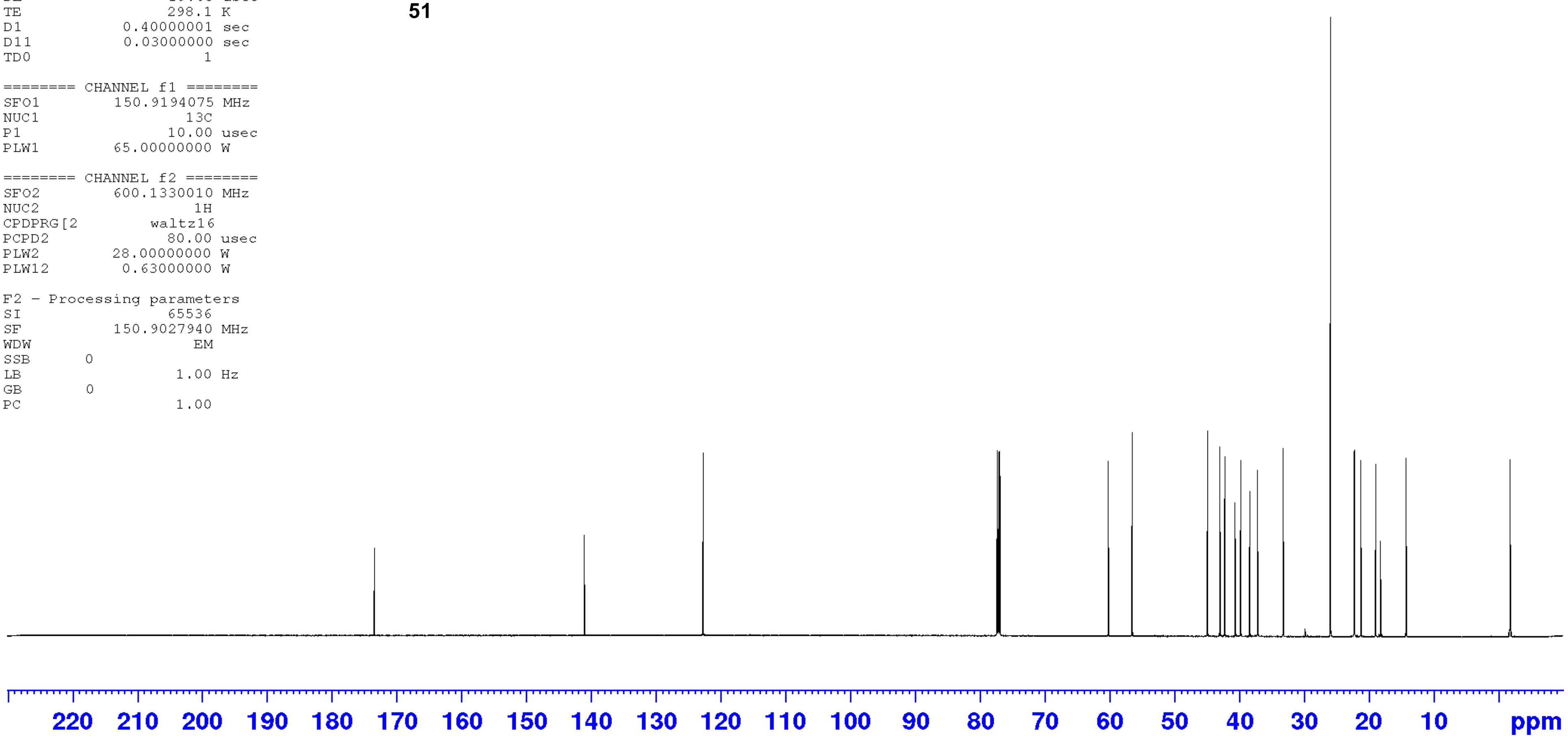

```
Current Data Parameters
NAME          JC 3-269 col
EXPNO                    1
PROCNO                   1

F2 - Acquisition Parameters
Date_             20210228
Time                 19.35
INSTRUM              av600
PROBHD    5 mm CPBBO BB-
PULPROG               zg30
TD                   98074
SOLVENT              CDCl3
NS                       8
DS                       2
SWH               9615.385 Hz
FIDRES            0.098042 Hz
AQ               5.0998478 sec
RG                       8
DW                  52.000 usec
DE                   13.70 usec
TE                   298.1 K
D1              0.10000000 sec
TD0                      1

======== CHANNEL f1 ========
SFO1         600.1342009 MHz
NUC1                    1H
P1                   12.00 usec
PLW1           28.00000000 W

F2 - Processing parameters
SI                   65536
SF           600.1300350 MHz
WDW                     EM
SSB      0
LB                    0.30 Hz
GB       0
PC                    1.00
```

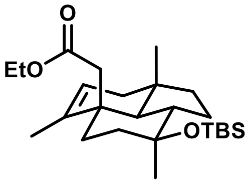

52

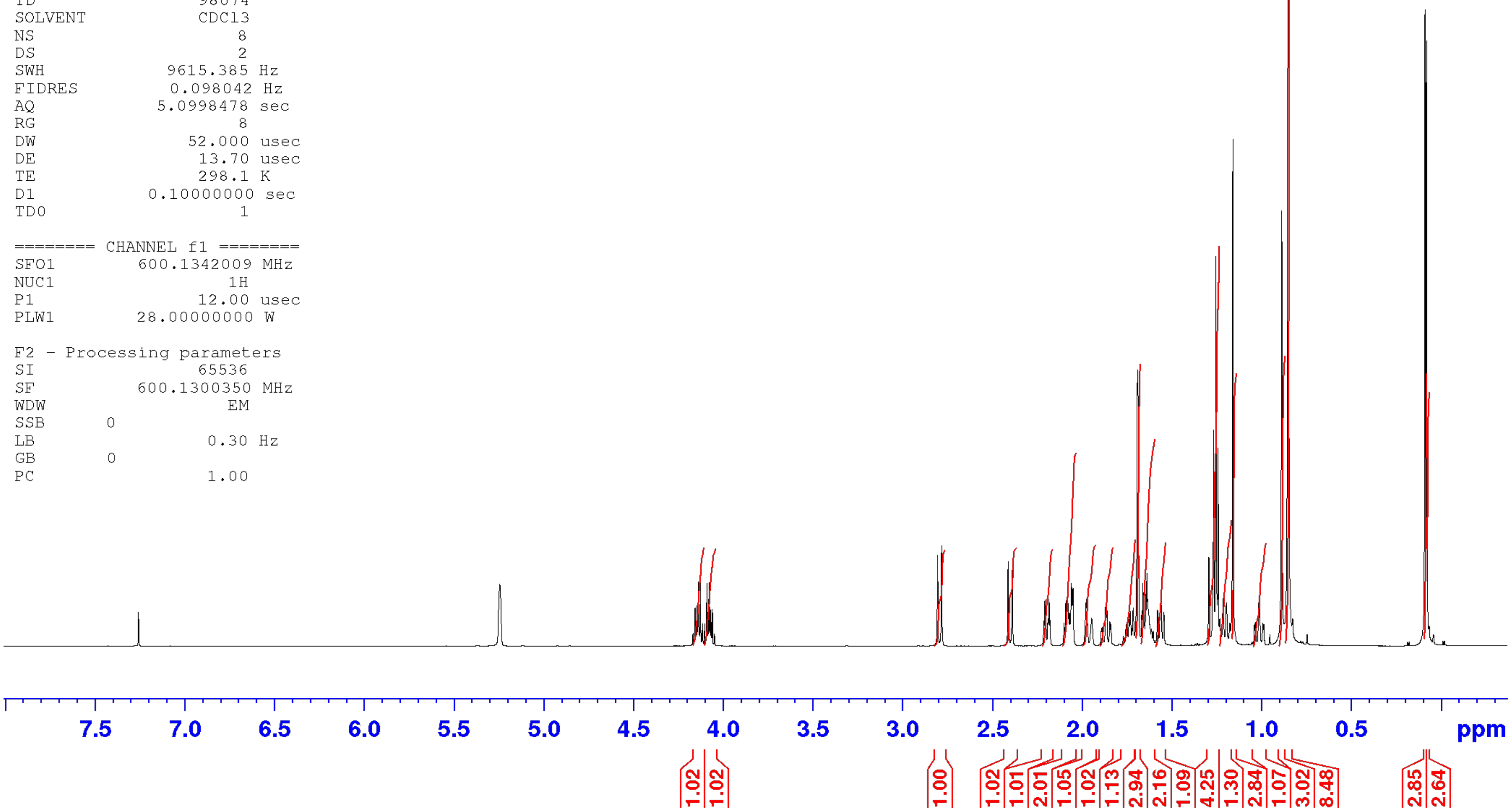

```
Current Data Parameters
NAME        JC 5-001 col
EXPNO                  2
PROCNO                 1

F2 - Acquisition Parameters
Date_           20210424
Time               19.43
INSTRUM            av600
PROBHD    5 mm CPBBO BB-
PULPROG          zgdc30
TD                 65536
SOLVENT            CDCl3
NS                   164
DS                     4
SWH            36231.883 Hz
FIDRES          0.552855 Hz
AQ             0.9043968 sec
RG                  2050
DW                13.800 usec
DE                 19.65 usec
TE                 298.1 K
D1            0.40000001 sec
D11           0.03000000 sec
TD0                    1

======== CHANNEL f1 ========
SFO1         150.9194075 MHz
NUC1                 13C
P1                 10.00 usec
PLW1         65.00000000 W

======== CHANNEL f2 ========
SFO2         600.1330010 MHz
NUC2                  1H
CPDPRG[2          waltz16
PCPD2              80.00 usec
PLW2         28.00000000 W
PLW12         0.63000000 W

F2 - Processing parameters
SI                 65536
SF           150.9027928 MHz
WDW                   EM
SSB      0
LB                  1.00 Hz
GB       0
PC                  1.00
```

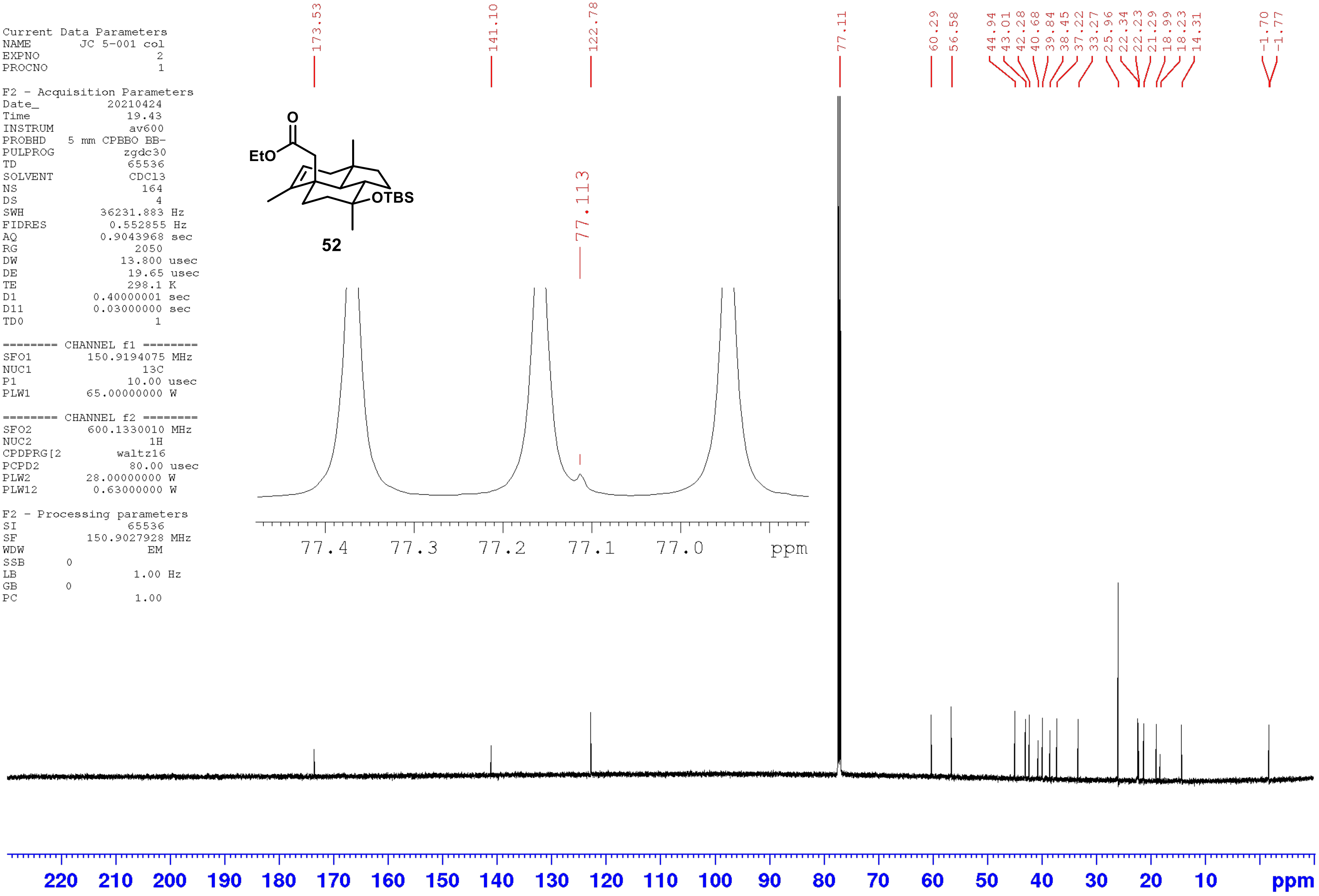

```
Current Data Parameters
NAME      JC 3-185 bot socl2
EXPNO                    1
PROCNO                   1

F2 - Acquisition Parameters
Date_             20200824
Time                 23.44
INSTRUM              av600
PROBHD   5 mm CPBBO BB-
PULPROG               zg30
TD                   98074
SOLVENT              CDCl3
NS                       8
DS                       2
SWH              9615.385 Hz
FIDRES           0.098042 Hz
AQ              5.0998478 sec
RG                      10
DW                  52.000 usec
DE                   13.70 usec
TE                   298.0 K
D1             0.10000000 sec
TD0                      1

======== CHANNEL f1 ========
SFO1         600.1342009 MHz
NUC1                    1H
P1                   12.00 usec
PLW1         28.00000000 W

F2 - Processing parameters
SI                   65536
SF           600.1300341 MHz
WDW                     EM
SSB      0
LB                    0.30 Hz
GB       0
PC                    1.00
```

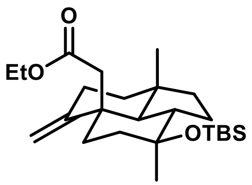

S17

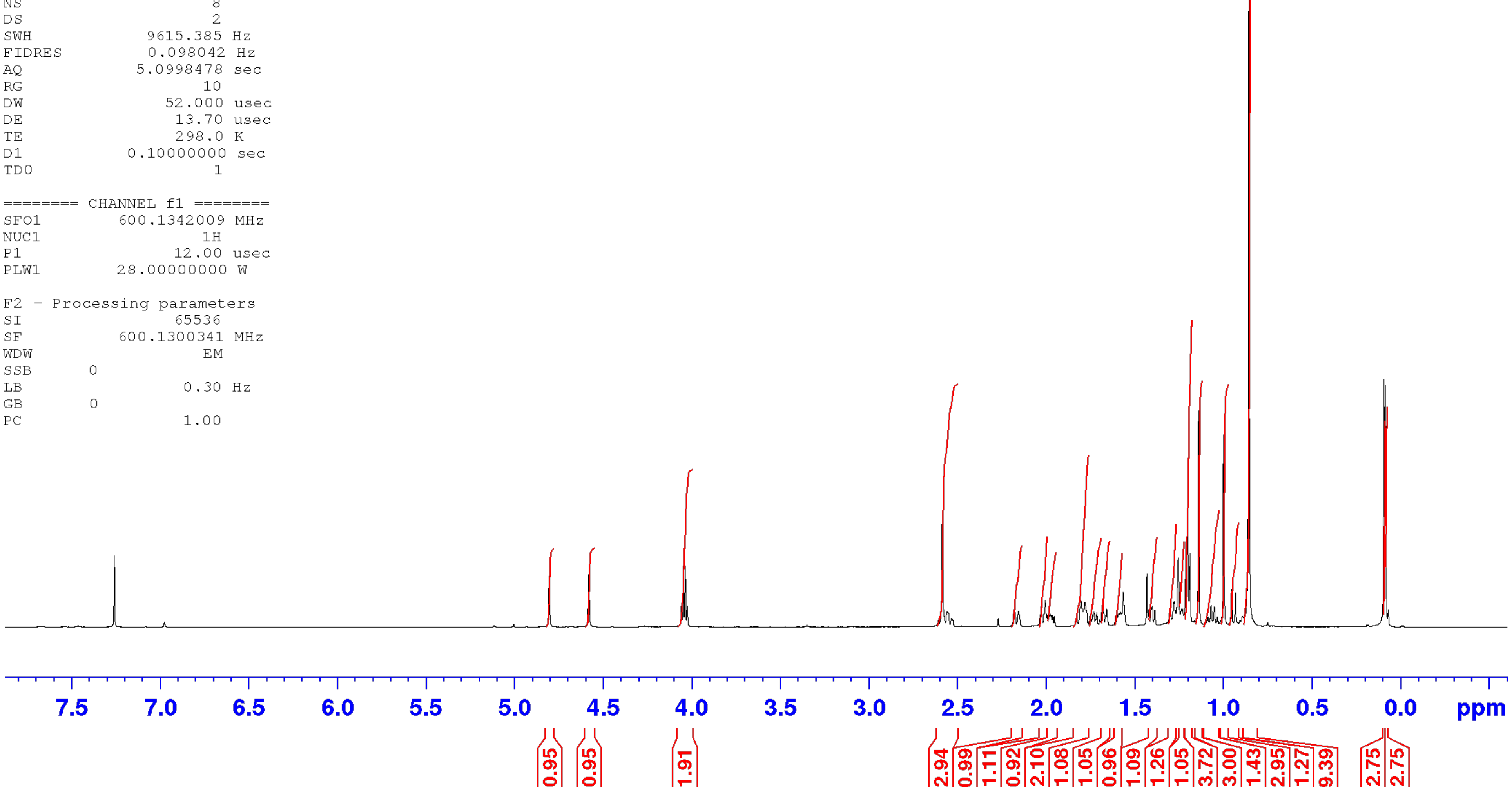

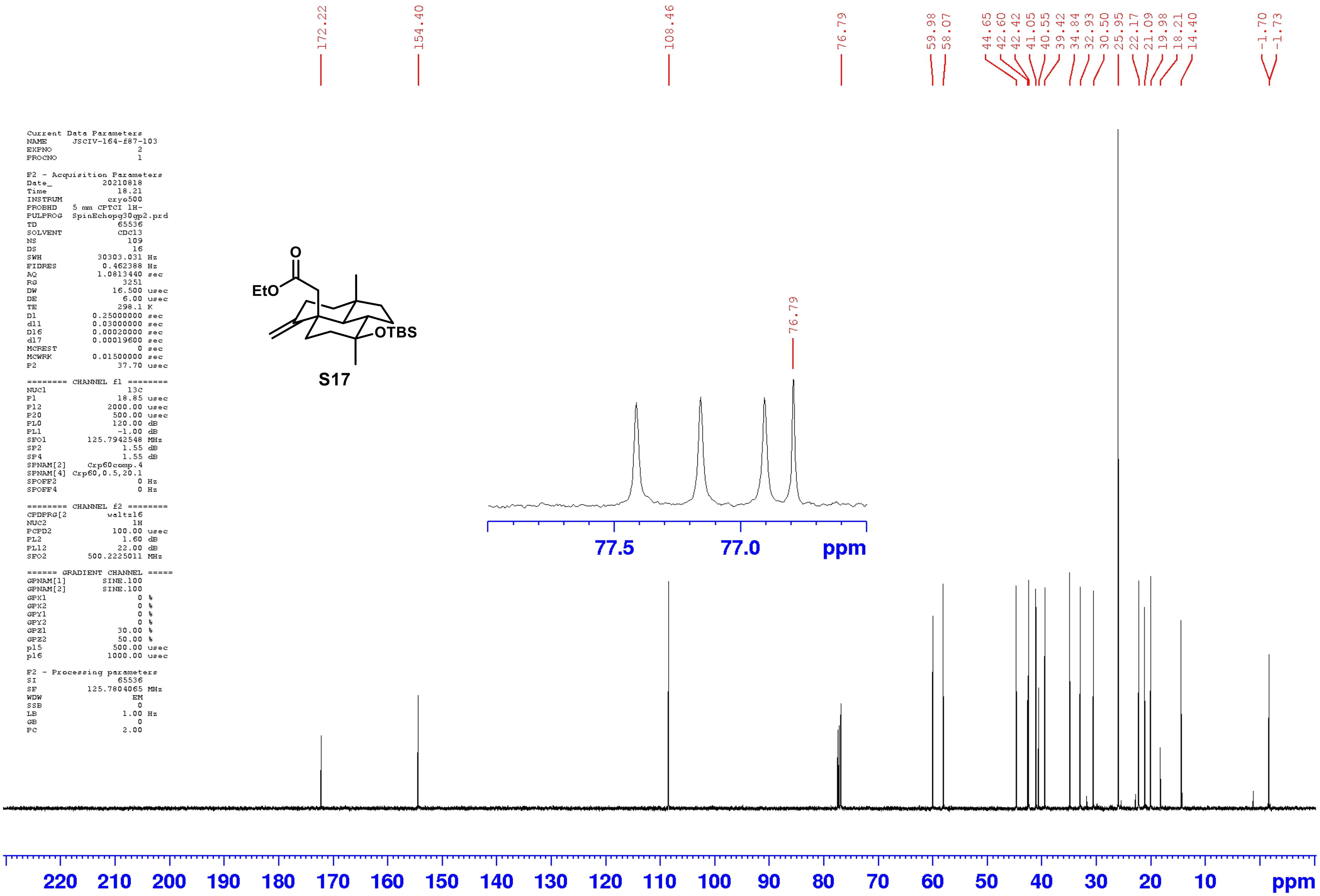
Current Data Parameters
NAME JSCIV-164-f87-103
EXPNO 2
PROCNO 1
F2 - Acquisition Parameters
Date_ 20210818
Time 18.21
INSTRUM cryo500
PROBHD 5 mm CPTCI 1H-
PULPROG SpinEchopg30gp2.prd
TD 65536
SOLVENT CDCl3
NS 109
DS 16
SWH 30303.031 Hz
FIDRES 0.462388 Hz
AQ 1.0813440 sec
RG 3251
DW 16.500 usec
DE 6.00 usec
TE 298.1 K
D1 0.25000000 sec
d11 0.03000000 sec
D16 0.00020000 sec
d17 0.00019600 sec
MCREST 0 sec
MCWRK 0.01500000 sec
P2 37.70 usec
======== CHANNEL f1 ========
NUC1 13C
P1 18.85 usec
P12 2000.00 usec
P20 500.00 usec
PL0 120.00 dB
PL1 -1.00 dB
SFO1 125.7942548 MHz
SP2 1.55 dB
SP4 1.55 dB
SPNAM[2] Crp60comp.4
SPNAM[4] Crp60,0.5,20.1
SPOFF2 0 Hz
SPOFF4 0 Hz
======== CHANNEL f2 ========
CPDPRG[2 waltz16
NUC2 1H
PCPD2 100.00 usec
PL2 1.60 dB
PL12 22.00 dB
SFO2 500.2225011 MHz
====== GRADIENT CHANNEL =====
GPNAM[1] SINE.100
GPNAM[2] SINE.100
GPX1 0 %
GPX2 0 %
GPY1 0 %
GPY2 0 %
GPZ1 30.00 %
GPZ2 50.00 %
p15 500.00 usec
p16 1000.00 usec
F2 - Processing parameters
SI 65536
SF 125.7804065 MHz
WDW EM
SSB 0
LB 1.00 Hz
GB 0
PC 2.00
172.22
154.40
108.46
76.79
59.98
58.07
44.65
42.60
42.42
41.05
40.55
39.42
34.84
32.93
30.50
25.95
22.17
21.09
19.98
18.21
14.40
-1.70
-1.73
76.79
77.5
77.0
ppm
220 210 200 190 180 170 160 150 140 130 120 110 100 90 80 70 60 50 40 30 20 10
ppm
O
EtO
OTBS

S17

```
Current Data Parameters
NAME      JC 3-270 top dibal COL
EXPNO                    1
PROCNO                   1

F2 - Acquisition Parameters
Date_            20210301
Time                 3.38
INSTRUM             av600
PROBHD    5 mm CPBBO BB-
PULPROG              zg30
TD                  98074
SOLVENT             CDCl3
NS                      8
DS                      2
SWH              9615.385 Hz
FIDRES           0.098042 Hz
AQ              5.0998478 sec
RG                      8
DW                 52.000 usec
DE                  13.70 usec
TE                  298.1 K
D1             0.10000000 sec
TD0                     1

======== CHANNEL f1 ========
SFO1          600.1342009 MHz
NUC1                   1H
P1                  12.00 usec
PLW1          28.00000000 W

F2 - Processing parameters
SI                  65536
SF            600.1300353 MHz
WDW                    EM
SSB       0
LB                   0.30 Hz
GB        0
PC                   1.00
```

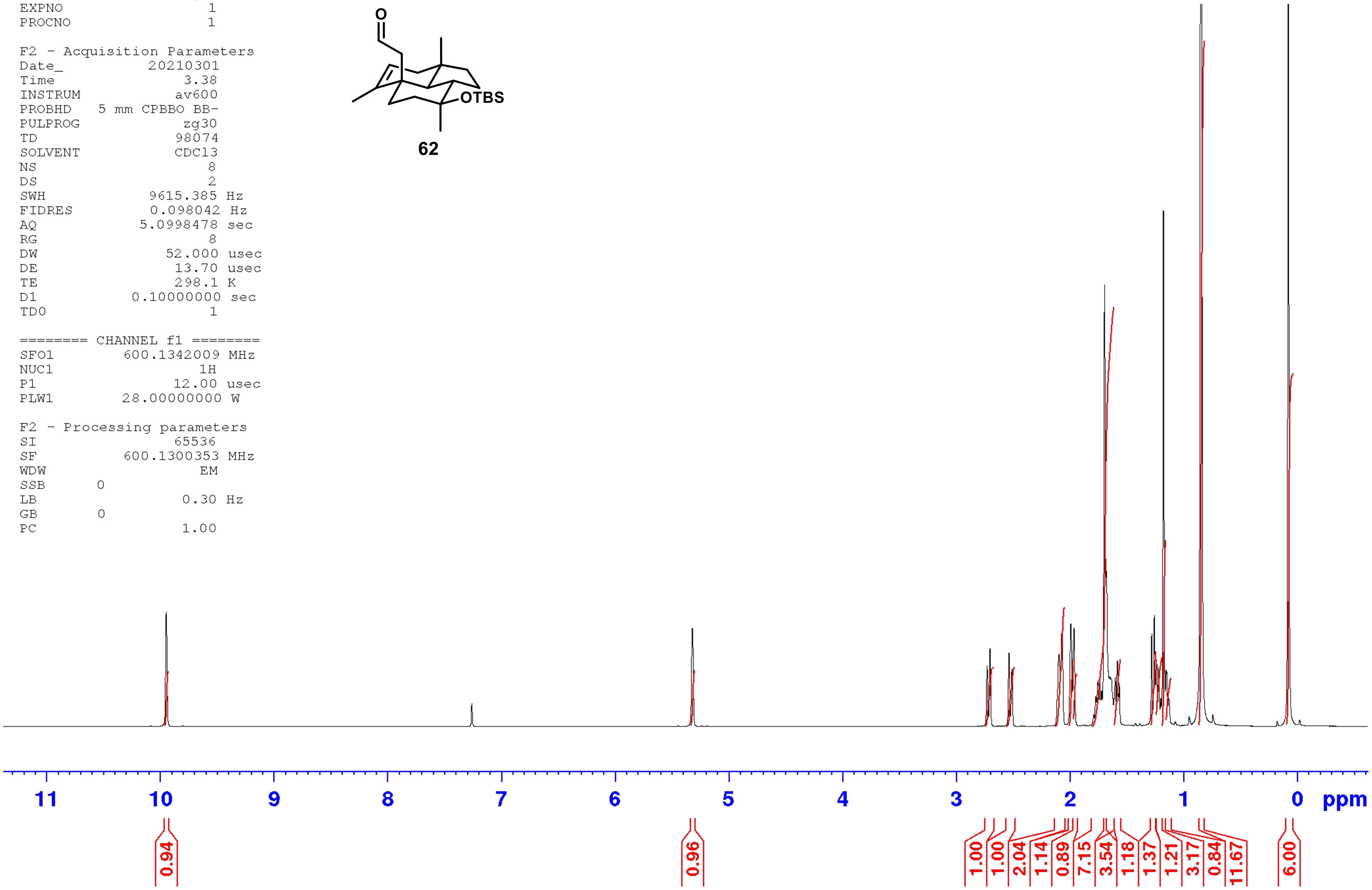

```
Current Data Parameters
NAME      JSCIV-162-f2-7
EXPNO                    2
PROCNO                   1

F2 - Acquisition Parameters
Date_            20210816
Time                15.42
INSTRUM             av600
PROBHD   5 mm CPBBO BB-
PULPROG           zgdc30
TD                 65536
SOLVENT            CDCl3
NS                   143
DS                     4
SWH            36231.883 Hz
FIDRES          0.552855 Hz
AQ             0.9043968 sec
RG                  2050
DW                13.800 usec
DE                 19.63 usec
TE                 298.0 K
D1            0.40000001 sec
D11           0.03000000 sec
TD0                    1

======== CHANNEL f1 ========
SFO1         150.9194080 MHz
NUC1                 13C
P1                 10.10 usec
PLW1         64.00000000 W

======== CHANNEL f2 ========
SFO2         600.1330010 MHz
NUC2                  1H
CPDPRG[2           waltz16
PCPD2              80.00 usec
PLW2         30.00000000 W
PLW12         0.37154001 W

F2 - Processing parameters
SI                 65536
SF           150.9027937 MHz
WDW                   EM
SSB                    0
LB                  1.00 Hz
GB                     0
PC                  1.00
```

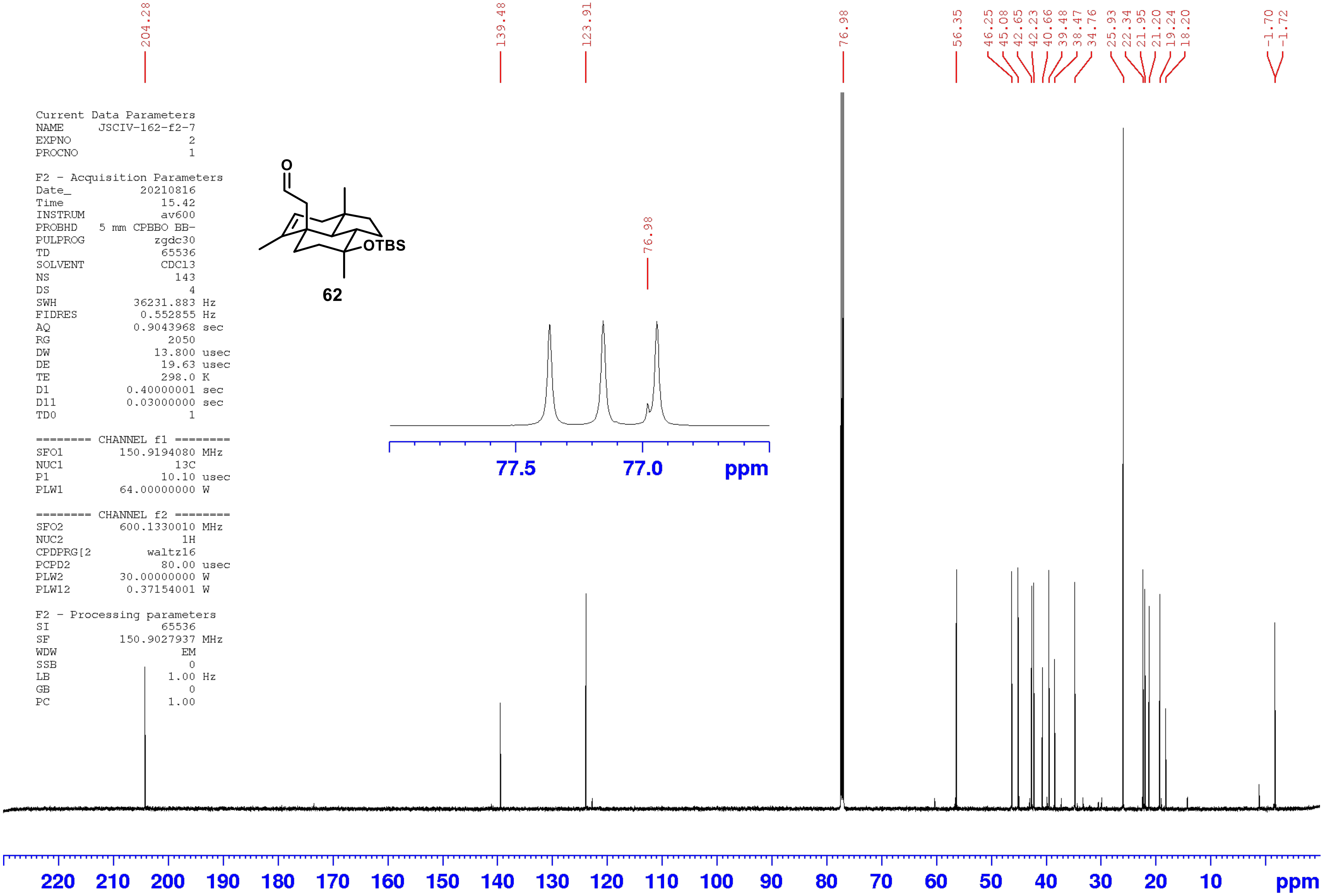

62

Current Data Parameters
NAME JC 5-003 dibal bot col
EXPNO 1
PROCNO 1

F2 - Acquisition Parameters
Date_ 20210423
Time 18.43
INSTRUM cryo500
PROBHD 5 mm CPTCI 1H-
PULPROG zg30
TD 81728
SOLVENT CDCl3
NS 8
DS 2
SWH 8012.820 Hz
FIDRES 0.098043 Hz
AQ 5.0998273 sec
RG 4
DW 62.400 usec
DE 6.00 usec
TE 298.0 K
D1 0.10000000 sec
MCREST 0 sec
MCWRK 0.01500000 sec

======== CHANNEL f1 ========
NUC1 1H
P1 9.75 usec
PL1 1.60 dB
SFO1 500.2235015 MHz

F2 - Processing parameters
SI 65536
SF 500.2200312 MHz
WDW EM
SSB 0
LB 0.30 Hz
GB 0
PC 2.00

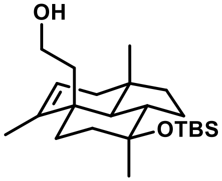

S18

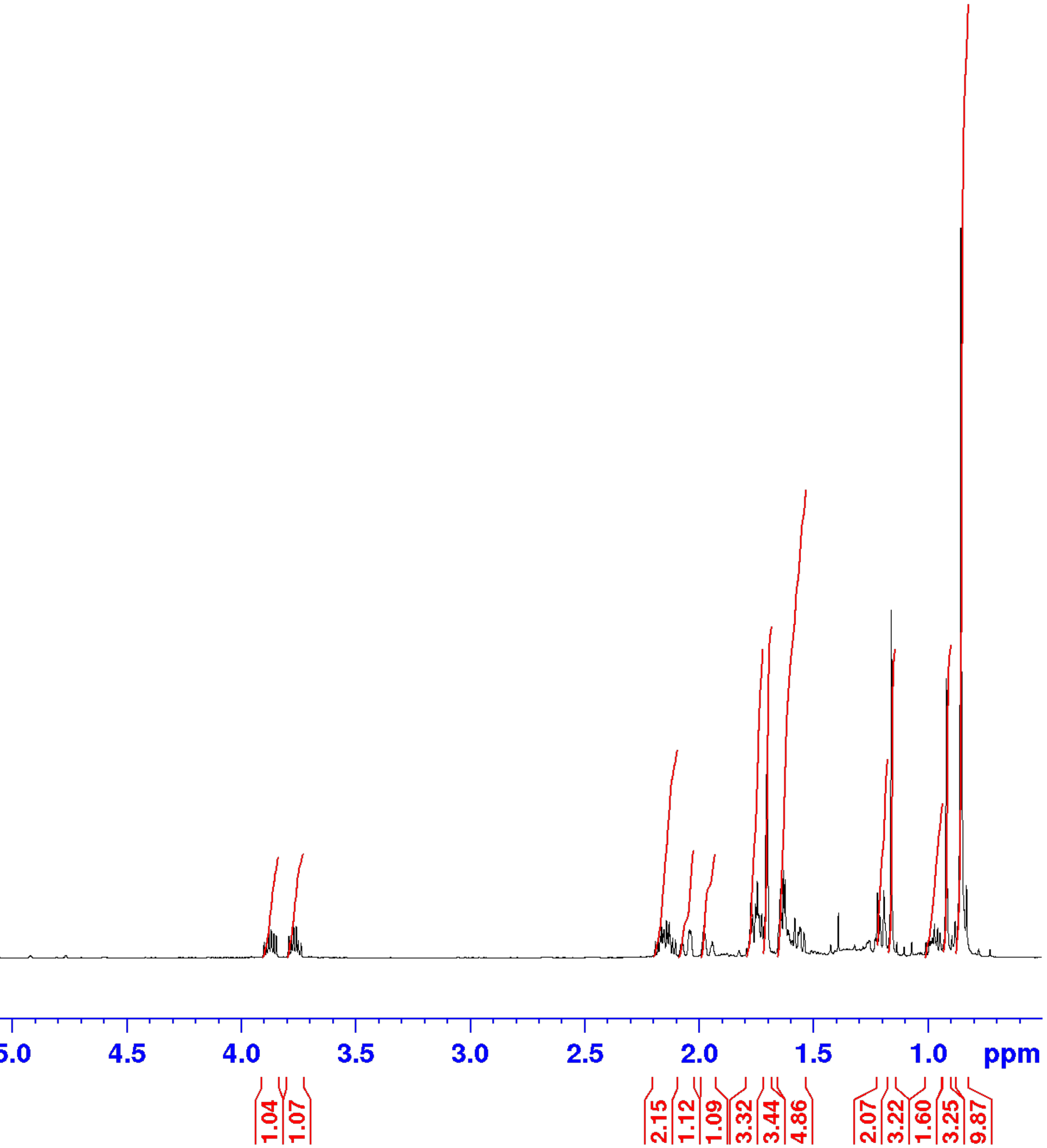

```
Current Data Parameters
NAME     JC 5-003 dibal bot col
EXPNO              2
PROCNO             1

F2 - Acquisition Parameters
Date_          20210423
Time              18.45
INSTRUM         cryo500
PROBHD   5 mm CPTCI 1H-
PULPROG  SpinEchopg30gp2.prd
TD                65536
SOLVENT           CDCl3
NS                   32
DS                   16
SWH           30303.031 Hz
FIDRES         0.462388 Hz
AQ            1.0813440 sec
RG               7298.2
DW               16.500 usec
DE                 6.00 usec
TE                298.0 K
D1           0.25000000 sec
d11          0.03000000 sec
D16          0.00020000 sec
d17          0.00019600 sec
MCREST                0 sec
MCWRK        0.01500000 sec
P2                37.70 usec

======== CHANNEL f1 ========
NUC1                13C
P1                18.85 usec
P12             2000.00 usec
P20              500.00 usec
PL0              120.00 dB
PL1               -1.00 dB
SFO1        125.7942548 MHz
SP2                1.55 dB
SP4                1.55 dB
SPNAM[2]    Crp60comp.4
SPNAM[4] Crp60,0.5,20.1
SPOFF2                0 Hz
SPOFF4                0 Hz

======== CHANNEL f2 ========
CPDPRG[2         waltz16
NUC2                 1H
PCPD2            100.00 usec
PL2                1.60 dB
PL12              22.00 dB
SFO2        500.2225011 MHz

====== GRADIENT CHANNEL =====
GPNAM[1]       SINE.100
GPNAM[2]       SINE.100
GPX1                  0 %
GPX2                  0 %
GPY1                  0 %
GPY2                  0 %
GPZ1              30.00 %
GPZ2              50.00 %
p15              500.00 usec
p16             1000.00 usec

F2 - Processing parameters
SI                65536
SF          125.7804066 MHz
WDW                  EM
SSB                   0
LB                 1.00 Hz
GB                    0
PC                 2.00
```

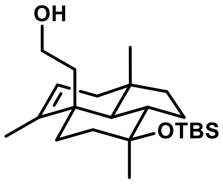

S18

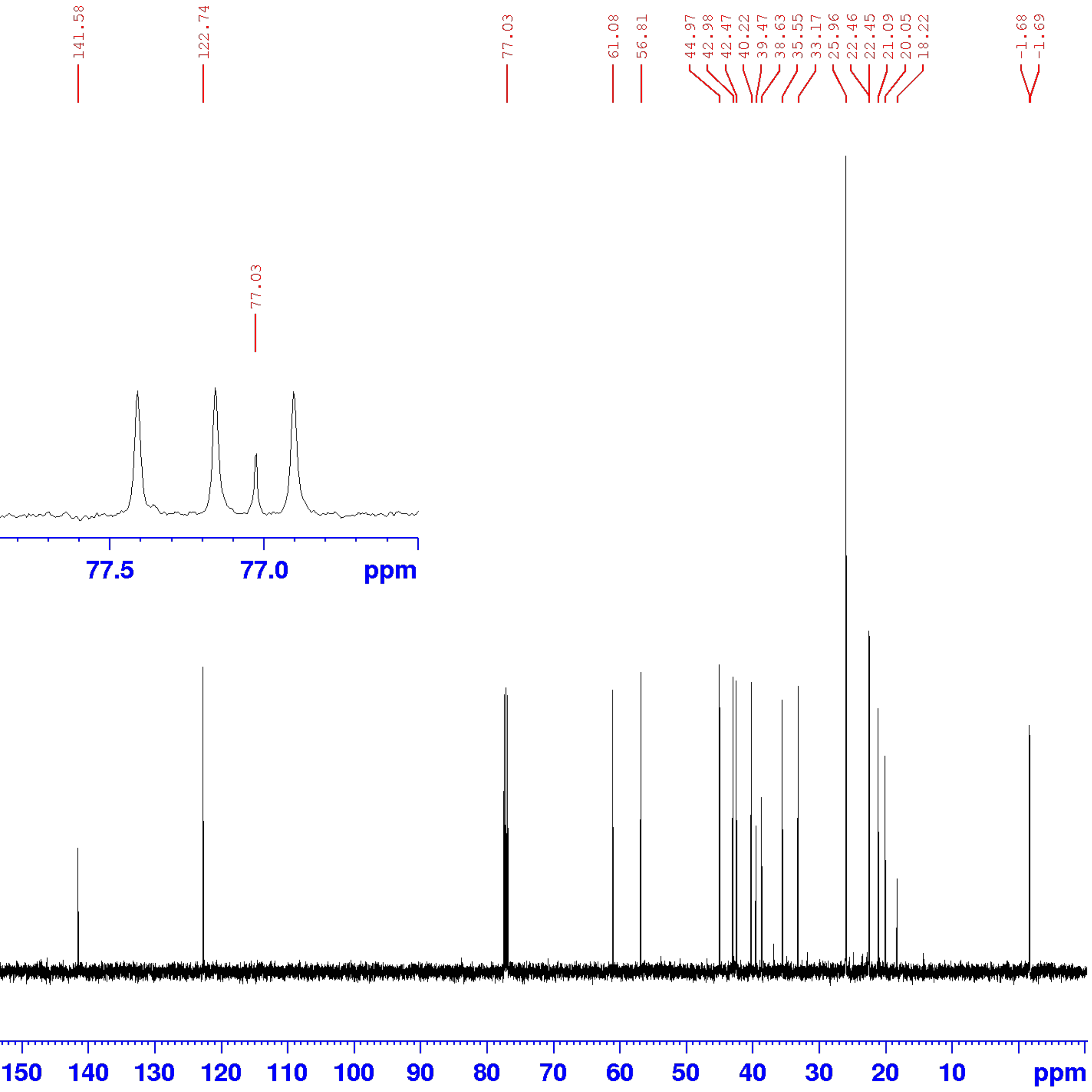

```
Current Data Parameters
NAME      JSCIV-242-f24-80
EXPNO                    1
PROCNO                   1

F2 - Acquisition Parameters
Date_             20211008
Time                 19.16
INSTRUM            cryo500
PROBHD   5 mm CPTCI 1H-
PULPROG               zg30
TD                   81728
SOLVENT              CDCl3
NS                       8
DS                       2
SWH              8012.820 Hz
FIDRES           0.098043 Hz
AQ              5.0998273 sec
RG                     7.1
DW                  62.400 usec
DE                    6.00 usec
TE                   298.0 K
D1              0.10000000 sec
MCREST                   0 sec
MCWRK           0.01500000 sec

======== CHANNEL f1 ========
NUC1                    1H
P1                    9.75 usec
PL1                   1.60 dB
SFO1           500.2235015 MHz

F2 - Processing parameters
SI                   65536
SF             500.2200311 MHz
WDW                     EM
SSB                      0
LB                    0.30 Hz
GB                       0
PC                    1.00
```

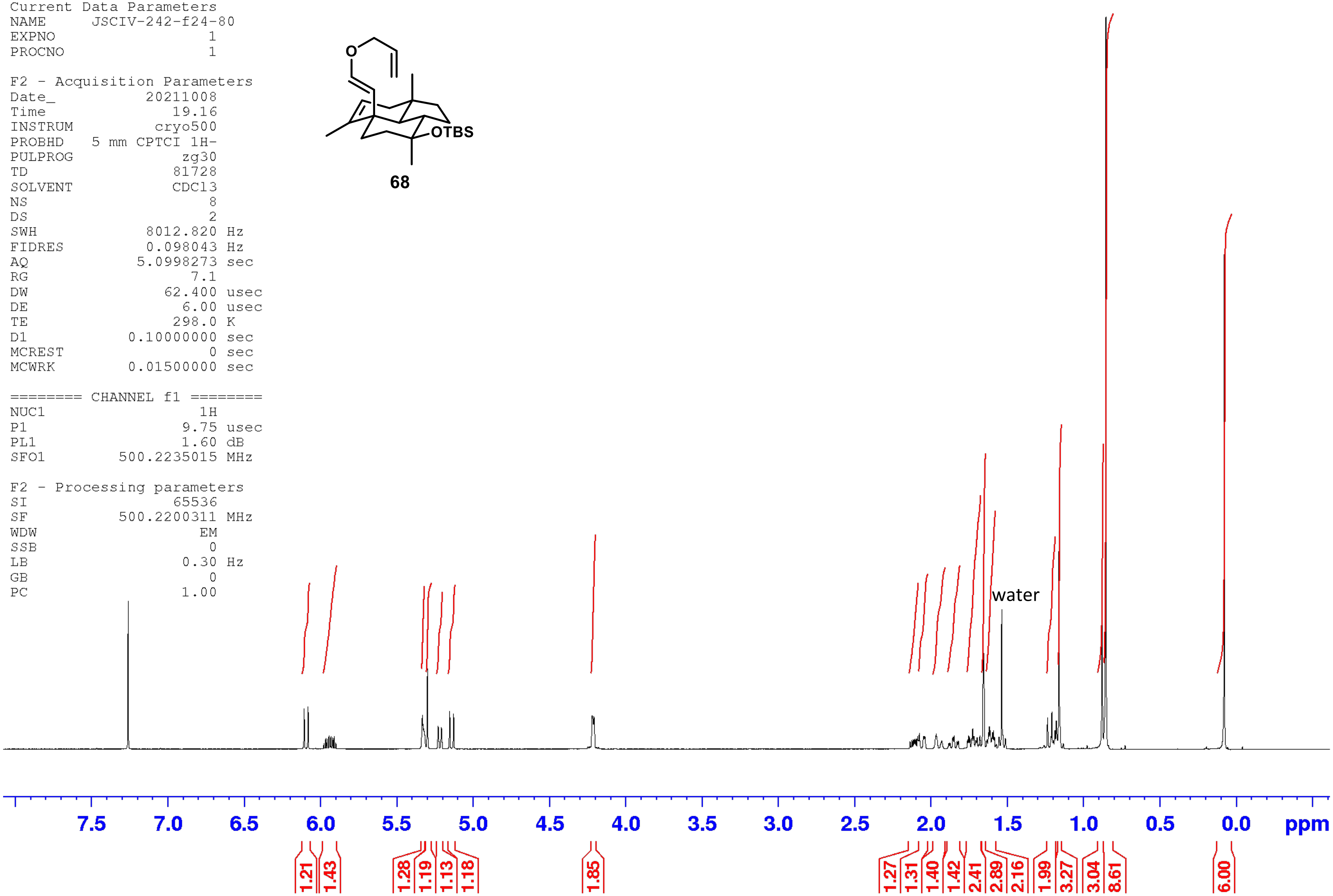

68

```
Current Data Parameters
NAME       JSCIV-242-f24-80
EXPNO                     2
PROCNO                    1

F2 - Acquisition Parameters
Date_              20211008
Time                  19.20
INSTRUM             cryo500
PROBHD    5 mm CPTCI 1H-
PULPROG   SpinEchopg30gp2.prd
TD                    65536
SOLVENT               CDCl3
NS                      120
DS                       16
SWH           30303.031 Hz
FIDRES         0.462388 Hz
AQ            1.0813440 sec
RG                   7298.2
DW               16.500 usec
DE                 6.00 usec
TE                298.0 K
D1           0.25000000 sec
d11          0.03000000 sec
D16          0.00020000 sec
d17          0.00019600 sec
MCREST                0 sec
MCWRK        0.01500000 sec
P2                37.70 usec

======== CHANNEL f1 ========
NUC1                    13C
P1                18.85 usec
P12             2000.00 usec
P20              500.00 usec
PL0              120.00 dB
PL1               -1.00 dB
SFO1        125.7942548 MHz
SP2                1.55 dB
SP4                1.55 dB
SPNAM[2]     Crp60comp.4
SPNAM[4]  Crp60,0.5,20.1
SPOFF2                0 Hz
SPOFF4                0 Hz

======== CHANNEL f2 ========
CPDPRG[2          waltz16
NUC2                     1H
PCPD2            100.00 usec
PL2                1.60 dB
PL12              22.00 dB
SFO2        500.2225011 MHz

====== GRADIENT CHANNEL =====
GPNAM[1]          SINE.100
GPNAM[2]          SINE.100
GPX1                  0 %
GPX2                  0 %
GPY1                  0 %
GPY2                  0 %
GPZ1              30.00 %
GPZ2              50.00 %
p15              500.00 usec
p16             1000.00 usec

F2 - Processing parameters
SI                    65536
SF          125.7804190 MHz
WDW                      EM
SSB                       0
LB                 1.00 Hz
GB                        0
PC                     2.00
```

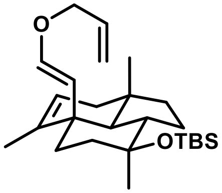

68

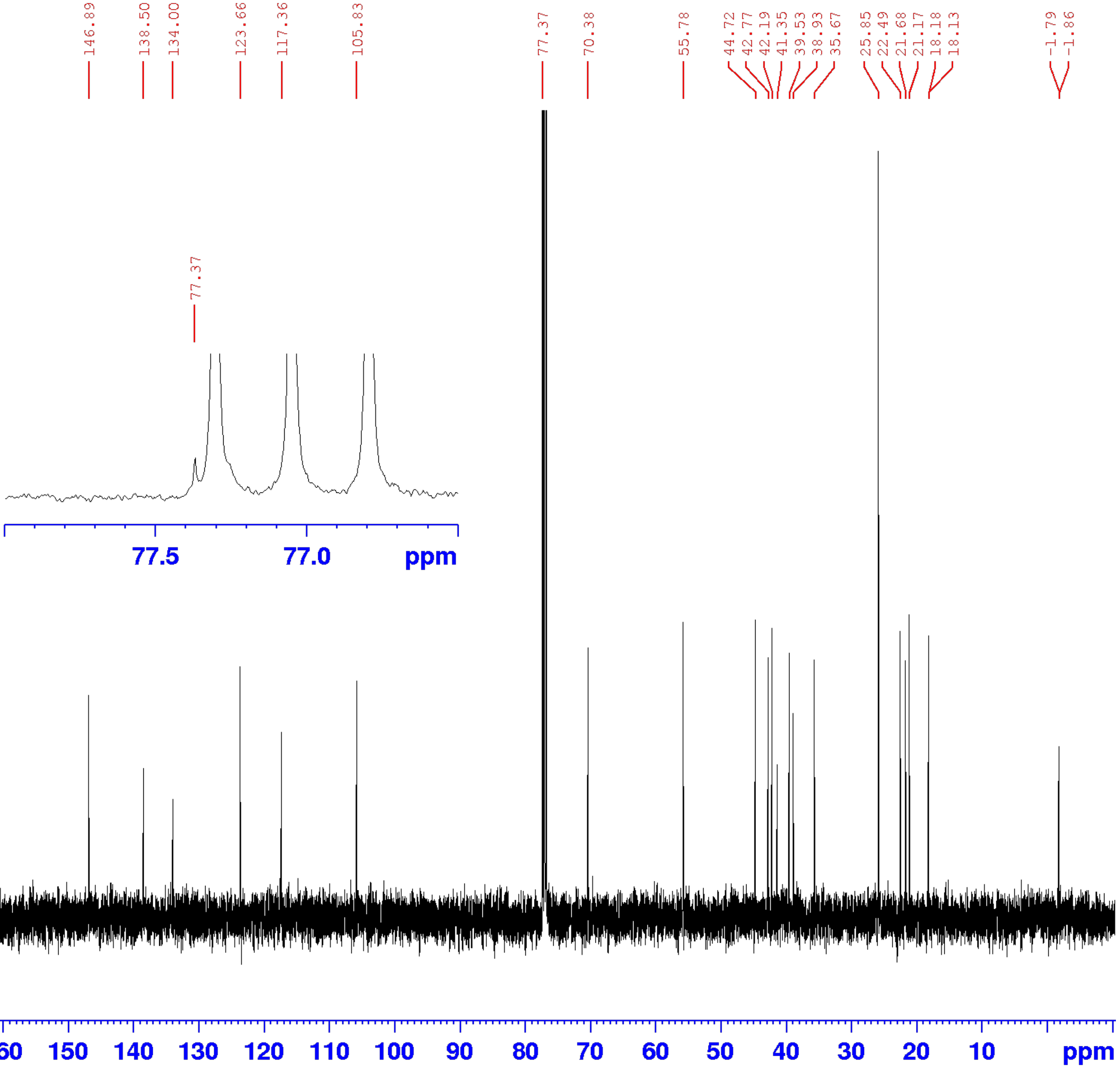

```
Current Data Parameters
NAME       JSCIV-197-f4-7
EXPNO                   1
PROCNO                  1

F2 - Acquisition Parameters
Date_           20210907
Time               19.05
INSTRUM            av600
PROBHD    5 mm CPBBO BB-
PULPROG             zg30
TD                 48074
SOLVENT            CDCl3
NS                    16
DS                     2
SWH             9615.385 Hz
FIDRES          0.200012 Hz
AQ             2.4998479 sec
RG                    10
DW                52.000 usec
DE                 14.23 usec
TE                 298.0 K
D1            3.00000000 sec
TD0                    1

======== CHANNEL f1 ========
SFO1        600.1342009 MHz
NUC1                 1H
P1                  9.50 usec
PLW1         30.00000000 W

F2 - Processing parameters
SI                 65536
SF           600.1300352 MHz
WDW                   EM
SSB                    0
LB                  0.30 Hz
GB                     0
PC                  1.00
```

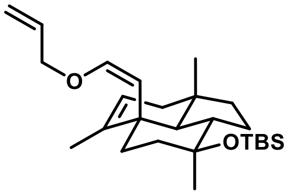

S19

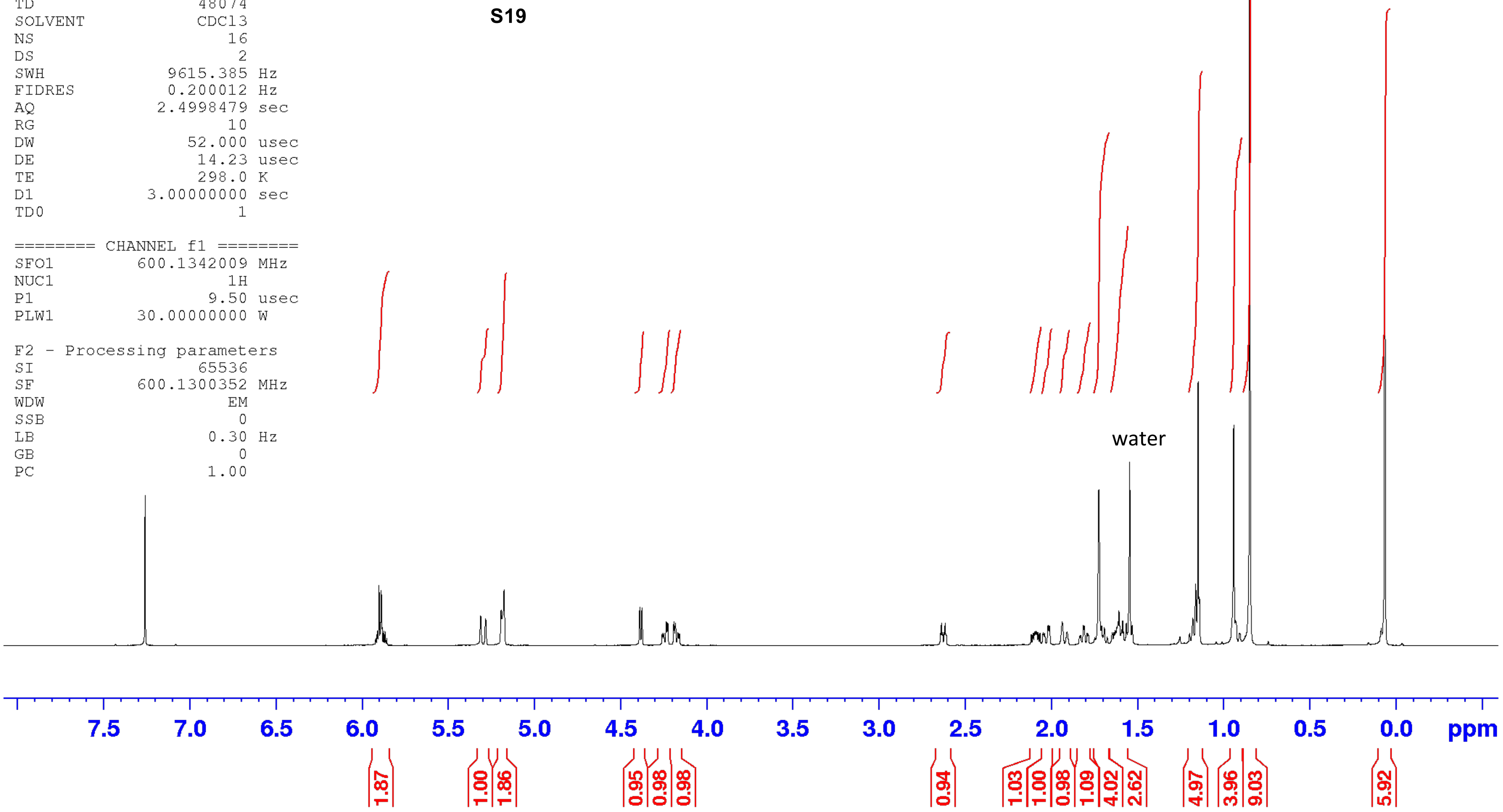

Current Data Parameters
NAME JSCV-146-col2-f2-6
EXPNO 2
PROCNO 1

F2 - Acquisition Parameters
Date_ 20220521
Time 14.55
INSTRUM av600
PROBHD 5 mm CPBBO BB-
PULPROG zgdc30
TD 65536
SOLVENT CDCl3
NS 198
DS 4
SWH 36231.883 Hz
FIDRES 0.552855 Hz
AQ 0.9043968 sec
RG 2050
DW 13.800 usec
DE 19.63 usec
TE 297.9 K
D1 0.40000001 sec
D11 0.03000000 sec
TD0 1

======== CHANNEL f1 ========
SFO1 150.9194080 MHz
NUC1 13C
P1 10.10 usec
PLW1 64.00000000 W

======== CHANNEL f2 ========
SFO2 600.1330010 MHz
NUC2 1H
CPDPRG[2 waltz16
PCPD2 80.00 usec
PLW2 30.00000000 W
PLW12 0.37154001 W

F2 - Processing parameters
SI 65536
SF 150.9027932 MHz
WDW EM
SSB 0
LB 1.00 Hz
GB 0
PC 1.00

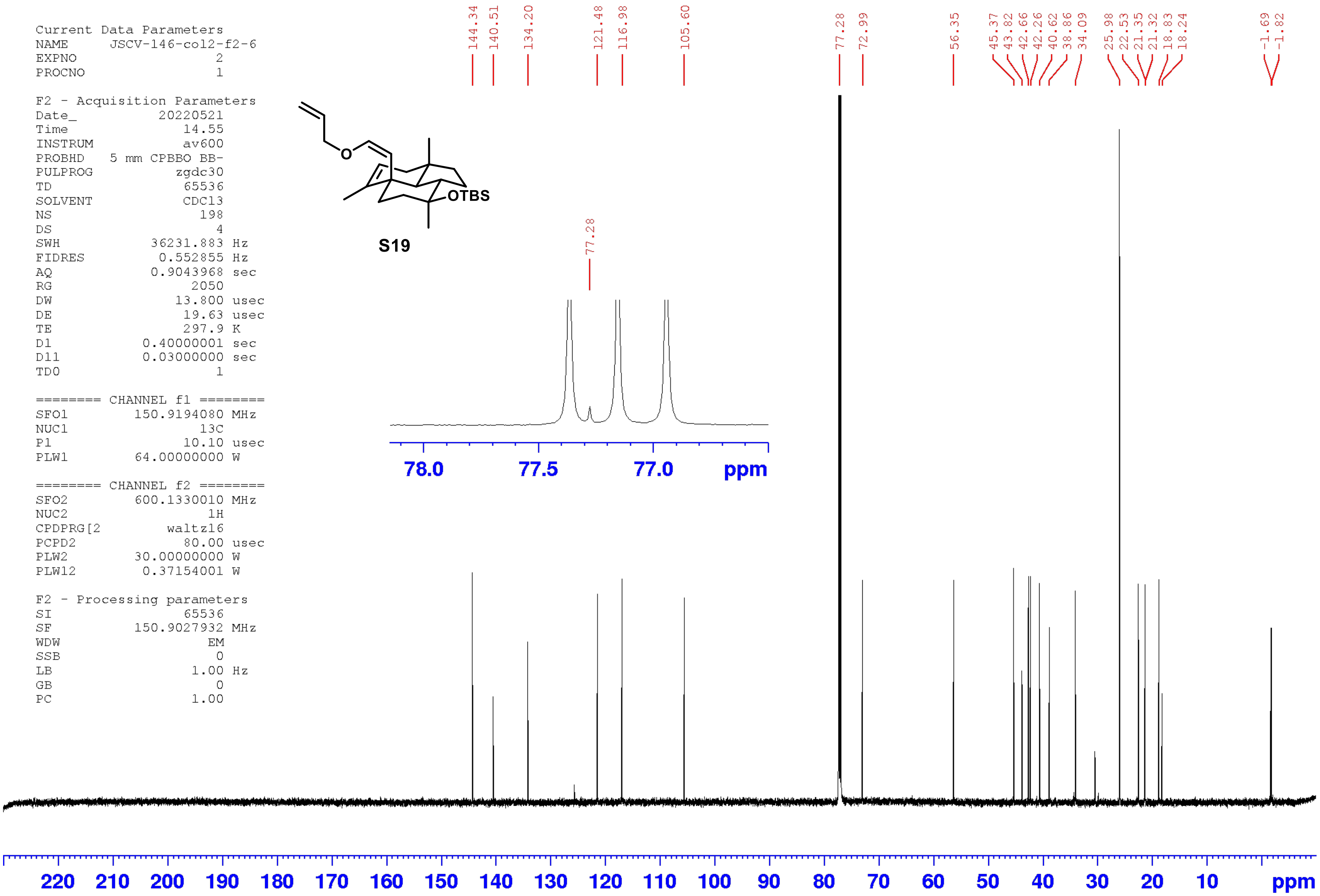

```
Current Data Parameters
NAME      JC 5-007 claisen TOP col
EXPNO              1
PROCNO             1

F2 - Acquisition Parameters
Date_          20210428
Time              20.55
INSTRUM           av600
PROBHD   5 mm CPBBO BB-
PULPROG            zg30
TD                98074
SOLVENT           CDCl3
NS                    8
DS                    2
SWH            9615.385 Hz
FIDRES         0.098042 Hz
AQ            5.0998478 sec
RG                    9
DW               52.000 usec
DE                13.70 usec
TE                298.0 K
D1           0.10000000 sec
TD0                   1

======== CHANNEL f1 ========
SFO1        600.1342009 MHz
NUC1                 1H
P1                12.00 usec
PLW1        28.00000000 W

F2 - Processing parameters
SI                65536
SF          600.1300344 MHz
WDW                  EM
SSB      0
LB                 0.30 Hz
GB       0
PC                 1.00
```

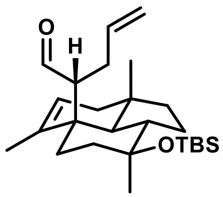

70

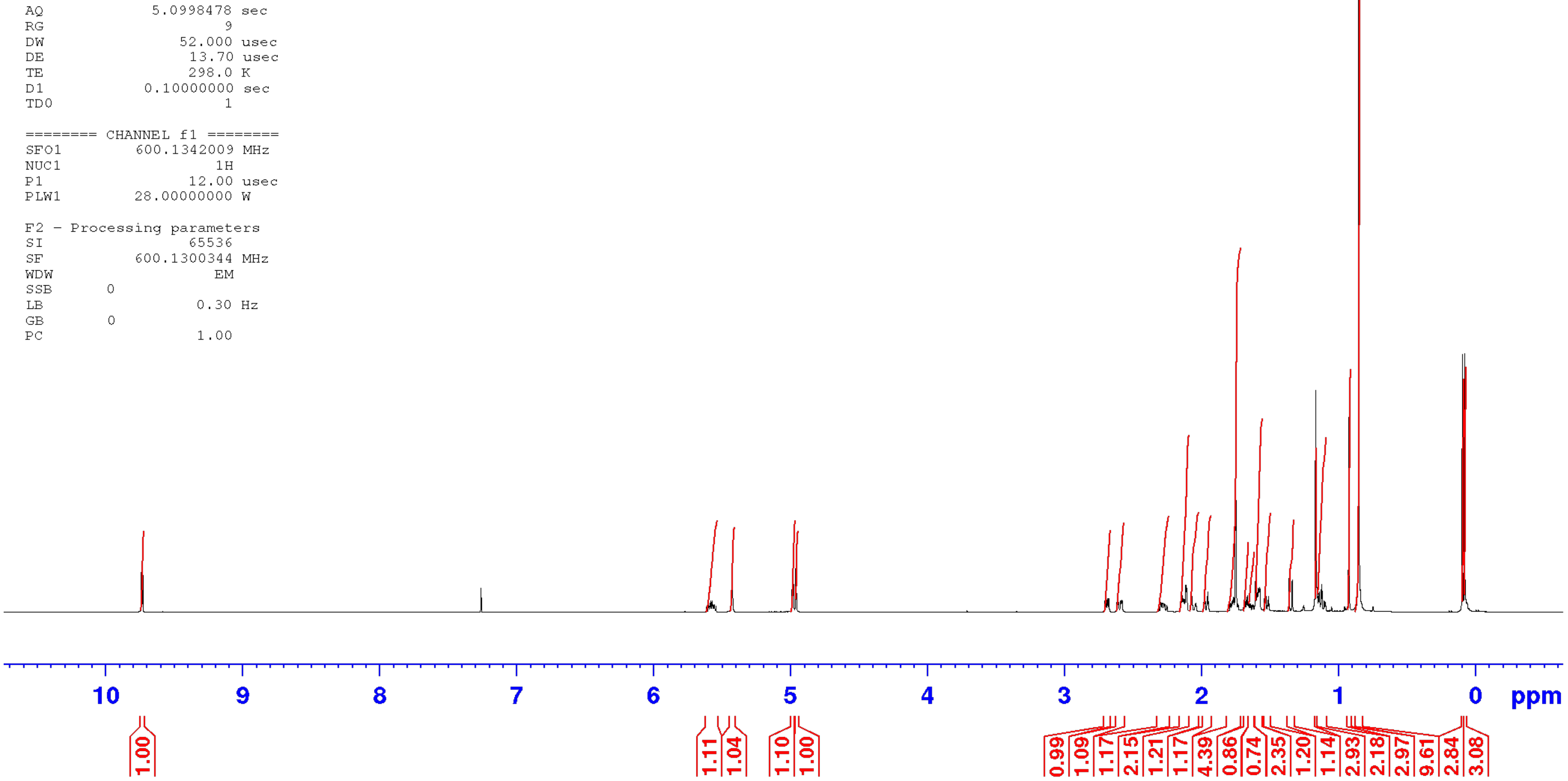

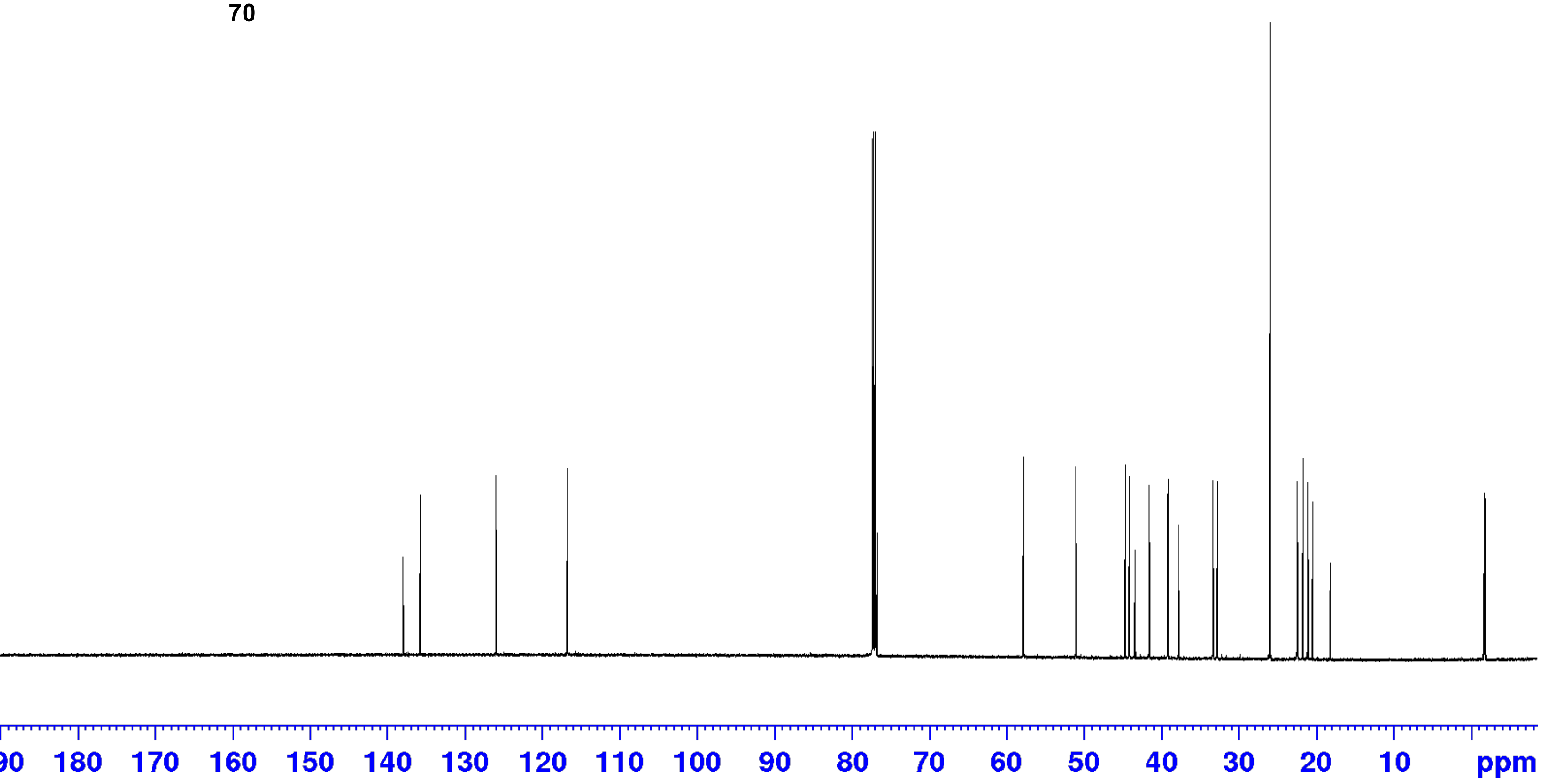


```
Current Data Parameters
NAME      JC 5-007 claisen TOP col
EXPNO                  2
PROCNO                 1

F2 - Acquisition Parameters
Date_           20210428
Time               21.00
INSTRUM            av600
PROBHD   5 mm CPBBO BB-
PULPROG          zgdc30
TD                 65536
SOLVENT            CDCl3
NS                   168
DS                     4
SWH            36231.883 Hz
FIDRES          0.552855 Hz
AQ             0.9043968 sec
RG                  2050
DW                13.800 usec
DE                 19.65 usec
TE                 298.0 K
D1            0.40000001 sec
D11           0.03000000 sec
TD0                    1

======== CHANNEL f1 ========
SFO1        150.9194075 MHz
NUC1                13C
P1                 10.00 usec
PLW1        65.00000000 W

======== CHANNEL f2 ========
SFO2        600.1330010 MHz
NUC2                 1H
CPDPRG[2         waltz16
PCPD2              80.00 usec
PLW2        28.00000000 W
PLW12        0.63000000 W

F2 - Processing parameters
SI                 65536
SF           150.9027935 MHz
WDW                   EM
SSB      0
LB                  1.00 Hz
GB       0
PC                  1.00
```

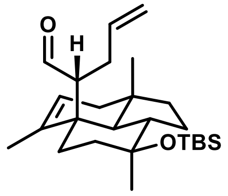

70

Current Data Parameters
NAME JC 5-065 bot
EXPNO 1
PROCNO 1

F2 - Acquisition Parameters
Date_ 20210708
Time 23.53
INSTRUM cryo500
PROBHD 5 mm CPTCI 1H-
PULPROG zg30
TD 16022
SOLVENT CDCl3
NS 8
DS 2
SWH 8012.820 Hz
FIDRES 0.500114 Hz
AQ 0.9997728 sec
RG 5
DW 62.400 usec
DE 6.00 usec
TE 298.0 K
D1 0.10000000 sec
MCREST 0 sec
MCWRK 0.01500000 sec

======== CHANNEL f1 ========
NUC1 1H
P1 9.75 usec
PL1 1.60 dB
SFO1 500.2235015 MHz

F2 - Processing parameters
SI 65536
SF 500.2200315 MHz
WDW EM
SSB 0
LB 0.30 Hz
GB 0
PC 1.00

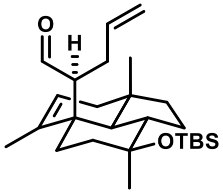

69

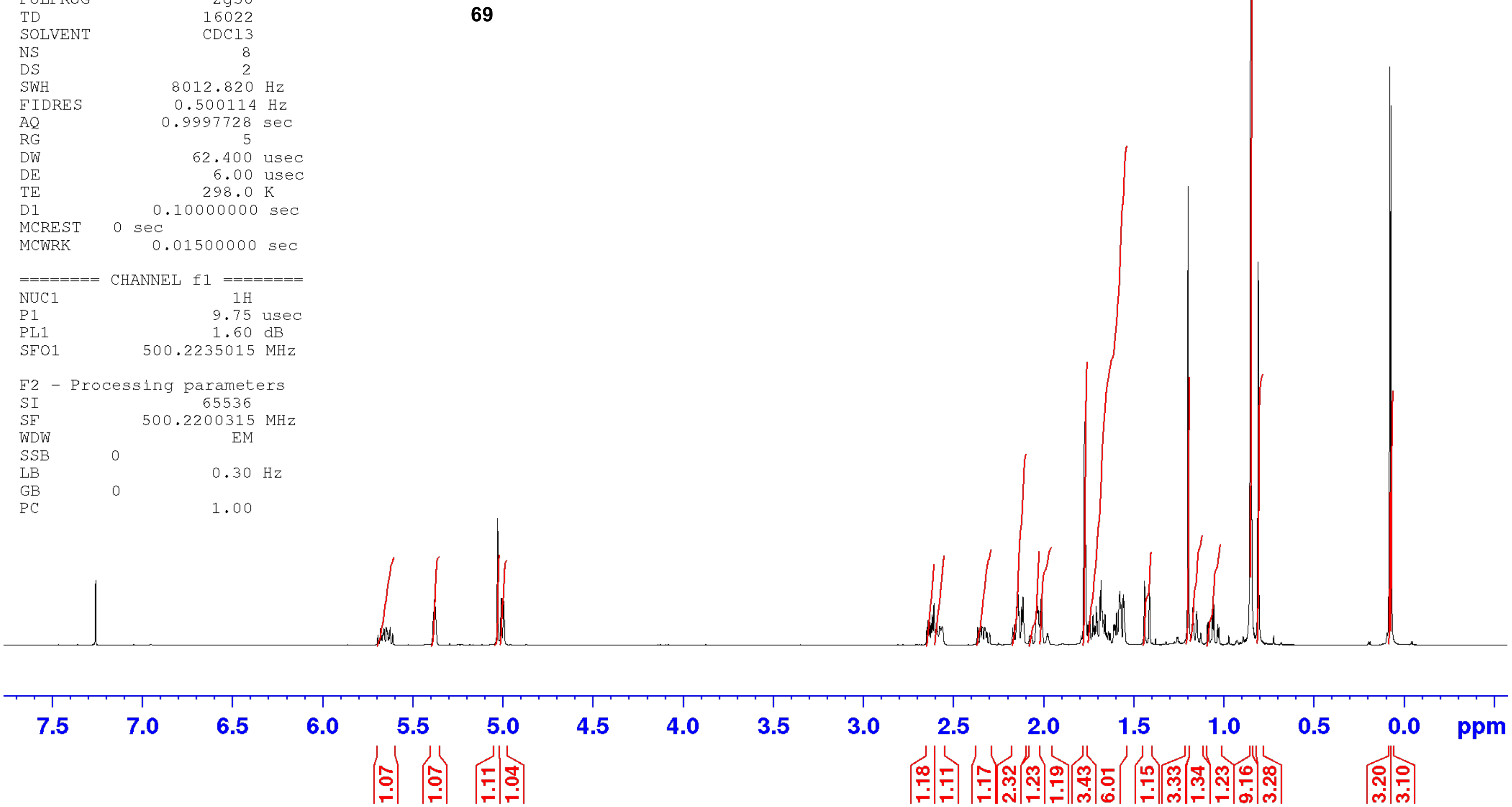

```
Current Data Parameters
NAME        JC 5-065 bot
EXPNO                  2
PROCNO                 1

F2 - Acquisition Parameters
Date_           20210708
Time               23.55
INSTRUM          cryo500
PROBHD   5 mm CPTCI 1H-
PULPROG  SpinEchopg30gp2.prd
TD                 65536
SOLVENT            CDCl3
NS                    77
DS                    16
SWH            30303.031 Hz
FIDRES          0.462388 Hz
AQ             1.0813440 sec
RG                7298.2
DW                16.500 usec
DE                  6.00 usec
TE                 298.0 K
D1            0.25000000 sec
d11           0.03000000 sec
D16           0.00020000 sec
d17           0.00019600 sec
MCREST   0 sec
MCWRK         0.01500000 sec
P2                 37.70 usec

======== CHANNEL f1 ========
NUC1                 13C
P1                 18.85 usec
P12              2000.00 usec
P20               500.00 usec
PL0               120.00 dB
PL1                -1.00 dB
SFO1         125.7942548 MHz
SP2                 1.55 dB
SP4                 1.55 dB
SPNAM[2]     Crp60comp.4
SPNAM[4] Crp60,0.5,20.1
SPOFF2   0 Hz
SPOFF4   0 Hz

======== CHANNEL f2 ========
CPDPRG[2]        waltz16
NUC2                  1H
PCPD2             100.00 usec
PL2                 1.60 dB
PL12               22.00 dB
SFO2         500.2225011 MHz

====== GRADIENT CHANNEL =====
GPNAM[1]        SINE.100
GPNAM[2]        SINE.100
GPX1      0 %
GPX2      0 %
GPY1      0 %
GPY2      0 %
GPZ1               30.00 %
GPZ2               50.00 %
p15               500.00 usec
p16              1000.00 usec

F2 - Processing parameters
SI                 65536
SF           125.7804062 MHz
WDW                   EM
SSB      0
LB                  1.00 Hz
GB       0
PC                  2.00
```

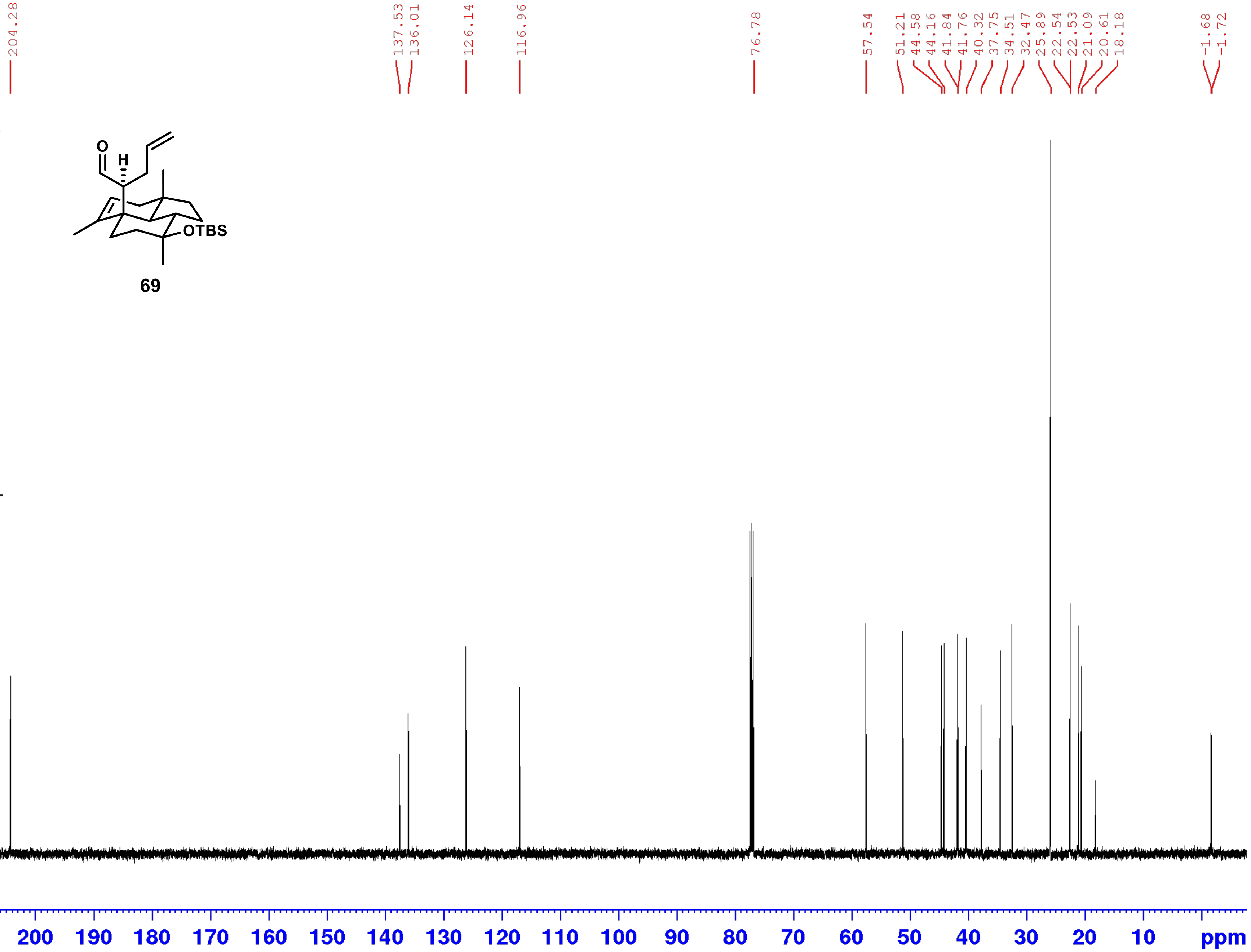

```
Current Data Parameters
NAME       JC 5-067 cr LAH
EXPNO                    1
PROCNO                   1

F2 - Acquisition Parameters
Date_             20210621
Time                  2.27
INSTRUM            cryo500
PROBHD    5 mm CPTCI 1H-
PULPROG               zg30
TD                   16022
SOLVENT              CDCl3
NS                       8
DS                       2
SWH               8012.820 Hz
FIDRES            0.500114 Hz
AQ               0.9997728 sec
RG                     3.6
DW                  62.400 usec
DE                    6.00 usec
TE                   298.0 K
D1              0.10000000 sec
MCREST                   0 sec
MCWRK           0.01500000 sec

======== CHANNEL f1 ========
NUC1                    1H
P1                    9.75 usec
PL1                   1.60 dB
SFO1           500.2235015 MHz

F2 - Processing parameters
SI                   65536
SF             500.2200309 MHz
WDW                     EM
SSB                      0
LB                    0.30 Hz
GB                       0
PC                    1.00
```

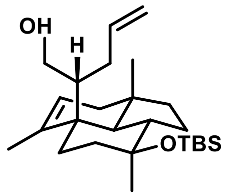

S20

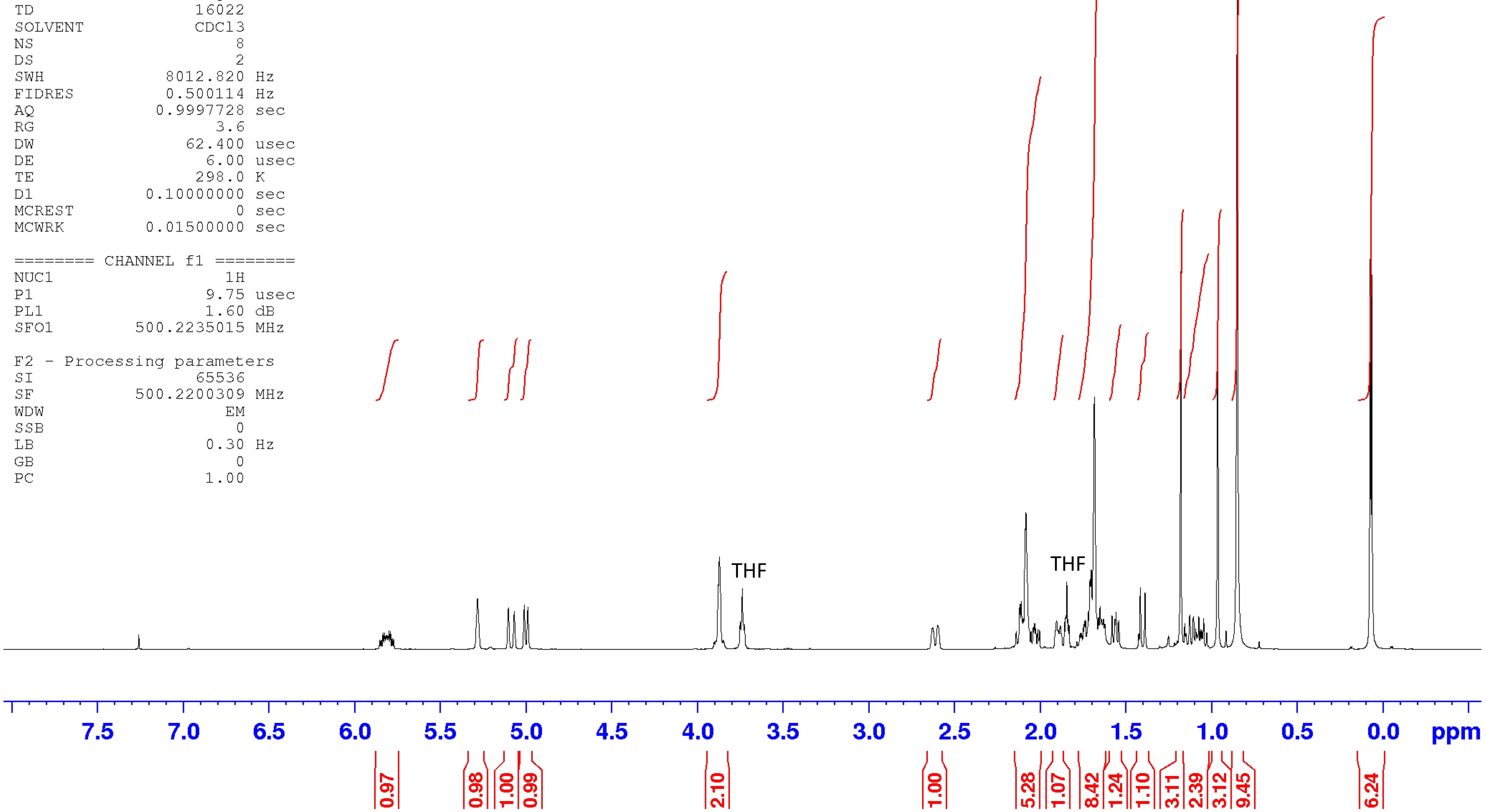

```
Current Data Parameters
NAME      JC 5-067 cr LAH
EXPNO                 2
PROCNO                1

F2 - Acquisition Parameters
Date_          20210621
Time               2.29
INSTRUM         cryo500
PROBHD   5 mm CPTCI 1H-
PULPROG  SpinEchopg30gp2.prd
TD                65536
SOLVENT           CDCl3
NS                   36
DS                   16
SWH           30303.031 Hz
FIDRES         0.462388 Hz
AQ            1.0813440 sec
RG               2580.3
DW               16.500 usec
DE                 6.00 usec
TE                298.0 K
D1           0.25000000 sec
d11          0.03000000 sec
D16          0.00020000 sec
d17          0.00019600 sec
MCREST                0 sec
MCWRK        0.01500000 sec
P2                37.70 usec

======== CHANNEL f1 ========
NUC1                13C
P1                18.85 usec
P12             2000.00 usec
P20              500.00 usec
PL0              120.00 dB
PL1               -1.00 dB
SFO1        125.7942548 MHz
SP2                1.55 dB
SP4                1.55 dB
SPNAM[2]     Crp60comp.4
SPNAM[4] Crp60,0.5,20.1
SPOFF2                0 Hz
SPOFF4                0 Hz

======== CHANNEL f2 ========
CPDPRG[2          waltz16
NUC2                 1H
PCPD2            100.00 usec
PL2                1.60 dB
PL12              22.00 dB
SFO2        500.2225011 MHz

====== GRADIENT CHANNEL =====
GPNAM[1]       SINE.100
GPNAM[2]       SINE.100
GPX1                  0 %
GPX2                  0 %
GPY1                  0 %
GPY2                  0 %
GPZ1              30.00 %
GPZ2              50.00 %
p15              500.00 usec
p16             1000.00 usec

F2 - Processing parameters
SI                65536
SF          125.7804077 MHz
WDW                  EM
SSB                   0
LB                 1.00 Hz
GB                    0
PC                 2.00
```

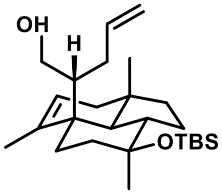

S20

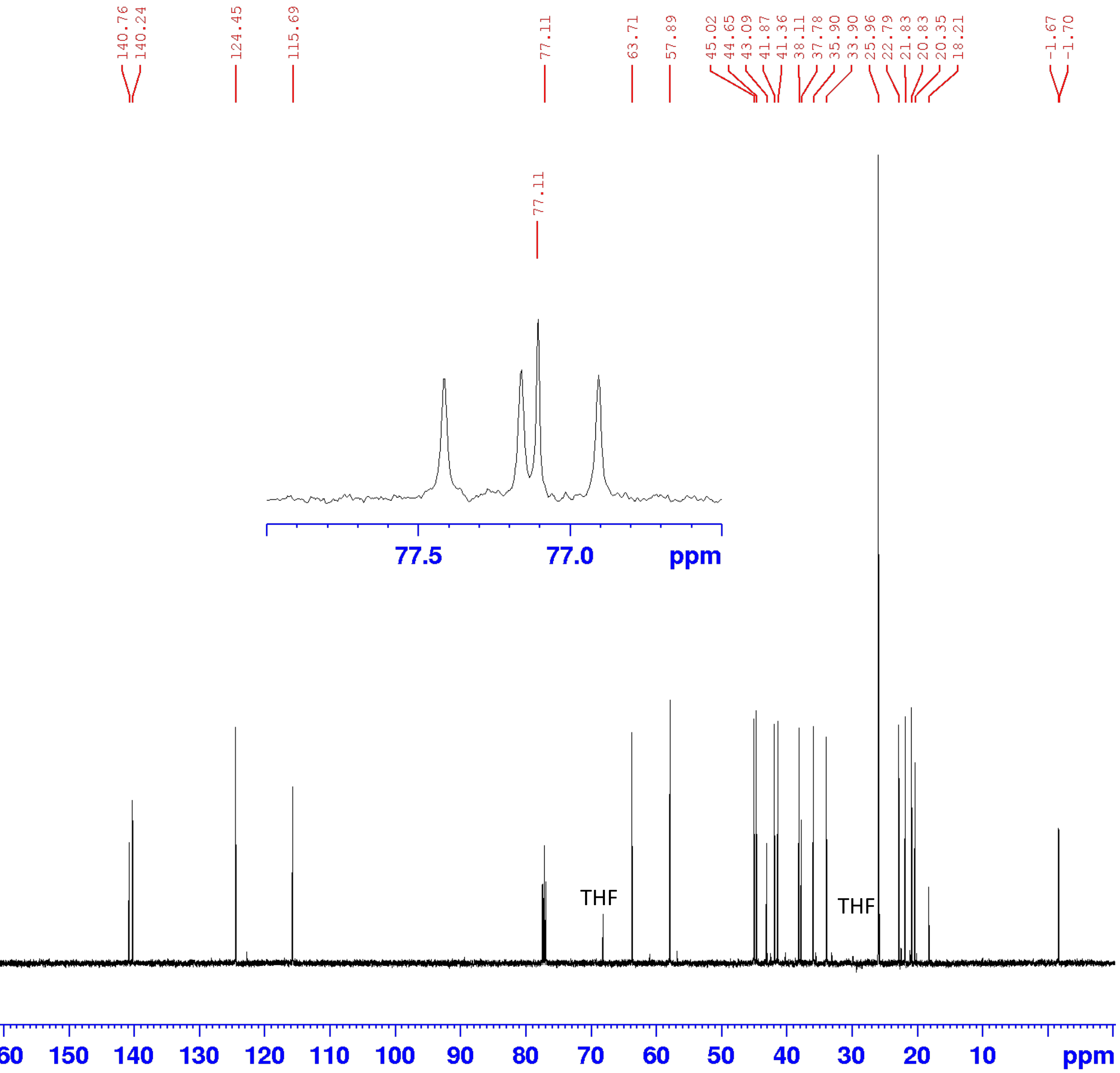

```
Current Data Parameters
NAME      JC 5-009 Ms col
EXPNO                 1
PROCNO                1

F2 - Acquisition Parameters
Date_            20210504
Time                 0.10
INSTRUM             av600
PROBHD   5 mm CPBBO BB-
PULPROG              zg30
TD                  98074
SOLVENT             CDCl3
NS                      8
DS                      2
SWH              9615.385 Hz
FIDRES           0.098042 Hz
AQ              5.0998478 sec
RG                      9
DW                 52.000 usec
DE                  13.70 usec
TE                  298.0 K
D1             0.10000000 sec
TD0                     1

======== CHANNEL f1 ========
SFO1        600.1342009 MHz
NUC1                 1H
P1                  12.00 usec
PLW1        28.00000000 W

F2 - Processing parameters
SI                  65536
SF          600.1300342 MHz
WDW                    EM
SSB      0
LB                   0.30 Hz
GB       0
PC                   1.00
```

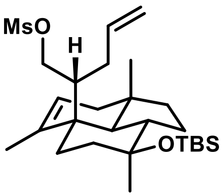

S21

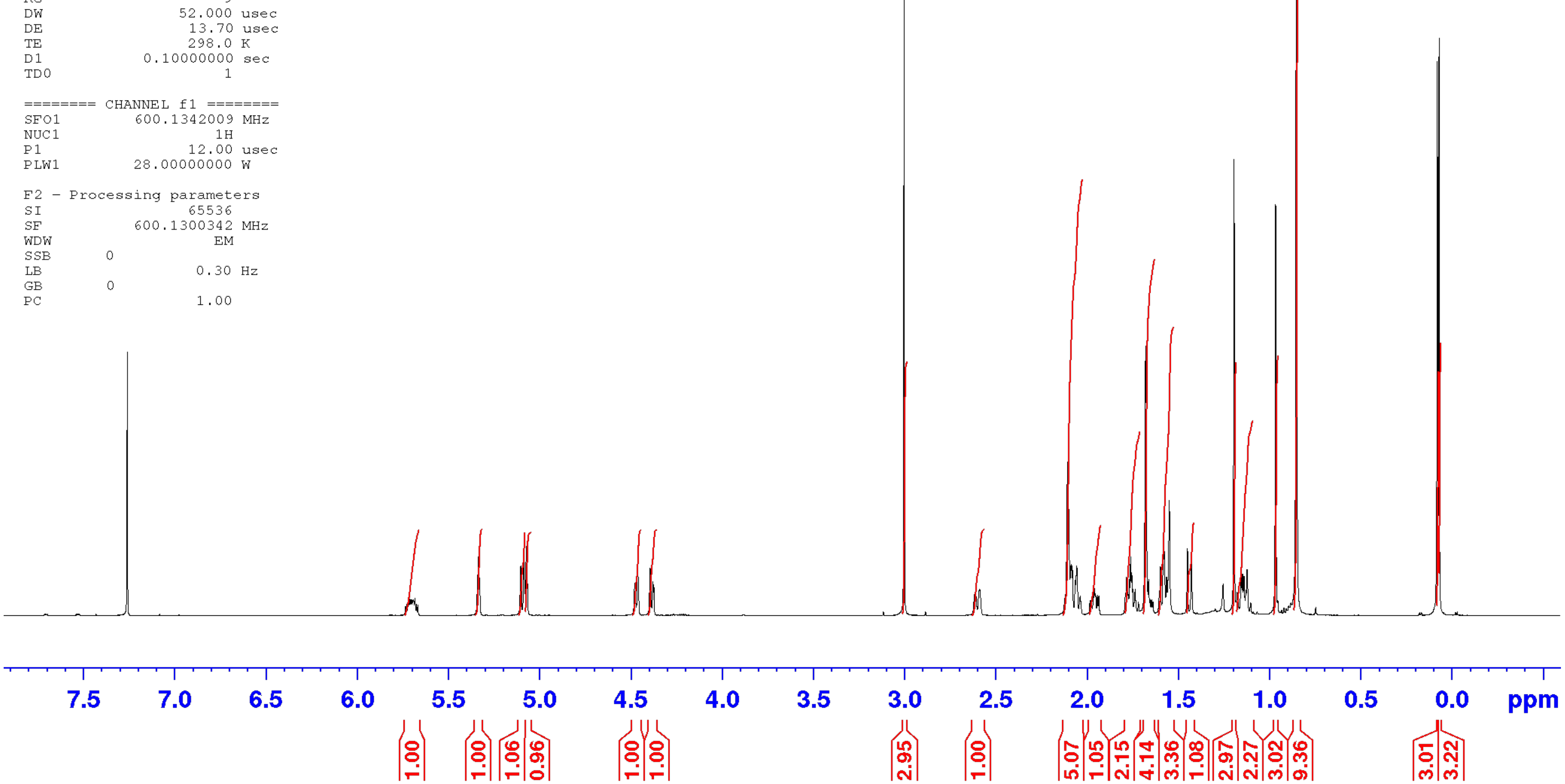

```
Current Data Parameters
NAME       JC 5-068 Ms col
EXPNO                  2
PROCNO                 1

F2 - Acquisition Parameters
Date_          20210622
Time               2.49
INSTRUM         cryo500
PROBHD   5 mm CPTCI 1H-
PULPROG  SpinEchopg30gp2.prd
TD                65536
SOLVENT           CDCl3
NS                  748
DS                   16
SWH           30303.031 Hz
FIDRES         0.462388 Hz
AQ            1.0813440 sec
RG                16384
DW               16.500 usec
DE                 6.00 usec
TE                298.0 K
D1           0.25000000 sec
d11          0.03000000 sec
D16          0.00020000 sec
d17          0.00019600 sec
MCREST   0 sec
MCWRK        0.01500000 sec
P2                37.70 usec

======== CHANNEL f1 ========
NUC1                13C
P1                18.85 usec
P12             2000.00 usec
P20              500.00 usec
PL0              120.00 dB
PL1               -1.00 dB
SFO1        125.7942548 MHz
SP2                1.55 dB
SP4                1.55 dB
SPNAM[2]     Crp60comp.4
SPNAM[4]  Crp60,0.5,20.1
SPOFF2   0 Hz
SPOFF4   0 Hz

======== CHANNEL f2 ========
CPDPRG[2         waltz16
NUC2                 1H
PCPD2            100.00 usec
PL2                1.60 dB
PL12              22.00 dB
SFO2        500.2225011 MHz

====== GRADIENT CHANNEL =====
GPNAM[1]        SINE.100
GPNAM[2]        SINE.100
GPX1      0 %
GPX2      0 %
GPY1      0 %
GPY2      0 %
GPZ1              30.00 %
GPZ2              50.00 %
p15              500.00 usec
p16             1000.00 usec

F2 - Processing parameters
SI                65536
SF          125.7804064 MHz
WDW                  EM
SSB      0
LB                 1.00 Hz
GB       0
PC                 2.00
```

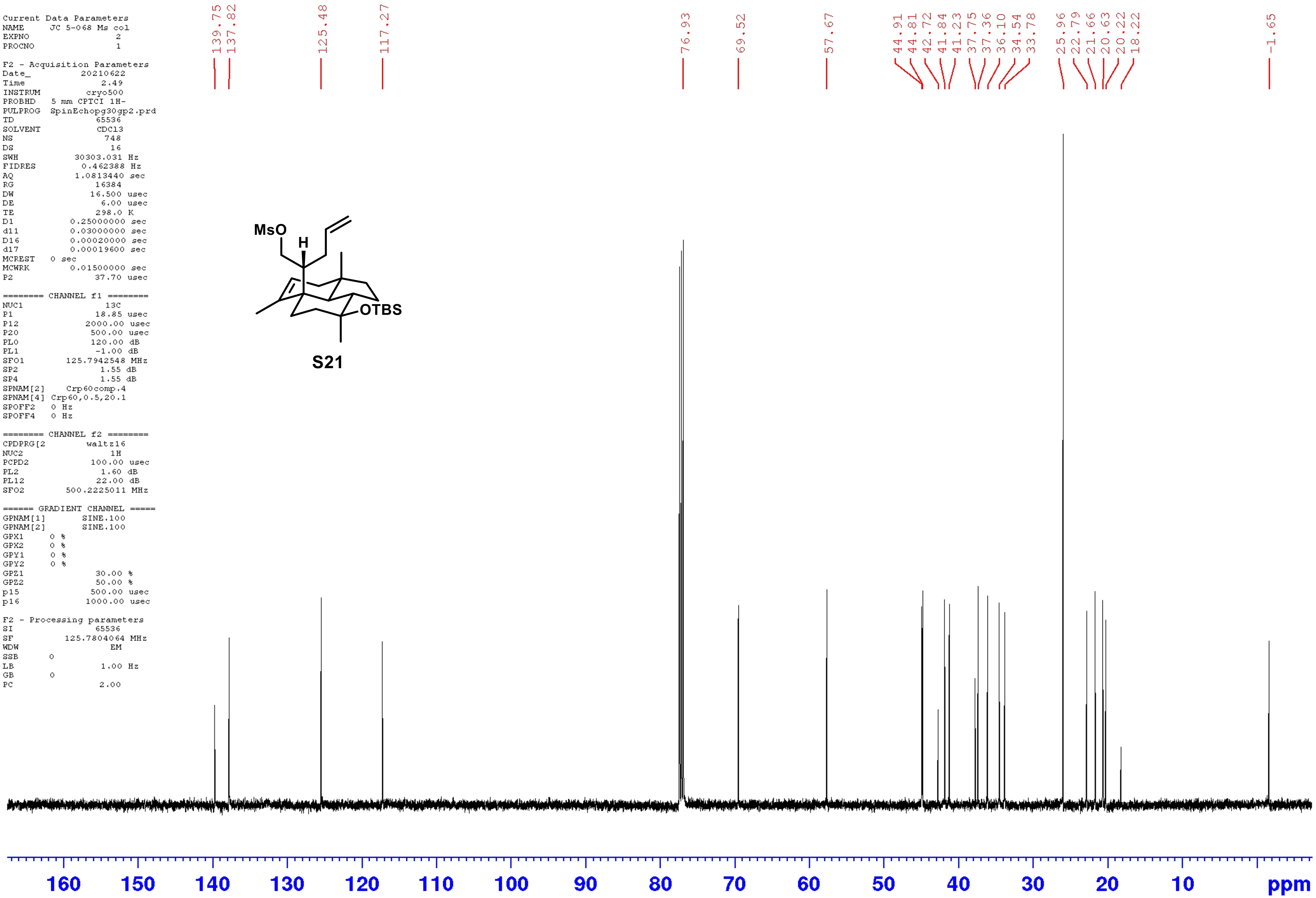

```
Current Data Parameters
NAME      JC 5-069 superH
EXPNO                 1
PROCNO                1

F2 - Acquisition Parameters
Date_           20210630
Time               18.31
INSTRUM          cryo500
PROBHD   5 mm CPTCI 1H-
PULPROG             zg30
TD                 16022
SOLVENT            CDCl3
NS                     8
DS                     2
SWH             8012.820 Hz
FIDRES          0.500114 Hz
AQ             0.9997728 sec
RG                   4.5
DW                62.400 usec
DE                  6.00 usec
TE                 298.0 K
D1            0.10000000 sec
MCREST   0 sec
MCWRK         0.01500000 sec

======== CHANNEL f1 ========
NUC1                  1H
P1                  9.75 usec
PL1                 1.60 dB
SFO1         500.2235015 MHz

F2 - Processing parameters
SI                 65536
SF           500.2200312 MHz
WDW                   EM
SSB       0
LB                  0.30 Hz
GB        0
PC                  1.00
```

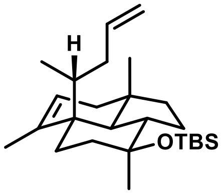

71

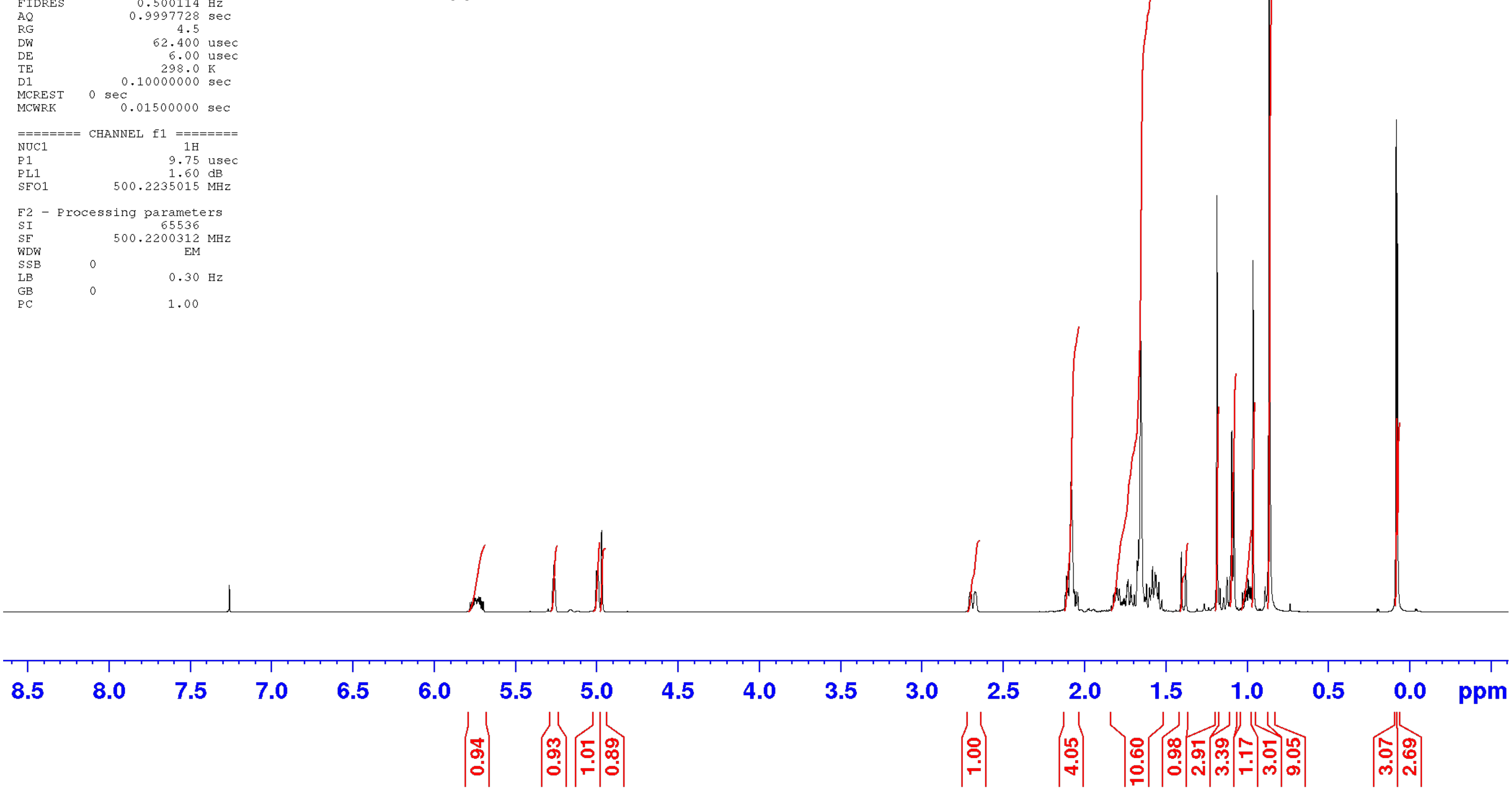

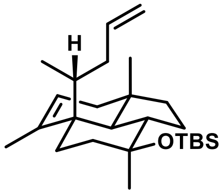

71

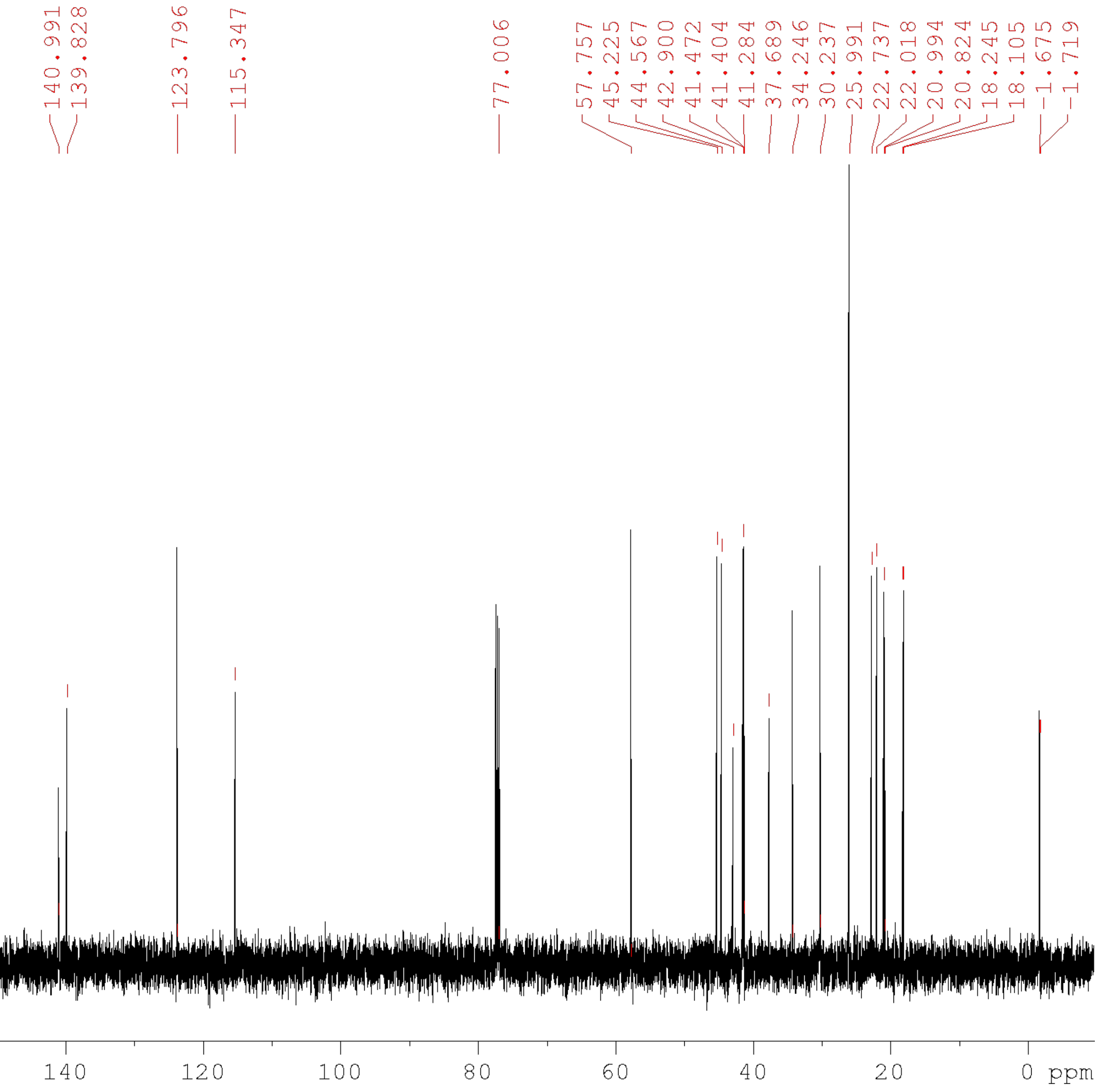

Current Data Parameters
NAME JC 5-069 superH
EXPNO 2
PROCNO 1
F2 - Acquisition Parameters
Date_ 20210630
Time 18.32
INSTRUM cryo500
PROBHD 5 mm CPTCI 1H-
PULPROG SpinEchopg30gp2.prd
TD 65536
SOLVENT CDCl3
NS 11
DS 16
SWH 30303.031 Hz
FIDRES 0.462388 Hz
AQ 1.0813440 sec
RG 5792.6
DW 16.500 usec
DE 6.00 usec
TE 298.0 K
D1 0.25000000 sec
d11 0.03000000 sec
D16 0.00020000 sec
d17 0.00019600 sec
MCREST 0 sec
MCWRK 0.01500000 sec
P2 37.70 usec
======== CHANNEL f1 ========
NUC1 13C
P1 18.85 usec
P12 2000.00 usec
P20 500.00 usec
PL0 120.00 dB
PL1 -1.00 dB
SFO1 125.7942548 MHz
SP2 1.55 dB
SP4 1.55 dB
SPNAM[2] Crp60comp.4
SPNAM[4] Crp60,0.5,20.1
SPOFF2 0 Hz
SPOFF4 0 Hz
======== CHANNEL f2 ========
CPDPRG[2 waltz16
NUC2 1H
PCPD2 100.00 usec
PL2 1.60 dB
PL12 22.00 dB
SFO2 500.2225011 MHz
====== GRADIENT CHANNEL =====
GPNAM[1] SINE.100
GPNAM[2] SINE.100
GPX1 0 %
GPX2 0 %
GPY1 0 %
GPY2 0 %
GPZ1 30.00 %
GPZ2 50.00 %
p15 500.00 usec
p16 1000.00 usec
F2 - Processing parameters
SI 65536
SF 125.7804050 MHz
WDW EM
SSB 0
LB 1.00 Hz
GB 0
PC 2.00
140.991
139.828
123.796
115.347
77.006
57.757
45.225
44.567
42.900
41.472
41.404
41.284
37.689
34.246
30.237
25.991
22.737
22.018
20.994
20.824
18.245
18.105
-1.675
-1.719
200
180
160
140
120
100
80
60
40
20
0 ppm

```
Current Data Parameters
NAME        JC 5-054 diol
EXPNO                   1
PROCNO                  1

F2 - Acquisition Parameters
Date_           20210708
Time               19.05
INSTRUM          cryo500
PROBHD   5 mm CPTCI 1H-
PULPROG             zg30
TD                 16022
SOLVENT            CDCl3
NS                     8
DS                     2
SWH             8012.820 Hz
FIDRES          0.500114 Hz
AQ             0.9997728 sec
RG                     8
DW                62.400 usec
DE                  6.00 usec
TE                 298.0 K
D1            0.10000000 sec
MCREST   0 sec
MCWRK         0.01500000 sec

======== CHANNEL f1 ========
NUC1                  1H
P1                  9.75 usec
PL1                 1.60 dB
SFO1         500.2235015 MHz

F2 - Processing parameters
SI                 65536
SF           500.2200312 MHz
WDW                   EM
SSB      0
LB                  0.30 Hz
GB       0
PC                  2.00
```

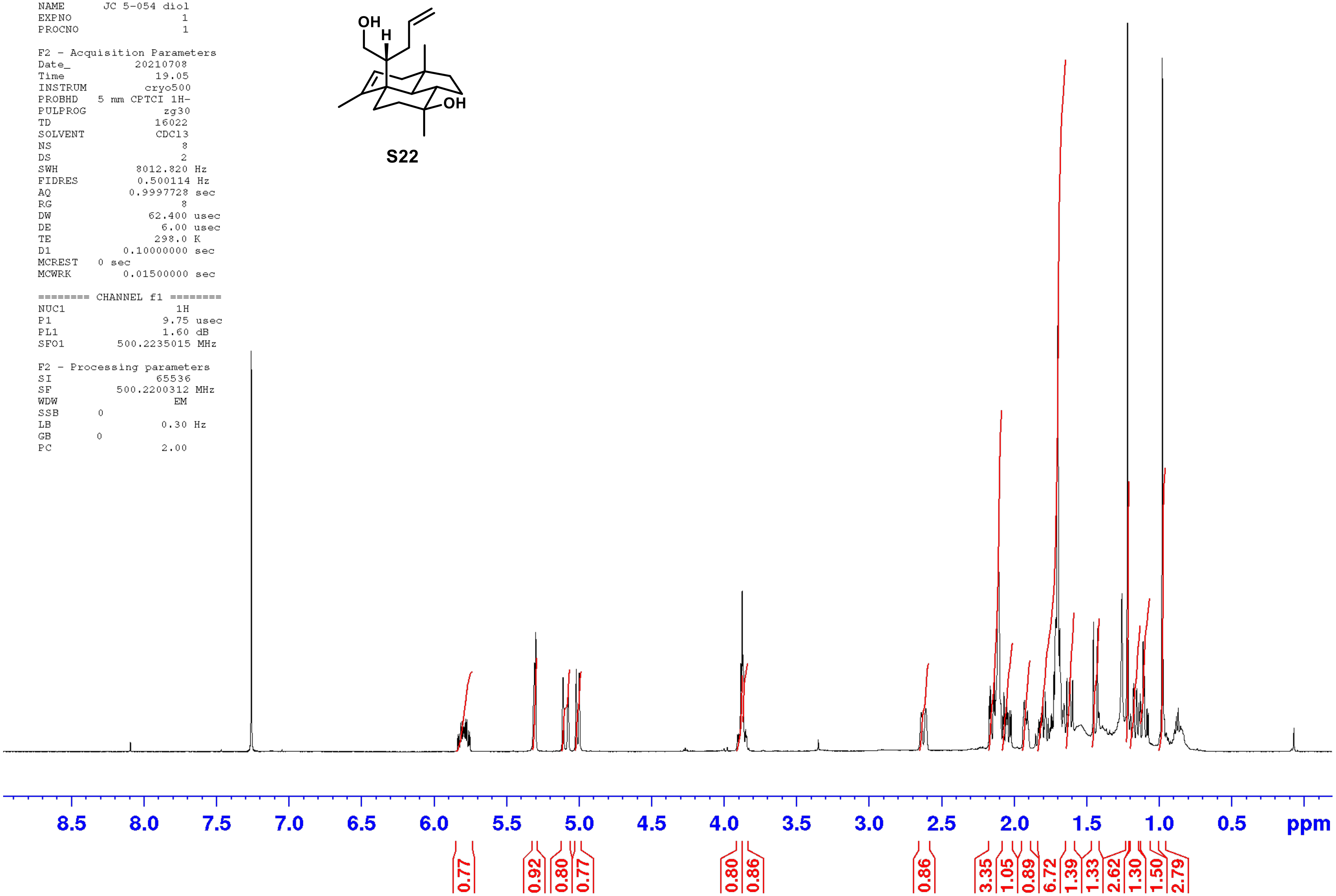

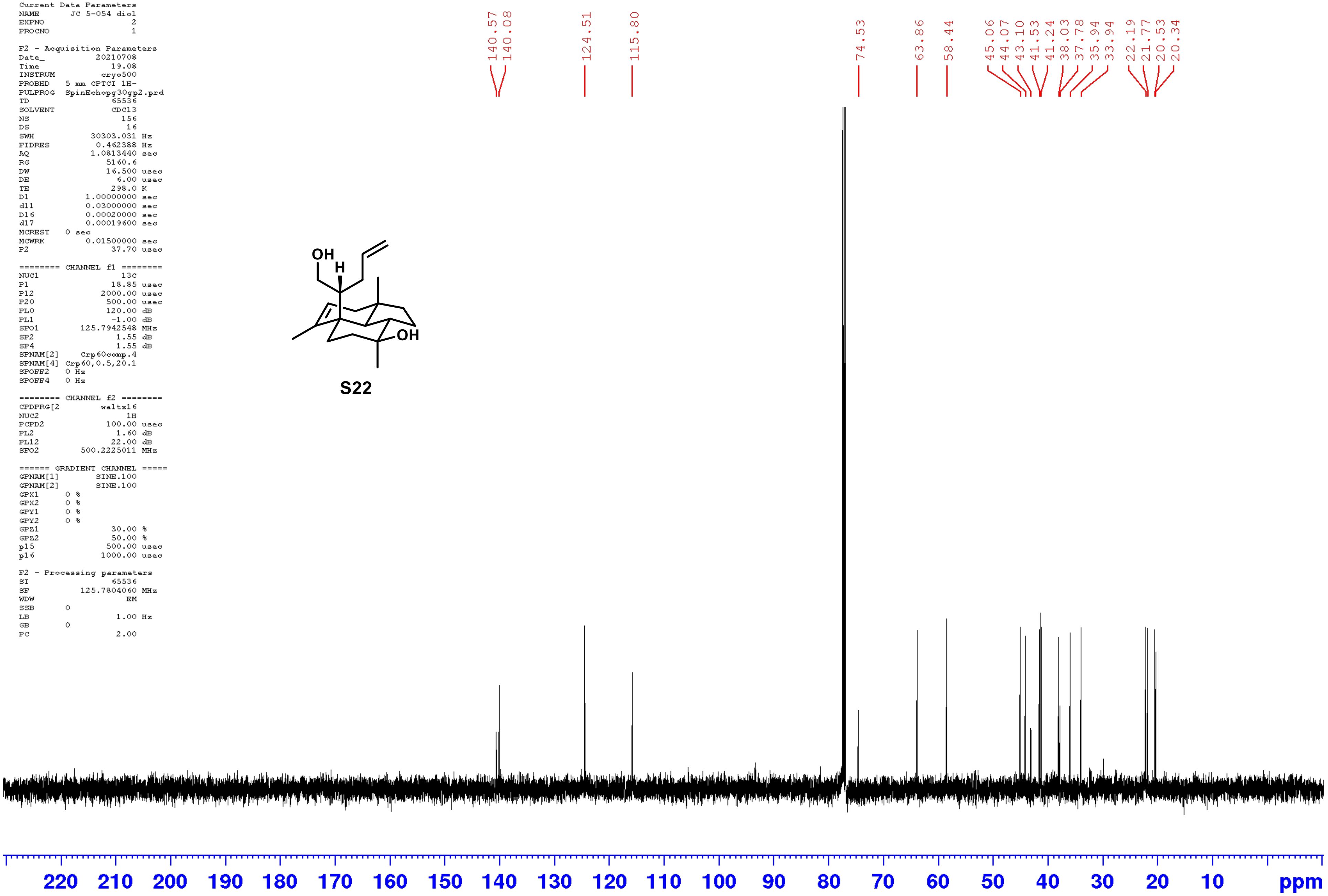
Current Data Parameters
NAME JC 5-054 diol
EXPNO 2
PROCNO 1
F2 - Acquisition Parameters
Date_ 20210708
Time 19.08
INSTRUM cryo500
PROBHD 5 mm CPTCI 1H-
PULPROG SpinEchopg30gp2.prd
TD 65536
SOLVENT CDCl3
NS 156
DS 16
SWH 30303.031 Hz
FIDRES 0.462388 Hz
AQ 1.0813440 sec
RG 5160.6
DW 16.500 usec
DE 6.00 usec
TE 298.0 K
D1 1.00000000 sec
d11 0.03000000 sec
D16 0.00020000 sec
d17 0.00019600 sec
MCREST 0 sec
MCWRK 0.01500000 sec
P2 37.70 usec
======== CHANNEL f1 ========
NUC1 13C
P1 18.85 usec
P12 2000.00 usec
P20 500.00 usec
PL0 120.00 dB
PL1 -1.00 dB
SFO1 125.7942548 MHz
SP2 1.55 dB
SP4 1.55 dB
SPNAM[2] Crp60comp.4
SPNAM[4] Crp60,0.5,20.1
SPOFF2 0 Hz
SPOFF4 0 Hz
======== CHANNEL f2 ========
CPDPRG[2 waltz16
NUC2 1H
PCPD2 100.00 usec
PL2 1.60 dB
PL12 22.00 dB
SFO2 500.2225011 MHz
====== GRADIENT CHANNEL =====
GPNAM[1] SINE.100
GPNAM[2] SINE.100
GPX1 0 %
GPX2 0 %
GPY1 0 %
GPY2 0 %
GPZ1 30.00 %
GPZ2 50.00 %
p15 500.00 usec
p16 1000.00 usec
F2 - Processing parameters
SI 65536
SF 125.7804060 MHz
WDW EM
SSB 0
LB 1.00 Hz
GB 0
PC 2.00
140.57
140.08
124.51
115.80
74.53
63.86
58.44
45.06
44.07
43.10
41.53
41.24
38.03
37.78
35.94
33.94
22.19
21.77
20.53
20.34
220 210 200 190 180 170 160 150 140 130 120 110 100 90 80 70 60 50 40 30 20 10 ppm

S22

Current Data Parameters
NAME JSCIV-292-f4-13
EXPNO 1
PROCNO 1

F2 - Acquisition Parameters
Date_ 20211208
Time 17.16
INSTRUM av600
PROBHD 5 mm CPBBO BB-
PULPROG zg30
TD 48074
SOLVENT CDCl3
NS 8
DS 2
SWH 7796.258 Hz
FIDRES 0.162172 Hz
AQ 3.0831459 sec
RG 8
DW 64.133 usec
DE 10.57 usec
TE 298.0 K
D1 4.00000000 sec
TD0 1

======== CHANNEL f1 ========
SFO1 600.1336008 MHz
NUC1 1H
P1 9.50 usec
PLW1 30.00000000 W

F2 - Processing parameters
SI 65536
SF 600.1300348 MHz
WDW EM
SSB 0
LB 0.30 Hz
GB 0
PC 1.00

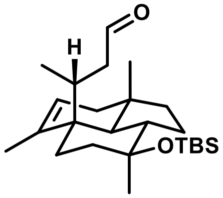

72

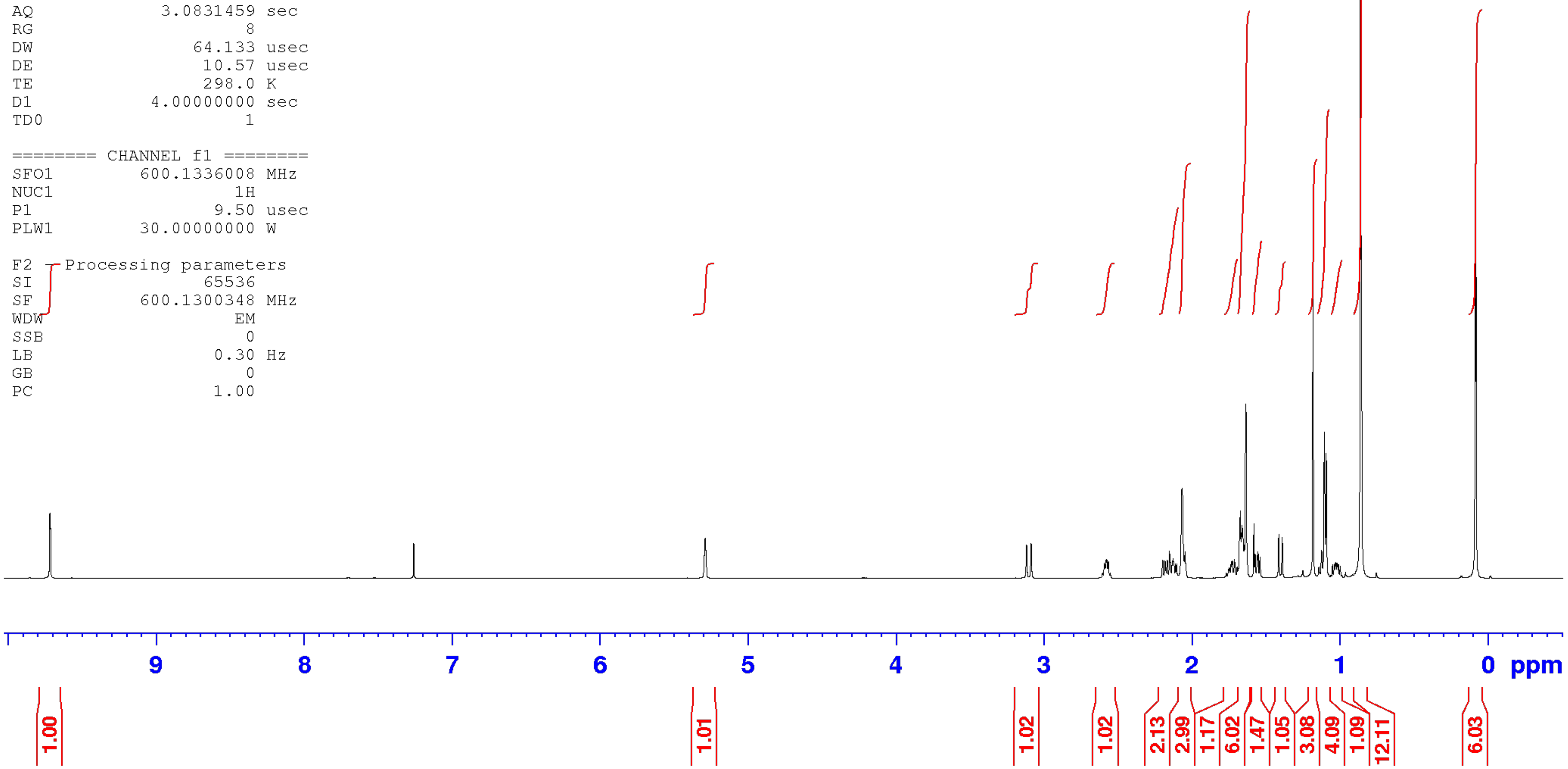

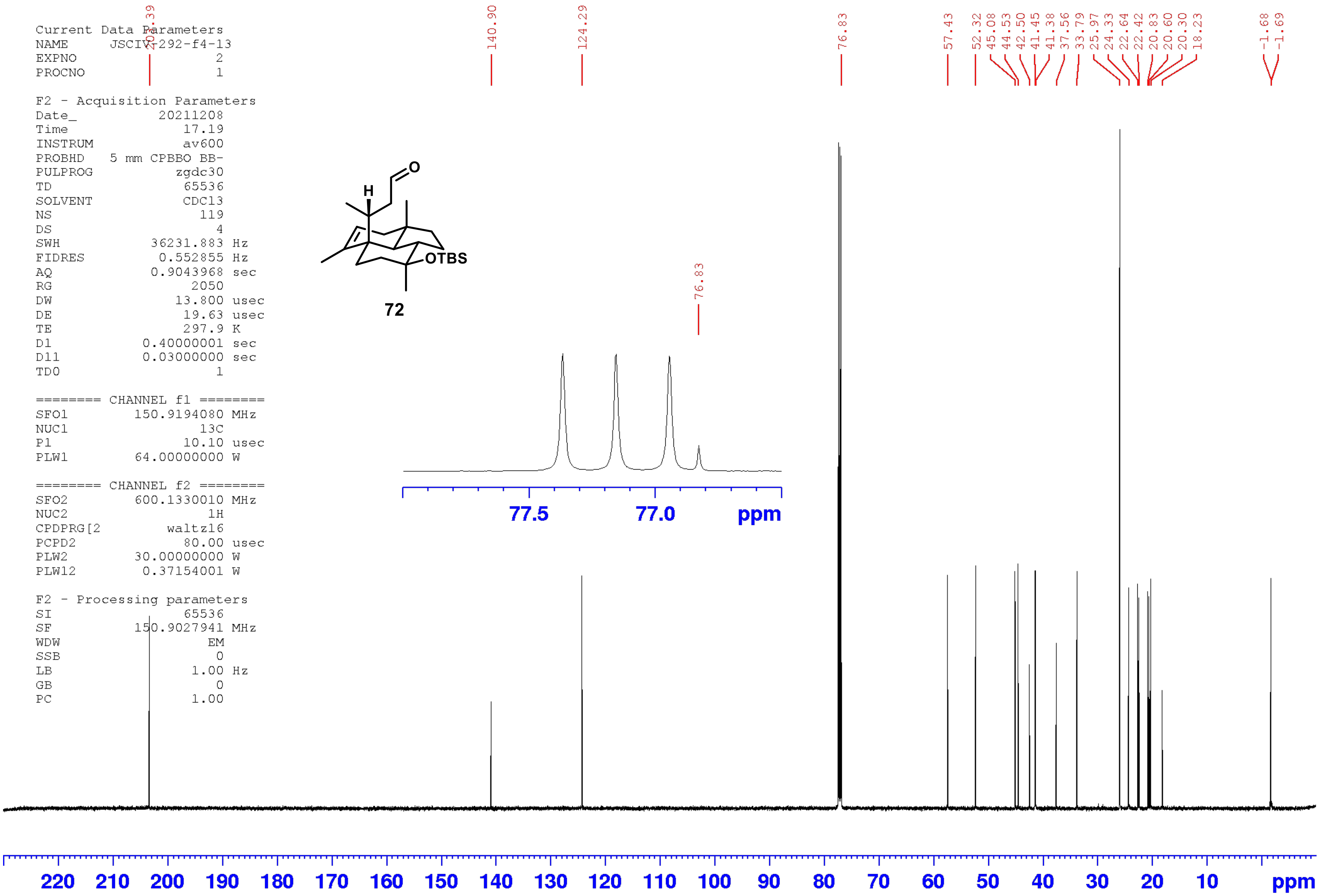
Current Data Parameters
NAME JSCIV-292-f4-13
EXPNO 2
PROCNO 1
F2 - Acquisition Parameters
Date_ 20211208
Time 17.19
INSTRUM av600
PROBHD 5 mm CPBBO BB-
PULPROG zgdc30
TD 65536
SOLVENT CDCl3
NS 119
DS 4
SWH 36231.883 Hz
FIDRES 0.552855 Hz
AQ 0.9043968 sec
RG 2050
DW 13.800 usec
DE 19.63 usec
TE 297.9 K
D1 0.40000001 sec
D11 0.03000000 sec
TD0 1
======== CHANNEL f1 ========
SFO1 150.9194080 MHz
NUC1 13C
P1 10.10 usec
PLW1 64.00000000 W
======== CHANNEL f2 ========
SFO2 600.1330010 MHz
NUC2 1H
CPDPRG[2 waltz16
PCPD2 80.00 usec
PLW2 30.00000000 W
PLW12 0.37154001 W
F2 - Processing parameters
SI 65536
SF 150.9027941 MHz
WDW EM
SSB 0
LB 1.00 Hz
GB 0
PC 1.00
140.90
124.29
76.83
57.43
52.32
45.08
44.53
42.50
41.45
41.38
37.56
33.79
25.97
24.33
22.64
22.42
20.83
20.60
20.30
18.23
-1.68
-1.69
O
H
OTBS
72
76.83
77.5
77.0
ppm
220 210 200 190 180 170 160 150 140 130 120 110 100 90 80 70 60 50 40 30 20 10
ppm

```
Current Data Parameters
NAME        JSCV-094-f3-8
EXPNO                   1
PROCNO                  1

F2 - Acquisition Parameters
Date_            20220304
Time                13.58
INSTRUM             gn500
PROBHD    5 mm broadband
PULPROG              zg30
TD                  40062
SOLVENT             CDCl3
NS                     16
DS                      2
SWH              7002.801 Hz
FIDRES           0.174799 Hz
AQ              2.8604269 sec
RG                    128
DW                 71.400 usec
DE                   6.00 usec
TE                  298.0 K
D1             3.00000000 sec
MCREST                  0 sec
MCWRK          0.01500000 sec

======== CHANNEL f1 ========
NUC1                   1H
P1                  12.00 usec
PL1                 -6.00 dB
SFO1          498.6529919 MHz

F2 - Processing parameters
SI                  65536
SF            498.6500284 MHz
WDW                    EM
SSB                     0
LB                   0.30 Hz
GB                      0
PC                   1.00
```

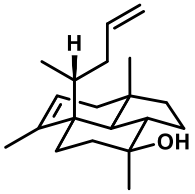

S23

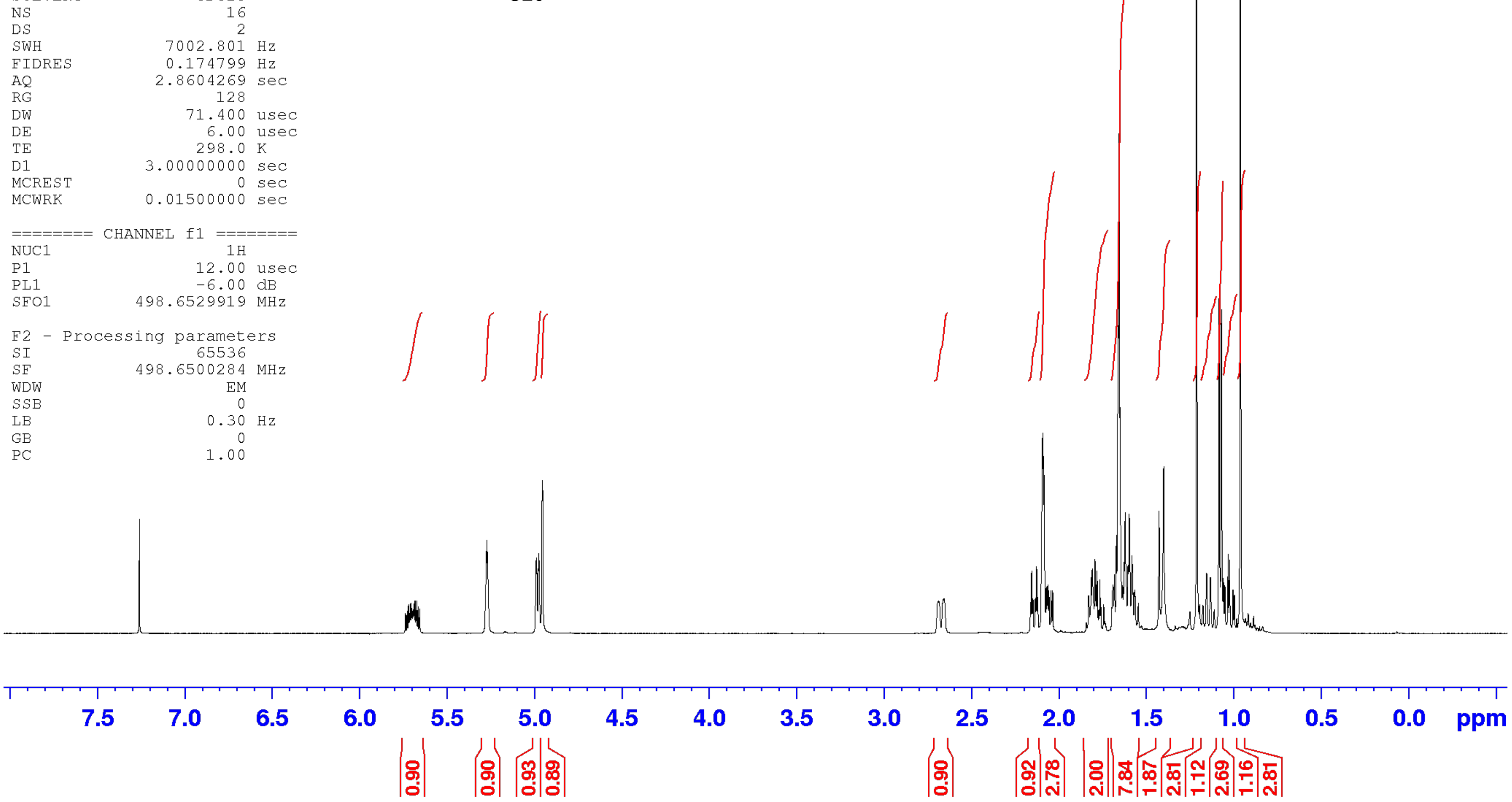

Current Data Parameters
NAME JSCV-094-f3-8
EXPNO 2
PROCNO 1

F2 - Acquisition Parameters
Date_ 20220304
Time 14.02
INSTRUM gn500
PROBHD 5 mm broadband
PULPROG zgdc30
TD 65536
SOLVENT CDCl3
NS 126
DS 4
SWH 30303.031 Hz
FIDRES 0.462388 Hz
AQ 1.0813440 sec
RG 32768
DW 16.500 usec
DE 6.00 usec
TE 297.9 K
D1 0.25000000 sec
d11 0.03000000 sec
MCREST 0 sec
MCWRK 0.01500000 sec

======== CHANNEL f1 ========
NUC1 13C
P1 14.20 usec
PL1 -6.00 dB
SFO1 125.3994349 MHz

======== CHANNEL f2 ========
CPDPRG[2 waltz16
NUC2 1H
PCPD2 100.00 usec
PL2 -6.00 dB
PL12 12.30 dB
SFO2 498.6524933 MHz

F2 - Processing parameters
SI 65536
SF 125.3856296 MHz
WDW EM
SSB 0
LB 0 Hz
GB 0
PC 2.00

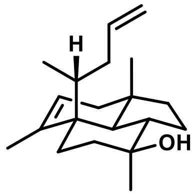

S23

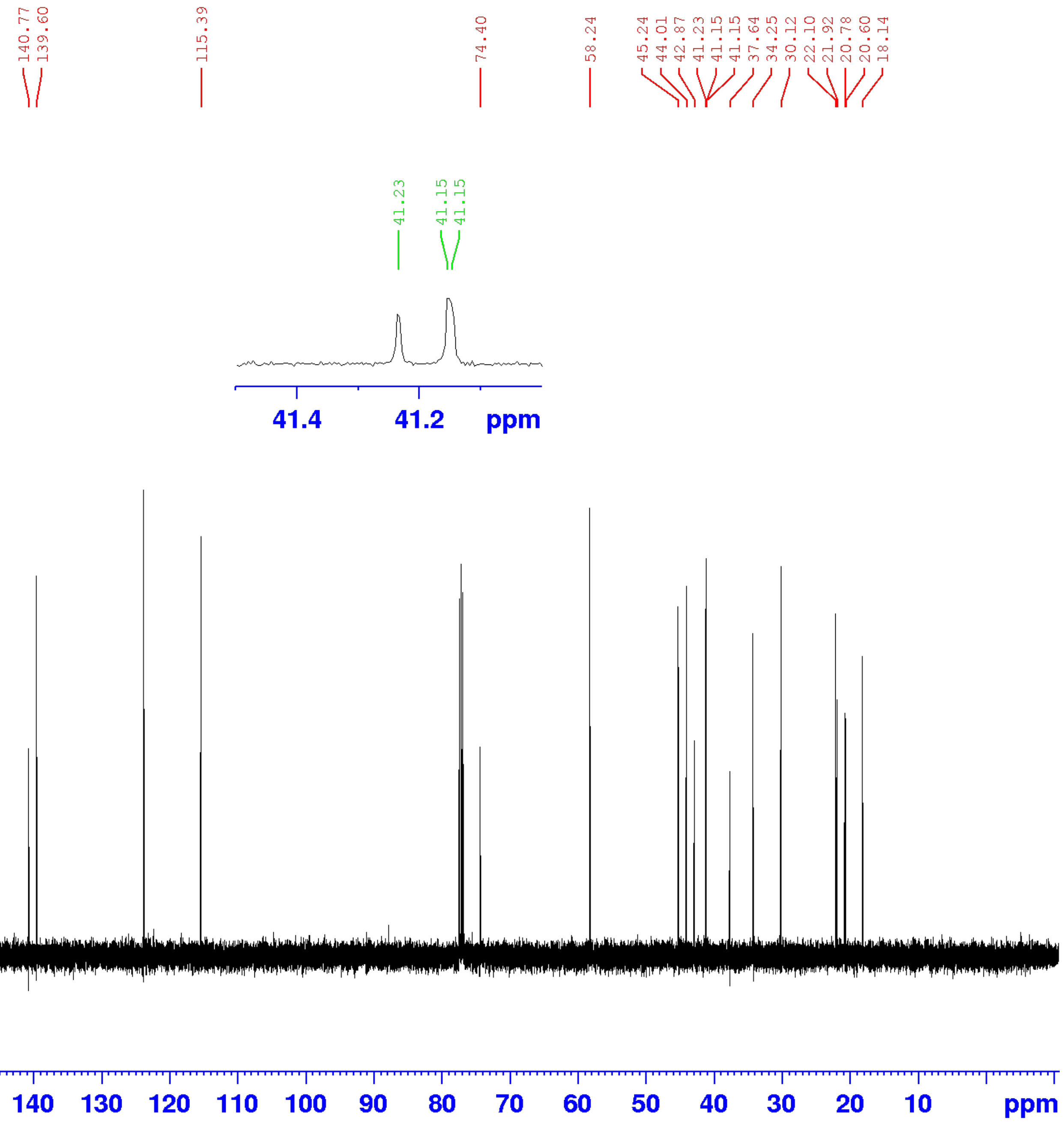

Current Data Parameters
NAME JSCV-047-f8-21
EXPNO 1
PROCNO 1

F2 - Acquisition Parameters
Date_ 20220125
Time 17.30
INSTRUM av600
PROBHD 5 mm CPBBO BB-
PULPROG zg30
TD 48074
SOLVENT CDCl3
NS 16
DS 2
SWH 9615.385 Hz
FIDRES 0.200012 Hz
AQ 2.4998479 sec
RG 10
DW 52.000 usec
DE 14.23 usec
TE 298.0 K
D1 3.00000000 sec
TD0 1

======== CHANNEL f1 ========
SFO1 600.1342009 MHz
NUC1 1H
P1 9.50 usec
PLW1 30.00000000 W

F2 - Processing parameters
SI 65536
SF 600.1300344 MHz
WDW EM
SSB 0
LB 0.30 Hz
GB 0
PC 1.00

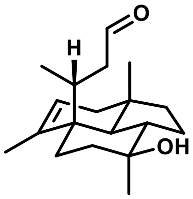

73

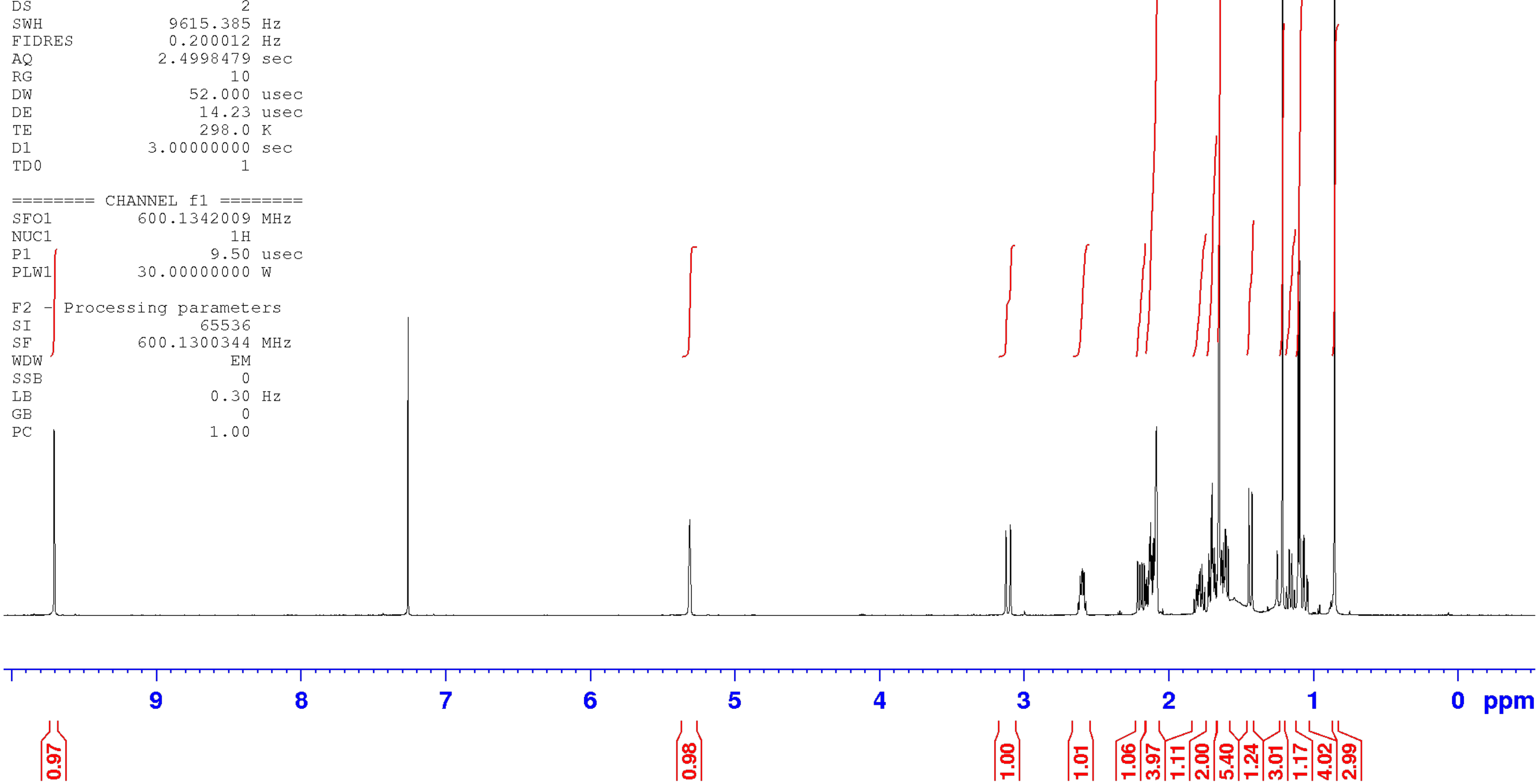

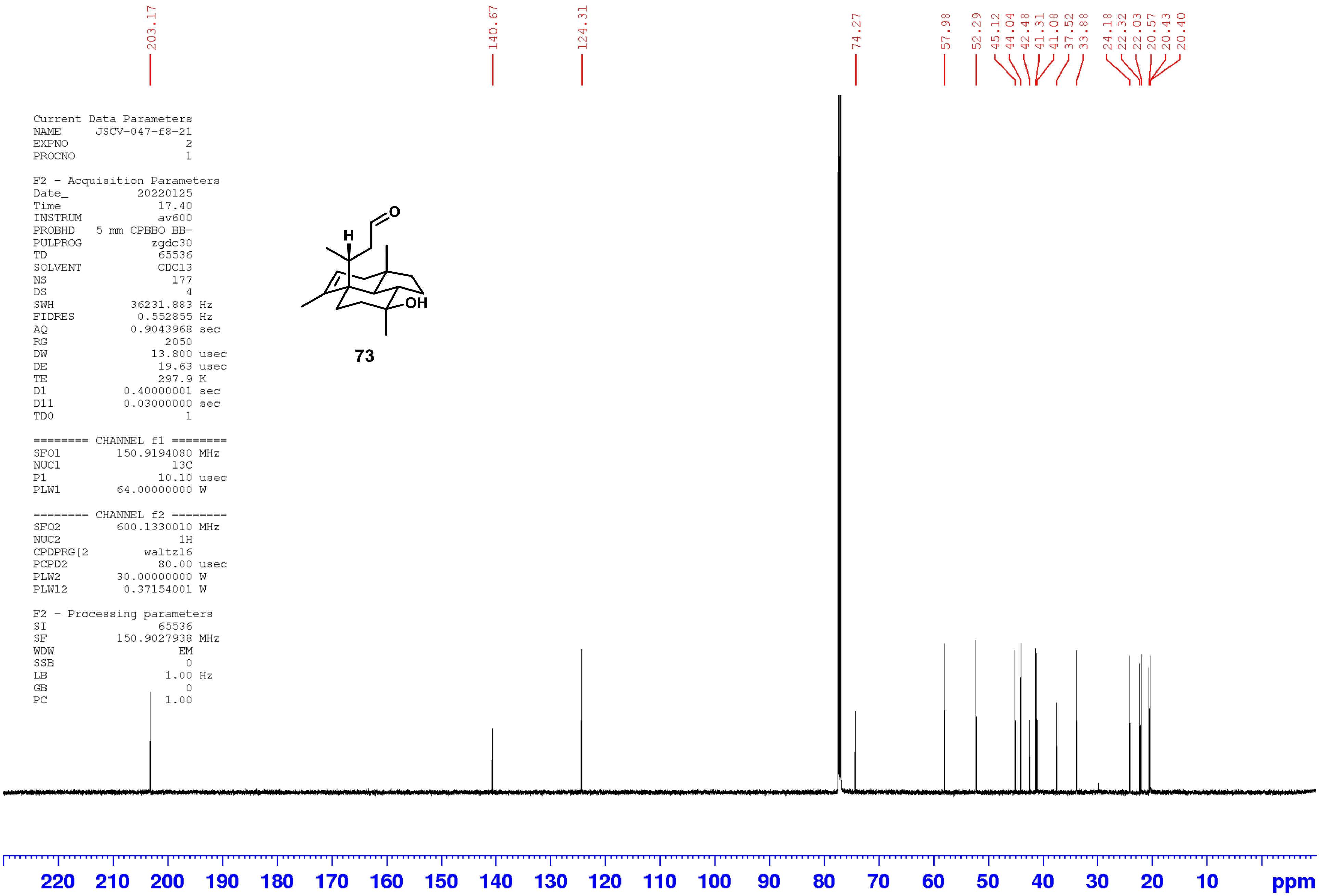
203.17
140.67
124.31
74.27
57.98
52.29
45.12
44.04
42.48
41.31
41.08
37.52
33.88
24.18
22.32
22.03
20.57
20.43
20.40
Current Data Parameters
NAME JSCV-047-f8-21
EXPNO 2
PROCNO 1
F2 - Acquisition Parameters
Date_ 20220125
Time 17.40
INSTRUM av600
PROBHD 5 mm CPBBO BB-
PULPROG zgdc30
TD 65536
SOLVENT CDCl3
NS 177
DS 4
SWH 36231.883 Hz
FIDRES 0.552855 Hz
AQ 0.9043968 sec
RG 2050
DW 13.800 usec
DE 19.63 usec
TE 297.9 K
D1 0.40000001 sec
D11 0.03000000 sec
TD0 1
======== CHANNEL f1 ========
SFO1 150.9194080 MHz
NUC1 13C
P1 10.10 usec
PLW1 64.00000000 W
======== CHANNEL f2 ========
SFO2 600.1330010 MHz
NUC2 1H
CPDPRG[2 waltz16
PCPD2 80.00 usec
PLW2 30.00000000 W
PLW12 0.37154001 W
F2 - Processing parameters
SI 65536
SF 150.9027938 MHz
WDW EM
SSB 0
LB 1.00 Hz
GB 0
PC 1.00
O
H
OH
73
220 210 200 190 180 170 160 150 140 130 120 110 100 90 80 70 60 50 40 30 20 10 ppm

Current Data Parameters
NAME JC 5-071 cr
EXPNO 1
PROCNO 1

F2 - Acquisition Parameters
Date_ 20210624
Time 3.27
INSTRUM cryo500
PROBHD 5 mm CPTCI 1H-
PULPROG zg30
TD 16022
SOLVENT CDCl3
NS 8
DS 2
SWH 8012.820 Hz
FIDRES 0.500114 Hz
AQ 0.9997728 sec
RG 8
DW 62.400 usec
DE 6.00 usec
TE 298.0 K
D1 0.10000000 sec
MCREST 0 sec
MCWRK 0.01500000 sec

======== CHANNEL f1 ========
NUC1 1H
P1 9.75 usec
PL1 1.60 dB
SFO1 500.2235015 MHz

F2 - Processing parameters
SI 65536
SF 500.2200314 MHz
WDW EM
SSB 0
LB 0.30 Hz
GB 0
PC 1.00

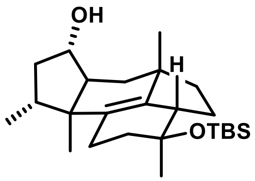

74

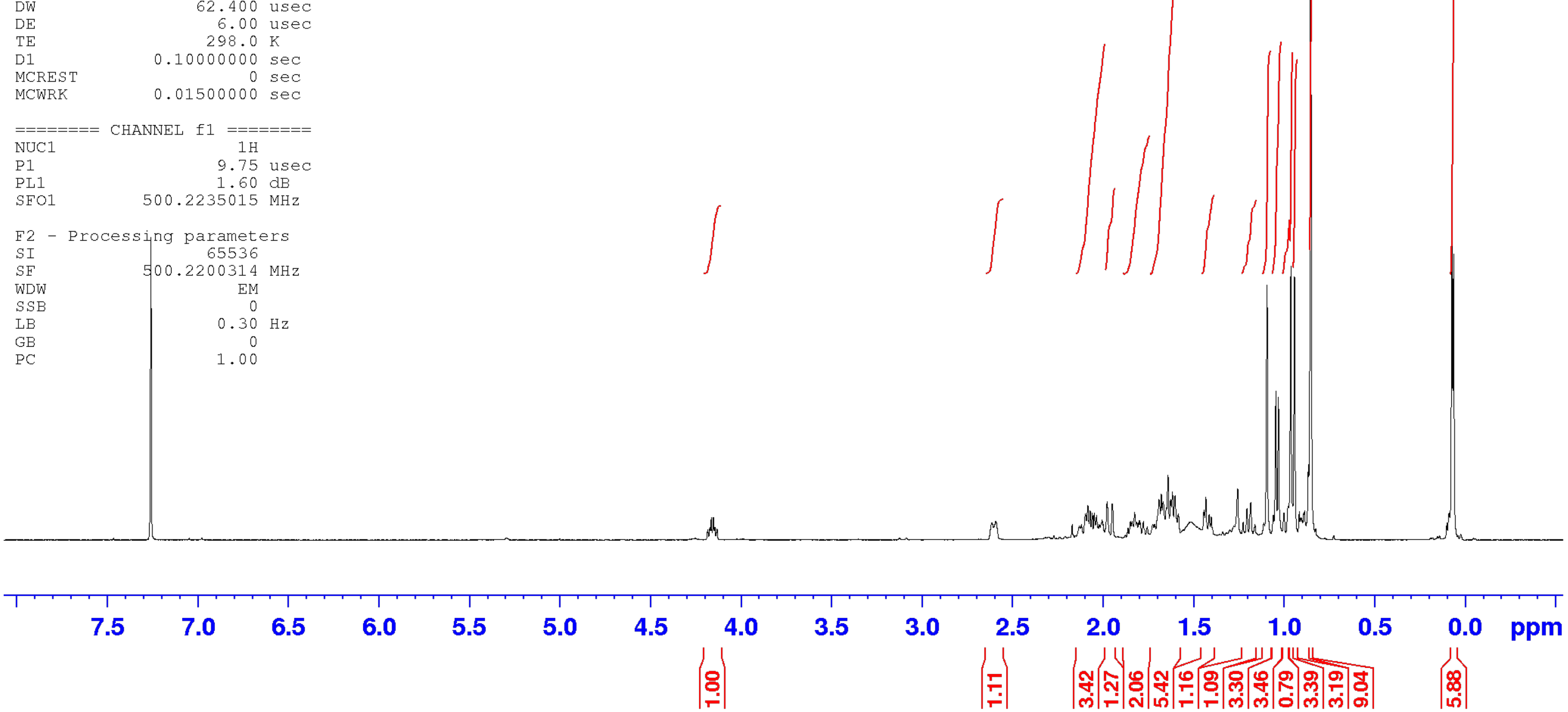

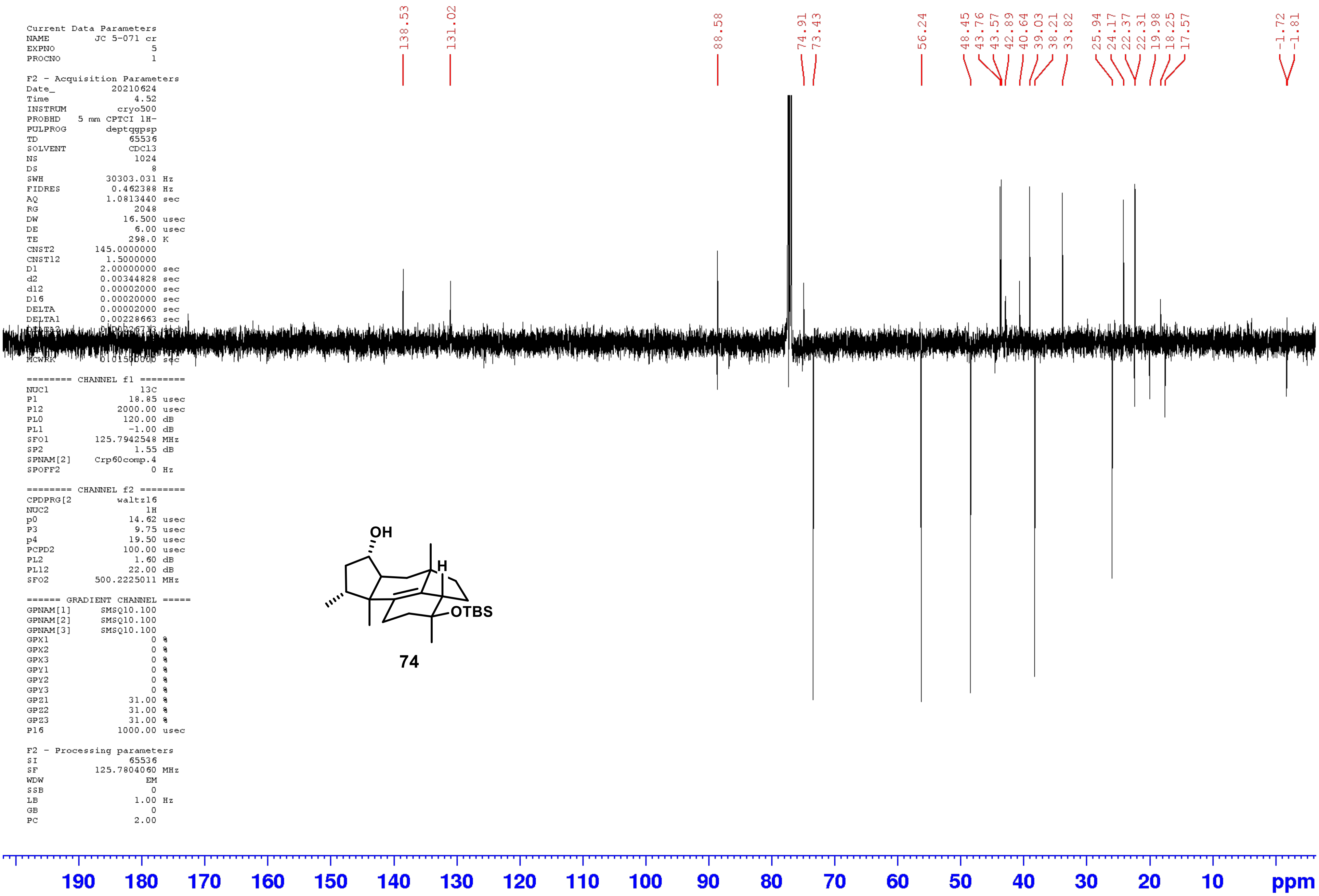

Current Data Parameters
NAME JC 5-071 cr
EXPNO 5
PROCNO 1
F2 - Acquisition Parameters
Date_ 20210624
Time 4.52
INSTRUM cryo500
PROBHD 5 mm CPTCI 1H-
PULPROG deptqgpsp
TD 65536
SOLVENT CDCl3
NS 1024
DS 8
SWH 30303.031 Hz
FIDRES 0.462388 Hz
AQ 1.0813440 sec
RG 2048
DW 16.500 usec
DE 6.00 usec
TE 298.0 K
CNST2 145.0000000
CNST12 1.5000000
D1 2.00000000 sec
d2 0.00344828 sec
d12 0.00002000 sec
D16 0.00020000 sec
DELTA 0.00002000 sec
DELTA1 0.00228663 sec
======== CHANNEL f1 ========
NUC1 13C
P1 18.85 usec
P12 2000.00 usec
PL0 120.00 dB
PL1 -1.00 dB
SFO1 125.7942548 MHz
SP2 1.55 dB
SPNAM[2] Crp60comp.4
SPOFF2 0 Hz
======== CHANNEL f2 ========
CPDPRG[2 waltz16
NUC2 1H
p0 14.62 usec
P3 9.75 usec
p4 19.50 usec
PCPD2 100.00 usec
PL2 1.60 dB
PL12 22.00 dB
SFO2 500.2225011 MHz
====== GRADIENT CHANNEL =====
GPNAM[1] SMSQ10.100
GPNAM[2] SMSQ10.100
GPNAM[3] SMSQ10.100
GPX1 0 %
GPX2 0 %
GPX3 0 %
GPY1 0 %
GPY2 0 %
GPY3 0 %
GPZ1 31.00 %
GPZ2 31.00 %
GPZ3 31.00 %
P16 1000.00 usec
F2 - Processing parameters
SI 65536
SF 125.7804060 MHz
WDW EM
SSB 0
LB 1.00 Hz
GB 0
PC 2.00
138.53
131.02
88.58
74.91
73.43
56.24
48.45
43.76
43.57
42.89
40.64
39.03
38.21
33.82
25.94
24.17
22.37
22.31
19.98
18.25
17.57
-1.72
-1.81
OH
H
OTBS
74
190
180
170
160
150
140
130
120
110
100
90
80
70
60
50
40
30
20
10
ppm

```
Current Data Parameters
NAME      JC 5-072 uwave carbonylene cr
EXPNO                      1
PROCNO                     1

F2 - Acquisition Parameters
Date_           20210624
Time               19.04
INSTRUM          cryo500
PROBHD    5 mm CPTCI 1H-
PULPROG             zg30
TD                 16022
SOLVENT            CDCl3
NS                     8
DS                     2
SWH             8012.820 Hz
FIDRES          0.500114 Hz
AQ             0.9997728 sec
RG                   6.3
DW                62.400 usec
DE                  6.00 usec
TE                 298.0 K
D1            0.10000000 sec
MCREST                 0 sec
MCWRK         0.01500000 sec

======== CHANNEL f1 ========
NUC1                  1H
P1                  9.75 usec
PL1                 1.60 dB
SFO1         500.2235015 MHz

F2 - Processing parameters
SI                 65536
SF           500.2200311 MHz
WDW                   EM
SSB                    0
LB                  0.30 Hz
GB                     0
PC                  1.00
```

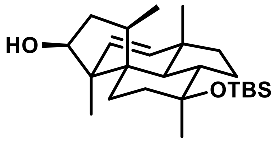

75

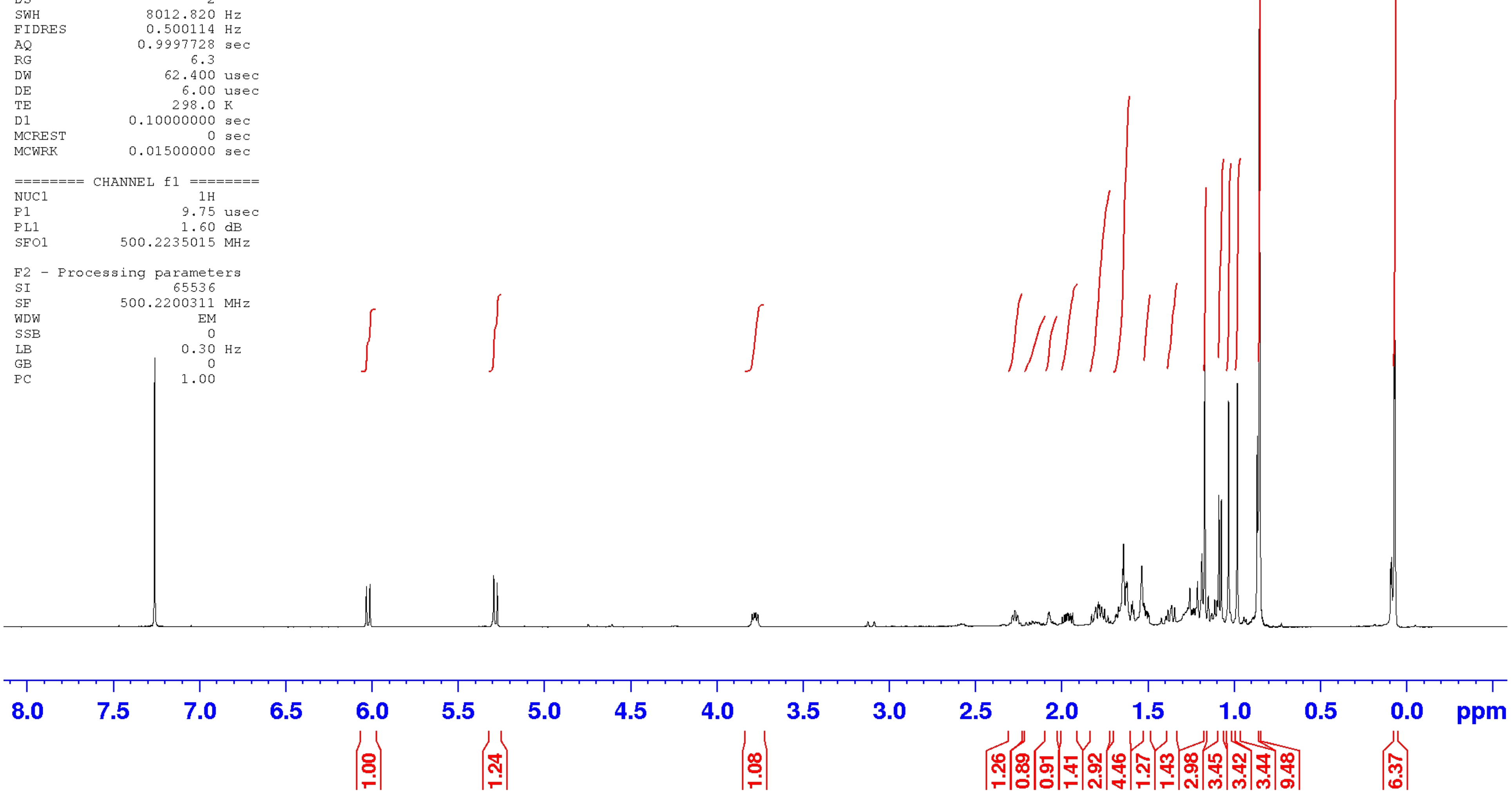

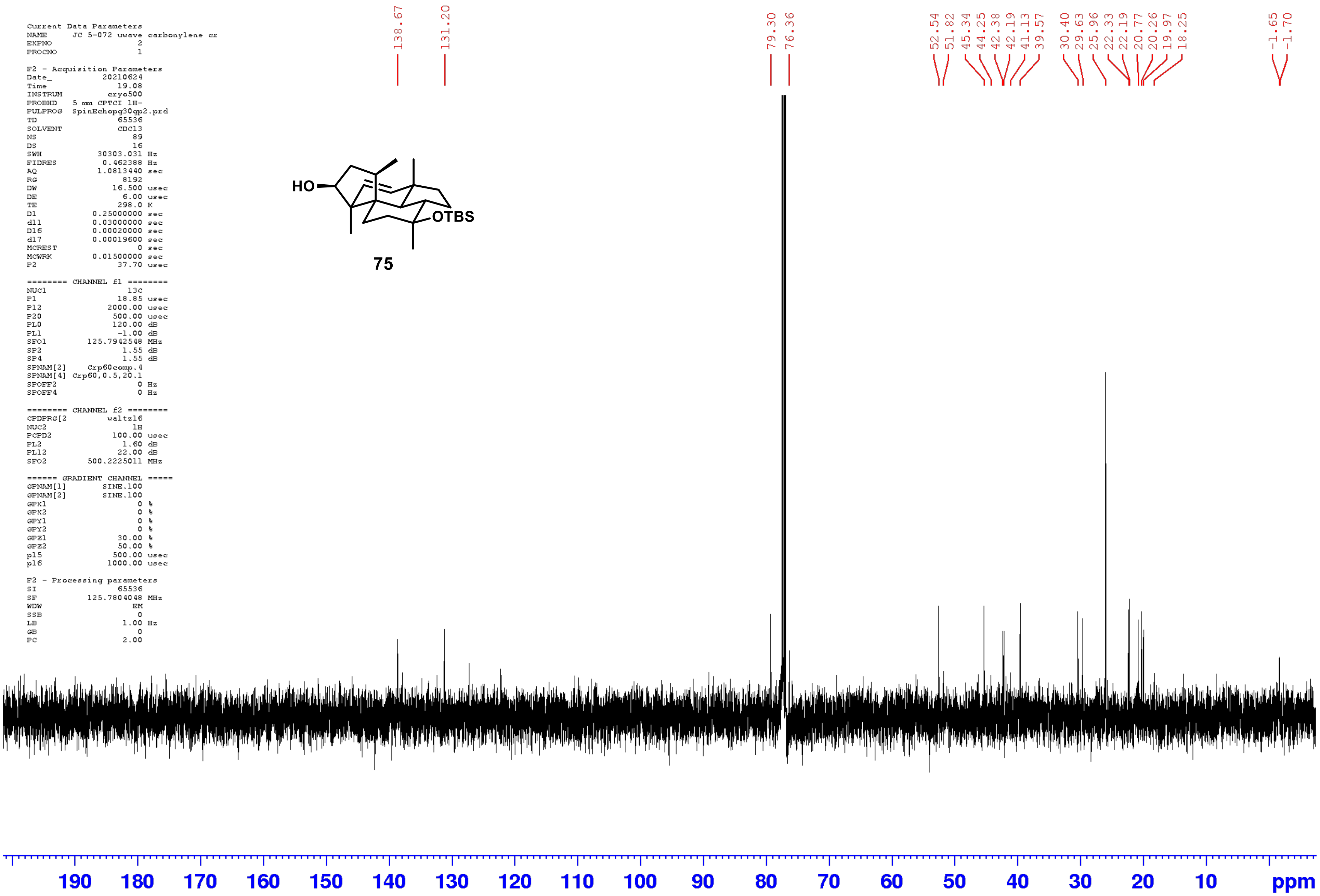
Current Data Parameters
NAME JC 5-072 uwave carbonylene cr
EXPNO 2
PROCNO 1
F2 - Acquisition Parameters
Date_ 20210624
Time 19.08
INSTRUM cryo500
PROBHD 5 mm CPTCI 1H-
PULPROG SpinEchopg30gp2.prd
TD 65536
SOLVENT CDCl3
NS 89
DS 16
SWH 30303.031 Hz
FIDRES 0.462388 Hz
AQ 1.0813440 sec
RG 8192
DW 16.500 usec
DE 6.00 usec
TE 298.0 K
D1 0.25000000 sec
d11 0.03000000 sec
D16 0.00020000 sec
d17 0.00019600 sec
MCREST 0 sec
MCWRK 0.01500000 sec
P2 37.70 usec
======== CHANNEL f1 ========
NUC1 13C
P1 18.85 usec
P12 2000.00 usec
P20 500.00 usec
PL0 120.00 dB
PL1 -1.00 dB
SFO1 125.7942548 MHz
SP2 1.55 dB
SP4 1.55 dB
SPNAM[2] Crp60comp.4
SPNAM[4] Crp60,0.5,20.1
SPOFF2 0 Hz
SPOFF4 0 Hz
======== CHANNEL f2 ========
CPDPRG[2 waltz16
NUC2 1H
PCPD2 100.00 usec
PL2 1.60 dB
PL12 22.00 dB
SFO2 500.2225011 MHz
====== GRADIENT CHANNEL =====
GPNAM[1] SINE.100
GPNAM[2] SINE.100
GPX1 0 %
GPX2 0 %
GPY1 0 %
GPY2 0 %
GPZ1 30.00 %
GPZ2 50.00 %
p15 500.00 usec
p16 1000.00 usec
F2 - Processing parameters
SI 65536
SF 125.7804048 MHz
WDW EM
SSB 0
LB 1.00 Hz
GB 0
PC 2.00
HO
OTBS
75
138.67
131.20
79.30
76.36
52.54
51.82
45.34
44.25
42.38
42.19
41.13
39.57
30.40
29.63
25.96
22.33
22.19
20.77
20.26
19.97
18.25
-1.65
-1.70
190
180
170
160
150
140
130
120
110
100
90
80
70
60
50
40
30
20
10
ppm

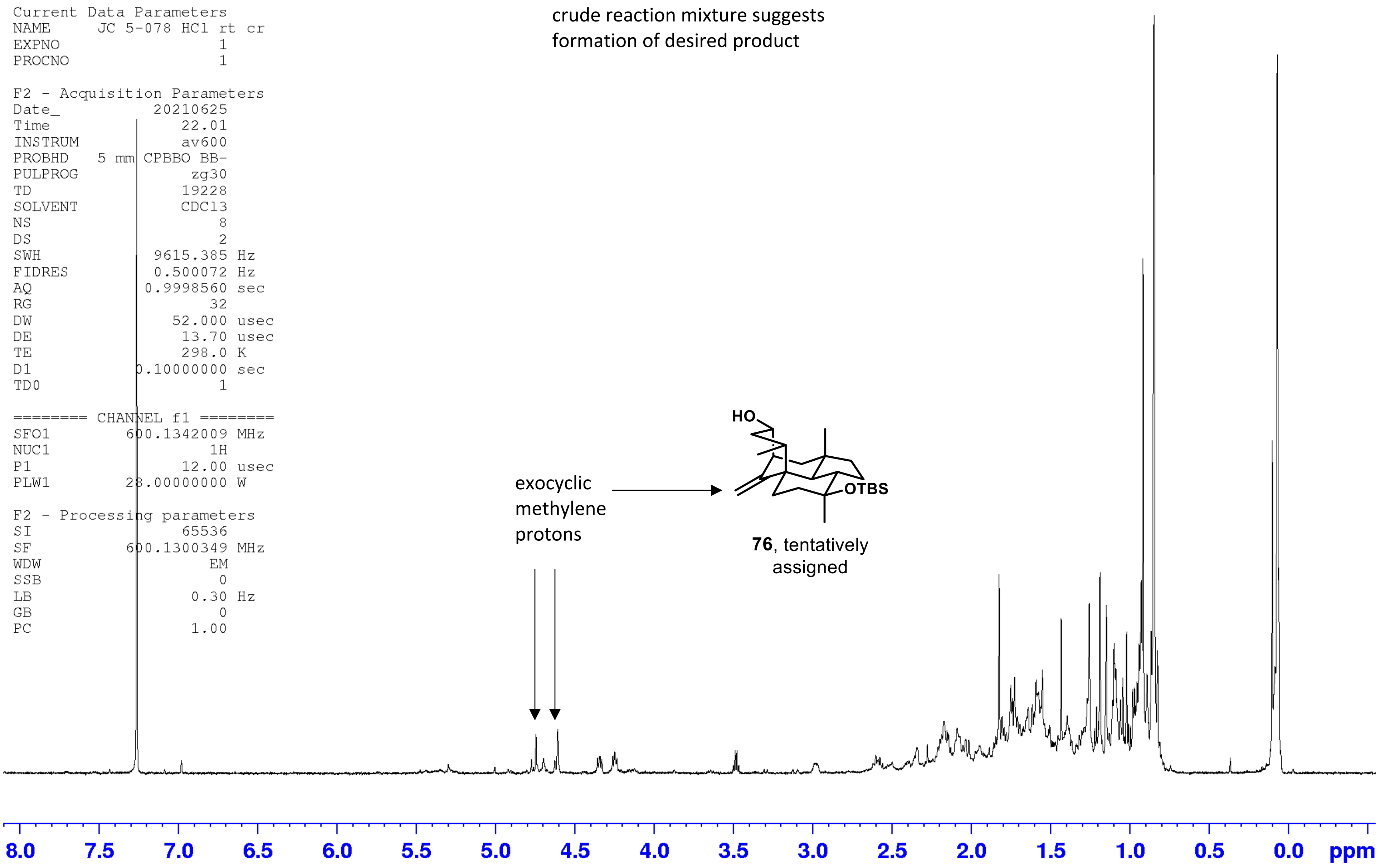
Current Data Parameters
NAME JC 5-078 HCl rt cr
EXPNO 1
PROCNO 1
F2 - Acquisition Parameters
Date_ 20210625
Time 22.01
INSTRUM av600
PROBHD 5 mm CPBBO BB-
PULPROG zg30
TD 19228
SOLVENT CDCl3
NS 8
DS 2
SWH 9615.385 Hz
FIDRES 0.500072 Hz
AQ 0.9998560 sec
RG 32
DW 52.000 usec
DE 13.70 usec
TE 298.0 K
D1 0.10000000 sec
TD0 1
======== CHANNEL f1 ========
SFO1 600.1342009 MHz
NUC1 1H
P1 12.00 usec
PLW1 28.00000000 W
F2 - Processing parameters
SI 65536
SF 600.1300349 MHz
WDW EM
SSB 0
LB 0.30 Hz
GB 0
PC 1.00
crude reaction mixture suggests
formation of desired product
HO
OTBS
exocyclic
methylene
protons
76, tentatively
assigned
8.0
7.5
7.0
6.5
6.0
5.5
5.0
4.5
4.0
3.5
3.0
2.5
2.0
1.5
1.0
0.5
0.0
ppm

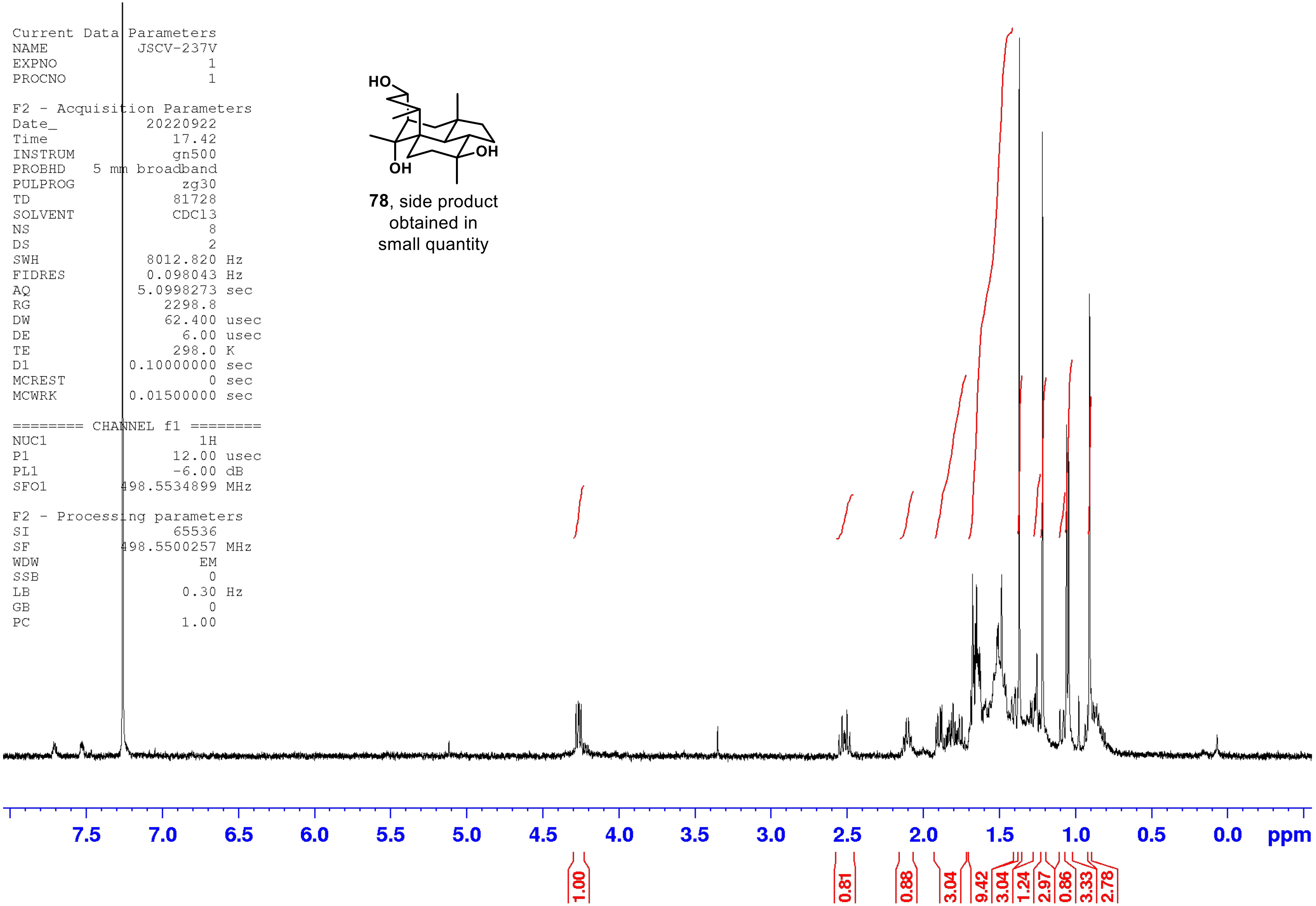
Current Data Parameters
NAME JSCV-237V
EXPNO 1
PROCNO 1
F2 - Acquisition Parameters
Date_ 20220922
Time 17.42
INSTRUM gn500
PROBHD 5 mm broadband
PULPROG zg30
TD 81728
SOLVENT CDCl3
NS 8
DS 2
SWH 8012.820 Hz
FIDRES 0.098043 Hz
AQ 5.0998273 sec
RG 2298.8
DW 62.400 usec
DE 6.00 usec
TE 298.0 K
D1 0.10000000 sec
MCREST 0 sec
MCWRK 0.01500000 sec
======== CHANNEL f1 ========
NUC1 1H
P1 12.00 usec
PL1 -6.00 dB
SFO1 498.5534899 MHz
F2 - Processing parameters
SI 65536
SF 498.5500257 MHz
WDW EM
SSB 0
LB 0.30 Hz
GB 0
PC 1.00
HO
OH
OH
78, side product obtained in small quantity
7.5
7.0
6.5
6.0
5.5
5.0
4.5
4.0
3.5
3.0
2.5
2.0
1.5
1.0
0.5
0.0
ppm
1.00
0.81
0.88
3.04
9.42
3.04
1.24
2.97
0.86
3.33
2.78

```
Current Data Parameters
NAME        JSCV-237V-2
EXPNO                 3
PROCNO                1

F2 - Acquisition Parameters
Date_          20220923
Time              18.38
INSTRUM           av600
PROBHD   5 mm CPTCI 1H-
PULPROG       deptqgpsp
TD                65536
SOLVENT           CDCl3
NS                  228
DS                    8
SWH           36231.883 Hz
FIDRES         0.552855 Hz
AQ            0.9043968 sec
RG                 2050
DW               13.800 usec
DE                18.00 usec
TE                298.0 K
CNST2       145.0000000
CNST12        1.5000000
D1           2.00000000 sec
D2           0.00344828 sec
D12          0.00002000 sec
D16          0.00020000 sec
TD0                   1

======== CHANNEL f1 ========
SFO1        150.9194080 MHz
NUC1                13C
P1                18.75 usec
P13             2000.00 usec
PLW0                  0 W
PLW1       164.00000000 W
SPNAM[5]    Crp60comp.4
[illegible]

======== CHANNEL f2 ========
SFO2        600.1330010 MHz
NUC2                 1H
CPDPRG[2        waltz16
P0                12.00 usec
P3                 8.00 usec
P4                16.00 usec
PCPD2             80.00 usec
PLW2         6.16599989 W
PLW12        0.04466800 W

====== GRADIENT CHANNEL =====
GPNAM[1]     SMSQ10.100
GPNAM[2]     SMSQ10.100
GPNAM[3]     SMSQ10.100
GPZ1              31.00 %
GPZ2              31.00 %
GPZ3              31.00 %
P16             1000.00 usec

F2 - Processing parameters
SI                65536
SF          150.9027935 MHz
WDW                  EM
SSB                   0
LB                 1.00 Hz
GB                    0
PC                 1.00
```

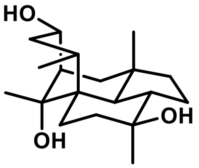

78, side product obtained in small quantity

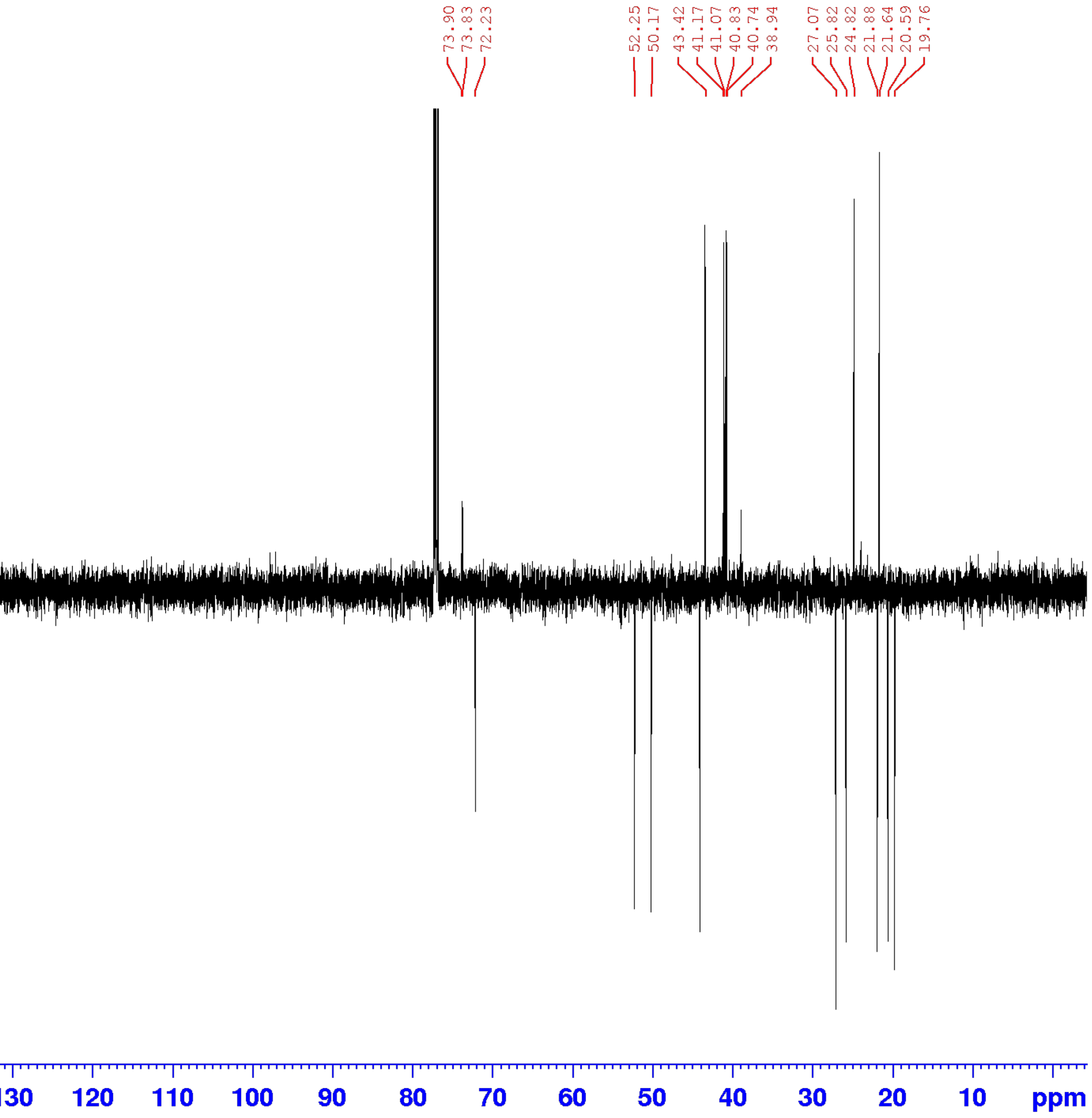

```
Current Data Parameters
NAME            JSCV-237V-2
EXPNO                     5
PROCNO                    1

F2 - Acquisition Parameters
Date_              20220923
Time                  18.53
INSTRUM               av600
PROBHD    5 mm CPTCI 1H-
PULPROG           cosygpqf
TD                     2048
SOLVENT               CDCl3
NS                        1
DS                       16
SWH               9615.385 Hz
FIDRES            4.695012 Hz
AQ               0.1064960 sec
RG                     2050
DW                   52.000 usec
DE                    10.00 usec
TE                    298.0 K
D0               0.00000300 sec
D1               1.48689198 sec
D13              0.00000400 sec
D16              0.00020000 sec
IN0              0.00010400 sec

======== CHANNEL f1 ========
SFO1          600.1342009 MHz
NUC1                     1H
P0                     8.00 usec
P1                     8.00 usec
PLW1             6.16599989 W

====== GRADIENT CHANNEL =====
GPNAM[1]        SMSQ10.100
GPZ1                  10.00 %
P16                 1000.00 usec

F1 - Acquisition parameters
TD                      256
SFO1               600.1342 MHz
FIDRES            75.120193 Hz
SW                   16.022 ppm
FnMODE                   QF

F2 - Processing parameters
SI                     1024
SF            600.1300323 MHz
WDW                    SINE
SSB                       0
LB                        0 Hz
GB                        0
PC                     1.00

F1 - Processing parameters
SI                     1024
MC2                      QF
SF            600.1300360 MHz
WDW                    SINE
SSB                       0
LB                        0 Hz
GB                        0
```

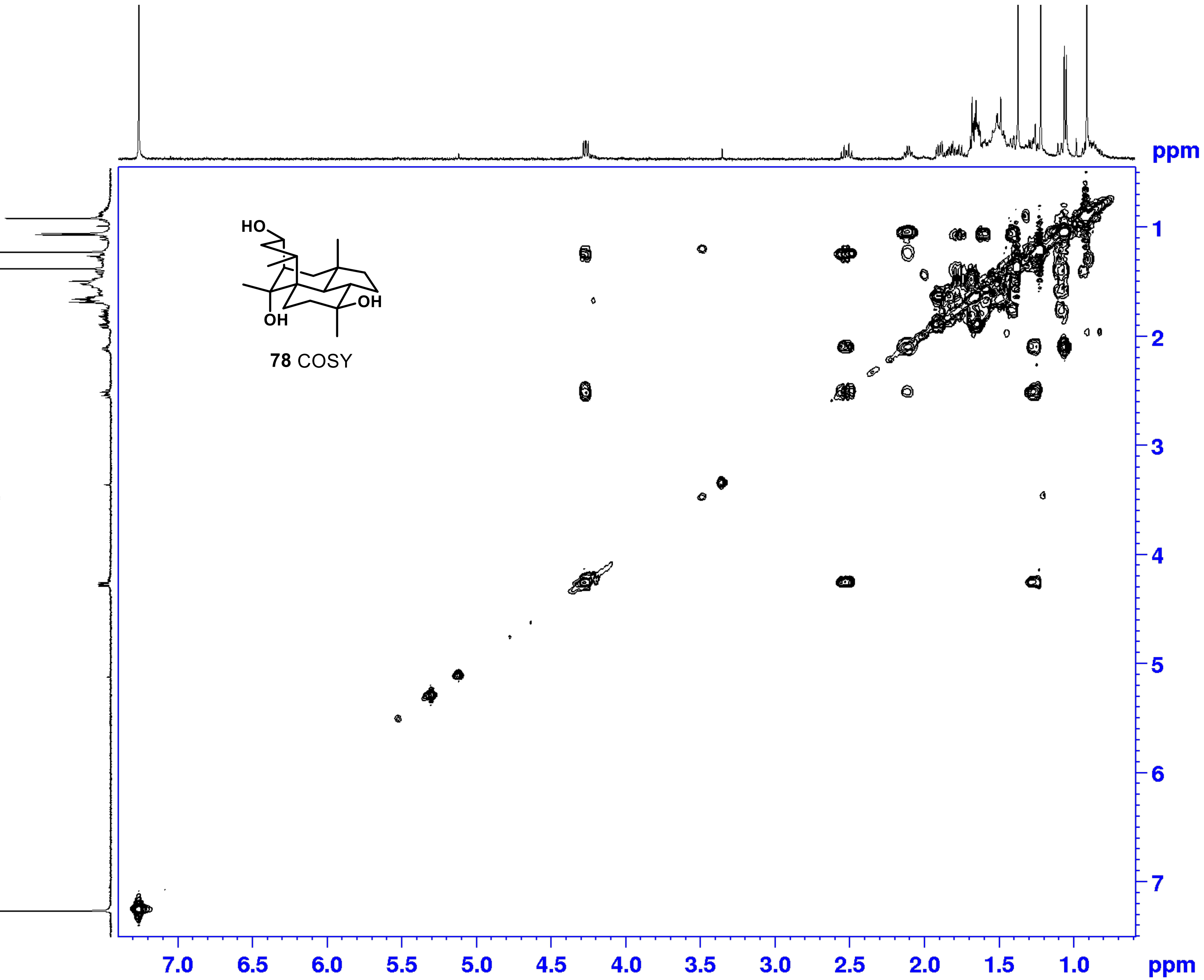

```
Current Data Parameters
NAME          JSCV-237V-2
EXPNO                   4
PROCNO                  1

F2 - Acquisition Parameters
Date_            20220923
Time                18.43
INSTRUM             av600
PROBHD   5 mm CPTCI 1H-
PULPROG     hsqcetgpsp.2
TD                   1024
SOLVENT             CDCl3
NS                      2
DS                     16
SWH             9615.385 Hz
FIDRES          9.390024 Hz
AQ             0.0532480 sec
RG                   2050
DW                 52.000 usec
DE                  10.00 usec
TE                  297.9 K
CNST2         145.0000000
D0             0.00000300 sec
D1             1.10000002 sec
D4             0.00172414 sec
D11            0.03000000 sec
D16            0.00020000 sec
IN0            0.00001380 sec
ZGOPTNS

======== CHANNEL f1 ========
SFO1          600.1342009 MHz
NUC1                   1H
P1                   8.00 usec
P2                  16.00 usec
P28                     0 usec
PLW1           6.16599989 W

======== CHANNEL f2 ========
SFO2          150.9194083 MHz
NUC2                  13C
CPDPRG[2             garp
P3                  18.75 usec
P14                500.00 usec
P24               2000.00 usec
PCPD2               65.00 usec
PLW0                    0 W
PLW2         164.00000000 W
PLW12         13.80399990 W
SPNAM[3] Crp60,0.5,20.1
SPOAL3              0.500
SPOFFS3                 0 Hz
SPW3          92.25700378 W
SPNAM[7]     Crp60comp.4
SPOAL7              0.500
SPOFFS7                 0 Hz
SPW7          92.25700378 W

====== GRADIENT CHANNEL =====
GPNAM[1]      SMSQ10.100
GPNAM[2]      SMSQ10.100
GPZ1                80.00 %
GPZ2                20.10 %
P16               1000.00 usec

F1 - Acquisition parameters
TD                    256
SFO1             150.9194 MHz
FIDRES         283.061584 Hz
SW                240.074 ppm
FnMODE     Echo-Antiecho

F2 - Processing parameters
SI                   1024
SF            600.1300360 MHz
WDW                    EM
SSB                     0
LB                   5.00 Hz
GB                      0
PC                   1.00

F1 - Processing parameters
SI                   1024
MC2         echo-antiecho
SF            150.9028030 MHz
WDW                 QSINE
SSB                     3
LB                      0 Hz
GB                      0
```

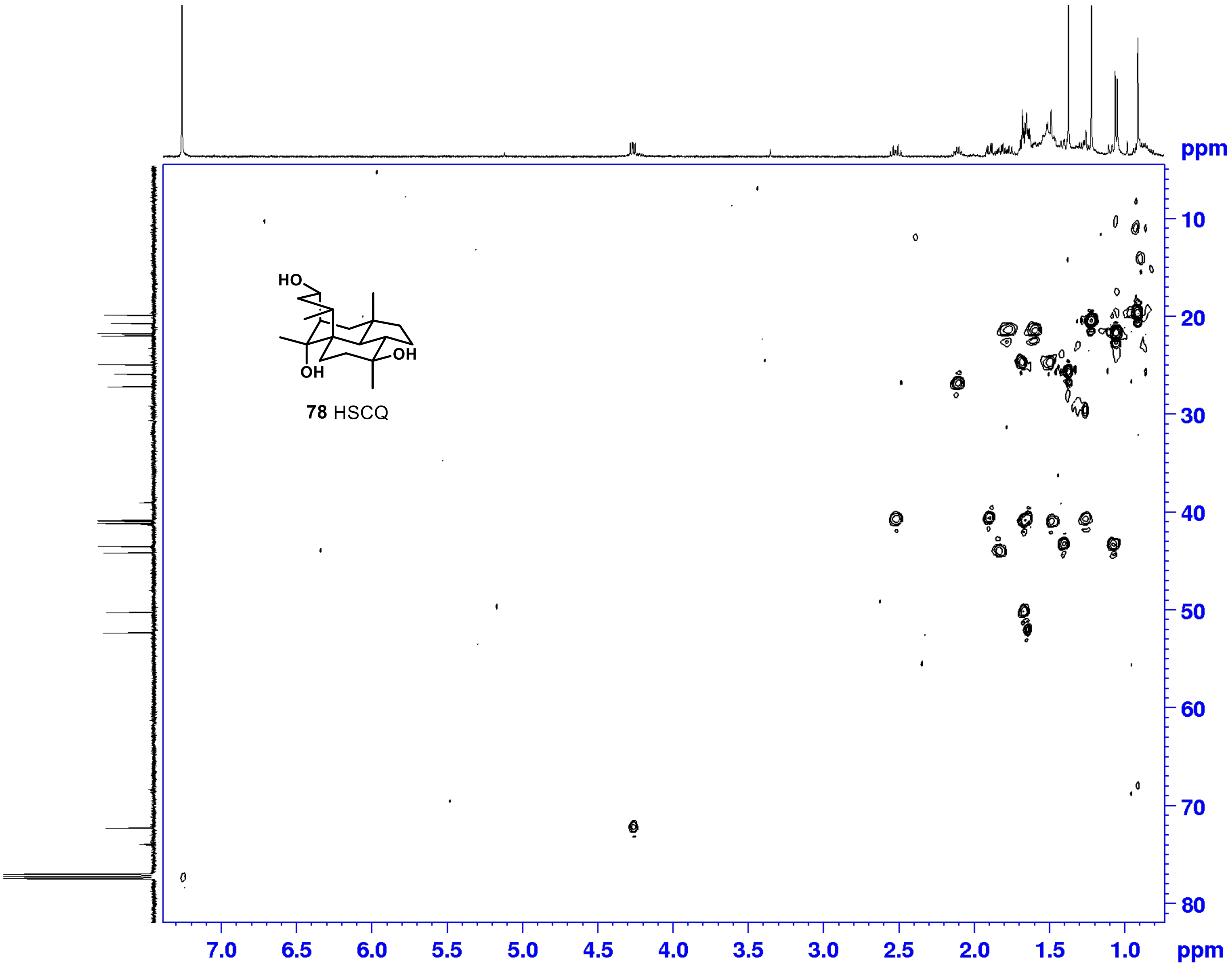

```
Current Data Parameters
NAME          JSCV-237V-2
EXPNO                   6
PROCNO                  1

F2 - Acquisition Parameters
Date_            20220923
Time                19.02
INSTRUM             av600
PROBHD   5 mm CPTCI 1H-
PULPROG     hmbcgplpndqf
TD                   4096
SOLVENT             CDCl3
NS                      2
DS                     16
SWH              9615.385 Hz
FIDRES           2.347506 Hz
AQ              0.2129920 sec
RG                   2050
DW                 52.000 usec
DE                  10.00 usec
TE                  298.0 K
CNST2         145.0000000
CNST13         10.0000000
D0             0.00000300 sec
D1             1.10000002 sec
D2             0.00344828 sec
D6             0.05000000 sec
D16            0.00020000 sec
IN0            0.00001380 sec

======== CHANNEL f1 ========
SFO1        600.1342009 MHz
NUC1                   1H
P1                   8.00 usec
P2                  16.00 usec
PLW1           6.16599989 W

======== CHANNEL f2 ========
SFO2        150.9194083 MHz
NUC2                  13C
P3                  18.75 usec
PLW2       164.00000000 W

====== GRADIENT CHANNEL =====
GPNAM[1]      SMSQ10.100
GPNAM[2]      SMSQ10.100
GPNAM[3]      SMSQ10.100
GPZ1                50.00 %
GPZ2                30.00 %
GPZ3                40.10 %
P16               1000.00 usec

F1 - Acquisition parameters
TD                    256
SFO1             150.9194 MHz
FIDRES         283.061584 Hz
SW                240.074 ppm
FnMODE                 QF

F2 - Processing parameters
SI                   2048
SF          600.1300314 MHz
WDW                  SINE
SSB                     0
LB                      0 Hz
GB                      0
PC                   1.00

F1 - Processing parameters
SI                   1024
MC2                    QF
SF          150.9028090 MHz
WDW                  SINE
SSB                     0
LB                      0 Hz
GB                      0
```

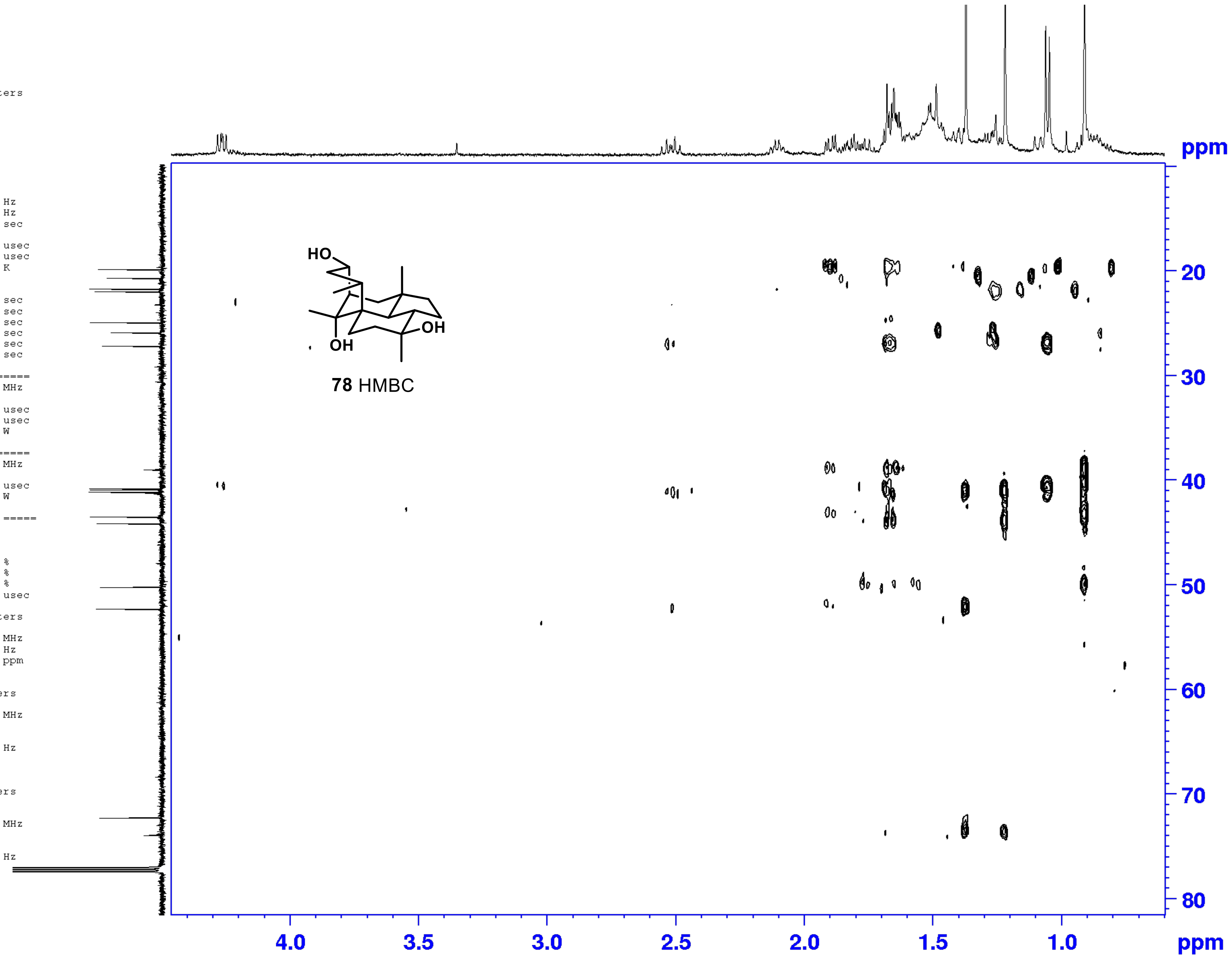

Current Data Parameters
NAME JSCV-237V-2
EXPNO 7
PROCNO 1

F2 - Acquisition Parameters
Date_ 20220923
Time 19.15
INSTRUM av600
PROBHD 5 mm CPTCI 1H-
PULPROG noesygpph
TD 2048
SOLVENT CDCl3
NS 2
DS 16
SWH 9615.385 Hz
FIDRES 4.695012 Hz
AQ 0.1064960 sec
RG 2050
DW 52.000 usec
DE 17.63 usec
TE 298.0 K
D0 0.00004181 sec
D1 2.00000000 sec
D8 1.00000000 sec
D16 0.00020000 sec
IN0 0.00010400 sec

======== CHANNEL f1 ========
SFO1 600.1342009 MHz
NUC1 1H
P1 8.00 usec
P2 16.00 usec
PLW1 6.16599989 W

====== GRADIENT CHANNEL =====
GPNAM[1] SMSQ10.100
GPZ1 40.00 %
P16 1000.00 usec

F1 - Acquisition parameters
TD 256
SFO1 600.1342 MHz
FIDRES 75.120193 Hz
SW 16.022 ppm
FnMODE States-TPPI

F2 - Processing parameters
SI 1024
SF 600.1300326 MHz
WDW QSINE
SSB 2
LB 0 Hz
GB 0
PC 1.00

F1 - Processing parameters
SI 1024
MC2 States-TPPI
SF 600.1300347 MHz
WDW QSINE
SSB 2
LB 0 Hz
GB 0

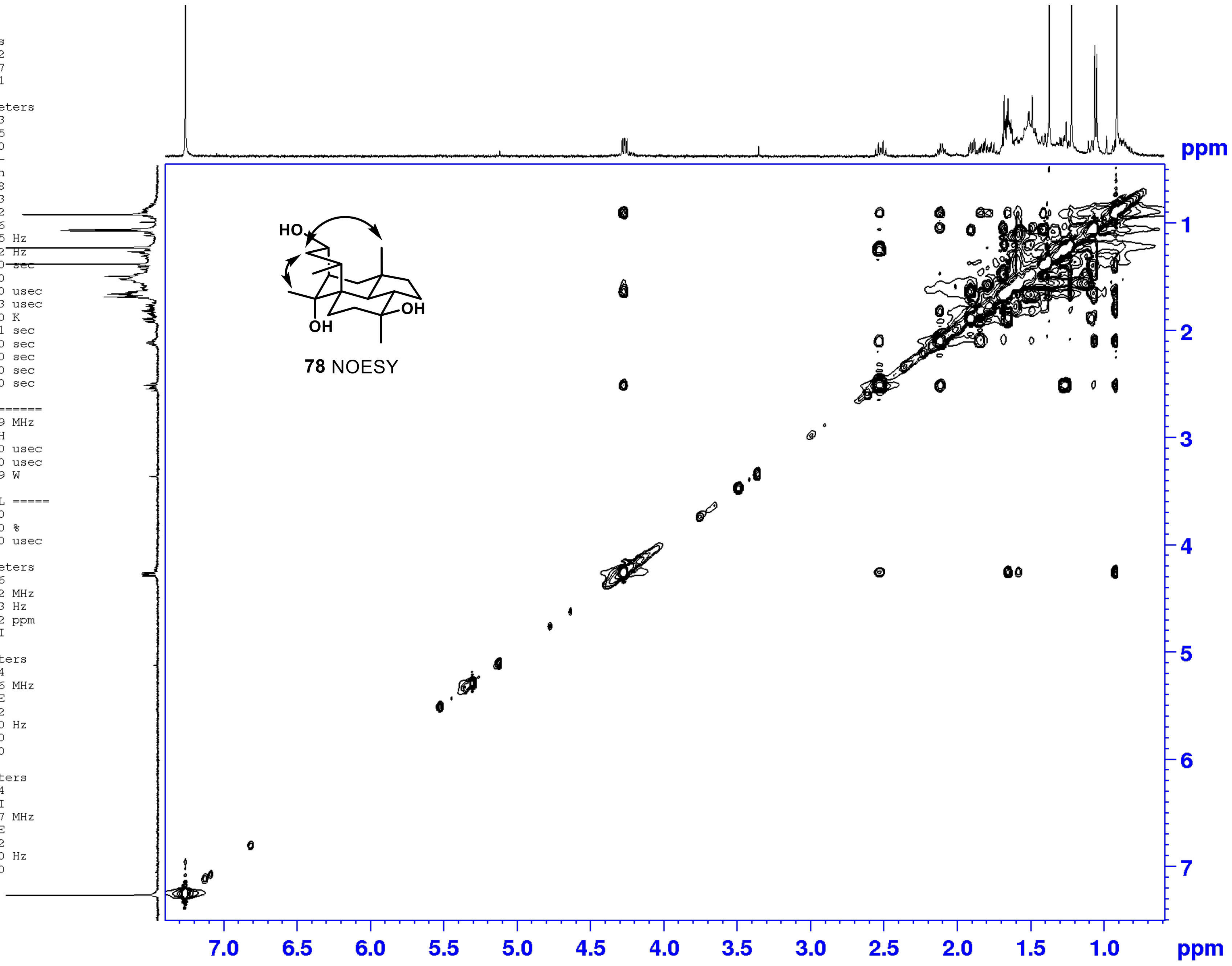

```
Current Data Parameters
NAME      JSCV-074-concd-rxn-mixture
EXPNO                    1
PROCNO                   1

F2 - Acquisition Parameters
Date_             20220211
Time                 17.53
INSTRUM              av600
PROBHD    5 mm CPBBO BB-
PULPROG               zg30
TD                   48074
SOLVENT              CDCl3
NS                       8
DS                       2
SWH               8403.361 Hz
FIDRES            0.174801 Hz
AQ               2.8604031 sec
RG                      10
DW                  59.500 usec
DE                   16.75 usec
TE                   297.9 K
D1              3.00000000 sec
TD0                      1

======== CHANNEL f1 ========
SFO1           600.1336008 MHz
NUC1                    1H
P1                    9.50 usec
PLW1           30.00000000 W

F2 - Processing parameters
SI                   65536
SF             600.1300342 MHz
WDW                     EM
SSB                      0
LB                    0.30 Hz
GB                       0
PC                    1.00
```

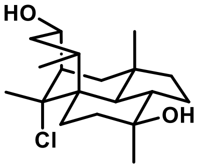

79, concentrated reaction mixture. Impure due to instability on SiO_2

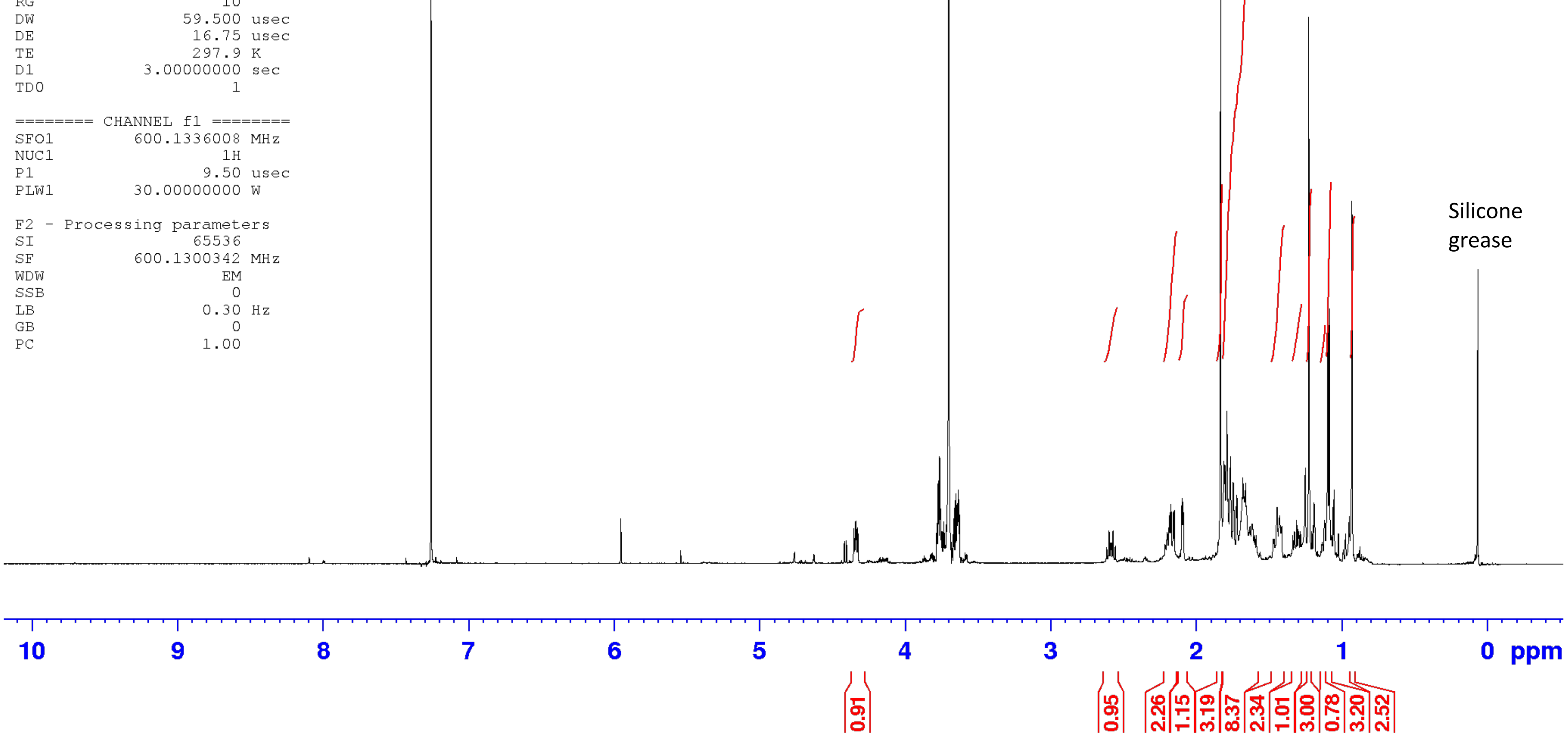

```
Current Data Parameters
NAME      JSCV-074-concd-rxn-mixture
EXPNO                     2
PROCNO                    1

F2 - Acquisition Parameters
Date_              20220211
Time                  17.57
INSTRUM               av600
PROBHD    5 mm CPBBO BB-
PULPROG             zgdc30
TD                   65536
SOLVENT              CDCl3
NS                     415
DS                       4
SWH              36231.883 Hz
FIDRES            0.552855 Hz
AQ               0.9043968 sec
RG                    2050
DW                  13.800 usec
DE                   19.63 usec
TE                   298.0 K
D1              0.40000001 sec
D11             0.03000000 sec
TD0                      1

======== CHANNEL f1 ========
SFO1           150.9194080 MHz
NUC1                   13C
P1                   10.10 usec
PLW1           64.00000000 W

======== CHANNEL f2 ========
SFO2           600.1330010 MHz
NUC2                    1H
CPDPRG[2           waltz16
PCPD2                80.00 usec
PLW2           30.00000000 W
PLW12           0.37154001 W

F2 - Processing parameters
SI                   65536
SF             150.9027934 MHz
WDW                     EM
SSB                      0
LB                    1.00 Hz
GB                       0
PC                    1.00
```

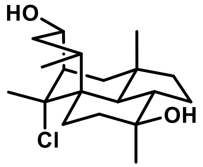

79, concentrated reaction mixture. Impure due to instability on SiO_2

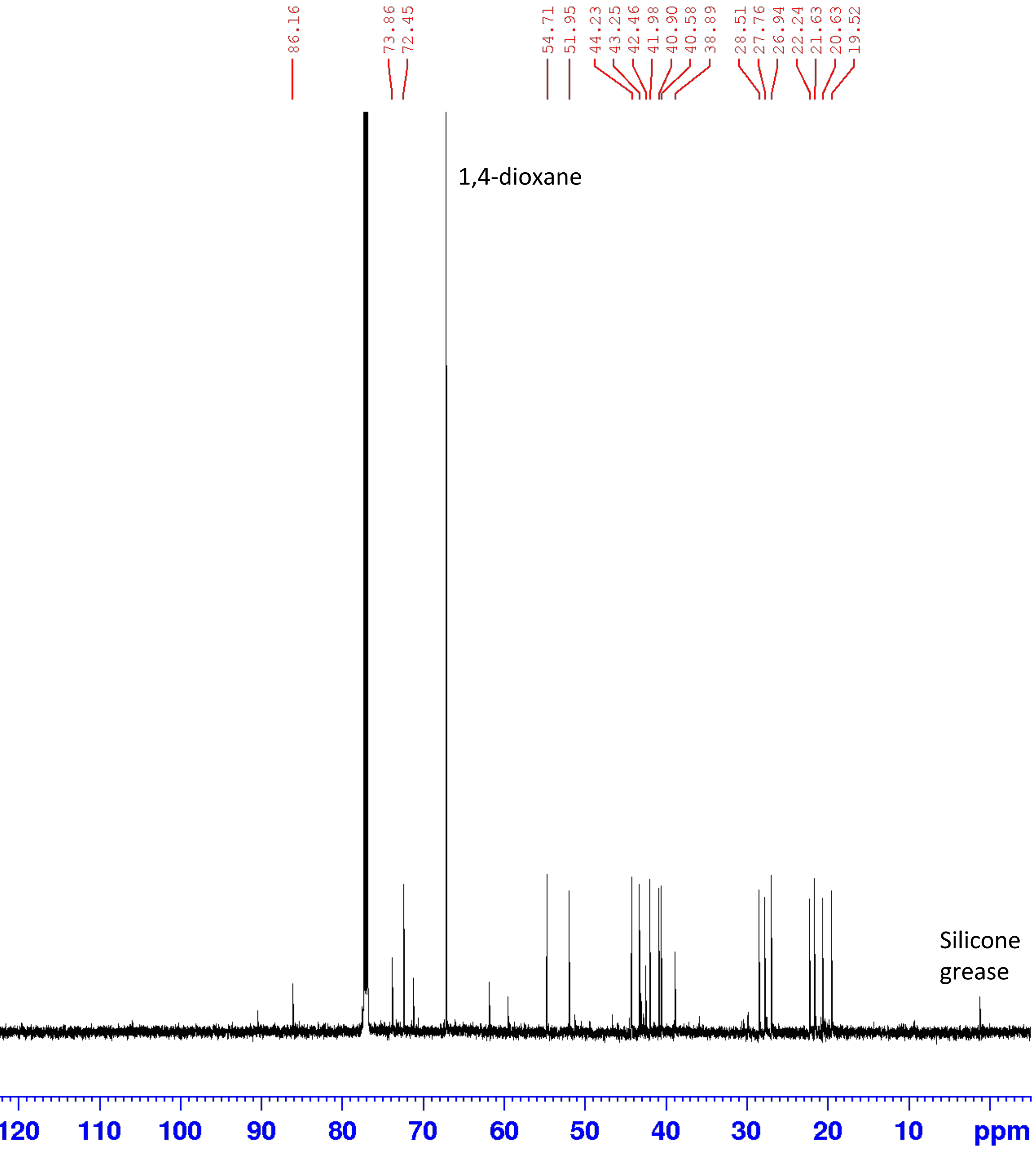

```
Current Data Parameters
NAME      JSCV-122-col5-f5-8
EXPNO                   1
PROCNO                  1

F2 - Acquisition Parameters
Date_            20220411
Time                15.48
INSTRUM             gn500
PROBHD   5 mm broadband
PULPROG              zg30
TD                  40062
SOLVENT             CDCl3
NS                     32
DS                      2
SWH              8012.820 Hz
FIDRES           0.200010 Hz
AQ              2.4998689 sec
RG                 2896.3
DW                 62.400 usec
DE                   6.00 usec
TE                  298.0 K
D1             3.00000000 sec
MCREST                  0 sec
MCWRK          0.01500000 sec

======== CHANNEL f1 ========
NUC1                   1H
P1                  12.00 usec
PL1                 -6.00 dB
SFO1          498.6534906 MHz

F2 - Processing parameters
SI                  65536
SF            498.6500284 MHz
WDW                    EM
SSB                     0
LB                   0.30 Hz
GB                      0
PC                   1.00
```

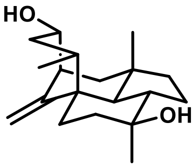

77

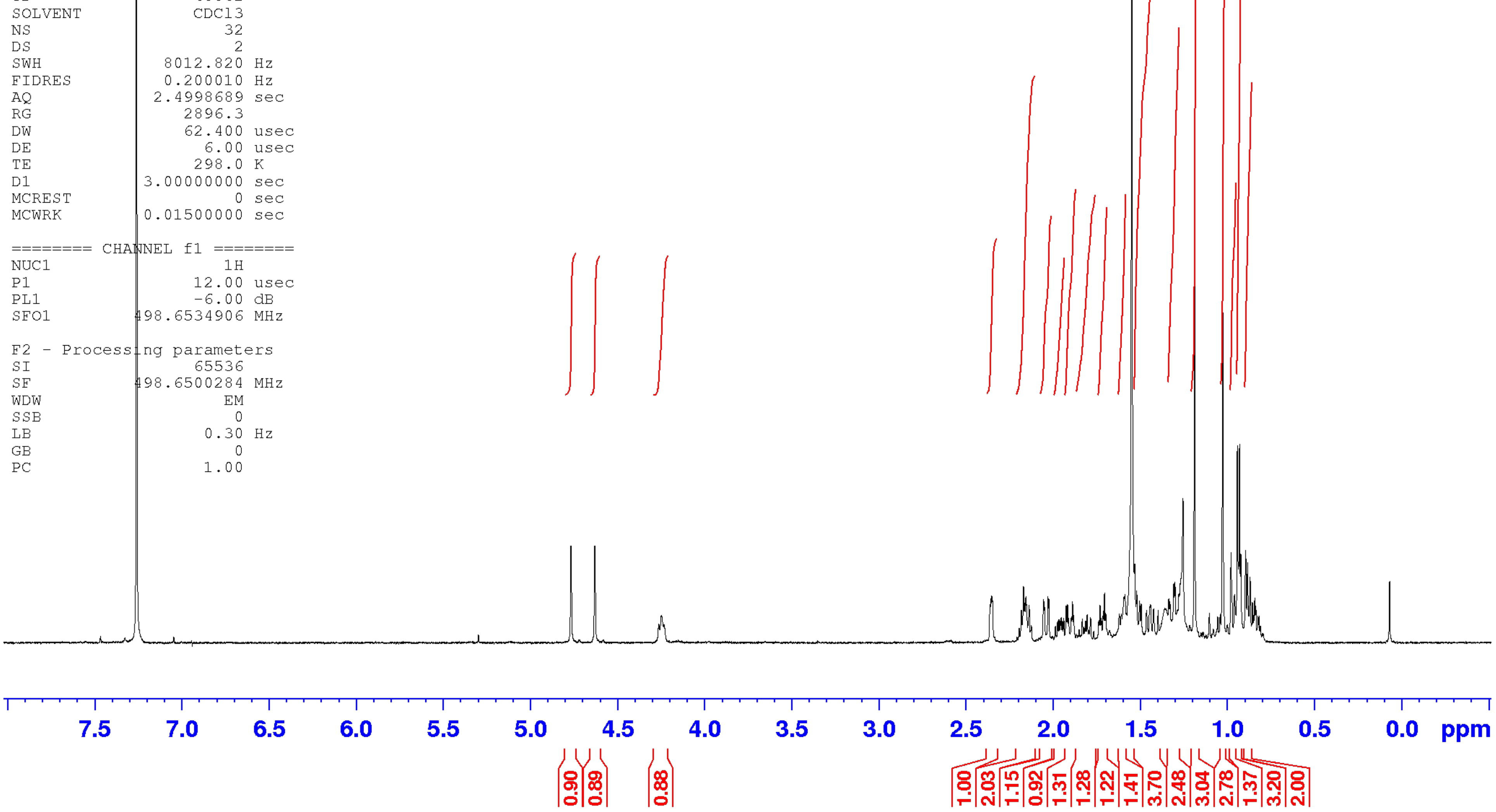

Current Data Parameters
NAME JSCV-122-col4-f9-23
EXPNO 2
PROCNO 1

F2 - Acquisition Parameters
Date_ 20220411
Time 11.34
INSTRUM av600
PROBHD 5 mm CPBBO BB-
PULPROG zgdc30
TD 65536
SOLVENT CDCl3
NS 404
DS 4
SWH 36231.883 Hz
FIDRES 0.552855 Hz
AQ 0.9043968 sec
RG 2050
DW 13.800 usec
DE 19.63 usec
TE 298.0 K
D1 0.40000001 sec
D11 0.03000000 sec
TD0 1

======== CHANNEL f1 ========
SFO1 150.9194080 MHz
NUC1 13C
P1 10.10 usec
PLW1 64.00000000 W

======== CHANNEL f2 ========
SFO2 600.1330010 MHz
NUC2 1H
CPDPRG[2 waltz16
PCPD2 80.00 usec
PLW2 30.00000000 W
PLW12 0.37154001 W

F2 - Processing parameters
SI 65536
SF 150.9027937 MHz
WDW EM
SSB 0
LB 1.00 Hz
GB 0
PC 1.00

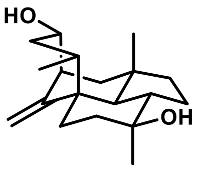

77

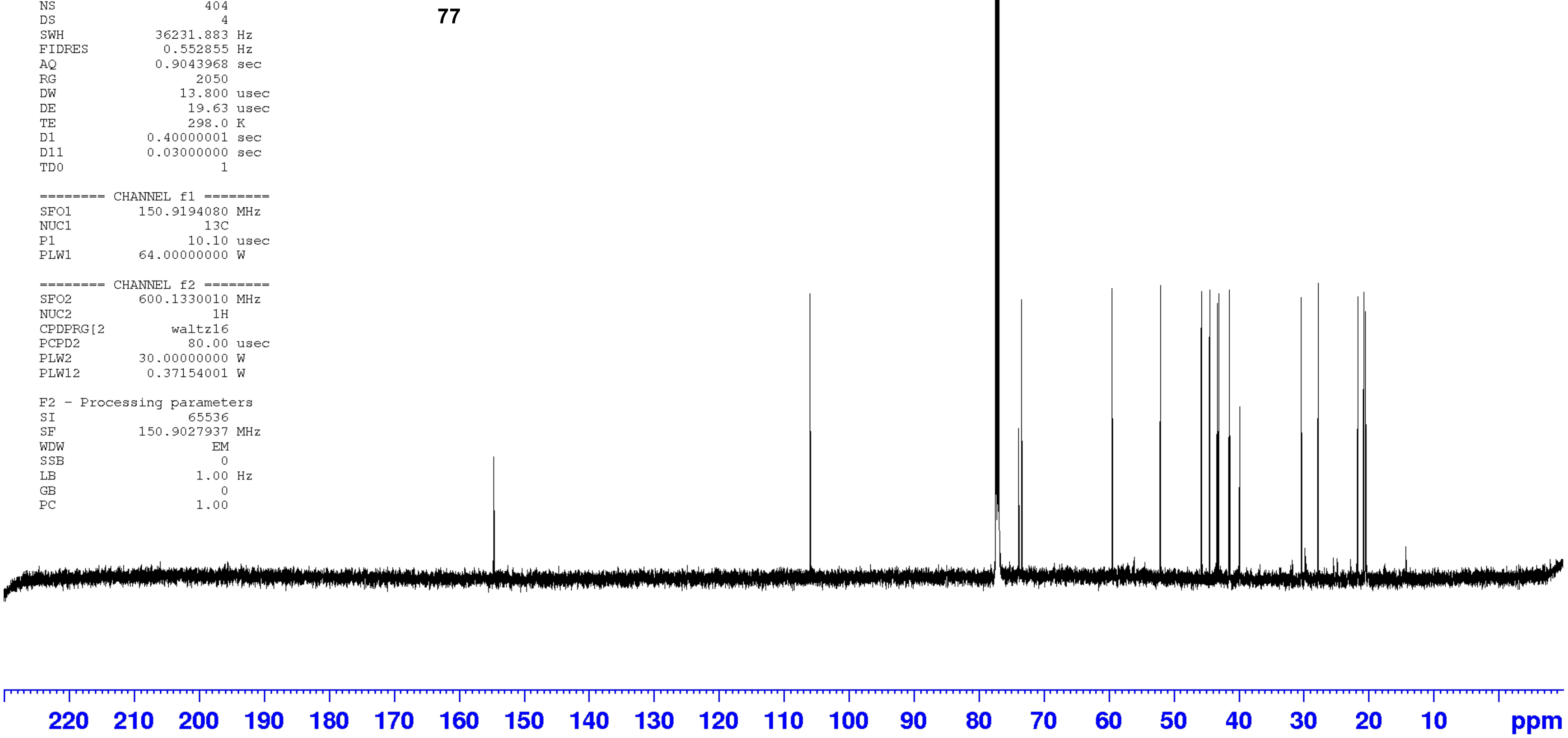

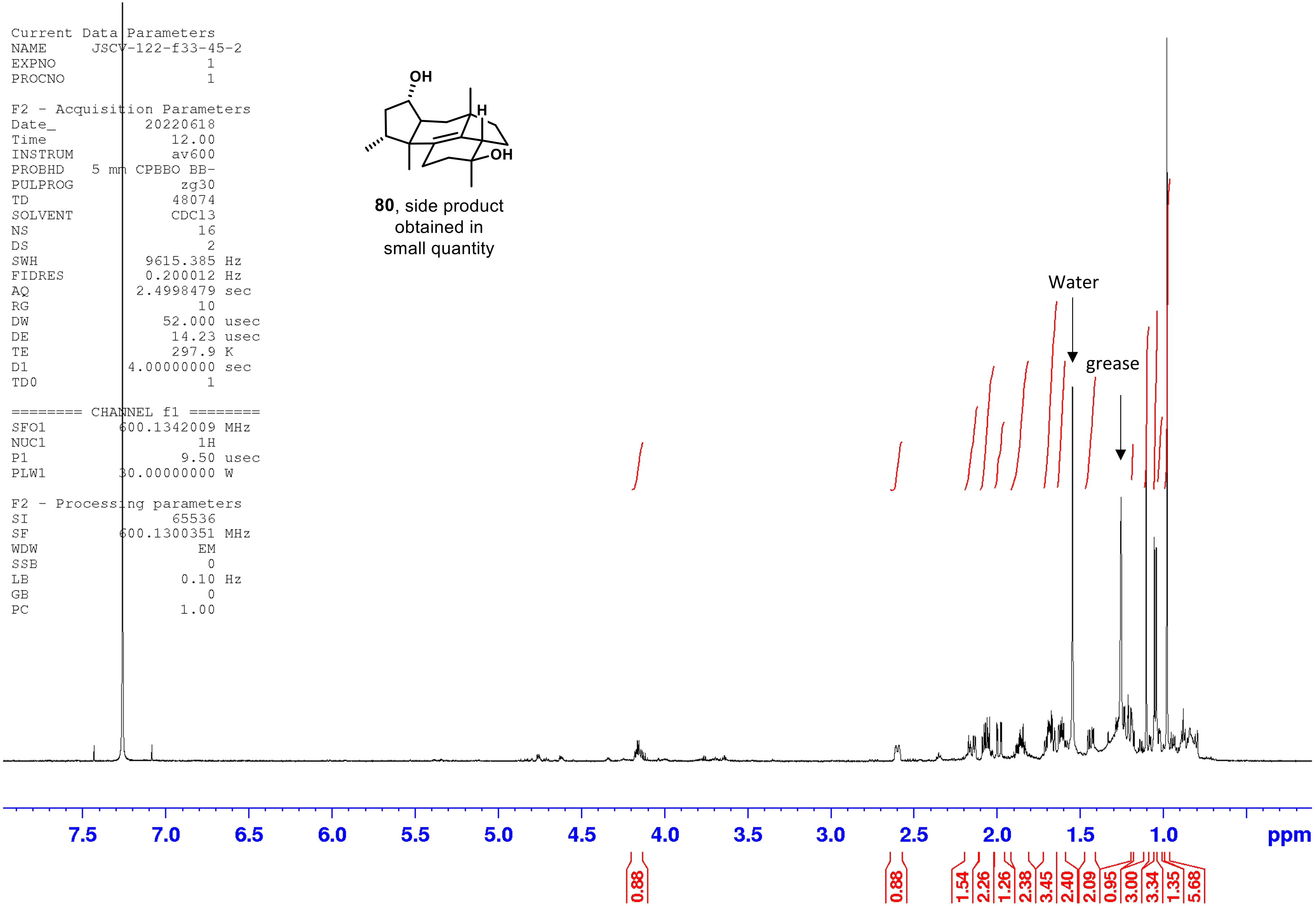
Current Data Parameters
NAME JSCV-122-f33-45-2
EXPNO 1
PROCNO 1
F2 - Acquisition Parameters
Date_ 20220618
Time 12.00
INSTRUM av600
PROBHD 5 mm CPBBO BB-
PULPROG zg30
TD 48074
SOLVENT CDCl3
NS 16
DS 2
SWH 9615.385 Hz
FIDRES 0.200012 Hz
AQ 2.4998479 sec
RG 10
DW 52.000 usec
DE 14.23 usec
TE 297.9 K
D1 4.00000000 sec
TD0 1
======== CHANNEL f1 ========
SFO1 600.1342009 MHz
NUC1 1H
P1 9.50 usec
PLW1 30.00000000 W
F2 - Processing parameters
SI 65536
SF 600.1300351 MHz
WDW EM
SSB 0
LB 0.10 Hz
GB 0
PC 1.00
OH
H
OH
80, side product obtained in small quantity
Water
grease
7.5
7.0
6.5
6.0
5.5
5.0
4.5
4.0
3.5
3.0
2.5
2.0
1.5
1.0
ppm
0.88
0.88
1.54
2.26
1.26
2.38
3.45
2.40
2.09
0.95
3.00
3.34
1.35
5.68

```
Current Data Parameters
NAME      JSCV-122-f33-45-2
EXPNO                     2
PROCNO                    1

F2 - Acquisition Parameters
Date_              20220618
Time                  12.03
INSTRUM               av600
PROBHD   5 mm CPBBO BB-
PULPROG             zgdc30
TD                    65536
SOLVENT               CDCl3
NS                      422
DS                        4
SWH             36231.883 Hz
FIDRES           0.552855 Hz
AQ              0.9043968 sec
RG                     2050
DW                   13.800 usec
DE                    19.63 usec
TE                    298.0 K
D1               0.40000001 sec
D11              0.03000000 sec
TD0                       1

======== CHANNEL f1 ========
SFO1         150.9194080 MHz
NUC1                    13C
P1                    10.10 usec
PLW1         64.00000000 W

======== CHANNEL f2 ========
SFO2         600.1330010 MHz
NUC2                     1H
CPDPRG[2            waltz16
PCPD2                 80.00 usec
PLW2         30.00000000 W
PLW12         0.37154001 W

F2 - Processing parameters
SI                    65536
SF           150.9027932 MHz
WDW                      EM
SSB                       0
LB                     1.00 Hz
GB                        0
PC                     1.00
```

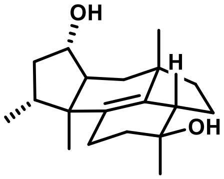

80, side product obtained in small quantity

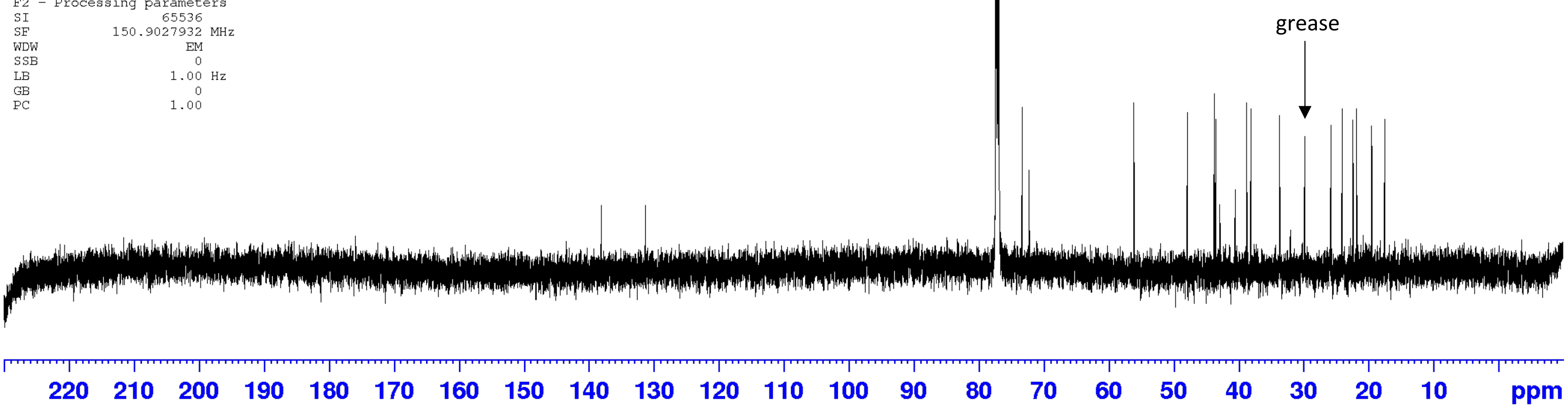

```
Current Data Parameters
NAME      JSCV-122-f33-45-2
EXPNO                     5
PROCNO                    1

F2 - Acquisition Parameters
Date_          20220618
Time              12.59
INSTRUM           av600
PROBHD   5 mm CPBBO BB-
PULPROG        cosygpqf
TD                 2048
SOLVENT           CDCl3
NS                    1
DS                   16
SWH            9615.385 Hz
FIDRES         4.695012 Hz
AQ            0.1064960 sec
RG                  724
DW               52.000 usec
DE                10.00 usec
TE                298.0 K
D0           0.00000300 sec
D1           1.48689198 sec
D13          0.00000400 sec
D16          0.00020000 sec
IN0          0.00010400 sec

======== CHANNEL f1 ========
SFO1        600.1342009 MHz
NUC1                 1H
P0                 9.50 usec
P1                 9.50 usec
PLW1        30.00000000 W

====== GRADIENT CHANNEL =====
GPNAM[1]      SMSQ10.100
GPZ1              10.00 %
P16             1000.00 usec

F1 - Acquisition parameters
TD                  256
SFO1           600.1342 MHz
FIDRES        75.120193 Hz
SW               16.022 ppm
FnMODE               QF

F2 - Processing parameters
SI                 1024
SF          600.1300345 MHz
WDW                SINE
SSB                   0
LB                    0 Hz
GB                    0
PC                 1.00

F1 - Processing parameters
SI                 1024
MC2                  QF
SF          600.1300375 MHz
WDW                SINE
SSB                   0
LB                    0 Hz
GB                    0
```

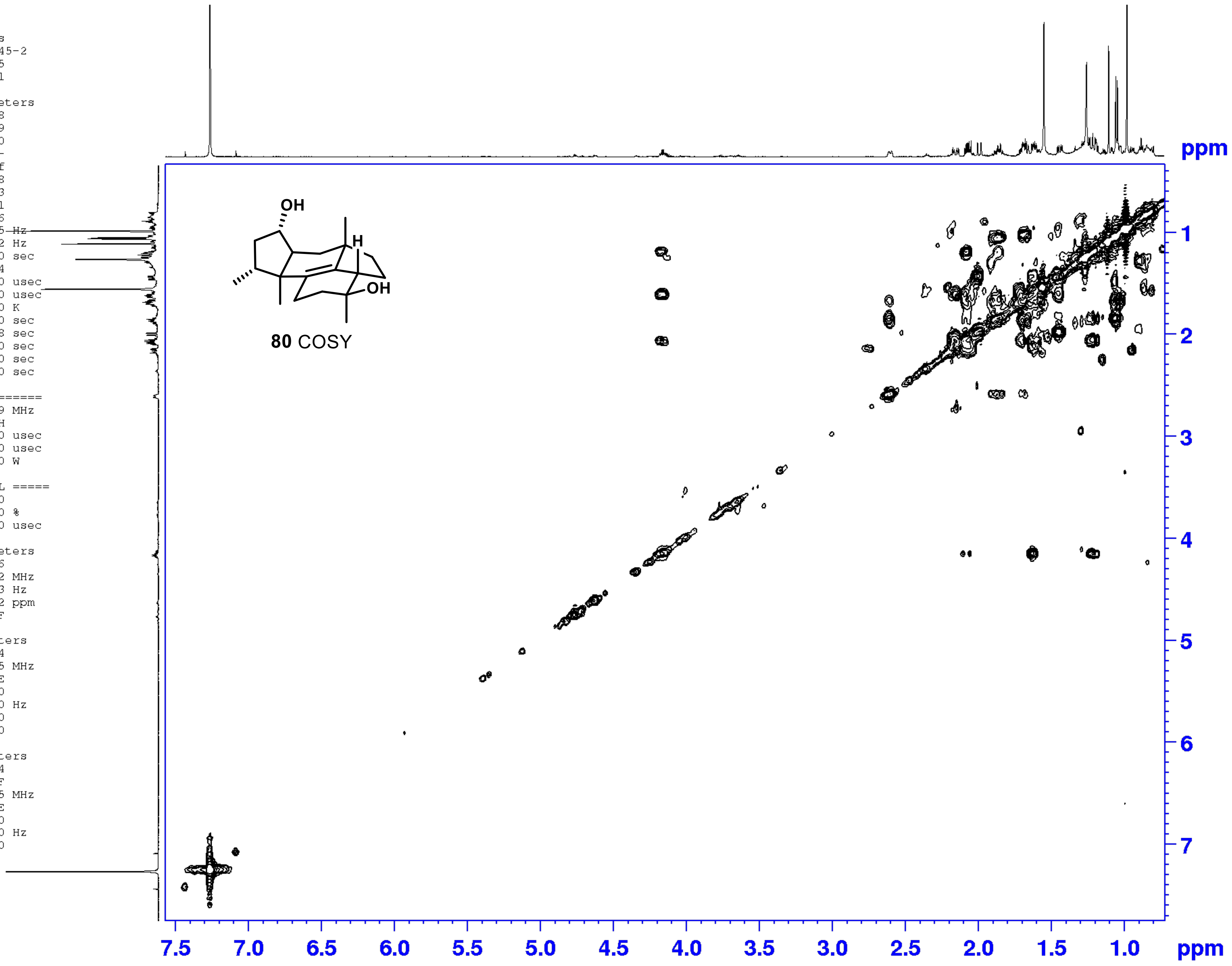

```
Current Data Parameters
NAME     JSCV-122-f33-45-2
EXPNO                    4
PROCNO                   1

F2 - Acquisition Parameters
Date_             20220618
Time                 12.39
INSTRUM              av600
PROBHD   5 mm CPBBO BB-
PULPROG     hsqcetgpsp.2
TD                    1024
SOLVENT              CDCl3
NS                       4
DS                      16
SWH               9615.385 Hz
FIDRES            9.390024 Hz
AQ               0.0532480 sec
RG                    2050
DW                  52.000 usec
DE                   10.00 usec
TE                   298.0 K
CNST2          145.0000000
D0              0.00000300 sec
D1              1.10000002 sec
D4              0.00172414 sec
D11             0.03000000 sec
D16             0.00020000 sec
IN0             0.00001380 sec
ZGOPTNS

======== CHANNEL f1 ========
SFO1        600.1342009 MHz
NUC1                    1H
P1                   10.00 usec
P2                   20.00 usec
P28                1000.00 usec
PLW1           30.00000000 W

======== CHANNEL f2 ========
SFO2        150.9194083 MHz
NUC2                   13C
CPDPRG[2              garp
P3                   10.10 usec
P14                 500.00 usec
P24                2000.00 usec
PCPD2                65.00 usec
PLW0                     0 W
PLW2           64.00000000 W
PLW12           1.47909999 W
SPNAM[3] Crp60,0.5,20.1
SPOAL3               0.500
SPOFFS3                  0 Hz
SPW3           10.00000000 W
SPNAM[7]     Crp60comp.4
SPOAL7               0.500
SPOFFS7                  0 Hz
SPW7           10.00000000 W

====== GRADIENT CHANNEL =====
GPNAM[1]      SMSQ10.100
GPNAM[2]      SMSQ10.100
GPZ1                 80.00 %
GPZ2                 20.10 %
P16                1000.00 usec

F1 - Acquisition parameters
TD                     256
SFO1              150.9194 MHz
FIDRES          283.061584 Hz
SW                 240.074 ppm
FnMODE     Echo-Antiecho

F2 - Processing parameters
SI                    1024
SF             600.1300335 MHz
WDW                     EM
SSB                      0
LB                    5.00 Hz
GB                       0
PC                    1.00

F1 - Processing parameters
SI                    1024
MC2         echo-antiecho
SF             150.9028024 MHz
WDW                  QSINE
SSB                      3
LB                       0 Hz
GB                       0
```

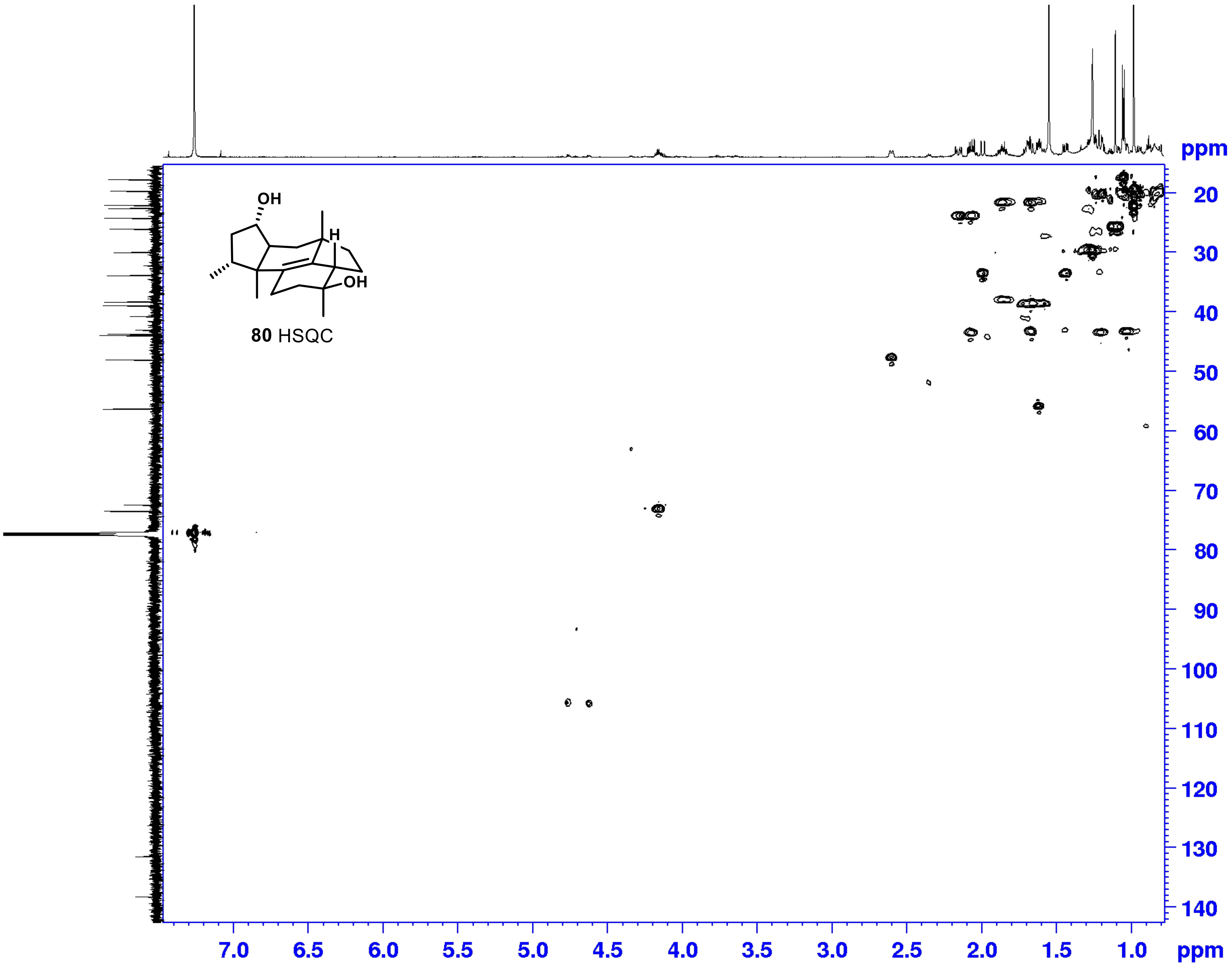

```
Current Data Parameters
NAME      JSCV-122-f33-45-2
EXPNO                    6
PROCNO                   1

F2 - Acquisition Parameters
Date_           20220618
Time               13.07
INSTRUM            av600
PROBHD   5 mm CPBBO BB-
PULPROG    hmbcgplpndqf
TD                  4096
SOLVENT            CDCl3
NS                     4
DS                    16
SWH             9615.385 Hz
FIDRES          2.347506 Hz
AQ             0.2129920 sec
RG                  2050
DW                52.000 usec
DE                 10.00 usec
TE                 298.0 K
CNST2        145.0000000
CNST13        10.0000000
D0            0.00000300 sec
D1            1.10000002 sec
D2            0.00344828 sec
D6            0.05000000 sec
D16           0.00020000 sec
IN0           0.00001380 sec

======== CHANNEL f1 ========
SFO1         600.1342009 MHz
NUC1                  1H
P1                  9.50 usec
P2                 19.00 usec
PLW1         30.00000000 W

======== CHANNEL f2 ========
SFO2         150.9194083 MHz
NUC2                 13C
P3                 10.10 usec
PLW2         64.00000000 W

====== GRADIENT CHANNEL =====
GPNAM[1]      SMSQ10.100
GPNAM[2]      SMSQ10.100
GPNAM[3]      SMSQ10.100
GPZ1               50.00 %
GPZ2               30.00 %
GPZ3               40.10 %
P16              1000.00 usec

F1 - Acquisition parameters
TD                   256
SFO1            150.9194 MHz
FIDRES        283.061584 Hz
SW               240.074 ppm
FnMODE                QF

F2 - Processing parameters
SI                  2048
SF           600.1300382 MHz
WDW                 SINE
SSB                    0
LB                     0 Hz
GB                     0
PC                  1.00

F1 - Processing parameters
SI                  1024
MC2                   QF
SF           150.9028242 MHz
WDW                 SINE
SSB                    0
LB                     0 Hz
GB                     0
```

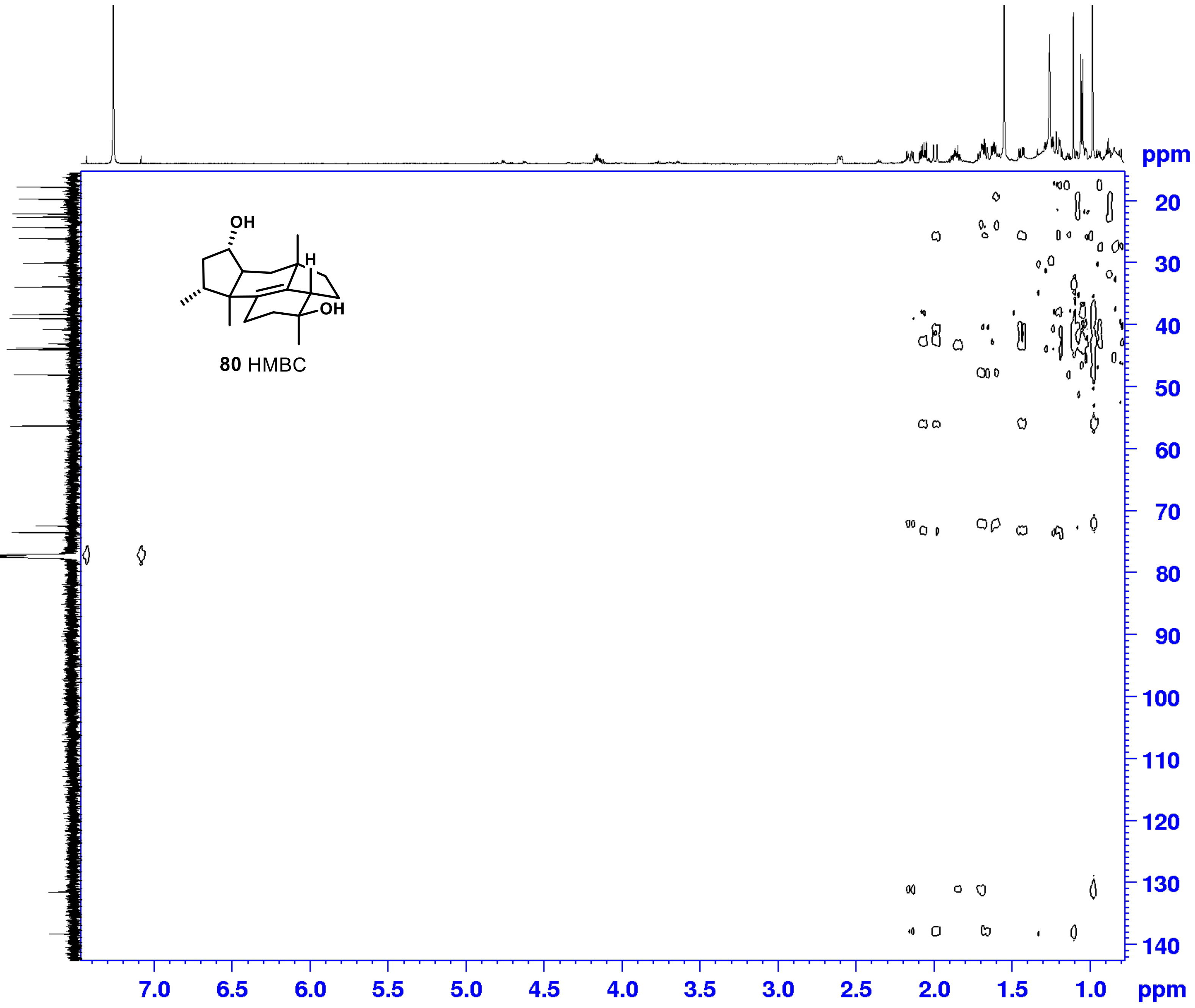

```
Current Data Parameters
NAME      JSCV-122-f33-45-2
EXPNO                  7
PROCNO                 1

F2 - Acquisition Parameters
Date_          20220618
Time              13.32
INSTRUM           av600
PROBHD   5 mm CPBBO BB-
PULPROG       noesygpph
TD                 2048
SOLVENT           CDCl3
NS                    2
DS                   16
SWH            9615.385 Hz
FIDRES         4.695012 Hz
AQ            0.1064960 sec
RG                  101
DW               52.000 usec
DE                16.68 usec
TE                297.9 K
D0           0.00003990 sec
D1           2.00000000 sec
D8           1.00000000 sec
D16          0.00020000 sec
IN0          0.00010400 sec

======== CHANNEL f1 ========
SFO1        600.1342009 MHz
NUC1                 1H
P1                 9.50 usec
P2                19.00 usec
PLW1        30.00000000 W

====== GRADIENT CHANNEL =====
GPNAM[1]      SMSQ10.100
GPZ1              40.00 %
P16             1000.00 usec

F1 - Acquisition parameters
TD                  256
SFO1           600.1342 MHz
FIDRES        75.120193 Hz
SW               16.022 ppm
FnMODE      States-TPPI

F2 - Processing parameters
SI                 1024
SF          600.1300407 MHz
WDW               QSINE
SSB                   2
LB                    0 Hz
GB                    0
PC                 1.00

F1 - Processing parameters
SI                 1024
MC2         States-TPPI
SF          600.1300319 MHz
WDW               QSINE
SSB                   2
LB                    0 Hz
GB                    0
```

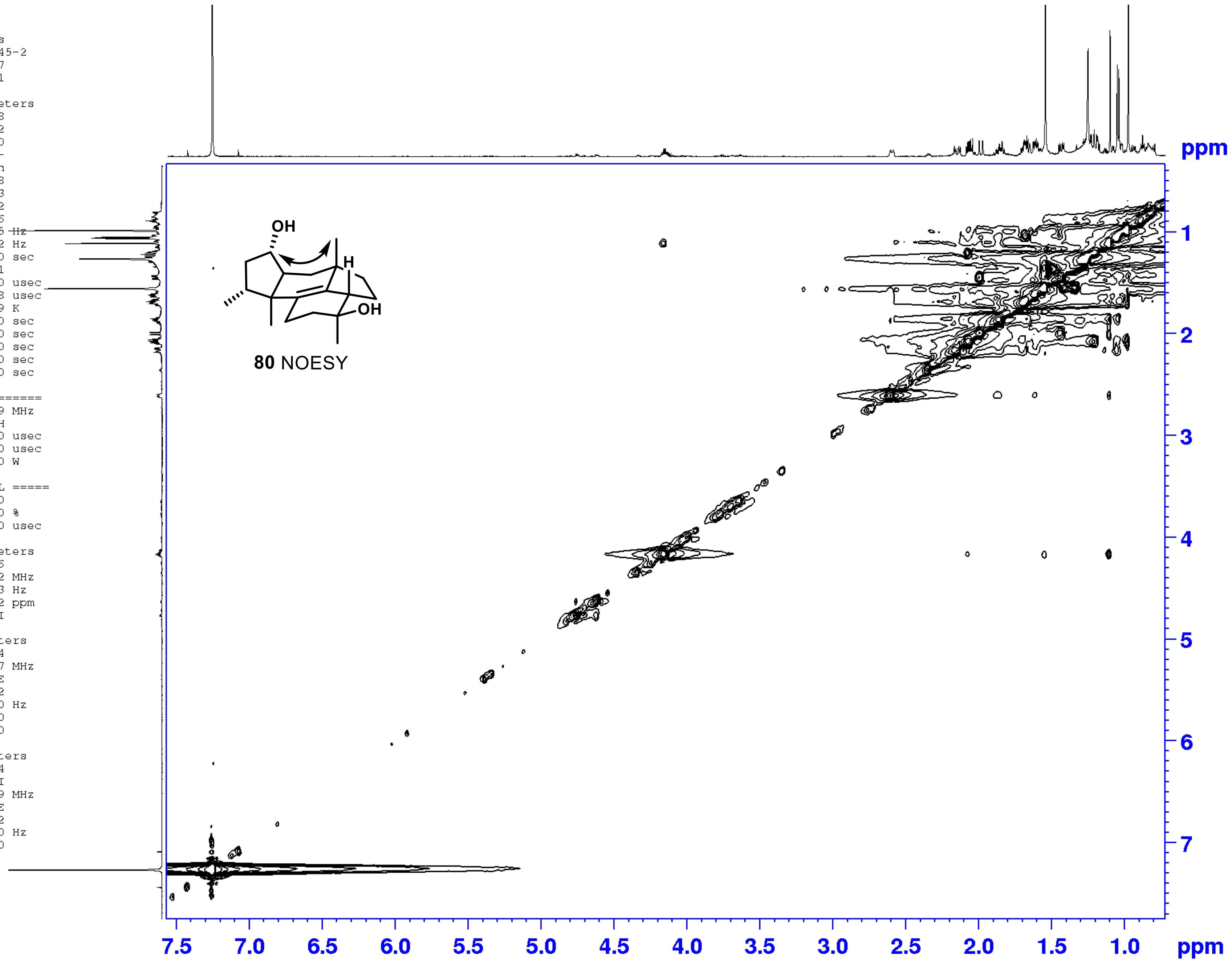

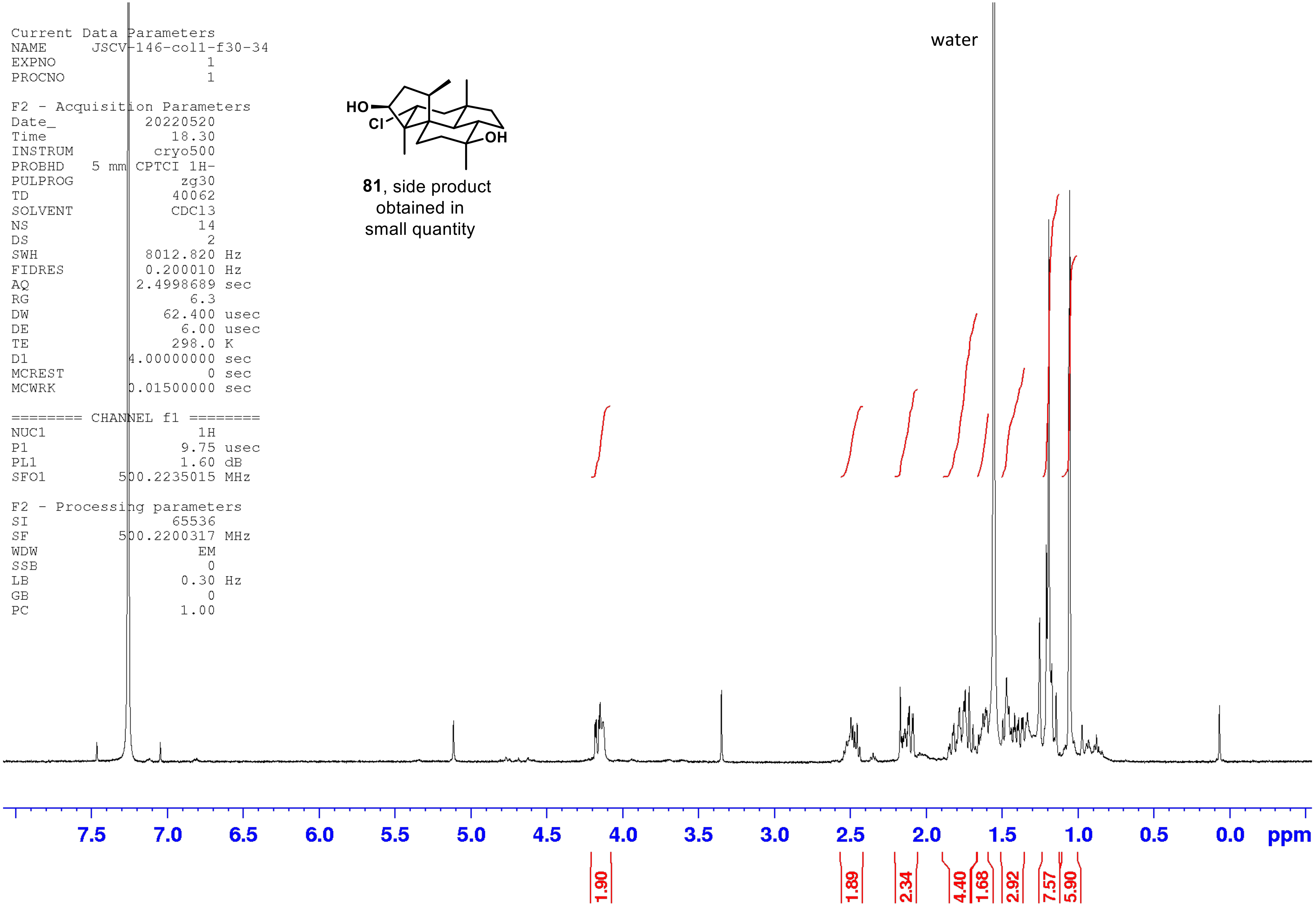

81, side product obtained in small quantity

```
Current Data Parameters
NAME      JSCV-146-f27-39-2
EXPNO                    2
PROCNO                   1

F2 - Acquisition Parameters
Date_             20220617
Time                 18.24
INSTRUM              av600
PROBHD   5 mm CPBBO BB-
PULPROG            zgdc30
TD                   65536
SOLVENT              CDCl3
NS                     367
DS                       4
SWH             36231.883 Hz
FIDRES           0.552855 Hz
AQ              0.9043968 sec
RG                    2050
DW                  13.800 usec
DE                   19.63 usec
TE                   298.0 K
D1             0.40000001 sec
D11            0.03000000 sec
TD0                      1

======== CHANNEL f1 ========
SFO1         150.9194080 MHz
NUC1                   13C
P1                   10.10 usec
PLW1         64.00000000 W

======== CHANNEL f2 ========
SFO2         600.1330010 MHz
NUC2                    1H
CPDPRG[2           waltz16
PCPD2                80.00 usec
PLW2         30.00000000 W
PLW12         0.37154001 W

F2 - Processing parameters
SI                   65536
SF           150.9027931 MHz
WDW                     EM
SSB                      0
LB                    1.00 Hz
GB                       0
PC                    1.00
```

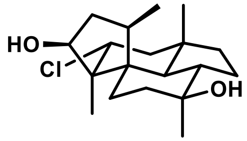

81, side product obtained in small quantity

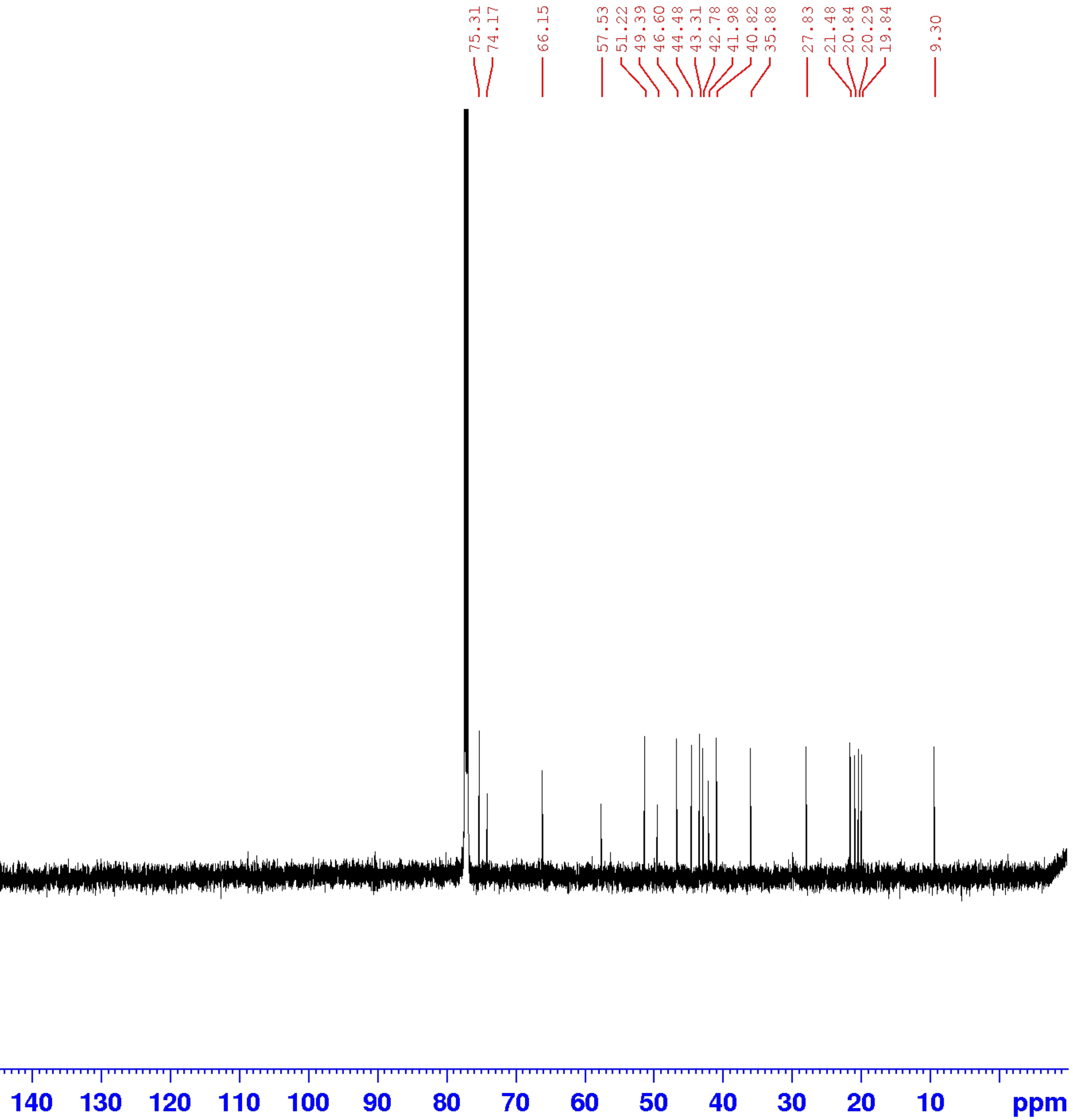

```
Current Data Parameters
NAME      JSCV-146-f27-39-2
EXPNO                  5
PROCNO                 1

F2 - Acquisition Parameters
Date_          20220617
Time              19.11
INSTRUM           av600
PROBHD   5 mm CPBBO BB-
PULPROG        cosygpqf
TD                 2048
SOLVENT           CDCl3
NS                    1
DS                   16
SWH            9615.385 Hz
FIDRES         4.695012 Hz
AQ            0.1064960 sec
RG                  724
DW               52.000 usec
DE                10.00 usec
TE                297.9 K
D0           0.00000300 sec
D1           1.48689198 sec
D13          0.00000400 sec
D16          0.00020000 sec
IN0          0.00010400 sec

======== CHANNEL f1 ========
SFO1        600.1342009 MHz
NUC1                 1H
P0                 9.50 usec
P1                 9.50 usec
PLW1        30.00000000 W

====== GRADIENT CHANNEL =====
GPNAM[1]      SMSQ10.100
GPZ1              10.00 %
P16             1000.00 usec

F1 - Acquisition parameters
TD                  256
SFO1           600.1342 MHz
FIDRES        75.120193 Hz
SW               16.022 ppm
FnMODE               QF

F2 - Processing parameters
SI                 1024
SF          600.1300349 MHz
WDW                SINE
SSB                   0
LB                    0 Hz
GB                    0
PC                 1.00

F1 - Processing parameters
SI                 1024
MC2                  QF
SF          600.1300326 MHz
WDW                SINE
SSB                   0
LB                    0 Hz
GB                    0
```

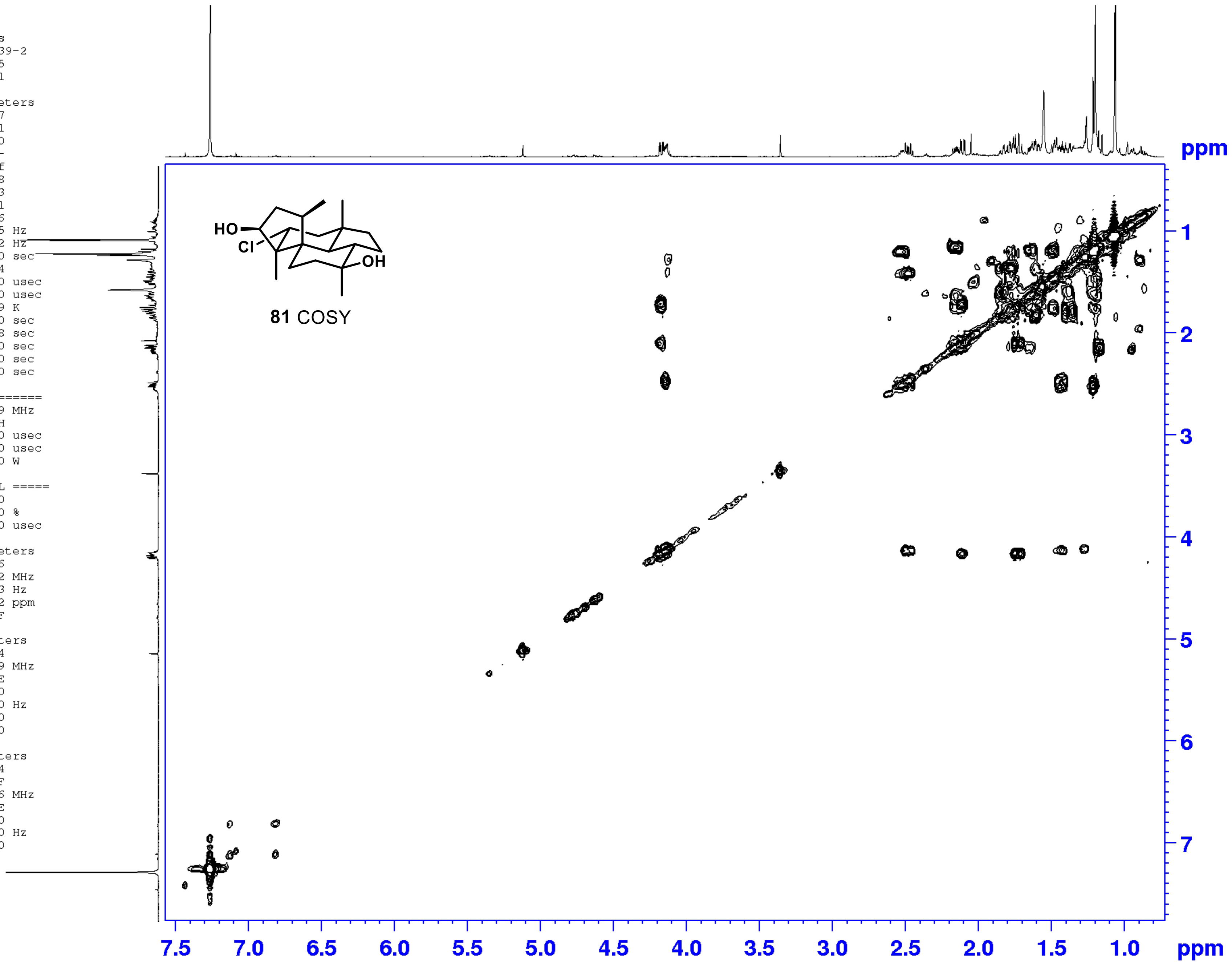

```
Current Data Parameters
NAME      JSCV-146-f27-39-2
EXPNO                    4
PROCNO                   1

F2 - Acquisition Parameters
Date_             20220617
Time                 19.00
INSTRUM              av600
PROBHD    5 mm CPBBO BB-
PULPROG     hsqcetgpsp.2
TD                    1024
SOLVENT              CDCl3
NS                       2
DS                      16
SWH              9615.385 Hz
FIDRES           9.390024 Hz
AQ              0.0532480 sec
RG                     912
DW                  52.000 usec
DE                   10.00 usec
TE                   297.9 K
CNST2         145.0000000
D0             0.00000300 sec
D1             1.10000002 sec
D4             0.00172414 sec
D11            0.03000000 sec
D16            0.00020000 sec
IN0            0.00001380 sec
ZGOPTNS

======== CHANNEL f1 ========
SFO1          600.1342009 MHz
NUC1                    1H
P1                   10.00 usec
P2                   20.00 usec
P28                1000.00 usec
PLW1          30.00000000 W

======== CHANNEL f2 ========
SFO2          150.9194083 MHz
NUC2                   13C
CPDPRG[2              garp
P3                   10.10 usec
P14                 500.00 usec
P24                2000.00 usec
PCPD2                65.00 usec
PLW0                     0 W
PLW2          64.00000000 W
PLW12          1.47909999 W
SPNAM[3] Crp60,0.5,20.1
SPOAL3               0.500
SPOFFS3                  0 Hz
SPW3          10.00000000 W
SPNAM[7]      Crp60comp.4
SPOAL7               0.500
SPOFFS7                  0 Hz
SPW7          10.00000000 W

====== GRADIENT CHANNEL =====
GPNAM[1]       SMSQ10.100
GPNAM[2]       SMSQ10.100
GPZ1                 80.00 %
GPZ2                 20.10 %
P16                1000.00 usec

F1 - Acquisition parameters
TD                     256
SFO1              150.9194 MHz
FIDRES          283.061584 Hz
SW                 240.074 ppm
FnMODE      Echo-Antiecho

F2 - Processing parameters
SI                    1024
SF            600.1300354 MHz
WDW                     EM
SSB                      0
LB                    5.00 Hz
GB                       0
PC                    1.00

F1 - Processing parameters
SI                    1024
MC2         echo-antiecho
SF            150.9028014 MHz
WDW                  QSINE
SSB                      3
LB                       0 Hz
GB                       0
```

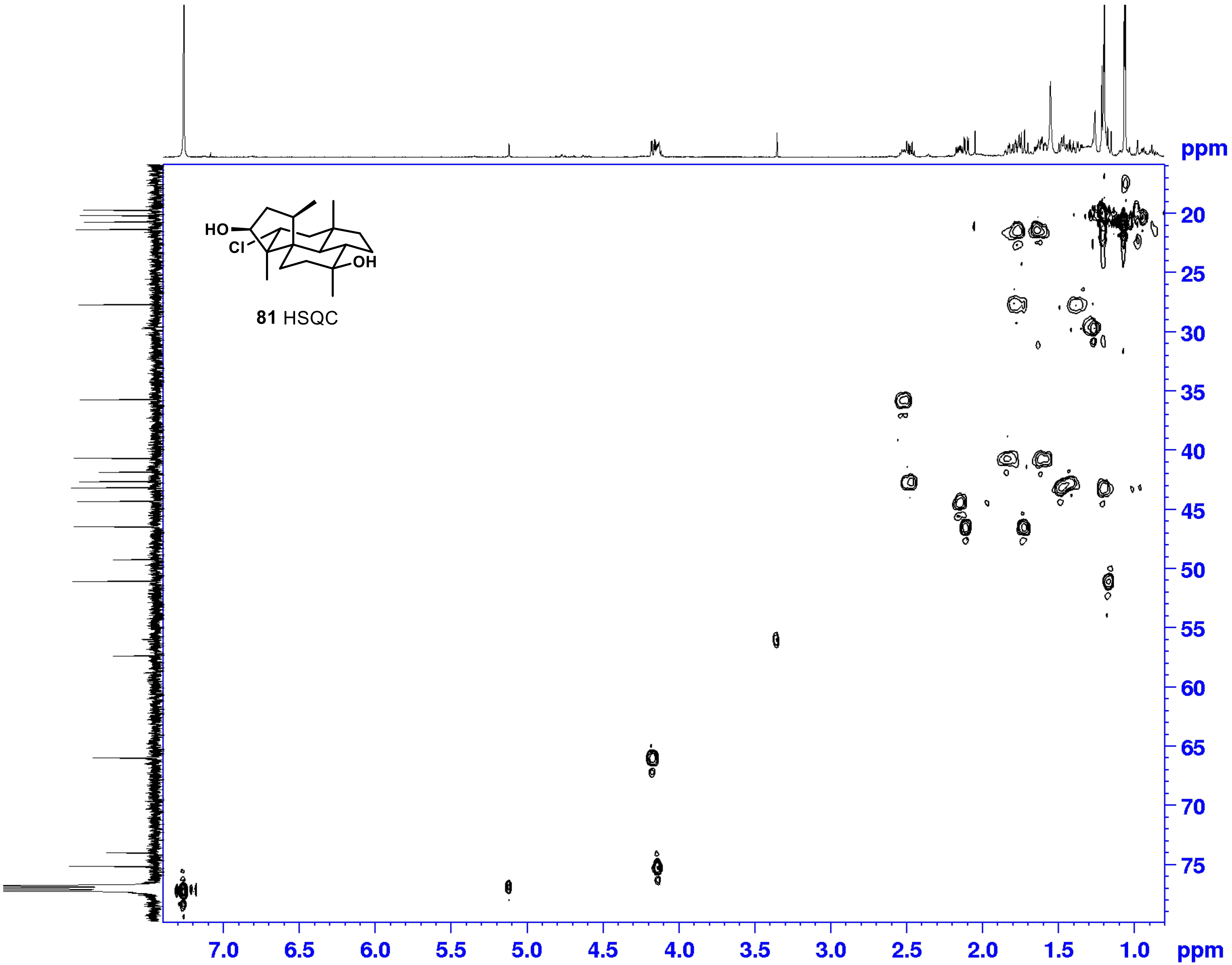

Current Data Parameters
NAME JSCV-146-f27-39-2
EXPNO 6
PROCNO 1

F2 - Acquisition Parameters
Date_ 20220617
Time 19.18
INSTRUM av600
PROBHD 5 mm CPBBO BB-
PULPROG hmbcgplpndqf
TD 4096
SOLVENT CDCl3
NS 2
DS 16
SWH 9615.385 Hz
FIDRES 2.347506 Hz
AQ 0.2129920 sec
RG 2050
DW 52.000 usec
DE 10.00 usec
TE 297.9 K
CNST2 145.0000000
CNST13 10.0000000
D0 0.00000300 sec
D1 1.10000002 sec
D2 0.00344828 sec
D6 0.05000000 sec
D16 0.00020000 sec
IN0 0.00001380 sec

======== CHANNEL f1 ========
SFO1 600.1342009 MHz
NUC1 1H
P1 9.50 usec
P2 19.00 usec
PLW1 30.00000000 W

======== CHANNEL f2 ========
SFO2 150.9194083 MHz
NUC2 13C
P3 10.10 usec
PLW2 64.00000000 W

====== GRADIENT CHANNEL =====
GPNAM[1] SMSQ10.100
GPNAM[2] SMSQ10.100
GPNAM[3] SMSQ10.100
GPZ1 50.00 %
GPZ2 30.00 %
GPZ3 40.10 %
P16 1000.00 usec

F1 - Acquisition parameters
TD 256
SFO1 150.9194 MHz
FIDRES 283.061584 Hz
SW 240.074 ppm
FnMODE QF

F2 - Processing parameters
SI 2048
SF 600.1300327 MHz
WDW SINE
SSB 0
LB 0 Hz
GB 0
PC 1.00

F1 - Processing parameters
SI 1024
MC2 QF
SF 150.9028234 MHz
WDW SINE
SSB 0
LB 0 Hz
GB 0

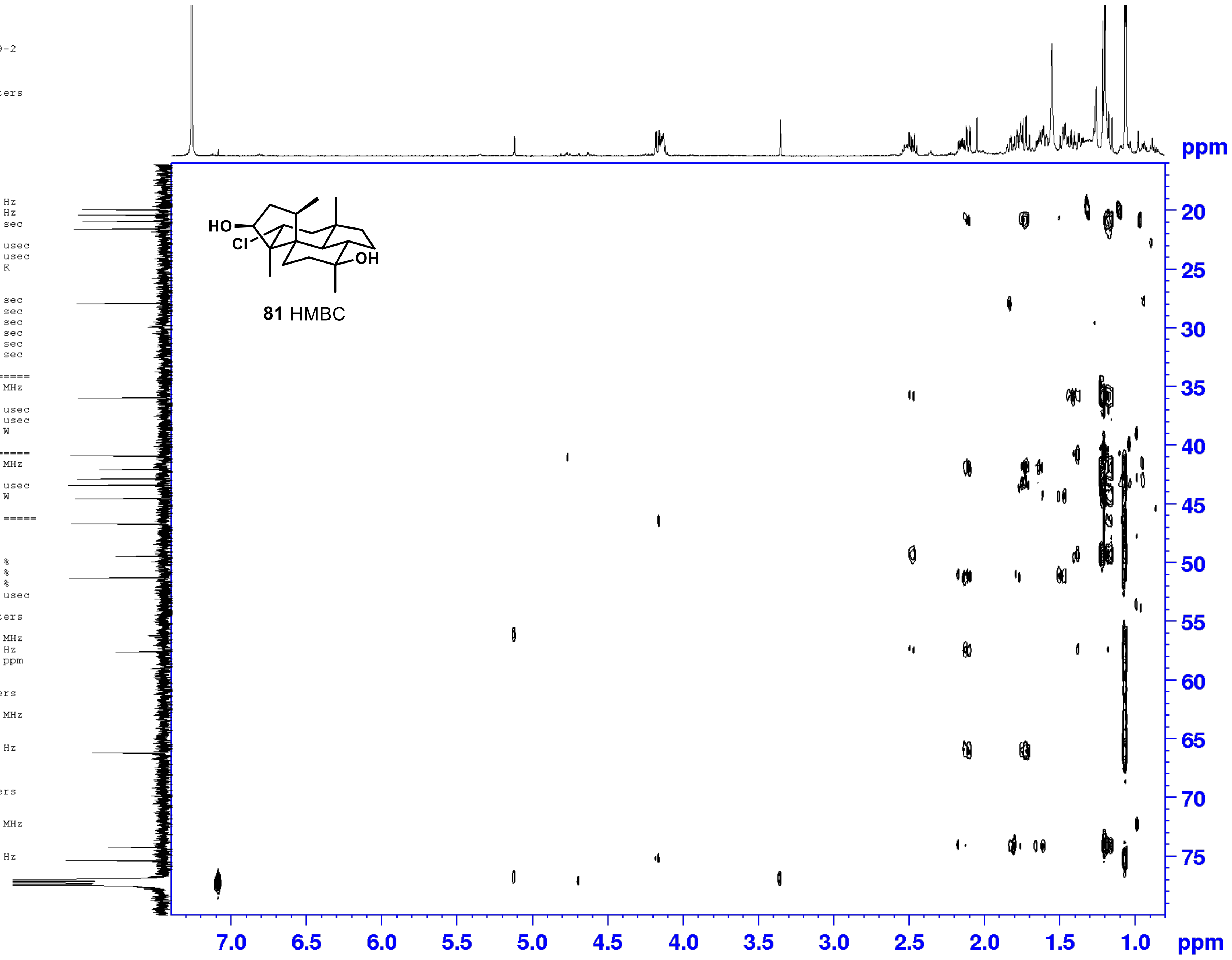

```
Current Data Parameters
NAME      JSCV-146-f27-39-2
EXPNO                     7
PROCNO                    1

F2 - Acquisition Parameters
Date_          20220617
Time              19.31
INSTRUM           av600
PROBHD   5 mm CPBBO BB-
PULPROG       noesygpph
TD                 2048
SOLVENT           CDCl3
NS                    2
DS                   16
SWH            9615.385 Hz
FIDRES         4.695012 Hz
AQ            0.1064960 sec
RG                  101
DW               52.000 usec
DE                16.68 usec
TE                298.0 K
D0           0.00003990 sec
D1           2.00000000 sec
D8           1.00000000 sec
D16          0.00020000 sec
IN0          0.00010400 sec

======== CHANNEL f1 ========
SFO1        600.1342009 MHz
NUC1                 1H
P1                 9.50 usec
P2                19.00 usec
PLW1        30.00000000 W

====== GRADIENT CHANNEL =====
GPNAM[1]      SMSQ10.100
GPZ1              40.00 %
P16             1000.00 usec

F1 - Acquisition parameters
TD                  256
SFO1           600.1342 MHz
FIDRES        75.120193 Hz
SW               16.022 ppm
FnMODE       States-TPPI

F2 - Processing parameters
SI                 1024
SF          600.1300380 MHz
WDW               QSINE
SSB                   2
LB                    0 Hz
GB                    0
PC                 1.00

F1 - Processing parameters
SI                 1024
MC2         States-TPPI
SF          600.1300331 MHz
WDW               QSINE
SSB                   2
LB                    0 Hz
GB                    0
```

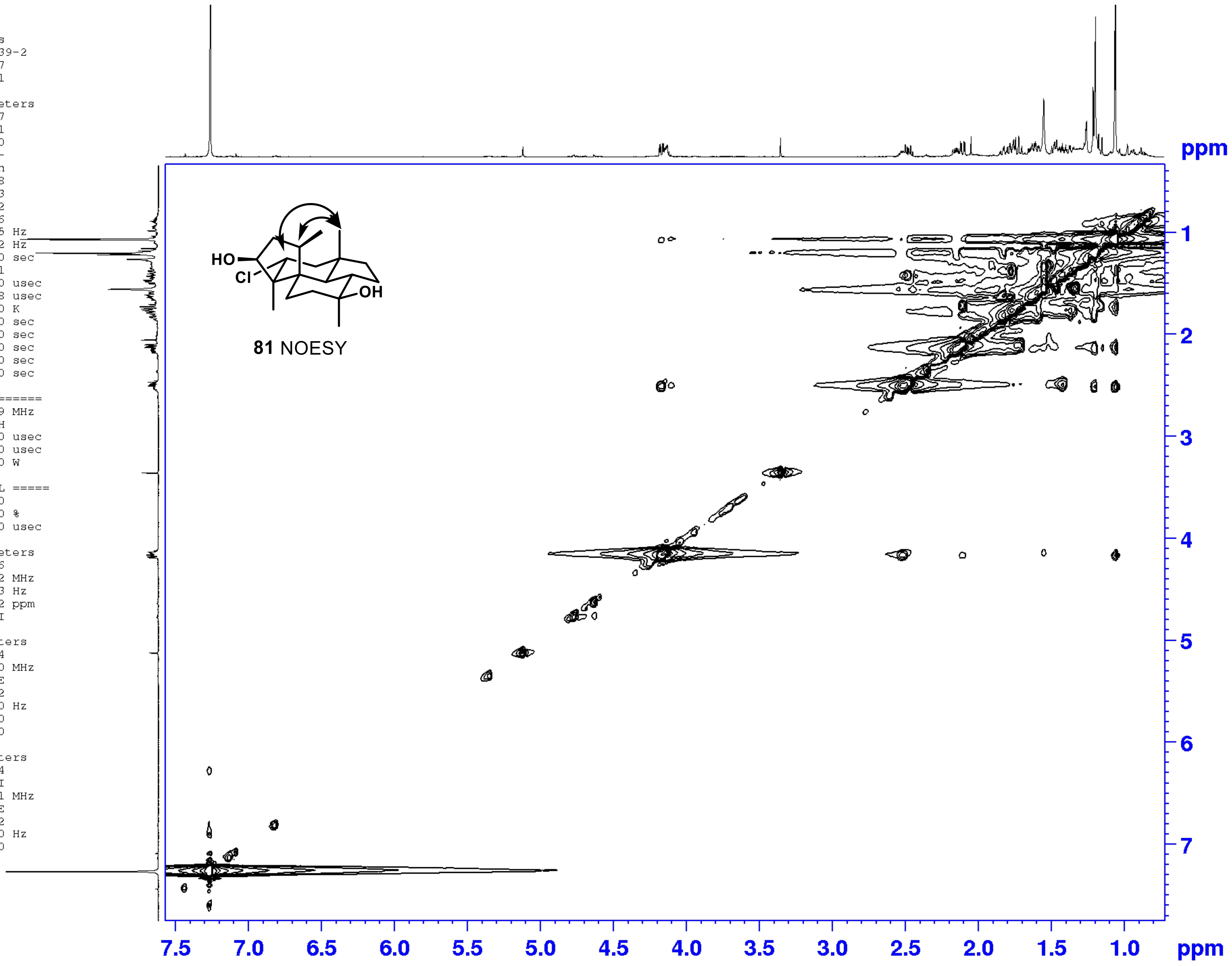

```
Current Data Parameters
NAME      JSCV-280-f6-20
EXPNO                  1
PROCNO                 1

F2 - Acquisition Parameters
Date_           20221109
Time               11.22
INSTRUM          cryo500
PROBHD    5 mm CPTCI 1H-
PULPROG             zg30
TD                 40062
SOLVENT            CDCl3
NS                     8
DS                     2
SWH             8012.820 Hz
FIDRES          0.200010 Hz
AQ             2.4998689 sec
RG                   6.3
DW                62.400 usec
DE                  6.00 usec
TE                 298.0 K
D1            6.00000000 sec
MCREST                 0 sec
MCWRK         0.01500000 sec

======== CHANNEL f1 ========
NUC1                  1H
P1                  9.75 usec
PL1                 1.60 dB
SFO1         500.2235015 MHz

F2 - Processing parameters
SI                 65536
SF           500.2200319 MHz
WDW                   EM
SSB                    0
LB                  0.30 Hz
GB                     0
PC                  1.00
```

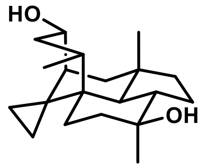

S24

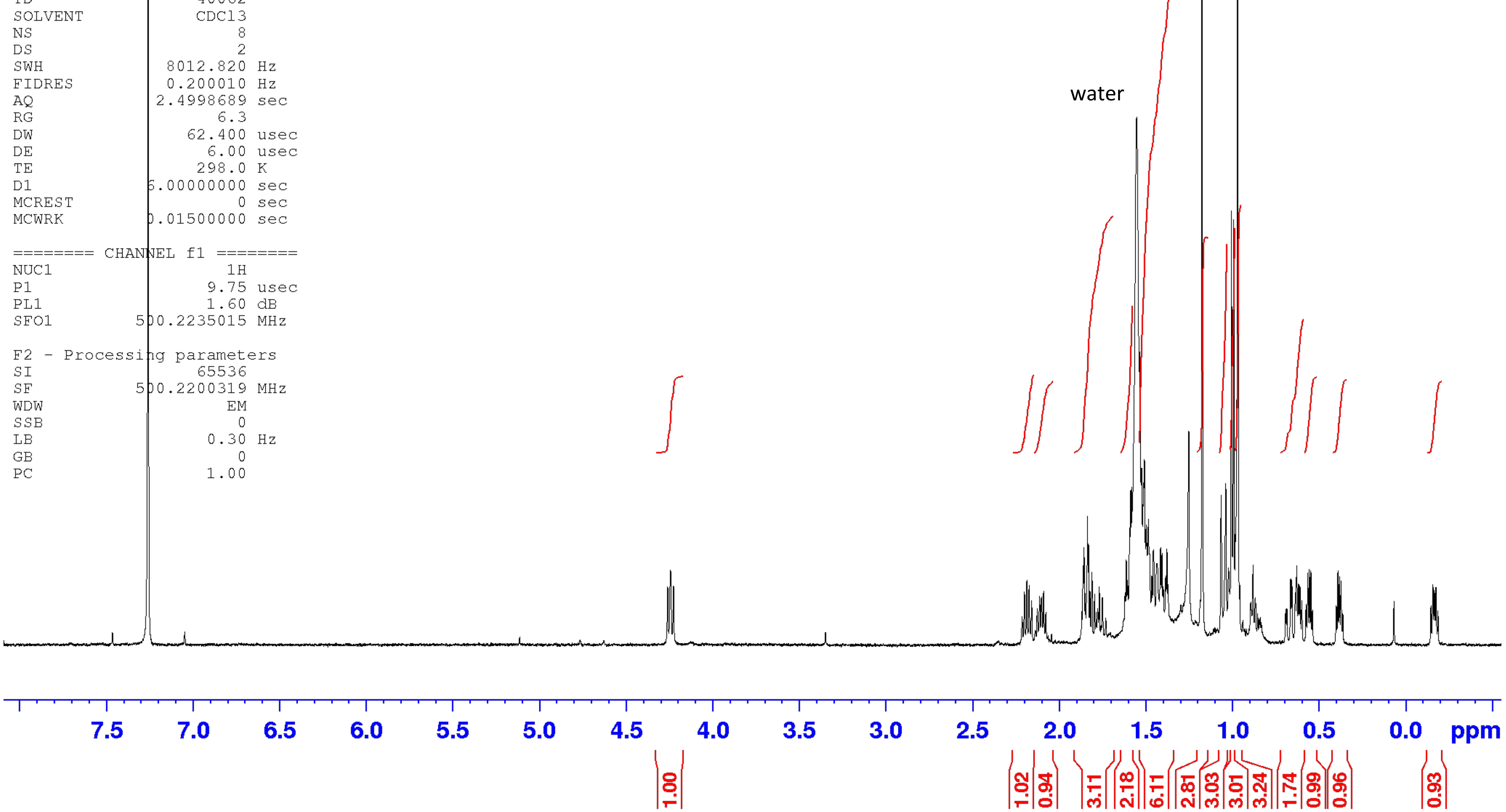

```
Current Data Parameters
NAME       JSCV-128-f6-17
EXPNO                   2
PROCNO                  1

F2 - Acquisition Parameters
Date_          20220415
Time              16.23
INSTRUM           av600
PROBHD   5 mm CPBBO BB-
PULPROG          zgdc30
TD                65536
SOLVENT           CDCl3
NS                  451
DS                    4
SWH           36231.883 Hz
FIDRES         0.552855 Hz
AQ            0.9043968 sec
RG                 2050
DW               13.800 usec
DE                19.63 usec
TE                298.0 K
D1           0.40000001 sec
D11          0.03000000 sec
TD0                   1

======== CHANNEL f1 ========
SFO1        150.9194080 MHz
NUC1                13C
P1                10.10 usec
PLW1        64.00000000 W

======== CHANNEL f2 ========
SFO2        600.1330010 MHz
NUC2                 1H
CPDPRG[2        waltz16
PCPD2             80.00 usec
PLW2        30.00000000 W
PLW12        0.37154001 W

F2 - Processing parameters
SI                65536
SF          150.9027927 MHz
WDW                  EM
SSB                   0
LB                 1.00 Hz
GB                    0
PC                 1.00
```

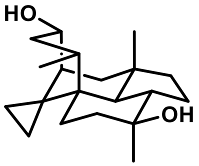

S24

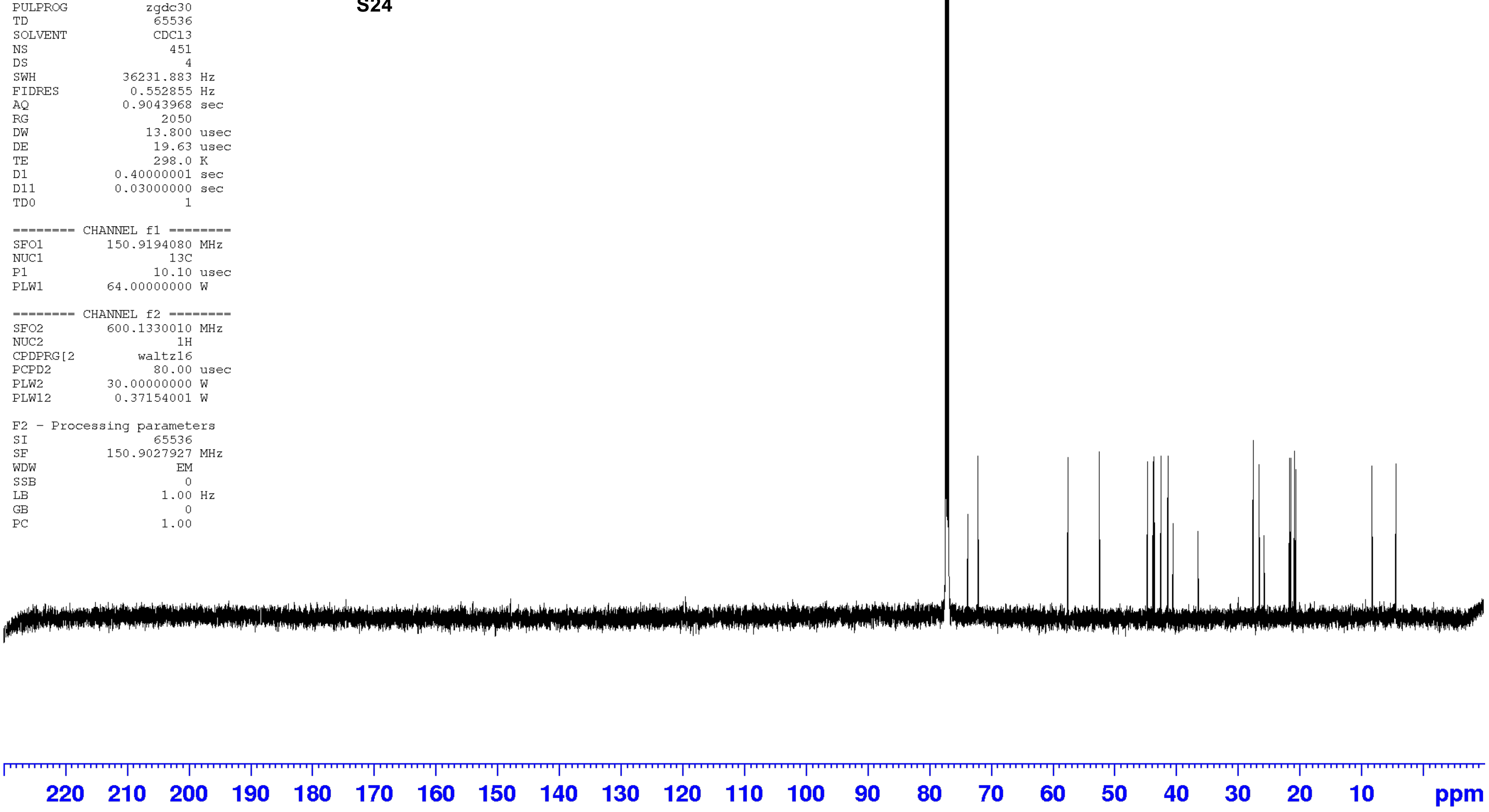

```
Current Data Parameters
NAME      JSCV-289B-f45-54
EXPNO                    1
PROCNO                   1

F2 - Acquisition Parameters
Date_             20221129
Time                 17.19
INSTRUM              av600
PROBHD   5 mm CPTCI 1H-
PULPROG               zg30
TD                   48074
SOLVENT              CDCl3
NS                      19
DS                       2
SWH               9615.385 Hz
FIDRES            0.200012 Hz
AQ               2.4998479 sec
RG                    2050
DW                  52.000 usec
DE                   14.54 usec
TE                   298.1 K
D1              6.00000000 sec
TD0                      1

======== CHANNEL f1 ========
SFO1           600.1342009 MHz
NUC1                    1H
P1                    8.00 usec
PLW1            6.16599989 W

F2 - Processing parameters
SI                   65536
SF             600.1300344 MHz
WDW                     EM
SSB                      0
LB                    0.30 Hz
GB                       0
PC                    1.00
```

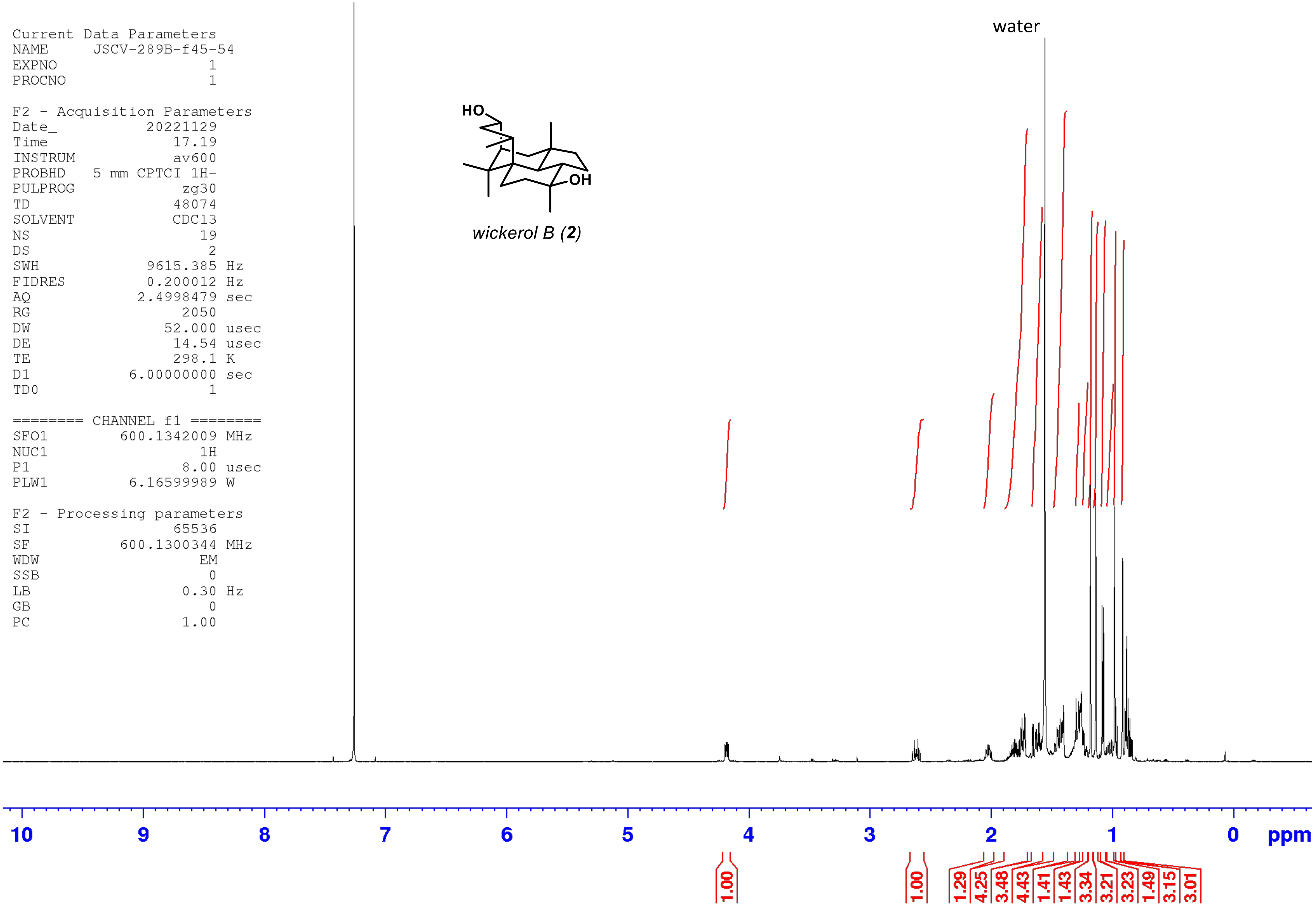

```
Current Data Parameters
NAME      JSCV-289B-f45-54
EXPNO                    2
PROCNO                   1

F2 - Acquisition Parameters
Date_             20221129
Time                 17.25
INSTRUM              av600
PROBHD   5 mm CPTCI 1H-
PULPROG  SpinEchopg30gp2.prdts
TD                  108692
SOLVENT              CDCl3
NS                     168
DS                      16
SWH              36231.883 Hz
FIDRES            0.333345 Hz
AQ               1.4999496 sec
RG                    2050
DW                  13.800 usec
DE                   18.44 usec
TE                   298.1 K
D1             4.00000000 sec
D11            0.03000000 sec
D16            0.00020000 sec
D17            0.00019600 sec
P2                   37.50 usec
TD0                      1

======== CHANNEL f1 ========
SFO1          150.9194080 MHz
NUC1                   13C
P1                   18.75 usec
P13                2000.00 usec
P26                 500.00 usec
PLW0                     0 W
PLW1          164.00000000 W
SPNAM[5]     Crp60comp.4
SPOAL5               0.500
SPOFFS5                  0 Hz
SPW5          92.25700378 W
SPNAM[8]  Crp60,0.5,20.1
SPOAL8               0.500
SPOFFS8                  0 Hz
SPW8          92.25700378 W

======== CHANNEL f2 ========
SFO2          600.1330010 MHz
NUC2                    1H
CPDPRG[2           waltz16
PCPD2                80.00 usec
PLW2            6.16599989 W
PLW12           0.04466800 W

====== GRADIENT CHANNEL =====
GPNAM[1]      SMSQ10.100
[illegible]
[illegible]     1000.00 usec

F2 - Processing parameters
SI                   65536
SF            150.9027931 MHz
WDW                     EM
SSB                      0
LB                    1.00 Hz
GB                       0
PC                    1.00
```

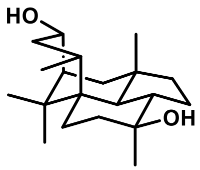

wickerol B (**2**)

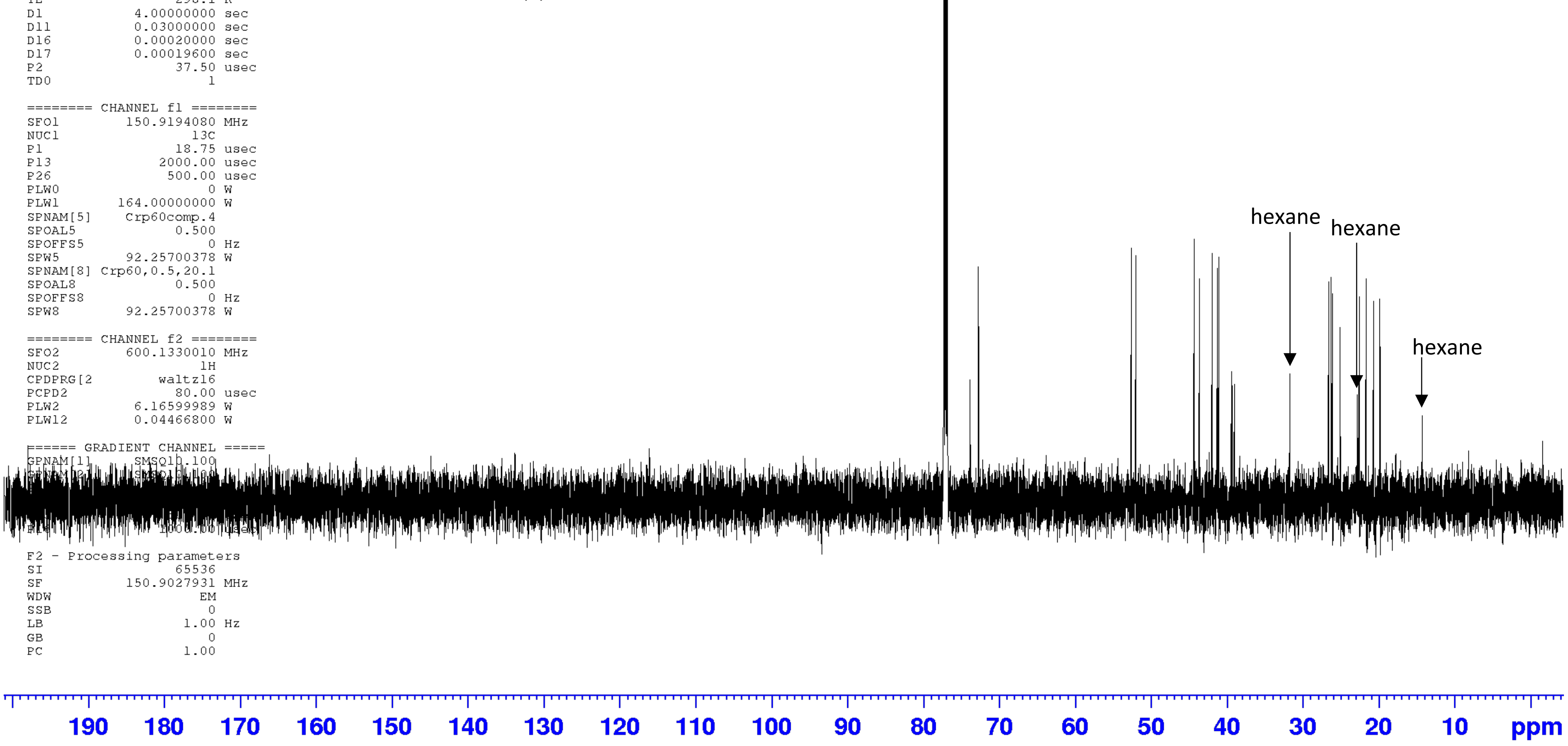

```
Current Data Parameters
NAME        JSCV-130B-f5-11-2
EXPNO                      1
PROCNO                     1

F2 - Acquisition Parameters
Date_               20220425
Time                   21.15
INSTRUM                av600
PROBHD    5 mm CPBBO BB-
PULPROG                 zg30
TD                     48072
SOLVENT                CDCl3
NS                        16
DS                         2
SWH                 9615.385 Hz
FIDRES              0.200020 Hz
AQ                 2.4997439 sec
RG                      20.2
DW                    52.000 usec
DE                     14.23 usec
TE                     298.0 K
D1                4.00000000 sec
TD0                        1

======== CHANNEL f1 ========
SFO1             600.1342009 MHz
NUC1                      1H
P1                      9.50 usec
PLW1             30.00000000 W

F2 - Processing parameters
SI                     65536
SF               600.1300343 MHz
WDW                       EM
SSB                        0
LB                      0.30 Hz
GB                         0
PC                      1.00
```

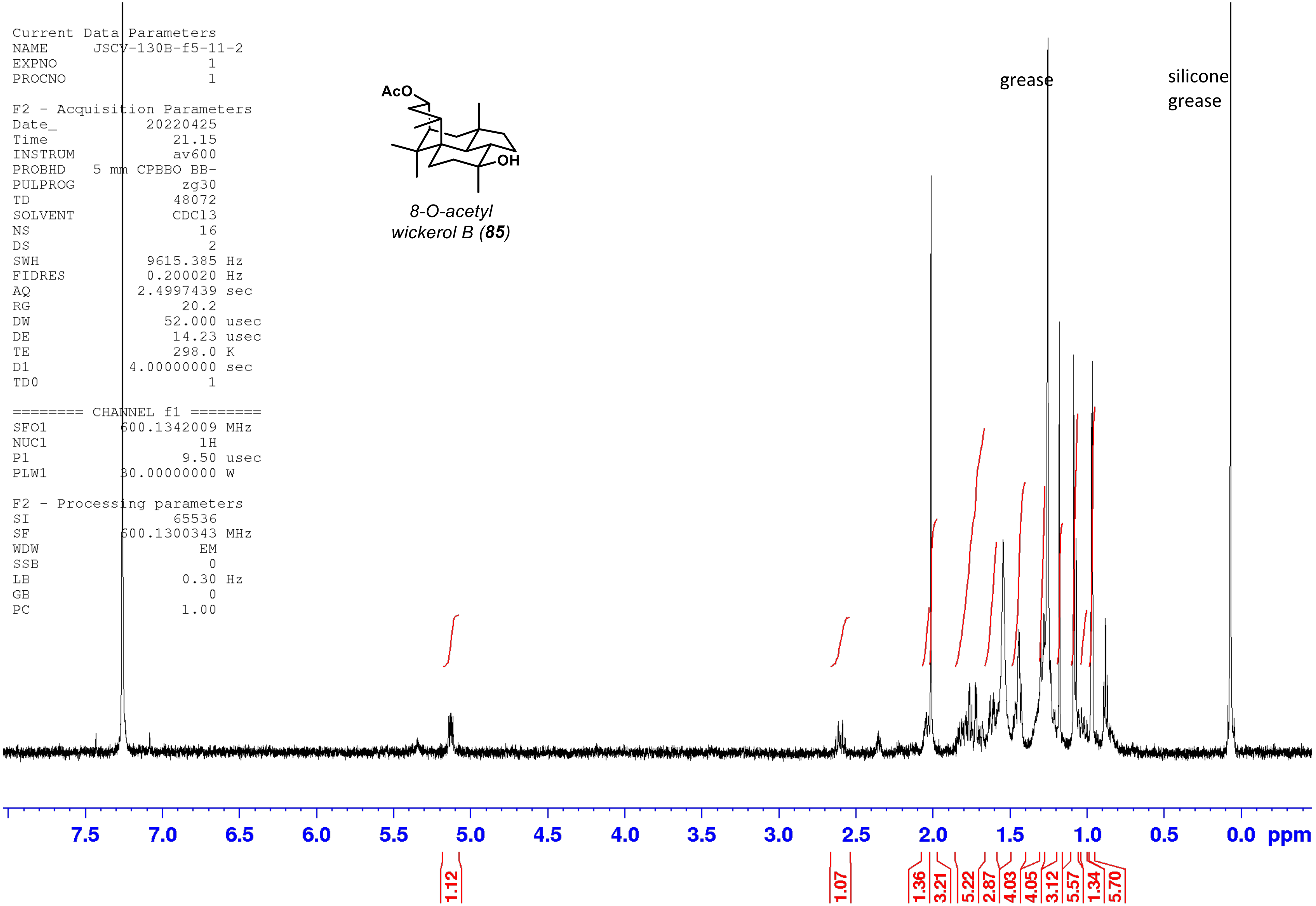

*8-O-acetyl wickerol B (**85**)*

```
Current Data Parameters
NAME       JSCV-130B-f5-11-2
EXPNO                      3
PROCNO                     1

F2 - Acquisition Parameters
Date_               20220426
Time                    5.53
INSTRUM                av600
PROBHD   5 mm CPBBO BB-
PULPROG              zgdc30
TD                     65536
SOLVENT                CDCl3
NS                     12288
DS                         4
SWH                36231.883 Hz
FIDRES              0.552855 Hz
AQ                 0.9043968 sec
RG                      2050
DW                    13.800 usec
DE                     19.63 usec
TE                     298.0 K
D1                1.50000000 sec
D11               0.03000000 sec
TD0                        1

======== CHANNEL f1 ========
SFO1          150.9194080 MHz
NUC1                     13C
P1                     10.10 usec
PLW1             64.00000000 W

======== CHANNEL f2 ========
SFO2          600.1330010 MHz
NUC2                      1H
CPDPRG[2             waltz16
PCPD2                  80.00 usec
PLW2             30.00000000 W
PLW12             0.37154001 W

F2 - Processing parameters
SI                     65536
SF            150.9027924 MHz
WDW                       EM
SSB                        0
LB                      1.00 Hz
GB                         0
PC                      1.00
```

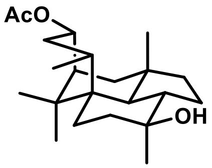

*8-O-acetyl wickerol B (**85**)*

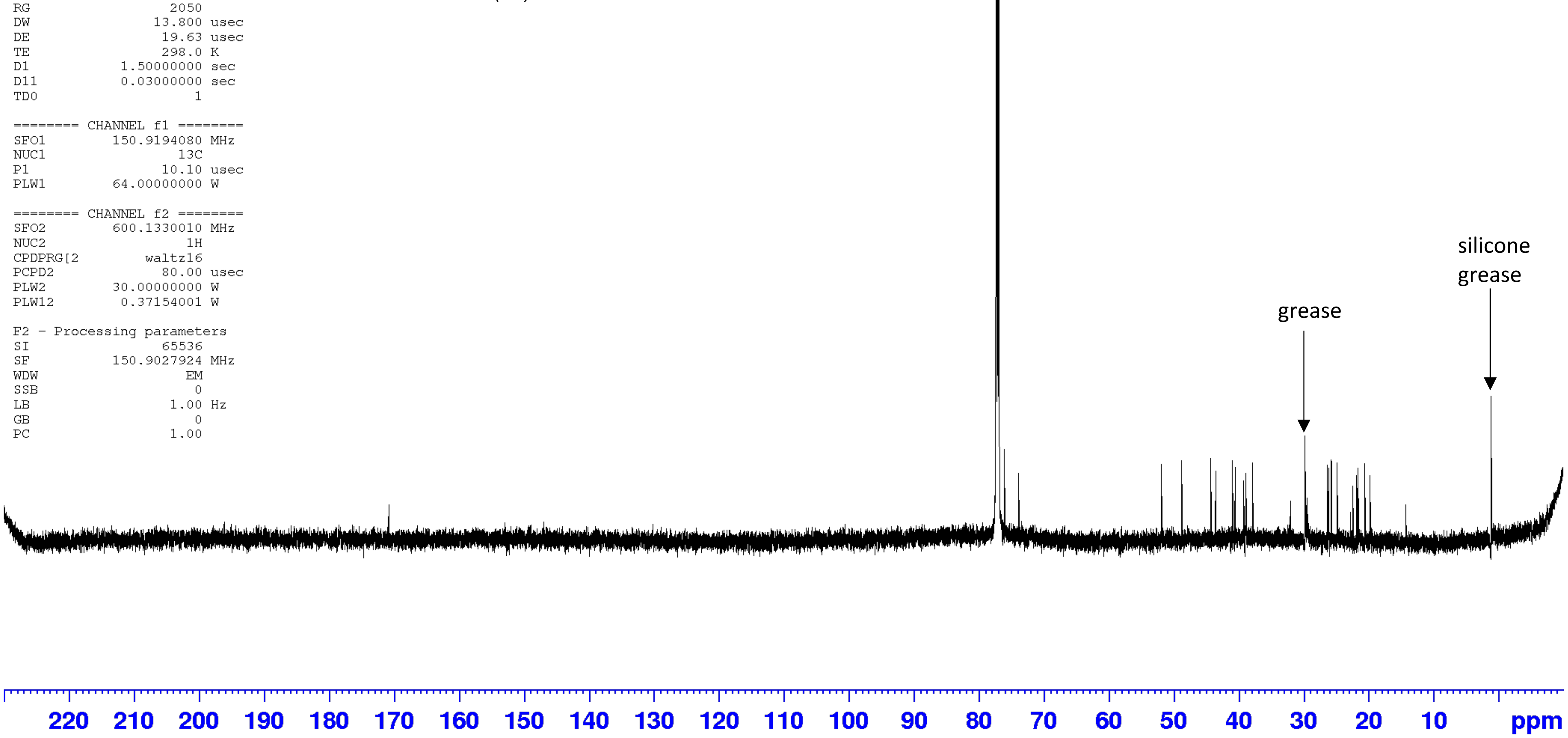

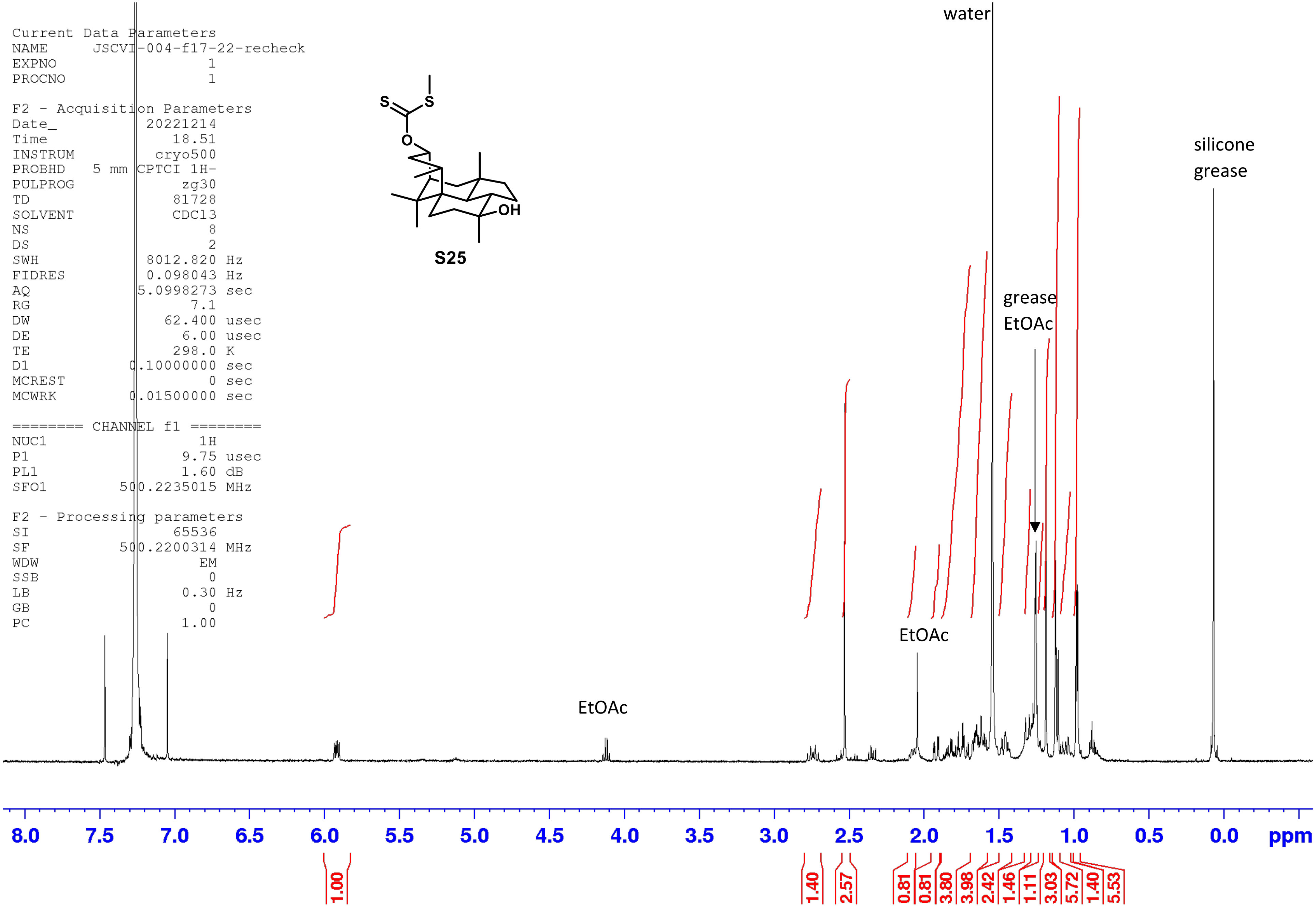
Current Data Parameters
NAME JSCVI-004-f17-22-recheck
EXPNO 1
PROCNO 1
F2 - Acquisition Parameters
Date_ 20221214
Time 18.51
INSTRUM cryo500
PROBHD 5 mm CPTCI 1H-
PULPROG zg30
TD 81728
SOLVENT CDCl3
NS 8
DS 2
SWH 8012.820 Hz
FIDRES 0.098043 Hz
AQ 5.0998273 sec
RG 7.1
DW 62.400 usec
DE 6.00 usec
TE 298.0 K
D1 0.10000000 sec
MCREST 0 sec
MCWRK 0.01500000 sec
======== CHANNEL f1 ========
NUC1 1H
P1 9.75 usec
PL1 1.60 dB
SFO1 500.2235015 MHz
F2 - Processing parameters
SI 65536
SF 500.2200314 MHz
WDW EM
SSB 0
LB 0.30 Hz
GB 0
PC 1.00
S25
water
silicone grease
grease EtOAc
EtOAc
EtOAc
8.0 7.5 7.0 6.5 6.0 5.5 5.0 4.5 4.0 3.5 3.0 2.5 2.0 1.5 1.0 0.5 0.0 ppm
1.00
1.40
2.57
0.81
0.81
3.80
3.98
2.42
1.46
1.11
3.03
5.72
1.40
5.53

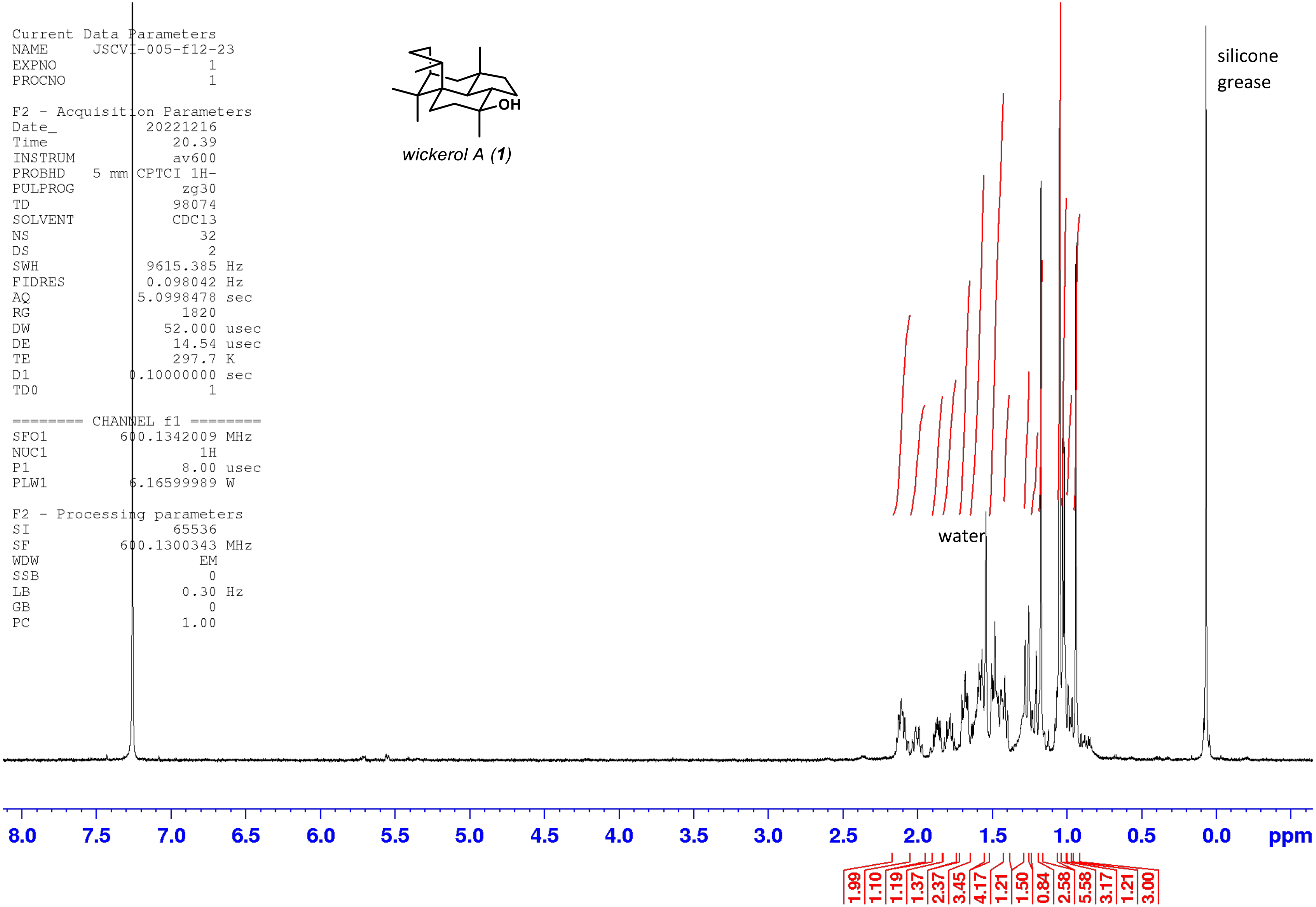

Current Data Parameters
NAME JSCVI-005-f12-23
EXPNO 1
PROCNO 1
F2 - Acquisition Parameters
Date_ 20221216
Time 20.39
INSTRUM av600
PROBHD 5 mm CPTCI 1H-
PULPROG zg30
TD 98074
SOLVENT CDCl3
NS 32
DS 2
SWH 9615.385 Hz
FIDRES 0.098042 Hz
AQ 5.0998478 sec
RG 1820
DW 52.000 usec
DE 14.54 usec
TE 297.7 K
D1 0.10000000 sec
TD0 1
======== CHANNEL f1 ========
SFO1 600.1342009 MHz
NUC1 1H
P1 8.00 usec
PLW1 6.16599989 W
F2 - Processing parameters
SI 65536
SF 600.1300343 MHz
WDW EM
SSB 0
LB 0.30 Hz
GB 0
PC 1.00
OH
wickerol A (1)
silicone grease
water
8.0 7.5 7.0 6.5 6.0 5.5 5.0 4.5 4.0 3.5 3.0 2.5 2.0 1.5 1.0 0.5 0.0 ppm
1.99 1.10 1.19 1.37 2.37 3.45 4.17 1.21 1.50 0.84 2.58 5.58 3.17 1.21 3.00

Current Data Parameters
NAME JSCVI-005-f12-23
EXPNO 2
PROCNO 1

F2 - Acquisition Parameters
Date_ 20221216
Time 20.46
INSTRUM av600
PROBHD 5 mm CPTCI 1H-
PULPROG SpinEchopg30gp2.prdts
TD 72460
SOLVENT CDCl3
NS 6144
DS 16
SWH 36231.883 Hz
FIDRES 0.500026 Hz
AQ 0.9999480 sec
RG 2050
DW 13.800 usec
DE 18.44 usec
TE 297.7 K
D1 5.00000000 sec
D11 0.03000000 sec
D16 0.00020000 sec
D17 0.00019600 sec
P2 37.50 usec
TD0 1

======== CHANNEL f1 ========
SFO1 150.9194080 MHz
NUC1 13C
P1 18.75 usec
P13 2000.00 usec
P26 500.00 usec
PLW0 0 W
PLW1 164.00000000 W
SPNAM[5] Crp60comp.4
SPOAL5 0.500
SPOFFS5 0 Hz
SPW5 92.25700378 W
SPNAM[8] Crp60,0.5,20.1
SPOAL8 0.500
SPOFFS8 0 Hz
SPW8 92.25700378 W

======== CHANNEL f2 ========
SFO2 600.1330010 MHz
NUC2 1H
CPDPRG[2 waltz16
PCPD2 80.00 usec
PLW2 6.16599989 W
PLW12 0.04466800 W

====== GRADIENT CHANNEL =====
GPNAM[1] SMSQ10.100
GPNAM[2] SMSQ10.100
GPZ1 30.00 %
GPZ2 50.00 %
P15 500.00 usec
P16 1000.00 usec

F2 - Processing parameters
[illegible]
SSB 0
LB 1.00 Hz
GB 0
PC 1.00

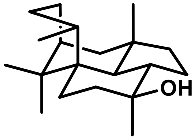

*wickerol A (**1**)*

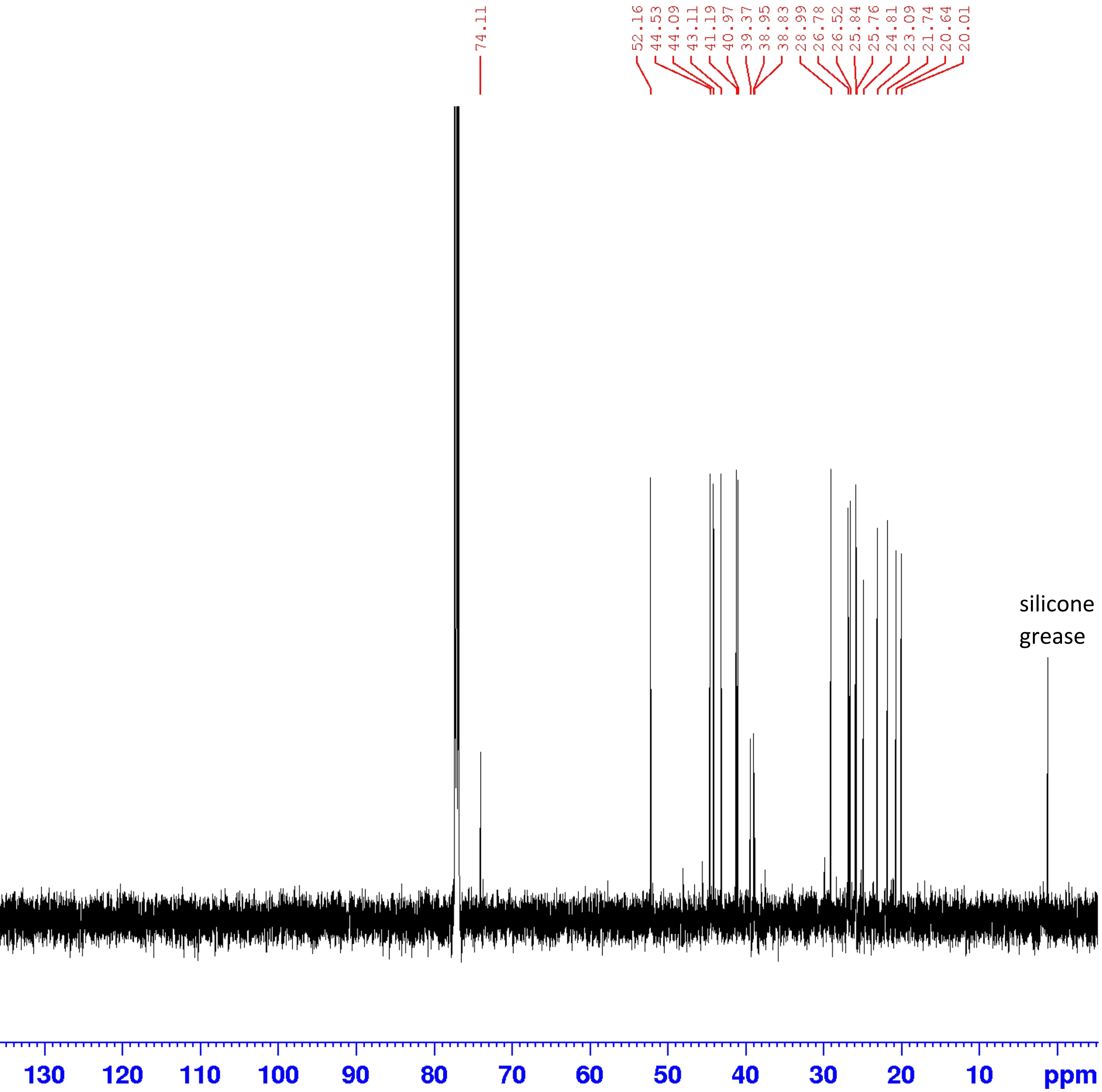

*Comparison of Spectra Data – wickerol A (**1**)*

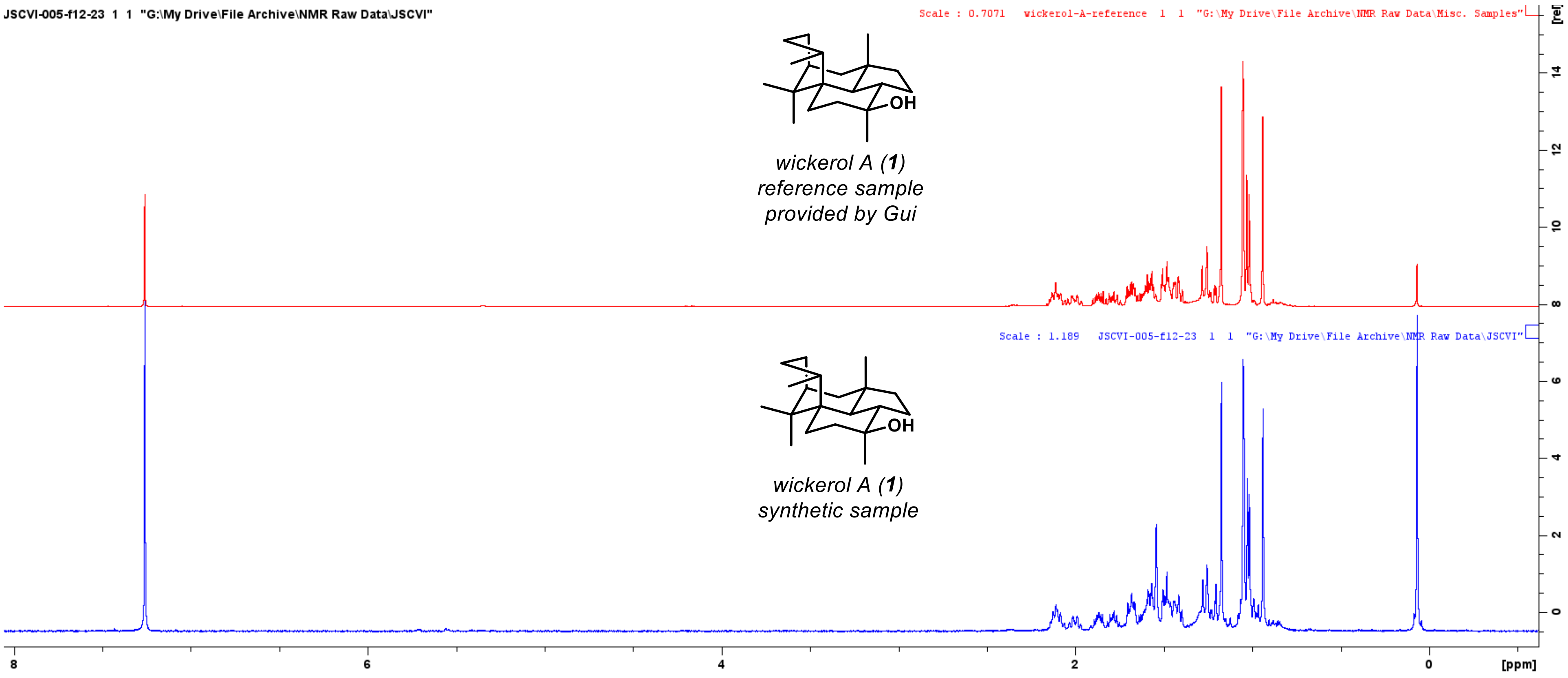

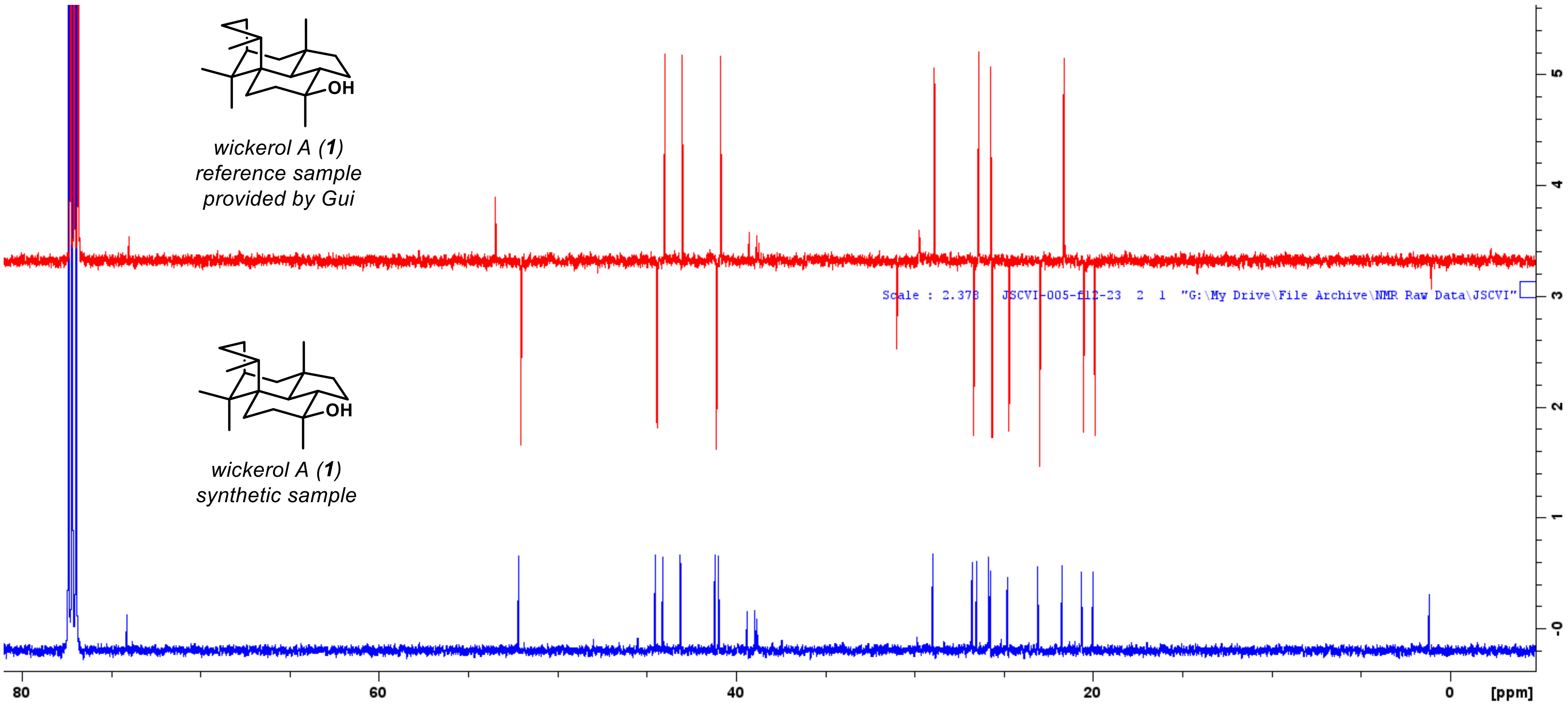
OH
wickerol A (1)
reference sample
provided by Gui
OH
wickerol A (1)
synthetic sample
Scale : 2.378 JSCVI-005-f12-23 2 1 "G:\My Drive\File Archive\NMR Raw Data\JSCVI"
80
60
40
20
0
[ppm]
5
4
3
2
1
0

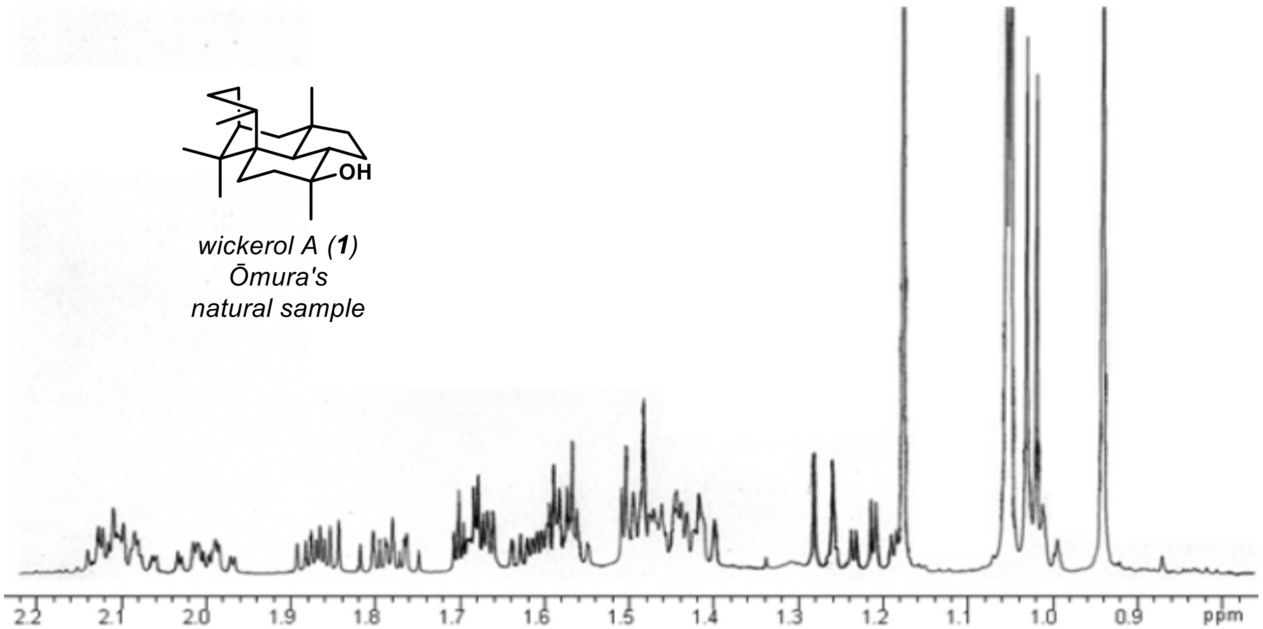
OH
wickerol A (**1**)
Ōmura's
natural sample
2.2 2.1 2.0 1.9 1.8 1.7 1.6 1.5 1.4 1.3 1.2 1.1 1.0 0.9 ppm

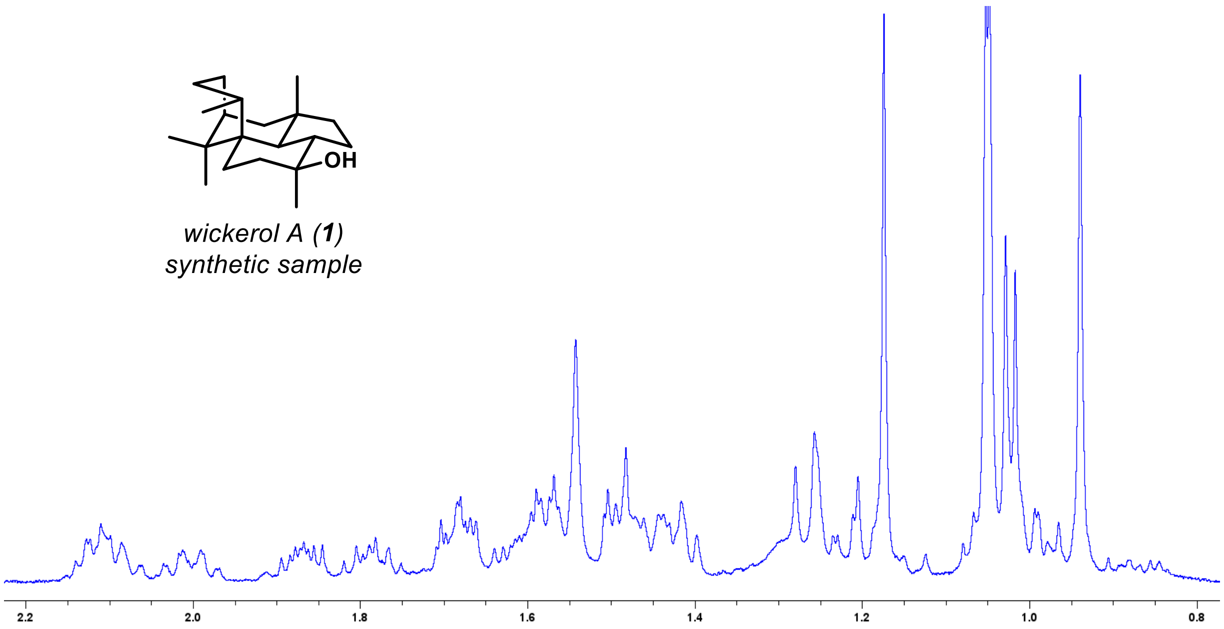
OH
wickerol A (**1**)
synthetic sample
2.2 2.0 1.8 1.6 1.4 1.2 1.0 0.8

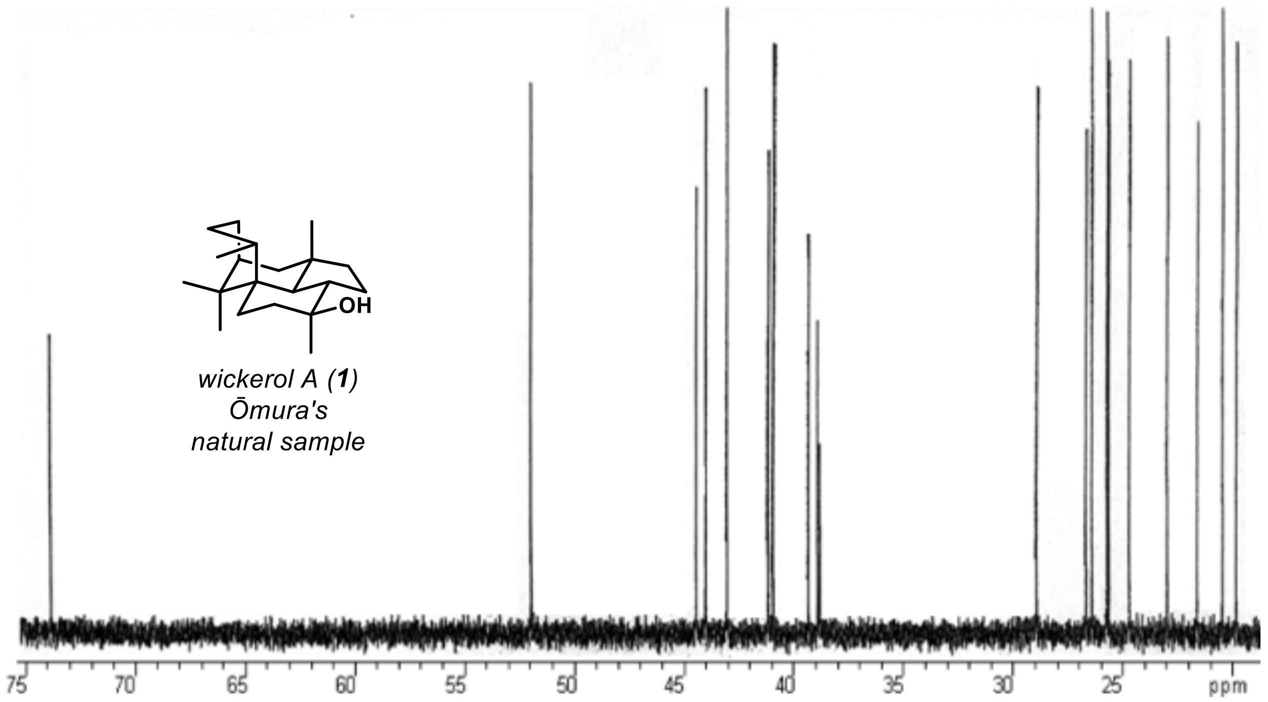
OH
wickerol A (**1**)
Ōmura's
natural sample
75
70
65
60
55
50
45
40
35
30
25
ppm

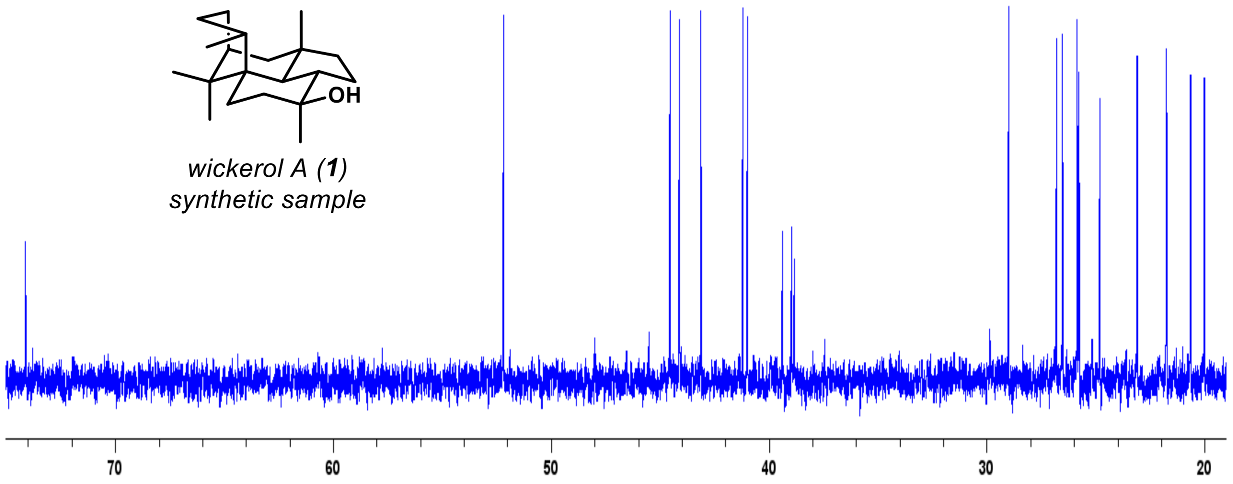
OH
wickerol A (**1**)
synthetic sample
70
60
50
40
30
20

*<u>Comparison of Spectra Data – wickerol B (**2**)</u>*

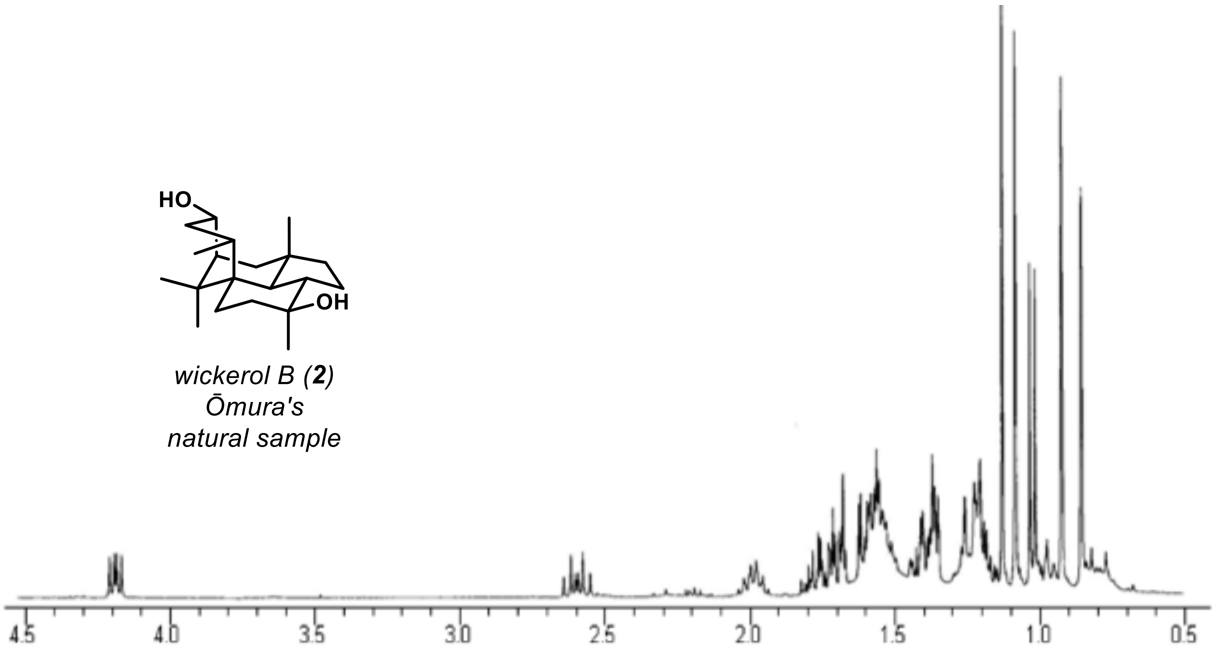

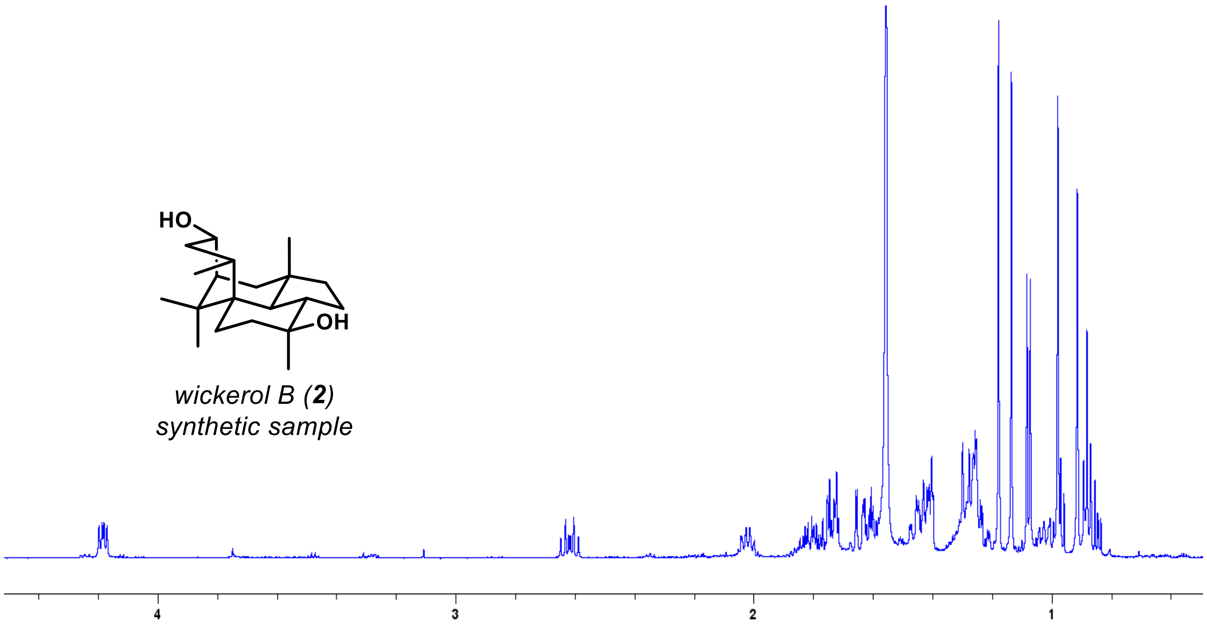

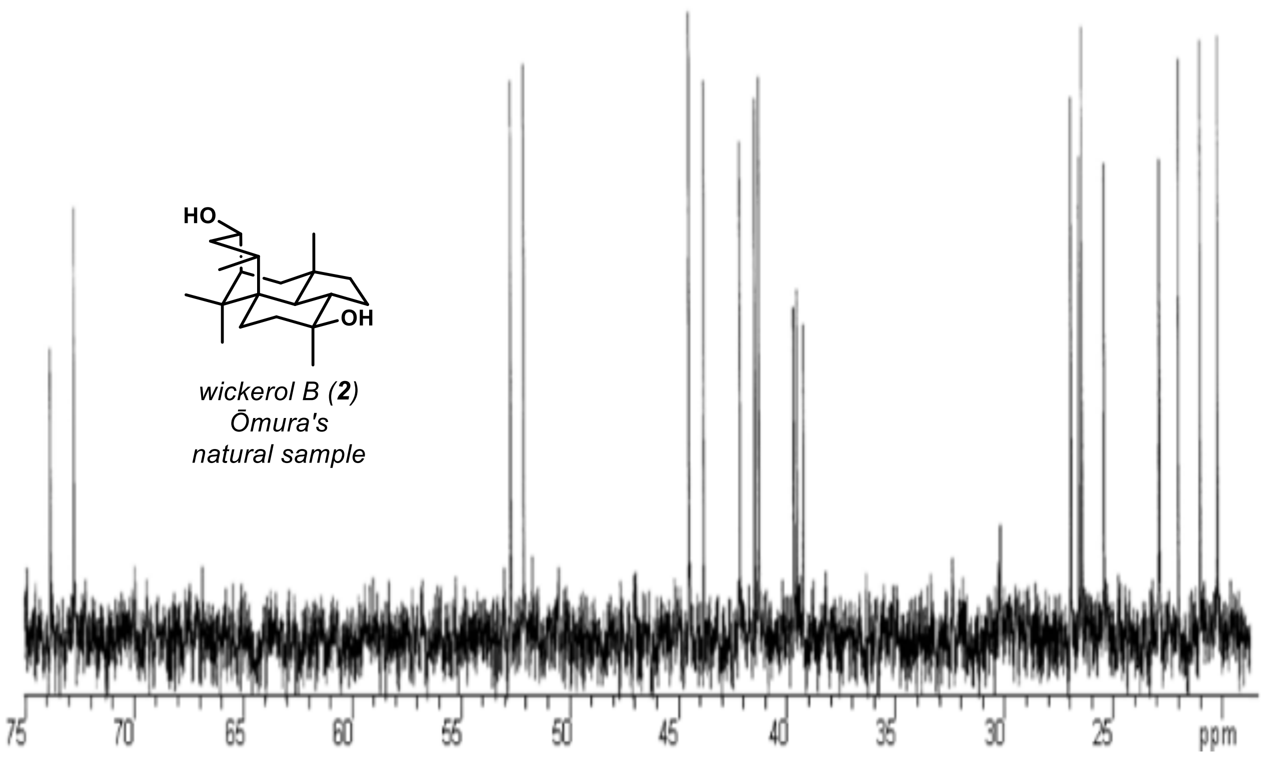

wickerol B (**2**)
Ōmura's
natural sample

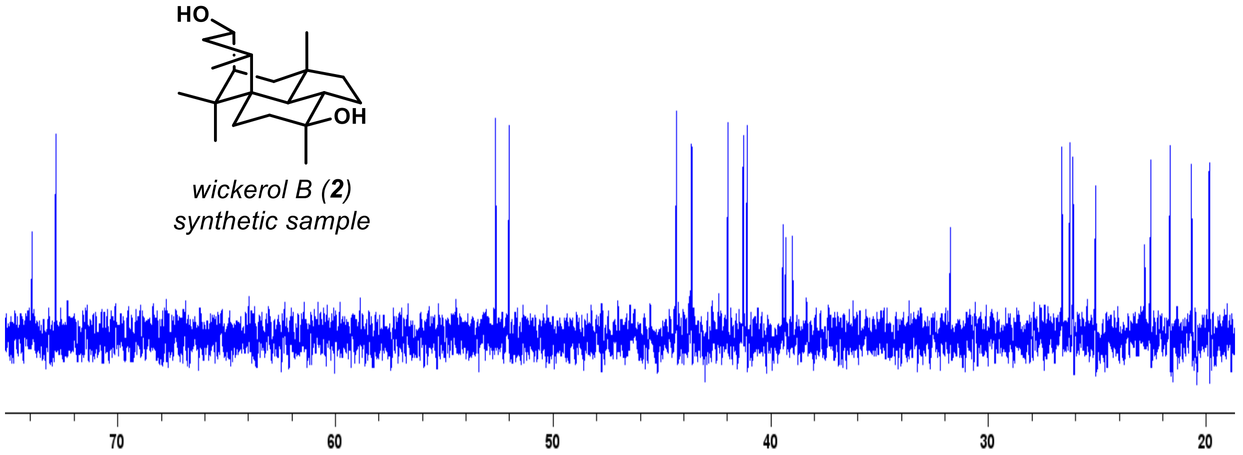

wickerol B (**2**)
synthetic sample

*Comparison of Spectral Data – 8-O-acetyl wickerol B (**85**)*

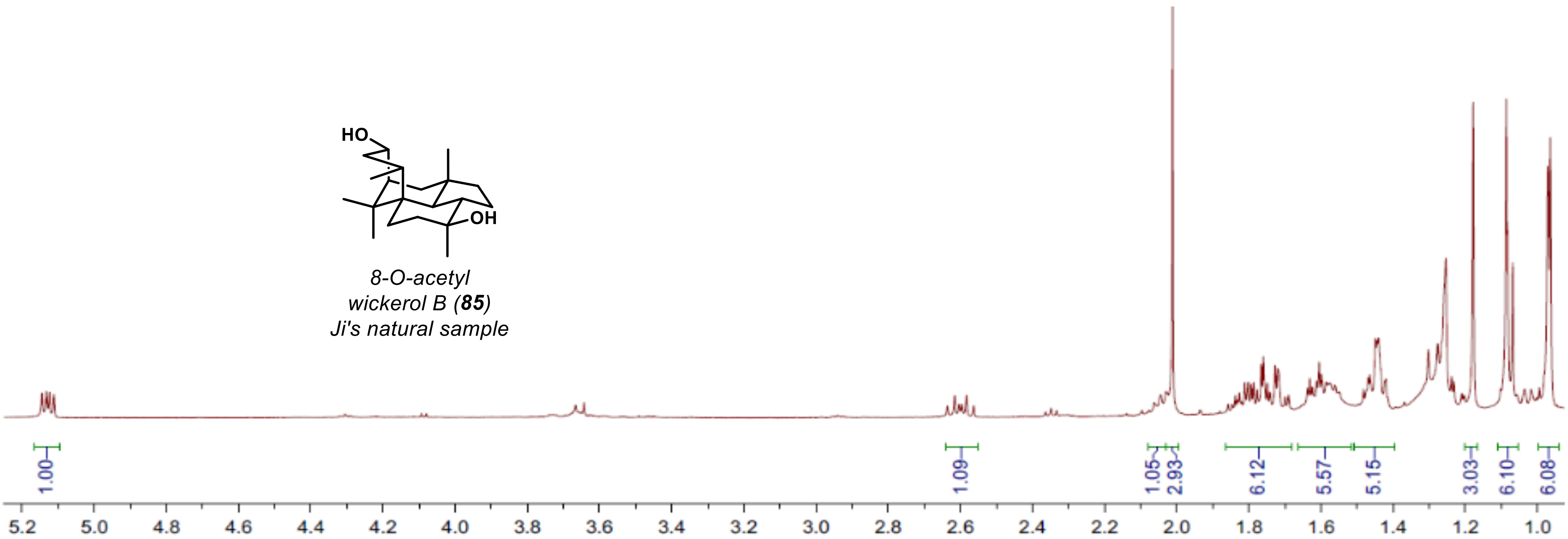

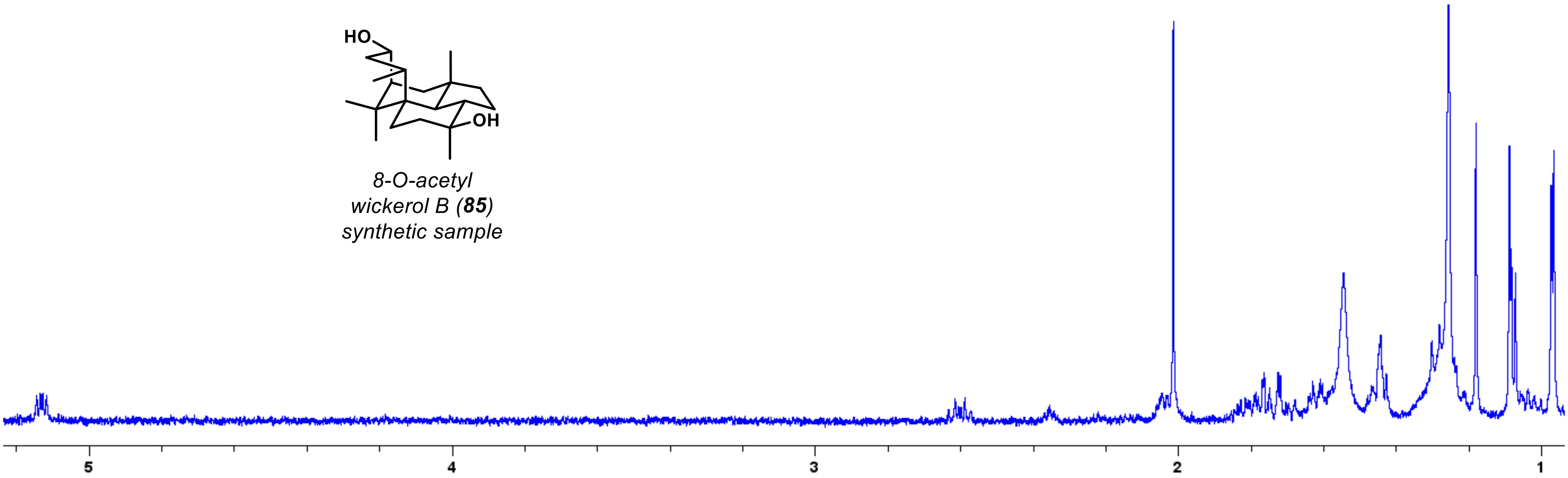

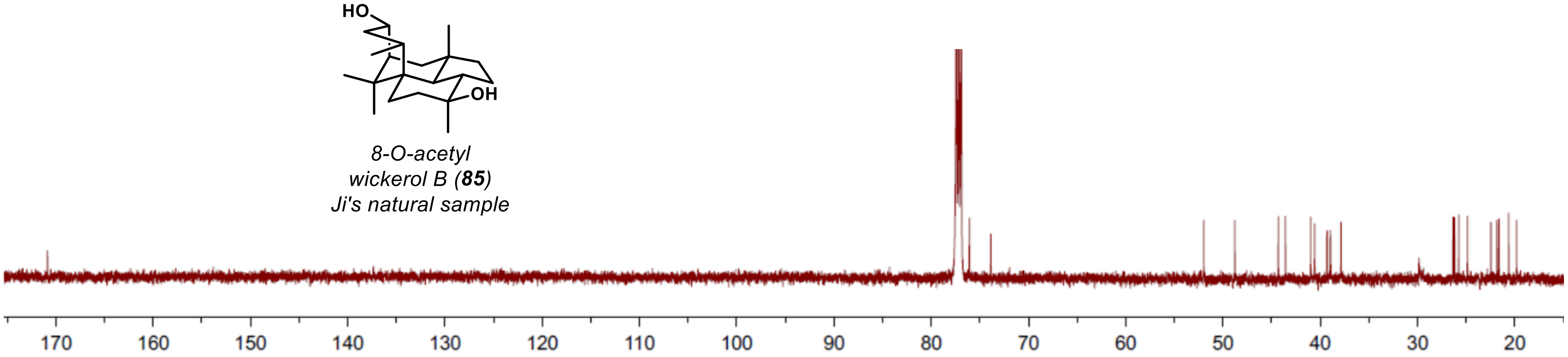

8-O-acetyl
*wickerol B (**85**)*
Ji's natural sample

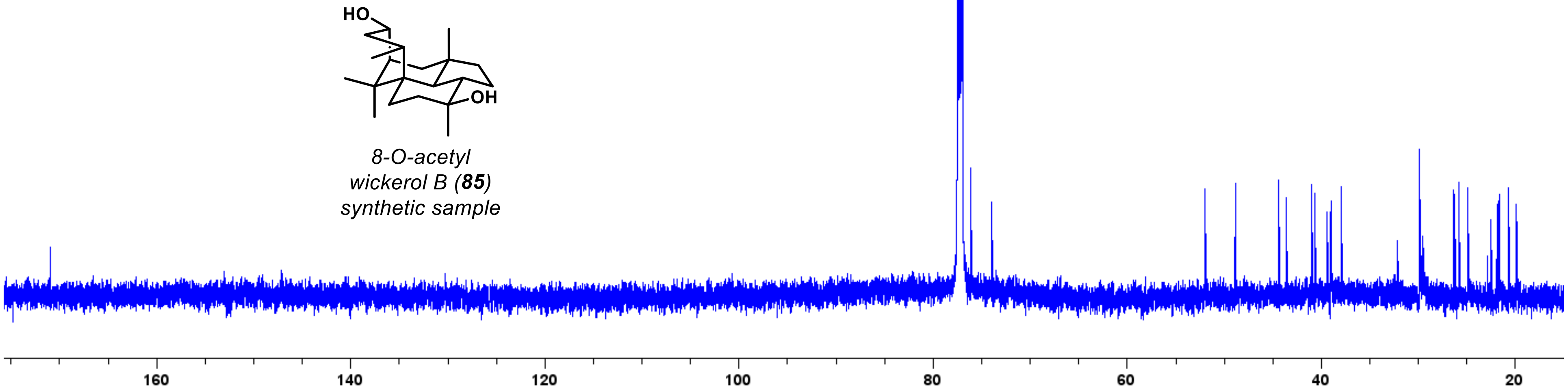

8-O-acetyl
*wickerol B (**85**)*
synthetic sample
